# Comparative efficacy and tolerability of nutraceuticals for depressive disorder: A systematic review and network meta-analysis

**DOI:** 10.1017/S0033291725000996

**Published:** 2025-05-02

**Authors:** Ying-Chih Cheng, Wei-Lieh Huang, Wen-Yin Chen, Yu-Chen Huang, Po-Hsiu Kuo, Yu-Kang Tu

**Affiliations:** 1 Department of Psychiatry, China Medical University Hsinchu Hospital, China Medical University, Hsinchu, Taiwan; 2 Institute of Epidemiology and Preventive Medicine, College of Public Health, National Taiwan University, Taipei, Taiwan; 3 Research Center of Big Data and Meta-analysis, Wan Fang Hospital, Taipei Medical University, Taipei, Taiwan; 4 Department of Psychiatry, National Taiwan University Hospital, Yunlin, Taiwan; 5 Department of Psychiatry, National Taiwan University Hospital, Taipei, Taiwan; 6 Department of Psychiatry, College of Medicine, National Taiwan University, Taipei, Taiwan; 7 Graduate Institute of Clinical Medicine, College of Medicine, National Taiwan University, Taipei, Taiwan; 8 Department of Psychiatry, Taipei City Psychiatric Center, Taipei City Hospital, Taipei, Taiwan; 9 School of Medicine, College of Medicine, Fu Jen Catholic University, New Taipei City, Taiwan; 10 Department of Dermatology, School of Medicine and College of Medicine, Taipei Medical University, Taipei, Taiwan; 11 Department of Dermatology, Wan Fang Hospital, Taipei Medical University, Taipei, Taiwan; 12 Department of Public Health, College of Public Health, National Taiwan University, Taipei, Taiwan; 13 Health Behaviors and Community Sciences, College of Public Health, National Taiwan University, Taipei, Taiwan; 14 Psychiatric Research Center, Wan Fang Hospital, Taipei Medical University, Taipei, Taiwan; 15 Department of Dentistry, National Taiwan University Hospital, Taipei, Taiwan

**Keywords:** depression, network meta-analysis, nutraceuticals, randomized controlled trials

## Abstract

**Background:**

Nutraceuticals have been taken as an alternative and add-on treatment for depressive disorders. Direct comparisons between different nutraceuticals and between nutraceuticals and placebo or antidepressants are limited. Thus, it is unclear which nutraceuticals are the most efficacious.

**Methods:**

We conducted a network meta-analysis to estimate the comparative efficacy and tolerability of nutraceuticals for the treatment of depressive disorder in adults. The primary outcome was the change in depressive symptoms, as measured by the standard mean difference (SMD). Secondary outcomes included response rate, remission rate, and anxiety. Tolerability was defined as all-cause discontinuation and adverse events. Frequentist random-effect NMA was conducted.

**Results:**

Hundred and ninety-two trials involving 17,437 patients and 44 nutraceuticals were eligible for inclusion. Adjunctive nutraceuticals consistently showed better efficacy than antidepressants (ADT) alone in outcomes including SMD, remission, and response. Notable combinations were Eicosapentaenoic acid + Docosahexaenoic Acid plus ADT (EPA + DHA + ADT) (SMD 1.04, 95% confidence interval 0.64–1.44), S-Adenosyl Methionine (SAMe) + ADT (0.99, 0.31–1.68), curcumin + ADT (1.03, 0.55–1.51), Zinc + ADT (1.59, 0.63–2.55), tryptophan + ADT (1.24, 0.32–2.16), and folate + ADT (0.64, 0.17–1.10). Additionally, four nutraceutical monotherapies demonstrated superior efficacy compared to ADT: EPA + DHA (0.6, 0.32–0.88), SAMe (0.52, 0.18–0.87), curcumin (0.62, −0.17 to 1.40) and saffron (0.69, 0.34–1.04). It is noted that EPA + DHA, SAMe, and curcumin showed strong performance as either monotherapies or adjuncts to ADT. Most nutraceuticals showed comparable tolerability to placebo.

**Conclusions:**

This extensive systematic review and NMA of nutraceuticals for treating depressive disorders indicated a number of nutraceuticals that could offer benefits, either as adjuncts or monotherapies.

## Introduction

Depression is estimated to affect the mental well-being of more than 264 million people annually and has the highest number of years lived with disability among several types of diseases (World Health Organization, 2017). Depression is a major medical and economic burden and would increase the risk of suicide (Lépine & Briley, 2011). Therefore, appropriate treatment of depression is critical. Current evidence-based treatment options for depression include antidepressant treatment (ADT), electroconvulsive therapy, repetitive transcranial magnetic stimulation (rTMS), and psychotherapy (Bauer et al., 2013). However, the treatment outcomes are not always satisfactory. Pharmacotherapy is usually the first choice, but response to ADT is limited in more than half of patients with depression. The quality of psychotherapy depends on the experience of the therapist. In addition, not all people have access to and can afford new techniques such as rTMS.

It has been estimated that approximately 40% of patients have not achieved remission after treatment with multiple ADTs (Rush et al., 2004). Moreover, adverse effects are common in patients receiving ADTs, thus leading to discontinuation of ADT treatment (Bull et al., 2002). Therefore, nutraceuticals are a common alternative (with or without standard ADTs) for patients experiencing emotional disturbances (Firth et al., 2019a). Nutraceuticals are defined as food or part of food that provides health benefits. The use of nutraceuticals among psychiatric patients has increased rapidly (Firth et al., 2019b). Recent studies have demonstrated the potential beneficial effects of nutraceuticals, such as omega-3 fatty acids, S-adenosyl-L-methionine (SAMe), folic acid, and vitamin D, on depression (Freeman et al., 2010, Sarris et al., 2016). Guidelines for prescribing omega-3 fatty acids for depression have also recently been developed, suggesting that 1–2 g of EPA is recommended for adjunctive use in depression (Guu et al., 2019). Additionally, clinical guidelines have indicated that other nutraceuticals such as vitamin D at doses between 1500 and 4000 international units (*IU*), zinc at dose of 25 mg, and probiotics at doses of 1–10 billion units CFU (colony-forming units) may be effective in the adjunctive treatment of depression (Sarris et al., 2022).

Many randomized controlled trials (RCTs) have investigated the effects of dozens of nutraceuticals on patients with depression (Firth et al., 2019b, Sarris et al., 2022). However, a high degree of heterogeneity was noted in study designs, patient recruitment criteria, and outcome assessment among these trials, rendering the interpretation of these findings challenging. Traditional pairwise meta-analyses conducted to evaluate the efficacy of specific nutraceuticals have often generated inconclusive results (Sarris et al., 2016). The most effective nutraceuticals remain unknown because many nutraceuticals have not been directly compared. Furthermore, it remains unclear whether nutraceuticals used as an adjunct to ADT or combination of nutraceuticals provide additional benefits. We, therefore, conducted a systematic review of RCTs and a network meta-analysis (NMA) and a component network meta-analysis (CNMA) to evaluate the efficacy of nutraceuticals as monotherapies or adjuncts to ADT in adult patients diagnosed with depressive disorder.(Cipriani et al., 2018, Huhn et al., 2019) The primary outcome was the efficacy of nutraceuticals in reducing depressive symptoms. The secondary outcomes were response rate, remission rate, all-cause discontinuation, and adverse reactions.

## Methods

### Search strategy and selection criteria

The systematic review and NMA was conducted according to the Preferred Reporting Items for Systematic Reviews and Meta-Analyses extension statement for NMA. The protocol for this review has been registered in the International Prospective Register of Systematic Reviews PROSPERO (CRD42020151158).

The search terms were constructed on the basis of the patient population (e.g. depressive disorder) and intervention (e.g. nutraceutical). We searched for ‘depress*’ OR ‘depression*’ OR ‘dysthymia*’ combined with a list of all included nutraceuticals on PubMed, EMBASE, MEDLINE, PsycINFO, CINAHL, and Cochrane Central Register of Controlled Trails (CENTRAL) from the date of their inception to December 2024, with no language restrictions. Nutraceuticals were defined as any nutritional supplements with beneficial effects on depressive symptoms, being added to standard care, regardless of their dosage and pharmaceutical form, alone or in combination, for example, vitamins, minerals, or herbs (i.e. fatty acids, amino acids, probiotics, Saint John’s wort (SJW) and curcumin). Studies involving traditional Chinese medicine were excluded, because these studies often involve complex multi-component formulations and have more complicated mechanisms of action. Because no standard classification of nutraceuticals is available, nutraceuticals with different chemical compositions are defined as different interventions.

We included RCTs with patients (1) diagnosed with a depressive disorder according to the standard operationalized diagnostic criteria (DSM-III, DSM-III-R, DSM-IV, DSM-IV-TR, DSM-5, ICD-10), who were current users of ADT or who had depressive symptoms above the threshold according to validated scales; (2) randomized to one of the treatment groups; (3) included in at least one treatment group that received one nutraceutical as a monotherapy or an adjunct to an ADT or received two or more nutraceuticals; and (4) aged ≥18 years. All ADT were considered the same intervention group. We excluded most crossover trials, but if they provided efficacy data before the crossover, the data from the first period of crossover trials were eligible for inclusion. Trials were excluded if they were designed to investigate relapse prevention or treatment discontinuation, if they did not report the target outcome, if they did not randomize patients to different treatments, or if none of the treatment groups received nutraceuticals. In the case of duplicate data, only studies with more detailed information and larger sample sizes were included.

### Data extraction and quality assessment

Two authors (YCC, WLH) independently extracted data regarding study characteristics, patient characteristics, interventions, outcomes, and other relevant findings from the published studies. Results were compared between the two reviewers, and discrepancies were resolved through discussion. If discrepancies remained, other authors were consulted to reach a final decision. Version 2 of the Cochrane Collaboration’s risk of bias assessment tool (RoB2) (Sterne et al., 2019) was used to assess the quality of included studies. Two other reviewers validated all extracted data (WYC and YCH), and any discrepancies were resolved through consensus. The confidence in the estimates was determined by integrating the overall rating of each study through the use of the Confidence In Network Meta-Analysis (CINeMA) approach (Nikolakopoulou et al., 2020).

### Outcomes

The primary outcome was treatment efficacy measured by the mean change in scores on the standard observer rating scale for depression (e.g. Hamilton Rating Scale for Depression [HAMD], Montgomery–Asberg Depression Rating Scale, Edinburgh Postnatal Depression Scale) or the mean changes in scores on depression scales rated by patients (e.g. Beck Depression Inventory or Depression Scale von Zerssen). Whenever possible, we used the intention-to-treat principle in the statistical analyses. When depressive symptoms were measured using more than one standard rating scale, a predefined hierarchy of scales (Supplementary Appendix 8, p. 90) was employed to determine which scale should be used. For all analyses, the outcomes reported at 8 weeks after treatment were recorded. If no information was available at 8 weeks, the outcome reported at the time point closest to 8 weeks was recorded. If two time points were equidistant, the one with a longer follow-up period was selected. When multiple doses of the same nutraceuticals were compared within the same trial, their results were combined by calculating a weighted average of the changes in the outcome and a pooled variance. Secondary outcomes included the response rate (measured by the proportion of patients who achieved a reduction of ≥50% in depression rating scores), the remission rate (measured by the proportion of patients who had depression scores below the remission cutoff value of each assessed scale), and anxiety (measured by the mean change in anxiety scale scores). The safety profile was assessed on the basis of all-cause discontinuation (measured by the number of patients who withdrew from the trial for any reason) and adverse event prevalence (measured by the proportion of patients experiencing at least one adverse event).

### Statistical analysis

We conducted the frequentist random-effect NMA. The relative treatment effects between the two interventions are expressed as the standard mean difference (SMD; Cohen’s d) for continuous outcomes and odds ratio (OR) for dichotomous outcomes with a 95% confidence interval (CI). The distribution and geometry of the evidence were examined by producing a network plot with node sizes proportional to the number of study participants and the width of edges proportional to the number of studies comparing the two treatments.

Transitivity, a key assumption in NMA, requires that included studies are sufficiently similar regarding the distribution of effect modifiers, ensuring valid indirect comparisons between treatments (Salanti, 2012). We assessed this by examining how major effect modifiers were distributed across the network. Consistency between direct and indirect evidence for each pair of treatments in the network was assessed globally using the design-by-treatment interaction model and locally using the node-splitting model (Higgins et al., 2012). The node-splitting model identifies inconsistencies by comparing direct evidence with indirect evidence derived from the network, highlighting potential areas of disagreement (Dias, Welton, Cladwell, & Ades, 2010).

We assessed global statistical heterogeneity across all comparisons by using the *I* ^2^ statistic, which ranges from 0% to 100% with 25%–49%, 50%–74%, and 75% or more being considered low, moderate, and high levels of heterogeneity, respectively (Higgins, Thompson, Deeks, & Altman, 2003). We used the comparison-adjusted funnel plot and Egger’s test to detect any small study effect by first arranging the treatments according to their ranking (Peters et al., 2008).

Treatments were ranked based on their *p*-scores, which range between 0 and 1. *p*-scores, derived from the point estimates and standard errors of relative treatment effects, indicate the level of certainty that one treatment is superior to another treatment, averaged across all competing treatments (Rücker and Schwarzer, 2015). Higher scores suggest a higher probability of one treatment being better than others in the network. P-scores provide an ordinal ranking of treatments, and numerical differences should not be interpreted as reflecting the magnitude of treatment effects.

To assess transitivity, we compared the distribution of clinical and methodological variables that could act as effect modifiers across treatment comparisons (Turner et al., 2012). To assess heterogeneity, subgroup analysis and network meta-regression were conducted for the following variables: (1) whether the participants met diagnostic criteria of major depressive disorder (MDD) or not, (2) publication year, (3) sponsorship from a pharmaceutical company, (4) baseline depression severity, (5) treatment duration, and (6) current comorbidity. The definitions of these variables can be found in Supplementary Appendix 7, p. 89. The sensitivity analysis of the primary outcome was evaluated by (1) conducting separate analyses for nutraceuticals as a monotherapy or an adjunctive therapy; (2) excluding studies with an overall high risk of bias; or (3) excluding studies using <1 g EPA. We also performed CNMA which estimates the effects of complex interventions by assuming that the treatment effects of an intervention consisting of two or more components are the sum of the effects of the individual components. We used CNMA to investigate whether the effects of nutraceuticals are additive when used as an adjunct or in combination with another nutraceutical or ADT. To test the additivity assumption, we first compared the results of standard NMA, which evaluates the relative effects of interventions without explicitly modeling their components, with those of CNMA, which decomposes the treatment effects into the contributions of individual components. This comparison allows us to assess whether component effects align with the overall effects observed in NMA.

We then tested the interaction between some adjunctive nutraceuticals and ADT and the interaction between nutraceuticals in combination therapy, with the interaction term included as an additional component, for primary outcome. A significant positive or negative interaction suggests a synergistic or antagonistic effect between two components. Only one interaction between two components was tested in each analysis.

We used the *netmeta* package for the statistical software R (version 4.1.2, R Foundation for Statistical Computing, Vienna, Austria) (Rücker, König, & Efthimiou, 2021) for most analyses and Stata (version 17.0, College Station, Texas, USA) for meta-regression.

## Results

**Figure 1.**
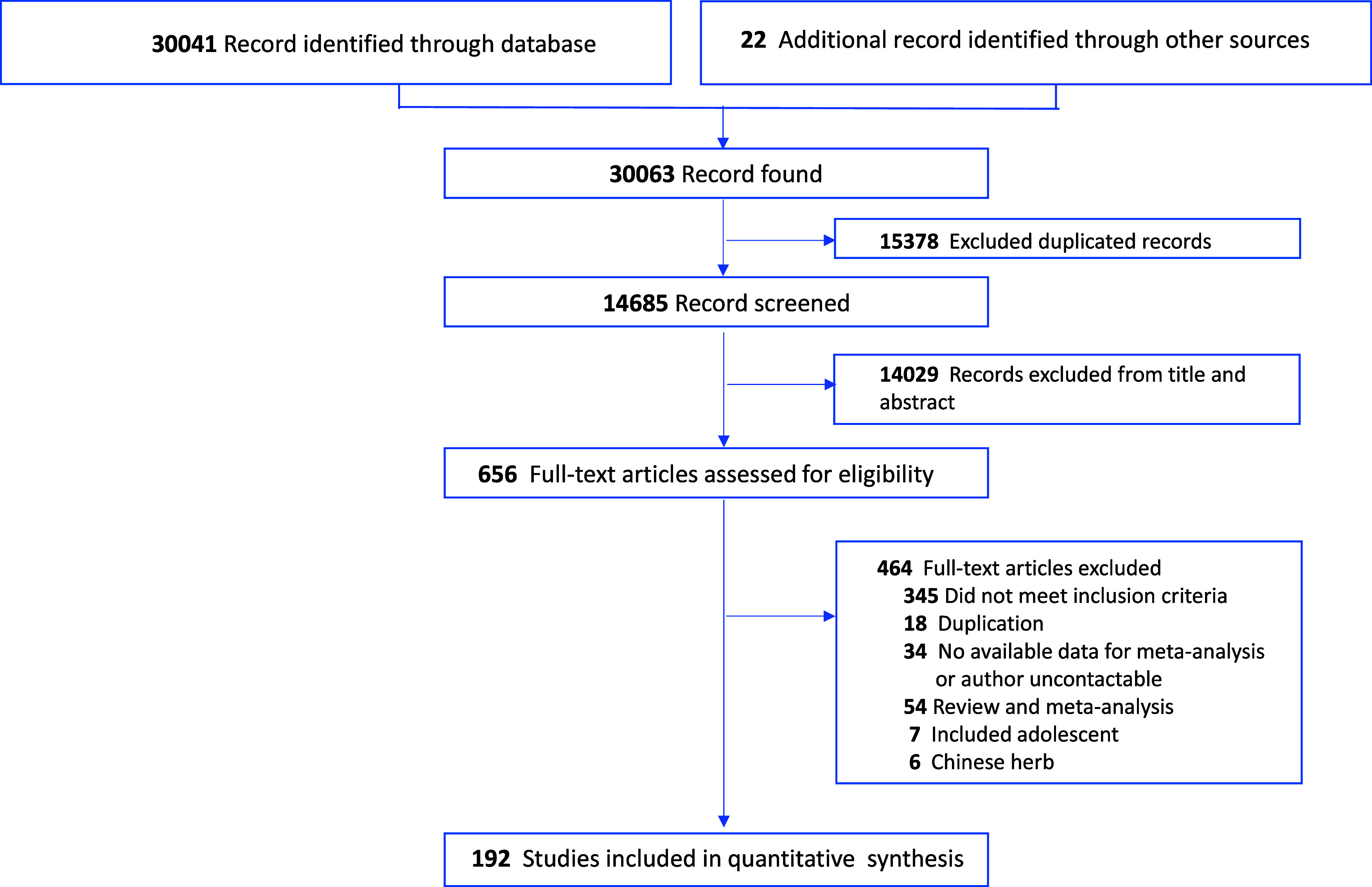
Study selection flowchart. ^a^ Other sources included references mining of the included studies.

The search strategy yielded 30,063 records. Following the screening of titles and abstracts, the full text of 656 articles was retrieved for further assessment. Figure 1 presents the flowchart of the literature search. In total, 192 RCTs, published between 1971 and 2024, were included in our analysis. These RCTs involved 17,437 participants and compared 44 nutraceuticals. Placebo was included as a control in 85 trials, and 22 trials included more than one active intervention. A total of 185 trials (96.4%) were double-blind. The characteristics of the included studies are summarized in the Supplementary Appendix 5 (pp. 42–59). The mean sample size was 42.22 participants (SD 39.42), and the median age was 43.68 years (range: 20–84.9). The median follow-up duration was 8.62 weeks (range: 2–56). Of the 192 trials, 34 (17.7%) recruited patients from North America, 80 (41.7%) from Asia, and 49 (25.5%) from Europe. Approximately two-thirds of patients had moderate or higher depressive symptoms, with a reported baseline mean severity score of 20.07 on HAMD-17 (SD 5.00). Among the 192 included trials, we assessed two with an overall low risk of bias, 154 trials with some concern regarding risk of bias, and 36 trials with a high risk of bias (Supplementary Appendix 6, pp. 70–88). The Cohen’s Kappa for the inter-rater agreement was 0.85 (95% CI: 0.54 to 1.00), which indicates a substantial agreement.

### Primary outcome: changes in scores on the depression rating scale

**Figure 2.**
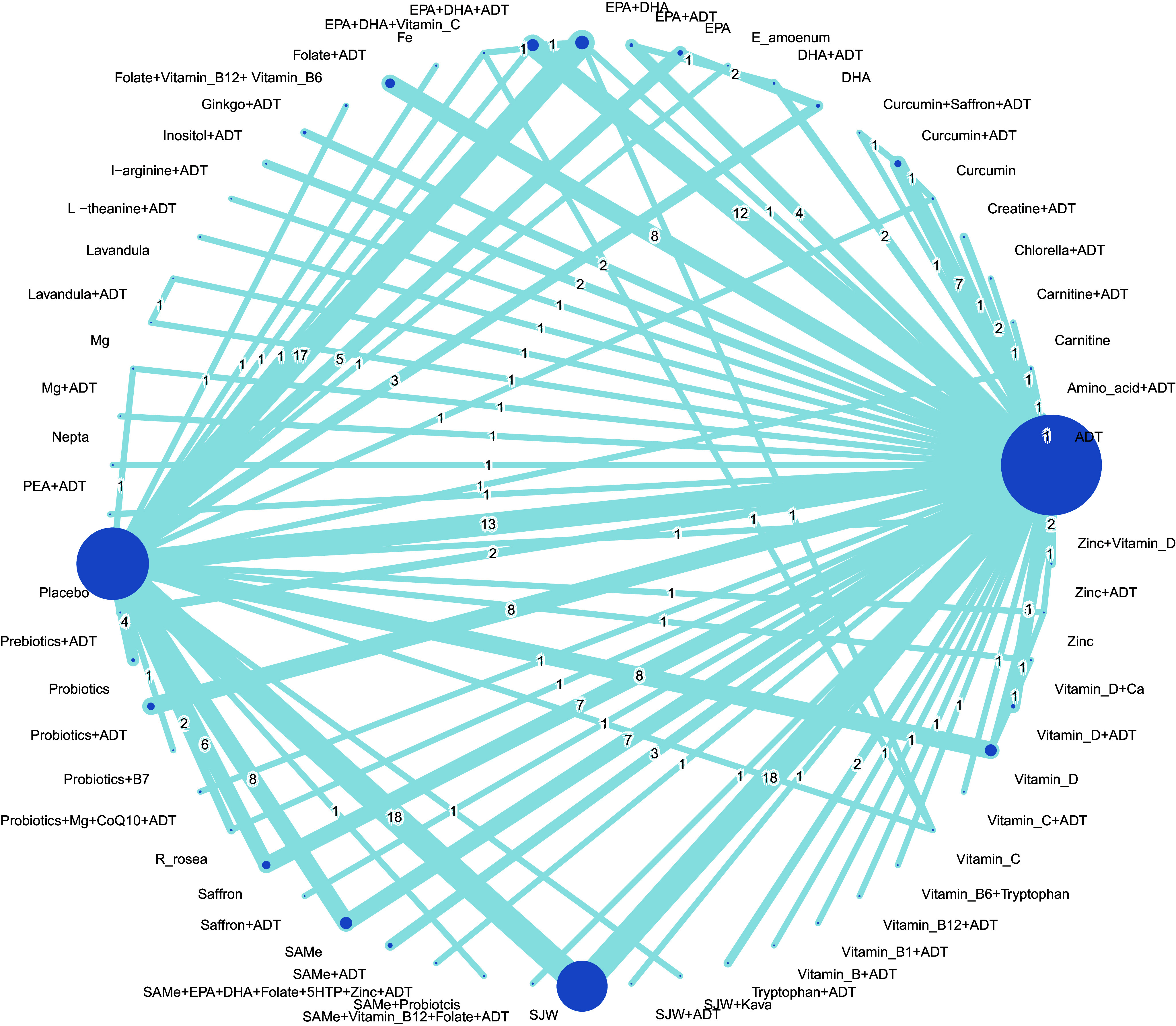
Network diagram for change in depressive symptoms. ^a^ Lines between nodes represent direct comparisons between trials, and the circle size is proportional to the size of the population that received each treatment. Line thickness is proportional to the number of studies providing data for the comparison. ^b^ Abbreviations: ADT: antidepressant; Ca: calcium; CoQ10: co-enzyme Q10; DHA: docosahexaenoic acid; *E amoenum*: *Echium amoenum*; EPA: eicosapentaenoic acid; Fe: ferrum; Mg: magnesium; PEA: palmitoylethanolamide; *R rosea*: *Rhodiola rosea*; SAMe: S-adenosyl methionin; SJW: St. John’s wort; vitamin B1: thiamine; vitamin B6: pyridoxine; vitamin B7: biotin; vitamin B: vitamin B complex; vitamin B12: cobalamin; vitamin C: Ascorbic acid; vitamin D: cholecalciferol; 5HTP: 5-hydroxytryptophan.

**Figure 3.**
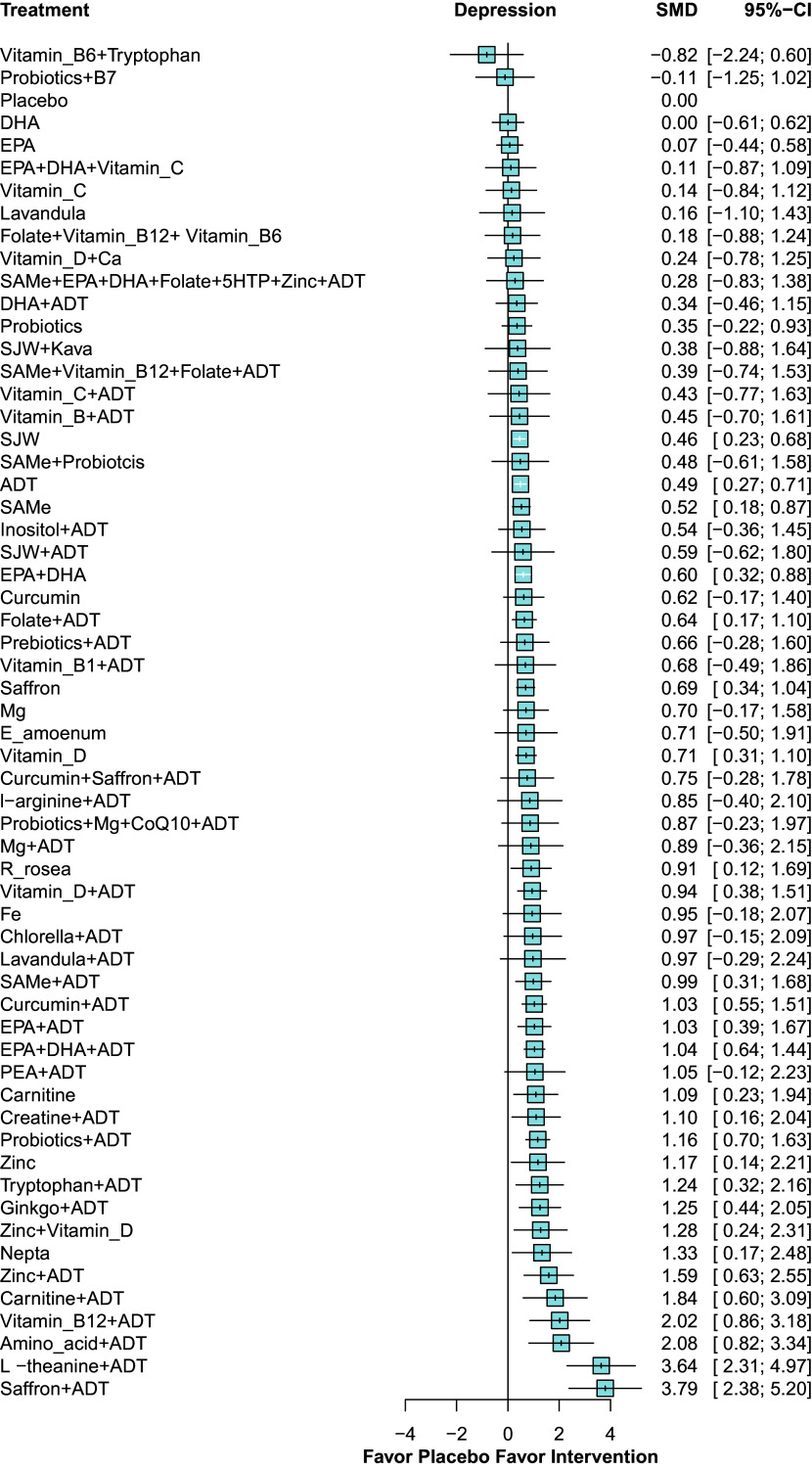
Forest plot for change in depressive symptoms. ^a^ Denotes significance at *p* < 0.05. ^b^ Abbreviations: ADT: antidepressant; Ca: calcium; CI: confidence interval; CoQ10: co-enzyme Q10; DHA: docosahexaenoic acid; *E amoenum*: *Echium amoenum*; EPA: eicosapentaenoic acid; Fe: ferrum; Mg: magnesium; PEA: palmitoylethanolamide; *R rosea*: *Rhodiola rosea*; SAMe: S-adenosyl methionine; SMD: standard mean difference; SJW: St. John’s wort; vitamin B1: thiamine; vitamin B6: pyridoxine; vitamin B7: biotin; vitamin B: vitamin B complex; vitamin B12: cobalamin; vitamin C: ascorbic acid; vitamin D: cholecalciferol; 5HTP: 5-hydroxytryptophan.

To determine the efficacy of nutraceuticals used either as an adjunct or as monotherapy, we analyzed 179 RCTs, comprising 16,362 patients and 232 pairwise direct comparisons between 60 treatment regimens. Network geometry in Figure 2 shows the distribution of evidence. ADT had the largest treatment node, followed by the placebo. All nutraceuticals, used as monotherapy or as an adjunct, were directly connected to ADT or a placebo. Fewer than half of the interventions formed closed loops, where inconsistency between direct and indirect evidence was evaluated. The forest plot in Figure 3 displays the relative effects of nutraceuticals on the SMD of depression rating scales compared with the placebo. Approximately two-thirds of the nutraceuticals demonstrated higher rankings than placebo in terms of efficacy. The SMD for those showing significantly greater efficacy than placebo ranged from 0.46 (95% CI 0.23–0.68) for St. John’s wort (SJW) to 3.79 (95% CI 2.38–5.20) for saffron + ADT. ADT exhibited significant benefits (SMD 0.49), and most nutraceuticals in combination with ADT showed greater efficacy than ADT alone. Moreover, 13 nutraceutical monotherapies were more effective than ADT alone. Among them, eight nutraceuticals used as an adjunct to ADT demonstrated superior efficacy rankings compared to their performance as standalone therapies. These included eicosapentaenoic acid plus docosahexaenoic acid (EPA + DHA), SAMe, curcumin, saffron, carnitine, vitamin D, zinc, and magnesium, indicating that these nutraceuticals were more effective whether employed as monotherapy or as an adjunct to ADT, as opposed to ADT alone (Figure 3).

### Response and remission rate outcome

**Figure 4.**
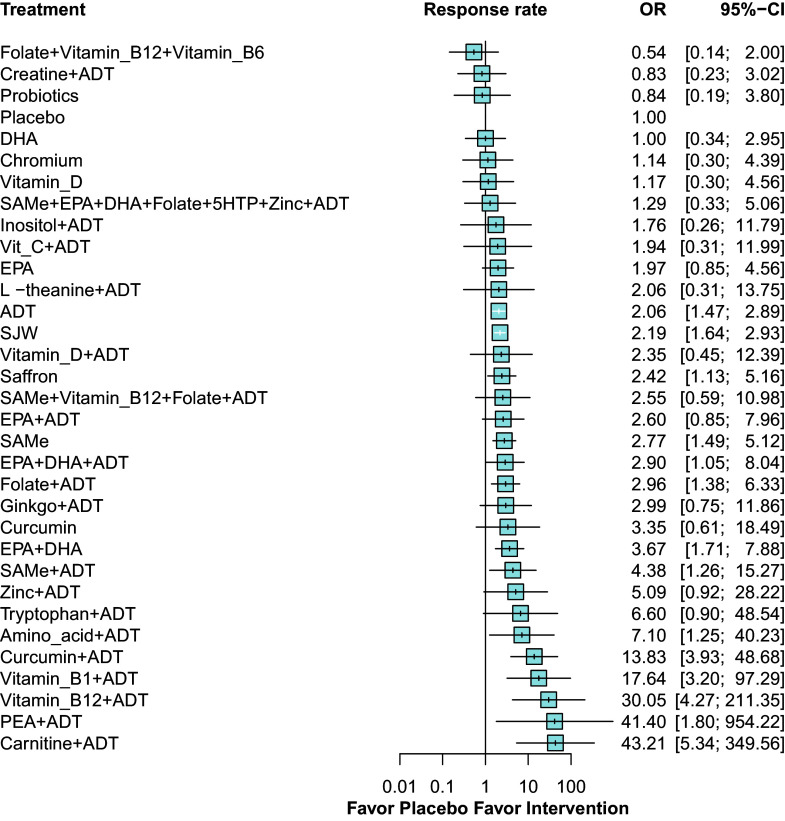
Forest plot for response rate. ^a^ Denotes significance at *p* < 0.05. ^b^ Abbreviations: ADT: antidepressant; CI: confidence interval; DHA: docosahexaenoic acid; EPA: eicosapentaenoic acid; OR: odds ratio; PEA: palmitoylethanolamide; SAMe: S-adenosyl methionine; SJW: St. John’s wort; vitamin B1: thiamine; vitamin B6: pyridoxine; vitamin B12: cobalamin; vitamin C: ascorbic acid; vitamin D: cholecalciferol; 5HTP: 5-hydroxytryptophan.

**Figure 5.**
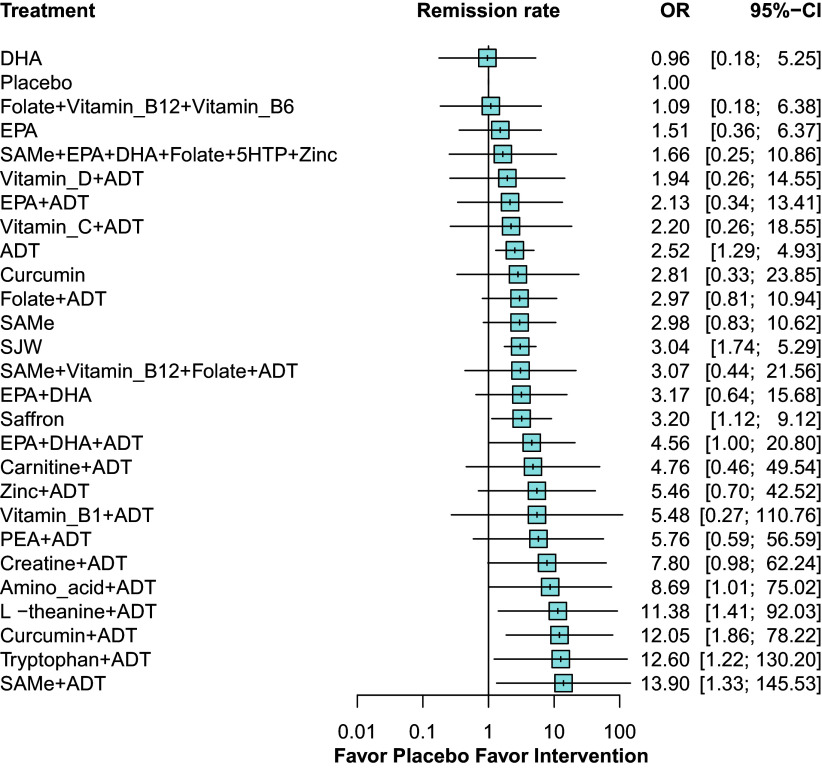
Forest plot for remission rate. ^a^ Denotes significance at *p* < 0.05. ^b^ Abbreviations: ADT: antidepressant; CI: confidence interval; DHA: docosahexaenoic acid; EPA: eicosapentaenoic acid; PEA: palmitoylethanolamide; SAMe: S-adenosyl methionine; SJW: St. John’s wort; vitamin B1: thiamine; vitamin B6: pyridoxine; vitamin B12: cobalamin; vitamin C: ascorbic acid; vitamin D: cholecalciferol; 5HTP: 5-hydroxytryptophan.

Fewer studies reported response rate (86 RCTs, comprising 9,185 patients) and remission rate (45 RCTs, comprising 4,604 patients) (Figures 4 and 5, respectively). As shown in the primary outcome, most adjunctive nutraceuticals showed higher response and remission rates than ADT alone. The complete results and treatment ranking can be found in the Supplementary Material (Supplementary Appendix 10, pp. 94–95, 100–101, 107–108).

In reducing depressive symptoms and response/remission rates, adjunctive nutraceuticals consistently exhibited higher efficacy than ADT alone, including EPA + DHA + ADT, SAMe + ADT, curcumin + ADT, zinc + ADT, amino acid + ADT, Palmitoylethanolamide (PEA) + ADT, tryptophan + ADT, vitamin B1 + ADT, carnitine + ADT and folate + ADT. Four nutraceutical monotherapies, namely EPA + DHA, SAMe, curcumin, and saffron, consistently showed greater efficacy than ADT alone for all treatment outcomes (Supplementary Appendix 10, pp. 110–111).

### Subgroup analysis and sensitivity analysis

Studies were stratified into mild, moderate, and severe groups based on patients’ baseline depression severity (Apaydin et al., 2016), and most adjunctive nutraceuticals consistently demonstrated superior efficacy in terms of depressive scores improvement compared to ADT alone across all subgroups (Supplementary Appendix 11, pp. 112–124). Within the mild depression category, nutraceuticals demonstrated significant improvements in depressive symptoms, ranging from an SMD of 0.54 for EPA + DHA (95% CI 0.11–0.97) to 1.59 for vitamin D + ADT (0.18–3.00) (Supplementary Appendix 11, pp. 113, 116). For moderate depression, the SMD of nutraceuticals, resulting in significant improvements in depressive symptoms, ranged from 0.48 (0.22–0.75) for SJW to 1.97 (0.85–3.09) for *Rhodiola rosea* (Supplementary Appendix 11, pp. 114, 117). For severe depression, the SMD of nutraceuticals, showing significant improvements in depressive symptoms, ranged from 1.64 (0.40–2.89) for EPA + DHA + ADT to 3.98 (2.12–5.85) for saffron+ADT (Supplementary Appendix 11, pp. 115, 118). A few nutraceutical monotherapies, including Nepta, saffron, and vitamin D, were consistently ranked higher than ADT for mild-to-moderate depression. For moderate-to-severe depression, adjunctive nutraceuticals, including EPA + DHA + ADT, SAMe + ADT, tryptophan + ADT, curcumin + ADT, folate + ADT, vitamin D + ADT and inositol + ADT were ranked better than ADT alone. The full results and treatment ranking for different baseline severities can be found in the Supplementary Material (Supplementary Appendix 11, pp. 123–124).

Two sub-analyses were undertaken for studies in which nutraceuticals were used as an adjunctive only or as monotherapy only. For nutraceuticals used as an adjunct only, almost all of them exhibited better efficacy than ADT alone. We noted that most of these results were based on direct evidence in network analysis. Among nutraceuticals used solely as monotherapies, the eight exhibited a higher ranking than ADT in the primary analysis and maintained their superior positions in comparison to ADT (Supplementary Appendix 13.1, 13.2, pp. 203–208). We also investigated the effects of various other factors (e.g. study duration and sponsorship) for subgroup analyses, and the findings were consistent with those of the primary analysis, adjunctive nutraceuticals demonstrate superior efficacy than ADT alone and placebo. Additionally, the ranking of the effective nutraceuticals, such as EPA + DHA, SAMe, curcumin, and saffron, remained higher rank than ADT alone (Supplementary Appendix 13, pp. 203–274).

### CNMA and interactive CNMA

CNMA and interactive CNMA were performed for the primary outcome where the effects of adjunctive nutraceuticals and nutraceuticals combination were available. While there was a significant difference in the model fit between the standard model (NMA) and the additive model (CNMA), the difference between the interactive CNMA and CNMA was not significant. For the primary outcome, the eight nutraceuticals that ranked higher than ADT, either used as an adjunct or monotherapy, mostly showed a non-significant synergistic effect with ADT except for vitamin D and Magnesium, which showed a non-significant antagonistic effect with ADT (Supplementary Appendix 12, pp. 125–202).

### Other secondary outcomes

For anxiety symptoms, 30 studies reported usable results, and most nutraceuticals combined with ADT showed a higher efficacy than ADT alone. However, only vitamin D supplementation demonstrated significant benefit against anxiety symptoms (SMD = 2.79, with a 95% CI of 1.06–4.51) (Supplementary Appendix 10, pp. 96, 102, 109).

ADT (OR 1.25, 95% CI 1–1.56) monotherapy was associated with a significantly higher all-cause discontinuation rate (Supplementary Appendix 10, p. 103) and higher adverse event rate (1.96, 1.37–2.81) than the placebo (Supplementary Appendix 10, p. 104). None of the nutraceuticals used as monotherapy or as an adjunct to ADT were associated with a significantly higher discontinuation rate than the placebo. Adjunctive nutraceuticals were associated with higher but non-significant adverse event rates compared with the placebo, but this may reflect a lack of statistical power due to the small number of studies with limited numbers of patients involved.

Heterogeneity was moderate to high for most outcomes, except for all-cause discontinuation rates (Supplementary Appendix 14, p. 276). None of the prespecified subgroup analyses substantially reduced the level of heterogeneity (Supplementary Appendix 15, pp. 277–278). The design-by-treatment interaction model showed global inconsistency in the NMAs for depressive symptoms and response and remission rates, but not for anxiety, all-cause discontinuation, and adverse events (Supplementary Appendix 16, pp. 279–287). The node-splitting model exhibited significant differences between estimates of direct and indirect evidence for some comparisons in efficacy outcomes, but not for tolerability (Supplementary Appendix 16, pp. 288–307). The comparison-adjusted funnel plots of the NMA for primary outcomes did not indicate publication bias (Supplementary Appendix 17, pp. 308–313). According to GRADE, the quality of evidence for primary outcomes was rated as very low for most comparisons. The quality of evidence was very low for an overall ranking of treatment in terms of efficacy and low for tolerability. Full information on CINeMA is described in Supplementary Appendix 19 (pp. 339–370).

Potential effect modifiers, including publication year and baseline depression severity, evaluated through meta-regression, did not significantly influence the relative treatment effects (Supplementary Appendix 18, pp. 314–335). The results of sensitivity analyses did not differ substantially from those of the main analysis (Supplementary Appendix 13.7–13.20, pp. 222–265).

## Discussion

This is the first NMA to investigate the effects of nutraceuticals on depression. Compared with previous meta-analyses of nutritional supplements for depression treatment, our NMA is a comprehensive assessment of the efficacy/tolerability of nutraceuticals compared with that of placebo or ADT. In general, nutraceutical + ADT was ranked higher than ADT alone or nutraceutical monotherapy. Four nutraceutical monotherapies (i.e. EPA + DHA, SAMe, curcumin, and saffron) consistently exhibited higher efficacy than the placebo and ADT alone for reducing depression symptoms and attaining higher response and remission rates. For mild-to-moderate depression, nutraceutical monotherapy, such as vitamin D and saffron, showed higher efficacy than ADT alone. For moderate-to-severe depression, adjunctive nutraceuticals, including EPA + DHA + ADT, SAMe + ADT, tryptophan+ADT, curcumin+ADT, folate + ADT, vitamin D + ADT, and inositol + ADT exhibited higher efficacy than ADT alone. For tolerability, most nutraceuticals had a similar all-cause discontinuation rate and adverse event rate compared with placebo.

A key mechanism for nutraceuticals’ benefits may involve modulation of gut microbiota. Nutraceuticals such as omega-3, probiotics, and curcumin have been shown to positively influence gut microbiota composition, alleviating dysbiosis and reducing systemic inflammation (Cenit et al., 2017). Gut dysbiosis, often linked to depression, disrupts serotonin signaling in the gastrointestinal tract and increases inflammatory cytokines (Clarke et al., 2019). By restoring microbiota balance, nutraceuticals may enhance serotonergic activity and improve gut-brain axis communication (Cenit et al., 2017). This mechanism complements ADTs, which can also modulate gut microbiota through serotonergic pathways (Yano et al., 2015). For instance, adjunctive therapies like EPA + DHA + ADT and curcumin + ADT likely act synergistically by targeting inflammation, gut microbiota, and neurotransmitter systems (Lopresti and Drummond, 2017, Su et al., 2015).

For all the efficacy outcomes, several nutraceuticals as an adjunctive to ADT were ranked higher than ADT, including EPA + DHA + ADT, SAMe + ADT, curcumin + ADT, zinc + ADT, tryptophan + ADT, carnitine + ADT, and folate + ADT. Among nutraceutical monotherapies, EPA + DHA, SAMe, curcumin, and saffron exhibited higher efficacy than ADT alone. EPA + DHA, SAMe, and curcumin consistently showed benefits in both as adjuncts to ADT and as monotherapy. Some nutraceuticals with superior ranking, such as amino acid + ADT, PEA+ADT, vitamin B1 + ADT, and Nepta, were tested by only one single RCT, and so their results should be cautiously interpreted before they are verified by future studies.

Our results demonstrate that certain nutraceuticals whether used as adjunctive therapy or monotherapy, can help improve depressive symptoms and even aid in response rates and remission of depression. These nutraceuticals work through a series of key pathways related to the pathogenesis of depression, such as inflammation (Suneson et al., 2021), monoamine imbalance (Hamon and Blier, 2013), Hypothalamic–Pituitary–Adrenal axis dysregulation (Hersey, Hashemi, & Reagon, 2022), impaired neurogenesis (Matrisciano et al., 2009), disrupted carbon metabolism (Sarris et al., 2019), and circadian rhythm (Daut and Fonken, 2019).

Our result showed the effect of nutraceuticals varied according to the severity of depression, one of the possible explanations is that the severity of depression may be associated with different underlying biochemical abnormalities, including imbalances in neurotransmitters and increased inflammatory responses. These biochemical abnormalities may be more pronounced in severe depression, which could potentially limit the effectiveness of nutraceutical interventions. Second, the severity of depression may reflect the severity of psychological and behavioral problems. In cases of severe depression, individuals may experience higher levels of psychological and emotional distress, these factors may also influence affecting the effectiveness of nutraceuticals.

In our stratified analyses for mild depression, most nutraceutical monotherapies were less effective than ADT, including the most widely studied nutraceutical such as omega-3. Although EPA + DHA monotherapy was ranked higher than the placebo, it was still lower than the ADT. This echoes that evidence for the use of omega-3 monotherapy in depression treatment is insufficient. Omega-3 is mainly recommended for augmented or accelerated ADT use (Guu et al., 2019). Some nutraceutical monotherapies, such as Nepta, showed better efficacy than ADT in mild-to-moderate depression; these results were based on single trials and should also be interpreted with caution. Two noteworthy nutraceutical monotherapies, vitamin D and saffron, showed significant benefits and better rankings than ADT. Those results were based on both direct and indirect evidence, and further large clinical trials are warranted to verify their effects on mild-to-moderate depression (Guu et al., 2019, Ravindran et al., 2016, Sarris et al., 2016).

Because the types of adverse reactions reported in each trial were somewhat different, we combined all adverse reactions for analysis. Among all nutraceutical monotherapies and adjunctive nutraceuticals, only ADT were associated with more adverse reactions. Most nutraceutical monotherapies exhibit a tolerability level similar to placebo. This is expected because ‘being similar to foods’ and ‘having low side effects’ are general expectations for nutraceuticals and are often the main reasons why patients take them (Fusar-Poli et al., 2019). Nutraceutical adjuncts to ADT might still lead to adverse reactions, but these reactions are likely to be primarily due to ADT itself. All-cause discontinuation, usually considered an index of effectiveness, is related to limited efficacy and intolerable adverse reactions (Huhn et al., 2019). Treatment rankings in the analysis of all-cause discontinuation are similar to those for efficacy, thereby supporting the notion that nutraceuticals elicit less adverse reactions in general.

Based on our findings, several nutraceuticals used as adjuncts to treatment have shown improvements in depression symptoms without significantly higher rates of adverse reactions compared with antidepressant therapy alone. For example, EPA + DHA + ADT and curcumin + ADT, which have more evidence in our study, showed significantly greater efficacy than the placebo on the main outcome. The effect sizes were also higher compared with antidepressant therapy alone, and the rates of adverse reactions for these two adjunctive treatments were similar to those for ADTs alone. These findings suggest that these specific nutraceuticals may be considered as priority options in clinical practice.

For trials reporting anxiety as another outcome, vitamin D was the most effective nutraceutical adjunct. Although omega-3 outperformed ADT and placebos, their differences were not statistically significant. This finding is inconsistent with the results of a previous meta-analysis, which showed that omega-3 significantly reduced anxiety symptoms compared with a placebo (Su et al., 2018). However, that meta-analysis included RCTs on patients exhibiting anxiety symptoms associated with various diagnoses, while our NMA only included patients with depression. Thus, nutraceuticals for anxiety symptoms may vary across patients with different diagnoses. Furthermore, evidence supporting the efficacy of vitamin D in anxiety treatment in our study came from just one study. More studies with greater sample sizes would be helpful for further clarifying the anxiolytic effect of vitamin D.

There is a significant difference in the model fit between the standard NMA model and the additive model (CNMA), indicating that for some component nutraceuticals, their combined effects are not additive. For instance, both vitamin D and DHA showed positive effects when used as a monotherapy, but when used as an adjunctive to ADT, the combined effects decreased. Non-additive effects were also observed when nutraceuticals were used in combination with other nutraceuticals. Because some combinations of nutraceuticals or nutraceuticals with ADT have very few trials, more studies are needed in the future to evaluate the interaction between nutraceuticals and ADT to identify the best combination therapies.

Due to the high heterogeneity in primary outcome, we did several subgroup and sensitivity analyses to explore the potential reasons. The heterogeneity was still moderate to high even if we split the main analysis into adjunctive and monotherapy nutraceuticals, or set many restrictions to the main analysis. Likewise, the high heterogeneity may imply that, unlike trials of psychopharmaceuticals, trials of nutraceuticals do not have a standard process, and there is no consensus or conclusion on the dose range or timing of use, resulting in such high heterogeneity across studies.

Our NMA has several limitations. First, according to CINeMA, we rated many comparisons as low or very low quality, which greatly limits the clinical application of these results. Second, standard classification of nutraceuticals is still lacking (Mishra, 2016). In our analysis, nutraceuticals with similar but not the same chemical compositions (e.g. various types of omega-3 fatty acids and various one-carbon cycle chemicals such as folic acid) were considered different interventions. However, nutraceuticals of similar chemical compositions may work in a shared mechanism (Mishra, 2016); combining them into one group may improve the interpretability of results. However, the heterogeneity remained moderate to high, even when we combined nutraceuticals with similar chemical compositions into the same group. Thirdly, in our study, different dosages of the same nutraceutical were considered as a single group. However, it is important to note that different dosages of certain nutraceuticals, such as omega-3 fatty acids, may affect the treatment response and tolerability. Additionally, the dosage of antidepressants may also influence treatment response and tolerability. Fourth, heterogeneity was observed in the definition of depression, tools for measuring depression, treatment duration, and concomitant medications in our NMA. The dosage and titrating procedure of a nutraceutical can vary across studies. This should be considered when interpreting the results. Fifth, only published data were included in our analysis, although including unpublished data would increase the sample size (Akhondzadeh Basti et al., 2007). However, we found that most unpublished trials lacked sufficient data for analysis, and they compared a wide range of nutrients and nutraceuticals instead of focusing on a specific few. Moreover, no evidence of publication bias was observed in our NMA, indicating that our conclusions are unlikely to be influenced by unpublished studies.

Sixth, the quality of the included studies was not consistently high; sample sizes in some trials were quite small (Levine et al., 1999, Nemets, Mishory, Levine, & Belmaker, 1999, Nowak et al., 2003). Therefore, although some nutraceuticals exhibited better treatment effects and high ranking, the benefits of those with small sample sizes should be interpreted cautiously. Seventh, although pharmacotherapy and nutraceutical interventions have been clearly described in each RCT, whether patients received psychosocial treatments at the same time was not clearly described in many studies. Finally, most studies did not report certain demographic characteristics, such as race and socioeconomic status. This limits the generalizability of our findings, as data about the target populations of the results are only partially available. Notwithstanding these limitations, our network meta-analysis represents the most comprehensive evidence of nutraceuticals for depressive disorder in adults. Our findings indicate that several nutraceuticals, including EPA + DHA, SAMe, and curcumin, whether used as standalone treatment or as adjuncts to ADT, have the potential to alleviate depressive symptoms. Our findings are important to depressive patients who are intolerant to the side effects of ADT or who show poor responses to ADT. These findings are useful for developing treatment guidelines for using nutraceuticals for patients with depression for making a shared decision between patients and their clinicians. Future large-scale RCTs investigating the treatment effect of nutraceuticals are warranted to corroborate the findings of our NMA.

## Conclusions

This comprehensive systematic review and NMA of nutraceuticals for treating depressive disorder has identified several nutraceuticals that are suitable for use as an adjunctive or monotherapy. Our analysis highlights the evidence supporting four nutraceuticals, namely EPA + DHA, SAMe, curcumin, and saffron, as potential add-on treatments or even stand-alone options for adults with depressive disorder. Our study suggests that some adjunctive nutraceuticals may offer superior therapeutic efficacy compared with the use of either antidepressants or nutraceuticals alone. As such, prioritizing adjunctive therapy may be beneficial when considering nutraceuticals for the treatment of depression. Given the relatively small sample sizes in clinical trials of nutraceuticals compared with pharmaceutical trials, further research with larger sample sizes is needed to validate our findings. Additional research is warranted to explore the relationship between therapeutic doses of individual nutraceuticals and their therapeutic efficacy and potential adverse effects.

## Supporting information

Cheng et al. supplementary material Cheng et al. supplementary material

## Data Availability

The data related to the present review are available upon request to the corresponding author.

## Abbreviations

ADT: antidepressant
CI: confidence interval
CINeMA: Confidence In Network Meta-Analysis
CNMA: component network meta-analysis
EPA: eicosapentaenoic acid
DHA: docosahexaenoic acid
DSM: Diagnostic and Statistical Manual
GRADE: the Grading of Recommendations, Assessment, Development and Evaluations
HAMD: Hamilton Rating Scale for Depression
MDD: major depressive disorder
NMA: network meta-analysis
OR: odds ratio
PEA: palmitoylethanolamide
SAMe: S-adenosyl methionine
SJW: Saint John’s wort
SMD: standard mean difference
RCT: randomized controlled trials
rTMS: repetitive transcranial magnetic stimulation
IU: international units

## Supplementary material

The supplementary material for this article can be found at http://doi.org/10.1017/S0033291725000996.
