# Supplementary material for: Comparative efficacy and tolerability of nutraceuticals for depressive disorder: A systematic review and network meta-analysis
Source: Psychol Med. 2025 May 2;55:e134. doi: 10.1017/S0033291725000996 (PMC12094663; doi:10.1017/S0033291725000996)
Supplement: Cheng et al. supplementary material [file S0033291725000996sup001.pdf]

Table of Cotents

Table of Cotents 1

1.PRISMA checklist 11

2.Study Protocol 16

2.1. Aim 16

2.2. PICOS, Inclusion and exclusion criteria 16

2.3. Data extraction 17

2.4. Statistical analysis 17

2.5. Subgroup analysis and network meta-regression 18

2.6. Risk of bias assessment 18

2.7. Changes to Protocol 19

3.Search strategy 20

3.1. PubMed 20

3.2. PsycINFO, PsycARTICLES, CINAHL,MEDLINE 22

3.3. Embase 24

4.References of included studies 25

5. Characteristics of included studies 42

6. Risk of bias 62

6.1.Included studies rated against the Cochrane Risk of Bias tool (ROB 1.0)) 62

6.2.Included studies rated against the revised Cochrane Risk of Bias tool (ROB 2.0) 70

7.Definition of covariates 89

7.1. Sponsorship 89

7.2. Baseline depressive severity 89

7.3. Risk of bias in the included studies 89

7.4. Depressive severity classification 89

7.5. Comorbidity classification 89

8.Hierarchy of depressive scales 90

9.Categorisation of depressive symptoms severity with cut-off scores 91

10.Results from network meta-analyses 92

10.1. Network plot of each outcome 92

10.1.B. Network plot for response rate.. 94

10.1.C. Network plot for remission rate 95

10.1.D. Network plot for change in anxiety symptoms... 96

10.1.E. Network plot for all cause discontinuation rate 97

10.1.F. Network plot for adverse events... 98

10.2. Forest plot of each outcome 99

10.2.A. Forest plot for changes in depressive symptoms 99

10.2.C. Forest plot for remission rate 101

10.2.D. Forest plot for change in anxiety symptoms.. 102

10.2.E. Forest plot for all cause discontinuation 103

10.2.F. Forest plot for adverse event 104

10.3. Treatment ranking and P score for each efficacy outcome...................................... 105

10.3.A. Treatment ranking and P score for changes in depressive symptoms 105

10.3.B. Treatment ranking and P score for response rate... 107

10.3.C. Treatment ranking and P score for remission ate... 108

10.3.D. Treatment ranking and P score for change in anxiety symptoms..................... 109

10.4. Summary table for Treatment ranking of efficacy outcome.................................... 110

11.Network result of subgroups with different baseline depressive severity 112

11.1. Network plot for subgroups with different baseline depressive severity 112

11.1.A. Network plot for mild depression.. 113

11.1.B. Network plot for moderate depression... 114

11.1.C. Network plot for severe depression 115

11.2. Forest plot for subgroups with different baseline depressive severity 116

11.2.A. Forest plot for mild depression.. 116

11.2.B. Forest plot for moderate depression... 117

11.2.C. Forest plot for severe depression 118

11.3. Treatment ranking and P score for subgroups with different baseline depressive severity 119

11.3.A. Treatment ranking and P score for mild depression 119

11.3.B. Treatment ranking and P score for moderate depression 120

11.3.C. Treatment ranking and P score for severe depression 122

11.4. Summary table of Treatment ranking for subgroups with different baseline depressive severity 123

12. Component network meta-analysis (CNMA) and Interaction CNMA 125

12.1. Component network meta-analysis(CNMA) 125

12.1.A. Estimates of the incremental standardize mean differences of each component in CNMA 125

12.1.B. Significant test between NMA and CNMA 127

12.1.C .Forest plot for CNMA of change in depressive symptoms (Additive Model).. 128

12.1.D. Forest plot for each component of change in depressive symptoms 129

12.1.E. Forest plot for combine result from NMA and CNMA 130

12.2 Interaction Component Network Meta analysis 131

12.2.A. Interaction CNMA for EPA+DHA+ADT 131

12.2.B. Interaction CNMA for EPA+DHA 135

12.2.C. Interaction CNMA for SAMe+ADT 139

12.2.D.Interaction CNMA for Curcumin+ADT 143

12.2.E. Interaction CNMA for Probiotics+ADT 147

12.2.F.Interaction CNMA for EPA+ADT 151

12.G.Interaction CNMA for DHA+ADT 155

12.2.H.Interaction CNMA for Vitamin D+ADT 159

12.2.I.Interaction CNMA for Saffron+ADT 163

12.2.J.Interaction CNMA for Zinc+ADT 167

12.2.K.Interaction CNMA for Carnitine+ADT 171

12.2.L.Interaction CNMA for Mg+ADT 175

12.2.M.Interaction CNMA for SJW+ADT 179

12.2.N.Interaction CNMA for Tryptophan+ADT 183

12.2.O.Interaction CNMA for Folate+ADT 187

12.2.P.Interaction CNMA for SAMe+Vitamin_B12+Folate+ADT 191

12.2.Q.Interaction CNMA for EPA+DHA+Vitamin_C 195

12.2.R.Interaction CNMA for Curcumin+Saffron+ADT 199

13.Subgroup analysis and Sensitivity analysis 203

13.1. Subgroup analysis for only adjunctive nutraceuticals 203

13.1-A. Network Plot for only adjunctive nutraceuticals 203

13.1-B. Forest Plot for only adjunctive nutraceuticals 204

13.1-C. Detailed result for only adjunctive nutraceuticals 205

13.2. Subgroup analysis for only nutraceuticals monotherapy 206

13.2-A. Network Plot for only nutraceuticals monotherapy 206

13.2-B. Forest Plot for only nutraceuticals monotherapy 207

13.2-C. Detailed result for only nutraceuticals monotherapy 208

13.3. Subgroup analysis for clinical population 209

13.3-A. Network Plot for clinical population 209

13.3-B. Forest Plot for clinical population 210

13.3-C. Detailed result for clinical population 211

13.4. Subgroup analysis for non-clinical population 213

13.4-A. Network Plot for non-clinical population 213

13.4-B. Forest Plot for non-clinical population 214

13.4-C. Detailed result for non-clinical population 215

13.5. Subgroup analysis of using SSRIs as ADT 216

13.5-A. Network Plot for subgroup analysis of using SSRIs as ADT 216

13.5-B. Forest Plot for subgroup analysis of using SSRIs as ADT 217

13.5-C. Detailed result for subgroup analysis of using SSRIs as ADT 218

13.6. Subgroup analysis of using fluoxetine as ADT 219

13.6-A. Network Plot for subgroup analysis of using fluoxetine as ADT 219

13.6-B. Forest Plot for subgroup analysis of using fluoxetine as ADT......................... 220

13.6-C. Detailed result for subgroup analysis of using fluoxetine as ADT.................. 221

13.7. Sensitivity analysis for excluding only female subjects.. 222

13.7-A. Network Plot for excluding only female subjects 222

13.7-B. Forest Plot for excluding only female subjects... 223

13.7-C. Detailed result for excluding only female subjects.. 224

13.8. Sensitivity analysis for participants aged less than 60 226

13.8-A. Network Plot for participants aged less than 60.. 226

13.8-B. Forest Plot for participants aged less than 60.. 227

13.11-C. Detailed result for participants aged less than 60 228

13.9. Sensitivity analysis for excluding perinatal depression and postpartum depression 230

13.9-A. Network Plot for excluding perinatal depression and postpartum depression . 230

13.9-B. Forest Plot for excluding perinatal depression and postpartum depression...... 231

13.9-C. Detailed result for excluding perinatal depression and postpartum depression 232

13.10. Sensitivity analysis for study duration between 4-12 weeks................................. 234

13.10-A. Network Plot for subgroup for study duration between 4-12weeks............... 234

13.10-B. Forest Plot for subgroup for study duration between 4-12 weeks................. 235

13.10-C. Detailed result for subgroup for study duration between 4-12 weeks............ 236

13.11. Sensitivity analysis for excluding studies using EPA less than 1g......................... 238

13.11-A. Network Plot for excluding studies using EPA less than 1g.......................... 238

13.14-B. Forest Plot for excluding studies using EPA less than 1g 239

13.14-C. Detailed result excluding studies using EPA less than 1g............................. 240

13.12. Sensitivity analysis for combining EPA and DHA to omega-3 polyunsaturated fatty acids group 242

13.12-A. Forest Plot for combining EPA and DHA to omega-3 polyunsaturated fatty acids group 242

13.12-B. Detailed result for combining EPA and DHA to n3 group............................. 243

13.13. Sensitivity analysis for combining SAMe, folate, vitamin B12 and vitamin B6 to one Carbon cycle group 245
13.13-A. Forest Plot for combining SAMe, folate, vitamin B12 and vitamin B6 to one Carbon cycle group.. 245
13.13-B. Detailed result for combining SAMe, folate, vitamin B12 and vitamin B6 to one Carbon cycle group.. 246
13.14. Sensitivity analysis for only omega-3 polyunsaturated fatty acids group 248
13.14-A. Network plot for only omega-3 polyunsaturated fatty acids group................ 248
13.14-B. Forest Plot for only omega-3 polyunsaturated fatty acids group.................... 249
13.14-C. Detailed result for only omega-3 polyunsaturated fatty acids group.............. 250
13.15. Sensitivity analysis for omega 3 group with studies using omega 3 greater than 1g 251
13.15-A. Network plot for omega 3 group with studies using omega 3 greater than 1g251
13.15-B. Forest Plot for omega 3 group with studies using omega 3 greater than 1g... 252
13.15-C. Detailed result for omega 3 group with studies using omega 3 greater than 1g 253
13.16. Sensitivity analysis for only St. John's wort group.. 254
13.16-A. Network plot for only St. John's wort group 254
13.16-B. Forest Plot for only St. John's wort group 255
13.16-C. Detailed result for only St. John's wort group 256
13.17. Sensitivity analysis for only Saffron 257
13.17-A. Network plot for only Saffron 257
13.17-B. Forest Plot for only Saffron 258
13.17-C. Detailed result for only Saffron 259
13.18. Sensitivity analysis for only SAMe 260
13.18-A. Network plot for only SAMe.. 260
13.18-B. Forest Plot for only SAMe 261
13.18-C. Detailed result for only SAMe.. 262
13.19. Sensitivity analysis for only Curcumin 263

13.19-A. Network plot for only Curcumin ... 263

13.19-B. Forest Plot for only Curcumin ... 264

13.19-C. Detailed result for only Curcumin ... 265

13.20..Detailed result of the subgroup analysis (patient without comorbidity versus with or unclear comorbidity) ... 266

13.21.Detailed result of the subgroup analysis (patient without industry sponsorship versus with or unclear industry sponsorship) ... 268

13.22.Detailed result of the subgroup analysis (study duration less than 8 week versus greater than 8 week) ... 270

13.23.Detailed result of the subgroup analysis (study publication before 2013 versus after 2013 ... 272

13.24.Detailed result of the sensitivity analysis excluding high risk of bias ... 274

14.Heterogeneity /Inconsistency test result, I^2 and heterogeneity estimate τ 2 ... 276

15.Changes in heterogeneity for sensitivity analyses & Subgroup analysis ... 277

15.1.Heterogeneity for sensitivity analysis and subgroup analysis ... 277

15.2 Heterogeneity of Network meta-analysis for specific nutraceuticals ... 278

16.Assessment of inconsistency results for each outcome ... 279

16.1. Summary result of Design by treatment model for each outcome ... 279

16.2 Evaluation of the inconsistency by Design by treatment model ... 280

16.2.A. Evaluation of the inconsistency by Design by treatment model for outcome as change in depressive symptoms ... 280

16.2.B. Evaluation of the inconsistency by Design by treatment model for outcome as response rate ... 282

16.2.C. Evaluation of the inconsistency by Design by treatment model as remission rate ... 283

16.2.D. Evaluation of the inconsistency by Design by treatment model for outcome as change in anxiety sympomts ... 284

16.2.E. Evaluation of the inconsistency by Design by treatment model for outcome as all cause discontinuation ... 285

16.2.F. Evaluation of the inconsistency by Design by treatment model for outcome as adverse event 287

16.3. Evaluation of the inconsistency by node split model 288

16.3.A. Evaluation of the inconsistency by node split model for outcome as change in depressive symptom 288

16.3.B. Evaluation of the inconsistency by node split model for outcome as response rate 292

16.3.C. Evaluation of the inconsistency by node split model for outcome as remission rate 295

16.3.D. Evaluation of the inconsistency by node split model for outcome as change in anxiety symptoms 299

16.3.E. Evaluation of the inconsistency by node split model for outcome as all cause discontinuation 300

16.3.F. Evaluation of the inconsistency by node split model for outcome as adverse event 304

17.Comparison-adjusted funnel plot for each outcome from the network meta-analysis . 308

17.1. Funnel plot of change in depressive sympotms 308

17.2. Funnel plot of reponse rate 309

17.3. Funnel plot of remission rate 310

17.4. Funnel plot of change in anxiety sympotms 311

17.5. Funnel plot of all cause discontinuation 312

17.6. Funnel plot of adverse event 313

18.Meta-regression 314

18.1. Meta-regression : Covariate as publication year 320

18.2. Meta-regression : Covariate as industry sponsorship 323

18.3 Meta-regression : Covariate as risk of bias 329

18.4 Meta-regression : Covariate as study length 332

18.5 Meta-regression : Covariate as baseline depressive severity 335

19.Grading the confidence in evidence with CINeMA 339

19.1.General 339

19.2.A. Change in depressive symptoms.. 錯誤! 尚未定義書籤。

19.2.A-1.Network plot for the network meta-analysis of change in depressive symptom using four different sizing and coloring combinations. .... 錯誤! 尚未定義書籤。

19.2.A-2.Indirectness chart for change in depressive symptom... 錯誤! 尚未定義書籤。

19.2.A-3.Confidence rating in trails for change in depressive symptom . 錯誤! 尚未定義書籤。

19.2.B. Response rate .... 錯誤! 尚未定義書籤。

19.2.B-1.Network plot for the network meta-analysis of response using four different sizing and coloring combinations. .... 錯誤! 尚未定義書籤。

19.2.B-2.Indirectness chart for response rate .... 錯誤! 尚未定義書籤。

19.2.B.3.Confidence rating in trails for response rate .... 錯誤! 尚未定義書籤。

19.2.C. Remission rate .... 錯誤! 尚未定義書籤。

19.2.C-1.Network plot for the network meta-analysis of remission using four different sizing and coloring combinations. .... 錯誤! 尚未定義書籤。

19.2.C-2. Indirectness chart for remission rate............................ 錯誤! 尚未定義書籤。

19.2.C-3. Confidence rating in trails for remission rate .... 錯誤! 尚未定義書籤。

19.2.D. Change in anxiety symptom .... 錯誤! 尚未定義書籤。

19.2.D-1.Network plot for the network meta-analysis of change in anxiety symptom using four different sizing and coloring combinations. .... 錯誤! 尚未定義書籤。

19.2.D-2.Indirectness chart for change in anxiety symptom .... 錯誤! 尚未定義書籤。

19.2.D-3.Confidence rating in trails for change in anxiety symptom .. 錯誤! 尚未定義書籤。

19.2.E. All cause discontinuation .... 錯誤! 尚未定義書籤。

19.2.E-1.Network plot for the network meta-analysis of all cause discontinuation using four different sizing and coloring combinations.......................... 錯誤! 尚未定義書籤。

19.2.E-2.Indirectness chart for all cause discontinuation .... 錯誤! 尚未定義書籤。

19.2.E-3.Confidence rating in trails for all cause discontinuation 錯誤! 尚未定義書籤。

19.2.F Adverse event.. 錯誤! 尚未定義書籤。

19.2.F-1.Network plot for the network meta-analysis of adverse event using four different sizing and coloring combinations. .... 錯誤! 尚未定義書籤。

19.2.F-2.Indirectness chart for adverse event............................... 錯誤! 尚未定義書籤。

19.2.F-3.Confidence rating in trails for adverse event .... 錯誤! 尚未定義書籤。

20. Assessment of transitivity 371

20.1. Assessment of transitivity-Study length.. 373

20.2. Assessment of transitivity-Baseline Severity (HAMD-17)...................................... 374

20.3. Assessment of transitivity-Mean Age 375

20.4. Assessment of transitivity-Publication year 376

20.5. Assessment of transitivity-Sample size.. 377

21 Unpublished clinical trials that meet our inclusion criteria. 378

1.PRISMA checklist

Section/Topic	Item #	Checklist Item [16]	Reported on Page #
**TITLE**			
Title	1	Identify the report as a systematic review *incorporating a network meta-analysis (or related form of meta-analysis).*	**1, Title section**
**ABSTRACT**			
Structured summary	2	Provide a structured summary including, as applicable: **Background:** main objectives; **Methods:** data sources; study eligibility criteria, participants, and interventions; study appraisal; and *synthesis methods, such as network meta-analysis.* **Results:** number of studies and participants identified; summary estimates with corresponding confidence/credible intervals; *treatment rankings may also be discussed. Authors may choose to summarize pairwise comparisons against a chosen treatment included in their analyses for brevity.* **Discussion/Conclusions:** limitations; conclusions and implications of findings. **Other:** primary source of funding; systematic review registration number with registry name.	**3, Abstract section**
**INTRODUCTION**			
Rationale	3	Describe the rationale for the review in the context of what is already known, *including mention of why a network meta-analysis has been conducted.*_	**6, Introduction (3rd paragraph)**
Objectives	4	Provide an explicit statement of questions being addressed, with reference to participants, interventions, comparisons, outcomes, and study design (PICOS).	**6, Introduction (3rd paragraph); Appendix 2**
**METHODS**			
Protocol and registration	5	Indicate whether a review protocol exists: **PROSPERO register：CRD42020151158**	**6-7, Method (1st paragraph)**
Eligibility criteria	6	Specify study characteristics (e.g., PICOS, length of follow-up) and report characteristics (e.g., years	**7, Method (2 nd &3nd paragraph);**

		considered, language, publication status) used as criteria for eligibility, giving rationale. *Clearly describe eligible treatments included in the treatment network, and note whether any have been clustered or merged into the same node (with justification).*_	**Appendix 2**

Information sources	7	Describe all information sources (e.g., databases with dates of coverage, contact with study authors to identify additional studies) in the search and date last searched.	**7, Method (2nd paragraph)**
Search	8	Present full electronic search strategy for at least one database, including any limits used, such that it could be repeated.	**7, Method (2nd paragraph); Appendix 3**
Study selection	9	State the process for selecting studies (i.e., screening, eligibility, included in systematic review, and, if applicable, included in the meta-analysis).	**7, Method (2nd paragraph); Appendix 1**
Data collection process	10	Describe method of data extraction from reports (e.g., piloted forms, independently, in duplicate) and any processes for obtaining and confirming data from investigators.	**8, Method (4th paragraph)**
Data items	11	List and define all variables for which data were sought (e.g., PICOS, funding sources) and any assumptions and simplifications made.	**8-9, Method (5th paragraph); Appendix 2**
**Geometry of the network**	**S1**	Describe methods used to explore the geometry of the treatment network under study and potential biases related to it. This should include how the evidence base has been graphically summarized for presentation, and what characteristics were compiled and used to describe the evidence base to readers.	**9-10, Method (6st & 7st paragraph)**
Risk of bias within individual studies	12	Describe methods used for assessing risk of bias of individual studies (including specification of whether this was done at the study or outcome level), and how this information is to be used in any data synthesis.	**8, Method (4st paragraph**
Summary measures	13	State the principal summary measures (e.g., risk ratio, difference in means). *Also describe the use of additional summary measures assessed, such as treatment rankings and surface under the cumulative*	**9-10, Method (6st *10st paragraph**

		ranking curve (SUCRA) values, as well as modified approaches used to present summary findings from meta-analyses.	
Planned methods of analysis	14	Describe the methods of handling data and combining results of studies for each network meta-analysis.	**9, Method (6st paragraph**
**Assessment of Inconsistency**	**S2**	Describe the statistical methods used to evaluate the agreement of direct and indirect evidence in the treatment network(s) studied. Describe efforts taken to address its presence when found.	**9-10, Method (7st & 8st paragraph**
Risk of bias across studies	15	Specify any assessment of risk of bias that may affect the cumulative evidence (e.g., publication bias, selective reporting within studies).	**10, Method (10st paragraph)**
Additional analyses	16	Describe methods of additional analyses if done, indicating which were pre-specified.	**10-11, Method (10st paragraph)**
**RESULTS†**			
Study selection	17	Give numbers of studies screened, assessed for eligibility, and included in the review, with reasons for exclusions at each stage, ideally with a flow diagram.	**11 Results (1st paragraph); Figure1**
**Presentation of network structure**	**S3**	Provide a network graph of the included studies to enable visualization of the geometry of the treatment network.	**Figure 2**
**Summary of network geometry**	**S4**	Provide a brief overview of characteristics of the treatment network. This may include commentary on the abundance of trials and randomized patients for the different interventions and pairwise comparisons in the network, gaps of evidence in the treatment network, and potential biases reflected by the network structure.	**11-12, Results (1st paragraph).**
Study characteristics	18	For each study, present characteristics for which data were extracted (e.g., study size, PICOS, follow-up period) and provide the citations.	**11-12, Results (1nd paragraph); Appendix 5**
Risk of bias within studies	19	Present data on risk of bias of each study and, if available, any outcome level assessment.	**11-12, Results (1nd paragraph); Appendix 6**
Results of individual	20	For all outcomes considered (benefits or harms), present, for each study: 1) simple summary data for	**12, Results (2nd &3rd paragraph);**

studies		each intervention group, and 2) effect estimates and confidence intervals.	**Appendix 10.1, 10.2**
Synthesis of results	21	Present results of each meta-analysis done, including confidence/credible intervals. If additional summary measures were explored (such as treatment rankings), these should also be presented.	**12-13, Results (2nd &3rd paragraph); Appendix 10.2, 10.3, 10.4**
**Exploration for inconsistency**	**S5**	Describe results from investigations of inconsistency. This may include such information as measures of model fit to compare consistency and inconsistency models, *P* values from statistical tests, or summary of inconsistency estimates from different parts of the treatment network.	**15-16, Results (11th paragraph); Appendix 14**
Risk of bias across studies	22	Present results of any assessment of risk of bias across studies for the evidence base being studied.	**12, Results (1st paragraph)**
Results of additional analyses	23	Give results of additional analyses, if done (e.g., sensitivity or subgroup analyses, meta-regression analyses, *alternative network geometries studied, alternative choice of prior distributions for Bayesian analyses,* and so forth).	**13-15, Results (4th -7th paragraph); Appendix 12,13**
**DISCUSSION**			
Summary of evidence	24	Summarize the main findings, including the strength of evidence for each main outcome; consider their relevance to key groups (e.g., healthcare providers, users, and policy-makers).	**16-17, Discussion (1st paragraph);**
Limitations	25	Discuss limitations at study and outcome level (e.g., risk of bias), and at review level (e.g., incomplete retrieval of identified research, reporting bias). *Comment on the validity of the assumptions, such as transitivity and consistency. Comment on any concerns regarding network geometry (e.g., avoidance of certain comparisons).*	**20-21, Discussion (10th paragraph)**
Conclusions	26	Provide a general interpretation of the results in the context of other evidence, and implications for future research.	**22, Discussion (11th paragraph) &Conclusions (1st paragraph)**
**FUNDING**			

Funding	27	Describe sources of funding for the systematic review and other support (e.g., supply of data); role of funders for the systematic review. This should also include information regarding whether funding has been received from manufacturers of treatments in the network and/or whether some of the authors are content experts with professional conflicts of interest that could affect use of treatments in the network.	**24**

PICOS = population, intervention, comparators, outcomes, study design.

† Authors may wish to plan for use of appendices to present all relevant information in full detail for items in this section.

2.Study Protocol

The original study protocol was registered with Propspero (No. CRD42020151158) https://www.crd.york.ac.uk/prospero/display_record.php?RecordID=151158

2.1. Aim

To do a systematic review and network meta-analysis to compare the efficacy and tolerability of different nutraceuticals on patients with depressive disorder.

2.2. PICOS, Inclusion and exclusion criteria

Patient	Patients with diagnosed with major depressive disorder or depressive disorder, or ongoing depression (defined as current use of antidepressant medication and moderate or above thereshold level of depressive symptom according to validated scales).
Intervention	Any nutritional intervention (nutrition monotherpay or adjunctive nutritional supplementation to antidepressants) would be considered, such as (fish oil, St John Wort, SAMe, tryptophan, folic acid, vitamin B, vitamin C, vitamin D, minerals, probiotics, saffron, curcumin.etc)
Comparator	The comparators will be any nutritional intervention (nutrition monotherpay or adjunctive nutrition plus to antidepressants) versus antidepressant versus placebo.
Outcomes	1. Change in scores on the standard observer rating scale for depression<br>2. Response rate<br>3. Remission rate<br>4. Change of Anxiety symptoms<br>5. All cause discontinuation<br>6. Adverse event
Study design	Prospective randomized controlled trials
Inclusion criteria	1. Randomized controlled trial.<br>2. Participant's age≧18 years.<br>3. Patients diagnosed with major depressive disorder or depressive disorder, or ongoing depression (defined as current use of antidepressant medication and moderate or above thereshold level of depressive symptom according to validated scales).

Exclusion criteria	1. Observational study designs, cross-over trial, or single arm study (But if crossover studies provided efficacy data before the crossover. The first period of crossover randomized trials was eligible for inclusion)<br>2. Randomized controlled trials with participants diagnosed as bipolar depression<br>3. Studies with relapse prevention or discontinuation designs<br>4. Not reporting the target outcome<br>5. With nonrandom allocation of treatments<br>6. No treatment group receiving nutraceuticals were excluded<br>7. Studies involving the use of traditional Chinese medicine

2.3. Data extraction

Two review authors (YCC and WLH) will independently screen titles and abstracts of all potential studies identified through the search. For studies identified as potentially eligible, we will retrieve the full-text report. Two review authors (YCC and WEH) will independently screen the full-text and identify studies for inclusion, and identify and record reasons for exclusion of the ineligible studies. We will resolve any disagreement through discussion or, if required, we will consult a third review author (PHK). We will identify and exclude duplicate records and we will collate multiple reports that relate to the same study so that each study rather than each report is the unit of interest in the review. We will record the selection process in sufficient detail to complete a PRISMA flow diagram and 'Characteristics of excluded studies' table.

2.4. Statistical analysis

A network meta-analysis will draw on both direct evidence and indirect evidence, with the benefit of randomization in each study retained. For indirect and direct evidence to be consistent, population and intervention characteristics must be similar across comparisons. The relative treatment effects between two interventions are expressed as the SMD for continuous outcomes and odds ratio (OR) for dichotomous outcomes with the 95% confidence interval (CI). The distribution and geometry of the evidence were examined by producing a network plot with node sizes

proportional to the number of study participants and the width of edges proportional to the number of studies comparing the two treatments. The assumption of transitivity in the network was assessed by considering the distributions of major effect modifiers for all comparisons within the networks. Consistency between direct and indirect evidence for each pair of treatments in the network was assessed globally by using the design-by-treatment interaction model and locally by using the node-splitting model. We ranked treatments according to their P-scores, ranging between 0 and 1. P-scores, derived from the point estimates and standard errors of treatment effects, are indices for measuring the extent of certainty to which a treatment is better than another treatment, averaged over all competing treatments. We used the *netmeta* package for the statistical software R (version 4.1.2, R Foundation for Statistical Computing, Vienna, Austria) for most analyses and Stata (version 17.0, College Station, Texas, USA) for meta-regression.

2.5. Subgroup analysis and network meta-regression

We conducted for the following variables: (1) publication year, (2) sponsorship from a pharmaceutical company, (3) baseline depression severity, (4) treatment duration, (5) and current comorbidity

2.6. Risk of bias assessment

Two review authors (YCC and WLH) independently assessed risk of bias for each included trial using the criteria outlined in the Cochrane Handbook for Systematic Reviews of Interventions. We will resolve any disagreements by discussion or by involving a third review author（WYC and YCH）. We assessed the risk of bias according to the following domains:

random sequence generation;
allocation concealment;
blinding of participants and personnel;
blinding of outcome assessment;
incomplete outcome data;

selective outcome reporting;
other bias.

We judged each potential source of bias as either high, low or unclear and provided a supporting quotation from the study report together with a justification for our judgment in the ‘Risk of bias' table.

2.7. Changes to Protocol

The following changes have been made to the original protocol and included in the final publication

- ✓ An additional sensitivity analysis was performed by excluding trials with high risk of bias
- ✓ An additional outcome as anxiety symptoms was performed

3.Search strategy

3.1. PubMed

Recent queries in pubmed		
Search	Query	Items found
#4	(((((Major depressive disorder) OR Depression) OR Depressive disorder) OR Major depression) AND ((Fish oils) OR Omega-3) OR Fatty acid) OR Fatty acids) OR Eicosapentaenoic acid) OR Docosahexaenoic Acid) OR Fatty acid, omega-3) OR Fatty acid, omega-6) OR Fatty acid, essential) OR EPA) OR DHA) OR Probiotics) OR Prebiotics) OR Lactobacillus) OR Bifidobacterium) OR bifidobacter) OR Tryptophane) OR Inositol) OR Amino acid) OR minerals) OR L-5-HTP) OR Vitamin C) OR Ascorbic acid) OR vitamins B) OR pyridoxine) OR cobalamin) OR Vitamin D) OR 25-hydroxyvitaminD) OR 25-hydroxyvitamin D3) OR 1,25 dihydroxyvitamin D3) OR 25(OH)D) OR cholecalciferol) OR calciferol) OR Selenium) OR s-adenocyl-methionine) OR SAMe) OR thiamine) OR Nutrition supplement) OR Complementary therapies) OR Alternative medicine) OR nutraceutical) OR Nutritional supplement) OR Folic acid) OR Folinic acid) OR Magnesium) OR St. John? wort) OR Creatine) OR Ginkgo biloba) OR Curcumin) OR Zinc)) AND (((((randomized controlled trial) OR controlled clinical trial) OR randomized) OR randomly) OR clinical study)	12,476
#3	((((randomized controlled trial) OR controlled clinical trial) OR randomized) OR randomly) OR clinical study	2,567,242
#2	(((Fish oils) OR Omega-3) OR Fatty acid) OR Fatty acids) OR Eicosapentaenoic acid) OR Docosahexaenoic Acid) OR Fatty acid, omega-3) OR Fatty acid, omega-6) OR Fatty acid, essential) OR EPA) OR DHA) OR Probiotics) OR Prebiotics) OR Lactobacillus) OR Bifidobacterium) OR bifidobacter) OR Tryptophane) OR Inositol) OR Amino acid) OR minerals) OR L-5-HTP) OR Vitamin C) OR Ascorbic acid) OR vitamins B) OR pyridoxine) OR cobalamin) OR Vitamin D) OR 25-hydroxyvitaminD) OR 25-hydroxyvitamin D3) OR 1,25 dihydroxyvitamin D3) OR 25(OH)D) OR cholecalciferol) OR calciferol) OR Selenium) OR s-adenocyl-methionine) OR SAMe) OR thiamine) OR Nutrition supplement) OR Complementary therapies) OR Alternative medicine) OR nutraceutical) OR Nutritional supplement) OR Folic acid) OR Folinic acid) OR Magnesium) OR St. John wort) OR Creatine) OR Ginkgo biloba) OR Curcumin) OR Zinc	2,703,618

#1	(((Major depressive disorder) OR Depression) OR Depressive disorder) OR Major depression	674,047

3.2. PsycINFO, PsycARTICLES, CINAHL,MEDLINE

#	Query	Limiters/Expanders	Last Run Via	Results
S5	(S1 OR S2) AND (depression or depressive disorder or depressive symptoms or major depressive disorder) AND randomized controlled trials	Expanders - Apply equivalent subjects Search modes - Find all my search terms	Interface - EBSCOhost Research Databases Search Screen - Advanced Search Database - CINAHL;APA PsycInfo;MEDLINE	9,,673
S5	(S1 OR S2) AND (depression or depressive disorder or depressive symptoms or major depressive disorder)	Expanders - Apply equivalent subjects Search modes - Find all my search terms	Interface - EBSCOhost Research Databases Search Screen - Advanced Search Database - CINAHL;APA PsycInfo;MEDLINE	30,707
S4	depressive disorder or depressive symptoms or major depressive disorder	Expanders - Apply equivalent subjects Search modes - Find all my search terms	Interface - EBSCOhost Research Databases Search Screen - Advanced Search Database - MEDLINE;APA PsycInfo;APA PsycArticles;CINAHL	500,478
S3	S1 OR S2	Expanders - Apply equivalent subjects Search modes - Find all my search terms	Interface - EBSCOhost Research Databases Search Screen - Advanced Search Database - CINAHL;APA PsycInfo;MEDLINE	2,098,355

S2	(folic acid OR magnesium OR (hypericum perforatum or st johns wort) OR creatinine OR carnitine OR crocus sativus OR thiamine) OR (saffron or crocus sativus) AND curcumin	Expanders - Apply equivalent subjects<br>Search modes - Find all my search terms	Interface - EBSCOhost Research Databases<br>Search Screen - Advanced Search<br>Database - MEDLINE;APA PsycInfo;APA PsycArticles;CINAHL	381,248
S1	(fish oil or omega-3 or n-3 fatty acids or epa or dha or fatty acid) OR probiotics OR tryptophan OR inositol OR amino acids OR (ascorbic acid or vitamin c) OR (pyridoxine or b6) OR cobalamin OR vitamin d OR selenium OR s-adenosylmethionine OR nutraceuticals	Expanders - Apply equivalent subjects<br>Search modes - Find all my search terms	Interface - EBSCOhost Research Databases<br>Search Screen - Advanced Search<br>Database - MEDLINE;APA PsycInfo;APA PsycArticles;CINAHL	1,776,041

3.3. Embase

No.	Query	Results
#4	#1AND #2 AND 'controlled study'/de	7892
#3	#1 AND #2	184,827
#2	#'major depression'/exp OR 'depression'/exp OR depression OR 'depressive disorder'/exp OR 'depressive disorder' OR (depressive AND ('disorder'/exp OR disorder)) OR 'major depression' OR (major AND depression) OR 'dysthymia'/exp OR dysthymia'	1,050,110
#1	'fish oil'/exp OR 'fish oil' OR 'omega amino acid'/exp OR 'omega amino acid' OR 'fatty acid'/exp OR 'fatty acid' OR 'docosahexaenoic acid'/exp OR 'docosahexaenoic acid' OR 'omega 3 fatty acid'/exp OR 'omega 3 fatty acid' OR 'probiotic agent'/exp OR 'probiotic agent' OR 'prebiotic agent'/exp OR 'prebiotic agent' OR 'lactobacillus'/exp OR lactobacillus OR 'bifidobacterium'/exp OR bifidobacterium OR 'bifidobacteriales'/exp OR bifidobacteriales OR 'tryptophan'/exp OR tryptophan OR 'inositol'/exp OR inositol OR 'amino acid'/exp OR 'amino acid' OR 'ascorbic acid'/exp OR 'ascorbic acid' OR 'vitamin b group'/exp OR 'vitamin b group' OR 'pyridoxine'/exp OR pyridoxine OR 'cobalamin derivative'/exp OR 'cobalamin derivative' OR 'vitamin d'/exp OR 'vitamin d' OR '25 hydroxyvitamin d'/exp OR '25 hydroxyvitamin d' OR 'colecalciferol derivative'/exp OR 'colecalciferol derivative' OR 'selenium'/exp OR selenium OR 's adenosylmethionine'/exp OR 's adenosylmethionine' OR 'thiamine'/exp OR thiamine OR 'nutrition'/exp OR nutrition OR 'nutraceutical'/exp OR nutraceutical OR 'folic acid derivative'/exp OR 'folic acid derivative' OR 'magnesium'/exp OR magnesium OR 'hypericum perforatum extract'/exp OR 'hypericum perforatum extract' OR 'creatinine'/exp OR creatinine OR 'curcumin'/exp OR curcumin OR 'zinc'/exp OR zinc OR 'carnitine'/exp OR carnitine OR 'crocus sativus'/exp OR 'crocus sativus'	7592,523

4. *References of included studies*

1. Ayuso Gutierrez JL, López-Ibor Alino JJ. Tryptophan and an MAOI (Nialamide) in the Treatment of Depression. *International Pharmacopsychiatry* 1971; **6**: 92-7.
2. Hoes MJ, Sijben N. The clinical significance of disordered renal excretion of xanthurenic acid in depressive patients. *Psychopharmacology* 1981; **75**(4): 346-9.
3. Coppen A, Chaudhry S, Swade C. Folic acid enhances lithium prophylaxis. *Journal of affective disorders* 1986; **10**(1): 9-13.
4. Thomas CS, Bottiglieri T, Edeh J, Carney MW, Reynolds EH, Toone BK. The influence of S-adenosylmethionine (SAM) on prolactin in depressed patients. *International clinical psychopharmacology* 1987; **2**(2): 97-102.
5. Bell KM, Plon L, Bunney WE, Jr., Potkin SG. S-adenosylmethionine treatment of depression: a controlled clinical trial. *The American journal of psychiatry* 1988; **145**(9): 1110-4.
6. Potkin SG, Bell K, Plon L, Bunney WE, Jr. Rapid antidepressant response with SAMe. A double-blind study. *The Alabama journal of medical sciences* 1988; **25**(3): 313-6.
7. Godfrey PS, Toone BK, Carney MW, et al. Enhancement of recovery from psychiatric illness by methylfolate. *Lancet (London, England)* 1990; **336**(8712): 392-5.
8. Kagan BL, Sultzer DL, Rosenlicht N, Gerner RH. Oral S-adenosylmethionine in depression: a randomized, double-blind, placebo-controlled trial. *The American journal of psychiatry* 1990; **147**(5): 591-5.
9. Gecele M, Francesetti G, Meluzzi A. Acetyl-L-Carnitine in Aged Subjects with Major Depression: Clinical Efficacy and Effects on the Circadian Rhythm of Cortisol. *Dementia and Geriatric Cognitive Disorders* 1991; **2**(6): 333-7.
10. Berlanga C, Ortega-Soto HA, Ontiveros M, Senties H. Efficacy of S-adenosyl-L-methionine in speeding the onset of action of imipramine. *Psychiatry research* 1992; **44**(3): 257-62.
11. De Vanna M, Rigamonti R. Oral S-adenosyl-L-methionine in depression. *Current Therapeutic Research* 1992; **52**(3): 478-85.
12. Fava M, Rosenbaum JF, Birnbaum R, Kelly K, Otto MW, MacLaughlin R. The thyrotropin response to thyrotropin-releasing hormone as a predictor of response to treatment in depressed outpatients. *Acta psychiatrica Scandinavica* 1992; **86**(1): 42-5.
13. Salmaggi P, Bressa GM, Nicchia G, Coniglio M, La Greca P, Le Grazie C. Double-blind, placebo-controlled study of S-adenosyl-L-methionine in depressed postmenopausal women. *Psychotherapy and psychosomatics* 1993; **59**(1): 34-40.
14. Bell KM, Potkin SG, Carreon D, Plon L. S-adenosylmethionine blood levels in major depression: changes with drug treatment. *Acta neurologica Scandinavica Supplementum* 1994; **154**: 15-8.
15. Hansgen KD, Vesper J, Ploch M. Multicenter double-blind study examining the antidepressant

effectiveness of the hypericum extract LI 160. *Journal of geriatric psychiatry and neurology* 1994; **7 Suppl 1**: S15-8.

16. Harrer G, Hubner WD, Podzuweit H. Effectiveness and tolerance of the hypericum extract LI 160 compared to maprotiline: a multicenter double-blind study. *Journal of geriatric psychiatry and neurology* 1994; **7 Suppl 1**: S24-8.
17. Hubner WD, Lande S, Podzuweit H. Hypericum treatment of mild depressions with somatic symptoms. *Journal of geriatric psychiatry and neurology* 1994; **7 Suppl 1**: S12-4.
18. Sommer H, Harrer G. Placebo-controlled double-blind study examining the effectiveness of an hypericum preparation in 105 mildly depressed patients. *Journal of geriatric psychiatry and neurology* 1994; **7 Suppl 1**: S9-11.
19. Vorbach EU, Hubner WD, Arnoldt KH. Effectiveness and tolerance of the hypericum extract LI 160 in comparison with imipramine: randomized double-blind study with 135 outpatients. *Journal of geriatric psychiatry and neurology* 1994; **7 Suppl 1**: S19-23.
20. Vorbach EU, Arnoldt KH, Hubner WD. Efficacy and tolerability of St. John's wort extract LI 160 versus imipramine in patients with severe depressive episodes according to ICD-10. *Pharmacopsychiatry* 1997; **30 Suppl 2**: 81-5.
21. Wheatley D. LI 160, an extract of St. John's wort, versus amitriptyline in mildly to moderately depressed outpatients--a controlled 6-week clinical trial. *Pharmacopsychiatry* 1997; **30 Suppl 2**: 77-80.
22. Laakmann G, Schule C, Baghai T, Kieser M. St. John's wort in mild to moderate depression: the relevance of hyperforin for the clinical efficacy. *Pharmacopsychiatry* 1998; **31 Suppl 1**: 54-9.
23. Schrader E, Meier B, Brattström A. Hypericum treatment of mild–moderate depression in a placebo–controlled study. A prospective, double–blind, randomized, placebo–controlled, multicentre study. 1998; **13**(3): 163-9.
24. Harrer G, Schmidt U, Kuhn U, Biller A. Comparison of equivalence between the St. John's wort extract LoHyp-57 and fluoxetine. *Arzneimittel-Forschung* 1999; **49**(4): 289-96.
25. Levine J, Mishori A, Susnosky M, Martin M, Belmaker RH. Combination of inositol and serotonin reuptake inhibitors in the treatment of depression. *Biological psychiatry* 1999; **45**(3): 270-3.
26. Nemets B, Mishory A, Levine J, Belmaker RHJJoNT. Inositol addition does not improve depression in SSRI treatment failures. 1999; **106**(7): 795-8.
27. Philipp M, Kohnen R, Hiller KO. Hypericum extract versus imipramine or placebo in patients with moderate depression: randomised multicentre study of treatment for eight weeks. *BMJ (Clinical research ed)* 1999; **319**(7224): 1534-8.
28. Brenner R, Azbel V, Madhusoodanan S, Pawlowska M. Comparison of an extract of hypericum (LI 160) and sertraline in the treatment of depression: a double-blind, randomized pilot study. *Clinical therapeutics* 2000; **22**(4): 411-9.

29. Coppen A, Bailey J. Enhancement of the antidepressant action of fluoxetine by folic acid: a randomised, placebo controlled trial. *Journal of affective disorders* 2000; **60**(2): 121-30.

30. Levitan RD, Shen JH, Jindal R, Driver HS, Kennedy SH, Shapiro CM. Preliminary randomized double-blind placebo-controlled trial of tryptophan combined with fluoxetine to treat major depressive disorder: antidepressant and hypnotic effects. *Journal of psychiatry & neuroscience : JPN* 2000; **25**(4): 337-46.

31. Schrader E. Equivalence of St John's wort extract (Ze 117) and fluoxetine: a randomized, controlled study in mild-moderate depression. *International clinical psychopharmacology* 2000; **15**(2): 61-8.

32. Woelk H. Comparison of St John's wort and imipramine for treating depression: randomised controlled trial. *BMJ (Clinical research ed)* 2000; **321**(7260): 536-9.

33. Kalb R, Trautmann-Sponsel RD, Kieser M. Efficacy and tolerability of hypericum extract WS 5572 versus placebo in mildly to moderately depressed patients. A randomized double-blind multicenter clinical trial. *Pharmacopsychiatry* 2001; **34**(3): 96-103.

34. Shelton RC, Keller MB, Gelenberg A, et al. Effectiveness of St John's wort in major depression: a randomized controlled trial. *Jama* 2001; **285**(15): 1978-86.

35. Behnke K, Jensen GS, Graubaum HJ, Gruenwald J. Hypericum perforatum versus fluoxetine in the treatment of mild to moderate depression. *Advances in therapy* 2002; **19**(1): 43-52.

36. Davidson J. Effect of Hypericum perforatum (St John's wort) in major depressive disorder: a randomized controlled trial. *Jama* 2002; **287**(14): 1807-14.

37. Delle Chiaie R, Pancheri P, Scapicchio P. Efficacy and tolerability of oral and intramuscular S-adenosyl-L-methionine 1,4-butanedisulfonate (SAMe) in the treatment of major depression: comparison with imipramine in 2 multicenter studies. *The American journal of clinical nutrition* 2002; **76**(5): 1172s-6s.

38. Lecrubier Y, Clerc G, Didi R, Kieser M. Efficacy of St. John's wort extract WS 5570 in major depression: a double-blind, placebo-controlled trial. *The American journal of psychiatry* 2002; **159**(8): 1361-6.

39. Nemets B, Stahl Z, Belmaker RH. Addition of omega-3 fatty acid to maintenance medication treatment for recurrent unipolar depressive disorder. *The American journal of psychiatry* 2002; **159**(3): 477-9.

40. Pancheri P, Scapicchio P, Chiaie RD. A double-blind, randomized parallel-group, efficacy and safety study of intramuscular S-adenosyl-L-methionine 1,4-butanedisulphonate (SAMe) versus imipramine in patients with major depressive disorder. *The international journal of neuropsychopharmacology* 2002; **5**(4): 287-94.

41. Peet M, Horrobin DF. A dose-ranging study of the effects of ethyl-eicosapentaenoate in patients with ongoing depression despite apparently adequate treatment with standard drugs. *Archives of general psychiatry* 2002; **59**(10): 913-9.

42. van Gurp G, Meterissian GB, Haiek LN, McCusker J, Bellavance F. St John's wort or

sertraline? Randomized controlled trial in primary care. *Canadian family physician Medecin de famille canadien* 2002; **48**: 905-12.

43. Akhondzadeh S, Kashani L, Fotouhi A, et al. Comparison of Lavandula angustifolia Mill. tincture and imipramine in the treatment of mild to moderate depression: a double-blind, randomized trial. *Progress in neuro-psychopharmacology & biological psychiatry* 2003; **27**(1): 123-7.
44. Marangell LB, Martinez JM, Zboyan HA, Kertz B, Kim HF, Puryear LJ. A double-blind, placebo-controlled study of the omega-3 fatty acid docosahexaenoic acid in the treatment of major depression. *The American journal of psychiatry* 2003; **160**(5): 996-8.
45. Nowak G, Siwek M, Dudek D, Zieba A, Pilc A. Effect of zinc supplementation on antidepressant therapy in unipolar depression: a preliminary placebo-controlled study. *Polish journal of pharmacology* 2003; **55**(6): 1143-7.
46. Su KP, Huang SY, Chiu CC, Shen WW. Omega-3 fatty acids in major depressive disorder. A preliminary double-blind, placebo-controlled trial. *European neuropsychopharmacology : the journal of the European College of Neuropsychopharmacology* 2003; **13**(4): 267-71.
47. Zanarini MC, Frankenburg FR. omega-3 Fatty acid treatment of women with borderline personality disorder: a double-blind, placebo-controlled pilot study. *The American journal of psychiatry* 2003; **160**(1): 167-9.
48. Akhondzadeh S, Fallah-Pour H, Afkham K, Jamshidi AH, Khalighi-Cigaroudi F. Comparison of Crocus sativus L. and imipramine in the treatment of mild to moderate depression: a pilot double-blind randomized trial [ISRCTN45683816]. *BMC complementary and alternative medicine* 2004; **4**: 12.
49. Uebelhack R, Gruenwald J, Graubaum HJ, Busch R. Efficacy and tolerability of Hypericum extract STW 3-VI in patients with moderate depression: a double-blind, randomized, placebo-controlled clinical trial. *Advances in therapy* 2004; **21**(4): 265-75.
50. Akhondzadeh S, Tahmacebi-Pour N, Noorbala AA, et al. Crocus sativus L. in the treatment of mild to moderate depression: a double-blind, randomized and placebo-controlled trial. *Phytotherapy research : PTR* 2005; **19**(2): 148-51.
51. Bjerkenstedt L, Edman GV, Alken RG, Mannel M. Hypericum extract LI 160 and fluoxetine in mild to moderate depression: a randomized, placebo-controlled multi-center study in outpatients. *European archives of psychiatry and clinical neuroscience* 2005; **255**(1): 40-7.
52. Docherty JP, Sack DA, Roffman M, Finch M, Komorowski JR. A double-blind, placebo-controlled, exploratory trial of chromium picolinate in atypical depression: Effect on carbohydrate craving. *Journal of Psychiatric Practice* 2005; **11**(5): 302-14.
53. Fava M, Alpert J, Nierenberg AA, et al. A Double-blind, randomized trial of St John's wort, fluoxetine, and placebo in major depressive disorder. *Journal of clinical psychopharmacology* 2005; **25**(5): 441-7.
54. Gastpar M, Singer A, Zeller K. Efficacy and tolerability of hypericum extract STW3 in long-

term treatment with a once-daily dosage in comparison with sertraline. *Pharmacopsychiatry* 2005; **38**(2): 78-86.

55. Noorbala AA, Akhondzadeh S, Tahmacebi-Pour N, Jamshidi AH. Hydro-alcoholic extract of Crocus sativus L. versus fluoxetine in the treatment of mild to moderate depression: a double-blind, randomized pilot trial. *Journal of ethnopharmacology* 2005; **97**(2): 281-4.
56. Silvers KM, Woolley CC, Hamilton FC, Watts PM, Watson RA. Randomised double-blind placebo-controlled trial of fish oil in the treatment of depression. *Prostaglandins, leukotrienes, and essential fatty acids* 2005; **72**(3): 211-8.
57. Szegedi A, Kohnen R, Dienel A, Kieser M. Acute treatment of moderate to severe depression with hypericum extract WS 5570 (St John's wort): randomised controlled double blind non-inferiority trial versus paroxetine. *BMJ (Clinical research ed)* 2005; **330**(7490): 503.
58. Gastpar M, Singer A, Zeller K. Comparative efficacy and safety of a once-daily dosage of hypericum extract STW3-VI and citalopram in patients with moderate depression: a double-blind, randomised, multicentre, placebo-controlled study. *Pharmacopsychiatry* 2006; **39**(2): 66-75.
59. Kasper S, Anghelescu IG, Szegedi A, Dienel A, Kieser M. Superior efficacy of St John's wort extract WS 5570 compared to placebo in patients with major depression: a randomized, double-blind, placebo-controlled, multi-center trial [ISRCTN77277298]. *BMC medicine* 2006; **4**: 14.
60. Moreno RA, Teng CT, Almeida KM, Tavares Junior H. Hypericum perforatum versus fluoxetine in the treatment of mild to moderate depression: a randomized double-blind trial in a Brazilian sample. *Revista brasileira de psiquiatria (Sao Paulo, Brazil : 1999)* 2006; **28**(1): 29-32.
61. Moshiri E, Basti AA, Noorbala AA, Jamshidi AH, Hesameddin Abbasi S, Akhondzadeh S. Crocus sativus L. (petal) in the treatment of mild-to-moderate depression: a double-blind, randomized and placebo-controlled trial. *Phytomedicine : international journal of phytotherapy and phytopharmacology* 2006; **13**(9-10): 607-11.
62. Randlov C, Mehlsen J, Thomsen CF, Hedman C, von Fircks H, Winther K. The efficacy of St. John's Wort in patients with minor depressive symptoms or dysthymia--a double-blind placebo-controlled study. *Phytomedicine : international journal of phytotherapy and phytopharmacology* 2006; **13**(4): 215-21.
63. Sayyah M, Sayyah M, Kamalinejad M. A preliminary randomized double blind clinical trial on the efficacy of aqueous extract of Echium amoenum in the treatment of mild to moderate major depression. *Progress in neuro-psychopharmacology & biological psychiatry* 2006; **30**(1): 166-9.
64. Akhondzadeh Basti A, Moshiri E, Noorbala AA, Jamshidi AH, Abbasi SH, Akhondzadeh S. Comparison of petal of Crocus sativus L. and fluoxetine in the treatment of depressed outpatients: a pilot double-blind randomized trial. *Progress in neuro-psychopharmacology &*

biological psychiatry 2007; **31**(2): 439-42.

65. Darbinyan V, Aslanyan G, Amroyan E, Gabrielyan E, Malmstrom C, Panossian A. Clinical trial of Rhodiola rosea L. extract SHR-5 in the treatment of mild to moderate depression. *Nordic journal of psychiatry* 2007; **61**(5): 343-8.

66. Grenyer BF, Crowe T, Meyer B, et al. Fish oil supplementation in the treatment of major depression: a randomised double-blind placebo-controlled trial. *Progress in neuro-psychopharmacology & biological psychiatry* 2007; **31**(7): 1393-6.

67. Ille R, Spona J, Zickl M, et al. "Add-On"-therapy with an individualized preparation consisting of free amino acids for patients with a major depression. *European archives of psychiatry and clinical neuroscience* 2007; **257**(4): 222-9.

68. Barragan-Rodriguez L, Rodriguez-Moran M, Guerrero-Romero F. Efficacy and safety of oral magnesium supplementation in the treatment of depression in the elderly with type 2 diabetes: a randomized, equivalent trial. *Magnesium research* 2008; **21**(4): 218-23.

69. da Silva TM, Munhoz RP, Alvarez C, et al. Depression in Parkinson's disease: a double-blind, randomized, placebo-controlled pilot study of omega-3 fatty-acid supplementation. *Journal of affective disorders* 2008; **111**(2-3): 351-9.

70. Freeman MP, Davis M, Sinha P, Wisner KL, Hibbeln JR, Gelenberg AJ. Omega-3 fatty acids and supportive psychotherapy for perinatal depression: a randomized placebo-controlled study. *Journal of affective disorders* 2008; **110**(1-2): 142-8.

71. Jazayeri S, Tehrani-Doost M, Keshavarz SA, et al. Comparison of therapeutic effects of omega-3 fatty acid eicosapentaenoic acid and fluoxetine, separately and in combination, in major depressive disorder. *The Australian and New Zealand journal of psychiatry* 2008; **42**(3): 192-8.

72. Raza-ur-Rahman, Ansari MA, Hayder Z, Siddiqui AA, Bukhari IA, Qayyum MA. Double blind placebo controlled clinical trial examining the effectiveness of St. John ' s wort (hypericum perforatum) in mild to moderate depression. *Journal of Pakistan Psychiatric Society* 2008; **5**: 105 – 11.

73. Rees AM, Austin MP, Parker GB. Omega-3 fatty acids as a treatment for perinatal depression: randomized double-blind placebo-controlled trial. *The Australian and New Zealand journal of psychiatry* 2008; **42**(3): 199-205.

74. Resler G, Lavie R, Campos J, et al. Effect of folic acid combined with fluoxetine in patients with major depression on plasma homocysteine and vitamin B12, and serotonin levels in lymphocytes. *Neuroimmunomodulation* 2008; **15**(3): 145-52.

75. Rogers PJ, Appleton KM, Kessler D, et al. No effect of n-3 long-chain polyunsaturated fatty acid (EPA and DHA) supplementation on depressed mood and cognitive function: a randomised controlled trial. *The British journal of nutrition* 2008; **99**(2): 421-31.

76. Su KP, Huang SY, Chiu TH, et al. Omega-3 fatty acids for major depressive disorder during pregnancy: results from a randomized, double-blind, placebo-controlled trial. *The Journal of*

clinical psychiatry 2008; **69**(4): 644-51.

77. Başoğlu C, Ateş MA, Algül A, et al. Adjuvant folate with escitalopram treatment and homocystein, folate, vitamin B-12 levels in patients with major depressive disorder. *Klinik Psikofarmakoloji Bulteni* 2009; **19**(2): 135-42.

78. Carney RM, Freedland KE, Rubin EH, Rich MW, Steinmeyer BC, Harris WS. Omega-3 augmentation of sertraline in treatment of depression in patients with coronary heart disease: a randomized controlled trial. *Jama* 2009; **302**(15): 1651-7.

79. Lucas M, Asselin G, Merette C, Poulin MJ, Dodin S. Ethyl-eicosapentaenoic acid for the treatment of psychological distress and depressive symptoms in middle-aged women: a double-blind, placebo-controlled, randomized clinical trial. *The American journal of clinical nutrition* 2009; **89**(2): 641-51.

80. Mischoulon D, Papakostas GI, Dording CM, et al. A double-blind, randomized controlled trial of ethyl-eicosapentaenoate for major depressive disorder. *The Journal of clinical psychiatry* 2009; **70**(12): 1636-44.

81. Sarris J, Kavanagh DJ, Deed G, Bone KM. St. John's wort and Kava in treating major depressive disorder with comorbid anxiety: a randomised double-blind placebo-controlled pilot trial. *Human psychopharmacology* 2009; **24**(1): 41-8.

82. Siwek M, Dudek D, Paul IA, et al. Zinc supplementation augments efficacy of imipramine in treatment resistant patients: a double blind, placebo-controlled study. *Journal of affective disorders* 2009; **118**(1-3): 187-95.

83. Bot M, Pouwer F, Assies J, et al. Eicosapentaenoic acid as an add-on to antidepressant medication for co-morbid major depression in patients with diabetes mellitus: a randomized, double-blind placebo-controlled study. *Journal of affective disorders* 2010; **126**(1-2): 282-6.

84. Liu J, Meng ZW, Li LY, Feng LS, Yang H. Effect of St. John's wort extract on depressive disorder in elderly patients with unstable angina. *World J Emerg Med* 2010; **1**(1): 41-4.

85. Mannel M, Kuhn U, Schmidt U, Ploch M, Murck H. St. John's wort extract LI160 for the treatment of depression with atypical features - a double-blind, randomized, and placebo-controlled trial. *Journal of psychiatric research* 2010; **44**(12): 760-7.

86. Papakostas GI, Mischoulon D, Shyu I, Alpert JE, Fava M. S-adenosyl methionine (SAMe) augmentation of serotonin reuptake inhibitors for antidepressant nonresponders with major depressive disorder a double-blind, randomized clinical trial. 2010.

87. Rondanelli M, Giacosa A, Opizzi A, et al. Effect of omega-3 fatty acids supplementation on depressive symptoms and on health-related quality of life in the treatment of elderly women with depression: a double-blind, placebo-controlled, randomized clinical trial. *Journal of the American College of Nutrition* 2010; **29**(1): 55-64.

88. Lesperance F, Frasure-Smith N, St-Andre E, Turecki G, Lesperance P, Wisniewski SR. The efficacy of omega-3 supplementation for major depression: a randomized controlled trial. *The Journal of clinical psychiatry* 2011; **72**(8): 1054-62.

89. Rapaport MH, Nierenberg AA, Howland R, Dording C, Schettler PJ, Mischoulon D. The treatment of minor depression with St. John's Wort or citalopram: failure to show benefit over placebo. *Journal of psychiatric research* 2011; **45**(7): 931-41.
90. Rondanelli M, Giacosa A, Opizzi A, et al. Long chain omega 3 polyunsaturated fatty acids supplementation in the treatment of elderly depression: effects on depressive symptoms, on phospholipids fatty acids profile and on health-related quality of life. *The journal of nutrition, health & aging* 2011; **15**(1): 37-44.
91. Gertsik L, Poland RE, Bresee C, Rapaport MH. Omega-3 fatty acid augmentation of citalopram treatment for patients with major depressive disorder. *Journal of clinical psychopharmacology* 2012; **32**(1): 61-4.
92. Lyoo IK, Yoon S, Kim TS, et al. A randomized, double-blind placebo-controlled trial of oral creatine monohydrate augmentation for enhanced response to a selective serotonin reuptake inhibitor in women with major depressive disorder. *The American journal of psychiatry* 2012; **169**(9): 937-45.
93. Pakseresht S, Boustani H, Azemi ME, Nilsaz J, Babapour R, Haghdust MR. Evaluation of Pharmaceutical Products of St. John's Wort Efficacy Added on Tricyclic Antidepressants in treating Major Depressive Disorder: A Double Blind Randomized Control Trial. *Jundishapur J Nat Pharm Prod* 2012; **7**(3): 106-10.
94. Papakostas GI, Shelton RC, Zajecka JM, et al. L-methylfolate as adjunctive therapy for SSRI-resistant major depression: results of two randomized, double-blind, parallel-sequential trials. *The American journal of psychiatry* 2012; **169**(12): 1267-74.
95. Rizzo AM, Corsetto PA, Montorfano G, et al. Comparison between the AA/EPA ratio in depressed and non depressed elderly females: omega-3 fatty acid supplementation correlates with improved symptoms but does not change immunological parameters. *Nutrition journal* 2012; **11**: 82.
96. Sarris J, Fava M, Schweitzer I, Mischoulon D. St John's wort (Hypericum perforatum) versus sertraline and placebo in major depressive disorder: continuation data from a 26-week RCT. *Pharmacopsychiatry* 2012; **45**(7): 275-8.
97. Bergman J, Miodownik C, Bersudsky Y, et al. Curcumin as an add-on to antidepressive treatment: a randomized, double-blind, placebo-controlled, pilot clinical study. *Clinical neuropharmacology* 2013; **36**(3): 73-7.
98. Bersani G, Meco G, Denaro A, et al. L-Acetylcarnitine in dysthymic disorder in elderly patients: a double-blind, multicenter, controlled randomized study vs. fluoxetine. *European neuropsychopharmacology : the journal of the European College of Neuropsychopharmacology* 2013; **23**(10): 1219-25.
99. Khajehnasiri F, Mortazavi SB, Allameh A, Akhondzadeh S. Effect of omega-3 and ascorbic acid on inflammation markers in depressed shift workers in Shahid Tondgoyan Oil Refinery, Iran: a randomized double-blind placebo-controlled study. *Journal of clinical biochemistry*

and nutrition 2013; **53**(1): 36-40.

100. Khoraminya N, Tehrani-Doost M, Jazayeri S, Hosseini A, Djazayery A. Therapeutic effects of vitamin D as adjunctive therapy to fluoxetine in patients with major depressive disorder. *The Australian and New Zealand journal of psychiatry* 2013; **47**(3): 271-5.

101. Lewis JE, Tiozzo E, Melillo AB, et al. The effect of methylated vitamin B complex on depressive and anxiety symptoms and quality of life in adults with depression. *ISRN Psychiatry* 2013; **2013**: 621453-.

102. Mozaffari-Khosravi H, Nabizade L, Yassini-Ardakani SM, Hadinedoushan H, Barzegar K. The effect of 2 different single injections of high dose of vitamin D on improving the depression in depressed patients with vitamin D deficiency: a randomized clinical trial. *Journal of clinical psychopharmacology* 2013; **33**(3): 378-85.

103. Mozaffari-Khosravi H, Yassini-Ardakani M, Karamati M, Shariati-Bafghi SE. Eicosapentaenoic acid versus docosahexaenoic acid in mild-to-moderate depression: a randomized, double-blind, placebo-controlled trial. *European neuropsychopharmacology : the journal of the European College of Neuropsychopharmacology* 2013; **23**(7): 636-44.

104. Mozurkewich EL, Clinton CM, Chilimigras JL, et al. The Mothers, Omega-3, and Mental Health Study: a double-blind, randomized controlled trial. *American journal of obstetrics and gynecology* 2013; **208**(4): 313.e1-9.

105. Nemets B, Levine J. A pilot dose-finding clinical trial of creatine monohydrate augmentation to SSRIs/SNRIs/NASA antidepressant treatment in major depression. *International clinical psychopharmacology* 2013; **28**(3): 127-33.

106. Safa M, Fallah Tafti S, Ghassem Boroujerdi F, Talischi F. Clinical trial in the treatment of 80 Iranian patients with major depression disorder by the combination of omega 3 fatty acid and a selective serotonin reuptake inhibitor. *Ther Adv Psychopharmacol* 2013; **3**(4): 186-90.

107. Syed EU, Wasay M, Awan S. Vitamin B12 supplementation in treating major depressive disorder: a randomized controlled trial. *Open Neurol J* 2013; **7**: 44-8.

108. Almeida OP, Ford AH, Hirani V, et al. B vitamins to enhance treatment response to antidepressants in middle-aged and older adults: results from the B-VITAGE randomised, double-blind, placebo-controlled trial. *The British journal of psychiatry : the journal of mental science* 2014; **205**(6): 450-7.

109. Bedson E, Bell D, Carr D, et al. Folate Augmentation of Treatment--Evaluation for Depression (FolATED): randomised trial and economic evaluation. *Health technology assessment (Winchester, England)* 2014; **18**(48): vii-viii, 1-159.

110. Dashti-Khavidaki S, Gharekhani A, Khatami MR, et al. Effects of omega-3 fatty acids on depression and quality of life in maintenance hemodialysis patients. *American journal of therapeutics* 2014; **21**(4): 275-87.

111. Kaviani M, Saniee L, Azima S, Sharif F, Sayadi M. The Effect of Omega-3 Fatty Acid Supplementation on Maternal Depression during Pregnancy: A Double Blind Randomized

Controlled Clinical Trial. *Int J Community Based Nurs Midwifery* 2014; **2**(3): 142-7.

112. Lopresti AL, Maes M, Maker GL, Hood SD, Drummond PD. Curcumin for the treatment of major depression: a randomised, double-blind, placebo controlled study. *Journal of affective disorders* 2014; **167**: 368-75.

113. Mischoulon D, Price LH, Carpenter LL, et al. A double-blind, randomized, placebo-controlled clinical trial of S-adenosyl-L-methionine (SAMe) versus escitalopram in major depressive disorder. *The Journal of clinical psychiatry* 2014; **75**(4): 370-6.

114. Sanmukhani J, Satodia V, Trivedi J, et al. Efficacy and safety of curcumin in major depressive disorder: a randomized controlled trial. *Phytotherapy research : PTR* 2014; **28**(4): 579-85.

115. Sarris J, Papakostas GI, Vitolo O, Fava M, Mischoulon D. S-adenosyl methionine (SAMe) versus escitalopram and placebo in major depression RCT: efficacy and effects of histamine and carnitine as moderators of response. *Journal of affective disorders* 2014; **164**: 76-81.

116. Shahmansouri N, Farokhnia M, Abbasi SH, et al. A randomized, double-blind, clinical trial comparing the efficacy and safety of Crocus sativus L. with fluoxetine for improving mild to moderate depression in post percutaneous coronary intervention patients. *Journal of affective disorders* 2014; **155**: 216-22.

117. Gavrilova SI, Kalyn YB, Safarova TP, et al. Optimization of the efficacy and safety of antidepressant therapy in patients of a geriatric psychiatric unit. *Zh Nevrol Psikhiatr Im S S Korsakova* 2015; **115**(6): 24-32.

118. Ginty AT, Conklin SM. Short-term supplementation of acute long-chain omega-3 polyunsaturated fatty acids may alter depression status and decrease symptomology among young adults with depression: A preliminary randomized and placebo controlled trial. *Psychiatry research* 2015; **229**(1-2): 485-9.

119. Mao JJ, Xie SX, Zee J, et al. Rhodiola rosea versus sertraline for major depressive disorder: A randomized placebo-controlled trial. *Phytomedicine : international journal of phytotherapy and phytopharmacology* 2015; **22**(3): 394-9.

120. Mischoulon D, Nierenberg AA, Schettler PJ, et al. A double-blind, randomized controlled clinical trial comparing eicosapentaenoic acid versus docosahexaenoic acid for depression. *The Journal of clinical psychiatry* 2015; **76**(1): 54-61.

121. Panahi Y, Badeli R, Karami GR, Badeli Z, Sahebkar A. A randomized controlled trial of 6-week Chlorella vulgaris supplementation in patients with major depressive disorder. *Complementary therapies in medicine* 2015; **23**(4): 598-602.

122. Panahi Y, Badeli R, Karami GR, Sahebkar A. Investigation of the efficacy of adjunctive therapy with bioavailability-boosted curcuminoids in major depressive disorder. *Phytotherapy research : PTR* 2015; **29**(1): 17-21.

123. Park Y, Park YS, Kim SH, Oh DH, Park YC. Supplementation of n-3 Polyunsaturated Fatty Acids for Major Depressive Disorder: A Randomized, Double-Blind, 12-Week, Placebo-Controlled Trial in Korea. *Annals of nutrition & metabolism* 2015; **66**(2-3): 141-8.

124. Sahraian A, Ghanizadeh A, Kazemeini F. Vitamin C as an adjuvant for treating major depressive disorder and suicidal behavior, a randomized placebo-controlled clinical trial. *Trials* 2015; **16**: 94.

125. Talaei A, Hassanpour Moghadam M, Sajadi Tabassi SA, Mohajeri SA. Crocin, the main active saffron constituent, as an adjunctive treatment in major depressive disorder: a randomized, double-blind, placebo-controlled, pilot clinical trial. *Journal of affective disorders* 2015; **174**: 51-6.

126. Yu JJ, Pei LB, Zhang Y, Wen ZY, Yang JL. Chronic Supplementation of Curcumin Enhances the Efficacy of Antidepressants in Major Depressive Disorder: A Randomized, Double-Blind, Placebo-Controlled Pilot Study. *Journal of clinical psychopharmacology* 2015; **35**(4): 406-10.

127. Akkasheh G, Kashani-Poor Z, Tajabadi-Ebrahimi M, et al. Clinical and metabolic response to probiotic administration in patients with major depressive disorder: A randomized, double-blind, placebo-controlled trial. *Nutrition (Burbank, Los Angeles County, Calif)* 2016; **32**(3): 315-20.

128. Ghaleiha A, Davari H, Jahangard L, et al. Adjuvant thiamine improved standard treatment in patients with major depressive disorder: results from a randomized, double-blind, and placebo-controlled clinical trial. *European archives of psychiatry and clinical neuroscience* 2016; **266**(8): 695-702.

129. Kolouri S, Firoozabadi A, Salehi A, et al. Nepeta menthoides Boiss. & Buhse freeze-dried aqueous extract versus sertraline in the treatment of major depression: A double blind randomized controlled trial. *Complementary therapies in medicine* 2016; **26**: 164-70.

130. Masoumi SZ, Kazemi F, Tavakolian S, et al. Effect of Citalopram in Combination with Omega-3 on Depression in Post-menopausal Women: A Triple Blind Randomized Controlled Trial. *Journal of clinical and diagnostic research : JCDR* 2016; **10**(10): Qc01-qc5.

131. Rapaport MH, Nierenberg AA, Schettler PJ, et al. Inflammation as a predictive biomarker for response to omega-3 fatty acids in major depressive disorder: a proof-of-concept study. *Molecular psychiatry* 2016; **21**(1): 71-9.

132. Ravi S, Khalili H, Abbasian L, Arbabi M, Ghaeli P. Effect of Omega-3 Fatty Acids on Depressive Symptoms in HIV-Positive Individuals: A Randomized, Placebo-Controlled Clinical Trial. *The Annals of pharmacotherapy* 2016; **50**(10): 797-807.

133. Sepehrmanesh Z, Abedi F. Acid Folic Supplementation in Major Depressive Disorder Treatment: A Double-Blind Randomized Clinical Trial. *Iranian Red Crescent Medical Journal* 2016; **19**(2).

134. Sepehrmanesh Z, Kolahdooz F, Abedi F, et al. Vitamin D Supplementation Affects the Beck Depression Inventory, Insulin Resistance, and Biomarkers of Oxidative Stress in Patients with Major Depressive Disorder: A Randomized, Controlled Clinical Trial. *The Journal of nutrition* 2016; **146**(2): 243-8.

135. Shinto L, Marracci G, Mohr DC, et al. Omega-3 Fatty Acids for Depression in Multiple

Sclerosis: A Randomized Pilot Study. *Plos One* 2016; **11**(1): e0147195-e.

136. Wang Y, Liu Y, Lian Y, Li N, Liu H, Li G. Efficacy of High-Dose Supplementation With Oral Vitamin D3 on Depressive Symptoms in Dialysis Patients With Vitamin D3 Insufficiency: A Prospective, Randomized, Double-Blind Study. *Journal of clinical psychopharmacology* 2016; **36**(3): 229-35.

137. Abedimanesh N, Ostadrahimi A, Bathaie SZ, et al. Effects of Saffron Aqueous Extract and Its Main Constituent, Crocin, on Health-Related Quality of Life, Depression, and Sexual Desire in Coronary Artery Disease Patients: A Double-Blind, Placebo-Controlled, Randomized Clinical Trial. *Iran Red Crescent Med J* 2017; **19**(9): e13676.

138. Ghajar A, Neishabouri SM, Velayati N, et al. Crocus sativus L. versus Citalopram in the Treatment of Major Depressive Disorder with Anxious Distress: A Double-Blind, Controlled Clinical Trial. *Pharmacopsychiatry* 2017; **50**(4): 152-60.

139. Kashani L, Eslatmanesh S, Saedi N, et al. Comparison of Saffron versus Fluoxetine in Treatment of Mild to Moderate Postpartum Depression: A Double-Blind, Randomized Clinical Trial. *Pharmacopsychiatry* 2017; **50**(2): 64-8.

140. Lopresti AL, Drummond PD. Efficacy of curcumin, and a saffron/curcumin combination for the treatment of major depression: A randomised, double-blind, placebo-controlled study. *Journal of affective disorders* 2017; **207**: 188-96.

141. Rajizadeh A, Mozaffari-Khosravi H, Yassini-Ardakani M, Dehghani A. Effect of magnesium supplementation on depression status in depressed patients with magnesium deficiency: A randomized, double-blind, placebo-controlled trial. *Nutrition (Burbank, Los Angeles County, Calif)* 2017; **35**: 56-60.

142. Romijn AR, Rucklidge JJ, Kuijer RG, Frampton C. A double-blind, randomized, placebo-controlled trial of Lactobacillus helveticus and Bifidobacterium longum for the symptoms of depression. *The Australian and New Zealand journal of psychiatry* 2017; **51**(8): 810-21.

143. Sheikh M, Hantoushzadeh S, Shariat M, Farahani Z, Ebrahiminasab O. The efficacy of early iron supplementation on postpartum depression, a randomized double-blind placebo-controlled trial. *European journal of nutrition* 2017; **56**(2): 901-8.

144. Tabeshpour J, Sobhani F, Sadjadi SA, et al. A double-blind, randomized, placebo-controlled trial of saffron stigma (Crocus sativus L.) in mothers suffering from mild-to-moderate postpartum depression. *Phytomedicine : international journal of phytotherapy and phytopharmacology* 2017; **36**: 145-52.

145. Alavi NM, Khademalhoseini S, Vakili Z, Assarian F. Effect of vitamin D supplementation on depression in elderly patients: A randomized clinical trial. *Clinical nutrition (Edinburgh, Scotland)* 2018.

146. Dai CX, Hu CC, Shang YS, Xie J. Role of Ginkgo biloba extract as an adjunctive treatment of elderly patients with depression and on the expression of serum S100B. *Medicine* 2018; **97**(39): e12421.

147. Ghazizadeh-Hashemi M, Ghajar A, Shalbafan MR, et al. Palmitoylethanolamide as adjunctive therapy in major depressive disorder: A double-blind, randomized and placebo-controlled trial. *Journal of affective disorders* 2018; **232**: 127-33.

148. Ghorbani Z, Nazari S, Etesam F, Nourimajd S, Ahmadpanah M, Razeghi S. The Effect of Synbiotic as an Adjuvant Therapy to Fluoxetine in Moderate Depression: A Randomized Multicenter Trial. *Archives of Neuroscience* 2018; **In Press**.

149. Jahangard L, Sadeghi A, Ahmadpanah M, et al. Influence of adjuvant omega-3-polyunsaturated fatty acids on depression, sleep, and emotion regulation among outpatients with major depressive disorders - Results from a double-blind, randomized and placebo-controlled clinical trial. *Journal of psychiatric research* 2018; **107**: 48-56.

150. Kanchanatawan B, Tangwongchai S, Sughondhabhirom A, et al. Add-on Treatment with Curcumin Has Antidepressive Effects in Thai Patients with Major Depression: Results of a Randomized Double-Blind Placebo-Controlled Study. *Neurotoxicity research* 2018; **33**(3): 621-33.

151. Kashani L, Esalatmanesh S, Eftekhari F, et al. Efficacy of Crocus sativus (saffron) in treatment of major depressive disorder associated with post-menopausal hot flashes: a double-blind, randomized, placebo-controlled trial. *Archives of gynecology and obstetrics* 2018; **297**(3): 717-24.

152. Majeed M, Nagabhushanam K, Arumugam S, Majeed S, Ali F. Bacillus coagulans MTCC 5856 for the management of major depression with irritable bowel syndrome: a randomised, double-blind, placebo controlled, multi-centre, pilot clinical study. *Food & nutrition research* 2018; **62**.

153. Opiyo RO, Nyasulu PS, Koigi RK, Obondo A, Ogoyi D, Kogi-Makau W. Effect of fish oil omega-3 fatty acids on reduction of depressive symptoms among HIV-seropositive pregnant women: a randomized, double-blind controlled trial. *Ann Gen Psychiatry* 2018; **17**: 49-.

154. Ryszewska-Pokrasniewicz B, Mach A, Skalski M, et al. Effects of Magnesium Supplementation on Unipolar Depression: A Placebo-Controlled Study and Review of the Importance of Dosing and Magnesium Status in the Therapeutic Response. *Nutrients* 2018; **10**(8).

155. Sarris J, Byrne GJ, Bousman C, et al. Adjunctive S-adenosylmethionine (SAMe) in treating non-remittent major depressive disorder: An 8-week double-blind, randomized, controlled trial<sup/>. *European neuropsychopharmacology : the journal of the European College of Neuropsychopharmacology* 2018; **28**(10): 1126-36.

156. Targum SD, Cameron BR, Ferreira L, MacDonald ID. An augmentation study of MSI-195 (S-adenosylmethionine) in Major Depressive Disorder. *Journal of psychiatric research* 2018; **107**: 86-96.

157. Zhang L, Wang S, Zhu Y, Yang T. Vitamin D3 as adjunctive therapy in the treatment of depression in tuberculosis patients: a short-term pilot randomized double-blind controlled

study. *Neuropsychiatric disease and treatment* 2018; **14**: 3103-9.

158. Carney RM, Freedland KE, Rubin EH, Rich MW, Steinmeyer BC, Harris WS. A Randomized Placebo-Controlled Trial of Omega-3 and Sertraline in Depressed Patients With or at Risk for Coronary Heart Disease. *The Journal of clinical psychiatry* 2019; **80**(4).

159. Chahwan B, Kwan S, Isik A, van Hemert S, Burke C, Roberts L. Gut feelings: A randomised, triple-blind, placebo-controlled trial of probiotics for depressive symptoms. *Journal of affective disorders* 2019; **253**: 317-26.

160. Chang JP, Chang SS, Yang HT, et al. Omega-3 polyunsaturated fatty acids in cardiovascular diseases comorbid major depressive disorder - Results from a randomized controlled trial. *Brain, behavior, and immunity* 2019.

161. Hansen JP, Pareek M, Hvolby A, et al. Vitamin D3 supplementation and treatment outcomes in patients with depression (D3-vit-dep). *BMC research notes* 2019; **12**(1): 203.

162. Kazemi A, Noorbala AA, Azam K, Eskandari MH, Djafarian K. Effect of probiotic and prebiotic vs placebo on psychological outcomes in patients with major depressive disorder: A randomized clinical trial. *Clinical nutrition (Edinburgh, Scotland)* 2019; **38**(2): 522-8.

163. Liang ZH, Jia YB, Wang ML, et al. Efficacy of ginkgo biloba extract as augmentation of venlafaxine in treating post-stroke depression. *Neuropsychiatric disease and treatment* 2019; **15**: 2551-7.

164. Nishi D, Su KP, Usuda K, et al. The Efficacy of Omega-3 Fatty Acids for Depressive Symptoms among Pregnant Women in Japan and Taiwan: A Randomized, Double-Blind, Placebo-Controlled Trial (SYNCHRO; NCT01948596). *Psychotherapy and psychosomatics* 2019; **88**(2): 122-4.

165. Rudzki L, Ostrowska L, Pawlak D, et al. Probiotic Lactobacillus Plantarum 299v decreases kynurenine concentration and improves cognitive functions in patients with major depression: A double-blind, randomized, placebo controlled study. *Psychoneuroendocrinology* 2019; **100**: 213-22.

166. Sarris J, Byrne GJ, Stough C, et al. Nutraceuticals for major depressive disorder- more is not merrier: An 8-week double-blind, randomised, controlled trial. *Journal of affective disorders* 2019; **245**: 1007-15.

167. Tayama J, Ogawa S, Nakaya N, et al. Omega-3 polyunsaturated fatty acids and psychological intervention for workers with mild to moderate depression: A double-blind randomized controlled trial. *Journal of affective disorders* 2019; **245**: 364-70.

168. Akhondzadeh S, Mostafavi S-A, Keshavarz SA, Mohammadi MR, Hosseini S, Eshraghian MR. A placebo controlled randomized clinical trial of Crocus sativus L. (saffron) on depression and food craving among overweight women with mild to moderate depression. *Journal of Clinical Pharmacy and Therapeutics* 2020; **45**(1): 134-43.

169. Amini S, Amani R, Jafarirad S, Cheraghian B, Sayyah M, Hemmati AA. The effect of vitamin D and calcium supplementation on inflammatory biomarkers, estradiol levels and severity of

symptoms in women with postpartum depression: a randomized double-blind clinical trial. *Nutr Neurosci* 2020: 1-11.

170. Asadi S, Gholami MS, Siassi F, Qorbani M, Sotoudeh G. Beneficial effects of nano-curcumin supplement on depression and anxiety in diabetic patients with peripheral neuropathy: A randomized, double-blind, placebo-controlled clinical trial. *Phytotherapy research : PTR* 2020; **34**(4): 896-903.

171. Kaviani M, Nikooyeh B, Zand H, Yaghmaei P, Neyestani TR. Effects of vitamin D supplementation on depression and some involved neurotransmitters. *Journal of affective disorders* 2020; **269**: 28-35.

172. Reininghaus EZ, Platzer M, Kohlhammer-Dohr A, et al. PROVIT: Supplementary Probiotic Treatment and Vitamin B7 in Depression-A Randomized Controlled Trial. *Nutrients* 2020; **12**(11).

173. Sakurai H, L LC, A RT, et al. Dose increase of S-Adenosyl-Methionine and escitalopram in a randomized clinical trial for major depressive disorder. *Journal of affective disorders* 2020; **262**: 118-25.

174. Sarris J, Murphy J, Stough C, et al. S-Adenosylmethionine (SAMe) monotherapy for depression: an 8-week double-blind, randomised, controlled trial. *Psychopharmacology* 2020; **237**(1): 209-18.

175. Vellekkatt F, Menon V, Rajappa M, Sahoo J. Effect of adjunctive single dose parenteral Vitamin D supplementation in major depressive disorder with concurrent vitamin D deficiency: A double-blind randomized placebo-controlled trial. *Journal of psychiatric research* 2020; **129**: 250-6.

176. Yosaee S, Soltani S, Esteghamati A, et al. Effects of zinc, vitamin D, and their co-supplementation on mood, serum cortisol, and brain-derived neurotrophic factor in patients with obesity and mild to moderate depressive symptoms: A phase II, 12-wk, 2 × 2 factorial design, double-blind, randomized, placebo-controlled trial. *Nutrition (Burbank, Los Angeles County, Calif)* 2020; **71**: 110601.

177. Zhu C, Zhang Y, Wang T, et al. Vitamin D supplementation improves anxiety but not depression symptoms in patients with vitamin D deficiency. *Brain Behav* 2020; **10**(11): e01760.

178. Kumar PNS, Menon V, Andrade C. A randomized, double-blind, placebo-controlled, 12-week trial of vitamin D augmentation in major depressive disorder associated with vitamin D deficiency. *J Affect Disord* 2022; **314**: 143-9.

179. Mischoulon D, Dunlop BW, Kinkead B, et al. Omega-3 Fatty Acids for Major Depressive Disorder With High Inflammation: A Randomized Dose-Finding Clinical Trial. *The Journal of clinical psychiatry* 2022; **83**(5).

180. Schaub AC, Schneider E, Vazquez-Castellanos JF, et al. Clinical, gut microbial and neural effects of a probiotic add-on therapy in depressed patients: a randomized controlled trial.

Translational psychiatry 2022; **12**(1): 227.
181. Tarutani S, Omori M, Ido Y, Yano M, Komatsu T, Okamura T. Effects of 4G-beta-D-Galactosylsucrose in patients with depression: A randomized, double-blinded, placebo-controlled, parallel-group comparative study. *J Psychiatr Res* 2022; **148**: 110-20.
182. Tian P, Chen Y, Zhu H, et al. Bifidobacterium breve CCFM1025 attenuates major depression disorder via regulating gut microbiome and tryptophan metabolism: A randomized clinical trial. *Brain, behavior, and immunity* 2022; **100**: 233-41.

183. Wu SK, Yang KJ, Liu WC, et al. The Efficacy of Omega-3 Fatty Acids as the Monotherapy for Depression: A Randomized, Double-Blind, Placebo-Controlled Pilot Study. *Nutrients* 2024; **16**(21).
184. Torkaman P, Meybodi AM, Kheradmand A, Eiliaei S, Ardakani MT. Effect of l-arginine compared to placebo on sexual function in women with major depressive disorder: a randomized controlled trial. *BMC Psychiatry* 2024; **24**(1).
185. Strodl E, Bambling M, Parnam S, Ritchie G, Cramb S, Vitetta L. Probiotics and magnesium orotate for the treatment of major depressive disorder: a randomised double blind controlled trial. *Sci Rep* 2024; **14**(1): 20841.
186. Lin SK, Kuo PH, Hsu CY, Chiu YH, Chen CH. The effects of Lactobacillus plantarum PS128 in patients with major depressive disorder: an eight-week double-blind, placebo-controlled study. *Asian J Psychiatr* 2024; **101**: 104210.
187. Khademi F, Tutunchi H, Vaghef-Mehrabani E, Ebrahimi-Mameghani M. The effect of prebiotic supplementation on serum levels of tryptophan and kynurenine in obese women with major depressive disorder: a double-blinded placebo-controlled randomized clinical trial. *BMC Res Notes* 2024; **17**(1): 316.
188. Hashemi-Mohammadabad N, Taghavi SA, Lambert N, Moshtaghi R, Bazarganipour F, Sharifi M. Adjuvant administration of probiotic effects on sexual function in depressant women undergoing SSRIs treatment: a double-blinded randomized controlled trial. *BMC Psychiatry* 2024; **24**(1): 44.
189. Gawlik-Kotelnicka O, Margulska A, Płeska K, Skowrońska A, Strzelecki D. Metabolic Status Influences Probiotic Efficacy for Depression-PRO-DEMET Randomized Clinical Trial Results. *Nutrients* 2024; **16**(9).
190. Shamabadi A, Kafi F, Arab Bafrani M, Asadigandomani H, A. Basti F, Akhondzadeh S. l-theanine adjunct to sertraline for major depressive disorder: A randomized, double-blind, placebo-controlled clinical trial. *Journal of Affective Disorders* 2023; **333**: 38-43.
191. Kolahdooz G, Vosough I, Sepahi S, Mohajeri SA. The effect of crocin versus sertraline in treatment of mild to moderate postpartum depression: a double-blind, randomized clinical trial. *Int Clin Psychopharmacol* 2023; **38**(1): 9-15.
192. Yang R, Wang L, Jin K, et al. Omega-3 Polyunsaturated Fatty Acids Supplementation

Alleviate Anxiety Rather Than Depressive Symptoms Among First-Diagnosed, Drug-Naïve Major Depressive Disorder Patients: A Randomized Clinical Trial. *Front Nutr* 2022; **9**: 876152.

5. *Characteristics of included studies*

Study ID	Author	Year	Nutraceuticals	Type of antidepressants	Country	N	Age	Female	%	Scale	Study Duration	Baseline measure	Severity	Sponsor Industry	Comorbidity
1	Ayuso	1971	Tryptophan+ADT	Not mention	Spain	14		13	92.86	HAMD	3	30.6	severe	unclear	unclear
1	Ayuso	1971	ADT	Not mention		15		12	80.00	HAMD	3	30.6			
2	HoesSijben	1981	Vitamin_B6+Tryptopohan		The Netherland	10	37.3	8	80.00	HAMD	4	26.4	severe	Y	N
2	HoesSijben	1981	ADT	Maprotiline		10	45.3	3	30.00	HAMD	4	28.2			
3	Coppen_1986	1986	Folate+ADT	Fluoxetine	U.K.	23	60.7		0.00	BDI	52	7.4	mild	unclear	unclear
3	Coppen_1986	1986	ADT	Fluoxetine		30	60.5		0.00	BDI	52	8.7			
4	Thomas	1987	SAMe		U.K.	9	56.9	7	77.78	HAMD	2	26.6	severe	unclear	unclear
4	Thomas	1987	Placebo			11	55.6	7	63.64	HAMD	2	25.2			
5	Bell_1988	1988	SAMe		USA	9	43		0.00	HAMD-21	2	40	severe	unclear	unclear
5	Bell_1988	1988	ADT	Imipramine		9	43		0.00	HAMD-21	2	39			
6	Potkin	1988	SAMe		USA	9			0.00	HAMD	2		unclear	unclear	unclear
6	Potkin	1988	ADT	Imipramine		9			0.00	HAMD	2				
7	Kagan	1990	SAMe		USA	9	42.2		0.00	HAMD-21	3	26.6	moderate	unclear	N
7	Kagan	1990	Placebo			9	42.2		0.00	HAMD-21	3	31			
8	Godfrey	1990	Folate+ADT	TCAs or MAOIs	U.K.	13	45	7	53.85	HAMD-17	12		unclear	N	N
8	Godfrey	1990	ADT	TCAs or MAOIs		11	46	6	54.55	HAMD-17	12				
9	Gecele	1991	Carnitine		Italy	14	71.3		0.00	HAMD	6	38.2	severe	unclear	N
9	Gecele	1991	Placebo			14	71.3		0.00	HAMD	6	36			
10	Berlanga	1992	SAMe+ADT	Imipramine	Mexico	20	43.8	15	75.00	HAMD-17	2	25.4	severe	unclear	N
10	Berlanga	1992	ADT	Imipramine		20	37.9	17	85.00	HAMD-17	2	25.3			
11	Fava_1992	1992	SAMe		USA	17	33.4			HAMD-24	6	27.2	moderate	unclear	N
11	Fava_1992	1992	Placebo			22	34.1		0.00	HAMD-24	6	24.6			

12	De VannaRigamont	1992	SAMe		Italy	15	48.4		0.00	HAMD-21	7	34	severe	Y	N
12	De VannaRigamont	1992	ADT	Imipramine		15	48.5		0.00	HAMD-21	7	31			
13	Salmaggi	1993	SAMe		Italy	30	range	30	100.00	HAMD-21	4	24.4	severe	Y	N
13	Salmaggi	1993	Placebo			30	range	30	100.00	HAMD-21	4	25.2			
14	Bell_1994	1994	SAMe		USA	11	43	7	63.64	HAMD-17	4	23	severe	unclear	unclear
14	Bell_1994	1994	ADT	Desipramine		6	33	6	100.00	HAMD-17	4	28			
15	Hubner	1994	SJW		Germany	20	50.4	12	60.00	HAMD	4	12.55	mile	Unclear	unclear
15	Hubner	1994	Placebo			19	51.5	10	52.63	HAMD	4	12.37			
16	Hansgen	1994	SJW		Germany	33	53	19	57.58	HAMD	4	21.8	moderate	N	unclear
16	Hansgen	1994	Placebo			34	53.5	23	67.65	HAMD	4	20.4			
17	Harrer	1994	SJW		Germany & Australia	51	43.8		0.00	HAMD	4	20.5	moderate	Unclear	unclear
17	Harrer	1994	ADT	Maprotiline		51	47.6		0.00	HAMD	4	21.5			
18	SommerHarrer	1994	SJW		Germany & Australia	42	45		0.00	HAMD	4	15.8	Mild	Unclear	unclear
18	SommerHarrer	1994	Placebo			47	45		0.00	HAMD	4	15.8			
19	Vorbach_1994	1994	SJW		Germany	67	52.8	33	49.25	HAMD	6	20.2	moderate	Unclear	N
19	Vorbach_1994	1994	ADT	Imipramine		68	54	31	45.59	HAMD	6	19.4			
20	Wheatley	1997	SJW		U.K.	67	42	57	85.07	HAMD	6	20.6	moderate	Unclear	N
20	Wheatley	1997	ADT	Amitriptyline		54	38	41	75.93	HAMD	6	20.8			
21	Vorbach	1997	SJW		Germany	107	48.8	78	72.90	HAMD-17	6	25.3	severe	Unclear	N
21	Vorbach	1997	ADT	Imipramine		102	50.1	76	74.51	HAMD-17	6	26.1			
22	Schrader	1998	SJW		Germany	80	47		0.00	HAMD-21	6	20	mild	Unclear	N
22	Schrader	1998	Placebo			79	39		0.00	HAMD-21	6	17			

23	Laakmann	1998	SJW		Germany	98	47.3	40	40.82	HAMD	6	20.6	moderate	Unclear	N
23	Laakmann	1998	Placebo			49	48.7	42	85.71	HAMD	6	21.2			
24	Nemets_1999	1999	Inositol+ADT	SSRIs	Israel	18	49.5	11	61.11	HAMD-24	4	30	moderate	Unclear	N
24	Nemets_1999	1999	ADT	SSRIs		18	51.5	11	61.11	HAMD-24	4	28.4			
25	Levine	1999	Inositol+ADT	SSRIs	Israel	13	45.9	10	76.92	HAMD	4	34.4	moderate	N	N
25	Levine	1999	ADT	SSRIs		14	49.6	9	64.29	HAMD	4	31			
26	Harrer _1999	1999	SJW		Germany & Australia	70	68.4	60	85.71	HAM-D-17	6	16.6	Mild	N	N
26	Harrer _1999	1999	ADT	Fluoxetine		79	69.1	69	87.34	HAM-D-17	6	17.18			
27	Philipp	1999	SJW		Germany	106	47	80	75.47	HAMD-17	8	22.7	moderate	Y	N
27	Philipp	1999	ADT	Imipramine		110	48	79	71.82	HAMD-17	8	22.2			
27	Philipp	1999	Placebo			47	43	38	80.85	HAMD-17	8	22.7			
28	Levitan	2000	Tryptophan+ADT	Fluoxetine	Canada	13	41.8		0.00	HAMD-29	8	27.2	moderate	N	N
28	Levitan	2000	ADT	Fluoxetine		17	45.5		0.00	HAMD-29	8	24			
29	CoppenBailey	2000	Folate+ADT	Fluoxetine	U.K.	62	41.9		0.00	HAMD	10	26.8	severe	unclear	N
29	CoppenBailey	2000	ADT	Fluoxetine		65	44.3	33	50.77	HAMD	10	26.6			
30	Schrader	2000	SJW		Germany	125	47	90	72.00	HAMD	6	19.6	moderate	Unclear	N
30	Schrader	2000	ADT	Fluoxetine		113	46	67	59.29	HAMD	6	19.5			
31	Brenner	2000	SJW		USA	15	44.2	10	66.67	HAMD-17	6	22.4	moderate	N	N
31	Brenner	2000	ADT	Sertraline		15	46.9	9	60.00	HAMD-17	6	22.1			
32	Woelk	2000	SJW		Germany	157	46.5		0.00	HAMD-17	6	22.4	moderate	N	N
32	Woelk	2000	ADT	Imipramine		167	45.4		0.00	HAMD-17	6	22.1			
33	Shelton	2001	SJW		USA	98	41.4	64	65.31	BDI	8	22	mild	Y	N
33	Shelton	2001	Placebo			102	43.3	64	62.75	BDI	8	24.2			
34	Kalb	2001	SJW		Germany	37	48	26	70.27	HAMD-17	6	19.7	moderate	Y	N

34	Kalb	2001	Placebo			35	49	22	62.86	HAMD-17	6	20.1			
35	Delle Chiaie	2002	SAMe		Italy	143	45.3	103	72.03	HAMD-17	6	25.1	severe	Y	unclear
35	Delle Chiaie	2002	ADT	Imipramine		135	44.6	93	68.89	HAMD-17	6	25.5			
36	Pancheri (Delle Chiaie 2)	2002	SAMe		Italy	146	48.2	102	69.86	HAMD-17	4	24.3	severe	Y	unclear
36	Pancheri (Delle Chiaie 2)	2002	ADT	Imipramine		147	48..8	83	56.46	HAMD-17	4	26			
37	PeetHorrobin	2002	EPA+DHA+ADT	Not mention	U.K.	52	54.2	57	109.62	HAMD-17	12		unclear	N	unclear
37	PeetHorrobin	2002	ADT	Not mention		18	52.1	57	316.67	HAMD-17	12				
38	Lecrubier	2002	SJW		France	186	40.2	142	76.34	HAMD-17	6	21.9	moderate	Y	N
38	Lecrubier	2002	Placebo			189	41.2	145	76.72	HAMD-17	6	21.9			
39	SJW group	2002	SJW		USA	113	43.1	73	64.60	HAMD-17	8	23.1	moderate	N	N
39	SJW group	2002	ADT	Sertraline		111	43.9	74	66.67	HAMD-17	8	22.6			
39	SJW group	2002	Placebo			116	40.2	77	66.38	HAMD-17	8	22.7			
40	Behnke	2002	SJW		Denmark	35	51.4	24	68.57	HAMD-17	6	20	moderate	unclear	N
40	Behnke	2002	ADT	Fluoxetine		35	48	23	65.71	HAMD-17	6	20.7			
41	van Gurp	2002	SJW		Canada	44	40.9	28	63.64	HAMD-17	12	18.9	moderate	N	N
41	van Gurp	2002	ADT	Sertraline		43	39.1	24	55.81	HAMD-17	12	19.7			
42	Nemets_2002	2002	EPA+ADT	Not mention	Israel	10	54.2	9	90.00	HAMD-24	4	24	moderate	unclear	N
42	Nemets_2002	2002	ADT	Not mention		10	52.1	8	80.00	HAMD-24	4	22			
43	Zanarini	2003	EPA		USA	20	26.3	20	100.00	MADRS	8	17.7	mild	N	Y
43	Zanarini	2003	Placebo			10	26.3	10	100.00	MADRS	8	18			
44	Su_2003	2003	EPA+DHA+ADT	Not mention	Taiwan	12	35.2	10	83.33	HAMD-21	8	22.5	moderate	Y	N
44	Su_2003	2003	ADT	Not mention		10	42.3	8	80.00	HAMD-21	8	22.1			
45	Marangell	2003	DHA		USA	18	46.8	14	77.78	HAMD-17	6	23.5	severe	Y	N
45	Marangell	2003	Placebo			17	47.9	14	82.35	HAMD-17	6	28.5			

46	Nowak	2003	Zinc+ADT	Clomipramine , Amitriptyline, Citalopram, Fluoxetine	Poland	6	42.2	2	33.33	HAMD-17	12	24.5	moderate	N	unclear
46	Nowak	2003	ADT	Clomipramine , Amitriptyline, Citalopram, Fluoxetine		8	43.4	6	75.00	HAMD-17	12	22.6			
47	Akhondzadeh	2003	Lavandula		Iran	15	33.53	7	46.67	HAMD-17	4	19.6	moderate	N	N
47	Akhondzadeh	2003	ADT	Imipramine		15	33.93	8	53.33	HAMD-17	4	19			
47	Akhondzadeh	2003	Lavandula+ADT	Imipramine		15	31.53	6	40.00	HAMD-17	4	19			
48	Akhondzadeh_2004	2004	Saffron		Iran	15	35.53	9	60.00	HAMD-17	6	19.2	moderate	N	N
48	Akhondzadeh_2004	2004	ADT	Imipramine		15	32.53	8	53.33	HAMD-17	6	19			
49	Uebelhack	2004	SJW		Germany	70	46.4	49	70.00	HAMD-17	6	22.8	moderate	unclear	N
49	Uebelhack	2004	Placebo			70	43.3	45	64.29	HAMD-17	6	22.6			
50	Silvers	2005	DHA+ADT	Not mention	New Zealand	40	39.8	22	55.00	HAMD-SF	12	11.5	moderate	N	N
50	Silvers	2005	ADT	Not mention		37	37.7	19	51.35	HAMD-SF	12	12.4			
51	Docherty	2005	Chromium		USA	70	46.4	54	77.14	HAMD-29	8				
51	Docherty	2005	Placebo			40	45.5	24	60.00	HAMD-29	8				
52	Akhondzadeh_2005	2005	Saffron		Iran	20	37.3	9	45.00	HAMD-17	6	>18	mild	N	unclear
52	Akhondzadeh_2005	2005	Placebo			20	35.25	9	45.00	HAMD-17	6	>18			
53	Noorbala	2005	Saffron		Iran	20	37.3	9	45.00	HAMD-17	6	>18	moderate	N	N
53	Noorbala	2005	ADT	Fluoxetine		20	36.5	11	55.00	HAMD-17	6	>18			
54	Bjerkenstedt	2005	SJW		Sweden	54	49.1	43	79.63	HAMD-17	4	24.9	severe	Y	N

54	Bjerkenstedt	2005	ADT	Fluoxetine		54	50.4	41	75.93	HAMD-17	4	23.8			
54	Bjerkenstedt	2005	Placebo			55	51.4	45	81.82	HAMD-17	4	25.2			
55	Szegedi	2005	SJW		Germany	122	49	85	69.67	HAMD-17	6	25.5	severe	Y	N
55	Szegedi	2005	ADT	Paroxetine		122	45.5	83	68.03	HAMD-17	6	25.5			
56	Gastpar_2005	2005	SJW		Germany	106	48.3	84	79.25	HAMD-17	24	22	moderate	unclear	N
56	Gastpar_2005	2005	ADT	Sertraline		77	49.5	53	68.83	HAMD-17	24	22.1			
57	Fava_2005	2005	SJW		USA	45	37.4	24	53.33	HAMD-17	8	19.6	moderate	Y	N
57	Fava_2005	2005	ADT	Fluoxetine		47	36.7	25	53.19	HAMD-17	8	19.6			
57	Fava_2005	2005	Placebo			43	37.8	28	65.12	HAMD-17	8	19.9			
58	Moshiri	2006	Saffron		Iran	20	35.45	9	45.00	HAMD-17	6	>18	moderate	N	N
58	Moshiri	2006	Placebo			20	35.85	8	40.00	HAMD-17	6	>18			
59	Gastpar_2006	2006	SJW		Germany	131	50.8	86	65.65	HAMD-17	6	21.9	moderate	unclear	N
59	Gastpar_2006	2006	ADT	Citalopram		127	49.3	82	64.57	HAMD-17	6	21.8			
59	Gastpar_2006	2006	Placebo			130	49.4	95	73.08	HAMD-17	6	22			
60	Kasper_2006	2006	SJW		Germany	243	46.19	67	56.30	HAMD-17	6		moderate	Y	unclear
60	Kasper_2006	2006	Placebo			81	46.9	82	66.10	HAMD-17	6				
61	Moreno	2006	SJW		Brazil	20	37.2	17	85.00	HAMD-21	8	15	mild	Y	N
61	Moreno	2006	ADT	Fluoxetine		20	37.7	17	85.00	HAMD-21	8	15.8			
61	Moreno	2006	Placebo			26	45.9	22	84.62	HAMD-21	8	16.4			
62	Randlov	2006	SJW		Denmark	87	50.9		0.00	HAMD	6	7 月 17 日	mild	Y	N
62	Randlov	2006	Placebo			42	50.9		0.00	HAMD	6	7 月 17 日			
63	Sayyah	2006	E_amoenum		Iran	19	29.5	7	36.84	HAMD-17	6	27	severe	N	N
63	Sayyah	2006	Placebo			16	34.7	7	43.75	HAMD-17	6	27			
64	Grenyer	2007	EPA+DHA+ADT	Not mention	Australia	40	45.27		0.00	BDI	16	25	moderate	N	N

64	Grenyer	2007	ADT	Not mention		43	45.27		0.00	BDI	16	27.8		
65	Akhondzadeh_Bast	2007	Saffron		Iran	20	35.55	10	50.00	HAMD-17	8	18-25	moderate	N	N
65	Akhondzadeh_Bast	2007	ADT	Fluoxetine		20	34.09	11	55.00	HAMD-17	8	18-25			
66	Ille	2007	Amino_acid+ADT	Mirtazapine	Australia	20	48.9	17	85.00	HAMD	4	26.7	moderate	N	unclear
66	Ille	2007	ADT	Mirtazapine		20	43.8	15	75.00	HAMD	4	21.7			
67	Darbinyan	2007	R_rosea		Armenia	31	44.9	21	67.74	HAMD-21	6	24.16	moderate	Y	N
67	Darbinyan	2007	Placebo			29	44.6	17	58.62	HAMD-21	6	24.17			
68	Resler	2008	Folate+ADT	Fluoxetine	Venezuela	14	35.04	36	257.14	HAMD-17	6	22.5	moderate	unclear	N
68	Resler	2008	ADT	Fluoxetine		13	35.04		0.00	HAMD-17	6	21.85			
69	Jazayeri	2008	EPA+ADT	Fluoxetine	Iran	16	34.5	9	56.25	HAMD-24	8	>15	moderate	N	N
69	Jazayeri	2008	ADT	Fluoxetine		16	35.1	12	75.00	HAMD-24	8	>15			
69	Jazayeri	2008	EPA			16	34.9	12	75.00	HAMD-24	8	>15			
70	Rogers	2008	EPA+DHA		U.K.	109	38	85	77.98	BDI	12	13.9	mild	N	N
70	Rogers	2008	Placebo			109	38.2	83	76.15	BDI	12	13.9			
71	Rees	2008	EPA+DHA		Australia	13	31.2	13	100.00	MADRS	6	30.2	moderate	unclear	N
71	Rees	2008	Placebo			13	34.5	13	100.00	MADRS	6	29.2			
72	Su_2008	2008	EPA+DHA		Taiwan	18	range	18	100.00	HAMD-21	8	22.3	moderate	N	N
72	Su_2008	2008	Placebo			18	range	18	100.00	HAMD-21	8	22.3			
73	da_Silva	2008	EPA+DHA		Brazil	6	64.4		0.00	MADRS	12	23.5	moderate	N	Y
73	da_Silva	2008	EPA+DHA+ADT	Not mention		8	64.4		0.00	MADRS	12	23			
73	da_Silva	2008	ADT	Not mention		9	64.4		0.00	MADRS	12	24			
73	da_Silva	2008	Placebo			7	64.4		0.00	MADRS	12	25.5			
74	Freeman	2008	EPA+DHA		USA	28	31	28	100.00	HAMD	8	18.86	mild	N	N
74	Freeman	2008	Placebo			23	29.7	23	100.00	HAMD	8	17.43			

75	Barragan	2008	Mg		Mexico	12	69		0.00	Yasavage	12	17.9	moderate	N	Y
75	Barragan	2008	ADT	Imipramine		11	66.4		0.00	Yasavage	12	11.4			
76	Raza-ur-Rahman	2008	SJW		Pakistan	56	33.89	13	23.21	HAMD-17	6	18.04	mild	Y	N
76	Raza-ur-Rahman	2008	Placebo			56	36.29	12	21.43	HAMD-17	6	17.5			
77	Başoğlu	2009	Folate+ADT	Escitalopram	Turkey	20	29	11	55.00	MADRS	6	31.5	severe	unclear	N
77	Başoğlu	2009	ADT	Escitalopram		15	30	8	53.33	MADRS	6	32			
78	Mischoulon_2009	2009	EPA		USA	11	42		0.00	HAMD-17	8	21.6	moderate	N	N
78	Mischoulon_2009	2009	Placebo			13	42		0.00	HAMD-17	8	20.5			
79	Carney	2009	EPA+DHA+ADT	Sertraline	USA	62	58.1	22	35.48	HAMD-21	10	21.2	moderate	N	Y
79	Carney	2009	ADT	Sertraline		60	58.6	19	31.67	HAMD-21	10	19.2			
80	Lucas	2009	EPA		Canada	55	48.4		0.00	HAMD-21	8	17.1	mild	N	N
80	Lucas	2009	Placebo			51	49.1		0.00	HAMD-21	8	16.3			
81	Sarris_2009	2009	SJW+Kava		Australia	13	42.9	27		BDI-II	8	23.3	mild	Y	N
81	Sarris_2009	2009	Placebo			15	42.9	24		BDI-II	8	22.6			
82	Siwek_2009	2009	Zinc+ADT	Imipramine	Poland	27.00	46.20	19.00	70.37	HAMD	12	22.9	moderate	N	unclear
82	Siwek_2009	2009	ADT	Imipramine		25.00	45.70	21.00	84.00	HAMD	12	22.9			
83	Papakosta_2010	2010	SAMe+ADT	SSRIs, SNRIs	USA	39		21	53.85	HAMD-17	6	19.1	moderate	N	N
83	Papakosta_2010	2010	ADT	SSRIs, SNRIs		34		23	67.65	HAMD-17	6	19.9			
84	Bot	2010	EPA+ADT	Not mention	The Netherland	13	53.1	8	61.54	MADRS	12	26.3	moderate	Y	N
84	Bot	2010	ADT	Not mention		12	55	5	41.67	MADRS	12	26.4			
85	Rondanelli	2010	EPA+DHA		Italy	22	84.9		0.00	GDS-30	8	17.1	moderate	N	N
85	Rondanelli	2010	Placebo			24	83.07		0.00	GDS-30	8	16.7			
86	Mannel	2010	SJW		Germany	100	47	81	81.00	HAMD-17	8	12.4	mild	Y	N

86	Mannel	2010	Placebo			100	46.6	85	85.00	HAMD-17	8	12			
87	Liu	2010	SJW		China	44	67	23	52.27	HAMD-17	12	24.72	severe	N	Y
87	Liu	2010	Placebo			40	67	20	50.00	HAMD-17	12	24.42			
88	Rapaport_2011	2011	SJW		USA	26	42.2	13	50.00	HAMD-17	12	13.6	mild	N	unclear
88	Rapaport_2011	2011	ADT	Citalopram		24	51.3	13	54.17	HAMD-17	12	12.5			
88	Rapaport_2011	2011	Placebo			23	51.4	11	47.83	HAMD-17	12	13.3			
89	Lesperance_2011	2011	EPA+DHA+ADT	Not mention	Canada	218	46.6	143	65.60	MADRS	8	27.96	mild	Y	N
89	Lesperance_2011	2011	ADT	Not mention		214	45.4	153	71.50	MADRS	8	28.64			
90	Gertsik	2012	EPA+DHA+ADT	Citalopram	USA	18	40.5		0.00	HAMD-21	8	25.3	moderate	N	N
90	Gertsik	2012	ADT	Citalopram		22	40.5		0.00	HAMD-21	8	25.3			
91	Rizzo	2012	EPA+DHA		Italy	22	46.8	22	100.00	GDS-30	6	47	mild	N	N
91	Rizzo	2012	Placebo			24	83	24	100.00	GDS-30	6	48			
92	Lyoo	2012	Creatine+ADT	SSRIs	South Korea	17	45.7	17	100.00	HAMD-17	8	17.1	moderate	N	N
92	Lyoo	2012	ADT	SSRIs		22	47.5	22	100.00	HAMD-17	8	16.7			
93	Sarris_2012	2012	SJW		Australia	35	45	22	62.86	HAMD-17	8	26.9	severe	N	N
93	Sarris_2012	2012	ADT	Sertraline		49	46	31	63.27	HAMD-17	8	26.7			
93	Sarris_2012	2012	Placebo			40	42	26	65.00	HAMD-17	8	22.8	moderate	N	unclear
94	Papakostas_2012	2012	Folate+ADT	SSRIs	USA	36	47.9	25	69.44	HAMD-17	8	22.8			
94	Papakostas_2012	2012	ADT	SSRIs		112	47.9	78	69.64	HAMD-17	8	NR			
94	Papakostas_2012_2	2012	Folate+ADT	SSRIs	USA	19	48.4	13	68.42	HAMD-17	4	18.8	moderate	Y	N
94	Papakostas_2012_2	2012	ADT	SSRIs		56	48.4	39	69.64	HAMD-17	4	19.9			
95	Pakseresht	2012	SJW+ADT	TCAs	Iran	20	29.8	10	50.00	BDI	4	21.2			

95	Pakseresht	2012	ADT	TCAs		20	30	11	55.00	BDI	4	21.2		
96	Syed	2013	Vitamin_B12+ADT	Imipramine, SSRIs, Fluoxetine	Pakistan	34	37.68	16	47.06	HAMD-20	6	36	moderate	N	N
96	Syed	2013	ADT	Imipramine, SSRIs, Fluoxetine		39	36.56	19	48.72	HAMD-20	6	35			
97	Lewis	2013	Vitamin_B+ADT	Not mention	USA	30	49.3	11	36.67	BDI-II	6	23.21	moderate	N	N
97	Lewis	2013	ADT	Not mention		30	52.5	8	26.67	BDI-II	6	19.38			
98	Bersani	2013	Carnitine		Italy	41	72.23	32	78.05	HAMD-21	8	17.7	mild	N	unclear
98	Bersani	2013	ADT	Fluoxetine		39	71.42	37	94.87	HAMD-21	8	22.2			
99	Safa	2013	EPA+DHA+ADT	Fluvoxamine	Iran	40	37.3	21	52.50	HAM-D-21	24	24.72	moderate	N	N
99	Safa	2013	ADT	Fluvoxamine		40	40.27	22	55.00	HAM-D-21	24	24.72			
100	Mozurkewich	2013	EPA+ADT	Not mention	USA	39	29.9	39	100.00	BDI	12		severe	N	N
100	Mozurkewich	2013	DHA+ADT	Not mention		38	30.6	38	100.00	BDI	12				
100	Mozurkewich	2013	ADT	Not mention		41	30.4	41	100.00	BDI	8	34.24	mild	N	N
101	Khajehnasiri	2013	EPA+DHA+Vitamin_C		Iran	34	29.47		0.00	BDI-21	8	35.82			
101	Khajehnasiri	2013	EPA+DHA			34	31.71		0.00	BDI-21	7	14.24	mild	N	N
101	Khajehnasiri	2013	Vitamin_C			34	30.71		0.00	BDI-21	7	13.9			
102	Khajehnasiri	2013	Placebo			34	31.12		0.00	BDI-21	7	14.13			
102	Khoraminya	2013	Vitamin_D+ADT	Fluoxetine	Iran	20	38.1	3	15.00	HAMD-17	7	14.9			
102	Khoraminya	2013	ADT	Fluoxetine		20	39.65	3	15.00	HAMD-17	8	29.4	severe	N	N
103	Mozaffari-Khosravi_2013	2013	Vitamin_D		Iran	75	32.88	52	69.33	BDI-II	8	30.2			

103	Mozaffari-Khosravi_2013	2013	Placebo			34	33	26	76.47	BDI-II	12	27.01	mild	N	N
104	Bergman	2013	Curcumin+ADT	Escitalopram, Venlafaxine	Israel	20	65.8	9	45.00	HAMD	12	26.4			
104	Bergman	2013	ADT	Escitalopram, Venlafaxine		19	61.3	14	73.68	HAMD	5	32.7	severe	N	N
105	Nemets_2013	2013	Creatine+ADT	SSRIs, SNRIs, NASA	Israel	9	60.8	9	100.00	HAMD-24	5	32.7			
105	Nemets_2013	2013	ADT	SSRIs, SNRIs, NASA		9	50	5	55.56	HAMD-24	4	25.2	moderate	N	N
106	Mozaffari-Khosravi	2013	EPA+ADT	Not mention	Iran	21	37.5		0.00	HAMD-17	4	28.2			
106	Mozaffari-Khosravi	2013	DHA+ADT	Not mention		20	34		0.00	HAMD-17	12	15.9	mild	N	N
106	Mozaffari-Khosravi	2013	ADT	Not mention		21	33.8		0.00	HAMD-17	12	15.7			
107	Mischoulon_2014	2014	SAMe		USA	64	45		0.00	HAMD-17	12	15.5			
107	Mischoulon_2014	2014	ADT	Escitalopram		65	45		0.00	HAMD-17	12	18.98	moderate	N	N
107	Mischoulon_2014	2014	Placebo			60	45		0.00	HAMD-17	12	19.25			
108	Sarris _2014	2014	SAMe		Australia	18	47.67		0.00	HAMD-17	12	19.43			
108	Sarris _2014	2014	ADT	Escitalopram		20	47.67		0.00	HAMD-17	12	19.9	moderate	N	N
108	Sarris _2014	2014	Placebo			16	47.67		0.00	HAMD-17	12	20.83			
109	Bedson	2014	Folate+ADT	SSRIs ,TCAs	U.K.	223	45	144	64.57	MADRS	12	20.63			
109	Bedson	2014	ADT	SSRIs ,TCAs		217	45	136	62.67	MADRS	12	28.3	moderate	N	N

110	Almeida	2014	Folate+Vitamin_B12+ Vitamin_B6		Australia	77		38	49.35	MADRS	12	29		
110	Almeida	2014	Placebo			76		48	63.16	MADRS	8	27	moderate	N	N
111	Dashti-Khavidaki	2014	EPA+DHA		Iran	18	56.1	9	50.00	BDI	8	26			
111	Dashti-Khavidaki	2014	Placebo			16	56.5	8	50.00	BDI	16	25	mild	N	Y
112	Kaviani	2014	EPA+DHA		Iran	40	26.33		0.00	BDI	16	20			
112	Kaviani	2014	Placebo			40	25.15		0.00	BDI	6	16.52	mild	N	N
113	Sanmukhani	2014	Curcumin+ADT	Fluoxetine	India	18	40.4	13	72.22	HAMD-17	6	17.47			
113	Sanmukhani	2014	ADT	Fluoxetine		17	33.6	9	52.94	HAMD-17	6	21.9	moderate	N	N
113	Sanmukhani	2014	Curcumin			16	37.8	12	75.00	HAMD-17	6	21			
114	Shahmansouri	2014	Saffron		Iran	20	52.05	11	55.00	HAMD-17	6	19.3			
114	Shahmansouri	2014	ADT	Fluoxetine		20	53.1	14	70.00	HAMD-17	6	17	mild	N	N
115	Lopresti_2014	2014	Curcumin+ADT	Not mention	Australia	28	44.04	20	71.43	IDS-SR3	6	16.8			
115	Lopresti_2014	2014	ADT	Not mention		28	48.54	20	71.43	IDS-SR3	8	33.04	mild	Y	N
116	Park	2015	EPA+DHA+ADT	Not mention	South Korea	12	43.5	14	116.67	CESD	8	33.14			
116	Park	2015	ADT	Not mention		13	39.41	13	100.00	CESD	12	38.5	severe	N	N
117	Ginty	2015	EPA+DHA		USA	12	20.33	8	66.67	BDI	12	34.13			
117	Ginty	2015	Placebo			9	20	9	100.00	BDI	3	15.58	mild	N	unclear
118	Mischoulon_2015	2015	EPA		USA	60	46.2	38	63.33	HAMD-17	3	15.89			
118	Mischoulon_2015	2015	DHA			58	46.3	32	55.17	HAMD-17	8	19.3	moderate	N	N
118	Mischoulon_2015	2015	Placebo			59	45	35	59.32	HAMD-17	8	19.8			

119	Sahraian	2015	Vitamin_C+ADT	Citalopram	Iran	21	32.7	15	71.43	HAMD-21	8	19.2			
119	Sahraian	2015	ADT	Citalopram		22	34.2	17	77.27	HAMD-21	8	21.4	moderate	N	N
120	Panahi	2015	Curcumin+ADT	Not mention	Iran	61	40.69	31	50.82	BDI-II	8	22.4			
120	Panahi	2015	ADT	Not mention		50	40.4	29	58.00	BDI-II	6	38.66	moderate	unclear	N
121	Yu	2015	Curcumin+ADT	Escitalopram	China	50	44.14		0.00	HAMD-17	6	40.44			
121	Yu	2015	ADT	Escitalopram		50	45.22		0.00	HAMD-17	6	14.06	mild	N	N
122	Talaei	2015	Saffron+ADT	SSRIs(Fluoxetine, Sertraline,Citalopram)	Iran	20	35.9	18	90.00	BDI	6	14.28			
122	Talaei	2015	ADT	SSRIs(Fluoxetine, Sertraline, Citalopram)		20	36.5	16	80.00	BDI	4	35-40	severe	N	N
123	Panahi	2015	Chlorella+ADT	Not mention	Iran	42	40.79	24	57.14	BDI-II	4	35-40			
123	Panahi	2015	ADT	Not mention		50	40.4	29	58.00	BDI-II	6	40.4	moderate	unclear	N
124	Gavrilova	2015	Carnitine+ADT	Not mention	Russia	20	69.2	16	80.00	HAMD-17	6	40.44			
124	Gavrilova	2015	ADT	Not mention		20	71.5	16	80.00	HAMD-17	8	23.1	moderate	unclear	unclear
125	Mao	2015	R_rosea		USA	20	46.9	8	40.00	HAMD-17	8	22.55			
125	Mao	2015	ADT	Sertraline		19	41.4	10	52.63	HAMD-17	12	14.4	mild	N	N
125	Mao	2015	Placebo			18	46.7	8	44.44	HAMD-17	12	15.4			
126	Ghaleiha	2016	Vitamin_B1+ADT	Fluoxetine	Iran	25	35.28	13	52.00	HAMD-21	12	14.4			
126	Ghaleiha	2016	ADT	Fluoxetine		26	35.08	14	53.85	HAMD-21	12	31.4	severe	N	N
127	Masoumi	2016	EPA+DHA+ADT	Citalopram	Iran	30	55.17	30	100.00	BDI	12	32.77			
127	Masoumi	2016	ADT	Citalopram		30	55.67	30	100.00	BDI	4	25.89	mild	N	N
128	Shinto	2016	EPA+DHA+ADT	Not mention	USA	15	50.7	19	126.67	MADRS	4	26.35			
128	Shinto	2016	ADT	Not mention		16	51.9	17	106.25	MADRS	12	18.4	mild	N	Y
129	Rapaport_2016	2016	EPA		USA	52	46.1	30	57.69	HAMD-17	12	19.1			
129	Rapaport_2016	2016	DHA			51	46.1	30	58.82	HAMD-17	8	19.3	moderate	N	unclear
129	Rapaport_2016	2016	Placebo			52	46.1	30	57.69	HAMD-17	8	19.3			

130	Ravi	2016	EPA+DHA		Iran	50	39.5	18	36.00	BDI-II	8	19.3			
130	Ravi	2016	Placebo			50	39.84	17	34.00	BDI-II	8	28.42	mild	N	Y
131	Sepehrmanesh	2016	Vitamin_D		Iran	20	36.5	17	85.00	BDI-II	8	27.2			
131	Sepehrmanesh	2016	Placebo			20	36.1	17	85.00	BDI-II	8	25.2	mild	N	N
132	Wang	2016	Vitamin_D		China	212			0.00	BDI-II	8	28.5			
132	Wang	2016	Placebo			204			0.00	BDI-II	52	22.7	mild	unclear	Y
133	Akkasheh	2016	Probiotics		Iran	20	38.3		0.00	BDI	52	21.9			
133	Akkasheh	2016	Placebo			20	35.1		0.00	BDI	8		mild	N	N
134	Kolouri	2016	Nepta		Iran	33	35.42	25	75.76	BDI-II	8				
134	Kolouri	2016	ADT	Sertraline		33	35.12	25	75.76	BDI-II	6	27.42	mild	N	N
135	Rajizadeh	2017	Mg		Iran	26	32.2		0.00	BDI-II	6	24.51			
135	Rajizadeh	2017	Placebo			27	32.07		0.00	BDI-II	8	26.9	moderate	N	N
136	Sheikh	2017	Fe		Iran	35	32.3		0.00	EPDS	8	25.6			
136	Sheikh	2017	Placebo			35	31.3		0.00	EPDS	6	13.4	mild	N	Y
137	Kashani	2017	Saffron		Iran	32	29.21	32	100.00	HAMD-17	6	12.9			
137	Kashani	2017	ADT	Fluoxetine		32	32.09	32	100.00	HAMD-17	6	16.53	mild	N	Y
138	Tabeshpour	2017	Saffron		Iran	30	28.1	30	100.00	BDI-II	6	16.65			
138	Tabeshpour	2017	Placebo			30	38	30	100.00	BDI-II	8	20	mild	N	Y
139	Ghajar	2017	Saffron		Iran	30	37.9	11	36.67	HAMD-17	8	19.7			
139	Ghajar	2017	ADT	Citalopram		30	34.17	15	50.00	HAMD-17	6	17.2	mild	N	N
140	Romijn	2017	Probiotics		New Zealand	40	35.8	20	50.00	MADRS	6	17.5			
140	Romijn	2017	Placebo			39	35.1	30	76.92	MADRS	8	28.3	moderate	Y	N
141	Lopresti_2017	2017	Curcumin+Saffron+<br>ADT	Not mention	Australia	26	41.22	22	84.62	IDS-SR3	8	27			
141	Lopresti_2017	2017	Curcumin+ADT	Not mention		61	43.7	55	90.16	IDS-SR3	12	34.65	moderate	N	N

141	Lopresti_2017	2017	ADT	Not mention		36	42.11	32	88.89	IDS-SR3	12	34.15		
142	Abedimanesh_2017	2017	Saffron		Iran	39	54.89	18	46.15	BDI-II	12	35.29		
142	Abedimanesh_2017	2017	Placebo			19	56.63	11	57.89	BDI-II	8	26.97	moderate	N	Y
143	Targum	2018	SAMe+ADT	Not mention	USA	118	48..3	78	66.10	HAMD-17	8	27.42		
143	Targum	2018	ADT	Not mention		116	46.1	79	68.10	HAMD-17	6	21.2	mild	Y	N
144	Sarris_2018	2019	SAMe+Vitamin_B12+Folate+ADT	Not mention	Australia	37	43.4	29	78.38	MADRS	6	21.4		
144	Sarris_2018	2019	ADT	Not mention		39	45.9	29	74.36	MADRS	8	25.7	moderate	N	N
145	Ghorbani	2018	Probiotics+ADT	Fluoxetine	Iran	20	34.45	14	70.00	HAMD-17	8	25.6		
145	Ghorbani	2018	ADT	Fluoxetine		20	35.5	14	70.00	HAMD-17	10	22.9	moderate	N	unclear
146	Jahangard	2018	EPA+DHA		Iran	25	41.28	8	32.00	MADRS	10	23.5		
146	Jahangard	2018	ADT			25	43.64	8	32.00	MADRS	12	37.2	severe	N	N
147	Opiyo	2018	EPA+DHA		South Africa	96	26	96	100.00	BDI-II	12	32.6		
147	Opiyo	2018	Placebo			186	26	186	100.00	BDI-II	8	20	mild	N	Y
148	Alavi	2018	Vitamin_D+ADT	Not mention	Iran	39	68.7	19	48.72	GDS-15	8	21		
148	Alavi	2018	ADT	Not mention		39	67	20	51.28	GDS-15	8	9.25	mild	N	N
149	Zhang	2018	Vitamin_D		China	58	38.3	50	86.21	BDI-II	8	8.9		
149	Zhang	2018	Placebo			65	40.2	53	81.54	BDI-II	8	24.6	mild	unclear	Y
150	Kanchanatawan	2018	Curcumin+ADT	Not mention	Thailand	33	42.6		0.00	MADRS	8	23.3		
150	Kanchanatawan	2018	ADT	Not mention		32	46.2		0.00	MADRS	12	27.6	moderate	N	N
151	Ryszewska-Pokrasniewicz	2018	Mg+ADT	Fluoxetine	Poland	17	38.1		0.00	HAMD-21	12	27.8		
151	Ryszewska-Pokrasniewicz	2018	ADT	Fluoxetine		15	49.7		0.00	HAMD-21	8	30.5	moderate	N	N

152	Kashani	2018	Saffron		Iran	28	55.71	28	100.00	HAMD-17	8	27.5			
152	Kashani	2018	Placebo			28	55.43	28	100.00	HAMD-17	6	15.29	mild	N	Y
153	Majeed	2018	Probiotics		India	20	40.36	17	85.00	HAMD	6	15.96			
153	Majeed	2018	Placebo			20	43.88	17	85.00	HAMD	12	13.6	mild	Y	Y
154	Ghazizadeh-Hashemi	2018	PEA+ADT	Citalopram	Iran	27	35.37	8	29.63	HAMD-17	12	14.5			
154	Ghazizadeh-Hashemi	2018	ADT	Citalopram		27	33.96	11	40.74	HAMD-17	6	24.89	severe	N	N
155	Dai	2018	Ginkgo+ADT	Citalopram	China	68	66.48	35	51.47	HAMD-24	6	25			
155	Dai	2018	ADT	Citalopram		68	66.82	37	54.41	HAMD-24	8	31.03	moderate	N	N
156	Rudzki	2019	Probiotics+ADT	SSRIs	Poland	30	39.13	23	76.67	HAMD-17	8	31.04			
156	Rudzki	2019	ADT	SSRIs		30	38.9	20	66.67	HAMD-17	8	21.53	moderate	N	N
157	Chang	2019	EPA+DHA		Taiwan	30	61.1	12	40.00	HAMD-17	8	22			
157	Chang	2019	Placebo			29	61.93	16	55.17	HAMD-17	8	19	moderate	N	Y
158	Nishi	2019	EPA+DHA		Japan	15	32.3		0.00	HAMD-17	8	19.17			
158	Nishi	2019	Placebo			16	32.5		0.00	HAMD-17	12	14.2	mild	N	N
159	Tayama	2019	EPA+DHA		Japan	47	39.6	18	38.30	BDI-II	12	16.69			
159	Tayama	2019	Placebo			43	41.2	21	48.84	BDI-II	12	12.3	mild	N	unclear
160	Hansen	2019	Vitamin_D+ADT	SSRIs, SNRIs	Denmark	28	39.6		0.00	HAMD-17	12	12.2			
160	Hansen	2019	ADT	SSRIs, SNRIs		34	38.7		0.00	HAMD-17	12	18.4	moderate	N	N
161	Kazemi	2019	Probiotics+ADT	Sertraline, Fluoxetine, Citalopram, Amitriptyline	Iran	74	36.73	54	72.97	BDI	12	18			
161	Kazemi	2019	ADT	Sertraline, Fluoxetine, Citalopram, Amitriptyline		36	36	24	66.67	BDI	8	18.24	mild	N	N

162	Sarris_2019	2019	SAMe+EPA+DHA +Folic+5HTP+Zinc +ADT	SSRIs, SNRIs, NARIs TCAs, 5-HT2c antagonist	Australia	56	40.2		0.00	MADRS	8	18.74			
162	Sarris_2019	2019	ADT	SSRIs, SNRIs, NARIs TCAs, 5-HT2c antagonist		57	44.7		0.00	MADRS	8	24	moderate	N	N
163	Liang	2019	Ginkgo+ADT	Venlafaxine	China	40	60.86	18	45.00	HAMD	8	24.6			
163	Liang	2019	ADT	Venlafaxine		40	60.52	21	52.50	HAMD	8	23.7	moderate	N	Y
164	Carney_2019	2019	EPA+ADT	Sertraline	USA	71.00	58.8	26	36.62	HAMD-17	8	23.7			
164	Carney_2019	2019	ADT	Sertraline		73.00	60.5	30	41.10	HAMD-17	10	17.4	moderate	N	Y
165	Akhondzadeh_2 020	2020	Saffron		Iran	27.00	37.00	27.00	100.00	BDI-II	10	17			
165	Akhondzadeh_2 020	2020	Placebo			25.00	39.80	25.00	100.00	BDI-II	12	22.2	mild	N	unclear
166	Amini_2020	2020	Vitamin_D+Ca		Iran	26	26.88	26	100.00	EPDS	12	21.8			
166	Amini_2020	2020	Vitamin_D			26	29.25	26	100.00	EPDS	8	17.41	moderate	N	N
166	Amini_2020	2020	Placebo			26	28.92	26	100.00	EPDS	8	17.5			
167	Sakurai_2020	2020	SAMe		USA	35.00	45.83		0.00	HAMD-17	8	16.43			
167	Sakurai_2020	2020	ADT	Escitalopram		32.00	45.91		0.00	HAMD-17	12	18.11	moderate	N	N
167	Sakurai_2020	2020	Placebo			35.00	45.92		0.00	HAMD-17	12	19.81			
168	Sarris_2020	2020	SAMe		Australia	25.00	45.20	18.00	72.00	MADRS	12	19.36			
168	Sarris_2020	2020	Placebo			24.00	42.00	21.00	87.50	MADRS	8	22.4	mild	Y	N
169	Chahwan	2020	Probiotics		Australia	34	36.65	21	61.76	BDI	8	22.2			
169	Chahwan	2020	Placebo			37	35.49	28	75.68	BDI	8	28.91	moderate	N	N
170	Kaviani	2020	Vitamin_D		Iran	28	43.14	27	96.43	BDI	8	27.97			
170	Kaviani	2020	Placebo			28	42.86	23	82.14	BDI	8	23.86	mild	N	N

171	Reininghaus	2020	Probiotics+ Vitamin_B7		Austria	28	43	20	71.43	HAMD	8	21.79			
171	Reininghaus	2020	Placebo			33	40.11	27	81.82	HAMD	4	15.07	mild	Y	N
172	Vellekkatt	2020	Vitamin_D+ADT	Not mention	India	23	36.2	17	73.91	HAMD	4	14.73			
172	Vellekkatt	2020	ADT	Not mention		23	35.8	14	60.87	HAMD	12	19.4	mild	N	N
173	Asadi	2020	Curcumin		Iran	40		35	87.50	DASS	12	17.44			
173	Asadi	2020	Placebo			40		35	87.50	DASS	8	16.7	severe	N	Y
174	Yosaee	2020	Zinc+Vitamin_D		Iran	25	38.93		0.00	BDI-II	8	17.5			
174	Yosaee	2020	Zinc			24	38.71		0.00	BDI-II	12		mild	Y	Y
174	Yosaee	2020	Vitamin_D			27	38.28		0.00	BDI-II	12				
174	Yosaee	2020	Placebo			22	37.31		0.00	BDI-II	12				
175	Zhu	2020	Vitamin_D		China	62	46.3	44	70.97	HAMD	12				
175	Zhu	2020	Placebo			45	43.3	34	75.56	HAMD	8	30	severe	N	N
176	Sepehrmanes	2016	Folate+ADT	Not mention	Iran	45	35.11	26	57.78	HAMD	8	29.2			
176	Sepehrmanes	2016	ADT	Not mention		45	36.35	30	66.67	HAMD	26		Mild	unclear	unclear
177	Saccarello	2020	SAMe+Probiotics		Italy	45	48.6	38	84.44	Z-SDS	26				
177	Saccarello	2020	Placebo			44	47.5	35	79.55	Z-SDS	26				
178	Kumar	2022	Vitamin_D+ADT	Escitalopram	India	31	34.9	20	64.52	HAMD	12	25.7	moderate	N	N
178	Kumar	2022	ADT	Escitalopram		28	39.3	22	78.57	HAMD	12	25.8		N	N
179	Mischoulon	2022	EPA		USA	15	43.01	33	220.00	IDS-C30	12			N	Y
179	Mischoulon	2022	Placebo			15	50.3	12	80.00	IDS-C30	12			N	Y
180	Schaub	2022	Probiotics+ADT	Not mention	Switzerland.	9.00	39.43	14	155.56	HAMD	8			N	N
180	Schaub	2022	ADT	Not mention		11.00	38.77	13	118.18	HAMD	8			N	N
181	Tarutani	2022	Prebiotics+ADT	Not mention	Japan	25.00	53	8	32.00	MADRS	24		mild	N	N
181	Tarutani	2022	ADT	Not mention		20.00	53	9	45.00	MADRS	24			N	N

182	Tian	2022	Probiotics+ADT	Not mention	China	25.00	51.32	14	56.00	HAMD-24	4		mild	N	N
182	Tian	2022	ADT	Not mention		20.00	48.15	16	80.00	HAMD-24	4			N	N
183	Gawlik-Kotelnicka	2024	Probiotics+ADT		Poland	51	34.3	43	84.31	MADRS	8.5	20.43	moderate	N	N
183			ADT			44	35.7	38	86.36	MADRS	8.5	20.94		N	N
184	Hashemi-Mohammadabad	2024	Probiotics+ADT		Iran	58	35.03	58	100.00	HAMD	8	12.57	mild	N	N
184			ADT			54	35.03	54	100.00	HAMD	8	11.77		N	N
185	Khadem	2024	Prebiotics+ADT		Iran	17	35.03	17	100.00	HAMD	8	11.41	mild	N	N
185			ADT			17	35.11	17	100.00	HAMD	8	11.65		N	N
186	Lin	2024	Prebiotics+ADT		Taiwan	16	39.31	12	75.00	HAMD	8	19.88	moderate	N	N
186			ADT			16	36.81	12	75.00	HAMD	8	20.38		N	N
187	Strodl	2024	Probiotics+Mg+CoQ10+ADT		Australia	58	40.58	40	68.97	BDI	8	30.78	moderate	N	N
187			ADT			62	35.76	41	66.13	BDI	8	29.81		N	N
188	Yang	2022	EPA+DHA+ADT		China	36	26.33	21	58.33	HAMD	12	29.5	moderate	N	N
188			ADT			36	27.11	25	69.44	HAMD	12	29.97		N	N
189	Wu	2024	EPA+DHA		Taiwan	30	35	25	83.33	HAMD	12	19.17	moderate	N	N
189			Placebo			30	35	25	83.33	HAMD	12	20.67		N	N
190	Shamabadi	2023	L-theanine+ADT		Iran	25	34.44	13	52.00	HAMD	6	26.88	severe	N	N
190			ADT			25	32.52	9	36.00	HAMD	6	26.52		N	N
191	Torkaman	2024	L-arginine+ADT		Iran	16	40.5	16	100.00	HAMD	8	18.06	moderate	N	N
191			ADT			16	40.1	16	100.00	HAMD	8	15.06		N	N
192	Kolahdooz	2023	Saffron		Iran	32	27.93	32	100.00	HAMD	12	21.06	moderate	N	N
192			ADT			32	24.9	32	100.00	HAMD	12	20.75		N	N

ADT: Antidepressant; Ca: Calcium ; CESD: Center for Epidemiological Studies-Depression ; BDI: Beck depression inveotry ;DHA: Docosahexaenoic Acid ; DASS : Depression Anxiety

Stress Scales; E Amoenum : Echium amoenum; EPA :Eicosapentaenoic acid; EPDS : Edinburgh Postnatal Depression Scale; GDS :Geriatric Depression Scale; HAMD : Hamilton Depression Rating Scale ; IDS-SR: The inventory of depressive symptomatology-self rating; Fe : Ferrum;MADRS : Montgomery-Asberg Depression Rating Scale ; MAOIs:Monoamine oxidase inhibitor ;Mg: Magnesium; NARIs:Noradrenaline reuptake inhibiting antidepressants; PEA: Palmitoylethanolamide; R rosea : Rhodiola rosea; SAMe: S-Adenosyl Methionine ; SJW: St. John's wort ; SNRIs:Serotonin–norepinephrine reuptake inhibitors; SSRIs:Selective serotonin reuptake inhibitors; TCAs:Tricyclic antidepressants; Vitamin B1: Thiamine ; Vitamin B6: Pyridoxine; Vitamin B7: Biotin; Vitamin B: Vitamin B complex; Vitamin B12 : Cobalamin; Vitamin C: Ascorbic acid; Vitamin D: Cholecalciferol; 5HTP: 5-Hydroxytryptophan; Z-SDS: Zung Self-Rating Depression Scale

6. Risk of bias

6.1.Included studies rated against the Cochrane Risk of Bias tool (ROB 1.0))

Study ID	Author year	year	Random Sequence generation (selection bias) D1	Allocation Concealment (Selection bias) D1	Other sources of bias D2	Incomplete outcome data D3	Selective outcome reporting D5	Blinding of Participants and personnel D4	Blinding of outcome assessors D4	Overall
1	Ayuso GutierrezLópez-Ibor Alino 1971	1971	Unclear	Unclear	Unclear	Low	Low	Low	Low	Low
2	Hoes 1981	1981	Unclear	Unclear	Unclear	Low	Low	High	High	High
3	Coppen 1986	1986	Unclear	Unclear	High	Unclear	Unclear	Low	Low	High
4	Thomas 1987	1987	Unclear	Unclear	Unclear	Low	Low	Low	Low	Low
5	Bell 1988	1988	Unclear	Unclear	Unclear	Unclear	Unclear	Low	Low	Moderate
6	Potkin 1988	1988	Unclear	Unclear	Unclear	Unclear	Unclear	Low	Low	Moderate
7	Kagan 1990	1990	Low	Unclear	High	Unclear	Low	Low	Low	Moderate
8	Godfrey 1990	1990	Unclear	Unclear	Unclear	Low	Low	Low	Low	Low
9	Gecele 1991	1991	Unclear	Unclear	Unclear	Unclear	Low	Low	Low	Moderate
10	Berlanga 1992	1992	Unclear	Unclear	Unclear	Low	Low	Low	Low	Low
11	Fava 1992	1992	Unclear	Unclear	Unclear	High	Low	Low	Low	Moderate
12	De Vanna 1992	1992	Unclear	Unclear	Unclear	Low	Low	Low	Low	Low
13	Salmaggi 1993	1993	Unclear	Unclear	Unclear	Unclear	Low	Low	Low	Moderate
14	Bell 1994	1994	Unclear	Unclear	High	Unclear	Low	Low	Low	Moderate
15	Hubner 1994	1994	Low	Low	Unclear	Unclear	Low	Low	Low	Low
16	Hansgen 1994	1994	Low	Unclear	Unclear	Low	Unclear	Low	Low	Low
17	Vorbach 1994	1994	Low	Unclear	Unclear	Low	Low	Low	Low	Low

18	Harrer 1994	1994	Low	Unclear	Unclear	Low	Unclear	Low	Low	Low
19	Sommer 1994	1994	Low	Unclear	Unclear	Low	Unclear	Low	Low	Low
20	Wheatley 1997	1997	Low	Unclear	Unclear	Low	Low	Low	Low	Low
21	Vorbach 1997	1997	Low	Unclear	Unclear	Low	Low	Low	Low	Low
22	Laakmann 1998	1998	Low	Unclear	Unclear	Low	Low	Low	Low	Low
23	Schrader 1998	1998	Low	Low	Unclear	Low	Low	Low	Low	Low
24	Nemets 1999	1999	Unclear	Unclear	Unclear	Low	Low	Low	Low	Low
25	Levine 1999	1999	Unclear	Unclear	Unclear	Low	Low	Low	Low	Low
26	Harrer 1999	1999	Low	Unclear	Unclear	Low	High	Low	Low	Moderate
27	Philipp 1999	1999	Low	Unclear	Unclear	Low	Low	Low	Low	Low
28	Levitan 2000	2000	Unclear	Unclear	Unclear	Unclear	Low	Low	Low	Moderate
29	Coppen 2000	2000	Low	Unclear	Unclear	Low	Low	Low	Low	Low
30	Schrader 2000	2000	Low	Unclear	Unclear	Low	Low	Low	Low	Low
31	Woelk 2000	2000	Low	Unclear	Unclear	Unclear	Low	Low	Low	Low
32	Brenner 2000	2000	Low	Unclear	Unclear	Low	Low	Low	Low	Low
33	Shelton 2001	2001	Low	Low	Unclear	Low	Low	Low	Low	Low
34	Kalb 2001	2001	Low	Low	Unclear	Low	Low	Low	Low	Low
35	SJ group	2001	Low	Low	Unclear	Low	Low	Low	Low	Low
36	Delle Chiaie 2002	2002	Low	Low	Unclear	Unclear	Low	Low	Low	Low
37	Pancheri 2002	2002	Low	Unclear	Unclear	Unclear	Low	Low	Low	Low
38	Nemets 2002	2002	Low	Unclear	Unclear	Low	Low	Low	Low	Low
39	Peet 2002	2002	Low	Unclear	Unclear	Low	Low	Low	Low	Low
40	Lecrubier 2002	2002	Unclear	Unclear	Unclear	Low	Low	Low	Low	Low
41	Behnke 2002	2002	Low	Low	Unclear	High	Low	Low	Low	Moderate
42	van Gurp 2002	2002	Low	Low	Unclear	Low	Low	Low	Low	Low
43	Zanarini 2003	2003	Low	Unclear	Unclear	Low	Low	Low	Low	Low

44	Su 2003	2003	Unclear	Unclear	Unclear	High	Low	Low	Low	Moderate
45	Marangell 2003	2003	Unclear	Unclear	High	Low	Low	Low	Low	Moderate
46	Nowak 2003	2003	Unclear	Unclear	High	High	Low	Low	Low	High
47	Akhondzadeh 2003	2003	Unclear	Unclear	Unclear	Unclear	Low	Unclear	Unclear	High
48	Akhondzadeh 2004	2004	Low	Low	Unclear	Unclear	Low	Low	Low	Low
49	Uebelhack 2004	2004	Low	Low	Unclear	Low	Low	Low	Low	Low
50	Silvers 2005	2005	Low	Unclear	Unclear	Low	Low	Low	Low	Low
51	Docherty 2005	2005	Unclear	Unclear	Unclear	Low	Low	Low	Low	Low
52	Akhondzadeh 2005	2005	Low	Low	Unclear	Unclear	Low	Low	Low	Low
53	Noorbala 2005	2005	Low	Low	Unclear	Low	Low	Low	Low	Low
54	Bjerkenstedt 2005	2005	Low	Low	Unclear	Low	Low	Low	Low	Low
55	Szegedi 2005	2005	Low	Low	Unclear	Low	Low	Low	Low	Low
56	Gastpar 2005	2005	Low	Low	Unclear	Low	Low	Low	Low	Low
57	Fava 2005	2005	Low	Low	Unclear	High	Low	Low	Low	Moderate
58	Moshiri 2006	2006	Low	Low	Unclear	Low	Low	Low	Low	Low
59	Gastpar 2006	2006	Low	Unclear	Unclear	Unclear	Low	Low	Low	Low
60	Kasper 2006	2006	Low	Low	Unclear	Low	Low	Low	Low	Low
61	Moreno 2006	2006	Unclear	Unclear	Unclear	Low	Low	Unclear	Unclear	Moderate
62	Randlov 2006	2006	Unclear	Unclear	Unclear	Low	Low	Low	Low	Low
63	Sayyah 2006	2006	Low	Unclear	Unclear	Low	Low	Low	Low	Low
64	Grenyer 2007	2007	Low	Unclear	Unclear	Low	Low	Low	Low	Low
65	Akhondzadeh Basti 2007	2007	Low	Low	Unclear	Low	Low	Low	Low	Low
66	Darbinyan 2007	2007	Unclear	Low	Unclear	Unclear	Low	Low	Low	Low
67	Ille 2007	2007	Unclear	Unclear	Unclear	Unclear	Low	Low	Low	Moderate
68	Resler 2008	2008	Low	Unclear	Unclear	Low	Low	Low	Low	Low
69	Jazayeri 2008	2008	Low	Unclear	Unclear	Unclear	Low	Low	Low	Low

70	Rogers 2008	2008	Low	Low	Unclear	Unclear	Low	Low	Low	Low
71	Rees 2008	2008	Low	Low	Unclear	Low	Low	Low	Low	Low
72	Su 2008	2008	Low	Low	Unclear	Low	Low	Low	Low	Low
73	da Silva 2008	2008	Low	Low	Unclear	Low	Low	Low	Low	Low
74	Freeman 2008	2008	Unclear	Unclear	Unclear	Low	Low	Unclear	Unclear	Moderate
75	Barragan-Rodriguez 2008	2008	Low	Unclear	Unclear	High	Low	Unclear	Low	Moderate
76	Raza-ur-Rahman 2008	2008	Low	Low	Unclear	High	Low	Low	Low	Moderate
77	Başoğlu 2009	2009	Low	Unclear	Unclear	Low	Low	Low	Low	Low
78	Mischoulon 2009	2009	Low	Low	Unclear	Low	Low	Low	Low	Low
79	Carney 2009	2009	Low	Low	Unclear	Low	Low	Low	Low	Low
80	Sarris 2009	2009	Low	Low	Unclear	Low	Low	Low	Low	Low
81	Siwek 2009	2009	Low	Low	Unclear	Low	Low	Low	Low	Low
82	Papakostas 2010	2010	Unclear	Unclear	Unclear	High	Low	Low	Low	Moderate
83	Lucas 2009	2009	Low	Unclear	Unclear	Low	Low	Low	Low	Low
84	Bot 2010	2010	Unclear	Unclear	Unclear	Low	Low	Low	Low	Low
85	Mannel 2010	2010	Low	Low	Unclear	Low	Low	Low	Low	Low
86	Liu 2010	2010	Unclear	Unclear	Unclear	Unclear	Low	High	High	High
87	Rondanelli 2010	2011	Low	Low	Unclear	Low	Low	Low	Low	Low
88	Rapaport 2011	2011	Low	Low	Unclear	High	Low	Low	Low	Moderate
89	Papakostas 2012	2012	Unclear	Low	High	Low	Low	Low	Low	Moderate
90	Lesperance 2011	2011	Low	Low	Unclear	Low	Low	Low	Low	Low
91	Gertsik 2012	2012	Low	Unclear	Unclear	Low	Low	Low	Low	Low
92	Lyoo 2012	2012	Low	Unclear	Unclear	High	Low	Low	Low	Moderate
93	Sarris 2012	2012	Low	Low	Unclear	High	Low	Low	Low	Moderate
94	Pakseresht 2012	2012	Low	Unclear	Unclear	Unclear	Low	Low	Low	Low
95	Syed 2013	2013	Low	Low	High	Low	Low	High	High	High

96	Lewis 2013	2013	Low	Low	Unclear	Unclear	Low	Low	Low	Low
97	Rizzo 2012	2012	Low	Low	High	Unclear	Low	Low	Low	Moderate
98	Khajehnasiri 2013	2013	Low	Low	Unclear	Low	Low	Low	Low	Low
99	Mozaffari-Khosravi 2013	2013	Low	Low	Low	Low	Low	Low	Low	Low
100	Khoraminya 2013	2013	Low	Unclear	Unclear	Unclear	Low	Low	Low	Low
101	Mozaffari-Khosravi 2013	2013	Low	Unclear	Unclear	Unclear	Unclear	High	High	High
102	Bergman 2013	2013	Low	Low	Unclear	Low	Low	Low	Low	Low
103	Nemets 2013	2013	Unclear	Unclear	Unclear	Low	Low	Low	Low	Low
104	Bersani 2013	2013	Low	Unclear	Unclear	Unclear	Low	Low	Low	Low
105	Safa 2013	2013	Unclear	Unclear	High	Low	Low	Low	Low	Moderate
106	Mozurkewich 2013	2013	Low	Unclear	Low	Low	Low	Low	Low	Low
107	Mischoulon 2014	2014	Low	Unclear	Unclear	Low	Low	Low	Low	Low
108	Sarris 2014	2014	Low	Unclear	Unclear	Low	Low	Low	Low	Low
109	Bedson 2014	2014	Low	Low	Unclear	Low	Low	Low	Low	Low
110	Almeida 2014	2014	Low	Unclear	Unclear	Low	Low	Low	Low	Low
111	Dashti-Khavidaki 2014	2014	Low	Low	Unclear	Low	Low	Low	Low	Low
112	Kaviani 2014	2014	Low	Unclear	Unclear	Low	Low	Low	Low	Low
113	Sanmukhani 2014	2014	Low	Low	Unclear	Low	Low	Low	Low	Low
114	Lopresti 2014	2014	Low	Low	Unclear	Low	Low	Low	Low	Low
115	Shahmansouri 2014	2014	Low	Low	Unclear	Low	Low	Low	Low	Low
116	Park 2015	2015	Low	Low	Unclear	Low	Low	Low	Low	Low
117	Ginty 2015	2015	Low	Low	High	Low	Unclear	Low	Low	Moderate
118	Mischoulon 2015	2015	Low	Low	Unclear	Low	Low	Low	Low	Low
119	Sahraian 2015	2015	Low	Low	Unclear	Low	Low	Low	Low	Low
120	Panahi 2015	2015	Low	Unclear	Unclear	High	Low	High	High	High
121	Yu 2015	2015	Unclear	Unclear	Unclear	Unclear	Low	Low	Low	Low

122	Talaei 2015	2015	Low	Low	Unclear	Low	Low	Low	Low	Low
123	Mao 2015	2015	Low	Low	Unclear	Unclear	Low	Low	Low	Low
124	Panahi 2015 (2)	2015	Unclear	High	Unclear	High	Low	High	High	High
125	Gavrilova 2015	2015	Unclear	Unclear	Unclear	Low	Low	Low	Low	Moderate
126	Ghaleiha 2016	2016	Low	Low	Unclear	Unclear	Low	Low	Low	Low
127	Masoumi 2016	2016	Low	Low	Unclear	Low	Low	Low	Low	Low
128	Shinto 2016	2016	Low	Low	Unclear	Low	Low	Low	Low	Low
129	Rapaport 2016	2016	Unclear	Unclear	Unclear	Low	Low	Low	Low	Low
130	Ravi 2016	2016	Low	Low	Unclear	Low	Low	Low	Low	Low
131	Sepehrmanesh 2016	2016	Low	Low	Unclear	Low	Low	Low	Low	Low
132	Wang 2016	2016	Low	Low	Unclear	Low	Low	Low	Low	Low
133	Akkasheh 2016	2016	Low	Low	Unclear	High	Low	Low	Low	Moderate
134	Kolouri 2016	2016	Low	Low	Unclear	Low	Low	Low	Low	Low
135	Sepehrmanesh Abedi 2016	2016	Low	Unclear	Unclear	Low	Low	Low	Low	Low
136	Lopresti 2017	2017	Low	Low	Unclear	Low	Low	Low	Low	Low
137	Rajizadeh 2017	2017	Unclear	Unclear	Unclear	Low	Low	Low	Low	Low
138	Sheikh 2017	2017	Low	Low	Unclear	Low	Low	Low	Low	Low
139	Kashani 2017	2017	Low	Low	Unclear	Low	Low	Low	Low	Low
140	Tabeshpour 2017	2017	Low	Low	High	Low	Low	Low	Low	Moderate
141	Ghajar 2017	2017	Low	Low	Unclear	Low	Low	Low	Low	Low
142	Romijn 2017	2017	Low	Low	Unclear	High	Low	Low	Low	Moderate
143	Abedimanesh 2017	2017	Low	Low	Unclear	Low	Low	Low	Low	Low
144	Targum 2018	2018	Low	Unclear	Unclear	Low	Low	Low	Low	Low
145	Sarris 2018	2018	Low	Unclear	Unclear	Low	Low	Low	Low	Low
146	Jahangard 2018	2018	Low	Unclear	Unclear	Low	Low	Low	Low	Low
147	Opiyo 2018	2018	Low	Low	Unclear	High	Low	Low	Low	Moderate

148	Alavi 2018	2018	Low	Low	Unclear	Low	Low	Low	Low	Low
149	Zhang 2018	2018	Low	Low	Unclear	Low	Low	Low	Low	Low
150	Kanchanatawan 2018	2018	Low	Low	Unclear	Low	Low	Low	Low	Low
151	Ryszewska-Pokrasniewicz 2018	2018	Low	Unclear	Unclear	High	Low	Low	Low	Moderate
152	Kashani 2018	2018	Low	Low	Unclear	Low	Low	Low	Low	Low
153	Majeed 2018	2018	Unclear	Low	Unclear	High	Low	Low	Low	Moderate
154	Ghorbani 2018	2018	Low	Low	Unclear	Low	Low	Low	Low	Low
155	Ghazizadeh-Hashemi 2018	2018	Low	Low	Unclear	Low	Low	Low	Low	Low
156	Dai 2018	2018	High	High	Unclear	Unclear	Low	High	High	High
157	Chang 2019	2019	Unclear	Unclear	Unclear	Unclear	Low	Low	Low	Moderate
158	Nishi 2019	2019	Low	Unclear	Unclear	Unclear	Low	Low	Low	Low
159	Sarris 2019	2019	Low	Unclear	Unclear	Unclear	Low	Low	Low	Low
160	Hansen 2019	2019	Low	Low	Unclear	Unclear	Low	Low	Low	Low
161	Kazemi 2019	2019	Low	Low	Unclear	High	Low	Low	Low	Moderate
162	Rudzki 2019	2019	Low	Low	Unclear	High	Low	Low	Low	Moderate
163	Liang 2019	2019	Unclear	Unclear	Unclear	Unclear	Low	Low	Low	Moderate
164	Carney 2019	2019	Low	Low	Unclear	Low	Low	Low	Low	Low
165	Chahwan 2019	2019	Low	Low	Unclear	Low	Low	Low	Low	Low
166	Tayama 2019	2019	Low	Low	Unclear	Low	Low	Low	Low	Low
167	Akhondzadeh 2020	2020	Low	Low	Unclear	Low	Low	Low	Low	Low
168	Sarris 2020	2020	Low	Low	Unclear	Low	Low	Low	Low	Low
169	Sakurai 2020	2020	Low	Unclear	Unclear	High	Low	Low	Low	Moderate
170	Amini 2020	2020	Low	Low	Unclear	Low	Low	Low	Low	Low
171	Kaviani 2020	2020	Low	Low	Unclear	Low	Low	Low	Low	Low
172	Reininghaus 2020	2020	Low	Low	Unclear	Low	Low	Low	Low	Low
173	Vellekkatt 2020	2020	Low	Low	Unclear	Low	Low	Low	Low	Low

174	Asadi 2020	2020	Low	Low	Unclear	Low	Low	Low	Low	Low
175	Yosaee 2020	2020	Low	Low	Unclear	Low	Low	Low	Low	Low
176	Zhu 2020	2020	Low	Low	Unclear	Low	Unclear	Low	Low	Low
177	Saccarello et al. 2020	2020	Low	Low	Unclear	Low	Low	Low	Low	Low
178	Kumar 2022	2022	Low	Low	Unclear	Low	Low	Low	Low	Low
179	Mischoulon 2022	2022	Low	Low	Unclear	Low	Low	Low	Low	Low
180	Schaub 2022	2022	Low	Unclear	Unclear	Low	Low	Low	Low	Low
181	Tarutani 2022	2022	Low	Low	Unclear	Low	Low	Low	Low	Low
182	Tian 2022	2022	Low	Low	Unclear	Low	Low	Low	Low	Low
183	Gawlik-Kotelnicka 2022	2022	Low	Low	Unclear	Low	Low	Low	Low	Low
184	Hashemi-Mohammadabad 2024	2024	Low	Low	Unclear	Low	Low	Low	Low	Low
185	Khadem 2024	2024	Low	Low	Unclear	Low	Low	Low	Low	Low
186	Lin 2024	2024	Low	Low	Unclear	Low	Low	Low	Low	Low
187	Strodl 2024	2024	Low	Low	Unclear	Low	Low	Low	Low	Low
188	Yang 2024	2024	Low	Low	Unclear	Low	Low	Low	Low	Low
189	Wu 2022	2022	Low	Low	Unclear	Low	Low	Low	Low	Low
190	Shamabadi 2024	2024	Low	Low	Unclear	Low	Low	Low	Low	Low
191	Torkaman 2023	2023	Low	Low	Unclear	Low	Low	Low	Low	Low
192	Kolahdooz 2024	2024	Low	Low	Unclear	Low	Unclear	Low	Low	Low

6.2.Included studies rated against the revised Cochrane Risk of Bias tool (ROB 2.0)

	Risk of bias domains					
	D1	D2	D3	D4	D5	Overall
Ayuso GutierrezLópez-Ibor Alino 1971	-	-	+	+	+	-
Hoes 1981	-	-	+	X	+	X
Coppen 1986	-	X	-	+	-	X
Thomas 1987	-	-	+	+	+	-
Bell 1988	-	-	-	+	-	-
Potkin 1988	-	-	-	+	-	-
Kagan 1990	+	X	-	+	+	X
Godfrey 1990	-	-	+	+	+	-
Gecele 1991	-	-	-	+	+	-
Berlanga 1992	-	-	+	+	+	-

Fava 1992	-	-	X	+	+	X
De Vanna 1992	-	-	+	+	+	-
Salmaggi 1993	-	-	-	+	+	-
Bell 1994	-	X	-	+	+	X
Hubner 1994	+	-	-	+	+	-
Hansgen 1994	+	-	+	+	-	-
Vorbach 1994	+	-	+	+	+	-
Harrer 1994	+	-	+	+	-	-
Sommer 1994	+	-	+	+	-	-
Wheatley 1997	+	-	+	+	+	-
Vorbach 1997	+	-	+	+	+	-

Laakmann 1998	+	-	+	+	+	-
Schrader 1998	+	-	+	+	+	-
Nemets 1999	-	-	+	+	+	-
Levine 1999	-	-	+	+	+	-
Harrer 1999	+	-	+	+	X	X
Philipp 1999	+	-	+	+	+	-
Levitan 2000	-	-	-	+	+	-
Coppen 2000	+	-	+	+	+	-
Schrader 2000	+	-	+	+	+	-
Woelk 2000	+	-	-	+	+	-
Brenner 2000	+	-	+	+	+	-

Shelton 2001	+	-	+	+	+	-
Kalb 2001	+	-	+	+	+	-
SJ group	+	-	+	+	+	-
Delle Chiaie 2002	+	-	-	+	+	-
Pancheri 2002	+	-	-	+	+	-
Nemets 2002	+	-	+	+	+	-
Peet 2002	+	-	+	+	+	-
Lecrubier 2002	-	-	+	+	+	-
Behnke 2002	+	-	X	+	+	X
van Gurp 2002	+	-	+	+	+	-
Zanarini 2003	+	-	+	+	+	-

Su 2003	-	-	X	+	+	X
Marangell 2003	-	X	+	+	+	X
Nowak 2003	-	X	X	+	+	X
Akhondzadeh 2003	-	-	-	-	+	-
Akhondzadeh 2004	+	-	-	+	+	-
Uebelhack 2004	+	-	+	+	+	-
Silvers 2005	+	-	+	+	+	-
Docherty 2005	-	-	+	+	+	-
Akhondzadeh 2005	+	-	-	+	+	-
Noorbala 2005	+	-	+	+	+	-
Bjerkenstedt 2005	+	-	+	+	+	-

Szegedi 2005	+	-	+	+	+	-
Gastpar 2005	+	-	+	+	+	-
Fava 2005	+	-	X	+	+	X
Moshiri 2006	+	-	+	+	+	-
Gastpar 2006	+	-	-	+	+	-
Kasper 2006	+	-	+	+	+	-
Moreno 2006	-	-	+	-	+	-
Randlov 2006	-	-	+	+	+	-
Sayyah 2006	+	-	+	+	+	-
Grenyer 2007	+	-	+	+	+	-
Akhondzadeh Basti 2007	+	-	+	+	+	-

Darbinyan 2007	-	-	-	+	+	-
Ille 2007	-	-	-	+	+	-
Resler 2008	+	-	+	+	+	-
Jazayeri 2008	+	-	-	+	+	-
Rogers 2008	+	-	-	+	+	-
Rees 2008	+	-	+	+	+	-
Su 2008	+	-	+	+	+	-
da Silva 2008	+	-	+	+	+	-
Freeman 2008	-	-	+	-	+	-
Barragan-Rodriguez 2008	+	-	X	-	+	X
Raza-ur-Rahman 2008	+	-	X	+	+	X

Başoğlu 2009	+	-	+	+	+	-
Mischoulon 2009	+	-	+	+	+	-
Carney 2009	+	-	+	+	+	-
Sarris 2009	+	-	+	+	+	-
Siwek 2009	+	-	+	+	+	-
Papakostas 2010	-	-	X	+	+	X
Lucas 2009	+	-	+	+	+	-
Bot 2010	-	-	+	+	+	-
Mannel 2010	+	-	+	+	+	-
Liu 2010	-	-	-	X	+	X
Rondanelli 2010	+	-	+	+	+	-

Study

Rapaport 2011	+	-	X	+	+	X
Papakostas 2012	-	X	+	+	+	X
Lesperance 2011	+	-	+	+	+	-
Gertsik 2012	+	-	+	+	+	-
Lyoo 2012	+	-	X	+	+	X
Sarris 2012	+	-	X	+	+	X
Pakseresht 2012	+	-	-	+	+	-
Syed 2013	+	X	+	X	+	X
Lewis 2013	+	-	-	+	+	-
Rizzo 2012	+	X	-	+	+	X
Khajehnasiri 2013	+	-	+	+	+	-

Mozaffari-Khosravi 2013(Vit D)	+	+	+	+	+	+
Khoraminya 2013	+	-	-	+	+	-
Mozaffari-Khosravi 2013 (PUFAs)	+	-	-	X	-	X
Bergman 2013	+	-	+	+	+	-
Nemets 2013	-	-	+	+	+	-
Bersani 2013	+	-	-	+	+	-
Safa 2013	-	X	+	+	+	X
Mozurkewich 2013	+	+	+	+	+	+
Mischoulon 2014	+	-	+	+	+	-
Sarris 2014	+	-	+	+	+	-
Bedson 2014	+	-	+	+	+	-

Almeida 2014	+	-	+	+	+	-
Dashti-Khavidaki 2014	+	-	+	+	+	-
Kaviani 2014	+	-	+	+	+	-
Sanmukhani 2014	+	-	+	+	+	-
Lopresti 2014	+	-	+	+	+	-
Shahmansouri 2014	+	-	+	+	+	-
Park 2015	+	-	+	+	+	-
Ginty 2015	+	X	+	+	-	X
Mischoulon 2015	+	-	+	+	+	-
Sahraian 2015	+	-	+	+	+	-
Panahi 2015	+	-	X	X	+	X

Yu 2015	-	-	-	+	+	-
Talaei 2015	+	-	+	+	+	-
Mao 2015	+	-	-	+	+	-
Panahi 2015 (2)	-	-	X	X	+	X
Gavrilova 2015	-	-	+	+	+	-
Ghaleiha 2016	+	-	-	+	+	-
Masoumi 2016	+	-	+	+	+	-
Shinto 2016	+	-	+	+	+	-
Rapaport 2016	-	-	+	+	+	-
Ravi 2016	+	-	+	+	+	-
Sepehrmanesh 2016	+	-	+	+	+	-

Yu 2015	-	-	-	+	+	-
Talaei 2015	+	-	+	+	+	-
Mao 2015	+	-	-	+	+	-
Panahi 2015 (2)	-	-	X	X	+	X
Gavrilova 2015	-	-	+	+	+	-
Ghaleiha 2016	+	-	-	+	+	-
Masoumi 2016	+	-	+	+	+	-
Shinto 2016	+	-	+	+	+	-
Rapaport 2016	-	-	+	+	+	-
Ravi 2016	+	-	+	+	+	-
Sepehrmanesh 2016	+	-	+	+	+	-

Wang 2016	+	-	+	+	+	-
Akkasheh 2016	+	-	X	+	+	X
Kolouri 2016	+	-	+	+	+	-
Sepehrmanesh Abedi 2016	+	-	+	+	+	-
Lopresti 2017	+	-	+	+	+	-
Rajizadeh 2017	-	-	+	+	+	-
Sheikh 2017	+	-	+	+	+	-
Kashani 2017	+	-	+	+	+	-
Tabeshpour 2017	+	X	+	+	+	X
Ghajar 2017	+	-	+	+	+	-
Romijn 2017	+	-	X	+	+	X

Abedimanesh 2017	+	-	+	+	+	-
Targum 2018	+	-	+	+	+	-
Sarris 2018	+	-	+	+	+	-
Jahangard 2018	+	-	+	+	+	-
Opiyo 2018	+	-	X	+	+	X
Alavi 2018	+	-	+	+	+	-
Zhang 2018	+	-	+	+	+	-
Kanchanatawan 2018	+	-	+	+	+	-
Ryszewska-Pokrasniewicz 2018	+	-	X	+	+	X
Kashani 2018	+	-	+	+	+	-
Majeed 2018	-	-	X	+	+	X

Ghorbani 2018	+	-	+	+	+	-
Ghazizadeh-Hashemi 2018	+	-	+	+	+	-
Dai 2018	X	-	-	X	+	X
Chang 2019	-	-	-	+	+	-
Nishi 2019	+	-	-	+	+	-
Sarris 2019	+	-	-	+	+	-
Hansen 2019	+	-	-	+	+	-
Kazemi 2019	+	-	X	+	+	X
Rudzki 2019	+	-	X	+	+	X
Liang 2019	-	-	-	+	+	-
Carney 2019	+	-	+	+	+	-

Chahwan 2019	+	-	+	+	+	-
Tayama 2019	+	-	+	+	+	-
Akhondzadeh 2020	+	-	+	+	+	-
Sarris 2020	+	-	+	+	+	-
Sakurai 2020	+	-	X	+	+	X
Amini 2020	+	-	+	+	+	-
Kaviani 2020	+	-	+	+	+	-
Reininghaus 2020	+	-	+	+	+	-
Vellekkatt 2020	+	-	+	+	+	-
Asadi 2020	+	-	+	+	+	-
Yosaee 2020	+	-	+	+	+	-

Zhu 2020	+	-	+	+	-	-
Saccarello et al. 2020	+	-	+	+	+	-
Kumar 2022	+	-	+	+	+	-
Mischoulon 2022	+	-	+	+	+	-
Schaub 2022	+	-	+	+	+	-
Tarutani 2022	+	-	+	+	+	-
Tian 2022	+	-	+	+	+	-
Gawlik-Kotelnicka 2022	+	-	+	+	+	-
Hashemi-Mohammadabad 2024	+	-	+	+	+	-
Khadem 2024	+	-	+	+	+	-
Lin 2024	+	-	+	+	+	-

Strodl 2024	+	-	+	+	+	-
Yang 2024	+	-	+	+	+	-
Wu 2022	+	-	+	+	+	-
Shamabadi 2024	+	-	+	+	+	-
Torkaman 2023	+	-	+	+	+	-
Kolahdooz 2024	+	-	+	+	-	-

Domains:
D1: Bias arising from the randomization process.
D2: Bias due to deviations from intended intervention.
D3: Bias due to missing outcome data.
D4: Bias in measurement of the outcome.
D5: Bias in selection of the reported result.

Judgement
X High
- Some concerns
+ Low

7.Definition of covariates

7.1. Sponsorship

We rated each arm of a study as being “sponsored” when it was indicated anywhere in the text that the study was funded/sponsored by the company which manufactured or marketed the drug in question, or if one or more of the authors were affiliated with the company in question, or if the data came from the documents provided by or obtained from the company website. We rated the sponsorship as "Unclear" if the authors only listed the names of the companies in question in their declaration of conflicts of interest. When one of the arms was “sponsored” thus defined, we regarded that the study was sponsored.

7.2. Baseline depressive severity

To make the mean baseline severity scores of participants comparable across studies that used different rating scales we used the transformation tables provided online at http://ids-qids.org/interpretation.html. We converted all values into HAMD17 scale. We included studies that reported the use of a HAMD scale without specifying which HAMD scale was used by assuming that such studies used the HAMD17 scale.

7.3. Risk of bias in the included studies

Two independent reviewers assessed RoB of each study for the primary outcome of efficacy in the following domains: sequence generation, allocation concealment, blinding of participant, blinding of therapist, blinding of assessor, selective reporting and attrition.

Then we summatively rated the overall RoB of each study as follows : Studies were classified as having low risk of bias if none of the domains above was rated as high risk of bias and three or less were rated as unclear risk; moderate if one was rated as high risk of bias or none was rated as high risk of bias but four or more were rated as unclear risk, and all other cases were assumed to pertain to high risk of bias.

7.4. Depressive severity classification

We classified each study into three different levels of depression severity: mild, moderate, and severe according to baseline depressive scores, The severity level were classified according to the appendix 9 cut of score.

7.5. Comorbidity classification

We rated each arm of a study as “with comorbidity “ if the study included population with another physical disease. If the study was clearly stated excluding physical diseases, we rated it as "without comorbidity". We rated the comorbidity as "unclear" if the study did not have enough information to judge.

8.Hierarchy of depressive scales

Hierarchy	Depressive scales	Abbreviation
1	Hamilton Depression Rating Scale	HAMD
2	Montgomery Asberg Depression Rating Scale	MADRS
3	Beck Depression Inventory	BDI
4	Zung Self-Rating Depression Scale	Z-SDS
5	Centre for Epidemiologic Studies Depression Scale	CES-D
6	Edinburgh Postnatal Depression Scale	EPDS
7	Geriatric Depression Scale	GDS
8	Depression Anxiety Stress Scale	DASS

Note: where different depression symptom severity rating scales were used, for the purpose of pooling results, we chose the single best available outcome measure according to a hierarchy based on psychometric properties and appropriateness for use.

9.Categorisation of depressive symptoms severity with cut-off scores

Scale	Cut-off point
Beck Depression Inventory-I	Cut-Off for Clinical Diagnosis of Depression:<br>0–9 = minimal/no depression<br>10–18 = mild/moderate depression<br>19–29 = moderate/severe depression<br>30–63 = severe depression
Beck Depression Inventory-II	0-13 = minimal<br>14-19 = mild (13-14*= mild)<br>20-28 = moderate<br>29-63 = severe
Center for Epidemiological Studies-Depression (CES-D)	CES-D 20:<br>16 = "significant" or "mild" depressive symptomatology<br>CES-D 10:<br>11 = recommended as cut off (Equivalent to experiencing 6 symptoms for most of the previous week or a majority of symptoms on 1 or 2 days.)
Clinical Diagnosis/Meets DSM Criteria/MDI	26 = moderate-severe depression<br>0-19 = no depression<br>20-24 = mild depression<br>25-29 = moderate depression<br>30-50 = severe depression
Depression Anxiety Stress Scales (DASS-2)	0-4 = Normal<br>5-6 = Mild<br>7-10 = Moderate<br>11-13 = Severe<br>14+ = Extremely severe<br>12 = recommended cut-point
Geriatric Depression Scale (GDS)	GDS-15:<br>5-9 = mild<br>10-15 = moderate to severe<br>GDS Long Form (30 items)<br>11-20 = mild<br>21-30 = moderate to severe
Hamilton Rating Scale For Depression (HAMD)	0-6 = no depression<br>7-17 = mild depression<br>18-24 = moderate depression<br>24+ = severe depression
The self-reported Montgomery-Åsberg depression rating scal(MADRS-S)	13-19 = Mild<br>20+ = Moderate to Severe
Montgomery-Asberg Depression Rating Scale (MADRS)	7-19 = Mild<br>20-34 = Moderate<br>35-60 = Severe
Zung SDS (Zung Self Assessment Depression Scale)	50 = mild<br>60 = moderate<br>70 = severe

References: Apaydin, E. A., A. R. Maher, R. Shanman, M. S. Booth, J. N. V. Miles, M. E. Sorbero and S. Hempel (2016). "A systematic review of St. John's wort for major depressive disorder." Systematic Reviews **5**(1): 148.

10.Results from network meta-analyses

- ✓ Below we present the network diagram, the forest plot of the effect estimate for each active intervention versus placebo, and the P rank of all comparisons for each of the primary and four secondary outcomes.
- ✓ The following abbreviations are used in the figures and tables throughout the documents: ADT: Antidepressant; Ca: Calcium ; DHA: Docosahexaenoic Acid ; E Amoenum : Echium amoenum; EPA :Eicosapentaenoic acid; Fe : Ferrum; Mg: Magnesium; PEA: Palmitoylethanolamide; R rosea : Rhodiola rosea; SAMe: S-Adenosyl Methionine ; SJW: St. John's wort ;Vitamin B1: Thiamine ; Vitamin B6: Pyridoxine; Vitamin B7: Biotin; Vitamin B: Vitamin B complex; Vitamin B12 : Cobalamin; Vitamin C: Ascorbic acid; Vitamin D: Cholecalciferol; 5HTP: 5-Hydroxytryptophan

10.1. Network plot of each outcome

- ✓ Lines between nodes represent direct comparisons between trials, and circle size is proportional to the size of the population that received each treatment. Line thickness is proportional to the number of studies providing data to the comparison.

10.1.A. Network plot for changes in depressive symptoms

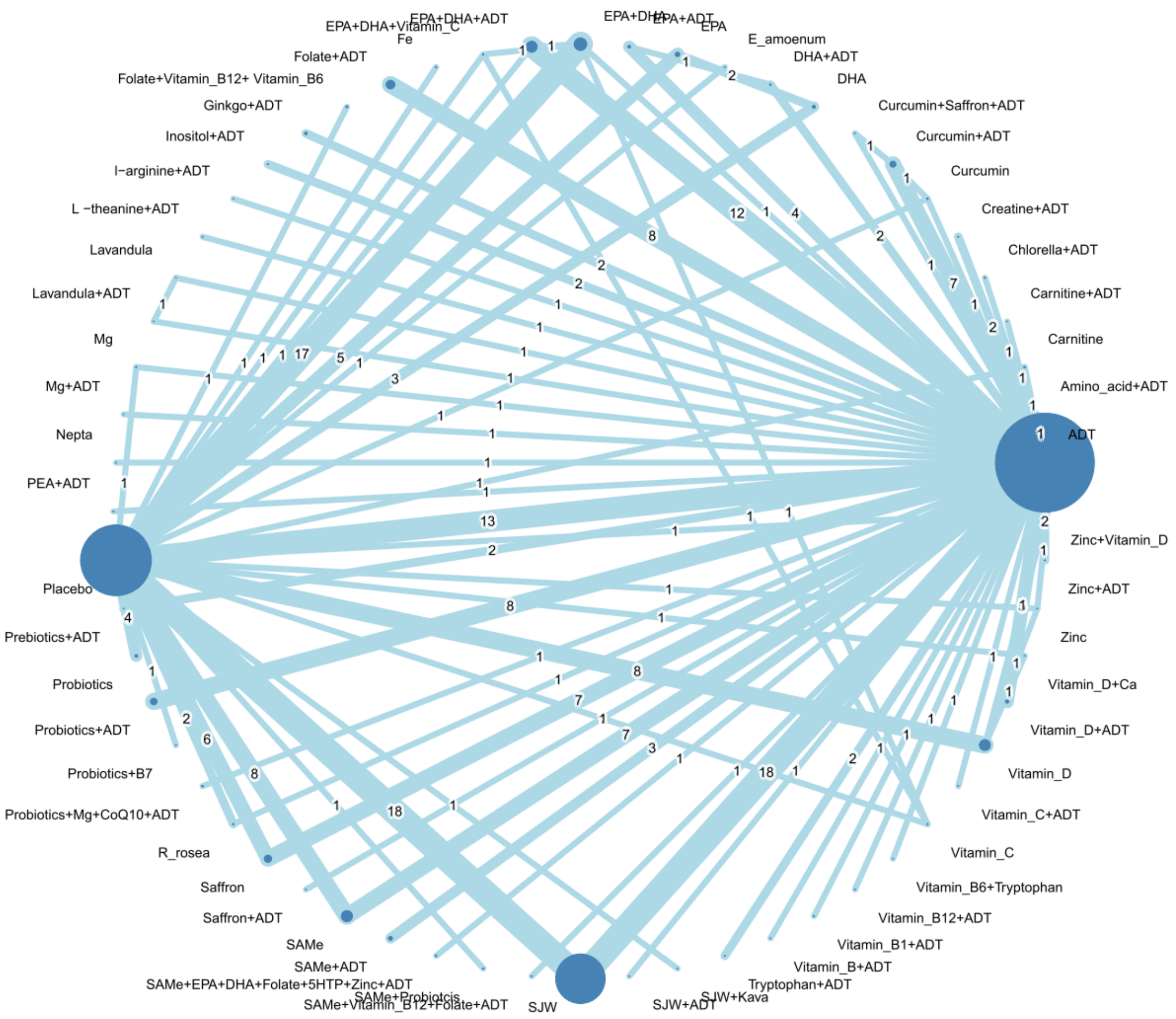

10.1.B. Network plot for response rate

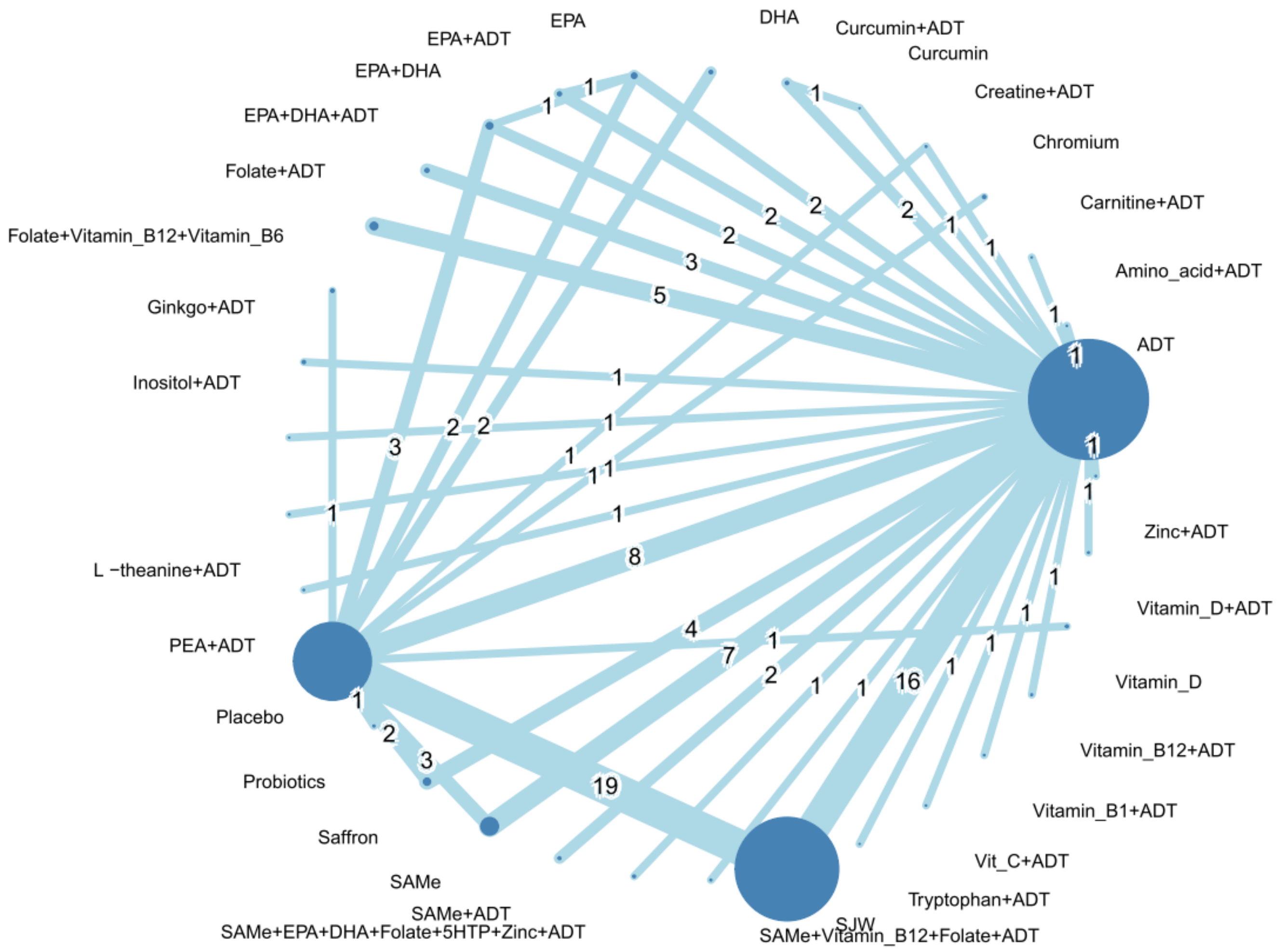

10.1.C. Network plot for remission rate

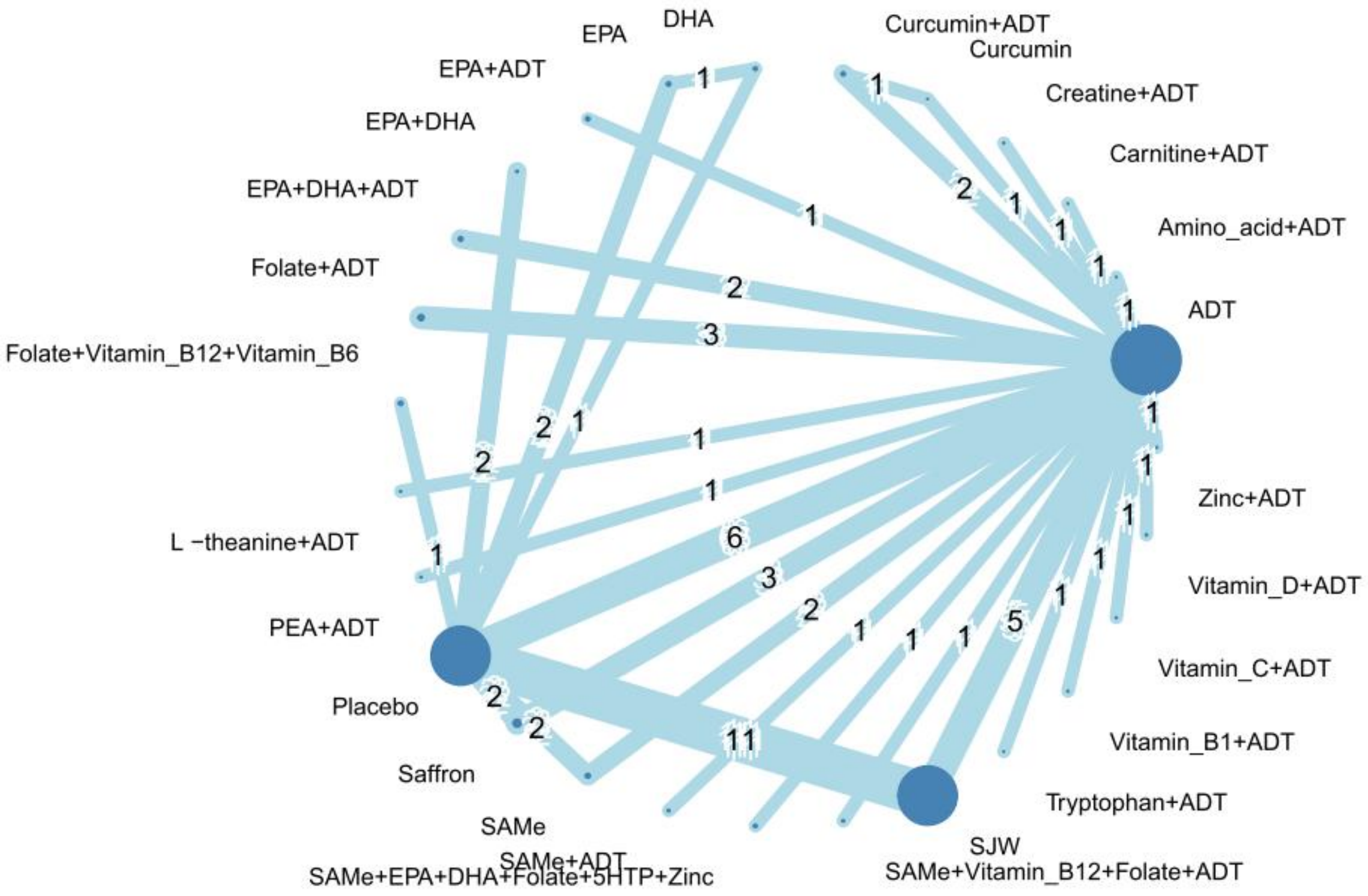

10.1.D. Network plot for change in anxiety symptoms

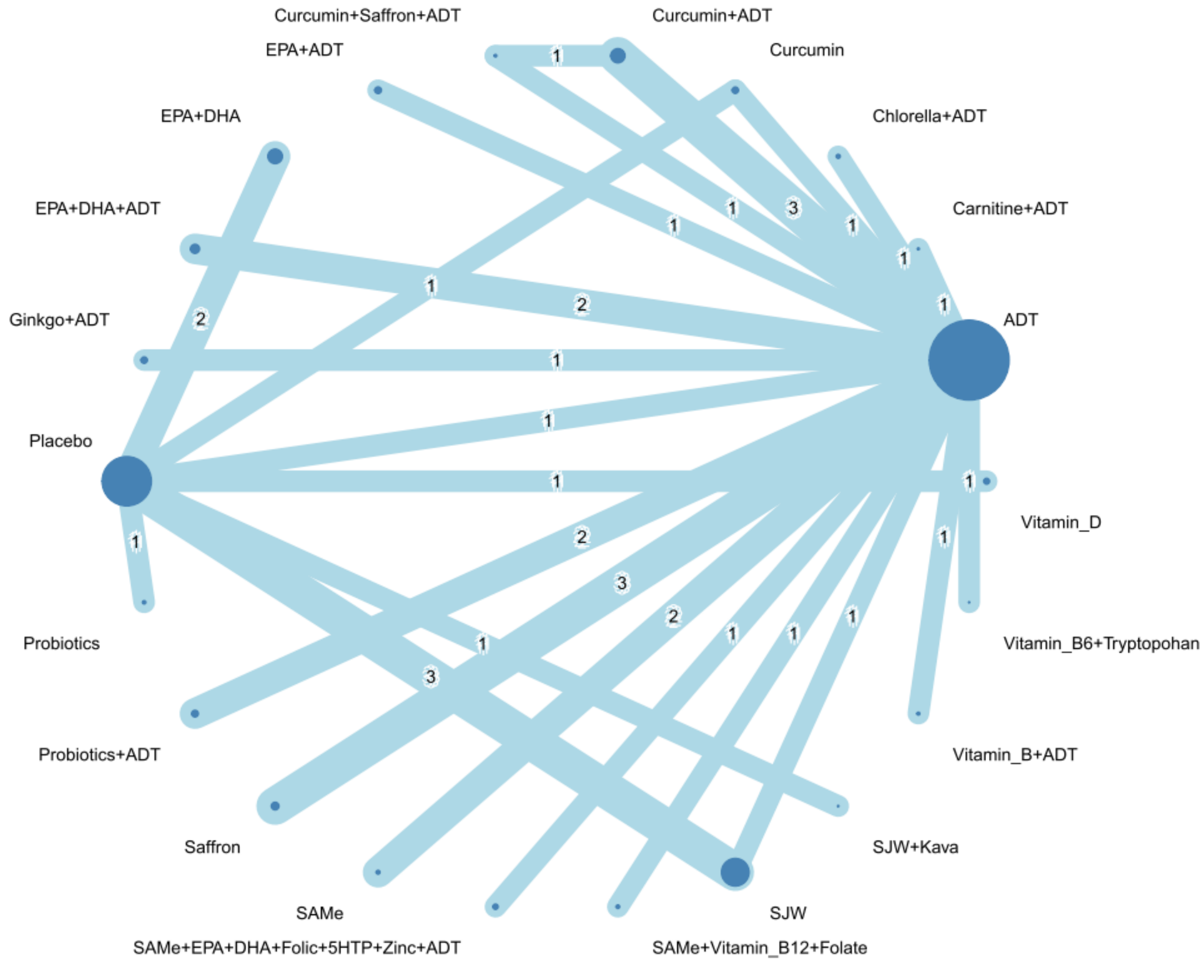

10.1.E. Network plot for all cause discontinuation rate

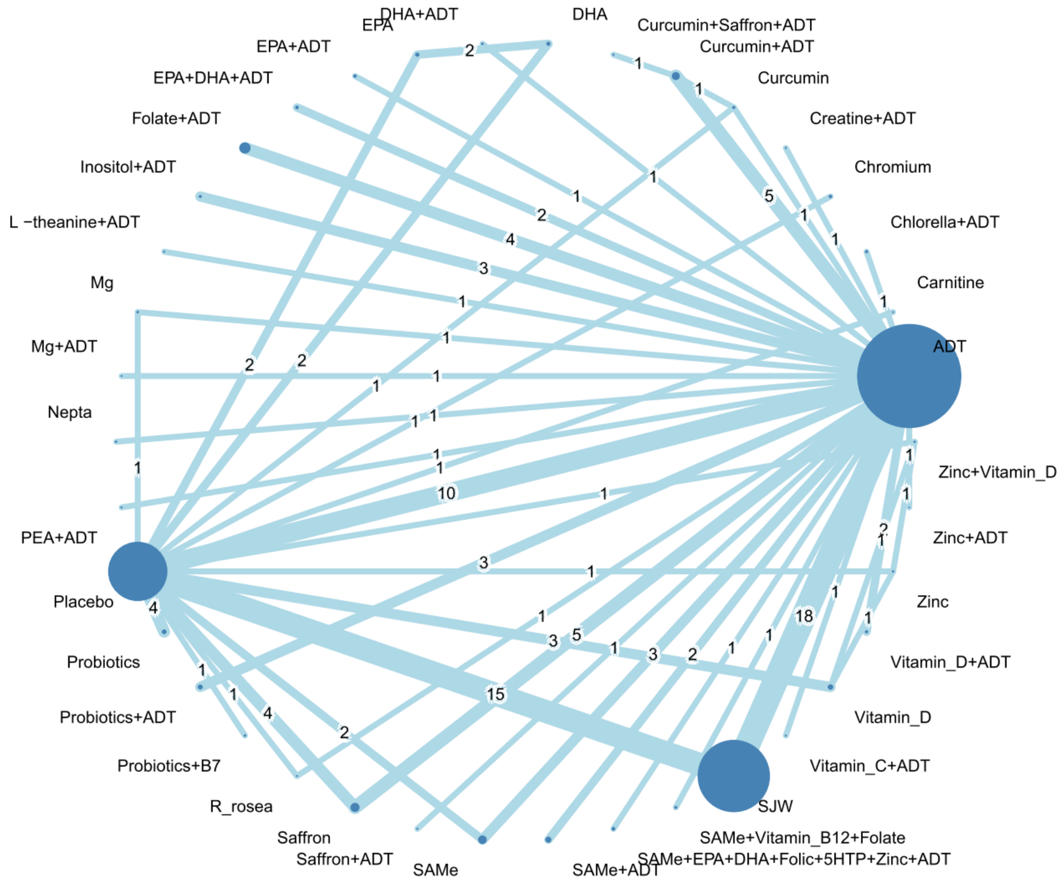

10.1.F. Network plot for adverse events

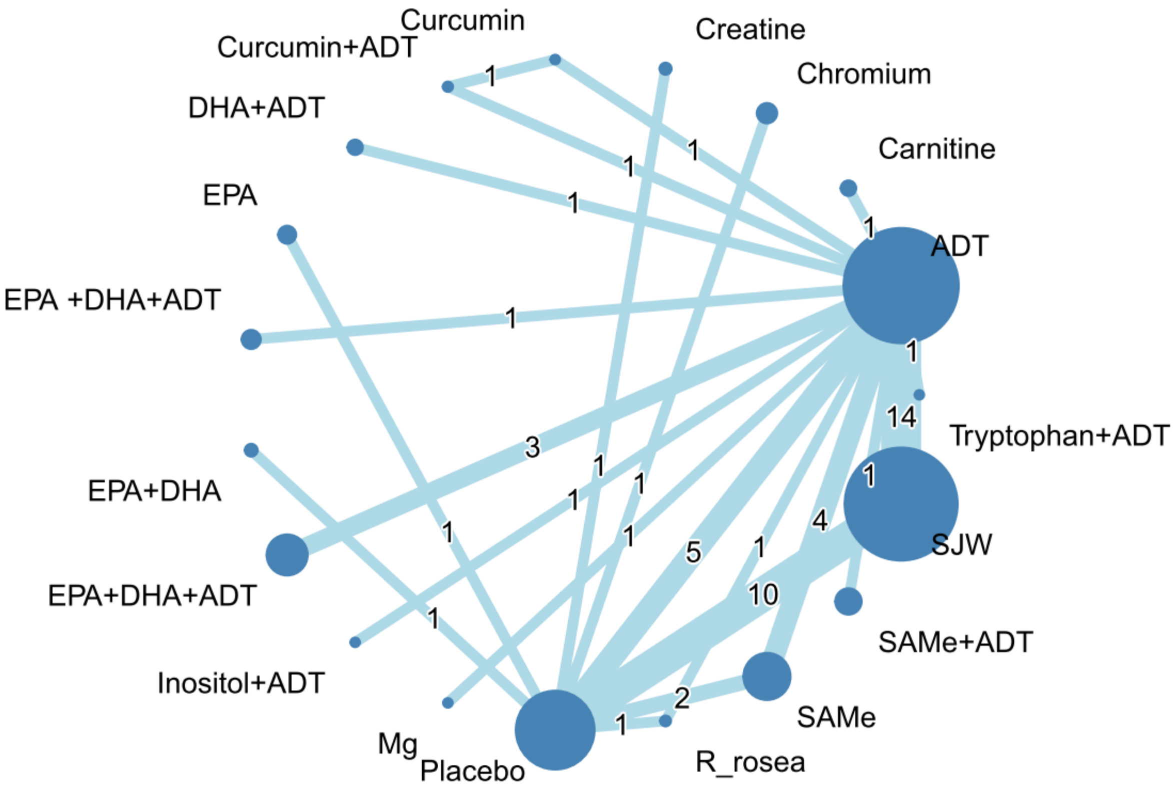

10.2. Forest plot of each outcome

10.2.A. Forest plot for changes in depressive symptoms

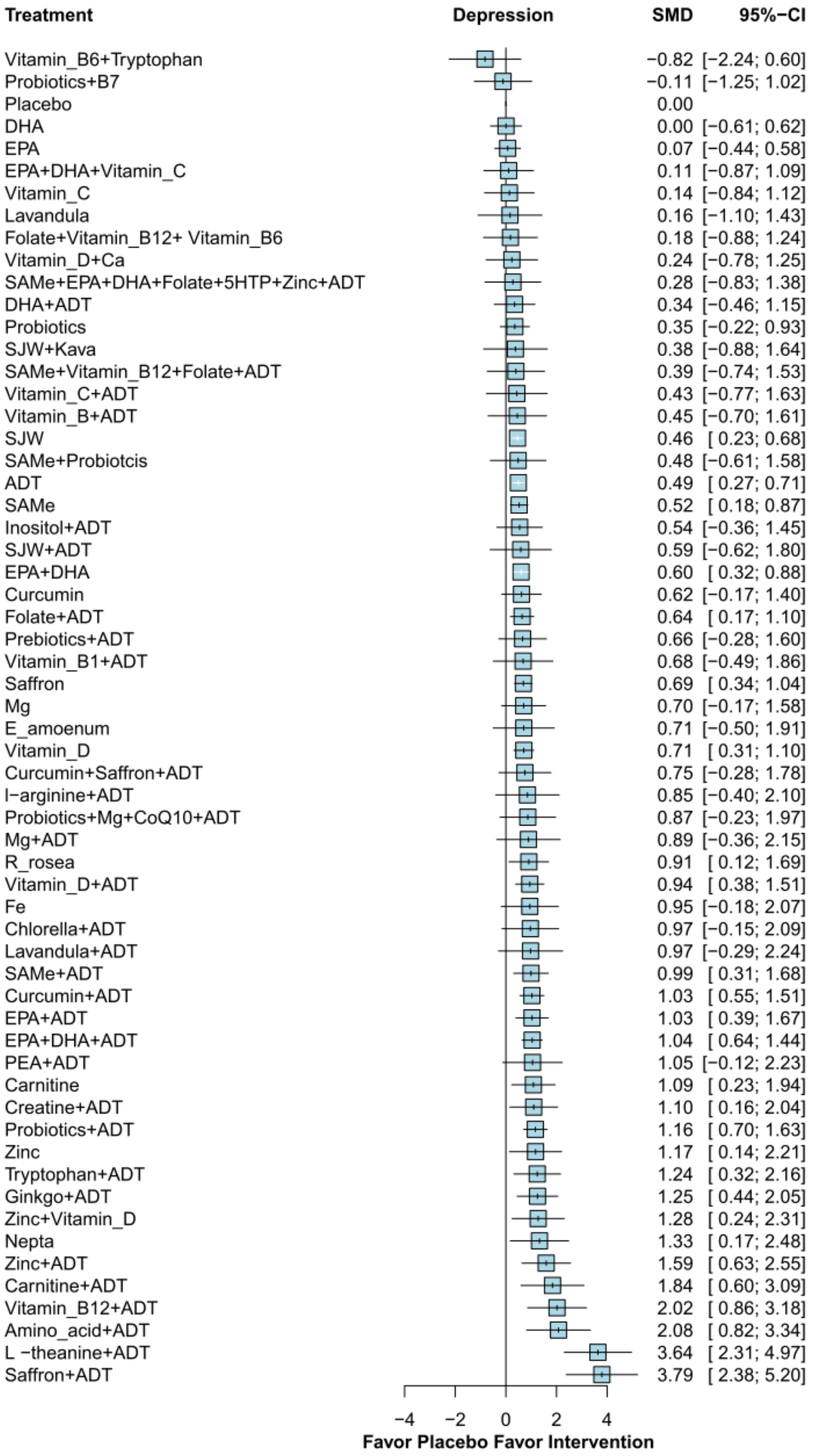

10.2.B. Forest plot for response rate

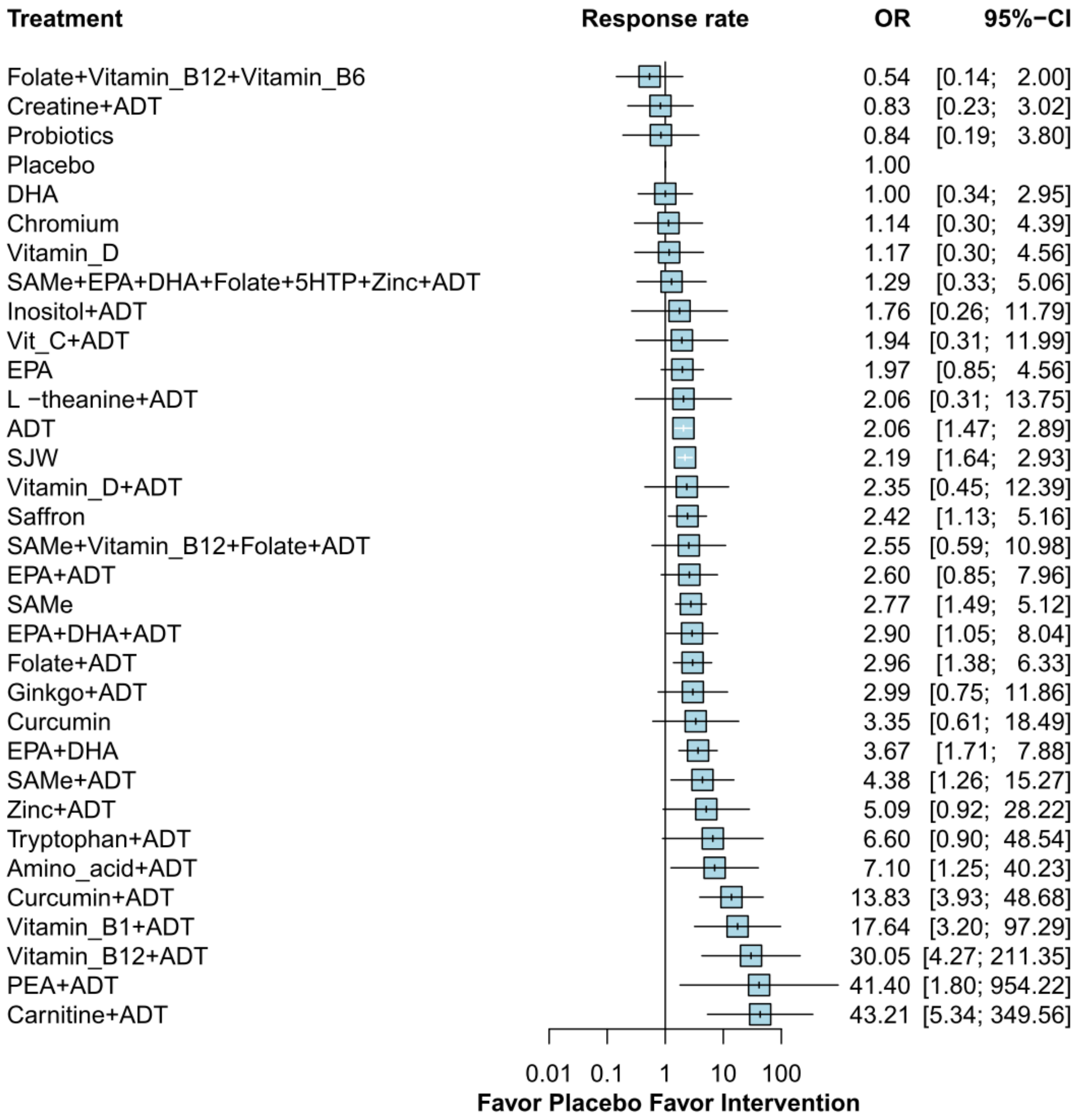

10.2.C. Forest plot for remission rate

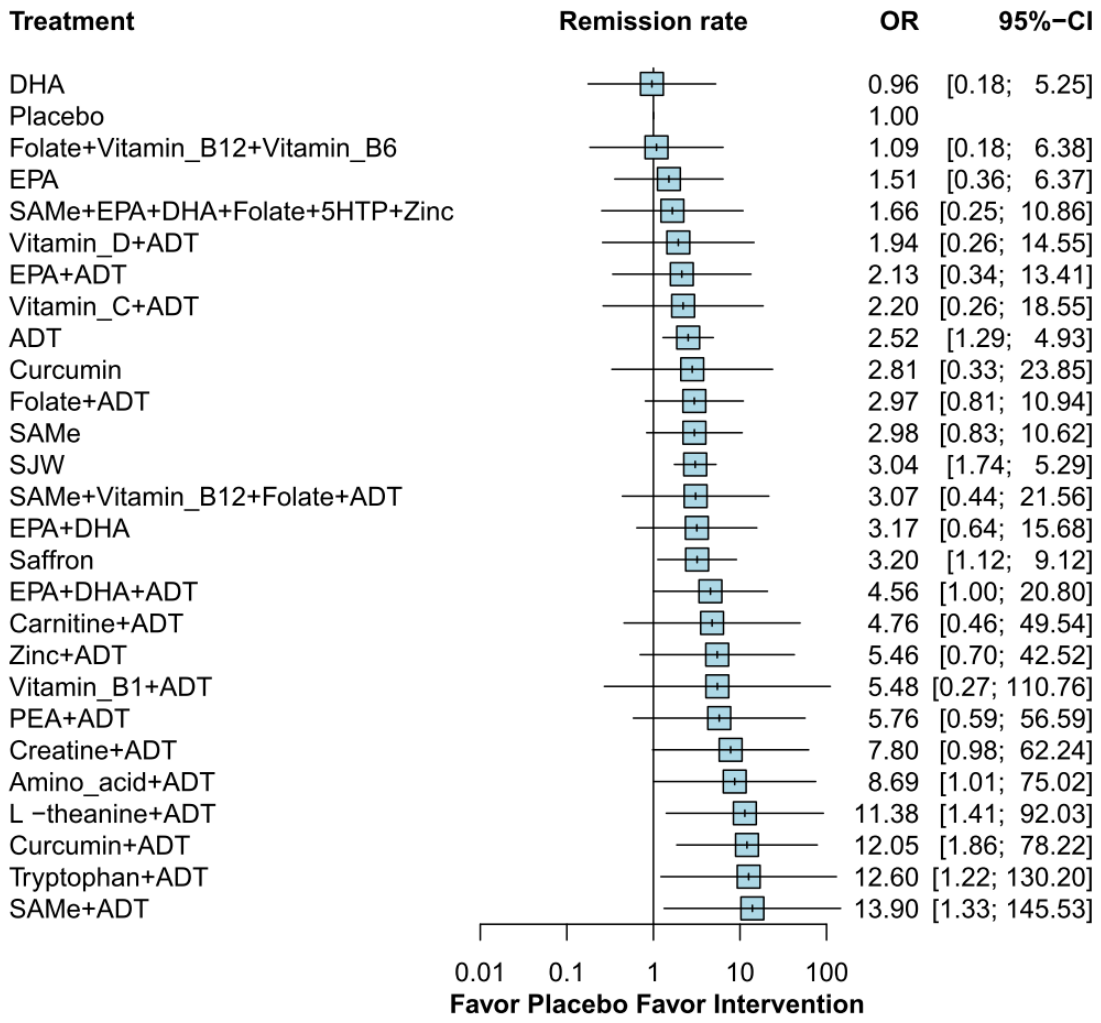

10.2.D. Forest plot for change in anxiety symptoms

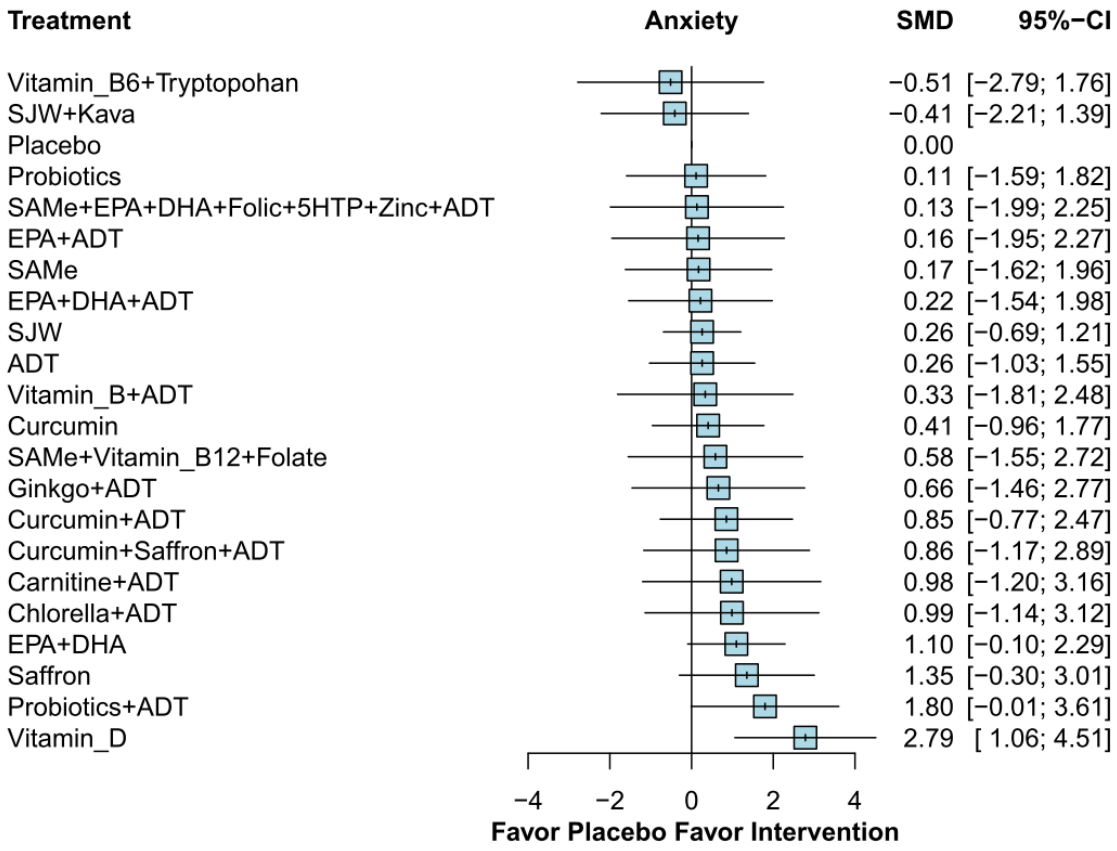

10.2.E. Forest plot for all cause discontinuation

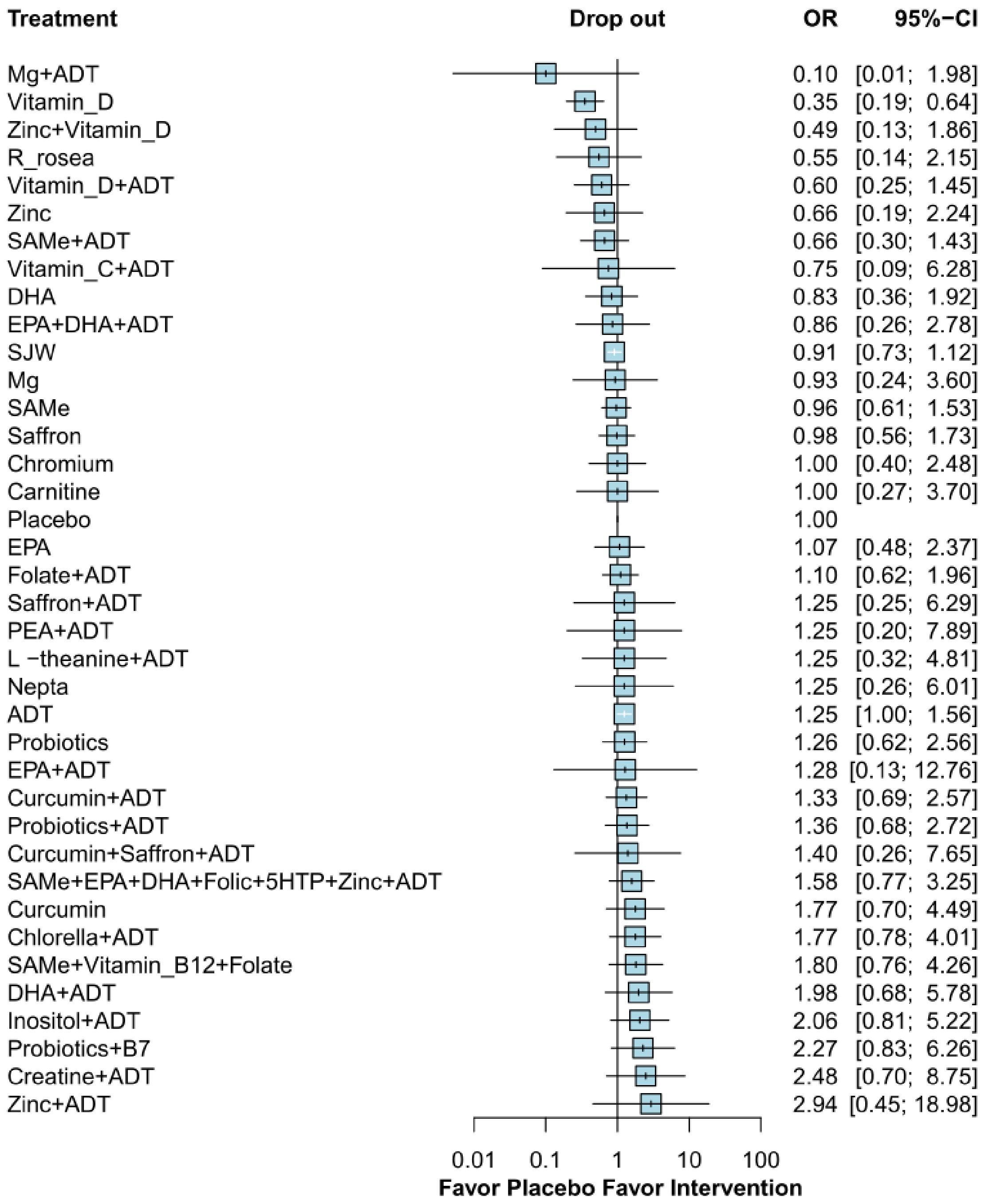

10.2.F. Forest plot for adverse event

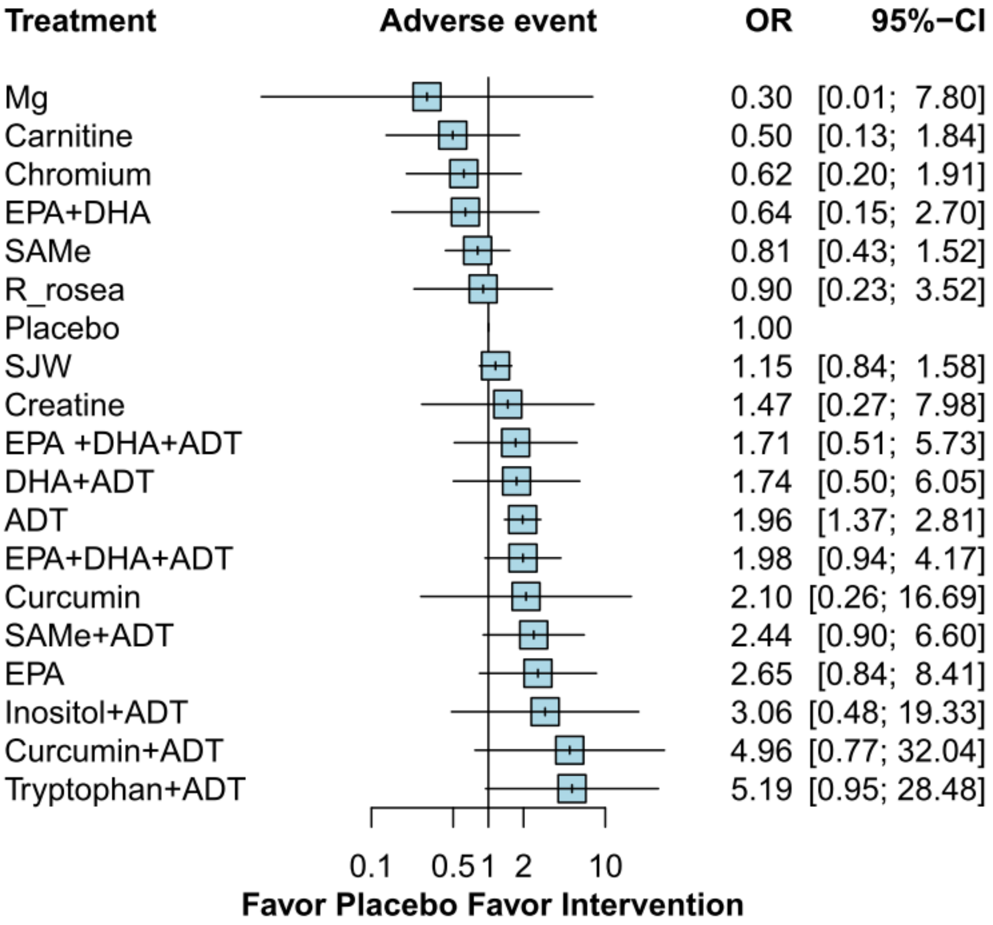

10.3. Treatment ranking and P score for each efficacy outcome

10.3.A. Treatment ranking and P score for changes in depressive symptoms

Ranking	Nutraceuticals	P-score
1	Saffron+ADT	0.9907
2	L-theanine+ADT	0.9881
3	Amino_acid+ADT	0.9016
4	Vitamin_B12+ADT	0.8998
5	Carnitine+ADT	0.8631
6	Zinc+ADT	0.8317
7	Ginkgo+ADT	0.7355
8	Nepta	0.7335
9	Probiotics+ADT	0.726
10	Zinc+Vitamin_D	0.7245
11	Tryptophan+ADT	0.7219
12	Zinc	0.6846
13	EPA+DHA+ADT	0.6685
14	Carnitine	0.6633
15	Creatine+ADT	0.6631
16	Curcumin+ADT	0.6608
17	EPA+ADT	0.6537
18	SAMe+ADT	0.6303
19	PEA+ADT	0.6282
20	Vitamin_D+ADT	0.6124
21	Chlorella+ADT	0.5949
22	Lavandula+ADT	0.5923
23	Fe	0.5845
24	R_rosea	0.5818
25	Mg+ADT	0.5571
26	Probiotics+Mg+CoQ10+ADT	0.5509
27	L-arginine+ADT	0.5406
28	Curcumin+Saffron+ADT	0.4991
29	Vitamin_D	0.4825
30	E_amoenum	0.4787
31	Mg	0.4777
32	Saffron	0.4733
33	Vitamin_B1+ADT	0.467
34	Prebiotics+ADT	0.4556
35	Folate+ADT	0.44
36	Curcumin	0.4323

37	SJW+ADT	0.4256
38	EPA+DHA	0.417
39	Inositol+ADT	0.3972
40	SAMe+Probiotics	0.379
41	SAMe	0.3697
42	Vitamin_B+ADT	0.3651
43	Vitamin_C+ADT	0.359
44	ADT	0.3433
45	SJW+Kava	0.3414
46	SAMe+Vitamin_B12+Folate+ADT	0.3397
47	SJW	0.3253
48	DHA+ADT	0.2959
49	Probiotics	0.2899
50	SAMe+EPA+DHA+Folate+5HTP+Zinc+ADT	0.2893
51	Vitamin_D+Ca	0.2685
52	Lavandula	0.2571
53	Folate+Vitamin_B12+Vitamin_B6	0.251
54	Vitamin_C	0.2279
55	EPA+DHA+Vitamin_C	0.2183
56	EPA	0.1651
57	Probiotics+B7	0.1606
58	DHA	0.1498
59	Placebo	0.1251
60	Vitamin_B6+Tryptophan	0.0495

10.3.B. Treatment ranking and P score for response rate

Ranking	Nutraceuticals	P-score
1	Carnitine+ADT	0.9442
2	Vitamin_B12+ADT	0.9229
3	PEA+ADT	0.9063
4	Vitamin_B1+ADT	0.8821
5	Curcumin+ADT	0.8706
6	Amino_acid+ADT	0.7436
7	Tryptophan+ADT	0.7147
8	Zinc+ADT	0.6711
9	SAMe+ADT	0.6565
10	EPA+DHA	0.6285
11	Curcumin	0.56
12	Folate+ADT	0.5527
13	EPA+DHA+ADT	0.5367
14	Ginkgo+ADT	0.5362
15	SAMe	0.5306
16	EPA+ADT	0.4962
17	SAMe+Vitamin_B12+Folate+ADT	0.4839
18	Saffron	0.4728
19	Vitamin_D+ADT	0.4578
20	SJW	0.4305
21	L-theanine+ADT	0.4199
22	Vit_C+ADT	0.4019
23	ADT	0.3947
24	EPA	0.3922
25	Inositol+ADT	0.3771
26	SAMe+EPA+DHA+Folate+5HTP+Zinc+ADT	0.2688
27	Vitamin_D	0.2457
28	Chromium	0.2387
29	DHA	0.1906
30	Probiotics	0.1744
31	Placebo	0.1595
32	Creatine+ADT	0.1566
33	Folate+Vitamin_B12+Vitamin_B6	0.0818

10.3.C. Treatment ranking and P score for remission ate

Ranking	Nutraceuticals	P-score
1	Curcumin+ADT	0.8123
2	SAMe+ADT	0.8092
3	Tryptophan+ADT	0.7927
4	L-theanine+ADT	0.7871
5	Amino_acid+ADT	0.7291
6	Creatine+ADT	0.709
7	PEA+ADT	0.6294
8	Zinc+ADT	0.624
9	Vitamin_B1+ADT	0.5976
10	EPA+DHA+ADT	0.5921
11	Carnitine+ADT	0.5812
12	Saffron	0.4872
13	EPA+DHA	0.483
14	SJW	0.4725
15	SAMe+Vitamin_B12+Folate+ADT	0.4701
16	SAMe	0.462
17	Folate+ADT	0.4597
18	Curcumin	0.4439
19	ADT	0.3928
20	Vitamin_C+ADT	0.3822
21	EPA+ADT	0.3654
22	Vitamin_D+ADT	0.3453
23	SAMe+EPA+DHA+Folate+5HTP+Zinc	0.2994
24	EPA	0.2696
25	Folate+Vitamin_B12+Vitamin_B6	0.2049
26	DHA	0.1739
27	Placebo	0.1246

10.3.D. Treatment ranking and P score for change in anxiety symptoms

Ranking	**Nutraceuticals**	P-score
1	Vitamin_D	0.9496
2	Probiotics+ADT	0.858
3	Saffron	0.7684
4	EPA+DHA	0.6758
5	Chlorella+ADT	0.6314
6	Carnitine+ADT	0.6271
7	Curcumin+ADT	0.6132
8	Curcumin+Saffron+ADT	0.596
9	Ginkgo+ADT	0.5297
10	SAMe+Vitamin_B12+Folate	0.5067
11	Curcumin	0.4505
12	Vitamin_B+ADT	0.4286
13	SJW	0.399
14	EPA+DHA+ADT	0.3792
15	ADT	0.3776
16	EPA+ADT	0.3735
17	Probiotics	0.3698
18	SAMe+EPA+DHA+Folic+5HTP+Zinc+ADT	0.3649
19	SAMe	0.3645
20	Placebo	0.2966
21	SJW+Kava	0.2361
22	Vitamin_B6+Tryptophan	0.2037

10.4. Summary table for Treatment ranking of efficacy outcome

Ranking	Depressive symptom	Response	Remission	Anxiety symptom
1	Saffron+ADT	Carnitine+ADT	Curcumin+ADT	Vitamin_D
2	L-theanine+ADT	Vitamin_B12+ADT	SAMe+ADT	Probiotics+ADT
3	Amino_acid+ADT	PEA+ADT	Tryptophan+ADT	Saffron
4	Vitamin_B12+ADT	Vitamin_B1+ADT	L-theanine+ADT	EPA+DHA
5	Carnitine+ADT	Curcumin+ADT	Amino_acid+ADT	Chlorella+ADT
6	Zinc+ADT	Amino_acid+ADT	Creatine+ADT	Carnitine+ADT
7	Ginkgo+ADT	Tryptophan+ADT	PEA+ADT	Curcumin+ADT
8	Nepta	Zinc+ADT	Zinc+ADT	Curcumin+Saffron+ADT
9	Probiotics+ADT	SAMe+ADT	Vitamin_B1+ADT	Ginkgo+ADT
10	Zinc+Vitamin_D	EPA+DHA	EPA+DHA+ADT	SAMe+Vitamin_B12+Folate
11	Tryptophan+ADT	Curcumin	Carnitine+ADT	Curcumin
12	Zinc	Folate+ADT	Saffron	Vitamin_B+ADT
13	EPA+DHA+ADT	EPA+DHA+ADT	EPA+DHA	SJW
14	Carnitine	Ginkgo+ADT	SJW	EPA+DHA+ADT
15	Creatine+ADT	SAMe	SAMe+Vitamin_B12+Folate+ADT	ADT
16	Curcumin+ADT	EPA+ADT	SAMe	EPA+ADT
17	EPA+ADT	SAMe+Vitamin_B12+Folate+ADT	Folate+ADT	Probiotics
18	SAMe+ADT	Saffron	Curcumin	SAMe+EPA+DHA+Folic+5HTP+Zinc+ADT
19	PEA+ADT	Vitamin_D+ADT	ADT	SAMe
20	Vitamin_D+ADT	SJW	Vitamin_C+ADT	Placebo
21	Chlorella+ADT	L-theanine+ADT	EPA+ADT	SJW+Kava
22	Lavandula+ADT	Vit_C+ADT	Vitamin_D+ADT	Vitamin_B6+Tryptophan
23	Fe	ADT	SAMe+EPA+DHA+Folate+5HTP+Zinc	
24	R_rosea	EPA	EPA	
25	Mg+ADT	Inositol+ADT	Folate+Vitamin_B12+Vitamin_B6	
26	Probiotics+Mg+CoQ10+ADT	SAMe+EPA+DHA+Folate+5HTP+Zinc+ADT	DHA	
27	L-arginine+ADT	Vitamin_D	Placebo	
28	Curcumin+Saffron+ADT	Chromium		
29	Vitamin_D	DHA		
30	E_amoenum	Probiotics		
31	Mg	Placebo		
32	Saffron	Creatine+ADT		

33	Vitamin_B1+ADT	Folate+Vitamin_B12+ Vitamin_B6
34	Prebiotics+ADT	
35	Folate+ADT	
36	Curcumin	
37	SJW+ADT	
38	EPA+DHA	
39	Inositol+ADT	
40	SAMe+Probiotics	
41	SAMe	
42	Vitamin_B+ADT	
43	Vitamin_C+ADT	
44	ADT	
45	SJW+Kava	
46	SAMe+Vitamin_B12+ Folate+ADT	
47	SJW	
48	DHA+ADT	
49	Probiotics	
50	SAMe+EPA+DHA+Folate+5HTP+Zinc+ADT	
51	Vitamin_D+Ca	
52	Lavandula	
53	Folate+Vitamin_B12+ Vitamin_B6	
54	Vitamin_C	
55	EPA+DHA+Vitamin_C	
56	EPA	
57	Probiotics+B7	
58	DHA	
59	Placebo	
60	Vitamin_B6+Tryptophan	
61		

11.Network result of subgroups with different baseline depressive severity

- ✓ Below we present the network diagram, the forest plot of the effect estimate for each active intervention versus placebo, and the P rank of all comparisons for each of the primary and four secondary outcomes.
- ✓ The following abbreviations are used in the figures and tables throughout the documents: ADT: Antidepressant; Ca: Calcium ; DHA: Docosahexaenoic Acid ; E Amoenum : Echium amoenum; EPA :Eicosapentaenoic acid; Fe : Ferrum; Mg: Magnesium; PEA: Palmitoylethanolamide; R rosea : Rhodiola rosea; SAMe: S-Adenosyl Methionine ; SJW: St. John's wort ;Vitamin B1: Thiamine ; Vitamin B6: Pyridoxine; Vitamin B7: Biotin; Vitamin B: Vitamin B complex; Vitamin B12 : Cobalamin; Vitamin C: Ascorbic acid; Vitamin D: Cholecalciferol; 5HTP: 5-Hydroxytryptophan

11.1. Network plot for subgroups with different baseline depressive severity

- ✓ Lines between nodes represent direct comparisons between trials, and circle size is proportional to the size of the population that received each treatment. Line thickness is proportional to the number of studies providing data to the comparison.

11.1.A. Network plot for mild depression

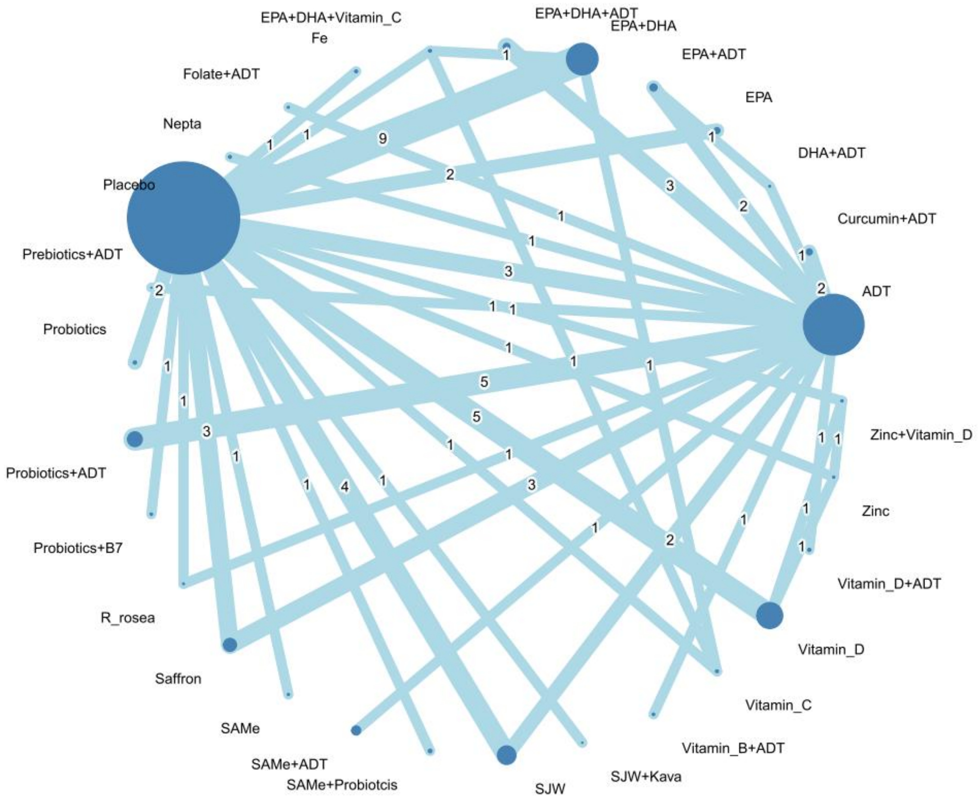

11.1.B. Network plot for moderate depression

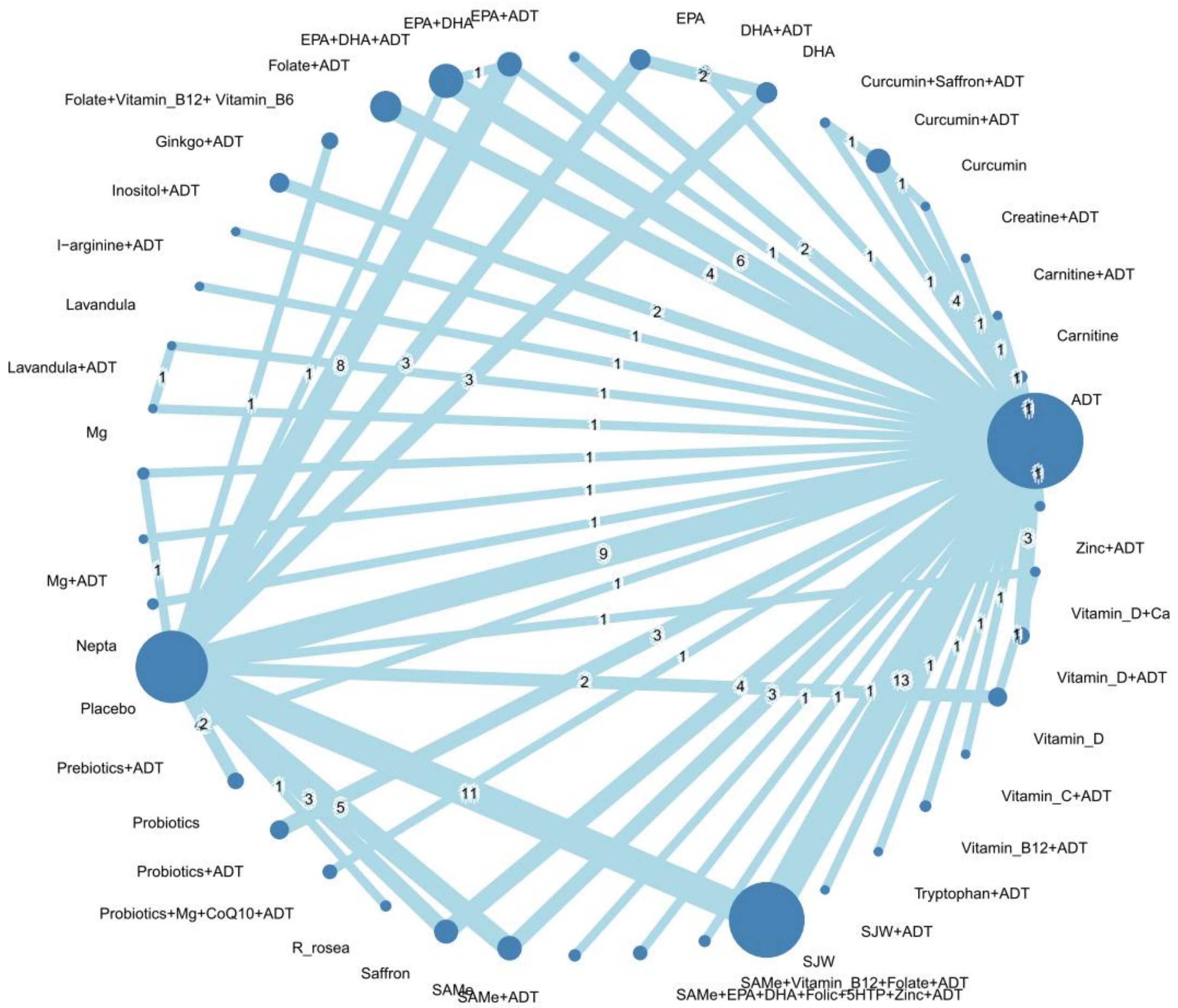

11.1.C. Network plot for severe depression

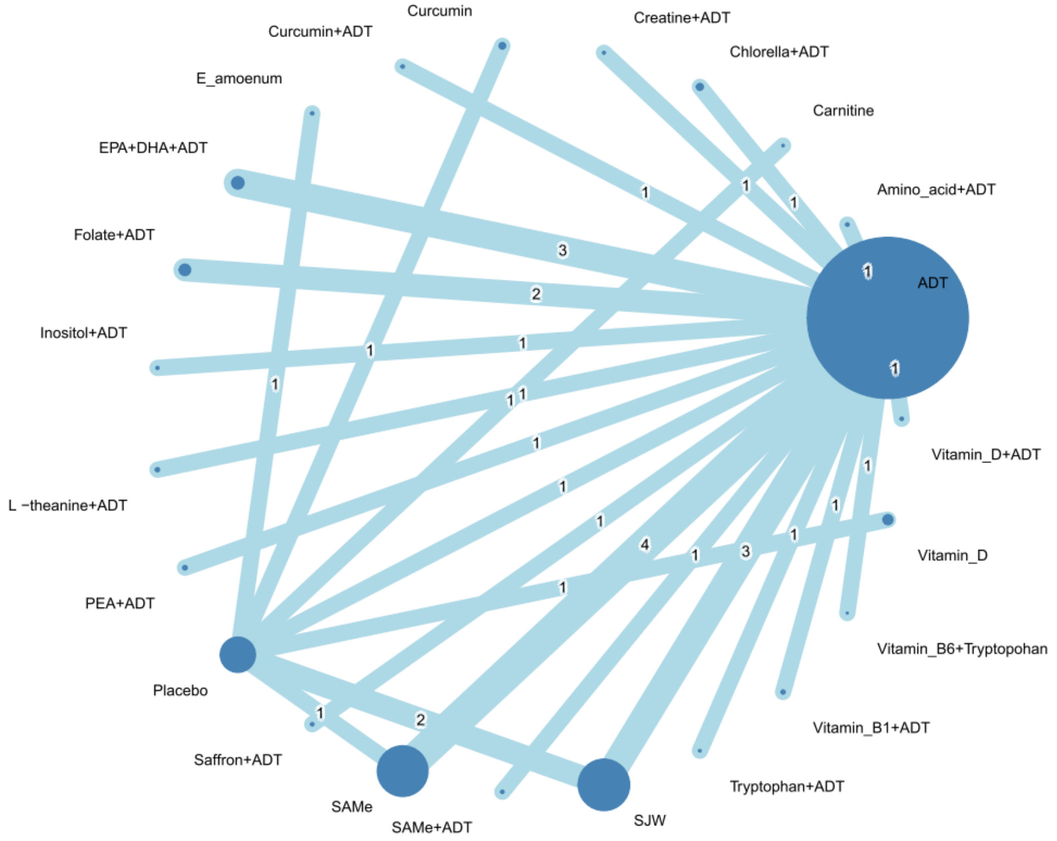

11.2. Forest plot for subgroups with different baseline depressive severity
11.2.A. Forest plot for mild depression

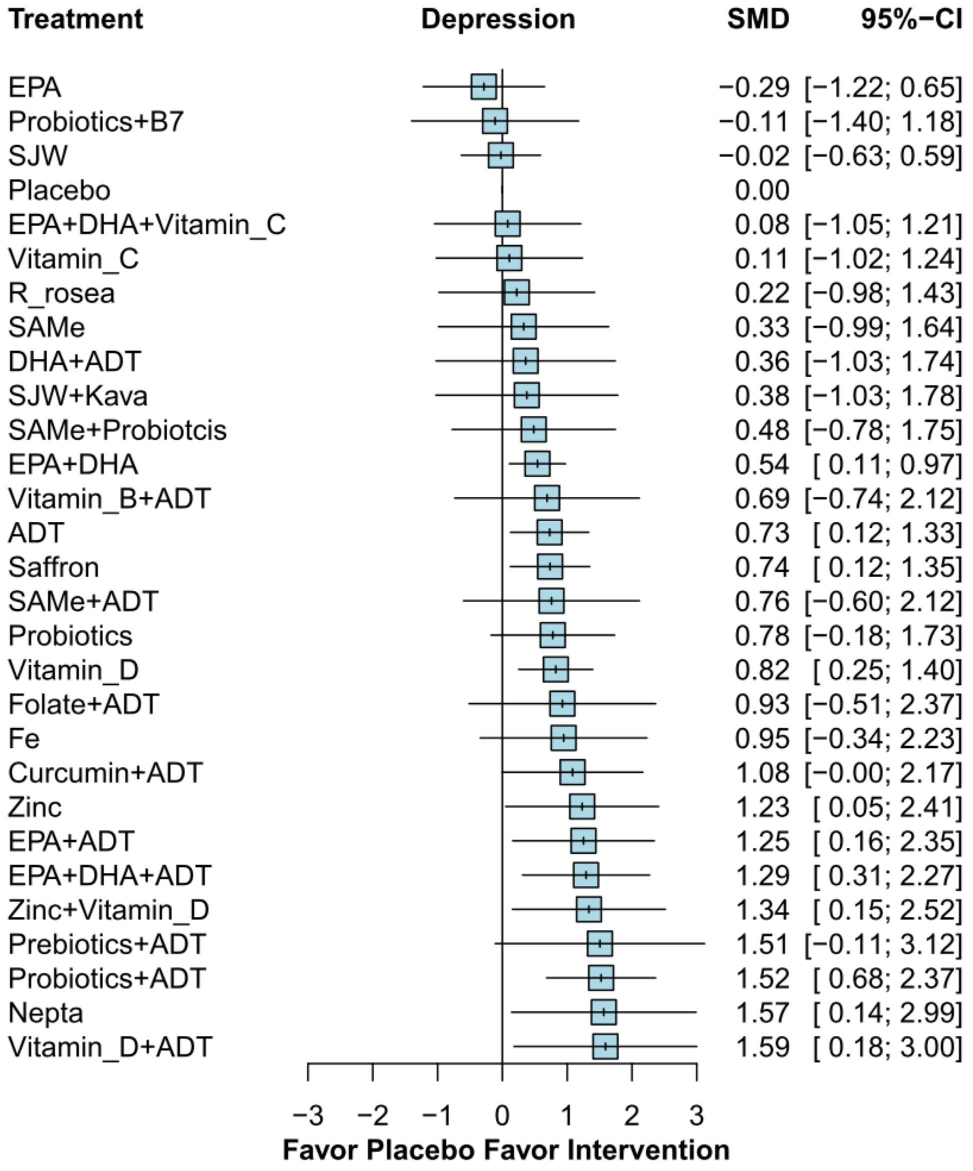

11.2.B. Forest plot for moderate depression

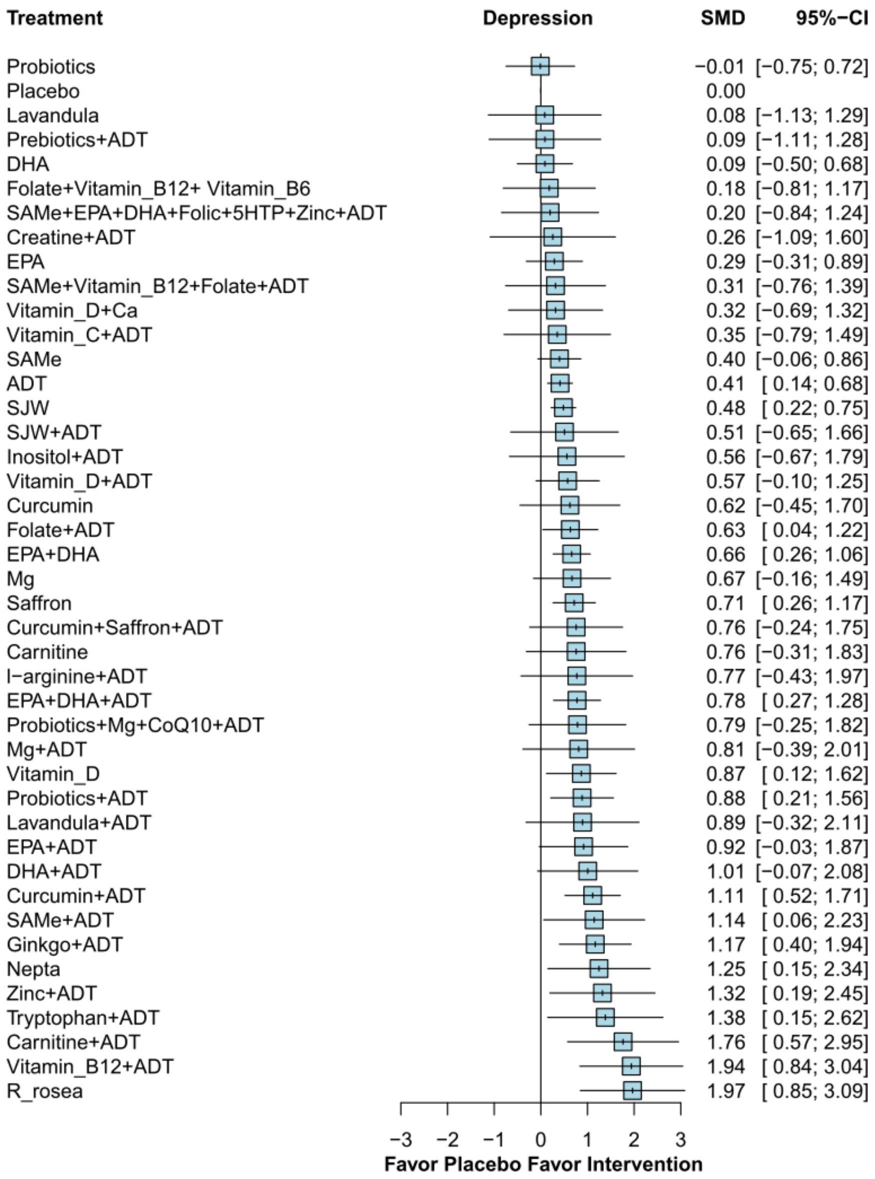

11.2.C. Forest plot for severe depression

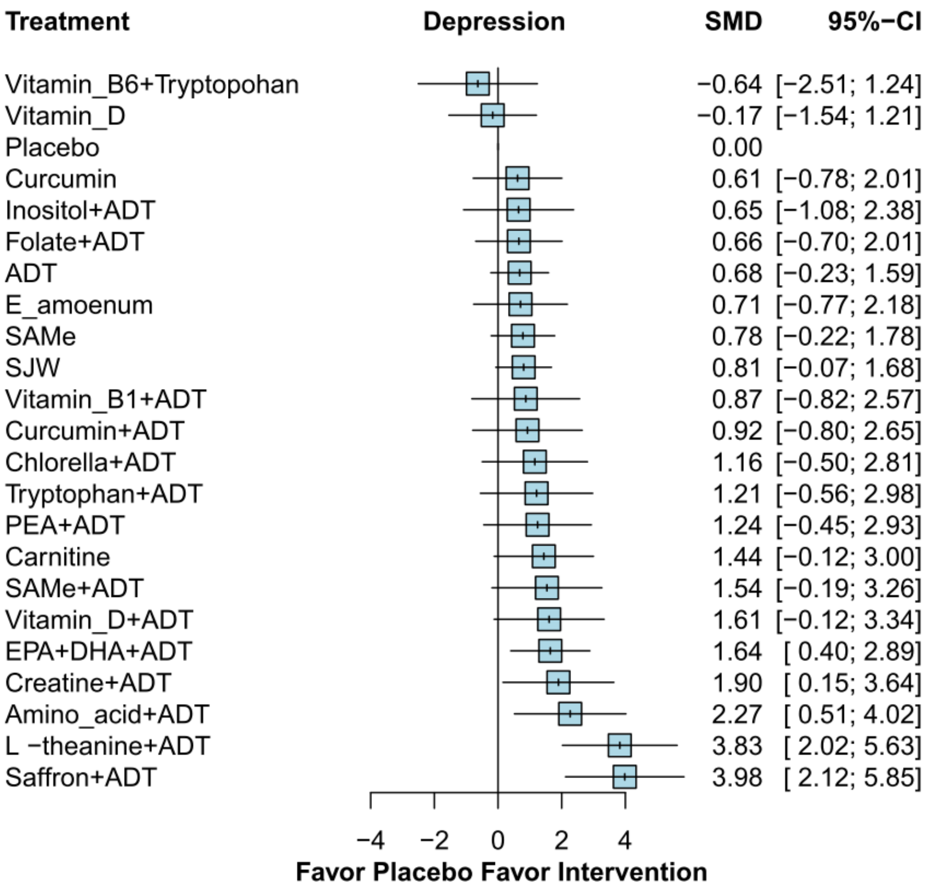

11.3. Treatment ranking and P score for subgroups with different baseline depressive severity

11.3.A. Treatment ranking and P score for mild depression

Ranking	Nutraceuticals	P-score
1	Probiotics+ADT	0.8394
2	Vitamin_D+ADT	0.8123
3	Nepta	0.8039
4	Prebiotics+ADT	0.7709
5	EPA+DHA+ADT	0.7483
6	Zinc+Vitamin_D	0.7445
7	EPA+ADT	0.7272
8	Zinc	0.7075
9	Curcumin+ADT	0.6587
10	Fe	0.5949
11	Folate+ADT	0.5807
12	Vitamin_D	0.5587
13	Probiotics	0.5334
14	SAMe+ADT	0.5149
15	Saffron	0.5115
16	ADT	0.5011
17	Vitamin_B+ADT	0.4872
18	EPA+DHA	0.421
19	SAMe+Probiotcis	0.4109
20	SJW+Kava	0.3739
21	DHA+ADT	0.3544
22	SAMe	0.3522
23	R_rosea	0.3011
24	Vitamin_C	0.2607
25	EPA+DHA+Vitamin_C	0.2513
26	Probiotics+B7	0.2005
27	SJW	0.1789
28	Placebo	0.1751
29	EPA	0.1248

11.3.B. Treatment ranking and P score for moderate depression

Ranking	Nutraceuticals	P-score
1	Vitamin_B12+ADT	0.9276
2	R_rosea	0.9267
3	Carnitine+ADT	0.8884
4	Tryptophan+ADT	0.7833
5	Zinc+ADT	0.773
6	Nepta	0.7498
7	Ginkgo+ADT	0.7483
8	Curcumin+ADT	0.7408
9	SAMe+ADT	0.711
10	DHA+ADT	0.6529
11	Probiotics+ADT	0.6211
12	EPA+ADT	0.6201
13	Vitamin_D	0.6068
14	Lavandula+ADT	0.5968
15	EPA+DHA+ADT	0.5681
16	Mg+ADT	0.5582
17	Probiotics+Mg+CoQ10+ADT	0.5517
18	L-arginine+ADT	0.5402
19	Curcumin+Saffron+ADT	0.5378
20	Carnitine	0.5367
21	Saffron	0.5319
22	EPA+DHA	0.499
23	Mg	0.4958
24	Folate+ADT	0.4774
25	Curcumin	0.4696
26	Vitamin_D+ADT	0.442
27	Inositol+ADT	0.4411
28	SJW+ADT	0.4151
29	SJW	0.3796
30	Vitamin_C+ADT	0.3431
31	SAMe	0.3332
32	ADT	0.3247
33	SAMe+Vitamin_B12+Folate+ADT	0.3216
34	Vitamin_D+Ca	0.321
35	Creatine+ADT	0.3148
36	EPA	0.284
37	SAMe+EPA+DHA+Folic+5HTP+Zinc+ADT	0.2672

38	Folate+Vitamin_B12+Vitamin_B6	0.2603
39	Prebiotics+ADT	0.2369
40	Lavandula	0.2348
41	DHA	0.1847
42	Probiotics	0.1599

11.3.C. Treatment ranking and P score for severe depression

Ranking	Nutraceuticals	P-score
1	Saffron+ADT	0.9712
2	L-theanine+ADT	0.9643
3	Amino_acid+ADT	0.8049
4	Creatine+ADT	0.7286
5	EPA+DHA+ADT	0.6951
6	Vitamin_D+ADT	0.6553
7	SAMe+ADT	0.6358
8	Carnitine	0.6033
9	PEA+ADT	0.5494
10	Tryptophan+ADT	0.5372
11	Chlorella+ADT	0.5231
12	Curcumin+ADT	0.4471
13	Vitamin_B1+ADT	0.4298
14	SJW	0.4052
15	SAMe	0.3941
16	E_amoenum	0.3864
17	Inositol+ADT	0.3589
18	Curcumin	0.3567
19	Folate+ADT	0.3494
20	ADT	0.3427
21	Vitamin_D	0.1483
22	Placebo	0.1352
23	Vitamin_B6+Tryptophan	0.078

11.4. Summary table of Treatment ranking for subgroups with different baseline depressive severity

Ranking	Mild	Moderate	Severe
1	Probiotics+ADT	Vitamin_B12+ADT	Saffron+ADT
2	Vitamin_D+ADT	R_rosea	L-theanine+ADT
3	Nepta	Carnitine+ADT	Amino_acid+ADT
4	Prebiotics+ADT	Tryptophan+ADT	Creatine+ADT
5	EPA+DHA+ADT	Zinc+ADT	EPA+DHA+ADT
6	Zinc+Vitamin_D	Nepta	Vitamin_D+ADT
7	EPA+ADT	Ginkgo+ADT	SAMe+ADT
8	Zinc	Curcumin+ADT	Carnitine
9	Curcumin+ADT	SAMe+ADT	PEA+ADT
10	Fe	DHA+ADT	Tryptophan+ADT
11	Folate+ADT	Probiotics+ADT	Chlorella+ADT
12	Vitamin_D	EPA+ADT	Curcumin+ADT
13	Probiotics	Vitamin_D	Vitamin_B1+ADT
14	SAMe+ADT	Lavandula+ADT	SJW
15	Saffron	EPA+DHA+ADT	SAMe
16	ADT	Mg+ADT	E_amoenum
17	Vitamin_B+ADT	Probiotics+Mg+CoQ10+ADT	Inositol+ADT
18	EPA+DHA	l-arginine+ADT	Curcumin
19	SAMe+Probiotcis	Curcumin+Saffron+ADT	Folate+ADT
20	SJW+Kava	Carnitine	ADT
21	DHA+ADT	Saffron	Vitamin_D
22	SAMe	EPA+DHA	Placebo
23	R_rosea	Mg	Vitamin_B6+Tryptophan
24	Vitamin_C	Folate+ADT	
25	EPA+DHA+Vitamin_C	Curcumin	
26	Probiotics+B7	Vitamin_D+ADT	
27	SJW	Inositol+ADT	
28	Placebo	SJW+ADT	
29	EPA	SJW	
30		Vitamin_C+ADT	
31		SAMe	
32		ADT	
33		SAMe+Vitamin_B12+Folate+ADT	
34		Vitamin_D+Ca	
35		Creatine+ADT	
36		EPA	
37		SAMe+EPA+DHA+Folic+5HTP+Zinc+ADT	
38		Folate+Vitamin_B12+Vitamin_B6	
39		Prebiotics+ADT	
40		Lavandula	

41	DHA
42	Probiotics

12. Component network meta-analysis (CNMA) and Interaction CNMA

12.1. Component network meta-analysis(CNMA)

12.1.A. Estimates of the incremental standardize mean differences of each component in CNMA

Component	iSMD	95% CI	z	p-value
5HTP	-2.1438	(-3.5493; -0.7382)	-2.99	0.0028
ADT	0.4718	(0.1925; 0.7512)	3.31	0.0009
Amino_acid	1.587	(0.3413; 2.8326)	2.5	0.0125
B7	-0.6969	(-1.8949; 0.5012)	-1.14	0.2543
Ca	-0.4126	(-1.4287; 0.6035)	-0.8	0.4261
Carnitine	1.1585	(0.4403; 1.8767)	3.16	0.0016
Chlorella	0.4761	(-0.6283; 1.5804)	0.84	0.3982
CoQ10	-0.7724	(-2.1310; 0.5863)	-1.11	0.2652
Creatine	0.6076	(-0.3116; 1.5269)	1.3	0.1951
Curcumin	0.4743	(0.0837; 0.8648)	2.38	0.0173
DHA	0.1346	(-0.1576; 0.4269)	0.9	0.3666
E_amoenum	0.6809	(-0.5551; 1.9168)	1.08	0.2803
EPA	0.3768	(0.0926; 0.6610)	2.6	0.0094
Fe	0.9197	(-0.2387; 2.0782)	1.56	0.1197
Folate	0.035	(-0.3610; 0.4310)	0.17	0.8625
Ginkgo	0.7582	(-0.0206; 1.5371)	1.91	0.0564
Inositol	0.0528	(-0.8254; 0.9310)	0.12	0.9062
Kava	-0.0757	(-1.3630; 1.2116)	-0.12	0.9083
L-arginine	0.3613	(-0.8775; 1.6001)	0.57	0.5676
L-theanine	3.1452	(1.8284; 4.4619)	4.68	< 0.0001
Lavandula	0.313	(-0.7774; 1.4033)	0.56	0.5738
Mg	0.5887	(-0.1398; 1.3172)	1.58	0.1132
Nepta	1.3078	(0.1341; 2.4816)	2.18	0.029
PEA	0.5617	(-0.5969; 1.7202)	0.95	0.342
Placebo	-0.0255	(-0.2523; 0.2013)	-0.22	0.8254
Prebiotics	0.1701	(-0.7489; 1.0891)	0.36	0.7167
Probiotcis	0.0394	(-1.1091; 1.1878)	0.07	0.9464
Probiotics	0.5585	(0.2159; 0.9012)	3.19	0.0014
R_rosea	0.8825	(0.0673; 1.6978)	2.12	0.0339
Saffron	0.7075	(0.3442; 1.0709)	3.82	0.0001
SAMe	0.4197	(0.0814; 0.7580)	2.43	0.015
SJW	0.427	(0.1301; 0.7239)	2.82	0.0048
Tryptophan	0.5281	(-0.2888; 1.3449)	1.27	0.2051
Vitamin_B	-0.0395	(-1.1808; 1.1019)	-0.07	0.946
Vitamin_B1	0.1908	(-0.9708; 1.3524)	0.32	0.7476

Vitamin_B12	0.636	(-0.1552; 1.4271)	1.58	0.1151
Vitamin_B6	-0.8302	(-1.9221; 0.2618)	-1.49	0.1362
Vitamin_C	-0.1477	(-0.8141; 0.5188)	-0.43	0.6641
Vitamin_D	0.5641	(0.2315; 0.8966)	3.32	0.0009
Zinc	0.9642	(0.3378; 1.5906)	3.02	0.0026

12.1.B. Significant test between NMA and CNMA

	Q	df	p-value
Additive model	1053.45	164	0
Standard model	935.93	145	< 0.0001
Difference	117.52	19	< 0.0001

12.1.C .Forest plot for CNMA of change in depressive symptoms (Additive Model)

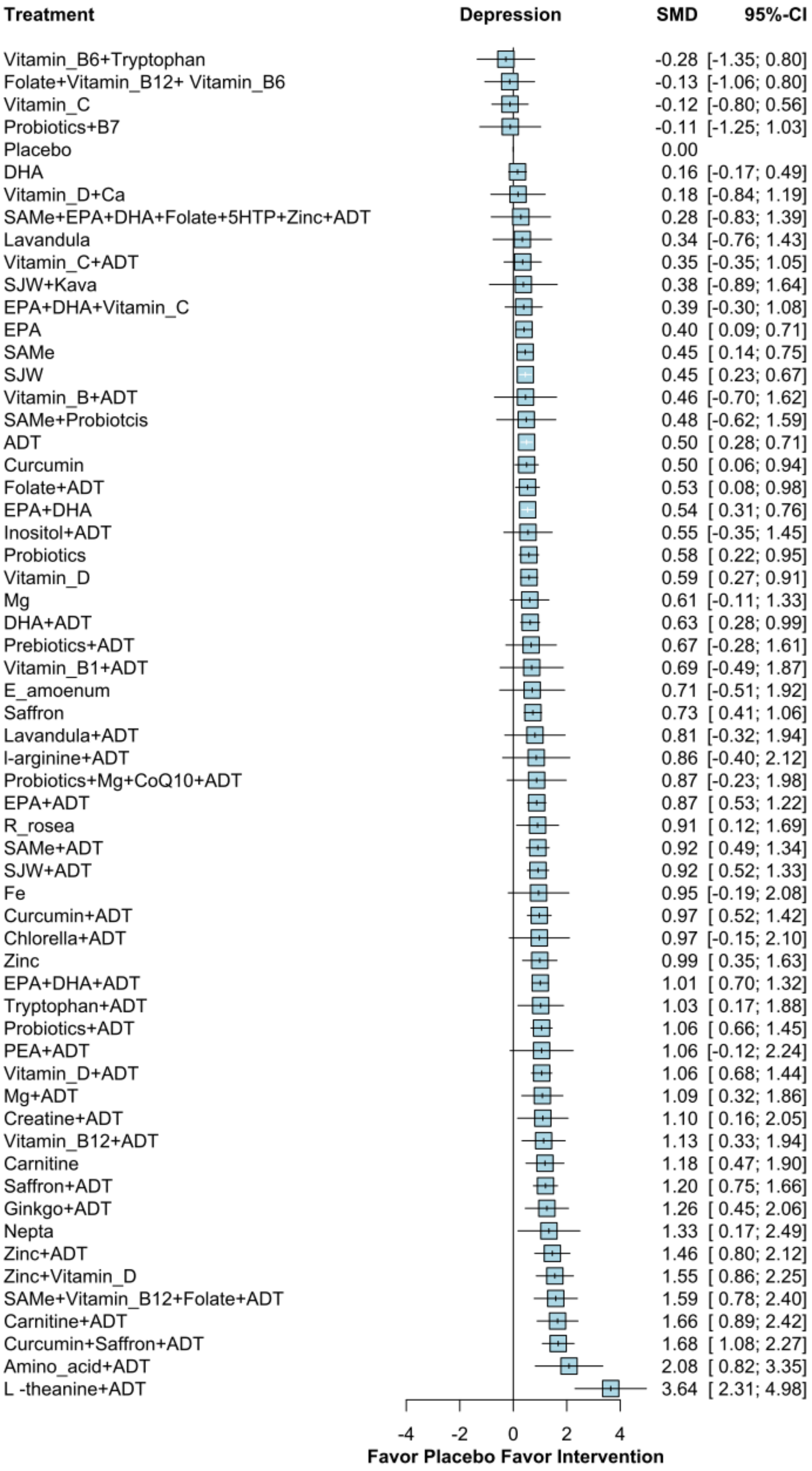

12.1.D. Forest plot for each component of change in depressive symptoms

Components	SMD	SE	SMD	95%-CI
5HTP	-2.1438	0.7171	-2.14	[-3.55; -0.74]
Vitamin_B6	-0.8302	0.5571	-0.83	[-1.92; 0.26]
CoQ10	-0.7724	0.6932	-0.77	[-2.13; 0.59]
B7	-0.6969	0.6113	-0.70	[-1.89; 0.50]
Ca	-0.4126	0.5184	-0.41	[-1.43; 0.60]
Vitamin_C	-0.1477	0.3400	-0.15	[-0.81; 0.52]
Kava	-0.0757	0.6568	-0.08	[-1.36; 1.21]
Vitamin_B	-0.0395	0.5823	-0.04	[-1.18; 1.10]
Placebo	-0.0255	0.1157	-0.03	[-0.25; 0.20]
Folate	0.0350	0.2021	0.03	[-0.36; 0.43]
Probiotcis	0.0394	0.5860	0.04	[-1.11; 1.19]
Inositol	0.0528	0.4481	0.05	[-0.83; 0.93]
DHA	0.1346	0.1491	0.13	[-0.16; 0.43]
Prebiotics	0.1701	0.4689	0.17	[-0.75; 1.09]
Vitamin_B1	0.1908	0.5927	0.19	[-0.97; 1.35]
Lavandula	0.3130	0.5563	0.31	[-0.78; 1.40]
l-arginine	0.3613	0.6320	0.36	[-0.88; 1.60]
EPA	0.3768	0.1450	0.38	[0.09; 0.66]
SAMe	0.4197	0.1726	0.42	[0.08; 0.76]
SJW	0.4270	0.1515	0.43	[0.13; 0.72]
ADT	0.4718	0.1425	0.47	[0.19; 0.75]
Curcumin	0.4743	0.1992	0.47	[0.08; 0.86]
Chlorella	0.4761	0.5635	0.48	[-0.63; 1.58]
Tryptophan	0.5281	0.4168	0.53	[-0.29; 1.34]
Probiotics	0.5585	0.1748	0.56	[0.22; 0.90]
PEA	0.5617	0.5911	0.56	[-0.60; 1.72]
Vitamin_D	0.5641	0.1697	0.56	[0.23; 0.90]
Mg	0.5887	0.3717	0.59	[-0.14; 1.32]
Creatine	0.6076	0.4690	0.61	[-0.31; 1.53]
Vitamin_B12	0.6360	0.4037	0.64	[-0.16; 1.43]
E_amoenum	0.6809	0.6306	0.68	[-0.56; 1.92]
Saffron	0.7075	0.1854	0.71	[0.34; 1.07]
Ginkgo	0.7582	0.3974	0.76	[-0.02; 1.54]
R_rosea	0.8825	0.4160	0.88	[0.07; 1.70]
Fe	0.9197	0.5911	0.92	[-0.24; 2.08]
Zinc	0.9642	0.3196	0.96	[0.34; 1.59]
Carnitine	1.1585	0.3664	1.16	[0.44; 1.88]
Nepta	1.3078	0.5989	1.31	[0.13; 2.48]
Amino_acid	1.5870	0.6355	1.59	[0.34; 2.83]
L -theanine	3.1452	0.6718	3.15	[1.83; 4.46]

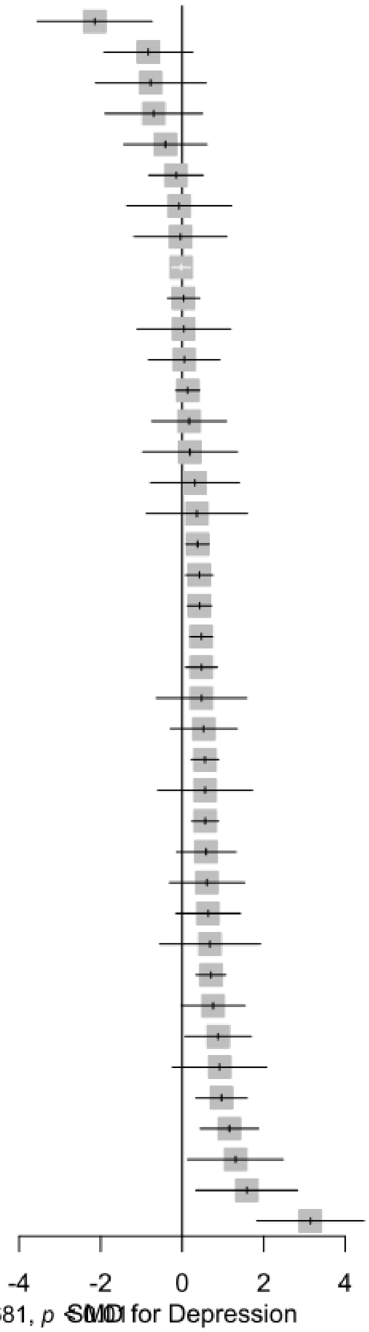

Heterogeneity: I^2 = 56%, τ^2 = 0.0681, p [illegible]

12.1.E. Forest plot for combine result from NMA and CNMA

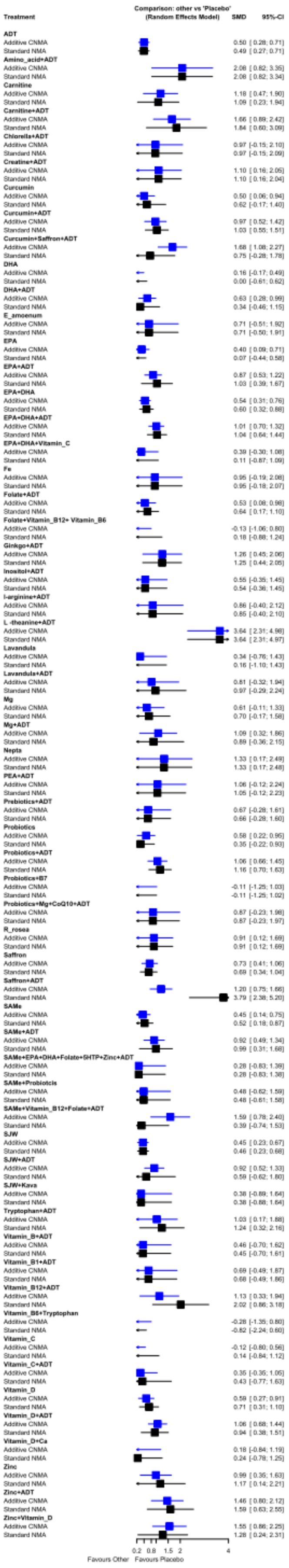

12.2 Interaction Component Network Meta analysis

12.2.A. Interaction CNMA for EPA+DHA+ADT

12.2.A-1. Estimates of the incremental standardize mean differences of each component in interaction CNMA

Intervention	iSMD95%-CI	z	p-value
5HTP	-2.0899 (-3.5564; -0.6233)	-2.79	0.0052
ADT	0.4551 (0.1501; 0.7602)	2.92	0.0035
Amino_acid	1.587 (0.3359; 2.8380)	2.49	0.0129
B7	-0.7098 (-1.9175; 0.4979)	-1.15	0.2493
Ca	-0.4165 (-1.4382; 0.6051)	-0.8	0.4242
Carnitine	1.1468 (0.4203; 1.8734)	3.09	0.002
Chlorella	0.4761 (-0.6344; 1.5865)	0.84	0.4007
CoQ10	-0.7542 (-2.1263; 0.6180)	-1.08	0.2814
Creatine	0.607 (-0.3159; 1.5300)	1.29	0.1974
Curcumin	0.4718 (0.0789; 0.8646)	2.35	0.0186
DHA	0.1148 (-0.2108; 0.4404)	0.69	0.4896
E_amoenum	0.6618 (-0.5873; 1.9108)	1.04	0.2991
EPA	0.3602 (0.0497; 0.6706)	2.27	0.023
Fe	0.9006 (-0.2718; 2.0731)	1.51	0.1322
Folate	0.0358 (-0.3623; 0.4339)	0.18	0.86
Ginkgo	0.7584 (-0.0248; 1.5416)	1.9	0.0577
Inositol	0.0528 (-0.8292; 0.9348)	0.12	0.9065
Kava	-0.0774 (-1.3702; 1.2154)	-0.12	0.9066
L-arginine	0.3613 (-0.8829; 1.6055)	0.57	0.5693
L-theanine	3.1452 (1.8233; 4.4670)	4.66	< 0.0001
Lavandula	0.3046 (-0.7922; 1.4013)	0.54	0.5862
Mg	0.5767 (-0.1601; 1.3135)	1.53	0.125
Nepta	1.2912 (0.1053; 2.4770)	2.13	0.0328
PEA	0.5617 (-0.6027; 1.7261)	0.95	0.3444
Placebo	-0.0446 (-0.3101; 0.2208)	-0.33	0.7417
Prebiotics	0.1706 (-0.7521; 1.0932)	0.36	0.7171
Probiotcis	0.0336 (-1.1218; 1.1890)	0.06	0.9546
Probiotics	0.5524 (0.2051; 0.8997)	3.12	0.0018
R_rosea	0.8641 (0.0345; 1.6936)	2.04	0.0412
Saffron	0.6928 (0.3120; 1.0737)	3.57	0.0004
SAMe	0.4064 (0.0525; 0.7603)	2.25	0.0244
SJW	0.4096 (0.0863; 0.7329)	2.48	0.013
Tryptophan	0.53 (-0.2905; 1.3504)	1.27	0.2055
Vitamin_B	-0.0395 (-1.1867; 1.1078)	-0.07	0.9462

Vitamin_B1	0.1908 (-0.9766; 1.3582)	0.32	0.7488
Vitamin_B12	0.6416 (-0.1545; 1.4377)	1.58	0.1142
Vitamin_B6	-0.8541 (-1.9632; 0.2550)	-1.51	0.1312
Vitamin_C	-0.1542 (-0.8257; 0.5173)	-0.45	0.6526
Vitamin_D	0.5528 (0.2091; 0.8965)	3.15	0.0016
Zinc	0.9593 (0.3289; 1.5896)	2.98	0.0029
EPA.DHA.ADT	0.0704 (-0.4240; 0.5648)	0.28	0.7802

12.2.A-2. Model Comparison for NMA, CNMA and interaction CNMA

	Q	df
Standard model (NMA)	935.93	145
Additive model (CNMA)	1053.45	164
interaction CNMA	1053.33	163

12.2.A-3. Forest plot for combine result from NMA , CNMA and Interaction CNMA

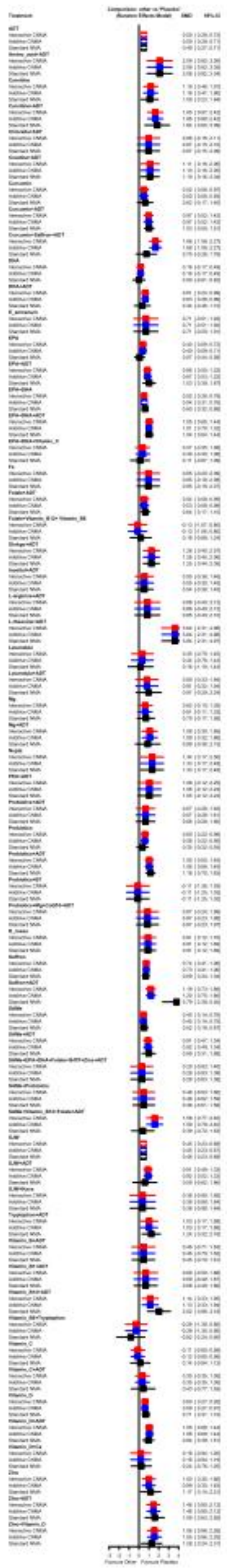

12.2.B. Interaction CNMA for EPA+DHA

12.2.B-1. Estimates of the incremental standardize mean differences of each component in interaction CNMA

Intervention	iSMD95%-CI	z	p-value
5HTP	-2.1226 (-3.5347; -0.7106)	-2.95	0.0032
ADT	0.5056 (0.2099; 0.8013)	3.35	0.0008
Amino_acid	1.5870 (0.3376; 2.8363)	2.49	0.0128
B7	-0.6716 (-1.8760; 0.5327)	-1.09	0.2744
Ca	-0.4047 (-1.4245; 0.6151)	-0.78	0.4367
Carnitine	1.1827 (0.4592; 1.9063)	3.2	0.0014
Chlorella	0.4761 (-0.6324; 1.5846)	0.84	0.3999
CoQ10	-0.8093 (-2.1767; 0.5582)	-1.16	0.2461
Creatine	0.6072 (-0.3146; 1.5290)	1.29	0.1967
Curcumin	0.4787 (0.0866; 0.8708)	2.39	0.0167
DHA	0.097 (-0.2149; 0.4088)	0.61	0.5424
E_amoenum	0.7188 (-0.5256; 1.9631)	1.13	0.2576
EPA	0.3562 (0.0649; 0.6475)	2.4	0.0166
Fe	0.9577 (-0.2097; 2.1251)	1.61	0.1079
Folate	0.0337 (-0.3637; 0.4311)	0.17	0.8679
Ginkgo	0.7584 (-0.0234; 1.5401)	1.9	0.0573
Inositol	0.0528 (-0.8280; 0.9336)	0.12	0.9064
Kava	-0.0727 (-1.3637; 1.2184)	-0.11	0.9122
l-arginine	0.3613 (-0.8812; 1.6038)	0.57	0.5687
L-theanine	3.1452 (1.8249; 4.4654)	4.67	0.0001
Lavandula	0.3299 (-0.7647; 1.4245)	0.59	0.5547
Mg	0.6129 (-0.1209; 1.3467)	1.64	0.1016
Nepta	1.3416 (0.1599; 2.5233)	2.23	0.0261
PEA	0.5617 (-0.6009; 1.7242)	0.95	0.3437
Placebo	0.0124 (-0.2387; 0.2635)	0.1	0.923
Prebiotics	0.1704 (-0.7511; 1.0919)	0.36	0.717
Probiotcis	0.0495 (-1.1036; 1.2026)	0.08	0.933
Probiotics	0.5712 (0.2256; 0.9168)	3.24	0.0012
R_rosea	0.5712 (0.2256; 0.9168)	3.24	0.0012
Saffron	0.9192 (0.0949; 1.7435)	2.19	0.0288
SAMe	0.7382 (0.3639; 1.1126)	3.87	0.0001
SJW	0.4475 (0.0995; 0.7956)	2.52	0.0117
Tryptophan	0.4619 (0.1484; 0.7754)	2.89	0.0039
Vitamin_B	0.5248 (-0.2944; 1.3441)	1.26	0.2093
Vitamin_B1	-0.0395 (-1.1849; 1.1059)	-0.07	0.9461

Vitamin_B12	0.1908 (-0.9748; 1.3563)	0.32	0.7484
Vitamin_B6	0.6254 (-0.1691; 1.4199)	1.54	0.1229
Vitamin_C	-0.7858 (-1.8886; 0.3171)	-1.4	0.1626
Vitamin_D	-0.0802 (-0.7753; 0.6149)	-0.23	0.8211
Zinc	0.5862 (0.2468; 0.9257)	3.38	0.0007
EPA.DHA	0.9748 (0.3458; 1.6038)	3.04	0.0024

12.2.B-2. Model Comparison for NMA, CNMA and interaction CNMA

	Q	df
Standard model (NMA)	935.93	145
Additive model (CNMA)	1053.45	164
interaction CNMA	1051.01	163

12.2.B-3. Forest plot for combine result from NMA , CNMA and Interaction CNMA

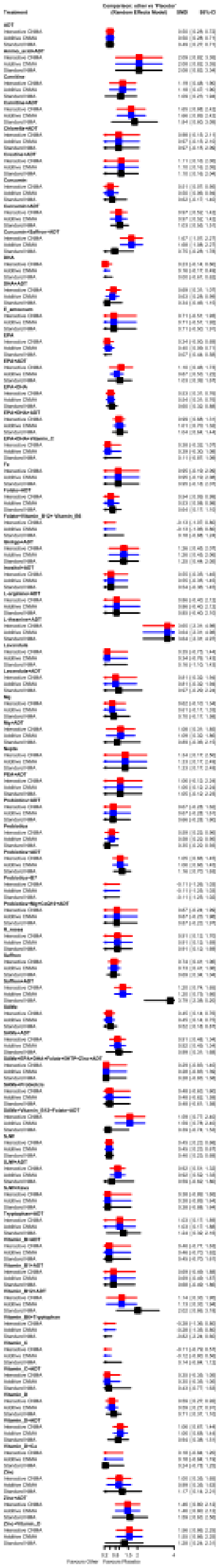

12.2.C. Interaction CNMA for SAMe+ADT

12.2.C-1. Estimates of the incremental standardize mean differences of each component in interaction CNMA

**Intervention**	**iSMD95%-CI**	**z**	**p-value**
5HTP	-2.1088 (-3.5446; -0.6730)	-2.88	0.004
ADT	0.4601 (0.1674; 0.7528)	3.08	0.0021
Amino_acid	1.5870 (0.3358; 2.8381)	2.49	0.0129
B7	-0.7031 (-1.9083; 0.5020)	-1.14	0.2528
Ca	-0.4145 (-1.4359; 0.6070)	-0.8	0.4265
Carnitine	1.1516 (0.4282; 1.8750)	3.12	0.0018
Chlorella	0.4761 (-0.6345; 1.5867)	0.84	0.4008
CoQ10	-0.7627 (-2.1305; 0.6052)	-1.09	0.2745
Creatine	0.6070 (-0.3160; 1.5301)	1.29	0.1974
Curcumin	0.4728 (0.0802; 0.8655)	2.36	0.0183
DHA	0.1322 (-0.1621; 0.4265)	0.88	0.3785
E_amoenum	0.6717 (-0.5717; 1.9152)	1.06	0.2897
EPA	0.3742 (0.0876; 0.6608)	2.56	0.0105
Fe	0.9106 (-0.2558; 2.0771)	1.53	0.126
Folate	0.0367 (-0.3615; 0.4350)	0.18	0.8565
Ginkgo	0.7584 (-0.0249; 1.5417)	1.9	0.0577
Inositol	0.0528 (-0.8293; 0.9349)	0.12	0.9066
Kava	-0.0747 (-1.3676; 1.2183)	-0.11	0.9099
L-arginine	0.3613 (-0.8831; 1.6057)	0.57	0.5693
L-theanine	3.1452 (1.8231; 4.4672)	4.66	0.0001
Lavandula	0.3071 (-0.7889; 1.4031)	0.55	0.5829
Mg	0.5819 (-0.1516; 1.3153)	1.55	0.12
Nepta	1.2961 (0.1132; 2.4790)	2.15	0.0317
PEA	0.5617 (-0.6029; 1.7262)	0.95	0.3445
Placebo	-0.0347 (-0.2716; 0.2022)	-0.29	0.7743
Prebiotics	0.1706 (-0.7522; 1.0934)	0.36	0.7171
Probiotcis	0.0598 (-1.1039; 1.2235)	0.1	0.9198
Probiotics	0.5557 (0.2106; 0.9008)	3.16	0.0016
R_rosea	0.8726 (0.0505; 1.6948)	2.08	0.0375
Saffron	0.6990 (0.3284; 1.0696)	3.7	0.0002
SAMe	0.3902 (-0.0080; 0.7884)	1.92	0.0548
SJW	0.4169 (0.1099; 0.7238)	2.66	0.0078
Tryptophan	0.5306 (-0.2900; 1.3512)	1.27	0.205
Vitamin_B	-0.0395 (-1.1869; 1.1079)	-0.07	0.9462
Vitamin_B1	0.1908 (-0.9768; 1.3583)	0.32	0.7488

Vitamin_B12	0.6489 (-0.1512; 1.4490)	1.59	0.1119
Vitamin_B6	-0.8515 (-1.9577; 0.2548)	-1.51	0.1314
Vitamin_C	-0.1508 (-0.8211; 0.5195)	-0.44	0.6593
Vitamin_D	0.5586 (0.2222; 0.8951)	3.25	0.0011
Zinc	0.9620 (0.3325; 1.5916)	3	0.0027
SAMe.ADT	0.1116 (-0.6538; 0.8771)	0.29	0.775

12.2.C-2. Model Comparison for NMA, CNMA and interaction CNMA

	Q	df
Standard model (NMA)	935.93	145
Additive model (CNMA)	1053.45	164
interaction CNMA	1053.12	163

12.2.C-3. Forest plot for combine result from NMA , CNMA and Interaction CNMA

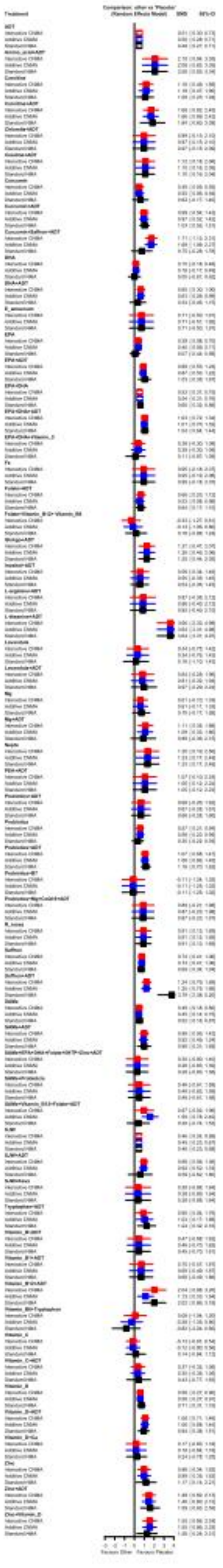

12.2.D.Interaction CNMA for Curcumin+ADT

12.2.D-1. Estimates of the incremental standardize mean differences of each component in interaction CNMA

Intervention	iSMD95%-CI	z	p-value
5HTP	-2.1259 (-3.5309; -0.7210)	-2.97	0.003
ADT	0.4579 (0.1776; 0.7382)	3.2	0.0014
Amino_acid	1.5870 (0.3420; 2.8319)	2.5	0.0125
B7	-0.7036 (-1.9009; 0.4938)	-1.15	0.2494
Ca	-0.4147 (-1.4302; 0.6008)	-0.8	0.4235
Carnitine	1.1501 (0.4322; 1.8680)	3.14	0.0017
Chlorella	0.4761 (-0.6275; 1.5797)	0.85	0.3978
CoQ10	-0.7611 (-2.1190; 0.5968)	-1.1	0.272
Creatine	0.6077 (-0.3111; 1.5265)	1.3	0.1949
Curcumin	0.1960 (-0.4450; 0.8370)	0.6	0.549
DHA	0.1319 (-0.1602; 0.4240)	0.89	0.3761
E_amoenum	0.6708 (-0.5646; 1.9062)	1.06	0.2872
EPA	0.3732 (0.0891; 0.6574)	2.57	0.01
Fe	0.9097 (-0.2482; 2.0675)	1.54	0.1236
Folate	0.0355 (-0.3603; 0.4313)	0.18	0.8604
Ginkgo	0.7582 (-0.0201; 1.5365)	1.91	0.0562
Inositol	0.0528 (-0.8249; 0.9305)	0.12	0.9062
Kava	-0.0741 (-1.3607; 1.2126)	-0.11	0.9102
L-arginine	0.3613 (-0.8768; 1.5994)	0.57	0.5674
L-theanine	3.1452 (1.8290; 4.4613)	4.68	0.0001
Lavandula	0.3060 (-0.7839; 1.3958)	0.55	0.5822
Mg	0.5808 (-0.1474; 1.3090)	1.56	0.118
Nepta	1.2939 (0.1206; 2.4672)	2.16	0.0307
PEA	0.5617 (-0.5962; 1.7195)	0.95	0.3417
Placebo	-0.0356 (-0.2630; 0.1918)	-0.31	0.759
Prebiotics	0.1701 (-0.7485; 1.0886)	0.36	0.7167
Probiotcis	0.0385 (-1.1092; 1.1863)	0.07	0.9475
Probiotics	0.5552 (0.2127; 0.8977)	3.18	0.0015
R_rosea	0.8714 (0.0563; 1.6864)	2.1	0.0361
Saffron	0.7280 (0.3629; 1.0930)	3.91	0.0001
SAMe	0.4105 (0.0720; 0.7490)	2.38	0.0175
SJW	0.4153 (0.1179; 0.7128)	2.74	0.0062
Tryptophan	0.5283 (-0.2882; 1.3447)	1.27	0.2047
Vitamin_B	-0.0395 (-1.1801; 1.1012)	-0.07	0.9459
Vitamin_B1	0.1908 (-0.9701; 1.3517)	0.32	0.7474

Vitamin_B12	0.6402 (-0.1505; 1.4309)	1.59	0.1125
Vitamin_B6	-0.8446 (-1.9363; 0.2470)	-1.52	0.1294
Vitamin_C	-0.1512 (-0.8172; 0.5149)	-0.44	0.6565
Vitamin_D	0.5582 (0.2256; 0.8907)	3.29	0.001
Zinc	0.9614 (0.3353; 1.5875)	3.01	0.0026
Curcumin.ADT	0.3722 (-0.3079; 1.0522)	1.07	0.2834

12.2.D-2. Model Comparison for NMA, CNMA and interaction CNMA

	Q	df
Standard model (NMA)	935.93	145
Additive model (CNMA)	1053.45	164
interaction CNMA	1043.65	163

12.2.D-3. Forest plot for combine result from NMA , CNMA and Interaction CNMA

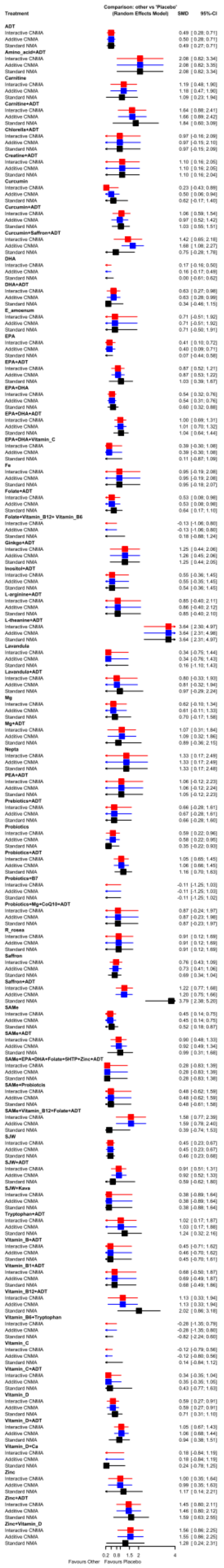

12.2.E. Interaction CNMA for Probiotics+ADT

12.2.E-1. Estimates of the incremental standardize mean differences of each component in interaction CNMA

Supplement	iSMD95%-CI	z	p-value
5HTP	-2.0820 (-3.4899; -0.6741)	-2.9	0.0038
ADT	0.4402 (0.1547; 0.7257)	3.02	0.0025
Amino_acid	1.5870 (0.3432; 2.8307)	2.5	0.0124
B7	-0.4657 (-1.7431; 0.8117)	-0.71	0.4749
Ca	-0.4208 (-1.4353; 0.5937)	-0.81	0.4163
Carnitine	1.1348 (0.4163; 1.8532)	3.1	0.002
Chlorella	0.4761 (-0.6262; 1.5783)	0.85	0.3972
CoQ10	-0.4777 (-1.9495; 0.9942)	-0.64	0.5247
Creatine	0.6078 (-0.3101; 1.5258)	1.3	0.1944
Curcumin	0.4697 (0.0798; 0.8596)	2.36	0.0182
DHA	0.1239 (-0.1685; 0.4164)	0.83	0.4062
E_amoenum	0.6415 (-0.5949; 1.8778)	1.02	0.3092
EPA	0.3628 (0.0778; 0.6478)	2.5	0.0126
Fe	0.8804 (-0.2785; 2.0393)	1.49	0.1365
Folate	0.0362 (-0.3591; 0.4316)	0.18	0.8574
Ginkgo	0.7582 (-0.0192; 1.5355)	1.91	0.0559
Inositol	0.0528 (-0.8241; 0.9296)	0.12	0.9061
Kava	-0.0805 (-1.3659; 1.2050)	-0.12	0.9023
L-arginine	0.3613 (-0.8756; 1.5982)	0.57	0.567
L-theanine	3.1452 (1.8302; 4.4602)	4.69	0.0001
Lavandula	0.2971 (-0.7921; 1.3863)	0.53	0.5929
Mg	0.5646 (-0.1643; 1.2934)	1.52	0.129
Nepta	1.2762 (0.1029; 2.4495)	2.13	0.033
PEA	0.5617 (-0.5949; 1.7182)	0.95	0.3412
Placebo	-0.0649 (-0.3039; 0.1740)	-0.53	0.5944
Prebiotics	0.1700 (-0.7477; 1.0877)	0.36	0.7166
Probiotcis	0.0274 (-1.1192; 1.1739)	0.05	0.9627
Probiotics	0.2880 (-0.3389; 0.9149)	0.9	0.3679
R_rosea	0.8454 (0.0283; 1.6625)	2.03	0.0426
Saffron	0.6774 (0.3100; 1.0447)	3.61	0.0003
SAMe	0.3923 (0.0505; 0.7341)	2.25	0.0245
SJW	0.3924 (0.0886; 0.6962)	2.53	0.0114
Tryptophan	0.5320 (-0.2837; 1.3477)	1.28	0.2012
Vitamin_B	-0.0395 (-1.1788; 1.0998)	-0.07	0.9459
Vitamin_B1	0.1908 (-0.9688; 1.3504)	0.32	0.7471

Vitamin_B12	0.6461 (-0.1439; 1.4361)	1.6	0.109
Vitamin_B6	-0.8750 (-1.9687; 0.2187)	-1.57	0.1169
Vitamin_C	-0.1613 (-0.8271; 0.5045)	-0.47	0.635
Vitamin_D	0.5410 (0.2060; 0.8760)	3.17	0.0015
Zinc	0.9533 (0.3275; 1.5790)	2.99	0.0028
Probiotics.ADT	0.3851 (-0.3629; 1.1332)	1.01	0.313

12.2.E-2. Model Comparison for NMA, CNMA and interaction CNMA

	Q	df
Standard model (NMA)	935.93	145
Additive model (CNMA)	1053.45	164
interaction CNMA	1043.65	163

12.2.E-3. Forest plot for combine result from NMA , CNMA and Interaction CNMA

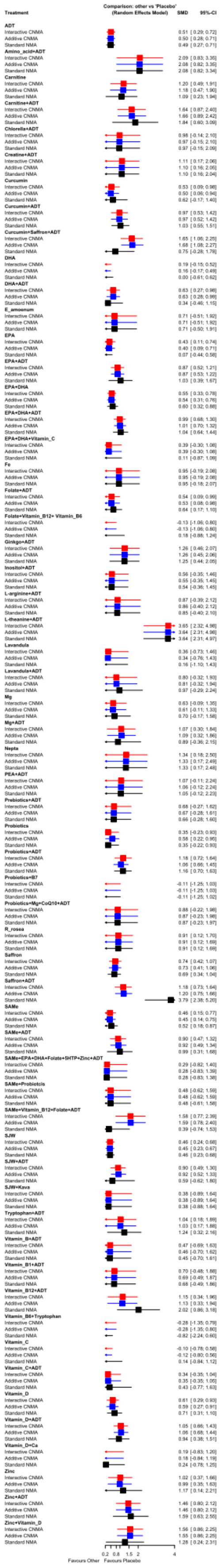

12.2.F.Interaction CNMA for EPA+ADT

12.2.F-1. Estimates of the incremental standardize mean differences of each component in interaction CNMA

Intervention	iSMD95%-CI	z	p-value
5HTP	-2.1128 (-3.5253; -0.7002)	-2.93	0.0034
ADT	0.4614 (0.1800; 0.7428)	3.21	0.0013
Amino_acid	1.5870 (0.3378; 2.8361)	2.49	0.0128
B7	-0.7057 (-1.9080; 0.4966)	-1.15	0.25
Ca	-0.4153 (-1.4348; 0.6042)	-0.8	0.4246
Carnitine	1.1509 (0.4303; 1.8715)	3.13	0.0017
Chlorella	0.4761 (-0.6323; 1.5844)	0.84	0.3999
CoQ10	-0.7602 (-2.1239; 0.6035)	-1.09	0.2746
Creatine	0.6072 (-0.3144; 1.5289)	1.29	0.1966
Curcumin	0.4726 (0.0807; 0.8644)	2.36	0.0181
DHA	0.1893 (-0.1316; 0.5102)	1.16	0.2475
E_amoenum	0.6678 (-0.5723; 1.9078)	1.06	0.2912
EPA	0.3027 (-0.0334; 0.6388)	1.77	0.0775
Fe	0.9067 (-0.2562; 2.0695)	1.53	0.1265
Folate	0.0355 (-0.3618; 0.4329)	0.18	0.861
Ginkgo	0.7583 (-0.0233; 1.5400)	1.9	0.0572
Inositol	0.0528 (-0.8279; 0.9335)	0.12	0.9064
Kava	-0.0773 (-1.3682; 1.2136)	-0.12	0.9065
L-arginine	0.3613 (-0.8810; 1.6037)	0.57	0.5687
L-theanine	3.1452 (1.8250; 4.4653)	4.67	0.0001
Lavandula	0.3077 (-0.7858; 1.4013)	0.55	0.5813
Mg	0.5807 (-0.1501; 1.3116)	1.56	0.1194
Nepta	1.2974 (0.1194; 2.4755)	2.16	0.0309
PEA	0.5617 (-0.6007; 1.7241)	0.95	0.3436
Placebo	-0.0386 (-0.2684; 0.1911)	-0.33	0.7418
Prebiotics	0.1704 (-0.7510; 1.0918)	0.36	0.717
Probiotcis	0.0350 (-1.1176; 1.1877)	0.06	0.9525
Probiotics	0.5543 (0.2104; 0.8983)	3.16	0.0016
R_rosea	0.8702 (0.0519; 1.6885)	2.08	0.0371
Saffron	0.6979 (0.3326; 1.0632)	3.74	0.0002
SAMe	0.4110 (0.0709; 0.7511)	2.37	0.0179
SJW	0.4156 (0.1164; 0.7147)	2.72	0.0065
Tryptophan	0.5295 (-0.2896; 1.3486)	1.27	0.2052
Vitamin_B	-0.0395 (-1.1847; 1.1058)	-0.07	0.9461
Vitamin_B1	0.1908 (-0.9747; 1.3562)	0.32	0.7484

Vitamin_B12	0.6395 (-0.1543; 1.4334)	1.58	0.1143
Vitamin_B6	-0.8460 (-1.9420; 0.2500)	-1.51	0.1303
Vitamin_C	-0.1522 (-0.8210; 0.5166)	-0.45	0.6556
Vitamin_D	0.5563 (0.2221; 0.8906)	3.26	0.0011
Zinc	0.9608 (0.3325; 1.5891)	3	0.0027
EPA.ADT	0.2938 (-0.4075; 0.9950)	0.82	0.4116

12.2.F-2. Model Comparison for NMA, CNMA and interaction CNMA

	Q	df
Standard model (NMA)	935.93	145
Additive model (CNMA)	1053.45	164
interaction CNMA	1052.81	163

12.2.F-3. Forest plot for combine result from NMA , CNMA and Interaction CNMA

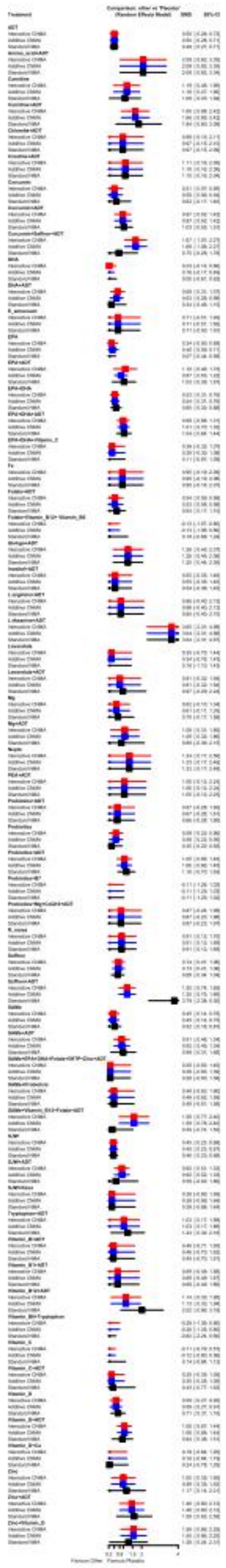

12.G.Interaction CNMA for DHA+ADT

12.2.G-1. Estimates of the incremental standardize mean differences of each component in interaction CNMA

Intervention	iSMD95%-CI	z	p-value
5HTP	-2.1690 (-3.5783; -0.7598)	-3.02	0.0026
ADT	0.4772 (0.1971; 0.7573)	3.34	0.0008
Amino_acid	1.5870 (0.3393; 2.8346)	2.49	0.0127
B7	-0.6925 (-1.8929; 0.5079)	-1.13	0.2582
Ca	-0.4112 (-1.4292; 0.6068)	-0.79	0.4286
Carnitine	1.1626 (0.4431; 1.8821)	3.17	0.0015
Chlorella	0.4761 (-0.6305; 1.5827)	0.84	0.3991
CoQ10	-0.7787 (-2.1401; 0.5827)	-1.12	0.2623
Creatine	0.6074 (-0.3132; 1.5280)	1.29	0.196
Curcumin	0.4750 (0.0837; 0.8662)	2.38	0.0173
DHA	0.2043 (-0.1228; 0.5313)	1.22	0.2209
E_amoenum	0.6875 (-0.5506; 1.9257)	1.09	0.2765
EPA	0.3259 (0.0216; 0.6302)	2.1	0.0358
Fe	0.9264 (-0.2344; 2.0872)	1.56	0.1178
Folate	0.0348 (-0.3620; 0.4316)	0.17	0.8634
Ginkgo	0.7583 (-0.0222; 1.5388)	1.9	0.0569
Inositol	0.0528 (-0.8268; 0.9324)	0.12	0.9063
Kava	-0.0749 (-1.3642; 1.2145)	-0.11	0.9094
L-arginine	0.3613 (-0.8795; 1.6021)	0.57	0.5682
L-theanine	3.1452 (1.8265; 4.4638)	4.67	0.0001
Lavandula	0.3156 (-0.7765; 1.4078)	0.57	0.5711
Mg	0.5928 (-0.1369; 1.3225)	1.59	0.1114
Nepta	1.3132 (0.1371; 2.4893)	2.19	0.0286
PEA	0.5617 (-0.5991; 1.7224)	0.95	0.3429
Placebo	-0.0189 (-0.2465; 0.2088)	-0.16	0.8708
Prebiotics	0.1703 (-0.7501; 1.0907)	0.36	0.7169
Probiotcis	0.0412 (-1.1096; 1.1921)	0.07	0.944
Probiotics	0.5608 (0.2175; 0.9041)	3.2	0.0014
R_rosea	0.8888 (0.0720; 1.7056)	2.13	0.0329
Saffron	0.7128 (0.3486; 1.0769)	3.84	0.0001
SAMe	0.4245 (0.0854; 0.7636)	2.45	0.0141
SJW	0.4328 (0.1351; 0.7305)	2.85	0.0044
Tryptophan	0.5275 (-0.2907; 1.3456)	1.26	0.2064
Vitamin_B	-0.0395 (-1.1830; 1.1041)	-0.07	0.9461
Vitamin_B1	0.1908 (-0.9730; 1.3545)	0.32	0.748

Vitamin_B12	0.6344 (-0.1583; 1.4270)	1.57	0.1168
Vitamin_B6	-0.8230 (-1.9171; 0.2711)	-1.47	0.1404
Vitamin_C	-0.1454 (-0.8132; 0.5224)	-0.43	0.6695
Vitamin_D	0.5679 (0.2346; 0.9012)	3.34	0.0008
Zinc	0.9661 (0.3387; 1.5936)	3.02	0.0025
DHA.ADT	-0.4078 (-1.2617; 0.4460)	-0.94	0.3492

12.2.G-2. Model Comparison for NMA, CNMA and interaction CNMA

	Q	df
Standard model (NMA)	935.93	145
Additive model (CNMA)	1053.45	164
interaction CNMA	1051.97	163

12.2.G-3. Forest plot for combine result from NMA , CNMA and Interaction CNMA

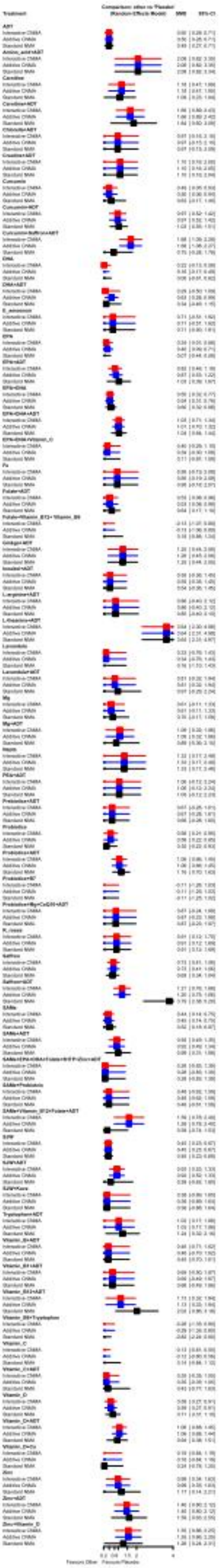

12.2.H.Interaction CNMA for Vitamin D+ADT

12.2.H-1. Estimates of the incremental standardize mean differences of each component in interaction CNMA

Intervention	iSMD95%-CI	z	p-value
5HTP	-2.1769 (-3.5872; -0.7666)	-3.03	0.0025
ADT	0.4886 (0.2028; 0.7745)	3.35	0.0008
Amino_acid	1.5870 (0.3415; 2.8324)	2.5	0.0125
B7	-0.6828 (-1.8817; 0.5160)	-1.12	0.2643
Ca	-0.4407 (-1.4616; 0.5803)	-0.85	0.3976
Carnitine	1.1710 (0.4516; 1.8905)	3.19	0.0014
Chlorella	0.4761 (-0.6280; 1.5802)	0.85	0.398
CoQ10	-0.7921 (-2.1523; 0.5681)	-1.14	0.2537
Creatine	0.6077 (-0.3114; 1.5267)	1.3	0.195
Curcumin	0.4767 (0.0862; 0.8672)	2.39	0.0167
DHA	0.1403 (-0.1526; 0.4332)	0.94	0.3478
E_amoenum	0.7018 (-0.5362; 1.9398)	1.11	0.2665
EPA	0.3841 (0.0987; 0.6695)	2.64	0.0083
Fe	0.9407 (-0.2200; 2.1014)	1.59	0.1122
Folate	0.0343 (-0.3616; 0.4302)	0.17	0.8652
Ginkgo	0.7582 (-0.0204; 1.5369)	1.91	0.0563
Inositol	0.0528 (-0.8252; 0.9308)	0.12	0.9062
Kava	-0.0731 (-1.3602; 1.2140)	-0.11	0.9114
L-arginine	0.3613 (-0.8772; 1.5998)	0.57	0.5675
L-theanine	3.1452 (1.8286; 4.4617)	4.68	< 0.0001
Lavandula	0.3214 (-0.7692; 1.4120)	0.58	0.5636
Mg	0.6015 (-0.1283; 1.3313)	1.62	0.1062
Nepta	1.3247 (0.1496; 2.4997)	2.21	0.0271
PEA	0.5617 (-0.5967; 1.7200)	0.95	0.3419
Placebo	-0.0046 (-0.2436; 0.2345)	-0.04	0.9701
Prebiotics	0.1701 (-0.7487; 1.0889)	0.36	0.7167
Probiotcis	0.0458 (-1.1026; 1.1943)	0.08	0.9376
Probiotics	0.5655 (0.2220; 0.9090)	3.23	0.0013
R_rosea	0.9023 (0.0841; 1.7205)	2.16	0.0307
Saffron	0.7234 (0.3556; 1.0913)	3.85	0.0001
SAMe	0.4342 (0.0920; 0.7765)	2.49	0.0129
SJW	0.4454 (0.1412; 0.7495)	2.87	0.0041
Tryptophan	0.5260 (-0.2908; 1.3427)	1.26	0.2069
Vitamin_B	-0.0395 (-1.1806; 1.1016)	-0.07	0.9459
Vitamin_B1	0.1908 (-0.9706; 1.3521)	0.32	0.7475

Vitamin_B12	0.6305 (-0.1607; 1.4218)	1.56	0.1183
Vitamin_B6	-0.8060 (-1.9012; 0.2892)	-1.44	0.1492
Vitamin_C	-0.1405 (-0.8073; 0.5264)	-0.41	0.6797
Vitamin_D	0.6410 (0.2078; 1.0741)	2.9	0.0037
Zinc	0.9706 (0.3439; 1.5973)	3.04	0.0024
Vitamin_D.ADT	-0.1873 (-0.8632; 0.4886)	-0.54	0.587

12.2.H-2. Model Comparison for NMA, CNMA and interaction CNMA

	Q	df
Standard model (NMA)	935.93	145
Additive model (CNMA)	1053.45	164
interaction CNMA	1046.09	163

12.2.H-3. Forest plot for combine result from NMA , CNMA and Interaction CNMA

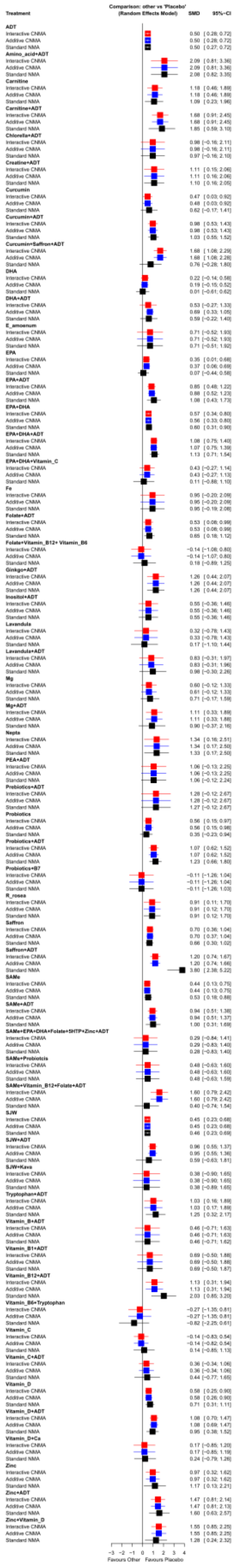

12.2.I.Interaction CNMA for Saffron+ADT

12.2.I-1. Estimates of the incremental standardize mean differences of each component in interaction CNMA

Intervention	iSMD95%-CI	z	p-value
5HTP	-2.0420 (-3.4270; -0.6570)	-2.89	0.0039
ADT	0.3971 (0.1191; 0.6751)	2.8	0.0051
Amino_acid	1.5870 (0.3567; 2.8172)	2.53	0.0115
B7	-0.7338 (-1.9144; 0.4467)	-1.22	0.2231
Ca	-0.4243 (-1.4259; 0.5773)	-0.83	0.4064
Carnitine	1.1121 (0.4031; 1.8211)	3.07	0.0021
Chlorella	0.4761 (-0.6109; 1.5630)	0.86	0.3906
CoQ10	-0.7101 (-2.0485; 0.6283)	-1.04	0.2984
Creatine	0.6094 (-0.2993; 1.5181)	1.31	0.1887
Curcumin	0.4771 (0.0923; 0.8618)	2.43	0.0151
DHA	0.1193 (-0.1688; 0.4074)	0.81	0.417
E_amoenum	0.6248 (-0.5953; 1.8450)	1	0.3155
EPA	0.3555 (0.0752; 0.6358)	2.49	0.0129
Fe	0.8637 (-0.2779; 2.0053)	1.48	0.1381
Folate	0.0375 (-0.3527; 0.4277)	0.19	0.8506
Ginkgo	0.7577 (-0.0087; 1.5242)	1.94	0.0527
Inositol	0.0527 (-0.8145; 0.9199)	0.12	0.9052
Kava	-0.0680 (-1.3399; 1.2038)	-0.1	0.9165
L-arginine	0.3613 (-0.8620; 1.5846)	0.58	0.5627
L-theanine	3.1452 (1.8430; 4.4474)	4.73	0.0001
Lavandula	0.2754 (-0.8017; 1.3525)	0.5	0.6162
Mg	0.5455 (-0.1740; 1.2650)	1.49	0.1373
Nepta	1.2331 (0.0760; 2.3902)	2.09	0.0367
PEA	0.5617 (-0.5803; 1.7037)	0.96	0.3351
Placebo	-0.0816 (-0.3070; 0.1438)	-0.71	0.4782
Prebiotics	0.1688 (-0.7396; 1.0773)	0.36	0.7156
Probiotcis	0.0346 (-1.0959; 1.1652)	0.06	0.9521
Probiotics	0.5395 (0.2016; 0.8773)	3.13	0.0017
R_rosea	0.8213 (0.0164; 1.6262)	2	0.0455
Saffron	0.5188 (0.1478; 0.8899)	2.74	0.0061
SAMe	0.3684 (0.0340; 0.7028)	2.16	0.0308
SJW	0.3633 (0.0691; 0.6576)	2.42	0.0155
Tryptophan	0.5291 (-0.2779; 1.3362)	1.29	0.1988
Vitamin_B	-0.0395 (-1.1640; 1.0851)	-0.07	0.9452
Vitamin_B1	0.1908 (-0.9543; 1.3358)	0.33	0.744
Vitamin_B12	0.6582 (-0.1215; 1.4379)	1.65	0.098

Vitamin_B6	-0.9063 (-1.9840; 0.1714)	-1.65	0.0993
Vitamin_C	-0.1671 (-0.8239; 0.4897)	-0.5	0.6181
Vitamin_D	0.5314 (0.2032; 0.8595)	3.17	0.0015
Zinc	0.9479 (0.3295; 1.5662)	3	0.0027
Saffron.ADT	2.7828 (1.3497; 4.2158)	3.81	0.0001

12.2.I-2. Model Comparison for NMA, CNMA and interaction CNMA

	Q	df
Standard model (NMA)	935.93	145
Additive model (CNMA)	1053.45	164
interaction CNMA	1018.82	163

12.2.I-3. Forest plot for combine result from NMA , CNMA and Interaction CNMA

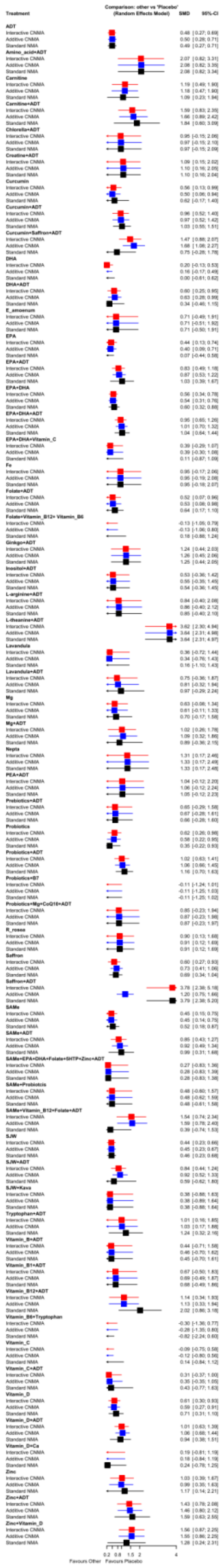

12.2.J.Interaction CNMA for Zinc+ADT

12.2.J-1. Estimates of the incremental standardize mean differences of each component in interaction CNMA

Intervention	iSMD95%-CI	z	p-value
5HTP	-2.0279 (-3.5539; -0.5020)	-2.6	0.0092
ADT	0.4687 (0.1885; 0.7490)	3.28	0.001
Amino_acid	1.587 (0.3396; 2.8343)	2.49	0.0126
B7	-0.6995 (-1.8996; 0.5005)	-1.14	0.2533
Ca	-0.4133 (-1.4310; 0.6045)	-0.8	0.4261
Carnitine	1.1563 (0.4369; 1.8756)	3.15	0.0016
Chlorella	0.4761 (-0.6302; 1.5824)	0.84	0.399
CoQ10	-0.7687 (-2.1298; 0.5923)	-1.11	0.2683
Creatine	0.6074 (-0.3130; 1.5279)	1.29	0.1958
Curcumin	0.4737 (0.0826; 0.8649)	2.37	0.0176
DHA	0.1336 (-0.1592; 0.4264)	0.89	0.3711
E_amoenum	0.6769 (-0.5610; 1.9149)	1.07	0.2838
EPA	0.3756 (0.0908; 0.6604)	2.59	0.0097
Fe	0.9158 (-0.2447; 2.0764)	1.55	0.1219
Folate	0.0352 (-0.3615; 0.4318)	0.17	0.8621
Ginkgo	0.7583 (-0.0219; 1.5385)	1.9	0.0568
Inositol	0.0528 (-0.8266; 0.9322)	0.12	0.9063
Kava	-0.0762 (-1.3652; 1.2129)	-0.12	0.9078
l-arginine	0.3613 (-0.8792; 1.6018)	0.57	0.5681
L-theanine	3.1452 (1.8268; 4.4636)	4.68	0.0001
Lavandula	0.3114 (-0.7805; 1.4033)	0.56	0.5762
Mg	0.5863 (-0.1433; 1.3159)	1.58	0.1152
Nepta	1.3047 (0.1289; 2.4806)	2.17	0.0296
PEA	0.5617 (-0.5988; 1.7221)	0.95	0.3428
Placebo	-0.0295 (-0.2575; 0.1986)	-0.25	0.8001
Prebiotics	0.1703 (-0.7499; 1.0904)	0.36	0.7169
Probiotcis	0.038 (-1.1125; 1.1885)	0.06	0.9484
Probiotics	0.5573 (0.2140; 0.9006)	3.18	0.0015
R_rosea	0.8788 (0.0621; 1.6955)	2.11	0.0349
Saffron	0.7047 (0.3405; 1.0689)	3.79	0.0001
SAMe	0.4172 (0.0780; 0.7563)	2.41	0.0159
SJW	0.4236 (0.1257; 0.7215)	2.79	0.0053
Tryptophan	0.5285 (-0.2894; 1.3465)	1.27	0.2054
Vitamin_B	-0.0395 (-1.1827; 1.1038)	-0.07	0.946
Vitamin_B1	0.1908 (-0.9727; 1.3542)	0.32	0.7479

Vitamin_B12	0.6371 (-0.1554; 1.4295)	1.58	0.1151
Vitamin_B6	-0.8351 (-1.9290; 0.2588)	-1.5	0.1346
Vitamin_C	-0.149 (-0.8166; 0.5186)	-0.44	0.6617
Vitamin_D	0.5615 (0.2281; 0.8949)	3.3	0.001
Zinc	0.853 (0.0104; 1.6956)	1.98	0.0472
Zinc.ADT	0.2498 (-1.0123; 1.5118)	0.39	0.6981

12.2.J-2. Model Comparison for NMA, CNMA and interaction CNMA

	Q	df
Standard model (NMA)	935.93	145
Additive model (CNMA)	1053.45	164
interaction CNMA	1053.18	163

\

12.2.J-3. Forest plot for combine result from NMA , CNMA and Interaction CNMA

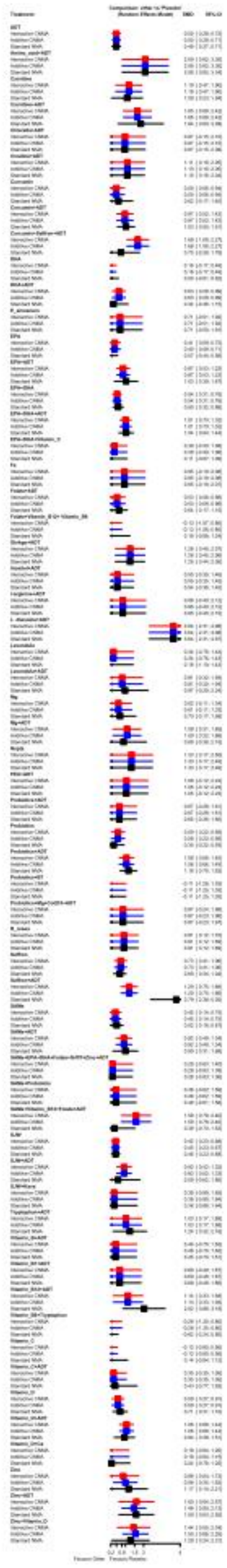

12.2.K.Interaction CNMA for Carnitine+ADT

12.2.K-1. Estimates of the incremental standardize mean differences of each component in interaction CNMA

Intervention	iSMD95%-CI	z	p-value
5HTP	-2.1337 (-3.5414; -0.7260)	-2.97	0.003
ADT	0.4637 (0.1809; 0.7465)	3.21	0.0013
Amino_acid	1.5870 (0.3405; 2.8335)	2.5	0.0126
B7	-0.7008 (-1.9000; 0.4984)	-1.15	0.252
Ca	-0.4138 (-1.4308; 0.6031)	-0.8	0.4251
Carnitine	1.0594 (0.1749; 1.9438)	2.35	0.0189
Chlorella	0.4761 (-0.6292; 1.5814)	0.84	0.3986
CoQ10	-0.7658 (-2.1261; 0.5944)	-1.1	0.2698
Creatine	0.6075 (-0.3123; 1.5274)	1.29	0.1955
Curcumin	0.4734 (0.0826; 0.8643)	2.37	0.0176
DHA	0.1331 (-0.1595; 0.4257)	0.89	0.3728
E_amoenum	0.6750 (-0.5623; 1.9122)	1.07	0.285
EPA	0.3748 (0.0902; 0.6595)	2.58	0.0099
Fe	0.9139 (-0.2460; 2.0737)	1.54	0.1225
Folate	0.0353 (-0.3610; 0.4317)	0.17	0.8613
Ginkgo	0.7583 (-0.0213; 1.5378)	1.91	0.0566
Inositol	0.0528 (-0.8260; 0.9316)	0.12	0.9063
Kava	-0.0748 (-1.3629; 1.2134)	-0.11	0.9094
L-arginine	0.3613 (-0.8783; 1.6009)	0.57	0.5678
L-theanine	3.1452 (1.8276; 4.4627)	4.68	< 0.0001
Lavandula	0.3089 (-0.7825; 1.4002)	0.55	0.5791
Mg	0.5841 (-0.1453; 1.3135)	1.57	0.1165
Nepta	1.2997 (0.1242; 2.4752)	2.17	0.0302
PEA	0.5617 (-0.5978; 1.7212)	0.95	0.3424
Placebo	-0.0314 (-0.2605; 0.1977)	-0.27	0.7881
Prebiotics	0.1702 (-0.7494; 1.0898)	0.36	0.7168
Probiotcis	0.0388 (-1.1107; 1.1882)	0.07	0.9473
Probiotics	0.5566 (0.2135; 0.8997)	3.18	0.0015
R_rosea	0.8760 (0.0594; 1.6926)	2.1	0.0355
Saffron	0.7015 (0.3365; 1.0666)	3.77	0.0002
SAMe	0.4144 (0.0747; 0.7542)	2.39	0.0168
SJW	0.4202 (0.1209; 0.7194)	2.75	0.0059
Tryptophan	0.5282 (-0.2892; 1.3456)	1.27	0.2053
Vitamin_B	-0.0395 (-1.1818; 1.1028)	-0.07	0.946
Vitamin_B1	0.1908 (-0.9718; 1.3533)	0.32	0.7477

Vitamin_B12	0.6385 (-0.1534; 1.4304)	1.58	0.114
Vitamin_B6	-0.8389 (-1.9326; 0.2548)	-1.5	0.1328
Vitamin_C	-0.1497 (-0.8168; 0.5174)	-0.44	0.66
Vitamin_D	0.5606 (0.2273; 0.8939)	3.3	0.001
Zinc	0.9626 (0.3357; 1.5895)	3.01	0.0026
Carnitine.ADT	0.2920 (-1.2255; 1.8095)	0.38	0.706

12.2.K-2. Model Comparison for NMA, CNMA and interaction CNMA

	Q	df
Standard model (NMA)	935.93	145
Additive model (CNMA)	1053.45	164
interaction CNMA	1051.39	163

12.2.K-3. Forest plot for combine result from NMA , CNMA and Interaction CNMA

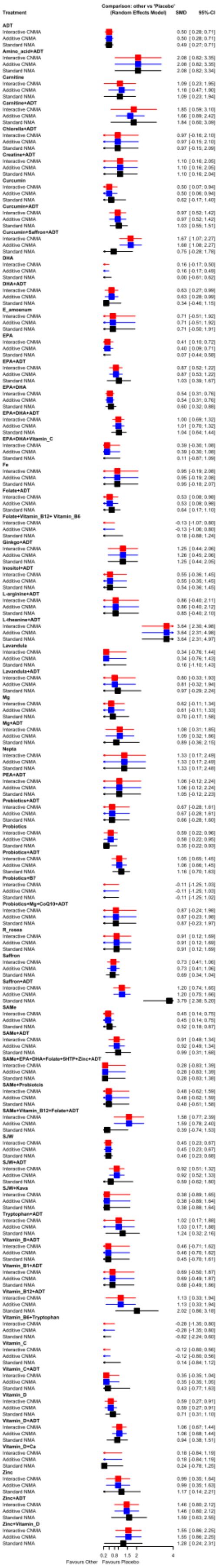

12.2.L.Interaction CNMA for Mg+ADT

12.2.L-1. Estimates of the incremental standardize mean differences of each component in interaction CNMA

Intervention	iSMD95%-CI	z	p-value
5HTP	-2.1543 (-3.5630; -0.7456)	-3	0.0027
ADT	0.4791 (0.1967; 0.7615)	3.33	0.0009
Amino_acid	1.5870 (0.3397; 2.8342)	2.49	0.0126
B7	-0.6929 (-1.8929; 0.5071)	-1.13	0.2577
Ca	-0.4113 (-1.4290; 0.6063)	-0.79	0.4282
Carnitine	1.1631 (0.4436; 1.8827)	3.17	0.0015
Chlorella	0.4761 (-0.6300; 1.5822)	0.84	0.3989
CoQ10	-0.8736 (-2.3375; 0.5903)	-1.17	0.2422
Creatine	0.6075 (-0.3129; 1.5278)	1.29	0.1958
Curcumin	0.4750 (0.0839; 0.8661)	2.38	0.0173
DHA	0.1363 (-0.1565; 0.4291)	0.91	0.3617
E_amoenum	0.6868 (-0.5512; 1.9248)	1.09	0.2769
EPA	0.3791 (0.0942; 0.6639)	2.61	0.0091
Fe	0.9257 (-0.2349; 2.0863)	1.56	0.118
Folate	0.0347 (-0.3619; 0.4313)	0.17	0.8637
Ginkgo	0.7583 (-0.0218; 1.5384)	1.91	0.0568
Inositol	0.0528 (-0.8265; 0.9321)	0.12	0.9063
Kava	-0.0762 (-1.3651; 1.2127)	-0.12	0.9078
L-arginine	0.3613 (-0.8790; 1.6017)	0.57	0.5681
L-theanine	3.1452 (1.8269; 4.4634)	4.68	< 0.0001
Lavandula	0.3166 (-0.7753; 1.4085)	0.57	0.5698
Mg	0.6879 (-0.2134; 1.5891)	1.5	0.1347
Nepta	1.3151 (0.1390; 2.4913)	2.19	0.0284
PEA	0.5617 (-0.5986; 1.7219)	0.95	0.3427
Placebo	-0.0196 (-0.2489; 0.2097)	-0.17	0.8671
Prebiotics	0.1702 (-0.7498; 1.0903)	0.36	0.7168
Probiotcis	0.0401 (-1.1102; 1.1904)	0.07	0.9455
Probiotics	0.5606 (0.2173; 0.9039)	3.2	0.0014
R_rosea	0.8889 (0.0718; 1.7059)	2.13	0.033
Saffron	0.7133 (0.3482; 1.0785)	3.83	0.0001
SAMe	0.4249 (0.0851; 0.7648)	2.45	0.0143
SJW	0.4334 (0.1342; 0.7327)	2.84	0.0045
Tryptophan	0.5279 (-0.2900; 1.3457)	1.27	0.2059
Vitamin_B	-0.0395 (-1.1825; 1.1036)	-0.07	0.946
Vitamin_B1	0.1908 (-0.9725; 1.3540)	0.32	0.7479

Vitamin_B12	0.6338 (-0.1586; 1.4262)	1.57	0.117
Vitamin_B6	-0.8224 (-1.9167; 0.2720)	-1.47	0.1408
Vitamin_C	-0.1456 (-0.8132; 0.5219)	-0.43	0.669
Vitamin_D	0.5675 (0.2339; 0.9011)	3.33	0.0009
Zinc	0.9659 (0.3387; 1.5932)	3.02	0.0025
Mg.ADT	-0.2873 (-1.8217; 1.2471)	-0.37	0.7136

12.2.L-2. Model Comparison for NMA, CNMA and interaction CNMA

	Q	df
Standard model (NMA)	935.93	145
Additive model (CNMA)	1053.45	164
interaction CNMA	1053.34	163

12.2.L-3. Forest plot for combine result from NMA , CNMA and Interaction CNMA

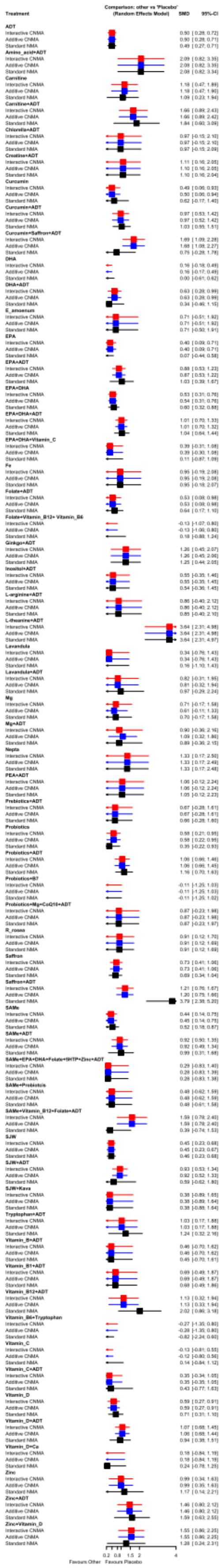

12.2.M.Interaction CNMA for SJW+ADT

12.2.M-1. Estimates of the incremental standardize mean differences of each component in interaction CNMA

Intervention	iSMD95%-CI	z	p-value
5HTP	-2.1636 (-3.5731; -0.7540)	-3.01	0.0026
ADT	0.4863 (0.2020; 0.7705)	3.35	0.0008
Amino_acid	1.5870 (0.3396; 2.8344)	2.49	0.0126
B7	-0.6894 (-1.8897; 0.5109)	-1.13	0.2603
Ca	-0.4103 (-1.4281; 0.6076)	-0.79	0.4295
Carnitine	1.1675 (0.4475; 1.8874)	3.18	0.0015
Chlorella	0.4761 (-0.6302; 1.5824)	0.84	0.399
CoQ10	-0.7846 (-2.1463; 0.5770)	-1.13	0.2587
Creatine	0.6074 (-0.3130; 1.5279)	1.29	0.1959
Curcumin	0.4757 (0.0845; 0.8669)	2.38	0.0172
DHA	0.1377 (-0.1552; 0.4306)	0.92	0.3569
E_amoenum	0.6921 (-0.5463; 1.9305)	1.1	0.2734
EPA	0.3809 (0.0959; 0.6660)	2.62	0.0088
Fe	0.9310 (-0.2301; 2.0920)	1.57	0.1161
Folate	0.0345 (-0.3622; 0.4311)	0.17	0.8648
Ginkgo	0.7583 (-0.0220; 1.5385)	1.9	0.0568
Inositol	0.0528 (-0.8266; 0.9322)	0.12	0.9063
Kava	-0.0863 (-1.3759; 1.2033)	-0.13	0.8956
L-arginine	0.3613 (-0.8792; 1.6018)	0.57	0.5681
L-theanine	3.1452 (1.8267; 4.4636)	4.68	0.0001
Lavandula	0.3202 (-0.7720; 1.4124)	0.57	0.5656
Mg	0.5972 (-0.1329; 1.3273)	1.6	0.1089
Nepta	1.3223 (0.1455; 2.4991)	2.2	0.0276
PEA	0.5617 (-0.5988; 1.7221)	0.95	0.3428
Placebo	-0.0143 (-0.2448; 0.2162)	-0.12	0.9031
Prebiotics	0.1703 (-0.7499; 1.0904)	0.36	0.7169
Probiotcis	0.0406 (-1.1099; 1.1911)	0.07	0.9448
Probiotics	0.5623 (0.2189; 0.9058)	3.21	0.0013
R_rosea	0.8947 (0.0771; 1.7123)	2.14	0.032
Saffron	0.7187 (0.3528; 1.0847)	3.85	0.0001
SAMe	0.4297 (0.0891; 0.7703)	2.47	0.0134
SJW	0.4488 (0.1419; 0.7558)	2.87	0.0042
Tryptophan	0.5278 (-0.2902; 1.3457)	1.26	0.206
Vitamin_B	-0.0395 (-1.1827; 1.1038)	-0.07	0.946
Vitamin_B1	0.1908 (-0.9727; 1.3542)	0.32	0.7479

Vitamin_B12	0.6316 (-0.1610; 1.4242)	1.56	0.1183
Vitamin_B6	-0.8149 (-1.9099; 0.2802)	-1.46	0.1447
Vitamin_C	-0.1438 (-0.8115; 0.5239)	-0.42	0.6729
Vitamin_D	0.5706 (0.2367; 0.9045)	3.35	0.0008
Zinc	0.9674 (0.3400; 1.5948)	3.02	0.0025
SJW.ADT	-0.3530 (-1.5899; 0.8838)	-0.56	0.5759

12.2.M-2. Model Comparison for NMA, CNMA and interaction CNMA

	Q	df
Standard model (NMA)	935.93	145
Additive model (CNMA)	1053.45	164
interaction CNMA	1053.21	163

12.2.M-3. Forest plot for combine result from NMA , CNMA and Interaction CNMA

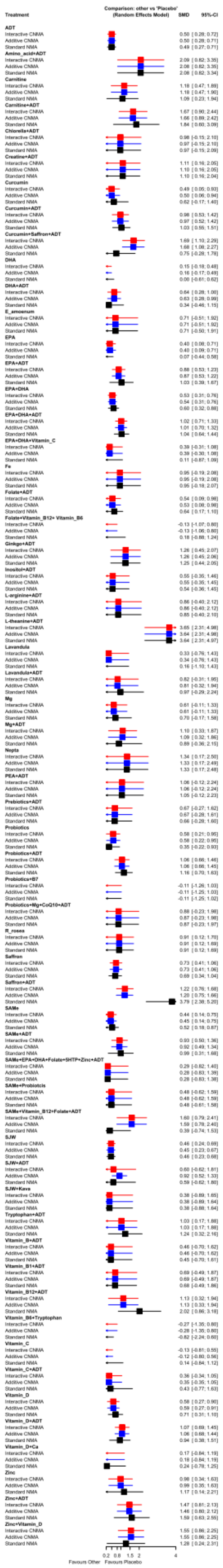

12.2.N.Interaction CNMA for Tryptophan+ADT

12.2.N-1. Estimates of the incremental standardize mean differences of each component in interaction CNMA

Intervention	iSMD95%-CI	z	p-value
5HTP	-2.1434 (-3.5477; -0.7391)	-2.99	0.0028
ADT	0.4685 (0.1893; 0.7476)	3.29	0.001
Amino_acid	1.5870 (0.3422; 2.8317)	2.5	0.0125
B7	-0.6914 (-1.8884; 0.5056)	-1.13	0.2576
Ca	-0.4109 (-1.4262; 0.6044)	-0.79	0.4276
Carnitine	1.1594 (0.4418; 1.8770)	3.17	0.0015
Chlorella	0.4761 (-0.6272; 1.5794)	0.85	0.3977
CoQ10	-0.7771 (-2.1345; 0.5804)	-1.12	0.2619
Creatine	0.6077 (-0.3109; 1.5263)	1.3	0.1947
Curcumin	0.4749 (0.0848; 0.8651)	2.39	0.017
DHA	0.1368 (-0.1552; 0.4289)	0.92	0.3584
E_amoenum	0.6890 (-0.5461; 1.9241)	1.09	0.2742
EPA	0.3796 (0.0955; 0.6636)	2.62	0.0088
Fe	0.9279 (-0.2296; 2.0854)	1.57	0.1161
Folate	0.0131 (-0.3843; 0.4105)	0.06	0.9485
Ginkgo	0.7582 (-0.0199; 1.5363)	1.91	0.0562
Inositol	0.0528 (-0.8247; 0.9303)	0.12	0.9061
Kava	-0.0699 (-1.3563; 1.2166)	-0.11	0.9152
L-arginine	0.3613 (-0.8765; 1.5992)	0.57	0.5673
L-theanine	3.1452 (1.8293; 4.4611)	4.68	0.0001
Lavandula	0.3113 (-0.7783; 1.4009)	0.56	0.5755
Mg	0.5908 (-0.1372; 1.3187)	1.59	0.1117
Nepta	1.3045 (0.1317; 2.4772)	2.18	0.0292
PEA	0.5617 (-0.5959; 1.7193)	0.95	0.3416
Placebo	-0.0174 (-0.2444; 0.2097)	-0.15	0.8807
Prebiotics	0.1700 (-0.7483; 1.0884)	0.36	0.7167
Probiotcis	0.0331 (-1.1143; 1.1806)	0.06	0.9549
Probiotics	0.5612 (0.2188; 0.9036)	3.21	0.0013
R_rosea	0.8874 (0.0728; 1.7021)	2.14	0.0328
Saffron	0.7090 (0.3460; 1.0721)	3.83	0.0001
SAMe	0.4341 (0.0952; 0.7730)	2.51	0.0121
SJW	0.4293 (0.1327; 0.7260)	2.84	0.0046
Tryptophan	-0.5323 (-2.5002; 1.4355)	-0.53	0.596
Vitamin_B	-0.0395 (-1.1798; 1.1009)	-0.07	0.9459
Vitamin_B1	0.1908 (-0.9699; 1.3514)	0.32	0.7473

Vitamin_B12	0.4643 (-0.3776; 1.3062)	1.08	0.2797
Vitamin_B6	-0.3148 (-1.7103; 1.0807)	-0.44	0.6584
Vitamin_C	-0.1449 (-0.8108; 0.5210)	-0.43	0.6698
Vitamin_D	0.5688 (0.2365; 0.9012)	3.35	0.0008
Zinc	0.9664 (0.3405; 1.5923)	3.03	0.0025
Tryptophan.ADT	1.2808 (-0.8819; 3.4434)	1.16	0.2458

12.2.N-2. Model Comparison for NMA, CNMA and interaction CNMA

	Q	df
Standard model (NMA)	935.93	145
Additive model (CNMA)	1053.45	164
interaction CNMA	1048.23	163

12.2.N-3. Forest plot for combine result from NMA , CNMA and Interaction CNMA

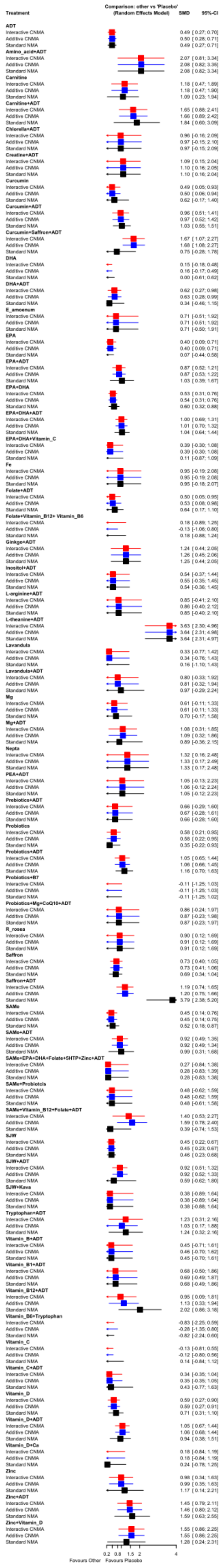

12.2.O.Interaction CNMA for Folate+ADT

12.2.O-1. Estimates of the incremental standardize mean differences of each component in interaction CNMA

Intervention	iSMD95%-CI	z	p-value
5HTP	-0.6329 (-2.6058; 1.3399)	-0.63	0.5295
ADT	0.5017 (0.2230; 0.7805)	3.53	0.0004
Amino_acid	1.5870 (0.3492; 2.8247)	2.51	0.012
B7	-0.6854 (-1.8745; 0.5037)	-1.13	0.2586
Ca	-0.4091 (-1.4179; 0.5996)	-0.79	0.4266
Carnitine	1.1745 (0.4611; 1.8880)	3.23	0.0013
Chlorella	0.4761 (-0.6194; 1.5716)	0.85	0.3943
CoQ10	-0.7927 (-2.1410; 0.5556)	-1.15	0.2492
Creatine	0.6085 (-0.3053; 1.5224)	1.31	0.1919
Curcumin	0.4770 (0.0894; 0.8646)	2.41	0.0159
DHA	0.1391 (-0.1510; 0.4292)	0.94	0.3474
E_amoenum	0.6977 (-0.5302; 1.9255)	1.11	0.2654
EPA	0.3818 (0.0996; 0.6640)	2.65	0.008
Fe	0.9365 (-0.2133; 2.0863)	1.6	0.1104
Folate	-1.5509 (-3.0699; -0.0318)	-2	0.0454
Ginkgo	0.7580 (-0.0146; 1.5305)	1.92	0.0545
Inositol	0.0527 (-0.8198; 0.9253)	0.12	0.9057
Kava	-0.0816 (-1.3610; 1.1978)	-0.13	0.9005
L-arginine	0.3613 (-0.8696; 1.5922)	0.58	0.5651
L-theanine	3.1452 (1.8358; 4.4545)	4.71	0.0001
Lavandula	0.3279 (-0.7557; 1.4116)	0.59	0.5531
Mg	0.6037 (-0.1201; 1.3276)	1.63	0.1021
Nepta	1.3377 (0.1725; 2.5030)	2.25	0.0244
PEA	0.5617 (-0.5885; 1.7118)	0.96	0.3385
Placebo	-0.0087 (-0.2345; 0.2170)	-0.08	0.9395
Prebiotics	0.1695 (-0.7441; 1.0831)	0.36	0.7162
Probiotcis	-0.0053 (-1.1454; 1.1348)	-0.01	0.9928
Probiotics	0.5639 (0.2237; 0.9040)	3.25	0.0012
R_rosea	0.9031 (0.0932; 1.7131)	2.19	0.0288
Saffron	0.7272 (0.3659; 1.0885)	3.94	0.0001
SAMe	0.4812 (0.1404; 0.8219)	2.77	0.0056
SJW	0.4497 (0.1543; 0.7451)	2.98	0.0028
Tryptophan	0.3887 (-0.4333; 1.2107)	0.93	0.354
Vitamin_B	-0.0395 (-1.1723; 1.0933)	-0.07	0.9455
Vitamin_B1	0.1908 (-0.9624; 1.3439)	0.32	0.7458

Vitamin_B12	1.5290 (0.3888; 2.6692)	2.63	0.0086
Vitamin_B6	-0.3164 (-1.4999; 0.8671)	-0.52	0.6003
Vitamin_C	-0.1419 (-0.8035; 0.5196)	-0.42	0.6741
Vitamin_D	0.5740 (0.2438; 0.9042)	3.41	0.0007
Zinc	0.9683 (0.3461; 1.5906)	3.05	0.0023
Folate.ADT	1.6994 (0.1268; 3.2721)	2.12	0.0342

12.2.O-2. Model Comparison for NMA, CNMA and interaction CNMA

	Q	df
Standard model (NMA)	935.93	145
Additive model (CNMA)	1053.45	164
interaction CNMA	1031.10	163

12.2.O-3. Forest plot for combine result from NMA , CNMA and Interaction CNMA

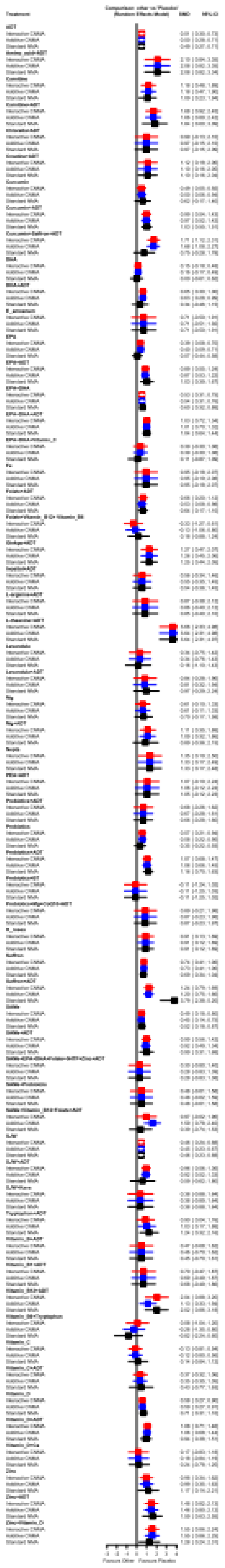

12.2.P.Interaction CNMA for SAMe+Vitamin_B12+Folate+ADT

12.2.P-1. Estimates of the incremental standardize mean differences of each component in interaction CNMA

Intervention	iSMD95%-CI	z	p-value
5HTP	-2.4004 (-3.7977; -1.0032)	-3.37	0.0008
ADT	0.5065 (0.2297; 0.7833)	3.59	0.0003
Amino_acid	1.5870 (0.3551; 2.8188)	2.53	0.0116
B7	-0.6713 (-1.8536; 0.5111)	-1.11	0.2658
Ca	-0.4048 (-1.4079; 0.5982)	-0.79	0.4289
Carnitine	1.1819 (0.4721; 1.8917)	3.26	0.0011
Chlorella	0.4761 (-0.6127; 1.5648)	0.86	0.3914
CoQ10	-0.8086 (-2.1490; 0.5318)	-1.18	0.2371
Creatine	0.6092 (-0.3006; 1.5190)	1.31	0.1894
Curcumin	0.4793 (0.0940; 0.8647)	2.44	0.0148
DHA	0.1447 (-0.1438; 0.4331)	0.98	0.3257
E_amoenum	0.7185 (-0.5033; 1.9402)	1.15	0.2491
EPA	0.3884 (0.1077; 0.6691)	2.71	0.0067
Fe	0.9574 (-0.1859; 2.1007)	1.64	0.1007
Folate	0.1507 (-0.2477; 0.5490)	0.74	0.4585
Ginkgo	0.7578 (-0.0100; 1.5255)	1.93	0.053
Inositol	0.0527 (-0.8156; 0.9210)	0.12	0.9053
Kava	-0.0733 (-1.3467; 1.2002)	-0.11	0.9102
L-arginine	0.3613 (-0.8636; 1.5862)	0.58	0.5632
L-theanine	3.1452 (1.8415; 4.4489)	4.73	< 0.0001
Lavandula	0.3304 (-0.7480; 1.4087)	0.6	0.5482
Mg	0.6129 (-0.1074; 1.3332)	1.67	0.0954
Nepta	1.3426 (0.1841; 2.5011)	2.27	0.0231
PEA	0.5617 (-0.5821; 1.7054)	0.96	0.3358
Placebo	0.0121 (-0.2133; 0.2375)	0.11	0.9162
Prebiotics	0.1690 (-0.7405; 1.0785)	0.36	0.7158
Probiotcis	-0.0328 (-1.1663; 1.1006)	-0.06	0.9547
Probiotics	0.5706 (0.2323; 0.9089)	3.31	0.0009
R_rosea	0.9195 (0.1136; 1.7253)	2.24	0.0253
Saffron	0.7370 (0.3775; 1.0965)	4.02	< 0.0001
SAMe	0.5296 (0.1875; 0.8716)	3.03	0.0024
SJW	0.4622 (0.1683; 0.7561)	3.08	0.0021
Tryptophan	0.7377 (-0.0823; 1.5576)	1.76	0.0779
Vitamin_B	-0.0395 (-1.1657; 1.0868)	-0.07	0.9452
Vitamin_B1	0.1908 (-0.9560; 1.3375)	0.33	0.7444

Vitamin_B12	1.5465 (0.5585; 2.5345)	3.07	0.0022
Vitamin_B6	-1.5202 (-2.6927; -0.3476)	-2.54	0.0111
Vitamin_C	-0.1348 (-0.7926; 0.5230)	-0.4	0.688
Vitamin_D	0.5863 (0.2577; 0.9148)	3.5	0.0005
Zinc	0.9737 (0.3545; 1.5929)	3.08	0.0021
SAMe.Vitamin_B12.Folate.ADT	-2.3229 (-3.8665; -0.7793)	-2.95	0.0032

12.2.P-2. Model Comparison for NMA, CNMA and interaction CNMA

	Q	df
Standard model (NMA)	935.93	145
Additive model (CNMA)	1053.45	164
interaction CNMA	1018.64	163

12.2.P-3. Forest plot for combine result from NMA, CNMA and Interaction CNMA

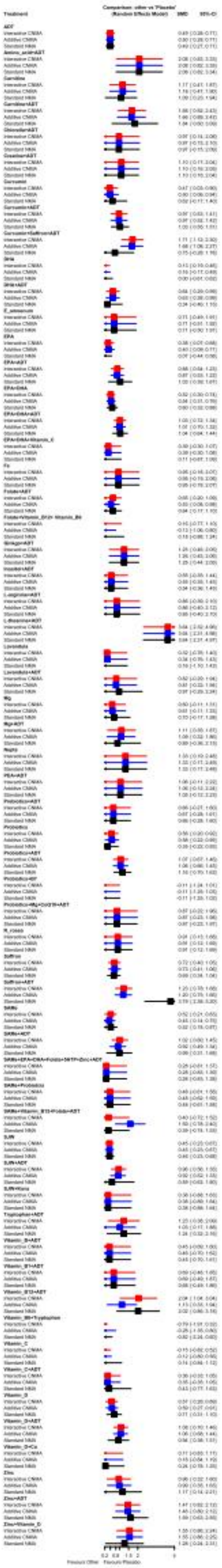

12.2.Q.Interaction CNMA for EPA+DHA+Vitamin_C

12.2.Q-1. Estimates of the incremental standardize mean differences of each component in interaction CNMA

5HTP	-2.2673 (-3.6912; -0.8434)	-3.12	0.0018
5HTP	-2.1772 (-3.5874; -0.7670)	-3.03	0.0025
ADT	0.4798 (0.1994; 0.7601)	3.35	0.0008
Amino_acid	1.5870 (0.3391; 2.8348)	2.49	0.0127
B7	-0.6903 (-1.8910; 0.5103)	-1.13	0.2598
Ca	-0.4105 (-1.4287; 0.6077)	-0.79	0.4294
Carnitine	1.1645 (0.4449; 1.8842)	3.17	0.0015
Chlorella	0.4761 (-0.6307; 1.5829)	0.84	0.3992
CoQ10	-0.7818 (-2.1435; 0.5800)	-1.13	0.2605
Creatine	0.6074 (-0.3134; 1.5281)	1.29	0.196
Curcumin	0.4753 (0.0840; 0.8667)	2.38	0.0173
DHA	0.1475 (-0.1465; 0.4416)	0.98	0.3254
E_amoenum	0.6907 (-0.5477; 1.9291)	1.09	0.2743
EPA	0.3878 (0.1021; 0.6735)	2.66	0.0078
Fe	0.9296 (-0.2315; 2.0907)	1.57	0.1166
Folate	0.0347 (-0.3621; 0.4316)	0.17	0.8638
Ginkgo	0.7583 (-0.0223; 1.5389)	1.9	0.0569
Inositol	0.0528 (-0.8269; 0.9325)	0.12	0.9063
Kava	-0.0745 (-1.3640; 1.2150)	-0.11	0.9099
L-arginine	0.3613 (-0.8797; 1.6023)	0.57	0.5682
L-theanine	3.1452 (1.8263; 4.4640)	4.67	< 0.0001
Lavandula	0.3169 (-0.7754; 1.4093)	0.57	0.5696
Mg	0.5948 (-0.1351; 1.3246)	1.6	0.1103
Nepta	1.3158 (0.1394; 2.4921)	2.19	0.0284
PEA	0.5617 (-0.5993; 1.7226)	0.95	0.343
Placebo	-0.0157 (-0.2438; 0.2125)	-0.13	0.8929
Prebiotics	0.1703 (-0.7502; 1.0908)	0.36	0.7169
Probiotcis	0.0422 (-1.1088; 1.1933)	0.07	0.9427
Probiotics	0.5619 (0.2185; 0.9053)	3.21	0.0013
R_rosea	0.8918 (0.0748; 1.7089)	2.14	0.0324
Saffron	0.7152 (0.3508; 1.0796)	3.85	0.0001
SAMe	0.4267 (0.0874; 0.7660)	2.47	0.0137
SJW	0.4357 (0.1376; 0.7337)	2.87	0.0042
Tryptophan	0.5272 (-0.2911; 1.3454)	1.26	0.2067
Vitamin_B	-0.0395 (-1.1832; 1.1043)	-0.07	0.9461
Vitamin_B1	0.1908 (-0.9732; 1.3547)	0.32	0.748

Vitamin_B12	0.6335 (-0.1593; 1.4263)	1.57	0.1173
Vitamin_B6	-0.8194 (-1.9137; 0.2750)	-1.47	0.1423
Vitamin_C	0.0343 (-0.7318; 0.8004)	0.09	0.9301
Vitamin_D	0.5698 (0.2363; 0.9033)	3.35	0.0008
Zinc	0.9670 (0.3395; 1.5946)	3.02	0.0025
EPA.DHA.Vitamin_C	-0.5192 (-1.5905; 0.5521)	-0.95	0.3422

12.2.Q-2. Model Comparison for NMA, CNMA and interaction CNMA

	Q	df
Standard model (NMA)	935.93	145
Additive model (CNMA)	1053.45	164
interaction CNMA	1052.35	163

12.2.Q-3. Forest plot for combine result from NMA , CNMA and Interaction CNMA

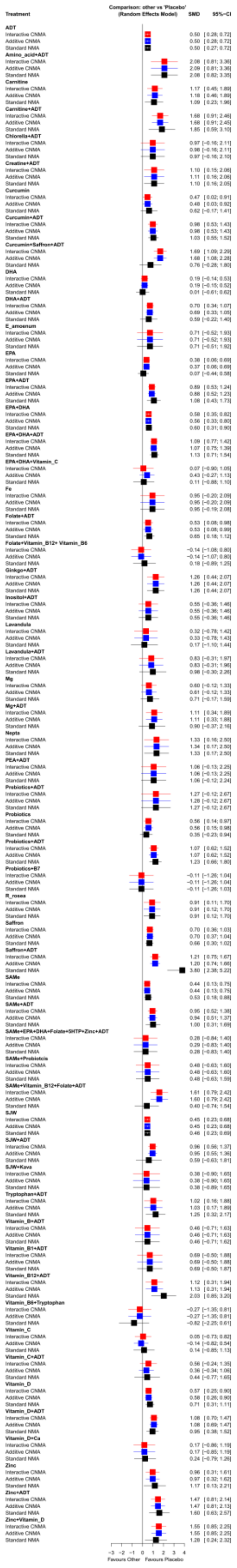

12.2.R.Interaction CNMA for Curcumin+Saffron+ADT

12.2.R-1. Estimates of the incremental standardize mean differences of each component in interaction CNMA

Intervention	iSMD95%-CI	z	p-value
5HTP	-2.2282 (-3.6310; -0.8255)	-3.11	0.0018
ADT	0.5368 (0.2517; 0.8219)	3.69	0.0002
Amino_acid	1.5870 (0.3450; 2.8289)	2.5	0.0123
B7	-0.6637 (-1.8579; 0.5306)	-1.09	0.276
Ca	-0.4023 (-1.4151; 0.6104)	-0.78	0.4362
Carnitine	1.1979 (0.4810; 1.9147)	3.27	0.0011
Chlorella	0.4761 (-0.6241; 1.5763)	0.85	0.3964
CoQ10	-0.8263 (-2.1810; 0.5284)	-1.2	0.2319
Creatine	0.6080 (-0.3087; 1.5248)	1.3	0.1936
Curcumin	0.5591 (0.1619; 0.9562)	2.76	0.0058
DHA	0.1480 (-0.1435; 0.4395)	0.99	0.3198
E_amoenum	0.7303 (-0.5027; 1.9633)	1.16	0.2457
EPA	0.3937 (0.1100; 0.6774)	2.72	0.0065
Fe	0.9692 (-0.1861; 2.1245)	1.64	0.1001
Folate	0.0323 (-0.3623; 0.4270)	0.16	0.8724
Ginkgo	0.7581 (-0.0178; 1.5340)	1.92	0.0555
Inositol	0.0528 (-0.8228; 0.9283)	0.12	0.906
Kava	-0.0819 (-1.3656; 1.2017)	-0.13	0.9005
L-arginine	0.3613 (-0.8738; 1.5964)	0.57	0.5664
L-theanine	3.1452 (1.8319; 4.4585)	4.69	0.0001
Lavandula	0.3455 (-0.7421; 1.4332)	0.62	0.5335
Mg	0.6264 (-0.1008; 1.3535)	1.69	0.0913
Nepta	1.3728 (0.2016; 2.5441)	2.3	0.0216
PEA	0.5617 (-0.5930; 1.7163)	0.95	0.3404
Placebo	0.0239 (-0.2069; 0.2547)	0.2	0.8391
Prebiotics	0.1698 (-0.7466; 1.0863)	0.36	0.7165
Probiotcis	0.0452 (-1.0990; 1.1895)	0.08	0.9382
Probiotics	0.5748 (0.2330; 0.9166)	3.3	0.001
R_rosea	0.9364 (0.1222; 1.7507)	2.25	0.0242
Saffron	0.8619 (0.4716; 1.2522)	4.33	0.0001
SAMe	0.4633 (0.1236; 0.8030)	2.67	0.0075
SJW	0.4827 (0.1822; 0.7831)	3.15	0.0016
Tryptophan	0.5265 (-0.2880; 1.3411)	1.27	0.2052
Vitamin_B	-0.0395 (-1.1768; 1.0979)	-0.07	0.9458
Vitamin_B1	0.1908 (-0.9669; 1.3484)	0.32	0.7467
Vitamin_B12	0.6157 (-0.1729; 1.4044)	1.53	0.126
Vitamin_B6	-0.7593 (-1.8497; 0.3311)	-1.36	0.1723

Vitamin_C	-0.1307 (-0.7950; 0.5337)	-0.39	0.6998
Vitamin_D	0.5931 (0.2605; 0.9256)	3.5	0.0005
Zinc	0.9776 (0.3530; 1.6022)	3.07	0.0022
Curcumin.Saffron.ADT	-1.1495 (-2.2295; -0.0695)	-2.09	0.037

12.2.R-2. Model Comparison for NMA, CNMA and interaction CNMA

	Q	df
Standard model (NMA)	935.93	145
Additive model (CNMA)	1053.45	164
interaction CNMA	1040.27	163

12.2.R-3. Forest plot for combine result from NMA , CNMA and Interaction CNMA

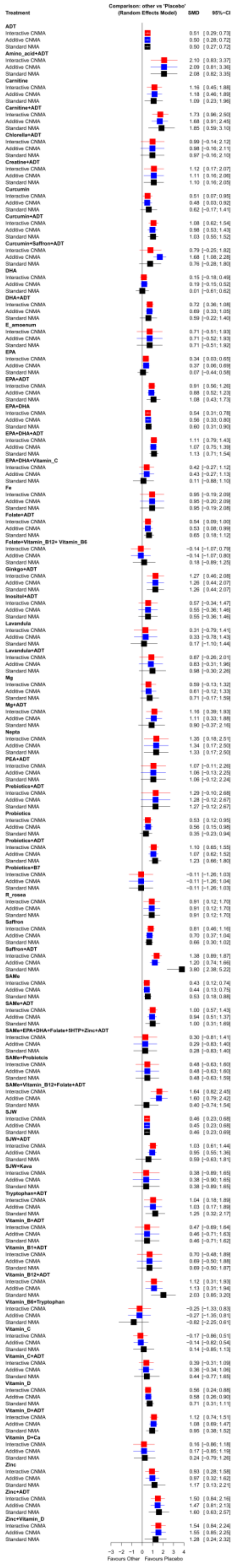

13.Subgroup analysis and Sensitivity analysis

13.1. Subgroup analysis for only adjunctive nutraceuticals

13.1-A. Network Plot for only adjunctive nutraceuticals

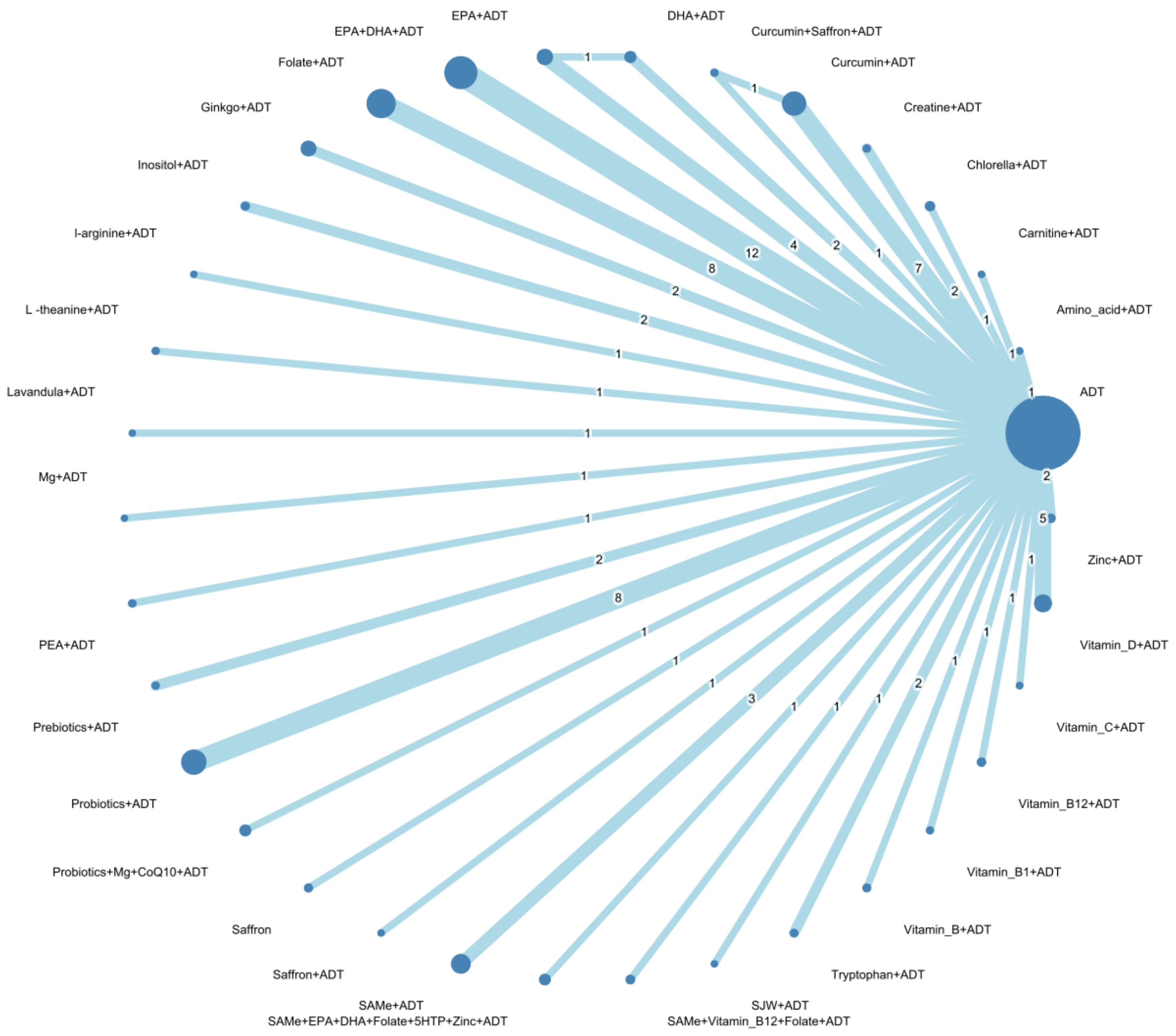

13.1-B. Forest Plot for only adjunctive nutraceuticals

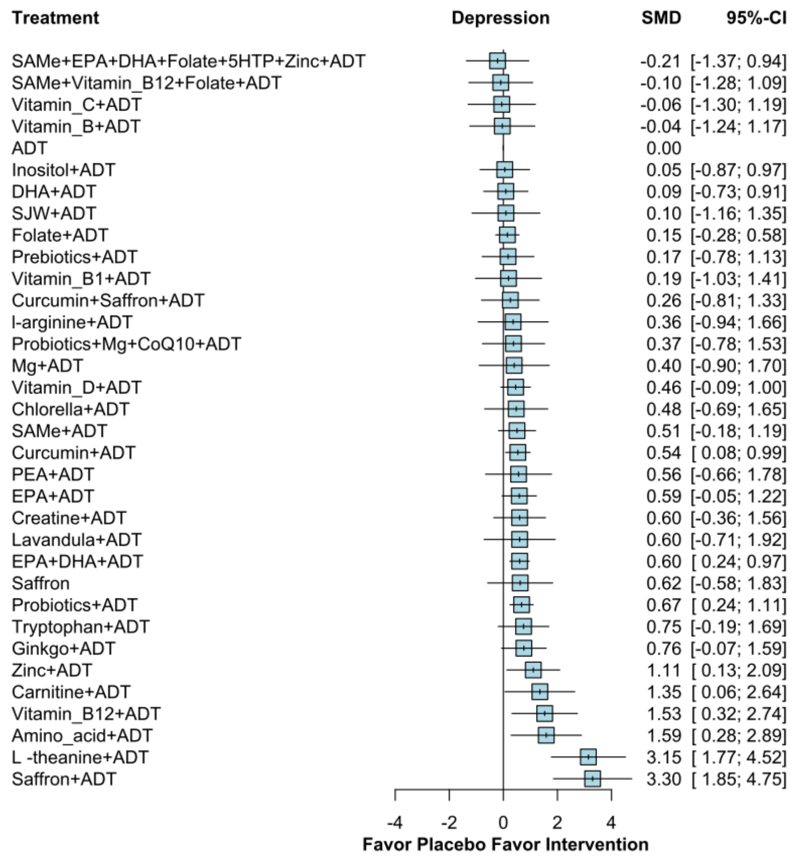

13.1-C. Detailed result for only adjunctive nutraceuticals

Intervention	SMD95%-CI	p-value
ADT	.	.
Amino_acid+ADT	1.587 (0.2827; 2.8912)	0.0171
Carnitine+ADT	1.3514 (0.0600; 2.6428)	0.0403
Chlorella+ADT	0.4761 (-0.6939; 1.6461)	0.4252
Creatine+ADT	0.6015 (-0.3580; 1.5609)	0.2192
Curcumin+ADT	0.536 (0.0798; 0.9923)	0.0213
Curcumin+Saffron+ADT	0.2589 (-0.8099; 1.3276)	0.635
DHA+ADT	0.0907 (-0.7294; 0.9108)	0.8283
EPA+ADT	0.5887 (-0.0460; 1.2233)	0.0691
EPA+DHA+ADT	0.6025 (0.2388; 0.9662)	0.0012
Folate+ADT	0.1499 (-0.2827; 0.5825)	0.497
Ginkgo+ADT	0.7599 (-0.0655; 1.5854)	0.0712
Inositol+ADT	0.0532 (-0.8666; 0.9729)	0.9098
l-arginine+ADT	0.3613 (-0.9363; 1.6590)	0.5853
L-theanine+ADT	3.1452 (1.7729; 4.5175)	< 0.0001
Lavandula+ADT	0.6021 (-0.7136; 1.9179)	0.3697
Mg+ADT	0.4006 (-0.8985; 1.6996)	0.5456
PEA+ADT	0.5617 (-0.6597; 1.7830)	0.3674
Prebiotics+ADT	0.1746 (-0.7845; 1.1337)	0.7212
Probiotics+ADT	0.6743 (0.2430; 1.1055)	0.0022
Probiotics+Mg+CoQ10+ADT	0.3749 (-0.7768; 1.5265)	0.5235
Saffron	0.6224 (-0.5807; 1.8256)	0.3106
Saffron+ADT	3.3016 (1.8513; 4.7520)	< 0.0001
SAMe+ADT	0.5055 (-0.1823; 1.1933)	0.1497
SAMe+EPA+DHA+Folate+5HTP+Zinc+ADT	-0.2134 (-1.3678; 0.9410)	0.7171
SAMe+Vitamin_B12+Folate+ADT	-0.0962 (-1.2787; 1.0864)	0.8733
SJW+ADT	0.0958 (-1.1614; 1.3530)	0.8813
Tryptophan+ADT	0.7486 (-0.1898; 1.6870)	0.1179
Vitamin_B+ADT	-0.0395 (-1.2445; 1.1655)	0.9488
Vitamin_B1+ADT	0.1908 (-1.0334; 1.4150)	0.7601
Vitamin_B12+ADT	1.529 (0.3170; 2.7410)	0.0134
Vitamin_C+ADT	-0.0585 (-1.3050; 1.1879)	0.9267
Vitamin_D+ADT	0.4551 (-0.0920; 1.0022)	0.103
Zinc+ADT	1.1081 (0.1279; 2.0882)	0.0267

13.2. Subgroup analysis for only nutraceuticals monotherapy

13.2-A. Network Plot for only nutraceuticals monotherapy

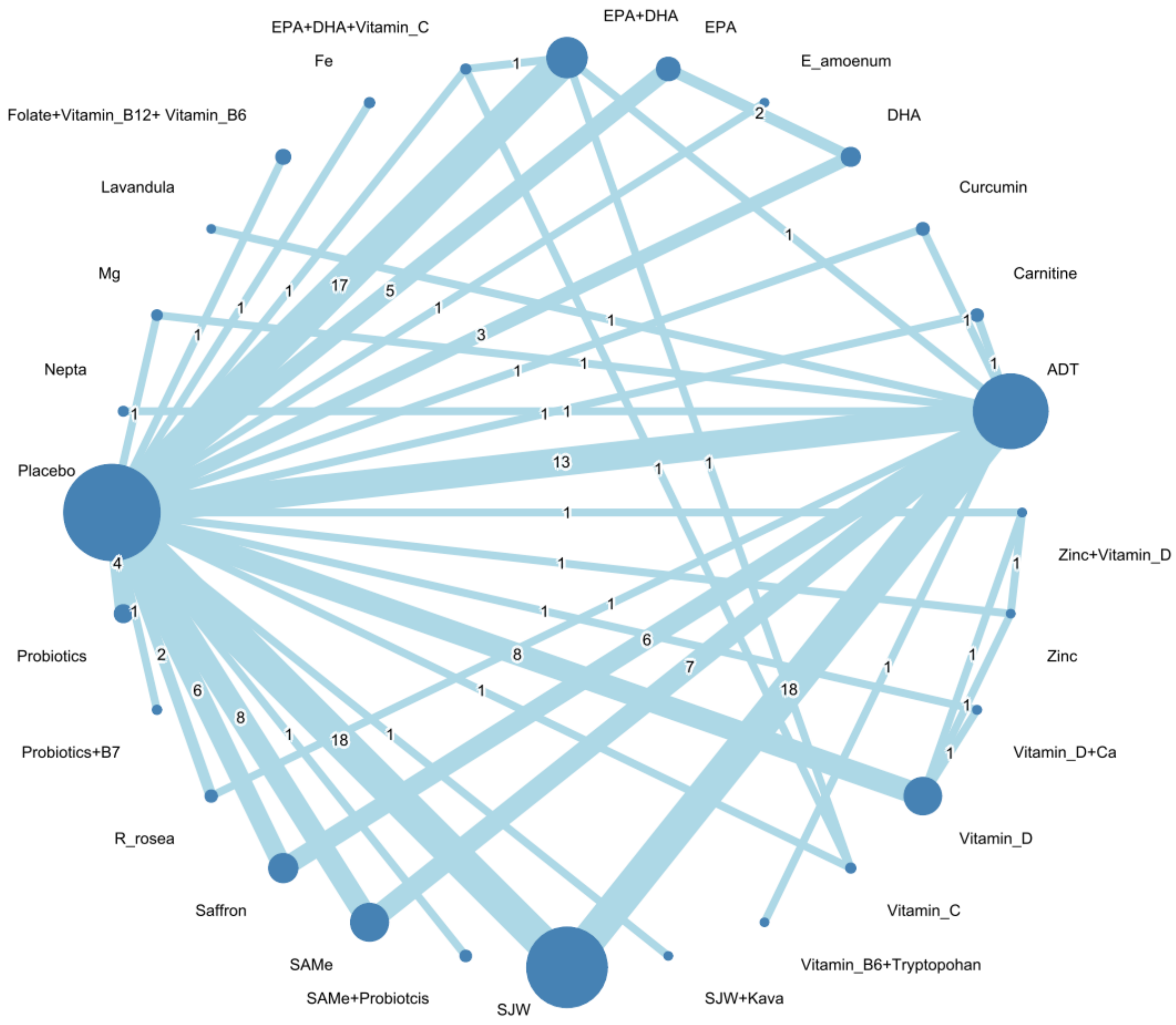

13.2-B. Forest Plot for only nutraceuticals monotherapy

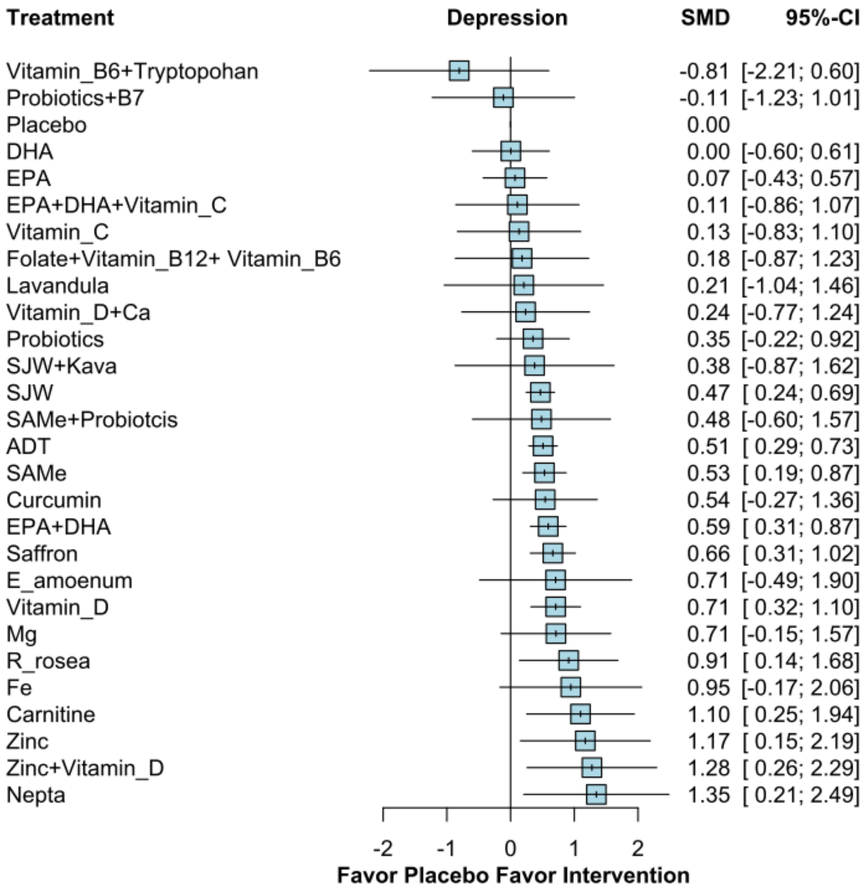

13.2-C. Detailed result for only nutraceuticals monotherapy

Intervention	SMD95%-CI	p-value
ADT	0.5098(0.2856; 0.7340)	< 0.0001
Carnitine	1.0978(0.2500; 1.9456)	0.0112
Curcumin	0.5431 (-0.2765; 1.3627)	0.1941
DHA	0.0041 (-0.6027; 0.6108)	0.9895
E_amoenum	0.7064 (-0.4904; 1.9032)	0.2473
EPA	0.0698 (-0.4317; 0.5712)	0.7851
EPA+DHA	0.5902 (0.3002; 0.8802)	< 0.0001
EPA+DHA+Vitamin_C	0.1067 (-0.8643; 1.0777)	0.8295
Fe	0.9453 (-0.1713; 2.0618)	0.0971
Folate+Vitamin_B12+ Vitamin_B6	0.1801 (-0.8704; 1.2305)	0.7369
Lavandula	0.2078 (-1.0456; 1.4612)	0.7453
Mg	0.7120 (-0.1510; 1.5749)	0.1059
Nepta	1.3458 (0.2030; 2.4887)	0.021
Placebo	.	.
Probiotics	0.3520 (-0.2190; 0.9230)	0.227
Probiotics+B7	-0.1128 (-1.2338; 1.0082)	0.8437
R_rosea	0.9118 (0.1369; 1.6867)	0.0211
Saffron	0.6631 (0.3079; 1.0183)	0.0003
SAMe	0.5321 (0.1902; 0.8740)	0.0023
SAMe+Probiotics	0.4846 (-0.6018; 1.5711)	0.382
SJW	0.4677 (0.2440; 0.6914)	< 0.0001
SJW+Kava	0.3768 (-0.8738; 1.6275)	0.5548
Vitamin_B6+Tryptophan	-0.8057 (-2.2155; 0.6040)	0.2626
Vitamin_C	0.1327 (-0.8383; 1.1036)	0.7889
Vitamin_D	0.7079 (0.3170; 1.0989)	0.0004
Vitamin_D+Ca	0.2359 (-0.7700; 1.2419)	0.6457
Zinc	1.1724 (0.1501; 2.1946)	0.0246
Zinc+Vitamin_D	1.2765 (0.2556; 2.2974)	0.0143

13.3. Subgroup analysis for clinical population

13.3-A. Network Plot for clinical population

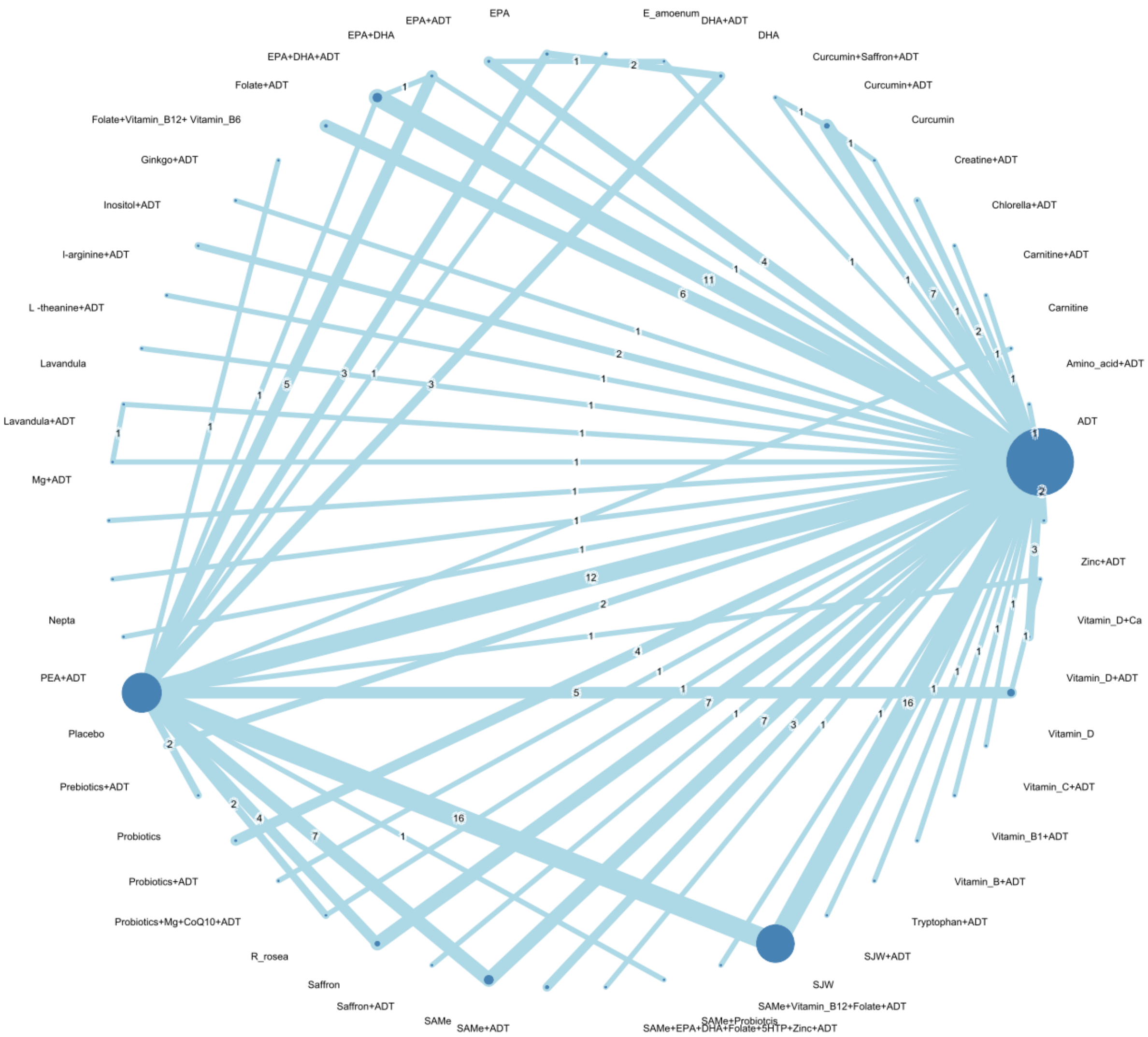

13.3-B. Forest Plot for clinical population

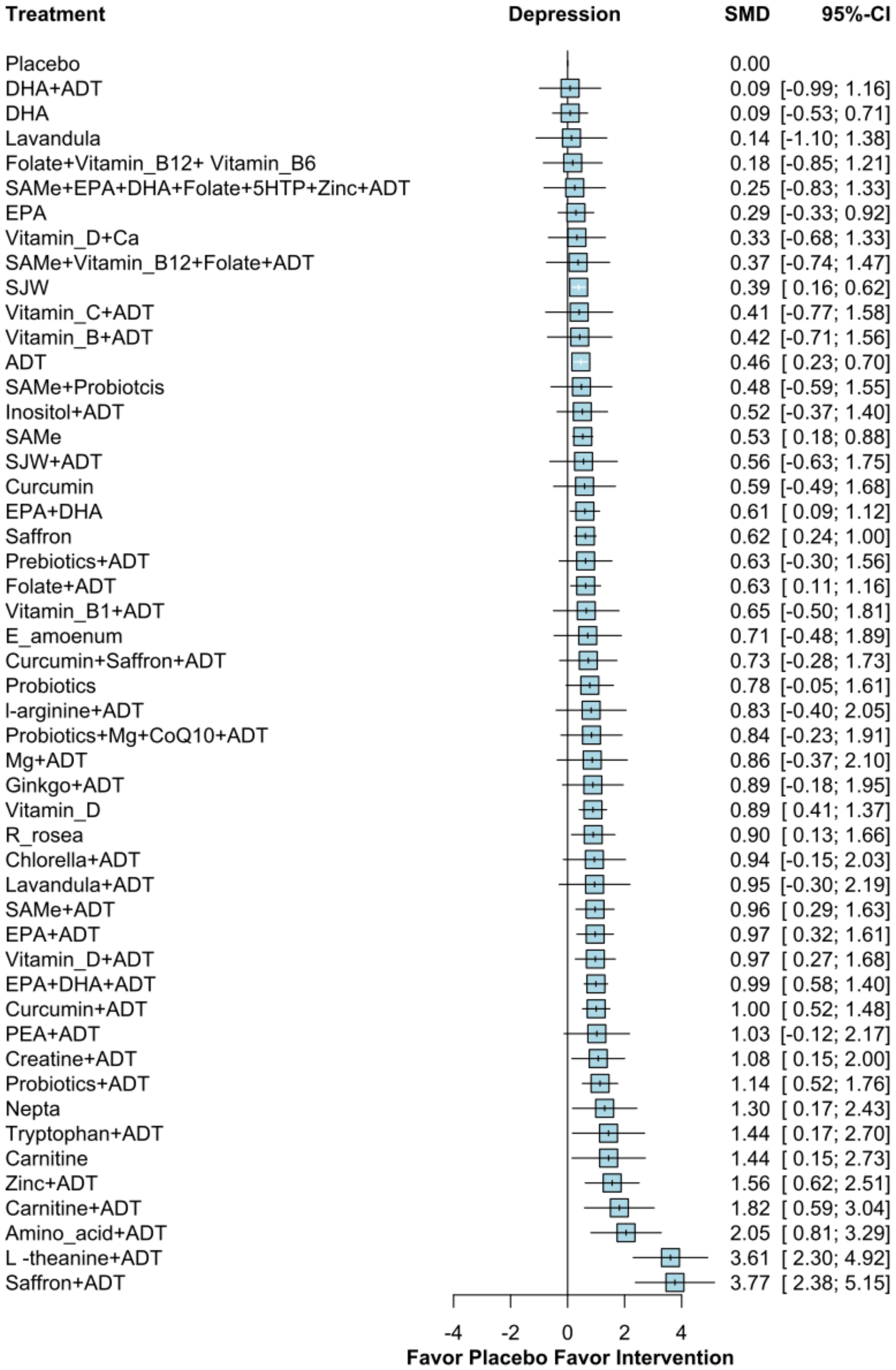

13.3-C. Detailed result for clinical population

Intervention	SMD95%-CI	p-value
ADT	0.4639 (0.2279; 0.6999)	0.0001
Amino_acid+ADT	2.0509 (0.8149; 3.2869)	0.0011
Carnitine	1.4393 (0.1518; 2.7268)	0.0284
Carnitine+ADT	1.8154 (0.5929; 3.0378)	0.0036
Chlorella+ADT	0.9400 (-0.1534; 2.0334)	0.092
Creatine+ADT	1.0753 (0.1478; 2.0029)	0.0231
Curcumin	0.5944 (-0.4900; 1.6788)	0.2827
Curcumin+ADT	1.0025 (0.5219; 1.4830)	< 0.0001
Curcumin+Saffron+ADT	0.7250 (-0.2838; 1.7339)	0.159
DHA	0.0898 (-0.5260; 0.7057)	0.7749
DHA+ADT	0.0877 (-0.9890; 1.1644)	0.8732
E_amoenum	0.7064 (-0.4753; 1.8881)	0.2413
EPA	0.2929 (-0.3329; 0.9186)	0.359
EPA+ADT	0.9666 (0.3232; 1.6100)	0.0032
EPA+DHA	0.6063 (0.0888; 1.1239)	0.0217
EPA+DHA+ADT	0.9927 (0.5820; 1.4035)	< 0.0001
Folate+ADT	0.6339 (0.1088; 1.1590)	0.018
Folate+Vitamin_B12+ Vitamin_B6	0.1801 (-0.8532; 1.2133)	0.7327
Ginkgo+ADT	0.8861 (-0.1807; 1.9529)	0.1035
Inositol+ADT	0.5165 (-0.3706; 1.4036)	0.2538
l-arginine+ADT	0.8253 (-0.4038; 2.0543)	0.1882
L-theanine+ADT	3.6091 (2.3015; 4.9167)	< 0.0001
Lavandula	0.1363 (-1.1044; 1.3769)	0.8296
Lavandula+ADT	0.9469 (-0.2960; 2.1897)	0.1354
Mg+ADT	0.8645 (-0.3660; 2.0950)	0.1685
Nepta	1.3000 (0.1705; 2.4294)	0.0241
PEA+ADT	1.0256 (-0.1226; 2.1738)	0.08
Prebiotics+ADT	0.6313 (-0.2960; 1.5586)	0.1821
Probiotics	0.7782 (-0.0527; 1.6091)	0.0664
Probiotics+ADT	1.1380 (0.5167; 1.7592)	0.0003
Probiotics+Mg+CoQ10+ADT	0.8388 (-0.2349; 1.9126)	0.1257
R_rosea	0.8988 (0.1336; 1.6640)	0.0213
Saffron	0.6230 (0.2441; 1.0019)	0.0013
Saffron+ADT	3.7656 (2.3763; 5.1548)	< 0.0001
SAMe	0.5317 (0.1804; 0.8829)	0.003
SAMe+ADT	0.9627 (0.2908; 1.6347)	0.005
SAMe+EPA+DHA+Folate+5HTP+Zinc+ADT	0.2505 (-0.8262; 1.3272)	0.6483

SAMe+Probiotics	0.4846 (-0.5852; 1.5545)	0.3746
SAMe+Vitamin_B12+Folate+ADT	0.3678 (-0.7391; 1.4746)	0.5149
SJW	0.3876 (0.1551; 0.6201)	0.0011
SJW+ADT	0.5598 (-0.6265; 1.7460)	0.355
Tryptophan+ADT	1.4357 (0.1690; 2.7023)	0.0263
Vitamin_B+ADT	0.4245 (-0.7063; 1.5553)	0.4619
Vitamin_B1+ADT	0.6547 (-0.4965; 1.8059)	0.265
Vitamin_C+ADT	0.4054 (-0.7694; 1.5802)	0.4988
Vitamin_D	0.8889 (0.4057; 1.3722)	0.0003
Vitamin_D+ADT	0.9726 (0.2695; 1.6756)	0.0067
Vitamin_D+Ca	0.3261 (-0.6764; 1.3286)	0.5238
Zinc+ADT	1.5632 (0.6183; 2.5082)	0.0012

13.4. Subgroup analysis for non-clinical population

13.4-A. Network Plot for non-clinical population

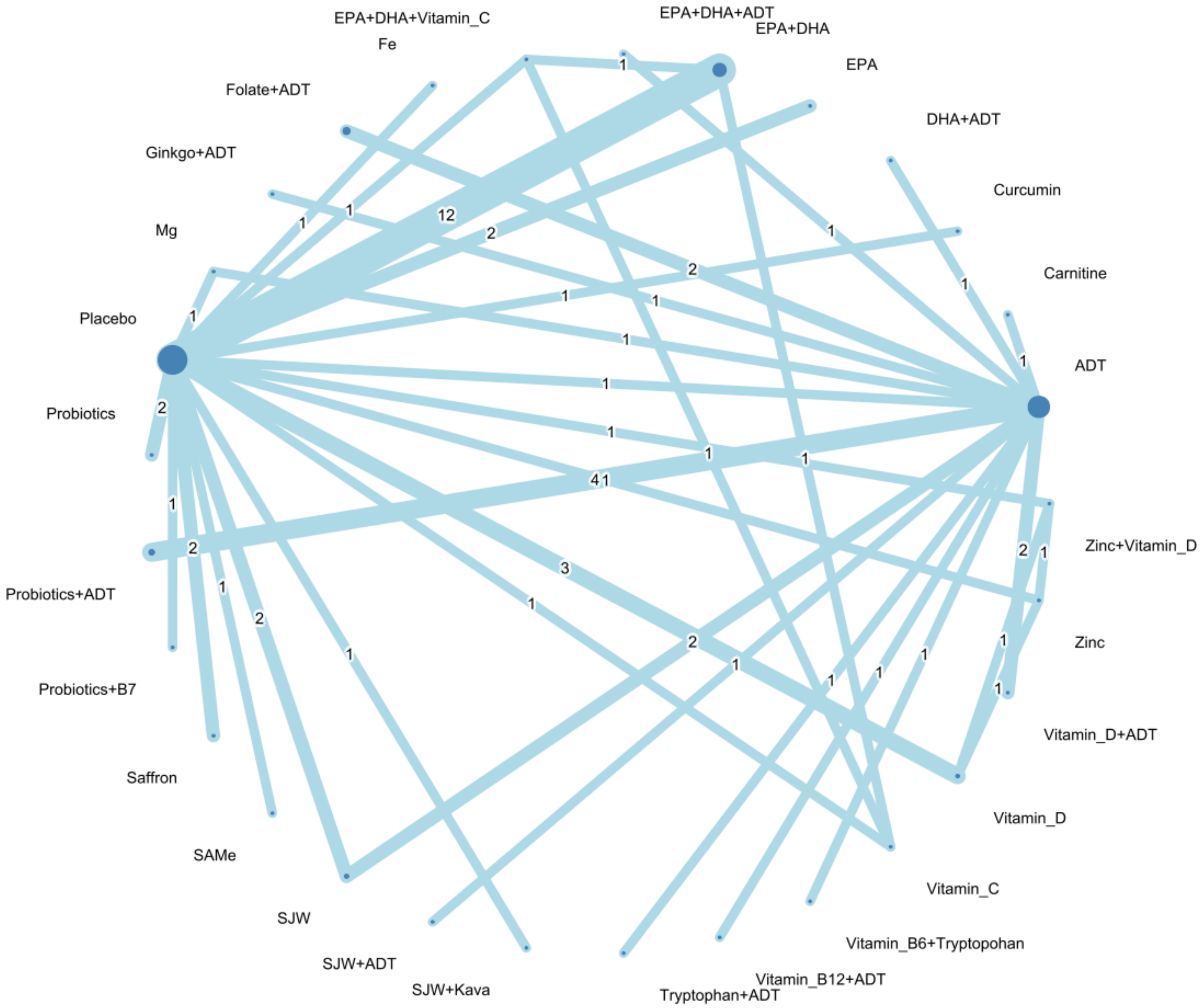

13.4-B. Forest Plot for non-clinical population

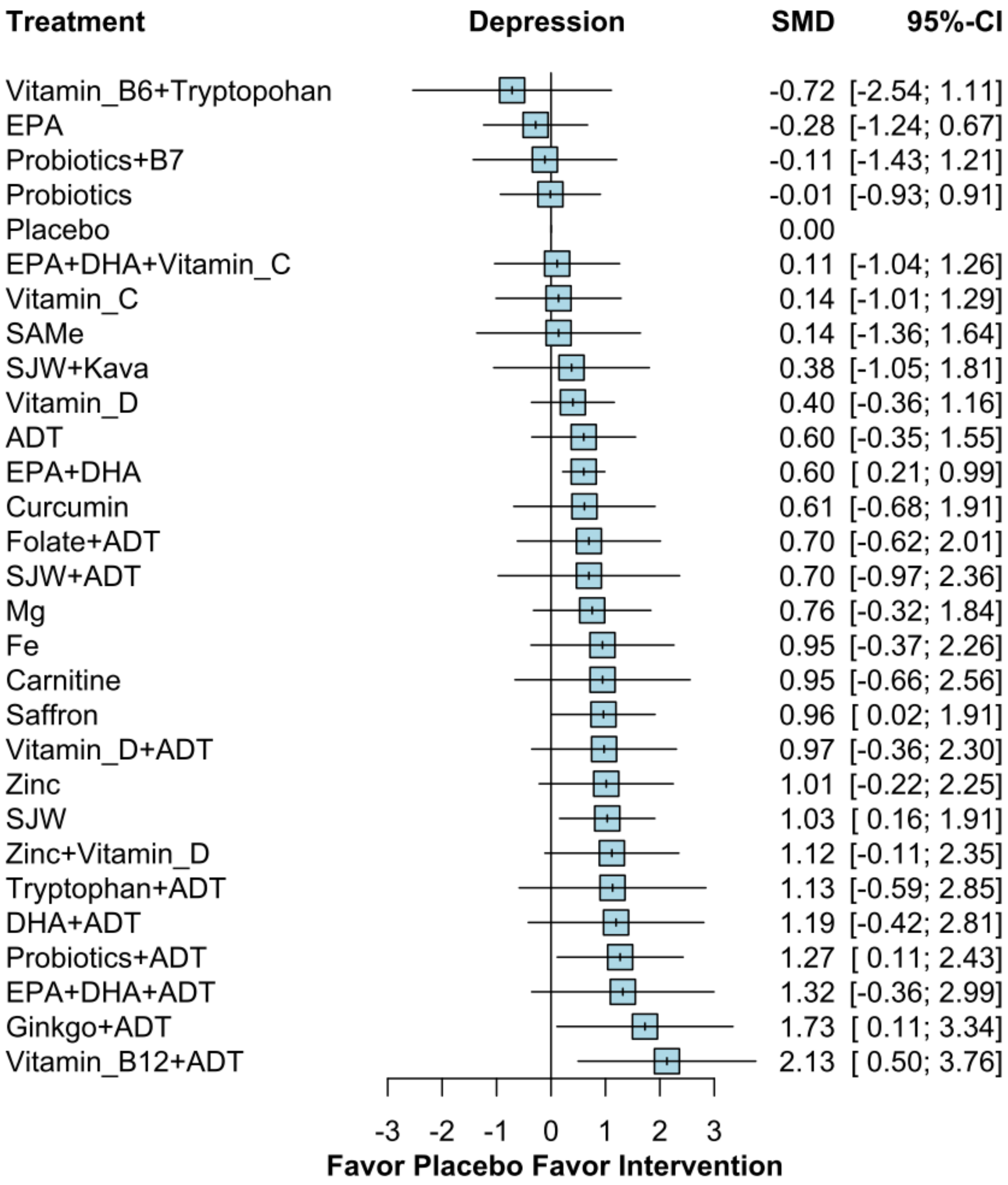

13.4-C. Detailed result for non-clinical population

Intervention	SMD95%-CI	p-value
ADT	0.6005 (-0.3535; 1.5545)	0.2173
Carnitine	0.9469 (-0.6622; 2.5561)	0.2488
Curcumin	0.6136 (-0.6846; 1.9118)	0.3543
DHA+ADT	1.1949 (-0.4185; 2.8083)	0.1466
EPA	-0.2832 (-1.2388; 0.6725)	0.5614
EPA+DHA	0.6012 (0.2132; 0.9891)	0.0024
EPA+DHA+ADT	1.3194 (-0.3561; 2.9948)	0.1227
EPA+DHA+Vitamin_C	0.1122 (-1.0370; 1.2614)	0.8483
Fe	0.9453 (-0.3694; 2.2599)	0.1587
Folate+ADT	0.6961 (-0.6191; 2.0114)	0.2996
Ginkgo+ADT	1.7258 (0.1081; 3.3434)	0.0365
Mg	0.7561 (-0.3234; 1.8356)	0.1698
Placebo	.	.
Probiotics	-0.0115 (-0.9307; 0.9077)	0.9805
Probiotics+ADT	1.2702 (0.1128; 2.4276)	0.0315
Probiotics+B7	-0.1128 (-1.4312; 1.2056)	0.8668
Saffron	0.9639 (0.0156; 1.9121)	0.0463
SAMe	0.1391 (-1.3650; 1.6431)	0.8562
SJW	1.0329 (0.1576; 1.9083)	0.0207
SJW+ADT	0.6963 (-0.9707; 2.3633)	0.413
SJW+Kava	0.3768 (-1.0534; 1.8071)	0.6056
Tryptophan+ADT	1.1301 (-0.5855; 2.8458)	0.1967
Vitamin_B12+ADT	2.1295 (0.4963; 3.7627)	0.0106
Vitamin_B6+Tryptopohan	-0.7151 (-2.5396; 1.1094)	0.4424
Vitamin_C	0.1381 (-1.0110; 1.2873)	0.8138
Vitamin_D	0.4016 (-0.3597; 1.1629)	0.3011
Vitamin_D+ADT	0.9746 (-0.3553; 2.3045)	0.1509
Zinc	1.0142 (-0.2179; 2.2463)	0.1067
Zinc+Vitamin_D	1.1181 (-0.1130; 2.3492)	0.0751

13.5. Subgroup analysis of using SSRIs as ADT

13.5-A. Network Plot for subgroup analysis of using SSRIs as ADT

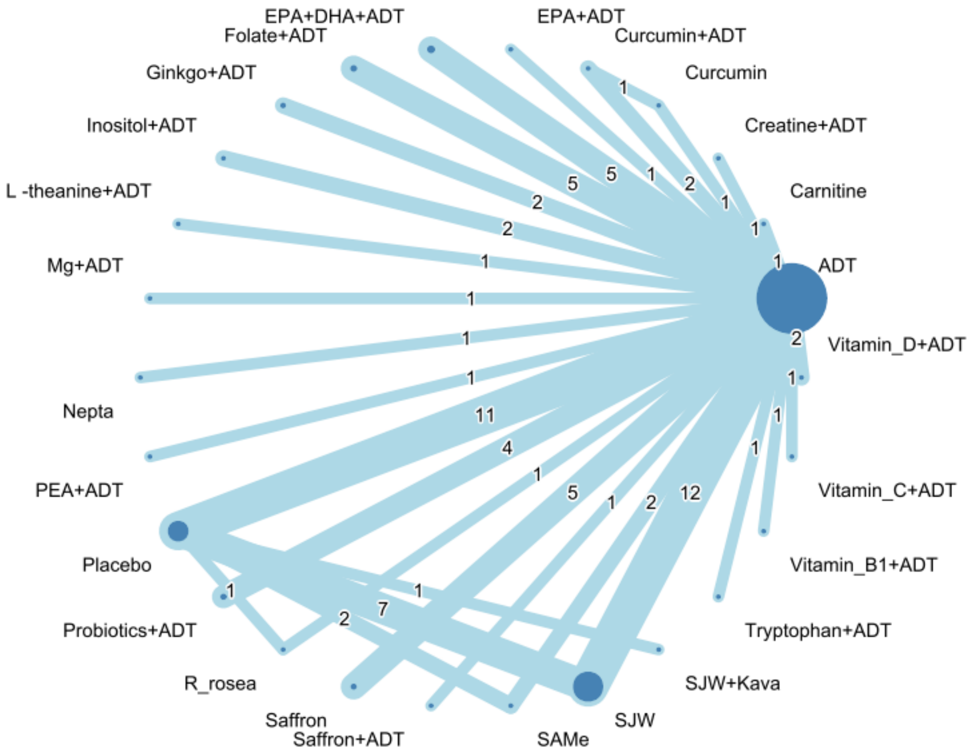

13.5-B. Forest Plot for subgroup analysis of using SSRIs as ADT

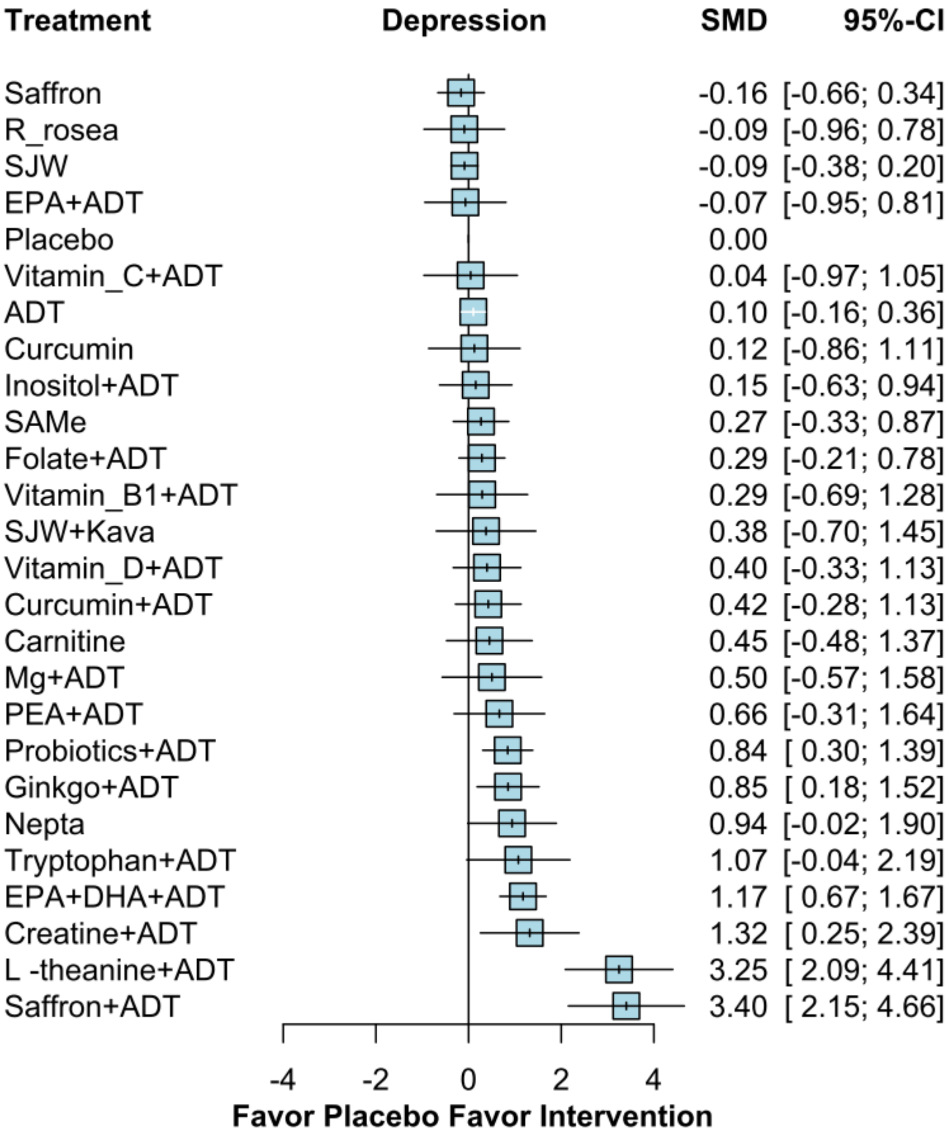

13.5-C. Detailed result for subgroup analysis of using SSRIs as ADT

Intervention	SMD95%-CI	p-value
ADT	0.1029 (-0.1574; 0.3631)	0.4386
Carnitine	0.4493 (-0.4762; 1.3749)	0.3414
Creatine+ADT	1.3198 (0.2544; 2.3852)	0.0152
Curcumin	0.1245 (-0.8628; 1.1118)	0.8048
Curcumin+ADT	0.4249 (-0.2842; 1.1340)	0.2402
EPA+ADT	-0.0688 (-0.9455; 0.8080)	0.8779
EPA+DHA+ADT	1.1749 (0.6749; 1.6750)	< 0.0001
Folate+ADT	0.2876 (-0.2084; 0.7835)	0.2558
Ginkgo+ADT	0.8519 (0.1831; 1.5207)	0.0125
Inositol+ADT	0.1539 (-0.6283; 0.9361)	0.6998
L-theanine+ADT	3.2480 (2.0866; 4.4094)	< 0.0001
Mg+ADT	0.5034 (-0.5705; 1.5773)	0.3582
Nepta	0.9389 (-0.0175; 1.8953)	0.0544
PEA+ADT	0.6645 (-0.3139; 1.6429)	0.1831
Placebo	.	.
Probiotics+ADT	0.8446 (0.3029; 1.3863)	0.0022
R_rosea	-0.0924 (-0.9637; 0.7790)	0.8354
Saffron	-0.1621 (-0.6643; 0.3401)	0.527
Saffron+ADT	3.4045 (2.1518; 4.6571)	< 0.0001
SAMe	0.2685 (-0.3306; 0.8675)	0.3797
SJW	-0.0883 (-0.3789; 0.2024)	0.5517
SJW+Kava	0.3768 (-0.6979; 1.4516)	0.4919
Tryptophan+ADT	1.0746 (-0.0405; 2.1897)	0.0589
Vitamin_B1+ADT	0.2936 (-0.6884; 1.2756)	0.5579
Vitamin_C+ADT	0.0443 (-0.9653; 1.0539)	0.9314
Vitamin_D+ADT	0.3978 (-0.3324; 1.1280)	0.2856

13.6. Subgroup analysis of using fluoxetine as ADT

13.6-A. Network Plot for subgroup analysis of using fluoxetine as ADT

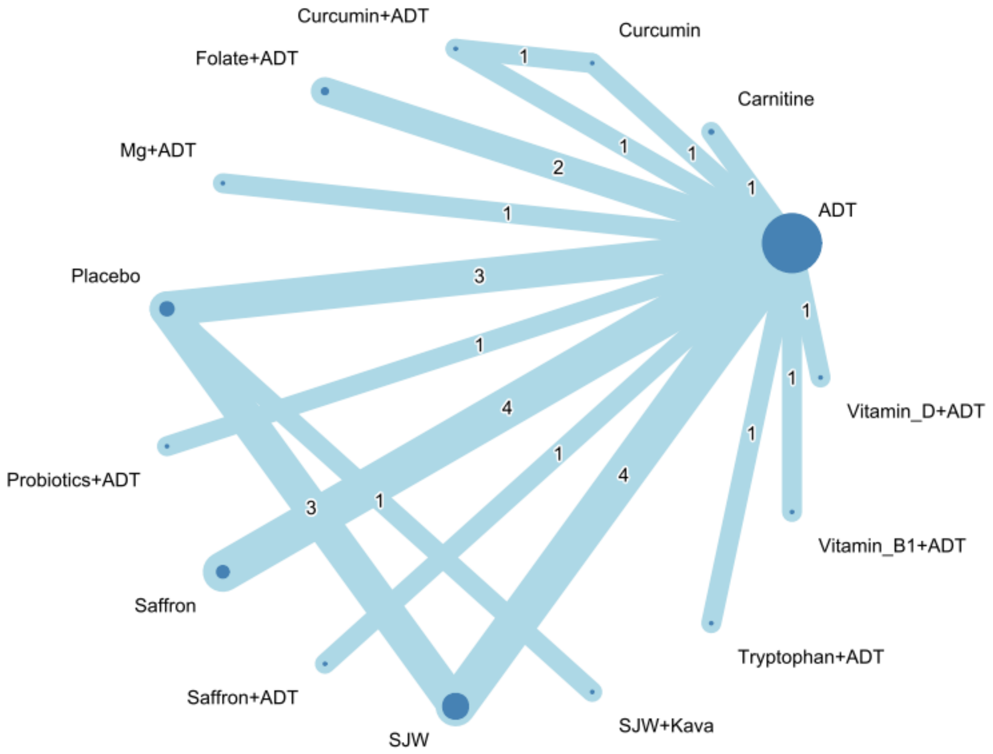

13.6-B. Forest Plot for subgroup analysis of using fluoxetine as ADT

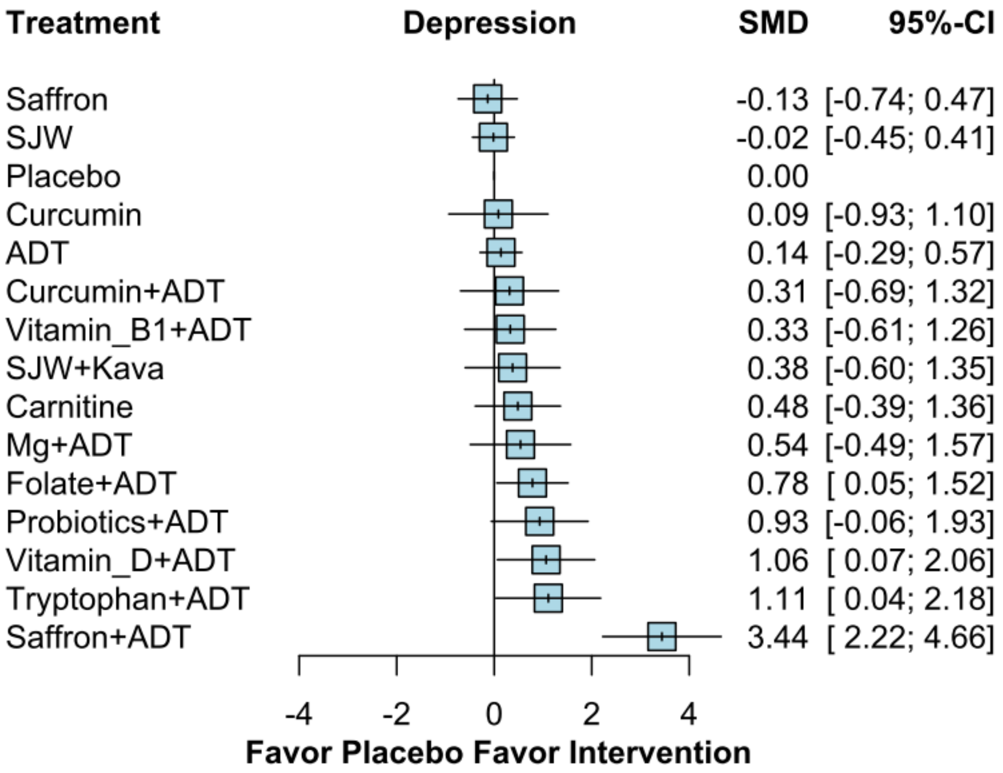

13.6-C. Detailed result for subgroup analysis of using fluoxetine as ADT

Intervention	SMD95%-CI	p-value
ADT	0.1382 (-0.2929; 0.5693)	0.5298
Carnitine	0.4847 (-0.3907; 1.3600)	0.2779
Curcumin	0.0856 (-0.9329; 1.1042)	0.8691
Curcumin+ADT	0.3134 (-0.6924; 1.3193)	0.5414
Folate+ADT	0.7841 (0.0525; 1.5156)	0.0357
Mg+ADT	0.5388 (-0.4922; 1.5697)	0.3057
Placebo	.	.
Probiotics+ADT	0.9328 (-0.0600; 1.9256)	0.0655
Saffron	-0.1339 (-0.7413; 0.4735)	0.6657
Saffron+ADT	3.4398 (2.2238; 4.6559)	< 0.0001
SJW	-0.0168 (-0.4469; 0.4134)	0.9391
SJW+Kava	0.3768 (-0.5961; 1.3498)	0.4478
Tryptophan+ADT	1.1099 (0.0361; 2.1838)	0.0428
Vitamin_B1+ADT	0.3290 (-0.6059; 1.2638)	0.4904
Vitamin_D+ADT	1.0640 (0.0658; 2.0623)	0.0367

13.7. Sensitivity analysis for excluding only female subjects

13.7-A. Network Plot for excluding only female subjects

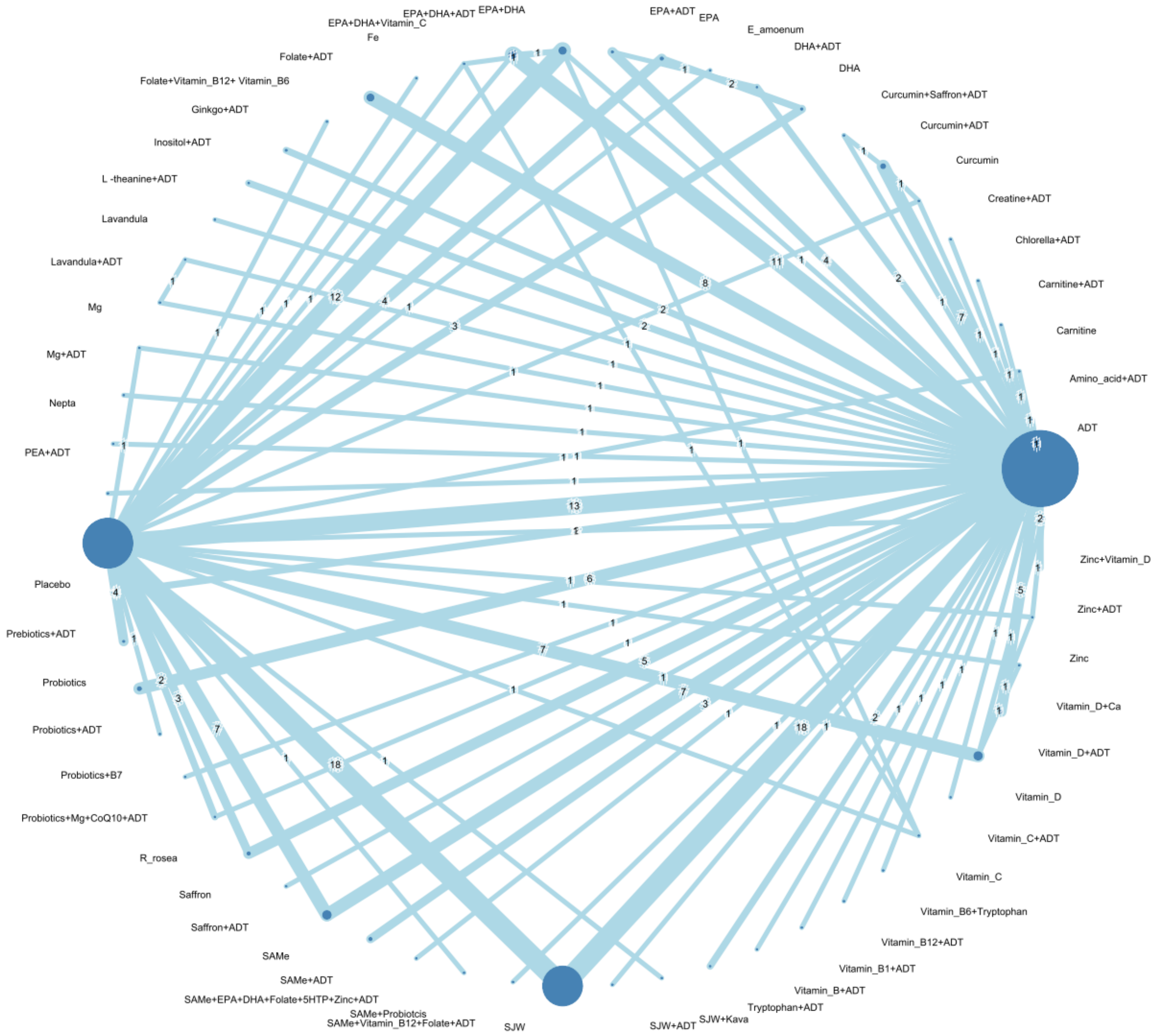

13.7-B. Forest Plot for excluding only female subjects

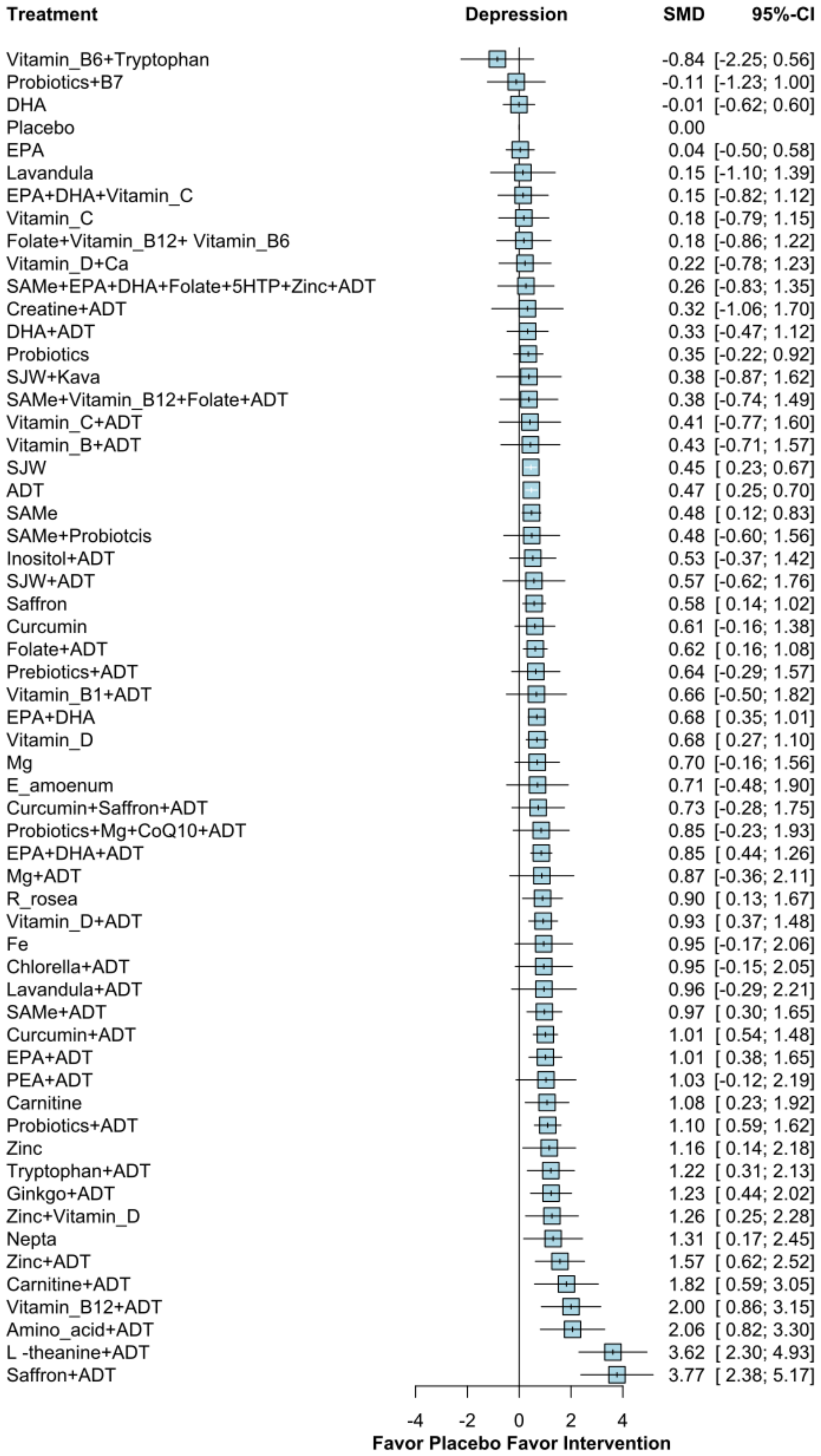

13.7-C. Detailed result for excluding only female subjects

Intervention	SMD95%-CI	p-value
ADT	0.4733 (0.2459; 0.7006)	< 0.0001
Amino_acid+ADT	2.0602 (0.8168; 3.3036)	0.0012
Carnitine	1.0759 (0.2318; 1.9199)	0.0125
Carnitine+ADT	1.8247 (0.5947; 3.0546)	0.0036
Chlorella+ADT	0.9493 (-0.1525; 2.0511)	0.0913
Creatine+ADT	0.3182 (-1.0592; 1.6956)	0.6507
Curcumin	0.6085 (-0.1631; 1.3800)	0.1222
Curcumin+ADT	1.0119 (0.5399; 1.4839)	< 0.0001
Curcumin+Saffron+ADT	0.7343 (-0.2798; 1.7484)	0.1559
DHA	-0.0078 (-0.6169; 0.6014)	0.9801
DHA+ADT	0.3260 (-0.4671; 1.1192)	0.4204
E_amoenum	0.7064 (-0.4847; 1.8975)	0.2451
EPA	0.0395 (-0.5042; 0.5833)	0.8867
EPA+ADT	1.0131 (0.3786; 1.6476)	0.0018
EPA+DHA	0.6815 (0.3506; 1.0124)	< 0.0001
EPA+DHA+ADT	0.8526 (0.4447; 1.2606)	< 0.0001
EPA+DHA+Vitamin_C	0.1522 (-0.8169; 1.1213)	0.7582
Fe	0.9453 (-0.1652; 2.0558)	0.0953
Folate+ADT	0.6215 (0.1606; 1.0824)	0.0082
Folate+Vitamin_B12+Vitamin_B6	0.1801 (-0.8640; 1.2241)	0.7354
Ginkgo+ADT	1.2307 (0.4373; 2.0242)	0.0024
Inositol+ADT	0.5259 (-0.3653; 1.4170)	0.2474
L-theanine+ADT	3.6184 (2.3038; 4.9330)	< 0.0001
Lavandula	0.1456 (-1.1025; 1.3936)	0.8192
Lavandula+ADT	0.9562 (-0.2941; 2.2064)	0.1339
Mg	0.6959 (-0.1633; 1.5550)	0.1124
Mg+ADT	0.8738 (-0.3642; 2.1118)	0.1665
Nepta	1.3093 (0.1717; 2.4469)	0.0241
PEA+ADT	1.0349 (-0.1212; 2.1911)	0.0794
Placebo	.	.
Prebiotics+ADT	0.6414 (-0.2898; 1.5727)	0.177
Probiotics	0.3516 (-0.2164; 0.9197)	0.225
Probiotics+ADT	1.1019 (0.5873; 1.6165)	< 0.0001
Probiotics+B7	-0.1128 (-1.2278; 1.0022)	0.8428
Probiotics+Mg+CoQ10+ADT	0.8481 (-0.2342; 1.9304)	0.1246
R_rosea	0.9014 (0.1301; 1.6727)	0.022
Saffron	0.5777 (0.1362; 1.0192)	0.0103

Saffron+ADT	3.7749 (2.3790; 5.1708)	< 0.0001
SAMe	0.4767 (0.1194; 0.8340)	0.0089
SAMe+ADT	0.9728 (0.2982; 1.6474)	0.0047
SAMe+EPA+DHA+Folate+5HTP+Zinc+ADT	0.2598 (-0.8254; 1.3451)	0.6389
SAMe+Probiotics	0.4846 (-0.5956; 1.5649)	0.3793
SAMe+Vitamin_B12+Folate+ADT	0.3771 (-0.7380; 1.4922)	0.5075
SJW	0.4494 (0.2259; 0.6729)	< 0.0001
SJW+ADT	0.5691 (-0.6249; 1.7630)	0.3502
SJW+Kava	0.3768 (-0.8684; 1.6221)	0.5531
Tryptophan+ADT	1.2216 (0.3111; 2.1321)	0.0085
Vitamin_B+ADT	0.4338 (-0.7051; 1.5727)	0.4554
Vitamin_B1+ADT	0.6640 (-0.4952; 1.8232)	0.2616
Vitamin_B12+ADT	2.0023 (0.8560; 3.1485)	0.0006
Vitamin_B6+Tryptophan	-0.8423 (-2.2478; 0.5632)	0.2402
Vitamin_C	0.1782 (-0.7908; 1.1472)	0.7185
Vitamin_C+ADT	0.4147 (-0.7679; 1.5974)	0.4919
Vitamin_D	0.6841 (0.2703; 1.0978)	0.0012
Vitamin_D+ADT	0.9263 (0.3699; 1.4826)	0.0011
Vitamin_D+Ca	0.2241 (-0.7791; 1.2272)	0.6615
Zinc	1.1600 (0.1403; 2.1798)	0.0258
Zinc+ADT	1.5735 (0.6242; 2.5228)	0.0012
Zinc+Vitamin_D	1.2641 (0.2457; 2.2825)	0.015

13.8. Sensitivity analysis for participants aged less than 60

13.8-A. Network Plot for participants aged less than 60

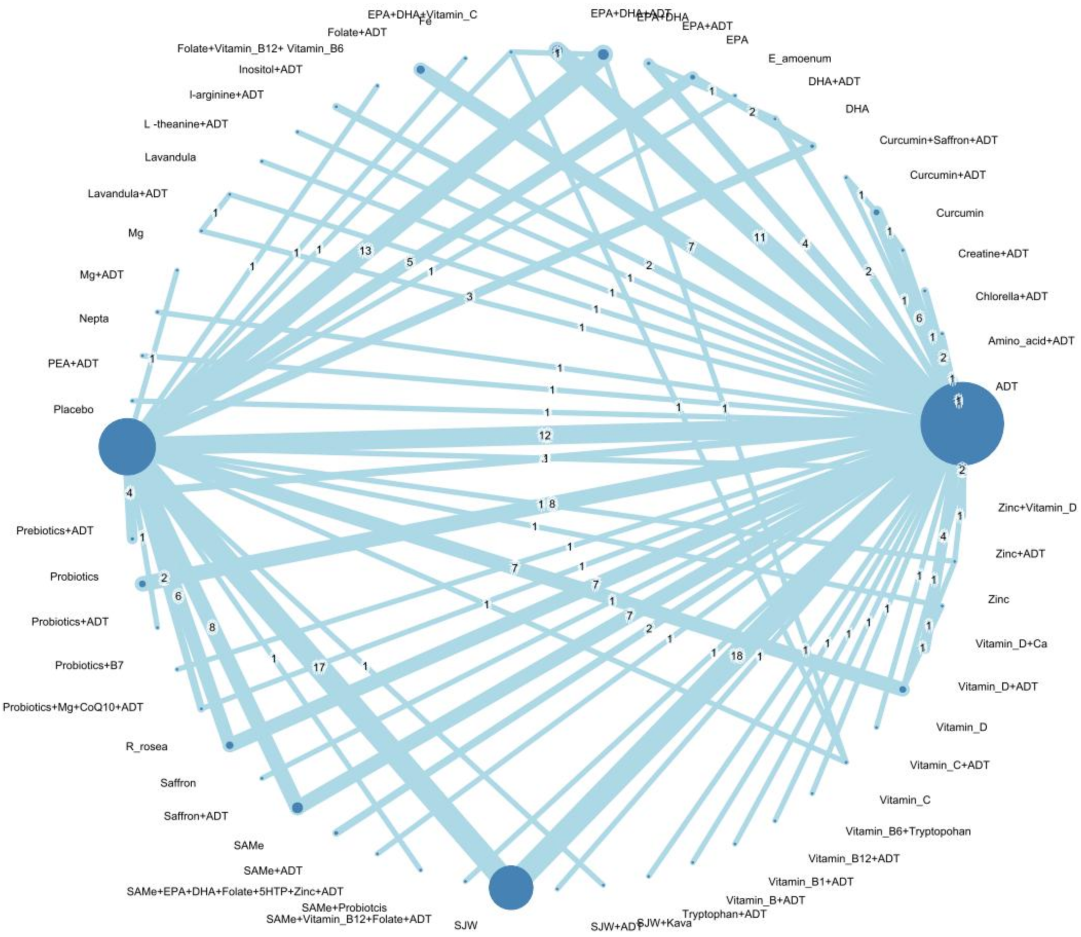

13.8-B. Forest Plot for participants aged less than 60

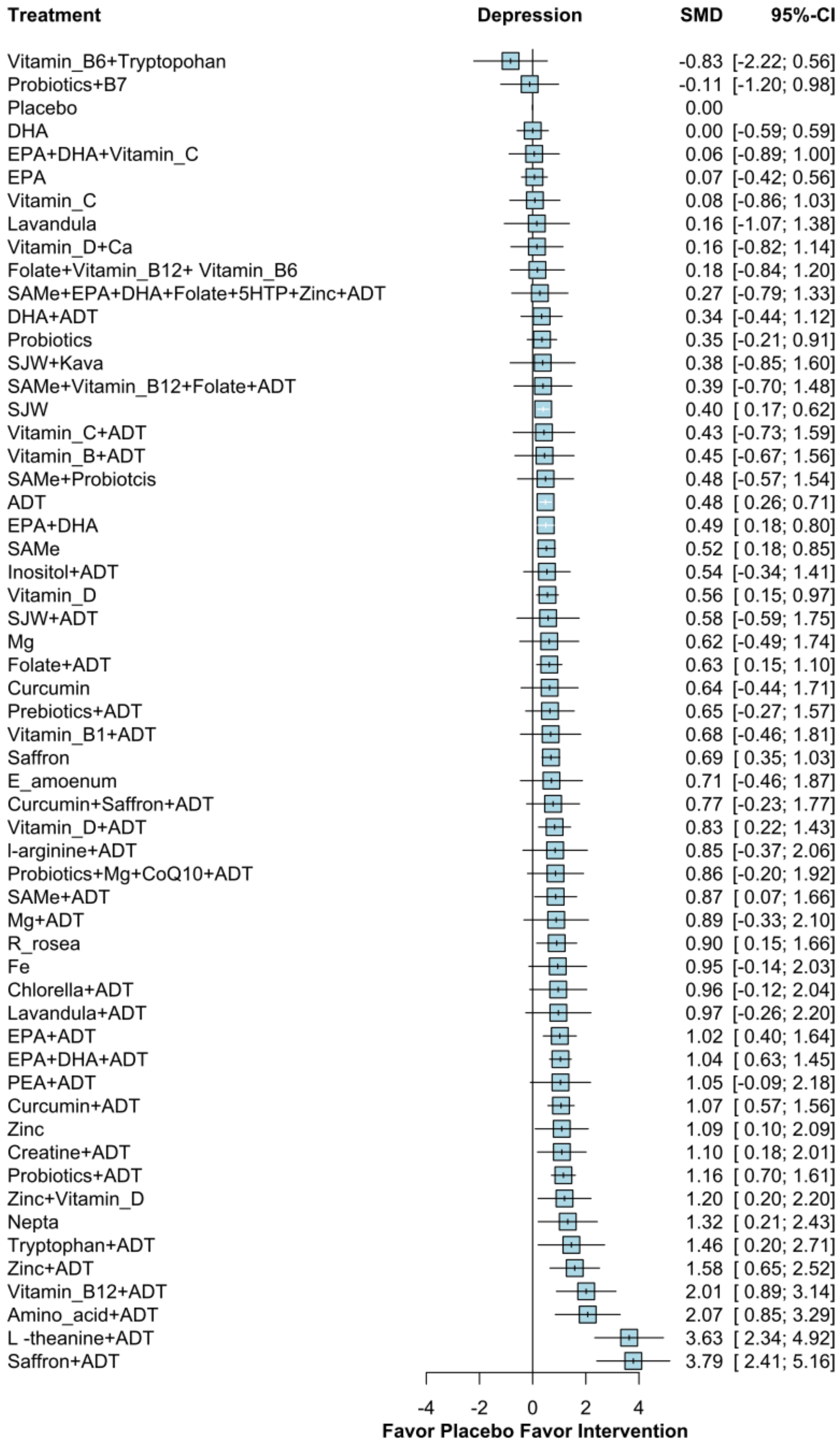

13.11-C. Detailed result for participants aged less than 60

Intervention	SMD95%-CI	p-value
ADT	0.4850 (0.2572; 0.7127)	< 0.0001
Amino_acid+ADT	2.0719 (0.8509; 3.2930)	0.0009
Chlorella+ADT	0.9611 (-0.1155; 2.0376)	0.0802
Creatine+ADT	1.0980 (0.1816; 2.0145)	0.0189
Curcumin	0.6376 (-0.4361; 1.7112)	0.2445
Curcumin+ADT	1.0673 (0.5699; 1.5648)	< 0.0001
Curcumin+Saffron+ADT	0.7690 (-0.2285; 1.7665)	0.1308
DHA	0.0023 (-0.5871; 0.5917)	0.9938
DHA+ADT	0.3379 (-0.4394; 1.1153)	0.3942
E_amoenum	0.7064 (-0.4613; 1.8741)	0.2358
EPA	0.0674 (-0.4202; 0.5551)	0.7863
EPA+ADT	1.0211 (0.3976; 1.6446)	0.0013
EPA+DHA	0.4925 (0.1831; 0.8019)	0.0018
EPA+DHA+ADT	1.0448 (0.6349; 1.4547)	< 0.0001
EPA+DHA+Vitamin_C	0.0580 (-0.8877; 1.0036)	0.9044
Fe	0.9453 (-0.1401; 2.0307)	0.0878
Folate+ADT	0.6257 (0.1488; 1.1026)	0.0101
Folate+Vitamin_B12+Vitamin_B6	0.1801 (-0.8372; 1.1973)	0.7287
Inositol+ADT	0.5374 (-0.3381; 1.4130)	0.2290
l-arginine+ADT	0.8463 (-0.3678; 2.0603)	0.1719
L-theanine+ADT	3.6302 (2.3366; 4.9237)	< 0.0001
Lavandula	0.1573 (-1.0685; 1.3831)	0.8014
Lavandula+ADT	0.9679 (-0.2601; 2.1959)	0.1224
Mg	0.6239 (-0.4889; 1.7366)	0.2718
Mg+ADT	0.8856 (-0.3300; 2.1011)	0.1533
Nepta	1.3210 (0.2079; 2.4341)	0.0200
PEA+ADT	1.0466 (-0.0855; 2.1788)	0.0700
Placebo	.	.
Prebiotics+ADT	0.6512 (-0.2651; 1.5674)	0.1636
Probiotics	0.3502 (-0.2055; 0.9059)	0.2167
Probiotics+ADT	1.1571 (0.7045; 1.6097)	< 0.0001
Probiotics+B7	-0.1128 (-1.2027; 0.9772)	0.8393
Probiotics+Mg+CoQ10+ADT	0.8599 (-0.1967; 1.9164)	0.1107
R_rosea	0.9050 (0.1492; 1.6607)	0.0189
Saffron	0.6881 (0.3503; 1.0259)	< 0.0001
Saffron+ADT	3.7866 (2.4106; 5.1626)	< 0.0001
SAMe	0.5177 (0.1833; 0.8521)	0.0024

SAMe+ADT	0.8659 (0.0737; 1.6580)	0.0322
SAMe+EPA+DHA+Folate+5HTP+Zinc+ADT	0.2716 (-0.7880; 1.3311)	0.6154
SAMe+Probiotics	0.4846 (-0.5698; 1.5390)	0.3677
SAMe+Vitamin_B12+Folate+ADT	0.3888 (-0.7014; 1.4790)	0.4845
SJW	0.3968 (0.1733; 0.6204)	0.0005
SJW+ADT	0.5808 (-0.5899; 1.7515)	0.3309
SJW+Kava	0.3768 (-0.8460; 1.5997)	0.5458
Tryptophan+ADT	1.4567 (0.2046; 2.7088)	0.0226
Vitamin_B+ADT	0.4455 (-0.6690; 1.5600)	0.4333
Vitamin_B1+ADT	0.6757 (-0.4594; 1.8109)	0.2433
Vitamin_B12+ADT	2.0140 (0.8920; 3.1360)	0.0004
Vitamin_B6+Tryptophan	-0.8306 (-2.2164; 0.5552)	0.2401
Vitamin_C	0.0839 (-0.8617; 1.0295)	0.8619
Vitamin_C+ADT	0.4265 (-0.7327; 1.5856)	0.4708
Vitamin_D	0.5575 (0.1464; 0.9685)	0.0079
Vitamin_D+ADT	0.8255 (0.2204; 1.4305)	0.0075
Vitamin_D+Ca	0.1611 (-0.8210; 1.1431)	0.7479
Zinc	1.0944 (0.0954; 2.0933)	0.0318
Zinc+ADT	1.5828 (0.6494; 2.5163)	0.0009
Zinc+Vitamin_D	1.1983 (0.2007; 2.1959)	0.0186

13.9. Sensitivity analysis for excluding perinatal depression and postpartum depression

13.9-A. Network Plot for excluding perinatal depression and postpartum depression

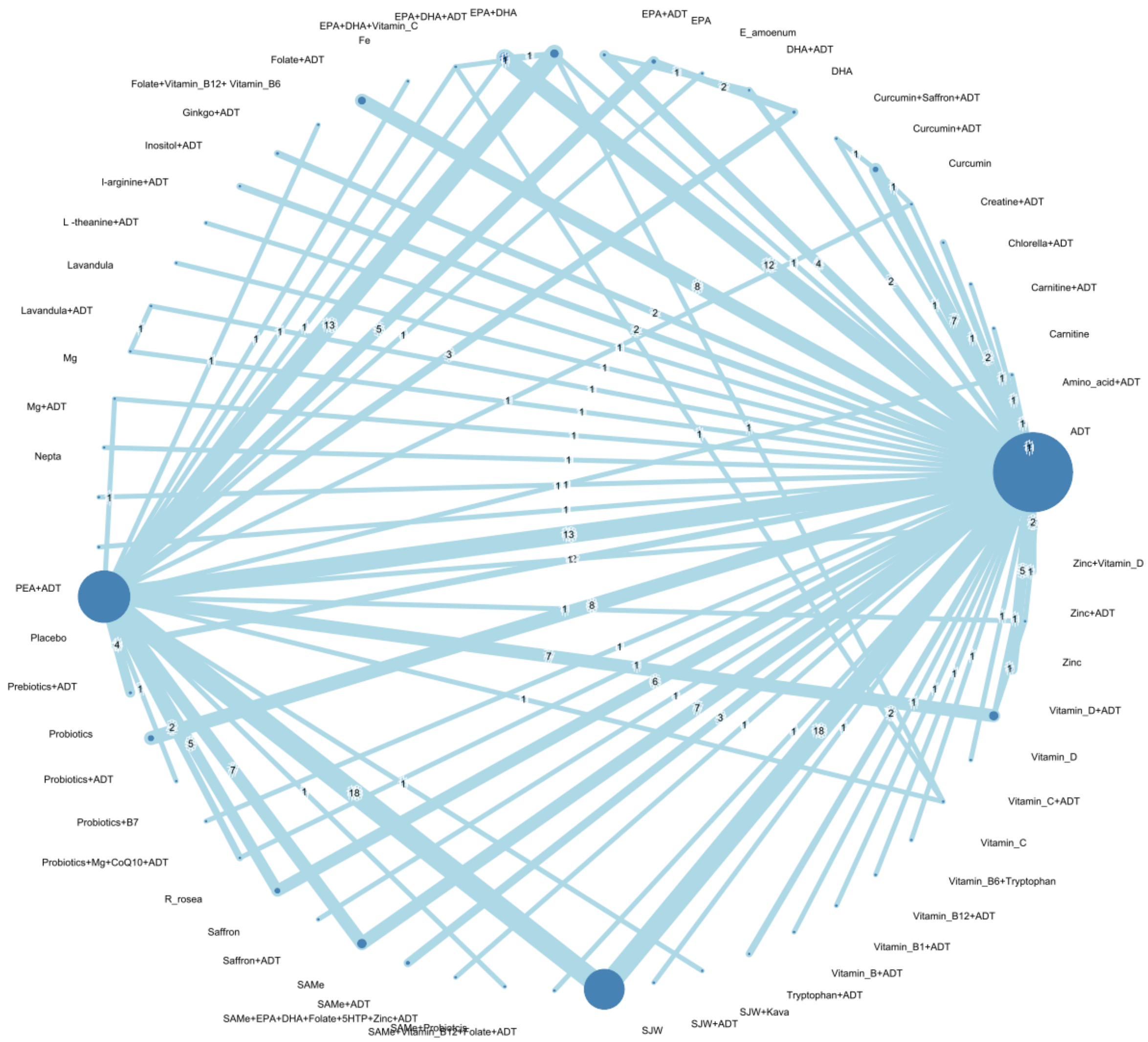

13.9-B. Forest Plot for excluding perinatal depression and postpartum depression

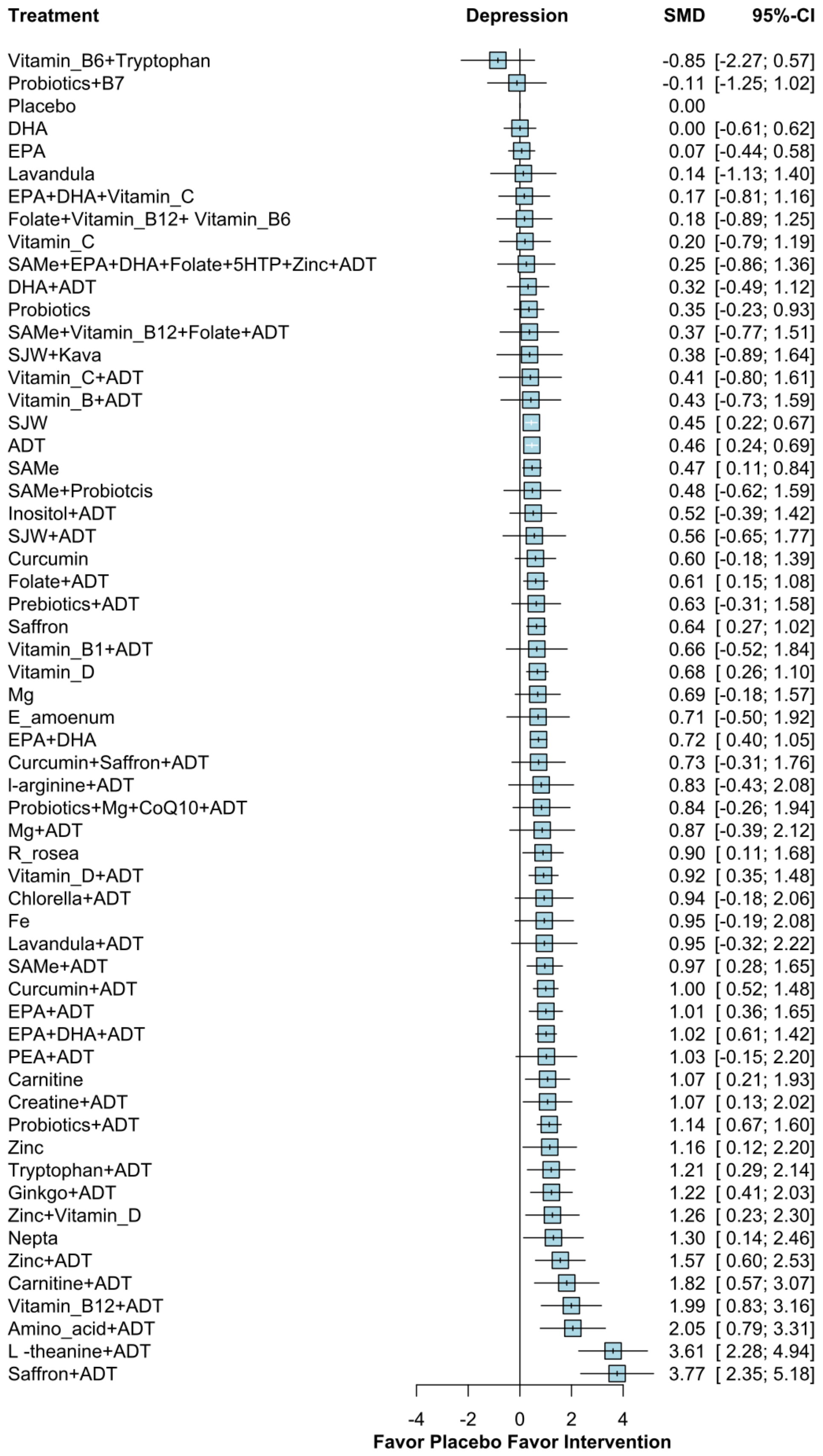

13.9-C. Detailed result for excluding perinatal depression and postpartum depression

Intervention	SMD95%-CI	p-value
ADT	0.4649 (0.2372; 0.6926)	< 0.0001
Amino_acid+ADT	2.0518 (0.7894; 3.3143)	0.0014
Carnitine	1.0727 (0.2144; 1.9311)	0.0143
Carnitine+ADT	1.8163 (0.5671; 3.0655)	0.0044
Chlorella+ADT	0.9410 (-0.1823; 2.0642)	0.1006
Creatine+ADT	1.0730 (0.1285; 2.0174)	0.026
Curcumin	0.6040 (-0.1812; 1.3892)	0.1317
Curcumin+ADT	1.0033 (0.5241; 1.4825)	< 0.0001
Curcumin+Saffron+ADT	0.7256 (-0.3069; 1.7580)	0.1684
DHA	0.0049 (-0.6103; 0.6200)	0.9876
DHA+ADT	0.3175 (-0.4891; 1.1242)	0.4404
E_amoenum	0.7064 (-0.5045; 1.9173)	0.2529
EPA	0.0708 (-0.4373; 0.5790)	0.7847
EPA+ADT	1.0077 (0.3637; 1.6516)	0.0022
EPA+DHA	0.7219 (0.3977; 1.0461)	< 0.0001
EPA+DHA+ADT	1.0163 (0.6142; 1.4185)	< 0.0001
EPA+DHA+Vitamin_C	0.1724 (-0.8144; 1.1591)	0.7321
Fe	0.9453 (-0.1865; 2.0770)	0.1016
Folate+ADT	0.6135 (0.1459; 1.0812)	0.0101
Folate+Vitamin_B12+Vitamin_B6	0.1801 (-0.8865; 1.2467)	0.7407
Ginkgo+ADT	1.2230 (0.4146; 2.0314)	0.003
Inositol+ADT	0.5176 (-0.3869; 1.4222)	0.262
l-arginine+ADT	0.8262 (-0.4295; 2.0818)	0.1972
L-theanine+ADT	3.6100 (2.2774; 4.9427)	< 0.0001
Lavandula	0.1372 (-1.1299; 1.4042)	0.8319
Lavandula+ADT	0.9478 (-0.3214; 2.2170)	0.1433
Mg	0.6925 (-0.1806; 1.5657)	0.1201
Mg+ADT	0.8655 (-0.3917; 2.1226)	0.1772
Nepta	1.3009 (0.1425; 2.4593)	0.0277
PEA+ADT	1.0265 (-0.1501; 2.2032)	0.0873
Placebo	.	.
Prebiotics+ADT	0.6347 (-0.3095; 1.5789)	0.1877
Probiotics	0.3528 (-0.2257; 0.9313)	0.232
Probiotics+ADT	1.1379 (0.6712; 1.6046)	< 0.0001
Probiotics+B7	-0.1128 (-1.2489; 1.0233)	0.8457
Probiotics+Mg+CoQ10+ADT	0.8398 (-0.2644; 1.9439)	0.136
R_rosea	0.8988 (0.1145; 1.6832)	0.0247

Saffron	0.6435 (0.2653; 1.0217)	0.0009
Saffron+ADT	3.7665 (2.3536; 5.1794)	< 0.0001
SAMe	0.4740 (0.1110; 0.8371)	0.0105
SAMe+ADT	0.9660 (0.2795; 1.6525)	0.0058
SAMe+EPA+DHA+Folate+5HTP+Zinc+ADT	0.2515 (-0.8555; 1.3585)	0.6561
SAMe+Probiotics	0.4846 (-0.6174; 1.5867)	0.3887
SAMe+Vitamin_B12+Folate+ADT	0.3687 (-0.7676; 1.5050)	0.5248
SJW	0.4453 (0.2182; 0.6724)	0.0001
SJW+ADT	0.5607 (-0.6531; 1.7745)	0.3653
SJW+Kava	0.3768 (-0.8873; 1.6410)	0.559
Tryptophan+ADT	1.2133 (0.2898; 2.1368)	0.01
Vitamin_B+ADT	0.4254 (-0.7342; 1.5851)	0.4721
Vitamin_B1+ADT	0.6556 (-0.5239; 1.8352)	0.276
Vitamin_B12+ADT	1.9939 (0.8270; 3.1608)	0.0008
Vitamin_B6+Tryptophan	-0.8507 (-2.2731; 0.5717)	0.2411
Vitamin_C	0.1983 (-0.7884; 1.1851)	0.6936
Vitamin_C+ADT	0.4064 (-0.7963; 1.6090)	0.5078
Vitamin_D	0.6801 (0.2585; 1.1018)	0.0016
Vitamin_D+ADT	0.9184 (0.3534; 1.4835)	0.0014
Zinc	1.1579 (0.1200; 2.1958)	0.0288
Zinc+ADT	1.5671 (0.6042; 2.5299)	0.0014

13.10. Sensitivity analysis for study duration between 4-12 weeks

13.10-A. Network Plot for subgroup for study duration between 4-12weeks

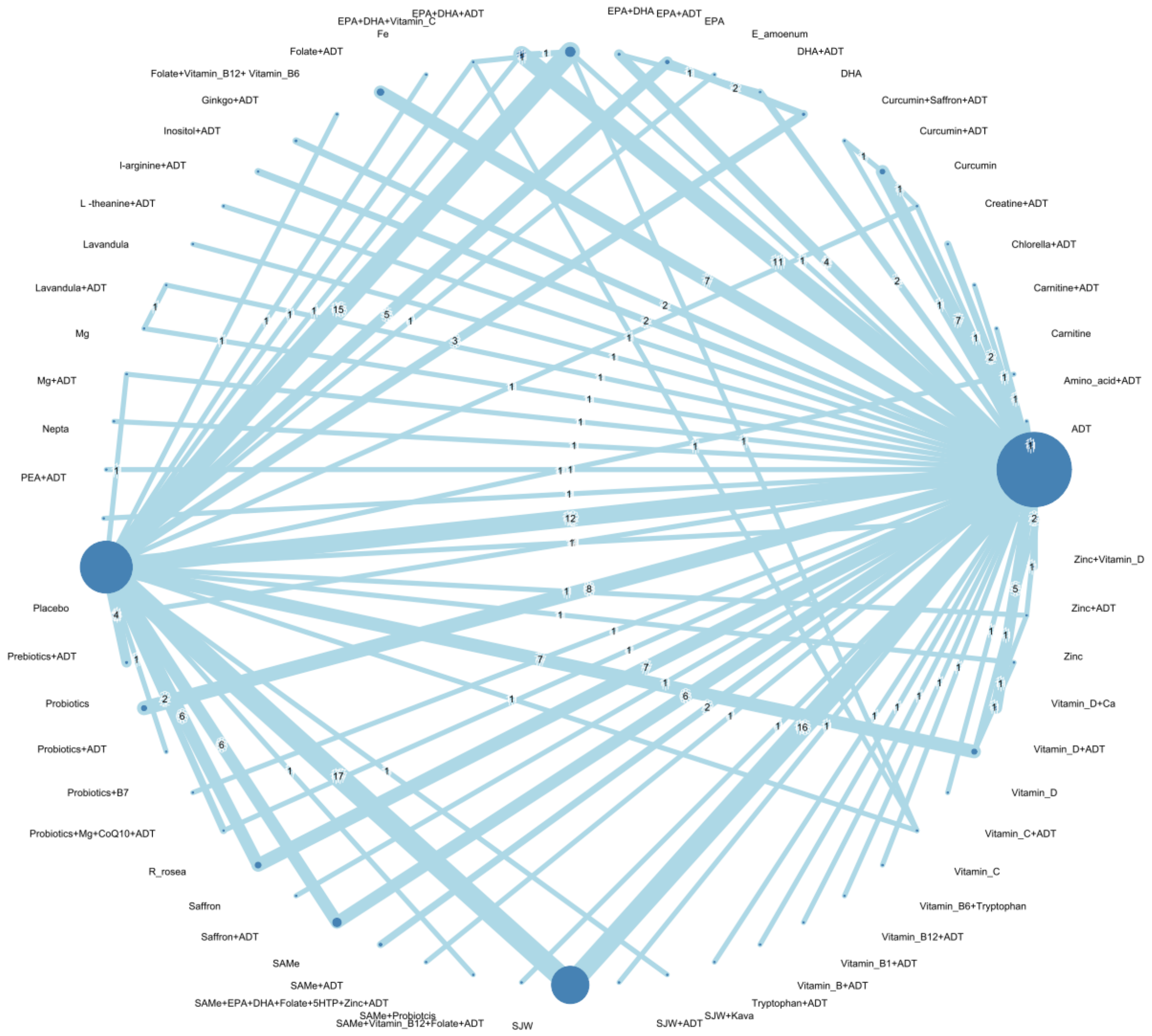

13.10-B. Forest Plot for subgroup for study duration between 4-12 weeks

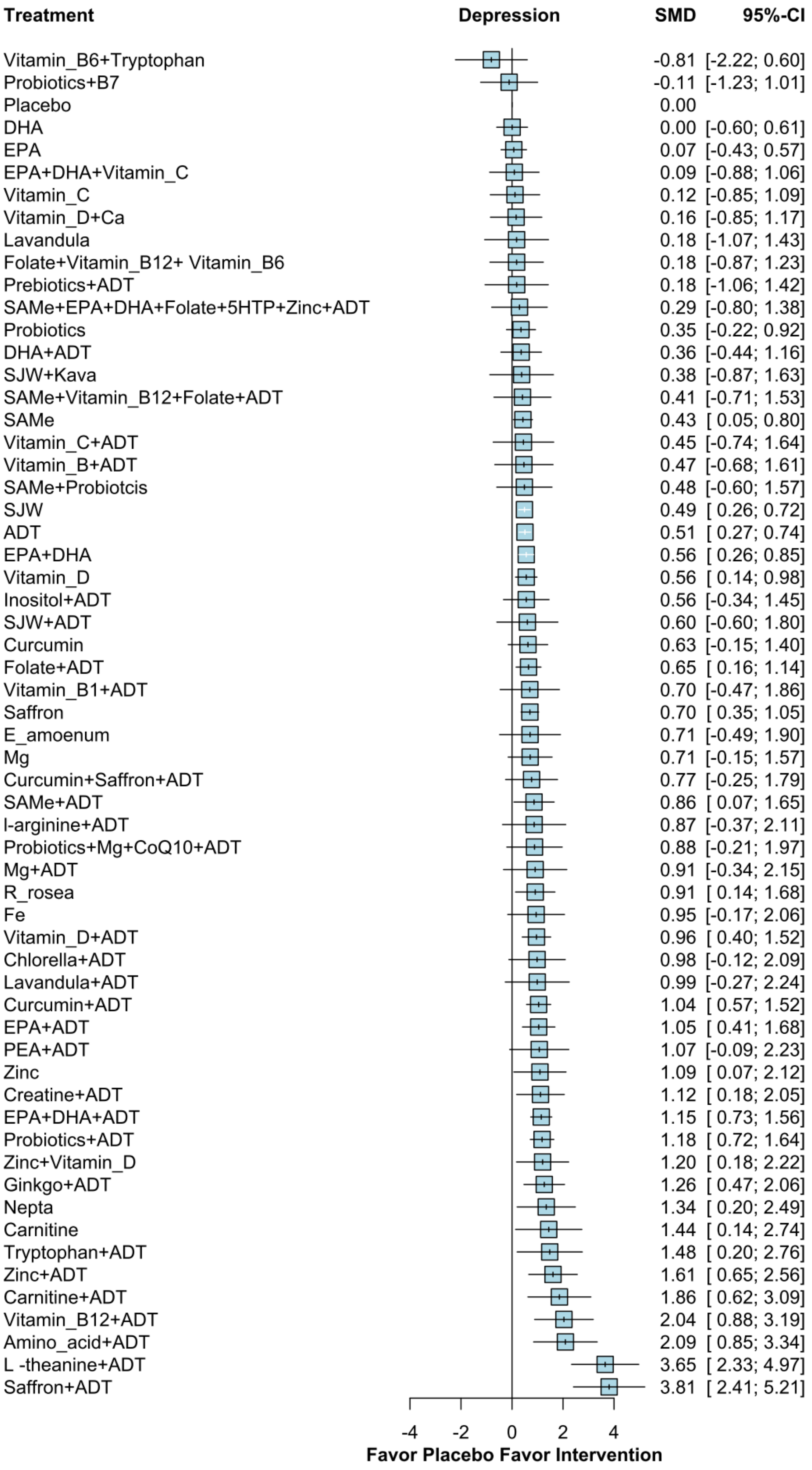

13.10-C. Detailed result for subgroup for study duration between 4-12 weeks

Intervention	SMD95%-CI	p-value
ADT	0.5063 (0.2747; 0.7379)	< 0.0001
Amino_acid+ADT	2.0933 (0.8451; 3.3414)	0.001
Carnitine	1.4393 (0.1393; 2.7393)	0.03
Carnitine+ADT	1.8577 (0.6230; 3.0924)	0.0032
Chlorella+ADT	0.9824 (-0.1248; 2.0895)	0.082
Creatine+ADT	1.1161 (0.1809; 2.0513)	0.0193
Curcumin	0.6253 (-0.1494; 1.4000)	0.1136
Curcumin+ADT	1.0435 (0.5682; 1.5189)	< 0.0001
Curcumin+Saffron+ADT	0.7665 (-0.2523; 1.7854)	0.1403
DHA	0.0040 (-0.6018; 0.6098)	0.9897
DHA+ADT	0.3591 (-0.4381; 1.1562)	0.3773
E_amoenum	0.7064 (-0.4889; 1.9017)	0.2467
EPA	0.0696 (-0.4311; 0.5704)	0.7851
EPA+ADT	1.0468 (0.4088; 1.6847)	0.0013
EPA+DHA	0.5577 (0.2639; 0.8515)	0.0002
EPA+DHA+ADT	1.1474 (0.7335; 1.5612)	< 0.0001
EPA+DHA+Vitamin_C	0.0905 (-0.8794; 1.0604)	0.8549
Fe	0.9453 (-0.1697; 2.0602)	0.0966
Folate+ADT	0.6477 (0.1591; 1.1364)	0.0094
Folate+Vitamin_B12+Vitamin_B6	0.1801 (-0.8687; 1.2288)	0.7365
Ginkgo+ADT	1.2639 (0.4661; 2.0617)	0.0019
Inositol+ADT	0.5590 (-0.3360; 1.4540)	0.2209
l-arginine+ADT	0.8676 (-0.3737; 2.1089)	0.1707
L-theanine+ADT	3.6515 (2.3324; 4.9706)	< 0.0001
Lavandula	0.1786 (-1.0742; 1.4314)	0.7799
Lavandula+ADT	0.9892 (-0.2657; 2.2442)	0.1224
Mg	0.7104 (-0.1519; 1.5727)	0.1064
Mg+ADT	0.9069 (-0.3359; 2.1496)	0.1526
Nepta	1.3423 (0.1996; 2.4851)	0.0213
PEA+ADT	1.0680 (-0.0933; 2.2292)	0.0715
Placebo	.	.
Prebiotics+ADT	0.1825 (-1.0582; 1.4231)	0.7732
Probiotics	0.3519 (-0.2183; 0.9221)	0.2265
Probiotics+ADT	1.1790 (0.7155; 1.6425)	< 0.0001
Probiotics+B7	-0.1128 (-1.2322; 1.0066)	0.8435
Probiotics+Mg+CoQ10+ADT	0.8812 (-0.2066; 1.9689)	0.1123
R_rosea	0.9108 (0.1367; 1.6849)	0.0211

Saffron	0.7001 (0.3542; 1.0460)	< 0.0001
Saffron+ADT	3.8079 (2.4078; 5.2080)	< 0.0001
SAMe	0.4270 (0.0538; 0.8001)	0.0249
SAMe+ADT	0.8632 (0.0734; 1.6530)	0.0322
SAMe+EPA+DHA+Folate+5HTP+Zinc+ADT	0.2929 (-0.7977; 1.3835)	0.5986
SAMe+Probiotics	0.4846 (-0.6002; 1.5694)	0.3813
SAMe+Vitamin_B12+Folate+ADT	0.4101 (-0.7103; 1.5305)	0.4731
SJW	0.4878 (0.2580; 0.7177)	< 0.0001
SJW+ADT	0.6021 (-0.5968; 1.8010)	0.3249
SJW+Kava	0.3768 (-0.8723; 1.6260)	0.5543
Tryptophan+ADT	1.4780 (0.1995; 2.7565)	0.0235
Vitamin_B+ADT	0.4668 (-0.6772; 1.6109)	0.4239
Vitamin_B1+ADT	0.6971 (-0.4672; 1.8613)	0.2406
Vitamin_B12+ADT	2.0353 (0.8839; 3.1867)	0.0005
Zinc	1.0948 (0.0705; 2.1191)	0.0362
Zinc+ADT	1.6070 (0.6538; 2.5601)	0.001
Zinc+Vitamin_D	1.1988 (0.1758; 2.2217)	0.0216

13.11. Sensitivity analysis for excluding studies using EPA less than 1g

13.11-A. Network Plot for excluding studies using EPA less than 1g

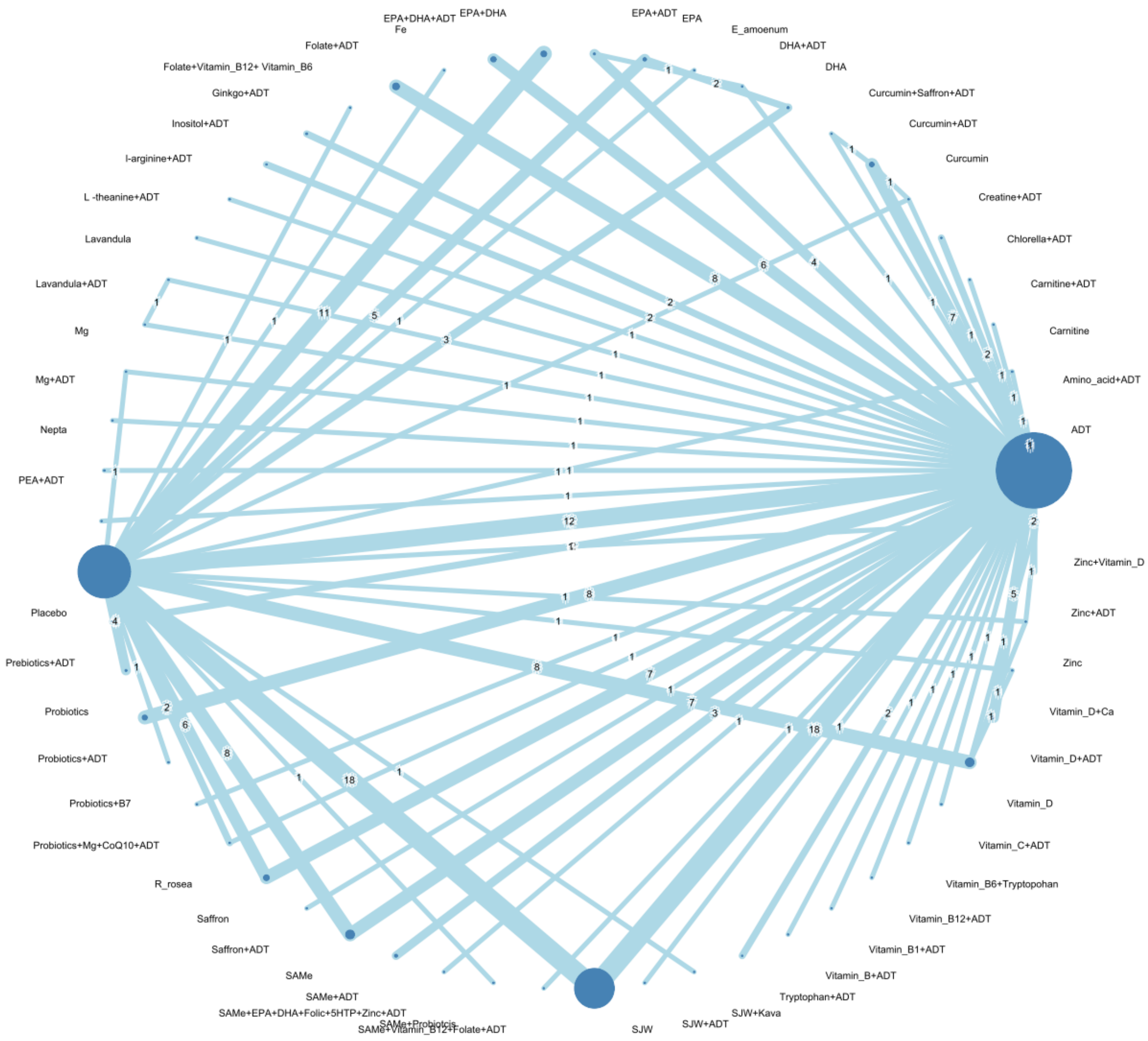

13.14-B. Forest Plot for excluding studies using EPA less than 1g

Treatment	SMD	95%-CI
Vitamin_B6+Tryptopohan	-0.78	[-2.16; 0.59]
Probiotics+B7	-0.11	[-1.19; 0.97]
Placebo	0.00	
DHA	0.00	[-0.58; 0.58]
EPA	0.07	[-0.42; 0.55]
DHA+ADT	0.15	[-0.90; 1.21]
Folate+Vitamin_B12+ Vitamin_B6	0.18	[-0.83; 1.19]
Lavandula	0.20	[-1.01; 1.42]
Vitamin_D+Ca	0.24	[-0.73; 1.21]
SAMe+EPA+DHA+Folic+5HTP+Zinc+ADT	0.32	[-0.73; 1.36]
Probiotics	0.35	[-0.20; 0.90]
SJW+Kava	0.38	[-0.84; 1.59]
SAMe+Vitamin_B12+Folate+ADT	0.44	[-0.64; 1.51]
EPA+DHA	0.46	[0.12; 0.80]
Vitamin_C+ADT	0.47	[-0.67; 1.62]
SJW	0.48	[0.26; 0.69]
SAMe+Probiotcis	0.48	[-0.56; 1.53]
Vitamin_B+ADT	0.49	[-0.61; 1.59]
ADT	0.53	[0.31; 0.75]
SAMe	0.54	[0.21; 0.87]
Inositol+ADT	0.58	[-0.28; 1.45]
SJW+ADT	0.63	[-0.53; 1.79]
Curcumin	0.64	[-0.11; 1.39]
Folate+ADT	0.68	[0.23; 1.12]
Prebiotics+ADT	0.70	[-0.21; 1.60]
E_amoenum	0.71	[-0.45; 1.86]
Vitamin_D	0.71	[0.33; 1.08]
Saffron	0.71	[0.38; 1.05]
Mg	0.72	[-0.11; 1.56]
Vitamin_B1+ADT	0.72	[-0.40; 1.85]
Curcumin+Saffron+ADT	0.79	[-0.19; 1.77]
EPA+DHA+ADT	0.82	[0.30; 1.33]
l-arginine+ADT	0.89	[-0.31; 2.10]
Probiotics+Mg+CoQ10+ADT	0.91	[-0.14; 1.95]
R_rosea	0.92	[0.17; 1.67]
Mg+ADT	0.93	[-0.27; 2.14]
Fe	0.95	[-0.13; 2.02]
Vitamin_D+ADT	0.98	[0.45; 1.52]
Chlorella+ADT	1.01	[-0.06; 2.07]
Lavandula+ADT	1.02	[-0.20; 2.23]
SAMe+ADT	1.03	[0.38; 1.68]
EPA+ADT	1.03	[0.41; 1.66]
Curcumin+ADT	1.07	[0.61; 1.53]
PEA+ADT	1.09	[-0.03; 2.21]
Carnitine	1.11	[0.29; 1.93]
Creatine+ADT	1.15	[0.24; 2.05]
Zinc	1.17	[0.19; 2.16]
Probiotics+ADT	1.20	[0.76; 1.65]
Zinc+Vitamin_D	1.28	[0.29; 2.26]
Tryptophan+ADT	1.28	[0.39; 2.17]
Ginkgo+ADT	1.29	[0.52; 2.05]
Nepta	1.37	[0.27; 2.47]
Zinc+ADT	1.63	[0.70; 2.55]
Carnitine+ADT	1.88	[0.69; 3.08]
Vitamin_B12+ADT	2.06	[0.95; 3.17]
Amino_acid+ADT	2.12	[0.91; 3.33]
L -theanine+ADT	3.68	[2.39; 4.96]
Saffron+ADT	3.83	[2.47; 5.20]

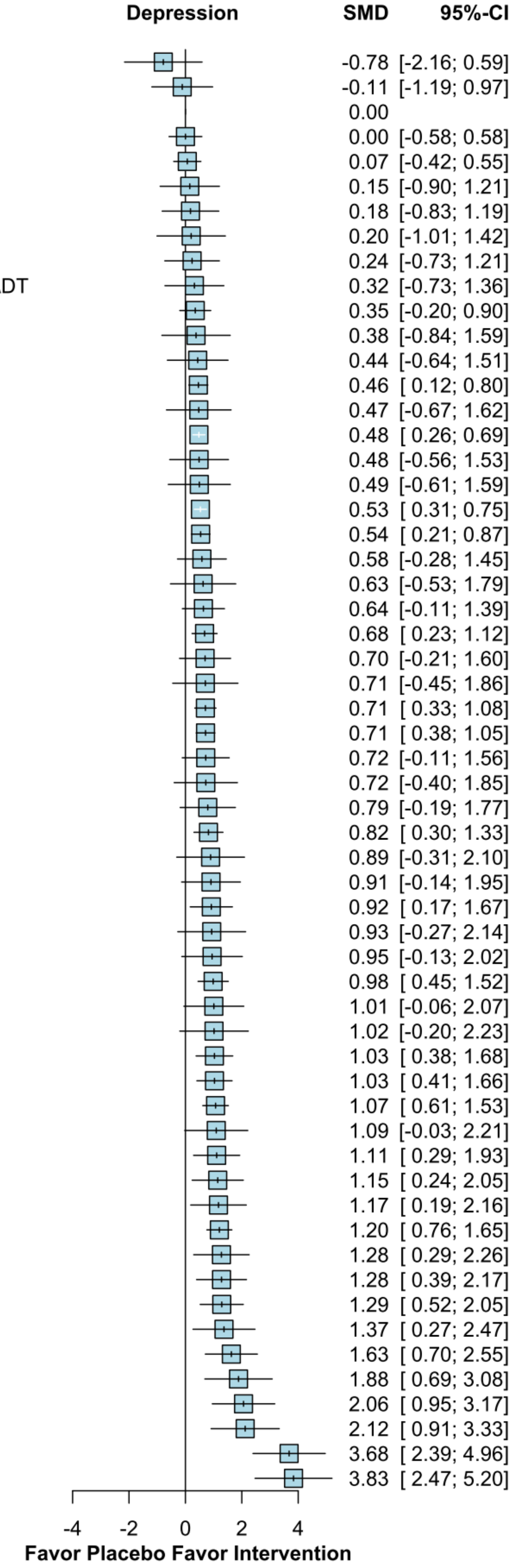

13.14-C. Detailed result excluding studies using EPA less than 1g

	Primary outcome	Excluding study with EPA <1g
	SMD (95%CI)	SMD (95%CI)
ADT	0.4910 (0.2672; 0.7148)	0.5322 (0.3143; 0.7500)
Amino_acid+ADT	2.0780 (0.8187; 3.3372)	2.1191 (0.9096; 3.3286)
Carnitine	1.0878 (0.2316; 1.9439)	1.1076 (0.2887; 1.9265)
Carnitine+ADT	1.8424 (0.5964; 3.0884)	1.8836 (0.6879; 3.0792)
Chlorella+ADT	0.9671 (-0.1526; 2.0868)	1.0082 (-0.0552; 2.0716)
Creatine+ADT	1.0994 (0.1575; 2.0412)	1.1464 (0.2390; 2.0538)
Curcumin	0.6174 (-0.1657; 1.4005)	0.6387 (-0.1089; 1.3863)
Curcumin+ADT	1.0285 (0.5519; 1.5050)	1.0695 (0.6137; 1.5253)
Curcumin+Saffron+ADT	0.7512 (-0.2780; 1.7805)	0.7929 (-0.1881; 1.7739)
DHA	0.0047 (-0.6089; 0.6184)	0.0017 (-0.5816; 0.5850)
DHA+ADT	0.3437 (-0.4601; 1.1475)	0.1546 (-0.8966; 1.2059)
E_amoenum	0.7064 (-0.5020; 1.9148)	0.7064 (-0.4512; 1.8640)
EPA	0.0706 (-0.4363; 0.5776)	0.0666 (-0.4162; 0.5494)
EPA+ADT	1.0334 (0.3920; 1.6748)	1.0307 (0.4055; 1.6559)
EPA+DHA	0.5994 (0.3165; 0.8823)	0.4599 (0.1215; 0.7983)
EPA+DHA+ADT	1.0373 (0.6378; 1.4367)	0.8175 (0.3018; 1.3332)
EPA+DHA+Vitamin_C	0.1113 (-0.8700; 1.0925)	
Fe	0.9453 (-0.1837; 2.0743)	0.9453 (-0.1291; 2.0197)
Folate+ADT	0.6396 (0.1747; 1.1045)	0.6796 (0.2346; 1.1246)
Folate+Vitamin_B12+Vitamin_B6	0.1801 (-0.8836; 1.2437)	0.1801 (-0.8255; 1.1856)
Ginkgo+ADT	1.2490 (0.4436; 2.0544)	1.2884 (0.5231; 2.0538)
Inositol+ADT	0.5437 (-0.3581; 1.4456)	0.5845 (-0.2817; 1.4508)
l-arginine+ADT	0.8523 (-0.4002; 2.1048)	0.8935 (-0.3090; 2.0959)
L-theanine+ADT	3.6362 (2.3065; 4.9658)	3.6773 (2.3947; 4.9599)
Lavandula	0.1633 (-1.1006; 1.4272)	0.2045 (-1.0099; 1.4188)
Lavandula+ADT	0.9739 (-0.2921; 2.2399)	1.0151 (-0.2015; 2.2316)
Mg	0.7039 (-0.1672; 1.5751)	0.7208 (-0.1141; 1.5557)
Mg+ADT	0.8916 (-0.3624; 2.1455)	0.9327 (-0.2712; 2.1367)
Nepta	1.3270 (0.1721; 2.4819)	1.3682 (0.2677; 2.4686)
PEA+ADT	1.0527 (-0.1206; 2.2259)	1.0938 (-0.0258; 2.2134)
Placebo	.	.
Prebiotics+ADT	0.6606 (-0.2810; 1.6022)	0.6974 (-0.2098; 1.6047)
Probiotics	0.3526 (-0.2245; 0.9298)	0.3496 (-0.2007; 0.8998)
Probiotics+ADT	1.1640 (0.7000; 1.6280)	1.2040 (0.7597; 1.6484)
Probiotics+B7	-0.1128 (-1.2462; 1.0206)	-0.1128 (-1.1918; 0.9663)

Probiotics+Mg+CoQ10+ADT	0.8659 (-0.2346; 1.9664)	0.9070 (-0.1362; 1.9502)
R_rosea	0.9063 (0.1237; 1.6889)	0.9186 (0.1698; 1.6673)
Saffron	0.6920 (0.3441; 1.0399)	0.7135 (0.3804; 1.0466)
Saffron+ADT	3.7926 (2.3826; 5.2027)	3.8338 (2.4680; 5.1996)
SAMe	0.5239 (0.1789; 0.8689)	0.5398 (0.2099; 0.8698)
SAMe+ADT	0.9919 (0.3082; 1.6756)	1.0289 (0.3776; 1.6801)
SAMe+EPA+DHA+Folate+5HTP+Zinc+ADT	0.2776 (-0.8258; 1.3810)	0.3187 (-0.7275; 1.3650)
SAMe+Probiotics	0.4846 (-0.6146; 1.5839)	0.4846 (-0.5585; 1.5277)
SAMe+Vitamin_B12+Folate+ADT	0.3948 (-0.7380; 1.5276)	0.4360 (-0.6412; 1.5132)
SJW	0.4583 (0.2327; 0.6840)	0.4786 (0.2634; 0.6938)
SJW+ADT	0.5868 (-0.6237; 1.7973)	0.6280 (-0.5306; 1.7866)
SJW+Kava	0.3768 (-0.8849; 1.6386)	0.3768 (-0.8363; 1.5900)
Tryptophan+ADT	1.2394 (0.3185; 2.1603)	1.2804 (0.3942; 2.1665)
Zinc	1.1722 (0.1393; 2.2051)	1.1730 (0.1868; 2.1593)
Zinc+ADT	1.5929 (0.6328; 2.5531)	1.6289 (0.7048; 2.5530)
Zinc+Vitamin_D	1.2763 (0.2448; 2.3078)	1.2772 (0.2923; 2.2620)

13.12. Sensitivity analysis for combining EPA and DHA to omega-3 polyunsaturated fatty acids group

13.12-A. Forest Plot for combining EPA and DHA to omega-3 polyunsaturated fatty acids group

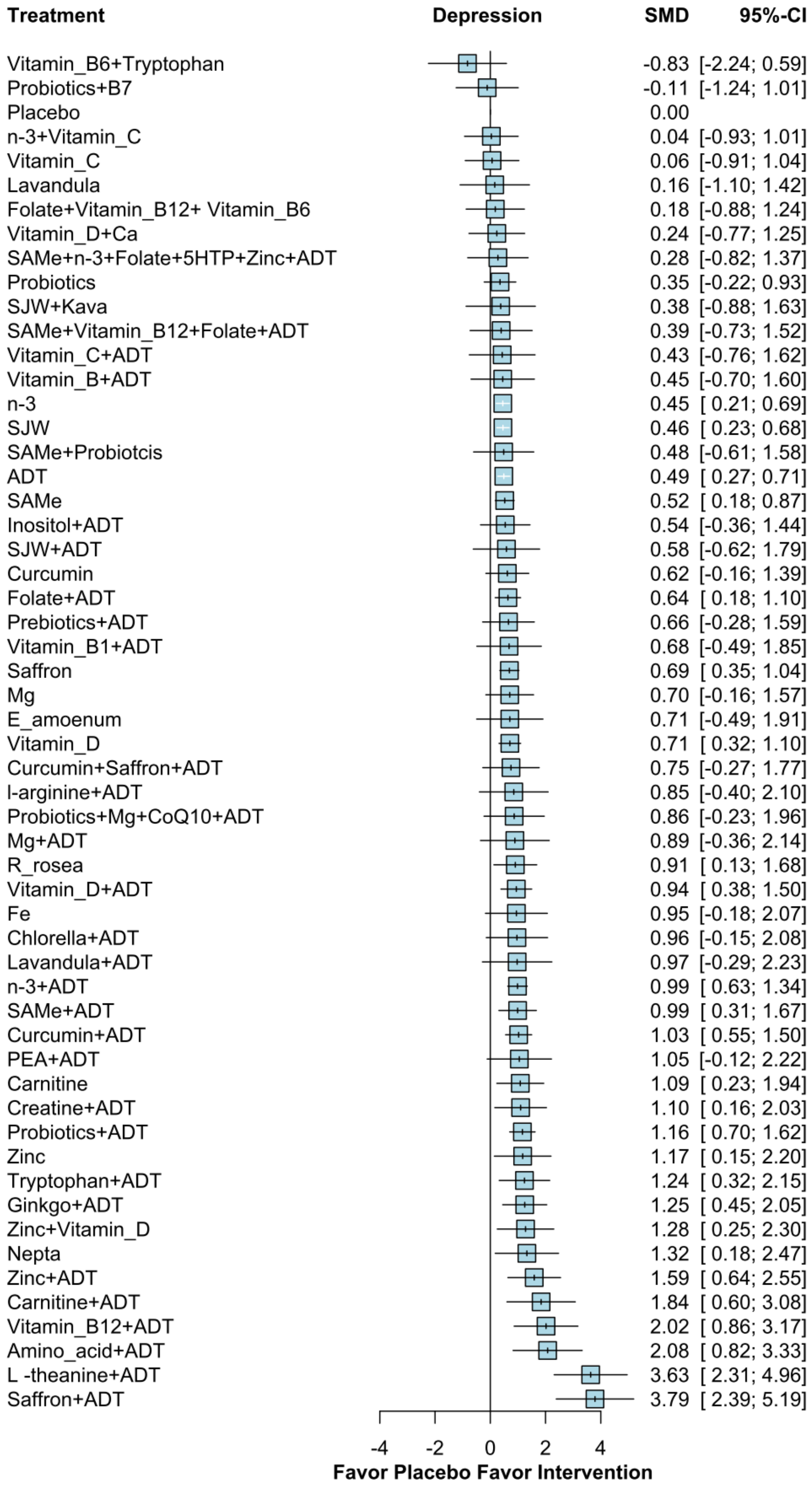

Abbreviation: n-3: Omega-3 polyunsaturated fatty acids

13.12-B. Detailed result for combining EPA and DHA to n3 group

Intervention	SMD95%-CI	p-value
ADT	0.4889 (0.2666; 0.7112)	< 0.0001
Amino_acid+ADT	2.0758 (0.8236; 3.3281)	0.0012
Carnitine	1.0859 (0.2350; 1.9369)	0.0124
Carnitine+ADT	1.8403 (0.6014; 3.0792)	0.0036
Chlorella+ADT	0.9650 (-0.1468; 2.0768)	0.0889
Creatine+ADT	1.0980 (0.1611; 2.0349)	0.0216
Curcumin	0.6164 (-0.1618; 1.3945)	0.1206
Curcumin+ADT	1.0266 (0.5532; 1.5000)	< 0.0001
Curcumin+Saffron+ADT	0.7493 (-0.2731; 1.7718)	0.1509
E_amoenum	0.7064 (-0.4950; 1.9077)	0.2491
Fe	0.9453 (-0.1762; 2.0667)	0.0985
Folate+ADT	0.6373 (0.1755; 1.0992)	0.0068
Folate+Vitamin_B12+Vitamin_B6	0.1801 (-0.8756; 1.2358)	0.7382
Ginkgo+ADT	1.2467 (0.4470; 2.0464)	0.0022
Inositol+ADT	0.5416 (-0.3551; 1.4383)	0.2365
l-arginine+ADT	0.8502 (-0.3952; 2.0956)	0.1809
L-theanine+ADT	3.6341 (2.3110; 4.9571)	< 0.0001
Lavandula	0.1612 (-1.0957; 1.4181)	0.8015
Lavandula+ADT	0.9718 (-0.2872; 2.2309)	0.1303
Mg	0.7029 (-0.1632; 1.5690)	0.1117
Mg+ADT	0.8895 (-0.3574; 2.1364)	0.1621
n-3	0.4522 (0.2111; 0.6933)	0.0002
n-3+ADT	0.9856 (0.6303; 1.3409)	< 0.0001
n-3+Vitamin_C	0.0379 (-0.9341; 1.0098)	0.9391
Nepta	1.3249 (0.1777; 2.4722)	0.0236
PEA+ADT	1.0506 (-0.1151; 2.2162)	0.0773
Placebo	.	.
Prebiotics+ADT	0.6579 (-0.2787; 1.5946)	0.1686
Probiotics	0.3522 (-0.2212; 0.9257)	0.2286
Probiotics+ADT	1.1617 (0.7008; 1.6226)	< 0.0001
Probiotics+B7	-0.1128 (-1.2387; 1.0131)	0.8443
Probiotics+Mg+CoQ10+ADT	0.8638 (-0.2287; 1.9562)	0.1212
R_rosea	0.9058 (0.1279; 1.6837)	0.0225
Saffron	0.6908 (0.3451; 1.0365)	< 0.0001
Saffron+ADT	3.7905 (2.3867; 5.1943)	< 0.0001
SAMe	0.5223 (0.1795; 0.8652)	0.0028
SAMe+ADT	0.9893 (0.3103; 1.6682)	0.0043

SAMe+n-3+Folate+5HTP+Zinc+ADT	0.2755 (-0.8199; 1.3708)	0.6221
SAMe+Probiotics	0.4846 (-0.6069; 1.5761)	0.3842
SAMe+Vitamin_B12+Folate+ADT	0.3927 (-0.7323; 1.5177)	0.4939
SJW	0.4573 (0.2332; 0.6813)	< 0.0001
SJW+ADT	0.5847 (-0.6185; 1.7879)	0.3409
SJW+Kava	0.3768 (-0.8781; 1.6318)	0.5562
Tryptophan+ADT	1.2373 (0.3214; 2.1532)	0.0081
Zinc	1.1723 (0.1459; 2.1987)	0.0252
Zinc+ADT	1.5902 (0.6351; 2.5452)	0.0011
Zinc+Vitamin_D	1.2764 (0.2513; 2.3015)	0.0147
4o		

Abbreviation: n-3: Omega-3 polyunsaturated fatty acids

13.13. Sensitivity analysis for combining SAMe, folate, vitamin B12 and vitamin B6 to one Carbon cycle group

13.13-A. Forest Plot for combining SAMe, folate, vitamin B12 and vitamin B6 to one Carbon cycle group

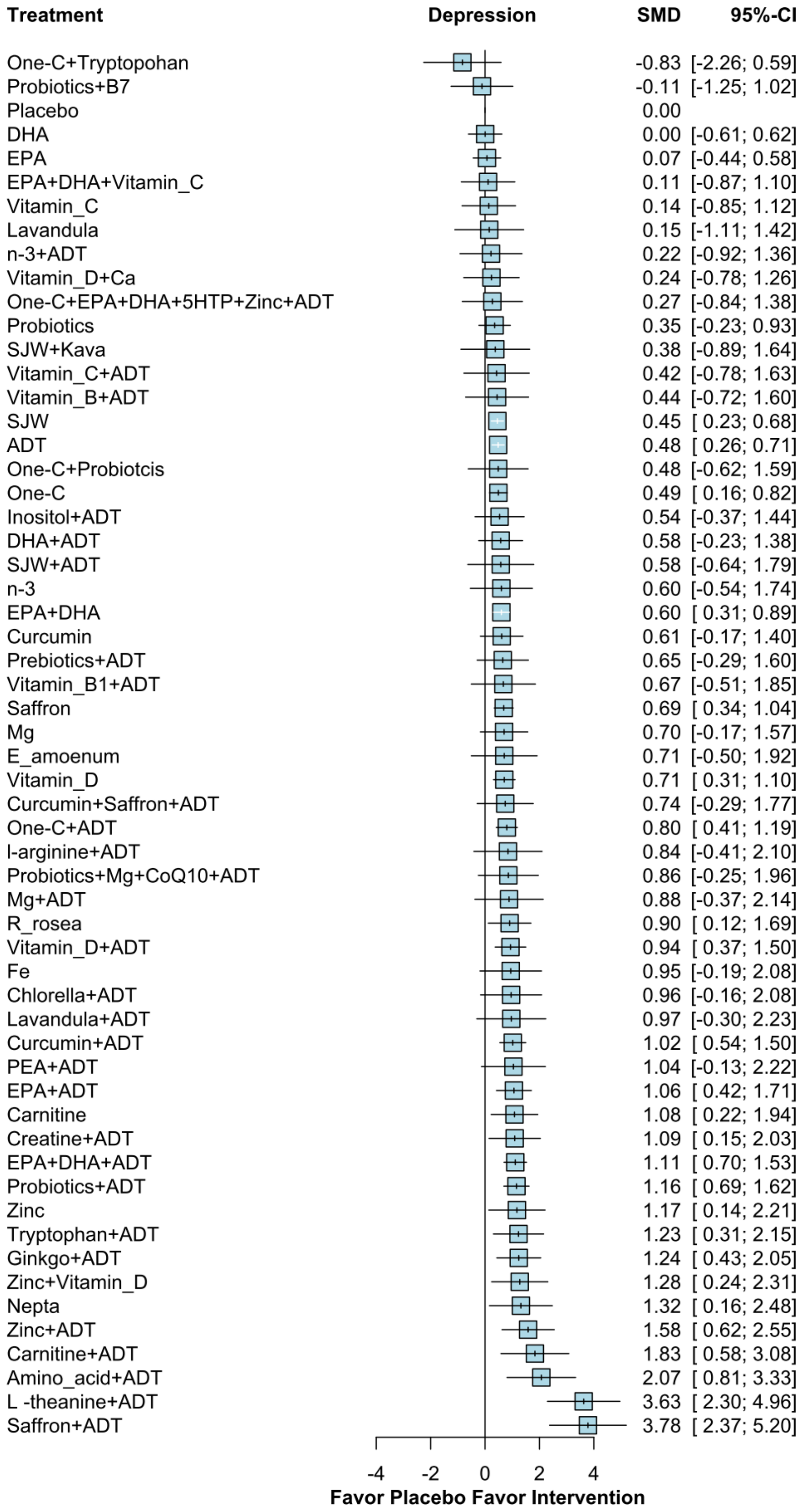

Abbreviation: One-C: one Carbon cycle group

13.13-B. Detailed result for combining SAMe, folate, vitamin B12 and vitamin B6 to one Carbon cycle group

Intervention	SMD95%-CI	p-value
ADT	0.4824 (0.2590; 0.7059)	< 0.0001
Amino_acid+ADT	2.0694 (0.8073; 3.3315)	0.0013
Carnitine	1.0830 (0.2247; 1.9413)	0.0134
Carnitine+ADT	1.8338 (0.5850; 3.0827)	0.004
Chlorella+ADT	0.9585 (-0.1643; 2.0814)	0.0943
Creatine+ADT	1.0905 (0.1468; 2.0341)	0.0235
Curcumin	0.6130 (-0.1722; 1.3981)	0.126
Curcumin+ADT	1.0201 (0.5426; 1.4976)	< 0.0001
Curcumin+Saffron+ADT	0.7428 (-0.2892; 1.7747)	0.1583
DHA	0.0049 (-0.6105; 0.6203)	0.9875
DHA+ADT	0.5752 (-0.2318; 1.3821)	0.1624
E_amoenum	0.7064 (-0.5050; 1.9178)	0.2531
EPA	0.0709 (-0.4375; 0.5792)	0.7847
EPA+ADT	1.0628 (0.4201; 1.7055)	0.0012
EPA+DHA	0.6009 (0.3081; 0.8938)	< 0.0001
EPA+DHA+ADT	1.1118 (0.6980; 1.5255)	< 0.0001
EPA+DHA+Vitamin_C	0.1120 (-0.8727; 1.0968)	0.8235
Fe	0.9453 (-0.1869; 2.0775)	0.1018
Ginkgo+ADT	1.2406 (0.4330; 2.0481)	0.0026
Inositol+ADT	0.5352 (-0.3685; 1.4389)	0.2458
l-arginine+ADT	0.8437 (-0.4116; 2.0990)	0.1877
L-theanine+ADT	3.6276 (2.2953; 4.9599)	< 0.0001
Lavandula	0.1547 (-1.1120; 1.4214)	0.8108
Lavandula+ADT	0.9654 (-0.3035; 2.2342)	0.1359
Mg	0.7002 (-0.1730; 1.5735)	0.116
Mg+ADT	0.8830 (-0.3738; 2.1398)	0.1685
n-3	0.6007 (-0.5419; 1.7433)	0.3028
n-3+ADT	0.2192 (-0.9223; 1.3608)	0.7066
Nepta	1.3185 (0.1605; 2.4764)	0.0256
One-C	0.4906 (0.1615; 0.8197)	0.0035
One-C+ADT	0.8016 (0.4137; 1.1896)	< 0.0001
One-C+EPA+DHA+5HTP+Zinc+ADT	0.2690 (-0.8376; 1.3756)	0.6337
One-C+Probiotics	0.4846 (-0.6179; 1.5872)	0.389
One-C+Tryptophan	-0.8331 (-2.2552; 0.5889)	0.2509
PEA+ADT	1.0441 (-0.1321; 2.2203)	0.0819
Placebo	.	.

Probiotics+ADT	1.1555 (0.6907; 1.6202)	< 0.0001
Probiotics+B7	-0.1128 (-1.2494; 1.0238)	0.8458
Probiotics+Mg+CoQ10+ADT	0.8573 (-0.2464; 1.9610)	0.1279
R_rosea	0.9038 (0.1193; 1.6884)	0.0239
Saffron	0.6874 (0.3388; 1.0361)	0.0001
Saffron+ADT	3.7841 (2.3715; 5.1966)	< 0.0001
SJW	0.4541 (0.2280; 0.6801)	< 0.0001
SJW+ADT	0.5782 (-0.6352; 1.7916)	0.3503
Zinc	1.1722 (0.1366; 2.2078)	0.0265
Zinc+ADT	1.5847 (0.6225; 2.5468)	0.0012
Zinc+Vitamin_D	1.2763 (0.2420; 2.3105)	0.0156

Abbreviation: One-C: one Carbon cycle group

13.14. Sensitivity analysis for only omega-3 polyunsaturated fatty acids group

13.14-A. Network plot for only omega-3 polyunsaturated fatty acids group

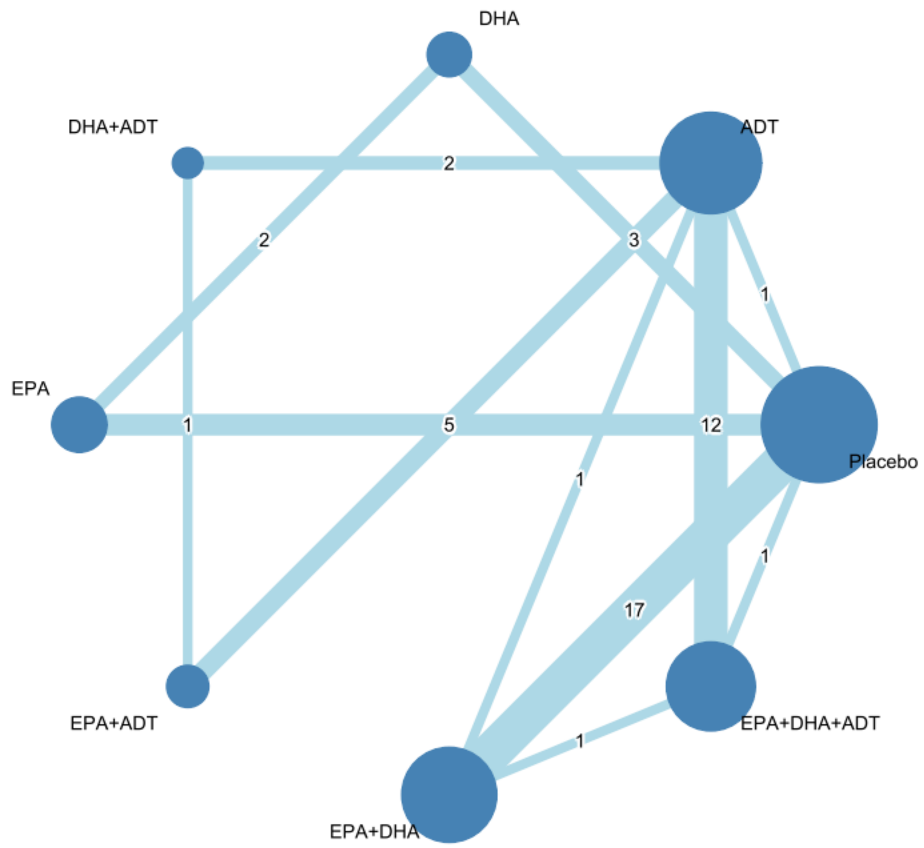

13.14-B. Forest Plot for only omega-3 polyunsaturated fatty acids group

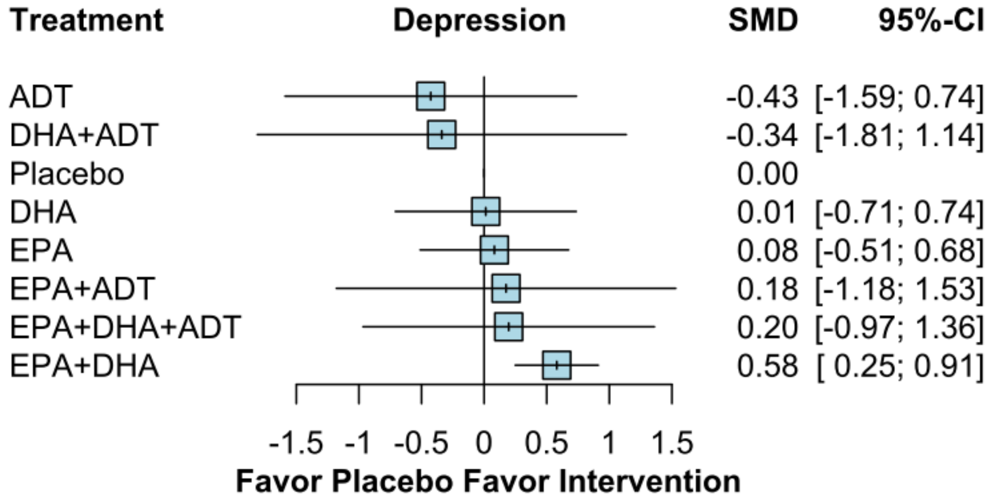

13.14-C. Detailed result for only omega-3 polyunsaturated fatty acids group

Intervention	SMD95%-CI	p-value
ADT	-0.4261 (-1.5902; 0.7380)	0.4731
DHA	0.0131 (-0.7096; 0.7358)	0.9716
DHA+ADT	-0.3383 (-1.8119; 1.1353)	0.6527
EPA	0.0820 (-0.5116; 0.6755)	0.7867
EPA+ADT	0.1750 (-1.1804; 1.5304)	0.8002
EPA+DHA	0.5801 (0.2479; 0.9124)	0.0006
EPA+DHA+ADT	0.1963 (-0.9679; 1.3605)	0.741
Placebo	.	.

13.15. Sensitivity analysis for omega 3 group with studies using omega 3 greater than 1g

13.15-A. Network plot for omega 3 group with studies using omega 3 greater than 1g

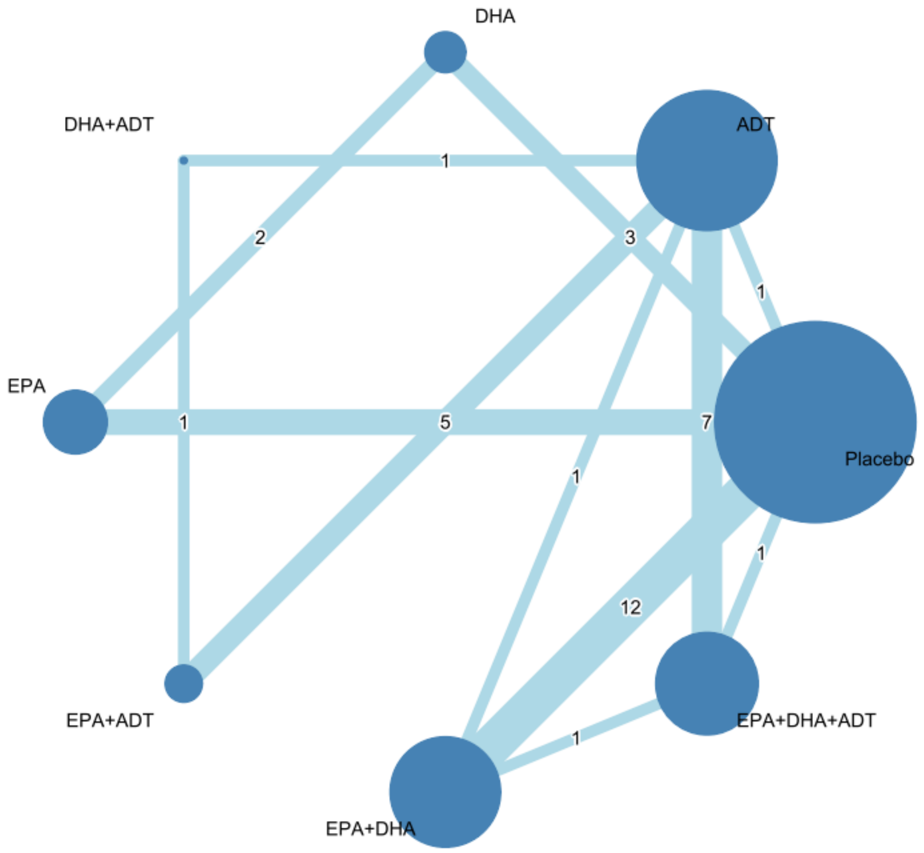

13.15-B. Forest Plot for omega 3 group with studies using omega 3 greater than 1g

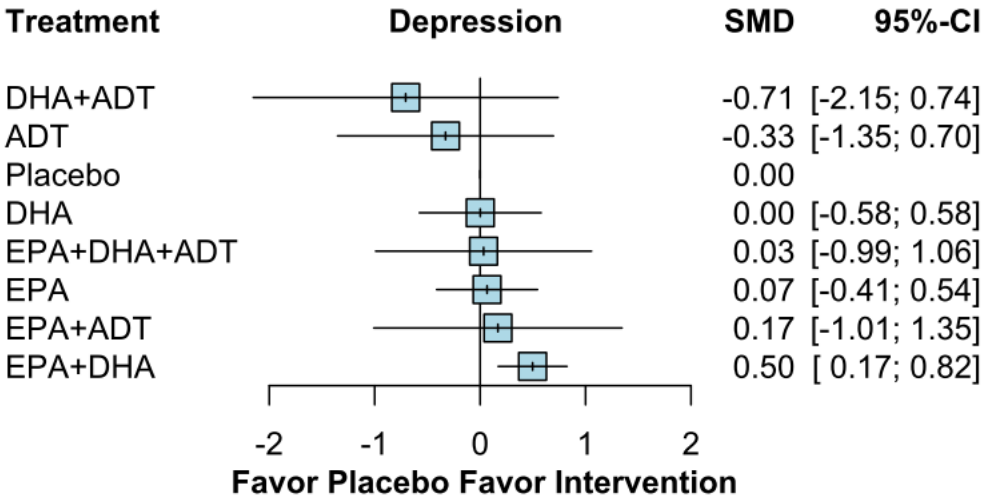

13.15-C. Detailed result for omega 3 group with studies using omega 3 greater than 1g

Intervention	SMD95%-CI	p-value
ADT	-0.3282 (-1.3522; 0.6957)	0.5298
DHA	0.0011 (-0.5769; 0.5792)	0.997
DHA+ADT	-0.7062 (-2.1518; 0.7393)	0.3383
EPA	0.0658 (-0.4128; 0.5445)	0.7875
EPA+ADT	0.1687 (-1.0089; 1.3463)	0.7788
EPA+DHA	0.4976 (0.1704; 0.8249)	0.0029
EPA+DHA+ADT	0.0328 (-0.9915; 1.0570)	0.95
Placebo	.	.

13.16. Sensitivity analysis for only St. John's wort group

13.16-A. Network plot for only St. John's wort group

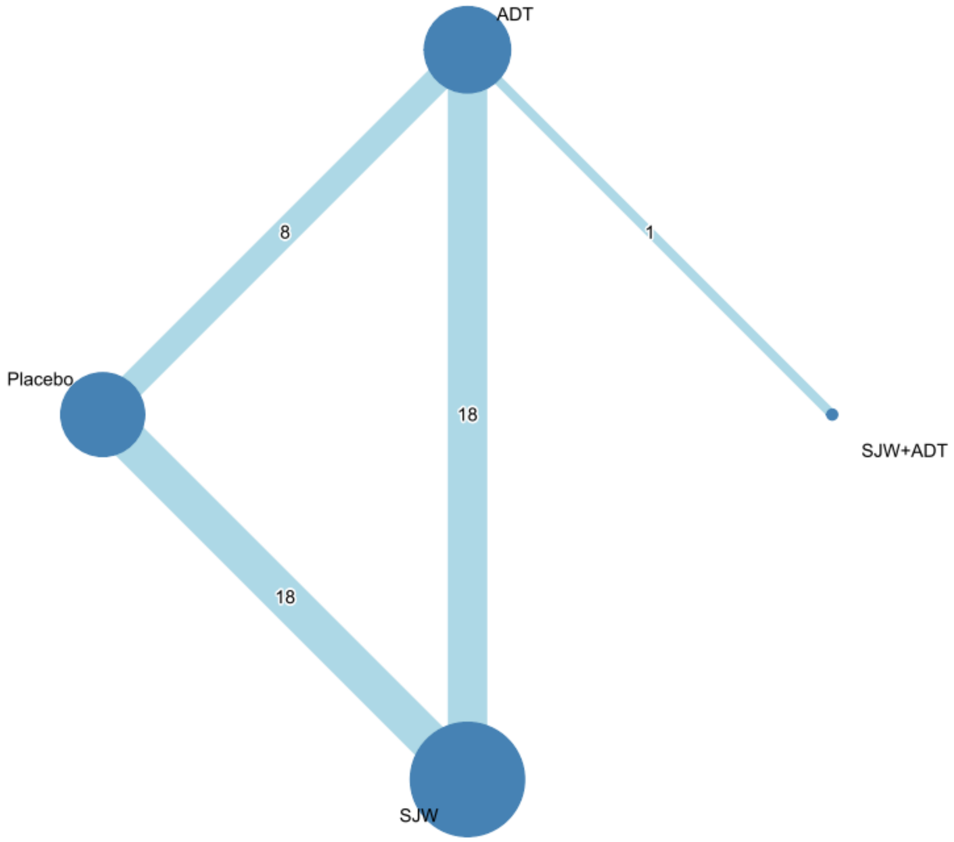

13.16-B. Forest Plot for only St. John's wort group

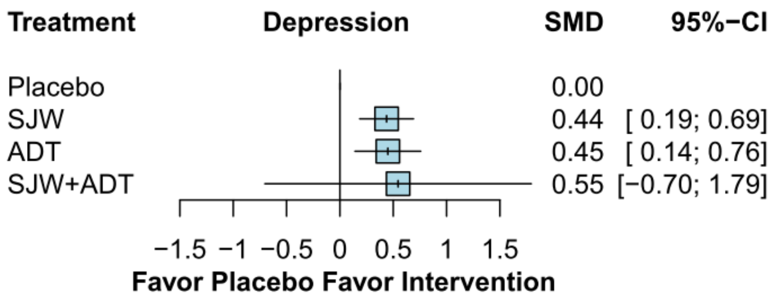

13.16-C. Detailed result for only St. John's wort group

Intervention	SMD95%-CI	p-value
ADT	0.4496 (0.1411; 0.7582)	0.0043
Placebo	.	.
SJW	0.4378 (0.1852; 0.6904)	0.0007
SJW+ADT	0.5454 (-0.7038; 1.7947)	0.3921

13.17. Sensitivity analysis for only Saffron

13.17-A. Network plot for only Saffron

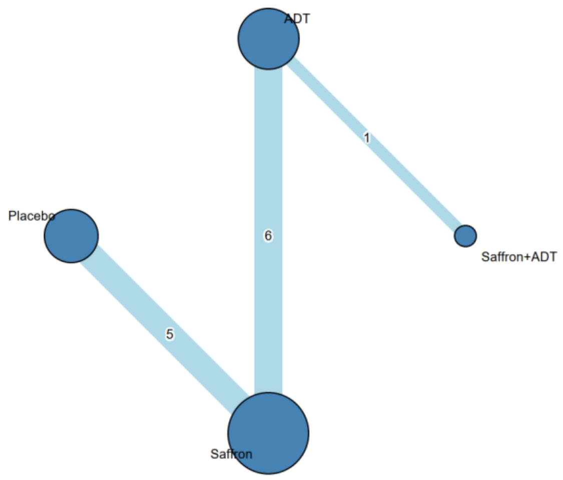

13.17-B. Forest Plot for only Saffron

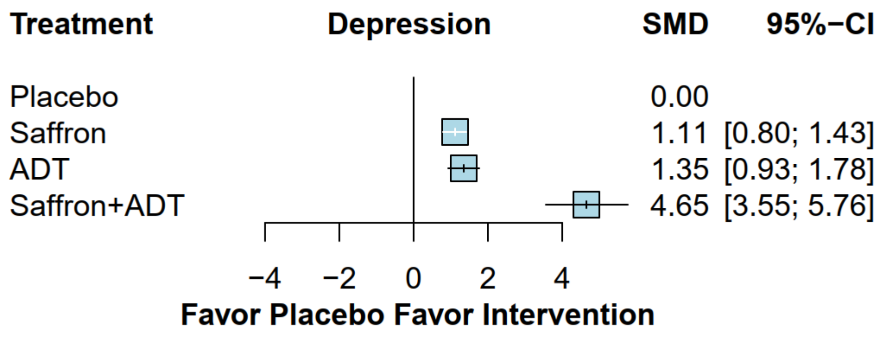

13.17-C. Detailed result for only Saffron

Intervention	SMD95%-CI	p-value
ADT	1.3532 (0.9289; 1.7776)	< 0.0001
Placebo	.	.
Saffron	1.1131 (0.7977; 1.4285)	< 0.0001
Saffron+ADT	4.6548 (3.5482; 5.7614)	< 0.0001

13.18. Sensitivity analysis for only SAMe

13.18-A. Network plot for only SAMe

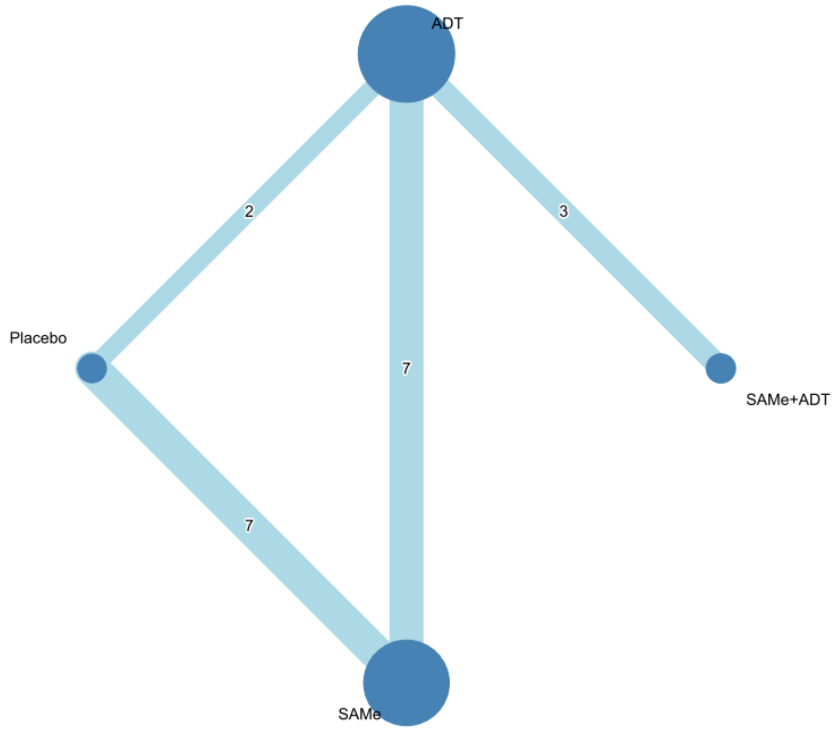

13.18-B. Forest Plot for only SAMe

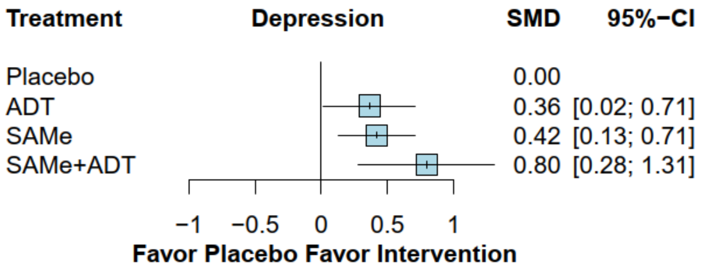

13.18-C. Detailed result for only SAMe

Intervention	SMD95%-CI	p-value
ADT	0.3635 (0.0179; 0.7090)	0.0392
Placebo	.	.
SAMe	0.4224 (0.1329; 0.7119)	0.0042
SAMe+ADT	0.7951 (0.2779; 1.3124)	0.0026

13.19. Sensitivity analysis for only Curcumin

13.19-A. Network plot for only Curcumin

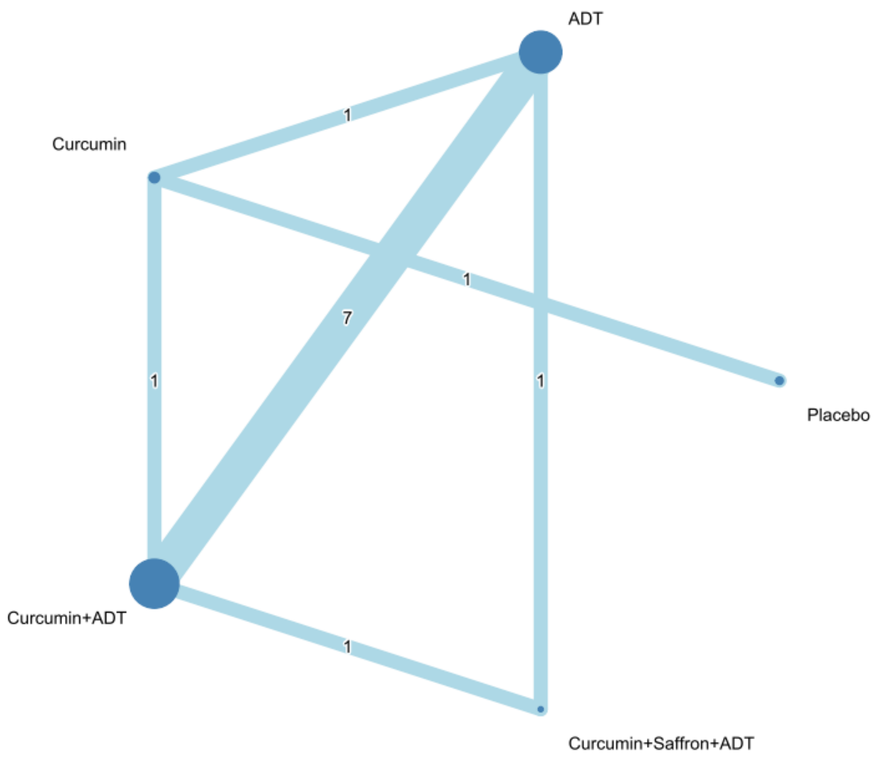

13.19-B. Forest Plot for only Curcumin

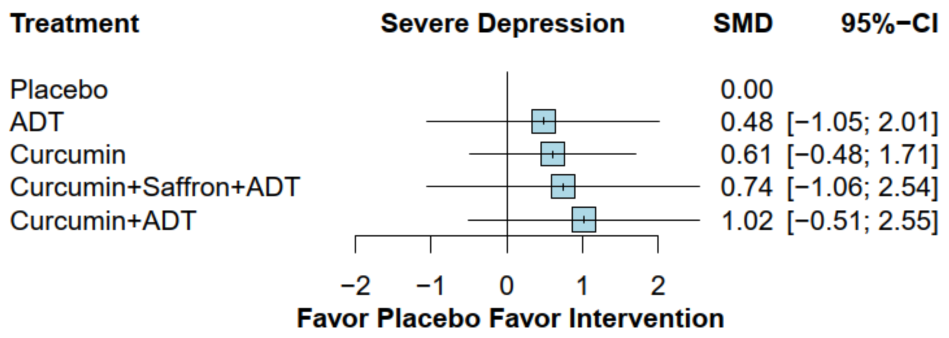

13.19-C. Detailed result for only Curcumin

**Intervention**	**SMD95%-CI**	**p-value**
ADT	0.4833 (-1.0477; 2.0144)	0.5361
Curcumin	0.6136 (-0.4815; 1.7087)	0.2721
Curcumin+ADT	1.0215 (-0.5091; 2.5521)	0.1909
Curcumin+Saffron+ADT	0.7440 (-1.0551; 2.5432)	0.4176
Placebo	.	.

13.20..Detailed result of the subgroup analysis (patient without comorbidity versus with or unclear comorbidity)

	Primary outcome	Without Comorbidity	With or unclear comorbidity
	SMD (95%CI)	SMD (95%CI)	SMD (95%CI)
ADT	0.4910 (0.2672; 0.7148)	0.5412 (0.2762; 0.8062)	0.3455 (-0.2403; 0.9314)
Amino_acid+ADT	2.0780 (0.8187; 3.3372)	2.0901 (0.8223; 3.3578)	1.9325 (0.3653; 3.4996)
Carnitine	1.0878 (0.2316; 1.9439)	1.1166 (0.2615; 1.9716)	
Carnitine+ADT	1.8424 (0.5964; 3.0884)		1.6969 (0.1404; 3.2534)
Chlorella+ADT	0.9671 (-0.1526; 2.0868)	1.0173 (-0.1037; 2.1383)	
Creatine+ADT	1.0994 (0.1575; 2.0412)	1.1504 (0.2025; 2.0982)	
Curcumin	0.6174 (-0.1657; 1.4005)	0.6714 (-0.4364; 1.7791)	0.6136 (-0.7314; 1.9585)
Curcumin+ADT	1.0285 (0.5519; 1.5050)	1.0792 (0.5770; 1.5813)	
Curcumin+Saffron+ADT	0.7512 (-0.2780; 1.7805)	0.8018 (-0.2321; 1.8357)	
DHA	0.0047 (-0.6089; 0.6184)	0.0235 (-0.7388; 0.7858)	-0.0131 (-1.2627; 1.2365)
DHA+ADT	0.3437 (-0.4601; 1.1475)	0.7249 (-0.0946; 1.5444)	
E_amoenum	0.7064 (-0.5020; 1.9148)	0.7064 (-0.4949; 1.9077)	
EPA	0.0706 (-0.4363; 0.5776)	0.0187 (-0.6227; 0.6601)	0.1493 (-0.8376; 1.1361)
EPA+ADT	1.0334 (0.3920; 1.6748)	1.4710 (0.7002; 2.2419)	0.1739 (-1.2607; 1.6085)
EPA+DHA	0.5994 (0.3165; 0.8823)	0.4730 (0.1072; 0.8388)	0.7751 (0.2465; 1.3038)
EPA+DHA+ADT	1.0373 (0.6378; 1.4367)	1.2424 (0.7704; 1.7145)	0.4955 (-0.4145; 1.4055)
EPA+DHA+Vitamin_C	0.1113 (-0.8700; 1.0925)	0.0482 (-0.9333; 1.0297)	
Fe	0.9453 (-0.1837; 2.0743)	0.9453 (-0.1726; 2.0631)	0.9453 (-0.4155; 2.3061)
Folate+ADT	0.6396 (0.1747; 1.1045)	0.6828 (0.1754; 1.1902)	0.5421 (-0.9570; 2.0412)
Folate+Vitamin_B12+Vitamin_B6	0.1801 (-0.8836; 1.2437)		
Ginkgo+ADT	1.2490 (0.4436; 2.0544)	0.9634 (-0.1317; 2.0584)	1.4708 (-0.0034; 2.9450)
Inositol+ADT	0.5437 (-0.3581; 1.4456)	0.5939 (-0.3143; 1.5021)	
l-arginine+ADT	0.8523 (-0.4002; 2.1048)		
L-theanine+ADT	3.6362 (2.3065; 4.9658)	3.6864 (2.3556; 5.0172)	
Lavandula	0.1633 (-1.1006; 1.4272)	0.2135 (-1.0515; 1.4786)	
Lavandula+ADT	0.9739 (-0.2921; 2.2399)	1.0242 (-0.2431; 2.2914)	
Mg	0.7039 (-0.1672; 1.5751)	0.6239 (-0.5240; 1.7717)	0.6610 (-0.9603; 2.2823)
Mg+ADT	0.8916 (-0.3624; 2.1455)	0.9418 (-0.3133; 2.1969)	
Nepta	1.3270 (0.1721; 2.4819)	1.3773 (0.2210; 2.5335)	
PEA+ADT	1.0527 (-0.1206; 2.2259)	1.1029 (-0.0716; 2.2774)	
Placebo	.		.
Prebiotics+ADT	0.6606 (-0.2810; 1.6022)	0.7102 (-0.2374; 1.6579)	

Probiotics	0.3526 (-0.2245; 0.9298)	0.1882 (-0.4647; 0.8411)	0.9051 (-0.5201; 2.3303)
Probiotics+ADT	1.1640 (0.7000; 1.6280)	1.2492 (0.7195; 1.7788)	0.8965 (-0.2718; 2.0647)
Probiotics+B7	-0.1128 (-1.2462; 1.0206)		
Probiotics+Mg+CoQ10+ADT	0.8659 (-0.2346; 1.9664)	0.8780 (-0.2322; 1.9882)	
R_rosea	0.9063 (0.1237; 1.6889)	0.1091 (-0.9278; 1.1461)	
Saffron	0.6920 (0.3441; 1.0399)	0.6984 (0.3341; 1.0628)	0.7431 (0.1479; 1.3383)
Saffron+ADT	3.7926 (2.3826; 5.2027)	3.8048 (2.3871; 5.2224)	
SAMe	0.5239 (0.1789; 0.8689)	0.4218 (-0.1593; 1.0029)	0.6116 (-0.2370; 1.4602)
SAMe+ADT	0.9919 (0.3082; 1.6756)	1.2359 (0.0837; 2.3882)	
SAMe+EPA+DHA+Folate+5HTP+Zinc+ADT	0.2776 (-0.8258; 1.3810)	0.2897 (-0.8233; 1.4028)	
SAMe+Probiotics	0.4846 (-0.6146; 1.5839)		
SAMe+Vitamin_B12+Folate+ADT	0.3948 (-0.7380; 1.5276)		
SJW	0.4583 (0.2327; 0.6840)	0.4762 (0.0393; 0.9131)	0.5822 (-0.0662; 1.2306)
SJW+ADT	0.5868 (-0.6237; 1.7973)	0.5989 (-0.6204; 1.8182)	
SJW+Kava	0.3768 (-0.8849; 1.6386)		
Tryptophan+ADT	1.2394 (0.3185; 2.1603)	1.4749 (0.1772; 2.7726)	0.8752 (-0.7060; 2.4563)
Zinc	1.1722 (0.1393; 2.2051)		1.2456 (-0.0243; 2.5155)
Zinc+ADT	1.5929 (0.6328; 2.5531)	1.6041 (0.6269; 2.5812)	1.4653 (0.2318; 2.6988)
Zinc+Vitamin_D	1.2763 (0.2448; 2.3078)		1.3498 (0.0809; 2.6187)

ADT: Antidepressant; Ca: Calcium ; DHA: Docosahexaenoic Acid ; E Amoenum : Echium amoenum; EPA :Eicosapentaenoic acid; Fe : Ferrum; Mg: Magnesium; PEA: Palmitoylethanolamide; R rosea : Rhodiola rosea; SAMe: S-Adenosyl Methionine ; SJW: St. John's wort ;Vitamin B1: Thiamine ; Vitamin B6: Pyridoxine; Vitamin B7: Biotin; Vitamin B: Vitamin B complex; Vitamin B12 : Cobalamin; Vitamin C: Ascorbic acid; Vitamin D: Cholecalciferol; 5HTP: 5-Hydroxytryptophan

13.21.Detailed result of the subgroup analysis (patient without industry sponsorship versus with or unclear industry sponsorship)

	Primary outcome	Without industry	With or uncealr industry
	SMD (95%CI)	SMD (95%CI)	SMD (95%CI)
ADT	0.4910 (0.2672; 0.7148)	0.5031 (0.1924; 0.8139)	0.4198 (0.0713; 0.7683)
Amino_acid+ADT	2.0780 (0.8187; 3.3372)	0.5031 (0.1924; 0.8139)	0.4198 (0.0713; 0.7683)
Carnitine	1.0878 (0.2316; 1.9439)	2.0901 (0.8223; 3.3578)	
Carnitine+ADT	1.8424 (0.5964; 3.0884)	0.8496 (-0.2893; 1.9885)	1.4393 (0.0751; 2.8035)
Chlorella+ADT	0.9671 (-0.1526; 2.0868)		1.7712 (0.4433; 3.0992)
Creatine+ADT	1.0994 (0.1575; 2.0412)		0.8959 (-0.3143; 2.1061)
Curcumin	0.6174 (-0.1657; 1.4005)	1.1126 (0.1531; 2.0722)	
Curcumin+ADT	1.0285 (0.5519; 1.5050)	0.5749 (-0.2118; 1.3615)	
Curcumin+Saffron+ADT	0.7512 (-0.2780; 1.7805)	0.8548 (0.2760; 1.4335)	1.4158 (0.5091; 2.3226)
DHA	0.0047 (-0.6089; 0.6184)	0.6671 (-0.3826; 1.7169)	
DHA+ADT	0.3437 (-0.4601; 1.1475)	-0.0908 (-0.7944; 0.6128)	0.2823 (-0.9882; 1.5527)
E_amoenum	0.7064 (-0.5020; 1.9148)	0.8421 (-0.0196; 1.7039)	
EPA	0.0706 (-0.4363; 0.5776)	0.7064 (-0.4916; 1.9044)	
EPA+ADT	1.0334 (0.3920; 1.6748)	0.0448 (-0.4658; 0.5555)	
EPA+DHA	0.5994 (0.3165; 0.8823)	0.3315 (-0.7681; 1.4312)	0.9403 (-0.1089; 1.9894)
EPA+DHA+ADT	1.0373 (0.6378; 1.4367)	0.6105 (0.3230; 0.8980)	0.3636 (-0.9672; 1.6944)
EPA+DHA+Vitamin_C	0.1113 (-0.8700; 1.0925)	1.0271 (0.5588; 1.4954)	1.0983 (0.1499; 2.0468)
Fe	0.9453 (-0.1837; 2.0743)	0.1168 (-0.8552; 1.0887)	
Folate+ADT	0.6396 (0.1747; 1.1045)		
Folate+Vitamin_B12+Vitamin_B6	0.1801 (-0.8836; 1.2437)	0.5245 (-0.3326; 1.3817)	0.6131 (0.0066; 1.2196)
Ginkgo+ADT	1.2490 (0.4436; 2.0544)		
Inositol+ADT	0.5437 (-0.3581; 1.4456)	1.2608 (0.4346; 2.0870)	
l-arginine+ADT	0.8523 (-0.4002; 2.1048)	0.6511 (-0.6426; 1.9447)	0.3860 (-0.9250; 1.6969)
L-theanine+ADT	3.6362 (2.3065; 4.9658)		
Lavandula	0.1633 (-1.1006; 1.4272)	3.6483 (2.3106; 4.9860)	
Lavandula+ADT	0.9739 (-0.2921; 2.2399)	0.1754 (-1.0969; 1.4478)	
Mg	0.7039 (-0.1672; 1.5751)	0.9861 (-0.2884; 2.2605)	
Mg+ADT	0.8916 (-0.3624; 2.1455)	0.7091 (-0.1599; 1.5780)	
Nepta	1.3270 (0.1721; 2.4819)	0.9037 (-0.3588; 2.1662)	
PEA+ADT	1.0527 (-0.1206; 2.2259)	1.3392 (0.1750; 2.5033)	
Placebo	.		
Prebiotics+ADT	0.6606 (-0.2810; 1.6022)		
Probiotics	0.3526 (-0.2245; 0.9298)		
Probiotics+ADT	1.1640 (0.7000; 1.6280)	0.3176 (-0.4916; 1.1267)	0.3910 (-0.4664; 1.2484)

Probiotics+B7	-0.1128 (-1.2462; 1.0206)	1.1759 (0.6674; 1.6844)	
Probiotics+Mg+CoQ10+ADT	0.8659 (-0.2346; 1.9664)		-0.1128 (-1.3062; 1.0806)
R_rosea	0.9063 (0.1237; 1.6889)		
Saffron	0.6920 (0.3441; 1.0399)	0.1091 (-0.9278; 1.1461)	1.9657 (0.7207; 3.2108)
Saffron+ADT	3.7926 (2.3826; 5.2027)		
SAMe	0.5239 (0.1789; 0.8689)		
SAMe+ADT	0.9919 (0.3082; 1.6756)		0.5505 (0.0981; 1.0029)
SAMe+EPA+DHA+Folate+5HTP+Zinc+ADT	0.2776 (-0.8258; 1.3810)		0.8110 (-0.0931; 1.7151)
SAMe+Probiotics	0.4846 (-0.6146; 1.5839)		
SAMe+Vitamin_B12+Folate+ADT	0.3948 (-0.7380; 1.5276)		
SJW	0.4583 (0.2327; 0.6840)		0.3236 (-0.8987; 1.5460)
SJW+ADT	0.5868 (-0.6237; 1.7973)		0.4221 (0.1349; 0.7093)
SJW+Kava	0.3768 (-0.8849; 1.6386)		
Tryptophan+ADT	1.2394 (0.3185; 2.1603)		0.3768 (-0.9390; 1.6927)
Zinc	1.1722 (0.1393; 2.2051)		0.9495 (-0.4072; 2.3062)
Zinc+ADT	1.5929 (0.6328; 2.5531)		1.2507 (0.1337; 2.3676)
Zinc+Vitamin_D	1.2763 (0.2448; 2.3078)		

ADT: Antidepressant; Ca: Calcium ; DHA: Docosahexaenoic Acid ; E Amoenum : Echium amoenum; EPA :Eicosapentaenoic acid; Fe : Ferrum; Mg: Magnesium; PEA: Palmitoylethanolamide; R rosea : Rhodiola rosea; SAMe: S-Adenosyl Methionine ; SJW: St. John's wort ;Vitamin B1: Thiamine ; Vitamin B6: Pyridoxine; Vitamin B7: Biotin; Vitamin B: Vitamin B complex; Vitamin B12 : Cobalamin; Vitamin C: Ascorbic acid; Vitamin D: Cholecalciferol; 5HTP: 5-Hydroxytryptophan

13.22.Detailed result of the subgroup analysis (study duration less than 8 week versus greater than 8 week)

	Primary outcome	< 8 week	> 8week
	SMD (95%CI)	SMD (95%CI)	SMD (95%CI)
ADT	0.4910 (0.2672; 0.7148)	0.6831 (0.2963;	0.3465 (0.0580; 0.6350
Amino_acid+ADTCurcumin	0.6174 (-0.1657; 1.4005)	2.2701 (0.9134; 3.6267)	
Curcumin+ADT	1.0285 (0.5519; 1.5050)	1.4393 (0.0694; 2.8093)	0.6929 (-0.4146;
Curcumin+Saffron+ADT	0.7512 (-0.2780; 1.7805)		1.6979 (0.4718; 2.9240)
DHA	0.0047 (-0.6089; 0.6184)	1.1592 (-0.0690; 2.3874)	
DHA+ADT	0.3437 (-0.4601; 1.1475)	0.5281 (-0.9524; 2.0085)	1.5634 (0.3366; 2.7902)
E_amoenum	0.7064 (-0.5020; 1.9148)	0.8752 (-0.3425; 2.0929)	0.6136 (-0.4586; 1.6857)
EPA	0.0706 (-0.4363; 0.5776)	1.3443 (0.6250; 2.0636)	0.7216 (0.0317; 1.4115)
EPA+ADT	1.0334 (0.3920; 1.6748)		0.5228 (-0.5214; 1.5669)
EPA+DHA	0.5994 (0.3165; 0.8823)	0.2823 (-0.9943; 1.5588)	-0.0918 (-0.7778;
EPA+DHA+ADT	1.0373 (0.6378; 1.4367)		0.3893 (-0.4163;
EPA+DHA+Vitamin_C	0.1113 (-0.8700; 1.0925)	0.7064 (-0.5646; 1.9774)	
Fe	0.9453 (-0.1837; 2.0743)		0.0429 (-0.4560;
Folate+ADT	0.6396 (0.1747; 1.1045)	2.1941 (0.6699; 3.7182)	0.7213 (0.0167; 1.4260)
Folate+Vitamin_B12+Vitamin_B6	0.1801 (-0.8836; 1.2437)	0.5894 (-0.0442; 1.2229)	0.5880 (0.2642; 0.9118)
Ginkgo+ADT	1.2490 (0.4436; 2.0544)	3.2583 (1.9152; 4.6014)	0.7998 (0.3494; 1.2502)
Inositol+ADT	0.5437 (-0.3581; 1.4456)	0.1063 (-0.9704; 1.1830	
l-arginine+ADT	0.8523 (-0.4002; 2.1048)	0.9453 (-0.2505; 2.1411)	
L-theanine+ADT	3.6362 (2.3065; 4.9658)	0.7917 (0.0597; 1.5237)	0.5351 (-0.0819; 1.1521)
Lavandula	0.1633 (-1.1006; 1.4272)		0.1801 (-0.8442; 1.2044)
Lavandula+ADT	0.9739 (-0.2921; 2.2399)		1.1034 (0.3030; 1.9037)
Mg	0.7039 (-0.1672; 1.5751)	0.7363 (-0.2590; 1.7315)	
Mg+ADT	0.8916 (-0.3624; 2.1455)	0.3554 (-1.0056; 1.7164)	
Nepta	1.3270 (0.1721; 2.4819)	1.1660 (-0.1969; 2.5290)	
PEA+ADT	1.0527 (-0.1206; 2.2259)		0.6404 (-0.2100; 1.4909)
Placebo	.		0.7470 (-0.4872; 1.9813)
Prebiotics+ADT	0.6606 (-0.2810; 1.6022)	1.5191 (0.2587; 2.7795)	
Probiotics	0.3526 (-0.2245; 0.9298)	1.2448 (-0.0324; 2.5220)	
Probiotics+ADT	1.1640 (0.7000; 1.6280)		
Probiotics+B7	-0.1128 (-1.2462;	1.3151 (0.0119; 2.6182)	1.1225 (-0.2433; 2.4883)
Probiotics+Mg+CoQ10+ADT	0.8659 (-0.2346; 1.9664)		0.3506 (-0.2083; 0.9095)
R_rosea	0.9063 (0.1237; 1.6889)		1.1041 (0.4739; 1.7342)
Saffron	0.6920 (0.3441; 1.0399)	-0.1128 (-1.3127; 1.0872)	
Saffron+ADT	3.7926 (2.3826; 5.2027)	1.9657 (0.7144; 3.2171)	0.0304 (-0.9840; 1.0448)
SAMe	0.5239 (0.1789; 0.8689)	0.8152 (0.2901; 1.3403)	0.5327 (0.0169; 1.0484)
SAMe+ADT	0.9919 (0.3082; 1.6756)	3.9847 (2.4871; 5.4824)	
SAMe+EPA+DHA+Folate+5HTP+Zinc+AD	0.2776 (-0.8258; 1.3810)	0.7215 (0.2249; 1.2181)	0.3395 (-0.1660; 0.8449)
SAMe+Probiotics	0.4846 (-0.6146; 1.5839)	1.1883 (0.4014; 1.9753)	
SAMe+Vitamin_B12+Folate+ADT	0.3948 (-0.7380; 1.5276)		0.1331 (-0.9478; 1.2140)
SJW	0.4583 (0.2327; 0.6840)	0.4846 (-0.6831; 1.6524)	

SJW+ADT	0.5868 (-0.6237; 1.7973)		0.2503 (-0.8606; 1.3612)
SJW+Kava	0.3768 (-0.8849; 1.6386)	0.6336 (0.2933; 0.9739)	0.2876 (-0.0350; 0.6103)
Tryptophan+ADT	1.2394 (0.3185; 2.1603)	0.7789 (-0.5326; 2.0904)	
Zinc	1.1722 (0.1393; 2.2051)		0.3768 (-0.8518; 1.6055)
Zinc+ADT	1.5929 (0.6328; 2.5531)	1.2128 (-0.1600; 2.5855)	1.3182 (0.0480; 2.5884)
Zinc+Vitamin_D	1.2763 (0.2448; 2.3078)		0.3070 (-0.8278; 1.4418)
ADT	0.4910 (0.2672; 0.7148)		0.5372 (-0.6179; 1.6924)
Amino_acid+ADT	2.0780 (0.8187; 3.3372)	2.2121 (0.9439; 3.4804)	
Carnitine	1.0878 (0.2316; 1.9439)	-0.6325 (-2.1391; 0.8742)	
Carnitine+ADT	1.8424 (0.5964; 3.0884)	0.1322 (-0.9444; 1.2089)	
Chlorella+ADT	0.9671 (-0.1526; 2.0868)		0.2880 (-0.8907; 1.4666)
Creatine+ADT	1.0994 (0.1575; 2.0412)		0.7084 (0.3263; 1.0905)
Curcumin	0.6174 (-0.1657; 1.4005)		0.7990 (0.2221; 1.3760)
Curcumin+ADT	1.0285 (0.5519; 1.5050)		0.2362 (-0.7484; 1.2208)
Curcumin+Saffron+ADT	0.7512 (-0.2780; 1.7805)		1.1728 (0.1715; 2.1740)
DHA	0.0047 (-0.6089; 0.6184)		1.4450 (0.4908; 2.3991)
DHA+ADT	0.3437 (-0.4601; 1.1475)		1.2769 (0.2770; 2.2767

ADT: Antidepressant; Ca: Calcium ; DHA: Docosahexaenoic Acid ; E Amoenum : Echium amoenum; EPA :Eicosapentaenoic acid; Fe : Ferrum; Mg: Magnesium; PEA: Palmitoylethanolamide; R rosea : Rhodiola rosea; SAMe: S-Adenosyl Methionine ; SJW: St. John's wort ;Vitamin B1: Thiamine ; Vitamin B6: Pyridoxine; Vitamin B7: Biotin; Vitamin B: Vitamin B complex; Vitamin B12 : Cobalamin; Vitamin C: Ascorbic acid; Vitamin D: Cholecalciferol; 5HTP: 5-Hydroxytryptophan

13.23.Detailed result of the subgroup analysis (study publication before 2013 versus after 2013

	Primary outcome	Before 2013	After 2013
	SMD (95%CI)	SMD (95%CI)	SMD (95%CI)
ADT	0.4910 (0.2672; 0.7148)	0.4760 (0.2091; 0.7429)	0.4651 (-0.0019; 0.9321)
Amino_acid+ADT	2.0780 (0.8187; 3.3372)	2.0629 (0.7976; 3.3283)	
Carnitine	1.0878 (0.2316; 1.9439)	1.4393 (0.1295; 2.7491)	0.8115 (-0.4513; 2.0744)
Carnitine+ADT	1.8424 (0.5964; 3.0884)		1.8165 (0.4484; 3.1846)
Chlorella+ADT	0.9671 (-0.1526; 2.0868)		0.9412 (-0.3129; 2.1953)
Creatine+ADT	1.0994 (0.1575; 2.0412)	1.6929 (0.4401; 2.9457)	0.3100 (-1.1920; 1.8120)
Curcumin	0.6174 (-0.1657; 1.4005)		0.6035 (-0.2483; 1.4553)
Curcumin+ADT	1.0285 (0.5519; 1.5050)		1.0020 (0.3667; 1.6372)
Curcumin+Saffron+ADT	0.7512 (-0.2780; 1.7805)		0.7244 (-0.4329; 1.8817)
DHA	0.0047 (-0.6089; 0.6184)	0.2823 (-0.9295; 1.4940)	-0.0465 (-0.8595; 0.7665)
DHA+ADT	0.3437 (-0.4601; 1.1475)	1.0704 (-0.0718; 2.2126)	0.0909 (-1.1609; 1.3427)
E_amoenum	0.7064 (-0.5020; 1.9148)	0.7064 (-0.4995; 1.9123)	
EPA	0.0706 (-0.4363; 0.5776)	-0.0280 (-0.7249; 0.6689)	0.1333 (-0.6791; 0.9458)
EPA+ADT	1.0334 (0.3920; 1.6748)	0.9898 (0.0013; 1.9782)	0.9767 (0.0108; 1.9425)
EPA+DHA	0.5994 (0.3165; 0.8823)	0.5931 (0.1451; 1.0411)	0.5979 (0.2129; 0.9830)
EPA+DHA+ADT	1.0373 (0.6378; 1.4367)	0.8138 (0.2869; 1.3407)	1.2441 (0.5495; 1.9388)
EPA+DHA+Vitamin_C	0.1113 (-0.8700; 1.0925)		0.1105 (-0.9350; 1.1561)
Fe	0.9453 (-0.1837; 2.0743)		0.9453 (-0.2488; 2.1393)
Folate+ADT	0.6396 (0.1747; 1.1045)	0.6502 (0.1334; 1.1670)	0.4747 (-0.7231; 1.6725)
Folate+Vitamin_B12+Vitamin_B6	0.1801 (-0.8836; 1.2437)		0.1801 (-0.9524; 1.3126)
Ginkgo+ADT	1.2490 (0.4436; 2.0544)		1.2249 (0.2802; 2.1695)
Inositol+ADT	0.5437 (-0.3581; 1.4456)	0.5287 (-0.3831; 1.4406)	
l-arginine+ADT	0.8523 (-0.4002; 2.1048)		
L-theanine+ADT	3.6362 (2.3065; 4.9658)		3.6103 (2.1656; 5.0549)
Lavandula	0.1633 (-1.1006; 1.4272)	0.1483 (-1.1216; 1.4182)	
Lavandula+ADT	0.9739 (-0.2921; 2.2399)	0.9589 (-0.3131; 2.2310)	
Mg	0.7039 (-0.1672; 1.5751)	0.7914 (-0.5403; 2.1232)	0.6239 (-0.5951; 1.8428)
Mg+ADT	0.8916 (-0.3624; 2.1455)		0.8657 (-0.5096; 2.2410)
Nepta	1.3270 (0.1721; 2.4819)		1.3011 (0.0155; 2.5868)
PEA+ADT	1.0527 (-0.1206; 2.2259)		1.0267 (-0.2754; 2.3289)
Placebo	.		
Prebiotics+ADT	0.6606 (-0.2810; 1.6022)		0.6393 (-0.4241; 1.7027)
Probiotics	0.3526 (-0.2245; 0.9298)		0.3558 (-0.2533; 0.9649)
Probiotics+ADT	1.1640 (0.7000; 1.6280)		1.1392 (0.5050; 1.7735)

Probiotics+B7	-0.1128 (-1.2462; 1.0206)		-0.1128 (-1.3110; 1.0854)
Probiotics+Mg+CoQ10+ADT	0.8659 (-0.2346; 1.9664)		0.8400 (-0.3971; 2.0770)
R_rosea	0.9063 (0.1237; 1.6889)	1.9657 (0.7805; 3.1510)	0.0900 (-1.0226; 1.2026)
Saffron	0.6920 (0.3441; 1.0399)	0.7216 (0.1521; 1.2911)	0.6575 (0.1685; 1.1464)
Saffron+ADT	3.7926 (2.3826; 5.2027)		3.7667 (2.2477; 5.2857)
SAMe	0.5239 (0.1789; 0.8689)	0.6043 (0.1533; 1.0554)	0.3893 (-0.1806; 0.9593)
SAMe+ADT	0.9919 (0.3082; 1.6756)	1.2658 (0.4048; 2.1268)	0.4945 (-0.7169; 1.7059)
SAMe+EPA+DHA+Folate+5HTP+Zinc +ADT	0.2776 (-0.8258; 1.3810)		0.2517 (-0.9879; 1.4912)
SAMe+Probiotics	0.4846 (-0.6146; 1.5839)		
SAMe+Vitamin_B12+Folate+ADT	0.3948 (-0.7380; 1.5276)		0.3689 (-0.8969; 1.6347)
SJW	0.4583 (0.2327; 0.6840)	0.4508 (0.2142; 0.6875)	0.6336 (0.2933; 0.9739)
SJW+ADT	0.5868 (-0.6237; 1.7973)	0.5718 (-0.6450; 1.7886)	0.7789 (-0.5326; 2.0904)
SJW+Kava	0.3768 (-0.8849; 1.6386)	0.3768 (-0.8825; 1.6362)	
Tryptophan+ADT	1.2394 (0.3185; 2.1603)	1.2244 (0.2937; 2.1551)	1.2128 (-0.1600; 2.5855)
Zinc	1.1722 (0.1393; 2.2051)		1.1714 (0.0828; 2.2599)
Zinc+ADT	1.5929 (0.6328; 2.5531)	1.5777 (0.6082; 2.5472)	
Zinc+Vitamin_D	1.2763 (0.2448; 2.3078)		1.2755 (0.1882; 2.3628)

ADT: Antidepressant; Ca: Calcium ; DHA: Docosahexaenoic Acid ; E Amoenum : Echium amoenum; EPA :Eicosapentaenoic acid; Fe : Ferrum; Mg: Magnesium; PEA: Palmitoylethanolamide; R rosea : Rhodiola rosea; SAMe: S-Adenosyl Methionine ; SJW: St. John's wort ;Vitamin B1: Thiamine ; Vitamin B6: Pyridoxine; Vitamin B7: Biotin; Vitamin B: Vitamin B complex; Vitamin B12 : Cobalamin; Vitamin C: Ascorbic acid; Vitamin D: Cholecalciferol; 5HTP: 5-Hydroxytryptophan

13.24.Detailed result of the sensitivity analysis excluding high risk of bias

	Primary outcome	Excluding study with high risk study
	**SMD (95%CI)**	**SMD (95%CI)**
ADT	0.4910 (0.2672; 0.7148)	0.4544 (0.2352; 0.6736)
Amino_acid+ADT	2.0780 (0.8187; 3.3372)	2.0414 (0.8084; 3.2744)
Carnitine	1.0878 (0.2316; 1.9439)	1.0640 (0.2274; 1.9006)
Carnitine+ADT	1.8424 (0.5964; 3.0884)	1.8058 (0.5864; 3.0253)
Chlorella+ADT	0.9671 (-0.1526; 2.0868)	
Creatine+ADT	1.0994 (0.1575; 2.0412)	1.0658 (0.1423; 1.9893)
Curcumin	0.6174 (-0.1657; 1.4005)	0.5470 (-0.2188; 1.3128)
Curcumin+ADT	1.0285 (0.5519; 1.5050)	0.7961 (0.3004; 1.2918)
Curcumin+Saffron+ADT	0.7512 (-0.2780; 1.7805)	0.6133 (-0.3944; 1.6209)
DHA	0.0047 (-0.6089; 0.6184)	0.0032 (-0.5946; 0.6010)
DHA+ADT	0.3437 (-0.4601; 1.1475)	0.5482 (-0.2376; 1.3340)
E_amoenum	0.7064 (-0.5020; 1.9148)	0.7064 (-0.4754; 1.8882)
EPA	0.0706 (-0.4363; 0.5776)	0.0686 (-0.4258; 0.5630)
EPA+ADT	1.0334 (0.3920; 1.6748)	1.0304 (0.4031; 1.6576)
EPA+DHA	0.5994 (0.3165; 0.8823)	0.5956 (0.3194; 0.8717)
EPA+DHA+ADT	1.0373 (0.6378; 1.4367)	0.9985 (0.6079; 1.3890)
EPA+DHA+Vitamin_C	0.1113 (-0.8700; 1.0925)	0.1094 (-0.8468; 1.0656)
Fe	0.9453 (-0.1837; 2.0743)	0.9453 (-0.1553; 2.0458)
Folate+ADT	0.6396 (0.1747; 1.1045)	0.5293 (0.0161; 1.0426)
Folate+Vitamin_B12+Vitamin_B6	0.1801 (-0.8836; 1.2437)	
Ginkgo+ADT	1.2490 (0.4436; 2.0544)	1.5797 (0.4672; 2.6922)
Inositol+ADT	0.5437 (-0.3581; 1.4456)	0.5070 (-0.3759; 1.3898)
l-arginine+ADT	0.8523 (-0.4002; 2.1048)	
L-theanine+ADT	3.6362 (2.3065; 4.9658)	3.5996 (2.2948; 4.9044)
Lavandula	0.1633 (-1.1006; 1.4272)	
Lavandula+ADT	0.9739 (-0.2921; 2.2399)	
Mg	0.7039 (-0.1672; 1.5751)	0.6875 (-0.1647; 1.5397)
Mg+ADT	0.8916 (-0.3624; 2.1455)	0.8550 (-0.3726; 2.0825)
Nepta	1.3270 (0.1721; 2.4819)	1.2904 (0.1642; 2.4166)
PEA+ADT	1.0527 (-0.1206; 2.2259)	1.0161 (-0.1289; 2.1610)
Placebo	.	
Prebiotics+ADT	0.6606 (-0.2810; 1.6022)	0.6218 (-0.3015; 1.5451)
Probiotics	0.3526 (-0.2245; 0.9298)	0.3511 (-0.2120; 0.9142)
Probiotics+ADT	1.1640 (0.7000; 1.6280)	1.1268 (0.6738; 1.5798)
Probiotics+B7	-0.1128 (-1.2462; 1.0206)	-0.1128 (-1.2178; 0.9923)

Probiotics+Mg+CoQ10+ADT	0.8659 (-0.2346; 1.9664)	0.8293 (-0.2411; 1.8996)
R_rosea	0.9063 (0.1237; 1.6889)	0.8961 (0.1312; 1.6610)
Saffron	0.6920 (0.3441; 1.0399)	0.6717 (0.3318; 1.0117)
Saffron+ADT	3.7926 (2.3826; 5.2027)	3.7560 (2.3694; 5.1427)
SAMe	0.5239 (0.1789; 0.8689)	0.5041 (0.1671; 0.8410)
SAMe+ADT	0.9919 (0.3082; 1.6756)	0.9532 (0.2869; 1.6195)
SAMe+EPA+DHA+Folate+5HTP+Zinc+ADT	0.2776 (-0.8258; 1.3810)	0.2410 (-0.8323; 1.3143)
SAMe+Probiotics	0.4846 (-0.6146; 1.5839)	
SAMe+Vitamin_B12+Folate+ADT	0.3948 (-0.7380; 1.5276)	
SJW	0.4583 (0.2327; 0.6840)	0.3809 (0.1571; 0.6047)
SJW+ADT	0.5868 (-0.6237; 1.7973)	0.5502 (-0.6329; 1.7333)
SJW+Kava	0.3768 (-0.8849; 1.6386)	0.3768 (-0.8595; 1.6132)
Tryptophan+ADT	1.2394 (0.3185; 2.1603)	1.2027 (0.3003; 2.1051)
Zinc	1.1722 (0.1393; 2.2051)	1.1635 (0.1520; 2.1750)
Zinc+ADT	1.5929 (0.6328; 2.5531)	1.3642 (0.2059; 2.5225)
Zinc+Vitamin_D	1.2763 (0.2448; 2.3078)	1.2676 (0.2575; 2.2778)

ADT: Antidepressant; Ca: Calcium ; DHA: Docosahexaenoic Acid ; E Amoenum : Echium amoenum; EPA :Eicosapentaenoic acid; Fe : Ferrum; Mg: Magnesium; PEA: Palmitoylethanolamide; R rosea : Rhodiola rosea; SAMe: S-Adenosyl Methionine ; SJW: St. John's wort ;Vitamin B1: Thiamine ; Vitamin B6: Pyridoxine; Vitamin B7: Biotin; Vitamin B: Vitamin B complex; Vitamin B12 : Cobalamin; Vitamin C: Ascorbic acid; Vitamin D: Cholecalciferol; 5HTP: 5-Hydroxytryptophan

14.Heterogeneity /Inconsistency test result, I^2 and heterogeneity estimate τ 2

**Outcome**	**No. of studies**	**Total Heterogeneity**	**Heterogeneity (within designs)**	**Inconsistency (between designs)**	I^2 **(95%CI)**	**τ 2**
**SMD**	179	< 0.0001	< 0.0001	< 0.0001	84.5% (82.2%; 86.5 %)	0.2683
**Response rate**	86	< 0.0001	< 0.0001	< 0.0001	63.7% (52.7%; 72.0%)	0.3143
**Remission rate**	45	< 0.0001	< 0.0001	< 0.0001	70.9% (57.4%; 80.1%)	0.6529
**Anxiety symptom**	30	< 0.0001	< 0.0001	0.1879	93.6% (90.6%; 95.6%)	0.7004
**All cause discontinuation**	89	0.9881	0.9916	0.5538	0% (0.0%; 28.9%)	0
**Adverse event**	42	0.0004	0.0026	0.0178	52.6% (28.5%; 68.5%)	0.1522

15.Changes in heterogeneity for sensitivity analyses & Subgroup analysis

15.1.Heterogeneity for sensitivity analysis and subgroup analysis

Including only studies with	τ	% of variance explained	Number of studies included
Unadjusted (Primary outcome)	0.5180	84.5% (82.2%; 86.5%)	179
Mild depression	0.6062	86.5% (82.1%; 89.9%)	52
Moderate depression	0.4771	83.7% (79.9%; 86.7%)	93
Severe depression	0.6738	91.3% (86.4%; 94.4%)	31
Combine One Carbone cycle	0.5198	84.8% (82.6%; 86.8%)	179
Combine Omega 3	0.5137	84.4% (82.1%; 86.5%)	179
Excluding EPA<1g	0.4868	83% (80.3%; 85.4%)	167
Without comorbidity	0.5136	84.8% (82.0%; 87.1%)	136
With & Unclear comorbidity	0.6469	87.5% (82.9%; 90.8%)	43
Exclude high risk of bias	0.5018	83.7% (81.2%; 85.9%)	168
Without industry sponsorship	0.5116	80.2% (75.9%; 83.7%)	118
With industry or unclear sponsorship	0.5519	89.6% (86.9%; 91.7%)	61
<8 weeks	0.5556	88.5% (85.4%; 90.9%)	71
≥ 8 week	0.4902	81.7% (78.0%; 84.9%)	108
Publication year>2013	0.5546	83.1% (78.9%; 86.5%)	95
Publication year<2013	0.5566;	83.3% (78.9%; 86.8%)	84
Only Adjunctive	0.5580	82.6% (77.7%; 86.5%)	78
Only Monotherapy	0.5091	85.5% (82.9%; 87.8%)	104
Clinical Depression	0.5017	84.4% (81.7%; 86.8%)	133
Non-Clinical depression	0.6216	86.7% (81.6%; 90.4%)	47
Only HAMD	0.4914	83.9% (80.8%; 86.4%)	118

EPA :Eicosapentaenoic acid; HAMD: Hamilton Depression Rating Scale; Omega 3: Omega-3 polyunsaturated fatty acids

15.2 Heterogeneity of Network meta-analysis for specific nutraceuticals

**Including only studies with**	**τ**	**% of variance explained**	**Number of studies included**
Omega 3 group	0.6280	86.3% (82.2%; 89.5%)	39
Adjunctive Omega 3	0.8008	90.9% (86.4%; 93.8%)	15
Omega 3 monotherapy	0.5321	83.1% (75.2%; 88.4%)	21
Omega 3 >1g	0.4814	76.3% (65.8%; 83.6%)	28
SJW	0.5304	90.9% (88.3%; 92.9%)	29
SJW monotherapy	0.6580	93.7% (90.8%; 95.7%)	12
Saffron	0.1888	28% (0.0%; 65.5%)	12
SAMe	0.2549	54.8% (19.1%; 74.7%)	15
Adjunctive SAMe	0.2774	65.3% (29.4%; 83.0%)	10
SAMe monotherapy	0.1954	24.4% (0.0%; 69.3%)	5
Curcumin	0.5097	80.7% (61.0%; 90.5%)	8
Adjunctive Curcumin	0.5106	80.8% (61.1%; 90.5%)	7

Omega 3: Omega-3 polyunsaturated fatty acids;SJW: St. John's wort; SAMe: S-Adenosyl Methionine

16.Assessment of inconsistency results for each outcome

16.1. Summary result of Design by treatment model for each outcome

Outcome	Between study variance ($\tau 2$)	Heterogeneity assessment	Q	df	P value
**Depression**	0.2451	High	37.04	23	0.0322
**Response**	0.2526	High	30.37	13	0.0042
**Remission**	0.4827	High	20.47	6	0.0023
**Anxiety**	1.0504	High	0.14	3	0.9861
**All cause discontinuation**	0	Low	9.74	11	0.5538
**Adverse event**	0.1522	Moderate	11.71	6	0.0688

16.2 Evaluation of the inconsistency by Design by treatment model

- ✓ The following abbreviations are used in the tables throughout the documents: ADT: Antidepressant; Ca: Calcium ; DHA: Docosahexaenoic Acid ; E Amoenum : Echium amoenum; EPA :Eicosapentaenoic acid; Fe : Ferrum; Mg: Magnesium; PEA: Palmitoylethanolamide; R rosea : Rhodiola rosea; SAMe: S-Adenosyl Methionine ; SJW: St. John's wort ;Vitamin B1: Thiamine ; Vitamin B6: Pyridoxine; Vitamin B7: Biotin; Vitamin B: Vitamin B complex; Vitamin B12 : Cobalamin; Vitamin C: Ascorbic acid; Vitamin D: Cholecalciferol; 5HTP: 5-Hydroxytryptophan

16.2.A. Evaluation of the inconsistency by Design by treatment model for outcome as change in depressive symptoms

**Design-specific decomposition of within-designs Q statistic**			
**Design**	**Q**	**df**	**p-value**
Placebo:SJW	161.03	9	< 0.0001
ADT:EPA+DHA+ADT	112.70	10	< 0.0001
Placebo:EPA+DHA	100.85	14	< 0.0001
Placebo:Vitamin_D	75.66	5	< 0.0001
Placebo:ADT:SJW	60.53	14	< 0.0001
ADT:SJW	49.25	9	< 0.0001
ADT:Curcumin+ADT	25.72	4	< 0.0001
ADT:Vitamin_D+ADT	19.16	4	0.0007
ADT:Probiotics+ADT	24.40	7	0.0010
ADT:Folate+ADT	20.94	7	0.0039
Placebo:EPA	10.76	2	0.0046
ADT:SAMe	12.82	3	0.0050
ADT:EPA+ADT	10.40	2	0.0055
ADT:SAMe+ADT	10.07	2	0.0065
ADT:Ginkgo+ADT	5.62	1	0.0178
Placebo:Saffron	13.63	5	0.0181
ADT:Creatine+ADT	5.44	1	0.0197
Placebo:Probiotics	8.08	3	0.0443
ADT:Prebiotics+ADT	3.52	1	0.0607
ADT:Saffron	10.51	6	0.1047
Placebo:SAMe	5.29	4	0.2590
ADT:Tryptophan+ADT	0.66	1	0.4152
ADT:Zinc+ADT	0.64	1	0.4238
ADT:Inositol+ADT	0.13	1	0.7214
Placebo:ADT:SAMe	1.09	4	0.8962
Placebo:SAMe	4.69	10	0.9108
**Between-designs Q statistic after detaching of single designs**			

**Detached design**	**Q**	**df**	**p-value**
Placebo:ADT:SJW	82.71	21	< 0.0001
Placebo:SJW	115.29	22	< 0.0001
Placebo:R_rosea	165.83	22	< 0.0001
ADT:Saffron	167.38	22	< 0.0001
Placebo:Saffron	167.38	22	< 0.0001
Placebo:ADT:R_rosea	165.82	21	< 0.0001
ADT:EPA+ADT	169.39	22	< 0.0001
ADT:DHA+ADT:EPA+ADT	169.36	21	< 0.0001
Placebo:EPA	181.66	22	< 0.0001
ADT:Curcumin+ADT	181.92	22	< 0.0001
ADT:SJW	182.14	22	< 0.0001
Placebo:EPA+DHA	183.07	22	< 0.0001
Placebo:DHA:EPA	180.85	21	< 0.0001
Placebo:ADT:EPA+DHA:EPA+DHA+ADT	178.55	20	< 0.0001
ADT:DHA+ADT	184.41	22	< 0.0001
ADT:Carnitine	184.60	22	< 0.0001
Placebo:Carnitine	184.60	22	< 0.0001
Placebo:DHA	185.53	22	< 0.0001
Placebo:SAMe	185.65	22	< 0.0001
Placebo:Vitamin_D	185.96	22	< 0.0001
ADT:SAMe	186.70	22	< 0.0001
ADT:EPA+DHA+ADT	186.79	22	< 0.0001
Placebo:Curcumin	186.82	22	< 0.0001
ADT:Mg	186.86	22	< 0.0001
Placebo:Mg	186.86	22	< 0.0001
ADT:Curcumin:Curcumin+ADT	185.24	21	< 0.0001
Placebo:ADT:SAMe	185.66	21	< 0.0001
Q statistic to assess consistency under the assumption of a full design-by-treatment interaction random effects model: Between designs,37.04 ; degree of freedom,23; p value, 0.0322 ; tau.within, 0.4951 , tau2..within, 0.2451			

16.2.B. Evaluation of the inconsistency by Design by treatment model for outcome as response rate

Design-specific decomposition of within-designs Q statistic			
**Design**	**Q**	**df**	**p-value**
Placebo:SJW	49.46	12	< 0.0001
ADT:Folate+ADT	20.35	4	0.0004
ADT:SJW	19.17	9	0.0238
Placebo:ADT:SJW	19.68	10	0.0324
Placebo:Saffron	3.54	1	0.0598
ADT:SAMe	8.75	4	0.0676
ADT:SAMe+ADT	3.10	1	0.0783
Placebo:EPA	0.47	1	0.4947
ADT:Saffron	1.97	3	0.5785
Placebo:EPA+DHA	0.86	2	0.6495
Placebo:DHA	0.15	1	0.7024
Placebo:ADT:SAMe	0.69	2	0.7099
ADT:EPA+DHA+ADT	0.57	2	0.7509
**Between-designs Q statistic after detaching of single designs**			
**Detached design**	**Q**	**df**	**p-value**
Placebo:ADT:SJW	27.09	11	0.0045
Placebo:SJW	36.00	12	0.0003
Placebo:Saffron	38.84	12	0.0001
ADT:Saffron	38.84	12	0.0001
ADT:Curcumin+ADT	44.90	12	< 0.0001
ADT:EPA+ADT	45.44	12	< 0.0001
ADT:EPA+DHA	45.99	12	< 0.0001
ADT:EPA:EPA+ADT	45.39	11	< 0.0001
Placebo:EPA+DHA	48.02	12	< 0.0001
Placebo:SAMe	48.40	12	< 0.0001
Placebo:Creatine+ADT	49.53	12	< 0.0001
ADT:Creatine+ADT	49.53	12	< 0.0001
ADT:SJW	49.68	12	< 0.0001
ADT:SAMe	50.01	12	< 0.0001
Placebo:EPA	50.09	12	< 0.0001
ADT:EPA:EPA+DHA	48.37	11	< 0.0001
Placebo:ADT:SAMe	48.73	11	< 0.0001
Q statistic to assess consistency under the assumption of a full design-by-treatment interaction random effects model: Between designs,30.37 ; degree of freedom,13; p value, 0.0042 ; tau.within, 0.5026 , tau2..within, 0.2526			

16.2.C. Evaluation of the inconsistency by Design by treatment model as remission rate

Design-specific decomposition of within-designs Q statistic			
**Design**	**Q**	**df**	**p-value**
Placebo:SJW	28.06	6	< 0.0001
Placebo:ADT:SJW	18.96	6	0.0042
ADT:EPA+DHA+ADT	1.95	1	0.1627
Placebo:Saffron	1.80	1	0.1795
ADT:Folate+ADT	3.04	2	0.2190
ADT:Saffron	2.34	2	0.3099
Placebo:ADT:SAMe	1.21	2	0.5468
Placebo:EPA+DHA	0.03	1	0.8619
**Between-designs Q statistic after detaching of single designs**			
**Detached design**	**Q**	**df**	**p-value**
Placebo:ADT:SJW	20.86	4	0.0003
Placebo:Saffron	23.78	5	0.0002
ADT:Saffron	23.78	5	0.0002
ADT:Curcumin+ADT	25.37	5	0.0001
Placebo:SJW	30.97	5	< 0.0001
ADT:SJW	34.22	5	< 0.0001
Placebo:EPA	34.35	5	< 0.0001
Q statistic to assess consistency under the assumption of a full design-by-treatment interaction random effects model: Between designs, 20.47; degree of freedom,6; p value, 0.0023; tau.within, 0.6948 , tau2..within, 0.4827			

16.2.D. Evaluation of the inconsistency by Design by treatment model for outcome as change in anxiety sympomts

Design-specific decomposition of within-designs Q statistic			
**Design**	**Q**	**df**	**p-value**
Placebo:EPA+DHA	47.19	1	< 0.0001
ADT:Saffron	51.27	2	< 0.0001
ADT:Probiotics+ADT	41.15	1	< 0.0001
ADT:Curcumin+ADT	25.37	1	< 0.0001
ADT:EPA+DHA+ADT	0.99	1	0.3194
Placebo:SJW	0.82	1	0.3664
ADT:SAMe	0.00	1	0.9738
**Between-designs Q statistic after detaching of single designs**			
**Detached design**	**Q**	**df**	**p-value**
ADT:Curcumin+ADT	0.36	2	0.8356
ADT:Curcumin	4.45	2	0.1082
Placebo:Curcumin	4.45	2	0.1082
Placebo:SJW	4.71	2	0.0949
Placebo:ADT:SJW	4.43	1	0.0353
Q statistic to assess consistency under the assumption of a full design-by-treatment interaction random effects model: Between designs, 0.14; degree of freedom,3; p value, 0.9861 ; tau.within, 1.0249 , tau2..within, 1.0504			

16.2.E. Evaluation of the inconsistency by Design by treatment model for outcome as all cause discontinuation

Design-specific decomposition of within-designs Q statistic			
**Design**	**Q**	**df**	**p-value**
ADT:EPA+DHA+ADT	5.86	1	0.0155
ADT:Folate+ADT	5.49	3	0.1394
ADT:Curcumin+ADT	2.76	3	0.4300
Placebo:SJW	5.30	6	0.5066
Placebo:DHA:EPA	1.22	2	0.5433
ADT:SAMe+ADT	0.21	1	0.6455
ADT:Inositol+ADT	0.86	2	0.6513
ADT:SJW	6.60	9	0.6783
Placebo:Probiotics	1.34	3	0.7185
Placebo:Saffron	0.77	3	0.8575
ADT:Vitamin_D+ADT	0.01	1	0.9166
Placebo:Vitamin_D	0.01	1	0.9215
ADT:SAMe	0.00	1	0.9905
Placebo:ADT:SJW	3.44	14	0.9980
ADT:Probiotics+ADT	0.00	2	0.9986
ADT:Saffron	0.00	4	1.0000
**Between-designs Q statistic after detaching of single designs**			
**Detached design**	**Q**	**df**	**p-value**
Placebo:SAMe	4.69	10	0.9108
Placebo:Mg	8.53	10	0.5768
ADT:Mg	8.53	10	0.5768
ADT:SJW	8.56	10	0.5745
Placebo:ADT:SJW	7.64	9	0.5711
ADT:Saffron	9.20	10	0.5137
Placebo:Saffron	9.20	10	0.5137
ADT:SAMe	9.63	10	0.4732
Placebo:SJW	9.66	10	0.4706
ADT:Curcumin+ADT	9.69	10	0.4684
Placebo:Curcumin	9.73	10	0.4647
Placebo:Vitamin_D	9.74	10	0.4638
Placebo:ADT:SAMe	9.28	9	0.4119
ADT:Curcumin:Curcumin+ADT	9.64	9	0.3801

Q statistic to assess consistency under the assumption of a full design-by-treatment interaction random effects model: Between designs,9.74 ; degree of freedom,11; p value, 0.5538 ; tau.within, 0 , tau2..within, 0

16.2.F. Evaluation of the inconsistency by Design by treatment model for outcome as adverse event

Design-specific decomposition of within-designs Q statistic			
**Design**	**Q**	**df**	**p-value**
ADT:EPA+DHA+ADT	3.39	2	0.1838
ADT:SAMe	7.79	2	0.0204
ADT:SJW	18.33	10	0.0496
Placebo:SJW	12.41	6	0.0535
Placebo:ADT:SJW	6.01	4	0.1988
**Between-designs Q statistic after detaching of single designs**			
**Detached design**	**Q**	**df**	**p-value**
ADT:SAMe	7.62	5	0.1787
ADT:SJW	15.32	5	0.0091
Placebo:SAMe	13.37	5	0.0201
Placebo:SJW	13.01	5	0.0233
Placebo:ADT:SAMe	7.34	4	0.1188
Placebo:ADT:SJW	13.45	4	0.0093
Q statistic to assess consistency under the assumption of a full design-by-treatment interaction random effects model: Between designs,11.76 ; degree of freedom,6; p value, 0.0688 ; tau.within, 0.3592 , tau2..within, 0.1290			

16.3. Evaluation of the inconsistency by node split model

- ✓ The following abbreviations are used in the tables throughout the documents: ADT: Antidepressant; Ca: Calcium ; DHA: Docosahexaenoic Acid ; E Amoenum : Echium amoenum; EPA :Eicosapentaenoic acid; Fe : Ferrum; Mg: Magnesium; PEA: Palmitoylethanolamide; R rosea : Rhodiola rosea; SAMe: S-Adenosyl Methionine ; SJW: St. John's wort ;Vitamin B1: Thiamine ; Vitamin B6: Pyridoxine; Vitamin B7: Biotin; Vitamin B: Vitamin B complex; Vitamin B12 : Cobalamin; Vitamin C: Ascorbic acid; Vitamin D: Cholecalciferol; 5HTP: 5-Hydroxytryptophan

16.3.A. Evaluation of the inconsistency by node split model for outcome as change in depressive symptom

Comparison	k	prop	nma	direct	indir.	Diff	z	p-value
ADT:Amino_acid+ADT	1	1	-1.587	-1.587	.	.		
ADT:Carnitine	1	0.59	-0.5968	-0.3465	-0.9587	0.6123	0.69	0.4883
ADT:Carnitine+ADT	1	1	-1.3514	-1.3514	.	.		
ADT:Chlorella+ADT	1	1	-0.4761	-0.4761	.	.		
ADT:Creatine+ADT	2	1	-0.6084	-0.6084	.	.		
ADT:Curcumin	1	0.41	-0.1264	0.0526	-0.2502	0.3027	0.37	0.7092
ADT:Curcumin+ADT	7	0.98	-0.5374	-0.5377	-0.5226	-0.0151	-0.01	0.9924
ADT:Curcumin+Saffron+ADT	1	0.79	-0.2602	-0.1127	-0.8049	0.6922	0.55	0.5806
ADT:DHA	0	0	0.4863	.	0.4863	.	.	
ADT:DHA+ADT	2	0.91	0.1473	-0.0679	2.2789	-2.3468	-1.72	0.0855
ADT:E_amoenum	0	0	-0.2154	.	-0.2154	.	.	
ADT:EPA	0	0	0.4204	.	0.4204	.	.	
ADT:EPA+ADT	4	0.96	-0.5424	-0.5069	-1.4835	0.9766	0.6	0.5509
ADT:EPA+DHA	1	0.06	-0.1084	-1.5931	-0.0202	-1.5729	-1.99	0.0463
ADT:EPA+DHA+ADT	12	0.97	-0.5463	-0.5964	1.3564	-1.9528	-1.79	0.0742
ADT:EPA+DHA+Vitamin_C	0	0	0.3798	.	0.3798	.	.	
ADT:Fe	0	0	-0.4543	.	-0.4543	.	.	
ADT:Folate+ADT	8	1	-0.1486	-0.1486	.	.		
ADT:Folate+Vitamin_B12+Vitamin_B6	0	0	0.3109	.	0.3109	.	.	
ADT:Ginkgo+ADT	2	1	-0.758	-0.758	.	.		
ADT:Inositol+ADT	2	1	-0.0527	-0.0527	.	.		
ADT: L-arginine+ADT	1	1	-0.3613	-0.3613	.	.		
ADT:L-theanine+ADT	1	1	-3.1452	-3.1452	.	.		
ADT:Lavandula	1	1	0.3277	0.3277	.	.		
ADT:Lavandula+ADT	1	1	-0.4829	-0.4829	.	.		
ADT:Mg	1	0.45	-0.2129	-0.3155	-0.1298	-0.1857	-0.21	0.8361
ADT:Mg+ADT	1	1	-0.4006	-0.4006	.	.		

ADT:Nepta	1	1	-0.836	-0.836	.	.		
ADT:PEA+ADT	1	1	-0.5617	-0.5617	.	.		
ADT:Placebo	13	0.51	0.491	0.1312	0.8663	-0.7351	-3.22	0.0013
ADT:Prebiotics+ADT	2	1	-0.1696	-0.1696	.	.		
ADT:Probiotics	0	0	0.1384	.	0.1384	.	.	
ADT:Probiotics+ADT	8	1	-0.673	-0.673	.	.		
ADT:Probiotics+B7	0	0	0.6038	.	0.6038	.	.	
ADT:Probiotics+Mg+CoQ10+ADT	1	1	-0.3749	-0.3749	.	.		
ADT:R_rosea	1	0.44	-0.4153	0.3403	-1.0179	1.3582	1.66	0.0967
ADT:Saffron	7	0.6	-0.201	0.1119	-0.6624	0.7743	2.18	0.0293
ADT:Saffron+ADT	1	1	-3.3016	-3.3016	.	.		
ADT:SAMe	7	0.64	-0.0329	-0.1153	0.1121	-0.2274	-0.62	0.5373
ADT:SAMe+ADT	3	1	-0.5009	-0.5009	.	.		
ADT:SAMe+EPA+DHA+Folate+5HTP+Zinc+ADT	1	1	0.2134	0.2134	.	.		
ADT:SAMe+Probiotcis	0	0	0.0064	.	0.0064	.	.	
ADT:SAMe+Vitamin_B12+Folate+ADT	1	1	0.0962	0.0962	.	.		
ADT:SJW	18	0.78	0.0327	0.1433	-0.3502	0.4935	1.79	0.074
ADT:SJW+ADT	1	1	-0.0958	-0.0958	.	.		
ADT:SJW+Kava	0	0	0.1142	.	0.1142	.	.	
ADT:Tryptophan+ADT	2	1	-0.7484	-0.7484	.	.		
ADT:Vitamin_B+ADT	1	1	0.0395	0.0395	.	.		
ADT:Vitamin_B1+ADT	1	1	-0.1908	-0.1908	.	.		
ADT:Vitamin_B12+ADT	1	1	-1.529	-1.529	.	.		
ADT:Vitamin_B6+Tryptophan	1	1	1.3156	1.3156	.	.		
ADT:Vitamin_C	0	0	0.3538	.	0.3538	.	.	
ADT:Vitamin_C+ADT	1	1	0.0585	0.0585	.	.		
ADT:Vitamin_D	0	0	-0.2167	.	-0.2167	.	.	
ADT:Vitamin_D+ADT	5	1	-0.4535	-0.4535	.	.		
ADT:Vitamin_D+Ca	0	0	0.2552	.	0.2552	.	.	
ADT:Zinc	0	0	-0.6812	.	-0.6812	.	.	
ADT:Zinc+ADT	2	1	-1.1019	-1.1019	.	.		
ADT:Zinc+Vitamin_D	0	0	-0.7853	.	-0.7853	.	.	
Amino_acid+ADT:Carnitine	0	0	0.9902	.	0.9902	.	.	
Amino_acid+ADT:Carnitine+ADT	0	0	0.2355	.	0.2355	.	.	
Amino_acid+ADT:Chlorella+ADT	0	0	1.1109	.	1.1109	.	.	

Amino_acid+ADT:Creatine+ADT	0	0	0.9786	.	0.9786	.	.	
Amino_acid+ADT:Curcumin	0	0	1.4606	.	1.4606	.	.	
Amino_acid+ADT:Curcumin+ADT	0	0	1.0495	.	1.0495	.	.	
Amino_acid+ADT:Curcumin+Saffron+ADT	0	0	1.3267	.	1.3267	.	.	
Amino_acid+ADT:DHA	0	0	2.0732	.	2.0732	.	.	
Amino_acid+ADT:DHA+ADT	0	0	1.7343	.	1.7343	.	.	
Amino_acid+ADT:E_amoenum	0	0	1.3716	.	1.3716	.	.	
Amino_acid+ADT:EPA	0	0	2.0073	.	2.0073	.	.	
Amino_acid+ADT:EPA+ADT	0	0	1.0445	.	1.0445	.	.	
Amino_acid+ADT:EPA+DHA	0	0	1.4786	.	1.4786	.	.	
Amino_acid+ADT:EPA+DHA+ADT	0	0	1.0407	.	1.0407	.	.	
Amino_acid+ADT:EPA+DHA+Vitamin_C	0	0	1.9667	.	1.9667	.	.	
Amino_acid+ADT:Fe	0	0	1.1327	.	1.1327	.	.	
Amino_acid+ADT:Folate+ADT	0	0	1.4384	.	1.4384	.	.	
Amino_acid+ADT:Folate+Vitamin_B12+Vitamin_B6	0	0	1.8979	.	1.8979	.	.	
Amino_acid+ADT:Ginkgo+ADT	0	0	0.8289	.	0.8289	.	.	
Amino_acid+ADT:Inositol+ADT	0	0	1.5342	.	1.5342	.	.	
Amino_acid+ADT:l-arginine+ADT	0	0	1.2256	.	1.2256	.	.	
Amino_acid+ADT:L-theanine+ADT	0	0	-1.5582	.	-1.5582	.	.	
Amino_acid+ADT:Lavandula	0	0	1.9146	.	1.9146	.	.	
Amino_acid+ADT:Lavandula+ADT	0	0	1.104	.	1.104	.	.	
Amino_acid+ADT:Mg	0	0	1.374	.	1.374	.	.	
Amino_acid+ADT:Mg+ADT	0	0	1.1864	.	1.1864	.	.	
Amino_acid+ADT:Nepta	0	0	0.7509	.	0.7509	.	.	
Amino_acid+ADT:PEA+ADT	0	0	1.0253	.	1.0253	.	.	
Amino_acid+ADT:Placebo	0	0	2.078	.	2.078	.	.	
Amino_acid+ADT:Prebiotics+ADT	0	0	1.4174	.	1.4174	.	.	
Amino_acid+ADT:Probiotics	0	0	1.7253	.	1.7253	.	.	
Amino_acid+ADT:Probiotics+ADT	0	0	0.914	.	0.914	.	.	
Amino_acid+ADT:Probiotics+B7	0	0	2.1907	.	2.1907	.	.	
Amino_acid+ADT:Probiotics+Mg+CoQ10+ADT	0	0	1.2121	.	1.2121	.	.	
Amino_acid+ADT:R_rosea	0	0	1.1716	.	1.1716	.	.	
Amino_acid+ADT:Saffron	0	0	1.3859	.	1.3859	.	.	
Amino_acid+ADT:Saffron+ADT	0	0	-1.7147	.	-1.7147	.	.	
Amino_acid+ADT:SAMe	0	0	1.5541	.	1.5541	.	.	

Amino_acid+ADT:SAMe+ADT	0	0	1.086	.	1.086	.	.	
Amino_acid+ADT:SAMe+EPA+DHA+Folate+5HTP+Zinc+ADT	0	0	1.8004	.	1.8004	.	.	
Amino_acid+ADT:SAMe+Probiotcis	0	0	1.5933	.	1.5933	.	.	
Amino_acid+ADT:SAMe+Vitamin_B12+Folate+ADT	0	0	1.6831	.	1.6831	.	.	
Amino_acid+ADT:SJW	0	0	1.6196	.	1.6196	.	.	
Amino_acid+ADT:SJW+ADT	0	0	1.4911	.	1.4911	.	.	
Amino_acid+ADT:SJW+Kava	0	0	1.7011	.	1.7011	.	.	
Amino_acid+ADT:Tryptophan+ADT	0	0	0.8385	.	0.8385	.	.	
Amino_acid+ADT:Vitamin_B+ADT	0	0	1.6264	.	1.6264	.	.	
Amino_acid+ADT:Vitamin_B1+ADT	0	0	1.3962	.	1.3962	.	.	
Amino_acid+ADT:Vitamin_B12+ADT	0	0	0.0579	.	0.0579	.	.	
Amino_acid+ADT:Vitamin_B6+Tryptophan	0	0	2.9025	.	2.9025	.	.	
Amino_acid+ADT:Vitamin_C	0	0	1.9407	.	1.9407	.	.	
Amino_acid+ADT:Vitamin_C+ADT	0	0	1.6455	.	1.6455	.	.	
Amino_acid+ADT:Vitamin_C	0	0	1.9407	.	1.9407	.	.	
Amino_acid+ADT:Vitamin_C+ADT	0	0	1.6455	.	1.6455	.	.	

16.3.B. Evaluation of the inconsistency by node split model for outcome as response rate

Comparison	k	prop	nma	direct	indir.	Diff	z	p-value
ADT:Amino_acid+ADT	1	1	0.2899	0.2899	.	.		
ADT:Carnitine+ADT	1	1	0.0476	0.0476	.	.		
ADT:Chromium	0	0	1.8061	.	1.8061	.	.	
ADT:Creatine+ADT	1	0.34	2.4864	4	1.9463	2.0552	0.51	0.6097
ADT:Curcumin	1	0.87	0.6133	1.1	0.0128	85.6799	1.76	0.0783
ADT:Curcumin+ADT	2	1	0.1488	0.1488	.	.		
ADT:DHA	0	0	2.0522	.	2.0522	.	.	
ADT:EPA	2	0.53	1.0444	0.6756	1.6945	0.3987	-1.08	0.2802
ADT:EPA+ADT	2	0.94	0.7905	0.827	0.3837	2.1557	0.33	0.7416
ADT:EPA+DHA	2	0.54	0.5601	0.6643	0.4584	1.4493	0.48	0.6347
ADT:EPA+DHA+ADT	3	1	0.7092	0.7092	.	.		
ADT:Folate+ADT	5	1	0.6957	0.6957	.	.		
ADT:Folate+Vitamin_B12+Vitamin_B6	0	0	3.8312	.	3.8312	.	.	
ADT:Ginkgo+ADT	1	1	0.6886	0.6886	.	.		
ADT:Inositol+ADT	1	1	1.1667	1.1667	.	.		
ADT:L -theanine+ADT	1	1	1	1	.	.		
ADT:PEA+ADT	1	1	0.0497	0.0497	.	.		
ADT:Placebo	8	0.47	2.0577	1.1253	3.5065	0.3209	-3.29	0.001
ADT:Probiotics	0	0	2.444	.	2.444	.	.	
ADT:Saffron	4	0.69	0.8509	1.6147	0.2094	7.713	2.54	0.011
ADT:SAMe	7	0.89	0.7441	0.7097	1.0789	0.6578	-0.46	0.6427
ADT:SAMe+ADT	2	1	0.47	0.47	.	.		
ADT:SAMe+EPA+DHA+Folate+5HTP+Zinc+ADT	1	1	1.6006	1.6006	.	.		
ADT:SAMe+Vitamin_B12+Folate+ADT	1	1	0.8075	0.8075	.	.		
ADT:SJW	16	0.82	0.9388	1.0367	0.5954	1.7412	1.38	0.1689
ADT:Tryptophan+ADT	1	1	0.3117	0.3117	.	.		
ADT:Vit_C+ADT	1	1	1.0625	1.0625	.	.		
ADT:Vitamin_B1+ADT	1	1	0.1167	0.1167	.	.		
ADT:Vitamin_B12+ADT	1	1	0.0685	0.0685	.	.		
ADT:Vitamin_D	0	0	1.7637	.	1.7637	.	.	
ADT:Vitamin_D+ADT	1	1	0.875	0.875	.	.		
ADT:Zinc+ADT	1	1	0.404	0.404	.	.		
Amino_acid+ADT:Carnitine+ADT	0	0	0.1642	.	0.1642	.	.	
Amino_acid+ADT:Chromium	0	0	6.229	.	6.229	.	.	
Amino_acid+ADT:Creatine+ADT	0	0	8.5754	.	8.5754	.	.	
Amino_acid+ADT:Curcumin	0	0	2.1153	.	2.1153	.	.	
Amino_acid+ADT:Curcumin+ADT	0	0	0.5133	.	0.5133	.	.	
Amino_acid+ADT:DHA	0	0	7.0779	.	7.0779	.	.	
Amino_acid+ADT:EPA	0	0	3.6022	.	3.6022	.	.	

Amino_acid+ADT:EPA+ADT	0	0	2.7265	.	2.7265	.	.	
Amino_acid+ADT:EPA+DHA	0	0	1.9316	.	1.9316	.	.	
Amino_acid+ADT:EPA+DHA+ADT	0	0	2.446	.	2.446	.	.	
Amino_acid+ADT:Folate+ADT	0	0	2.3994	.	2.3994	.	.	
Amino_acid+ADT:Folate+Vitamin_B12+Vitamin_B6	0	0	13.2136	.	13.2136	.	.	
Amino_acid+ADT:Ginkgo+ADT	0	0	2.3751	.	2.3751	.	.	
Amino_acid+ADT:Inositol+ADT	0	0	4.0238	.	4.0238	.	.	
Amino_acid+ADT:L -theanine+ADT	0	0	3.449	.	3.449	.	.	
Amino_acid+ADT:PEA+ADT	0	0	0.1714	.	0.1714	.	.	
Amino_acid+ADT:Placebo	0	0	7.0969	.	7.0969	.	.	
Amino_acid+ADT:Probiotics	0	0	8.4292	.	8.4292	.	.	
Amino_acid+ADT:Saffron	0	0	2.9349	.	2.9349	.	.	
Amino_acid+ADT:SAMe	0	0	2.5664	.	2.5664	.	.	
Amino_acid+ADT:SAMe+ADT	0	0	1.6211	.	1.6211	.	.	
Amino_acid+ADT:SAMe+EPA+DHA+Folate+5HTP+Zinc+ADT	0	0	5.5206	.	5.5206	.	.	
Amino_acid+ADT:SAMe+Vitamin_B12+Folate+ADT	0	0	2.7851	.	2.7851	.	.	
Amino_acid+ADT:SJW	0	0	3.238	.	3.238	.	.	
Amino_acid+ADT:Tryptophan+ADT	0	0	1.075	.	1.075	.	.	
Amino_acid+ADT:Vit_C+ADT	0	0	3.6645	.	3.6645	.	.	
Amino_acid+ADT:Vitamin_B1+ADT	0	0	0.4024	.	0.4024	.	.	
Amino_acid+ADT:Vitamin_B12+ADT	0	0	0.2361	.	0.2361	.	.	
Amino_acid+ADT:Vitamin_D	0	0	6.083	.	6.083	.	.	
Amino_acid+ADT:Vitamin_D+ADT	0	0	3.0179	.	3.0179	.	.	
Amino_acid+ADT:Zinc+ADT	0	0	1.3935	.	1.3935	.	.	
Carnitine+ADT:Chromium	0	0	37.9272	.	37.9272	.	.	
Carnitine+ADT:Creatine+ADT	0	0	52.2135	.	52.2135	.	.	
Carnitine+ADT:Curcumin	0	0	12.8797	.	12.8797	.	.	
Carnitine+ADT:Curcumin+ADT	0	0	3.1255	.	3.1255	.	.	
Carnitine+ADT:DHA	0	0	43.0955	.	43.0955	.	.	
Carnitine+ADT:EPA	0	0	21.9328	.	21.9328	.	.	
Carnitine+ADT:EPA+ADT	0	0	16.6008	.	16.6008	.	.	
Carnitine+ADT:EPA+DHA	0	0	11.7613	.	11.7613	.	.	
Carnitine+ADT:EPA+DHA+ADT	0	0	14.8931	.	14.8931	.	.	
Carnitine+ADT:Folate+ADT	0	0	14.6093	.	14.6093	.	.	
Carnitine+ADT:Folate+Vitamin_B12+Vitamin_B6	0	0	80.4545	.	80.4545	.	.	
Carnitine+ADT:Ginkgo+ADT	0	0	14.4615	.	14.4615	.	.	
Carnitine+ADT:Inositol+ADT	0	0	24.5	.	24.5	.	.	
Carnitine+ADT:L -theanine+ADT	0	0	21	.	21	.	.	
Carnitine+ADT:PEA+ADT	0	0	1.0436	.	1.0436	.	.	
Carnitine+ADT:Placebo	0	0	43.2111	.	43.2111	.	.	

Carnitine+ADT:Probiotics	0	0	51.3236	.	51.3236	.	.	
Carnitine+ADT:Saffron	0	0	17.8699	.	17.8699	.	.	
Carnitine+ADT:SAMe	0	0	15.6261	.	15.6261	.	.	
Carnitine+ADT:SAMe+ADT	0	0	9.8704	.	9.8704	.	.	
Carnitine+ADT:SAMe+EPA+DHA+Folate+5HTP +Zinc+ADT	0	0	33.6136	.	33.6136	.	.	
Carnitine+ADT:SAMe+Vitamin_B12+Folate+ADT	0	0	16.9575	.	16.9575	.	.	
Carnitine+ADT:SJW	0	0	19.7152	.	19.7152	.	.	
Carnitine+ADT:Tryptophan+ADT	0	0	6.5455	.	6.5455	.	.	
Carnitine+ADT:Vit_C+ADT	0	0	22.3125	.	22.3125	.	.	
Carnitine+ADT:Vitamin_B1+ADT	0	0	2.45	.	2.45	.	.	
Carnitine+ADT:Vitamin_B12+ADT	0	0	1.4378	.	1.4378	.	.	
Carnitine+ADT:Vitamin_D	0	0	37.0381	.	37.0381	.	.	
Carnitine+ADT:Vitamin_D+ADT	0	0	18.375	.	18.375	.	.	
Carnitine+ADT:Zinc+ADT	0	0	8.4848	.	8.4848	.	.	
Chromium:Creatine+ADT	0	0	1.3767	.	1.3767	.	.	
Chromium:Curcumin	0	0	0.3396	.	0.3396	.	.	
Chromium:Curcumin+ADT	0	0	0.0824	.	0.0824	.	.	
Chromium:DHA	0	0	1.1363	.	1.1363	.	.	
Chromium:EPA	0	0	0.5783	.	0.5783	.	.	
Chromium:EPA+ADT	0	0	0.4377	.	0.4377	.	.	
Chromium:EPA+DHA	0	0	0.3101	.	0.3101	.	.	
Chromium:EPA+DHA+ADT	0	0	0.3927	.	0.3927	.	.	
Chromium:Folate+ADT	0	0	0.3852	.	0.3852	.	.	
Chromium:Folate+Vitamin_B12+Vitamin_B6	0	0	2.1213	.	2.1213	.	.	
Chromium:Ginkgo+ADT	0	0	0.3813	.	0.3813	.	.	
Chromium:Inositol+ADT	0	0	0.646	.	0.646	.	.	
Chromium:L -theanine+ADT	0	0	0.5537	.	0.5537	.	.	
Chromium:PEA+ADT	0	0	0.0275	.	0.0275	.	.	
Chromium:Placebo	1	1	1.1393	1.1393	.	.		
Chromium:Probiotics	0	0	1.3532	.	1.3532	.	.	
Chromium:Saffron	0	0	0.4712	.	0.4712	.	.	
Chromium:SAMe	0	0	0.412	.	0.412	.	.	

16.3.C. Evaluation of the inconsistency by node split model for outcome as remission rate

Comparison	k	prop	nma	direct	indir.	Diff	z	p-value
ADT:Amino_acid+ADT	1	1	0.2899	0.2899	.	.		
ADT:Carnitine+ADT	1	1	0.5294	0.5294	.	.		
ADT:Creatine+ADT	1	1	0.3231	0.3231	.	.		
ADT:Curcumin	1	0.93	0.8971	1.875	0.0001	33590.75	2.58	0.0099
ADT:Curcumin+ADT	2	1	0.2091	0.2091	.	.		
ADT:DHA	0	0	2.6215	.	2.6215	.	.	
ADT:EPA	0	0	1.6686	.	1.6686	.	.	
ADT:EPA+ADT	1	1	1.1835	1.1835	.	.		
ADT:EPA+DHA	0	0	0.7939	.	0.7939	.	.	
ADT:EPA+DHA+ADT	2	1	0.5529	0.5529	.	.		
ADT:Folate+ADT	3	1	0.8485	0.8485	.	.		
ADT:Folate+Vitamin_B12+Vitamin_B6	0	0	2.3213	.	2.3213	.	.	
ADT:L -theanine+ADT	1	1	0.2215	0.2215	.	.		
ADT:PEA+ADT	1	1	0.4375	0.4375	.	.		
ADT:Placebo	6	0.69	2.5195	1.472	8.3959	0.1753	-2.35	0.0187
ADT:Saffron	3	0.69	0.7881	1.6626	0.1468	11.329	2.24	0.0251
ADT:SAMe	2	0.84	0.8466	0.8367	0.8993	0.9305	-0.04	0.9663
ADT:SAMe+ADT	1	1	0.1813	0.1812	.	.		
ADT:SAMe+EPA+DHA+Folate+5HTP+Zinc	1	1	1.5214	1.5214	.	.		
ADT:SAMe+Vitamin_B12+Folate+ADT	1	1	0.8203	0.8203	.	.		
ADT:SJW	5	0.71	0.829	0.9633	0.5689	1.6934	0.67	0.5053
ADT:Tryptophan+ADT	1	1	0.2	0.2	.	.		
ADT:Vitamin_B1+ADT	1	1	0.46	0.46	.	.		
ADT:Vitamin_C+ADT	1	1	1.1429	1.1429	.	.		
ADT:Vitamin_D+ADT	1	1	1.3	1.3	.	.		
ADT:Zinc+ADT	1	1	0.4615	0.4615	.	.		
Amino_acid+ADT:Carnitine+ADT	0	0	1.8259	.	1.8259	.	.	
Amino_acid+ADT:Creatine+ADT	0	0	1.1143	.	1.1143	.	.	
Amino_acid+ADT:Curcumin	0	0	3.0941	.	3.0941	.	.	
Amino_acid+ADT:Curcumin+ADT	0	0	0.721	.	0.721	.	.	
Amino_acid+ADT:DHA	0	0	9.0413	.	9.0413	.	.	
Amino_acid+ADT:EPA	0	0	5.755	.	5.755	.	.	
Amino_acid+ADT:EPA+ADT	0	0	4.0819	.	4.0819	.	.	

Amino_acid+ADT:EPA+DHA	0	0	2.738	.	2.738	.	.	
Amino_acid+ADT:EPA+DHA+ADT	0	0	1.9069	.	1.9069	.	.	
Amino_acid+ADT:Folate+ADT	0	0	2.9265	.	2.9265	.	.	
Amino_acid+ADT:Folate+Vitamin_B12+Vitamin_B6	0	0	8.0062	.	8.0062	.	.	
Amino_acid+ADT:L -theanine+ADT	0	0	0.7638	.	0.7638	.	.	
Amino_acid+ADT:PEA+ADT	0	0	1.5089	.	1.5089	.	.	
Amino_acid+ADT:Placebo	0	0	8.6898	.	8.6898	.	.	
Amino_acid+ADT:Saffron	0	0	2.718	.	2.718	.	.	
Amino_acid+ADT:SAMe	0	0	2.9201	.	2.9201	.	.	
Amino_acid+ADT:SAMe+ADT	0	0	0.6251	.	0.6251	.	.	
Amino_acid+ADT:SAMe+EPA+DHA+Folate+5HTP+Zinc	0	0	5.2472	.	5.2472	.	.	
Amino_acid+ADT:SAMe+Vitamin_B12+Folate+ADT	0	0	2.8292	.	2.8292	.	.	
Amino_acid+ADT:SJW	0	0	2.8591	.	2.8591	.	.	
Amino_acid+ADT:Tryptophan+ADT	0	0	0.6898	.	0.6898	.	.	
Amino_acid+ADT:Vitamin_B1+ADT	0	0	1.5865	.	1.5865	.	.	
Amino_acid+ADT:Vitamin_C+ADT	0	0	3.9417	.	3.9417	.	.	
Amino_acid+ADT:Vitamin_D+ADT	0	0	4.4837	.	4.4837	.	.	
Amino_acid+ADT:Zinc+ADT	0	0	1.5918	.	1.5918	.	.	
Carnitine+ADT:Creatine+ADT	0	0	0.6103	.	0.6103	.	.	
Carnitine+ADT:Curcumin	0	0	1.6945	.	1.6945	.	.	
Carnitine+ADT:Curcumin+ADT	0	0	0.3949	.	0.3949	.	.	
Carnitine+ADT:DHA	0	0	4.9516	.	4.9516	.	.	
Carnitine+ADT:EPA	0	0	3.1518	.	3.1518	.	.	
Carnitine+ADT:EPA+ADT	0	0	2.2355	.	2.2355	.	.	
Carnitine+ADT:EPA+DHA	0	0	1.4995	.	1.4995	.	.	
Carnitine+ADT:EPA+DHA+ADT	0	0	1.0443	.	1.0443	.	.	
Carnitine+ADT:Folate+ADT	0	0	1.6028	.	1.6028	.	.	
Carnitine+ADT:Folate+Vitamin_B12+Vitamin_B6	0	0	4.3847	.	4.3847	.	.	
Carnitine+ADT:L -theanine+ADT	0	0	0.4183	.	0.4183	.	.	
Carnitine+ADT:PEA+ADT	0	0	0.8264	.	0.8264	.	.	
Carnitine+ADT:Placebo	0	0	4.7591	.	4.7591	.	.	
Carnitine+ADT:Saffron	0	0	1.4886	.	1.4886	.	.	
Carnitine+ADT:SAMe	0	0	1.5992	.	1.5992	.	.	
Carnitine+ADT:SAMe+ADT	0	0	0.3424	.	0.3424	.	.	
Carnitine+ADT:SAMe+EPA+DHA+Folate+5HTP+Zinc	0	0	2.8737	.	2.8737	.	.	

Carnitine+ADT:SAMe+Vitamin_B12+Folate+ADT	0	0	1.5495	.	1.5495	.	.	
Carnitine+ADT:SJW	0	0	1.5658	.	1.5658	.	.	
Carnitine+ADT:Tryptophan+ADT	0	0	0.3778	.	0.3778	.	.	
Carnitine+ADT:Vitamin_B1+ADT	0	0	0.8689	.	0.8689	.	.	
Carnitine+ADT:Vitamin_C+ADT	0	0	2.1587	.	2.1587	.	.	
Carnitine+ADT:Vitamin_D+ADT	0	0	2.4556	.	2.4556	.	.	
Carnitine+ADT:Zinc+ADT	0	0	0.8718	.	0.8718	.	.	
Creatine+ADT:Curcumin	0	0	2.7768	.	2.7768	.	.	
Creatine+ADT:Curcumin+ADT	0	0	0.6471	.	0.6471	.	.	
Creatine+ADT:DHA	0	0	8.114	.	8.114	.	.	
Creatine+ADT:EPA	0	0	5.1647	.	5.1647	.	.	
Creatine+ADT:EPA+ADT	0	0	3.6632	.	3.6632	.	.	
Creatine+ADT:EPA+DHA	0	0	2.4572	.	2.4572	.	.	
Creatine+ADT:EPA+DHA+ADT	0	0	1.7113	.	1.7113	.	.	
Creatine+ADT:Folate+ADT	0	0	2.6264	.	2.6264	.	.	
Creatine+ADT:Folate+Vitamin_B12+Vitamin_B6	0	0	7.1851	.	7.1851	.	.	
Creatine+ADT:L -theanine+ADT	0	0	0.6855	.	0.6855	.	.	
Creatine+ADT:PEA+ADT	0	0	1.3542	.	1.3542	.	.	
Creatine+ADT:Placebo	0	0	7.7986	.	7.7986	.	.	
Creatine+ADT:Saffron	0	0	2.4392	.	2.4392	.	.	
Creatine+ADT:SAMe	0	0	2.6206	.	2.6206	.	.	
Creatine+ADT:SAMe+ADT	0	0	0.561	.	0.561	.	.	
Creatine+ADT:SAMe+EPA+DHA+Folate+5HTP+ Zinc	0	0	4.709	.	4.709	.	.	
Creatine+ADT:SAMe+Vitamin_B12+Folate+ADT	0	0	2.5391	.	2.5391	.	.	
Creatine+ADT:SJW	0	0	2.5659	.	2.5659	.	.	
Creatine+ADT:Tryptophan+ADT	0	0	0.619	.	0.619	.	.	
Creatine+ADT:Vitamin_B1+ADT	0	0	1.4238	.	1.4238	.	.	
Creatine+ADT:Vitamin_C+ADT	0	0	3.5374	.	3.5374	.	.	
Creatine+ADT:Vitamin_D+ADT	0	0	4.0238	.	4.0238	.	.	
Creatine+ADT:Zinc+ADT	0	0	1.4286	.	1.4286	.	.	
Curcumin:Curcumin+ADT	1	0.93	0.233	0.48	0	37413.0 8	2.58	0.0099
Curcumin:DHA	0	0	2.9221	.	2.9221	.	.	
Curcumin:EPA	0	0	1.86	.	1.86	.	.	
Curcumin:EPA+ADT	0	0	1.3192	.	1.3192	.	.	
Curcumin:EPA+DHA	0	0	0.8849	.	0.8849	.	.	

Curcumin:EPA+DHA+ADT	0	0	0.6163	.	0.6163	.	.	
Curcumin:Folate+ADT	0	0	0.9458	.	0.9458	.	.	
Curcumin:Folate+Vitamin_B12+Vitamin_B6	0	0	2.5876	.	2.5876	.	.	
Curcumin:L -theanine+ADT	0	0	0.2469	.	0.2469	.	.	
Curcumin:PEA+ADT	0	0	0.4877	.	0.4877	.	.	
Curcumin:Placebo	0	0	2.8085	.	2.8085	.	.	
Curcumin:Saffron	0	0	0.8784	.	0.8784	.	.	
Curcumin:SAMe	0	0	0.9437	.	0.9437	.	.	

16.3.D. Evaluation of the inconsistency by node split model for outcome as change in anxiety symptoms

Comparison	k	prop	nma	direct	indir.	Diff	z	p-value
ADT:Curcumin+ADT	3	1	-0.5931	-0.5931	.	.		
ADT:Placebo	1	0.82	0.1972	0.2062	0.1551	0.0511	0.03	0.9771
ADT:Saffron	3	1	-1.0345	-1.0345	.	.		
ADT:SJW	1	0.84	-0.0487	-0.0573	-0.0051	-0.0522	-0.03	0.9771
Curcumin+ADT:Placebo	0	0	0.7903	.	0.7903	.	.	
Curcumin+ADT:Saffron	0	0	-0.4414	.	-0.4414	.	.	
Curcumin+ADT:SJW	0	0	0.5444	.	0.5444	.	.	
Saffron:Placebo	0	0	1.2317	.	1.2317	.	.	
SJW:Placebo	3	1	0.2459	0.2459	.	.		
Saffron:SJW	0	0	0.9858	.	0.9858	.	.	

16.3.E. Evaluation of the inconsistency by node split model for outcome as all cause discontinuation

Comparison	k	prop	nma	direct	indir.	Diff	z	p-value
ADT:Carnitine	0	0	1.2474	.	1.2474	.	.	.
ADT:Chlorella+ADT	1	1	0.7051	0.705	.	.	.	.
ADT:Chromium	0	0	1.2485	.	1.2485	.	.	.
ADT:Creatine+ADT	1	1	0.5033	0.503	.	.	.	.
ADT:Curcumin	1	0.44	0.7053	0.608	0.7929	0.7673	-0.3	0.781
ADT:Curcumin+ADT	5	0.97	0.9349	0.929	1.1444	0.8115	-0.1	0.907
ADT:Curcumin+Saffron+ADT	0	0	0.8919	.	0.8919	.	.	.
ADT:DHA	0	0	1.4977	.	1.4977	.	.	.
ADT:DHA+ADT	1	1	0.6308	0.631	.	.	.	.
ADT:EPA	0	0	1.1686	.	1.1686	.	.	.
ADT:EPA+ADT	1	1	0.9724	0.972	.	.	.	.
ADT:EPA+DHA+ADT	1	1	6.269	6.269	.	.	.	.
ADT:Folate+ADT	4	1	1.1316	1.132	.	.	.	.
ADT:Inositol+ADT	4	1	0.8709	0.871	.	.	.	.
ADT:Mg	1	0.19	1.3381	6.579	0.9275	7.0933	1.1	0.272
ADT:Mg+ADT	1	1	12.4194	12.42	.	.	.	.
ADT:Nepta	1	1	1	1	.	.	.	.
ADT:PEA+ADT	1	1	1	1	.	.	.	.
ADT:Placebo	10	0.68	1.2474	1.183	1.3952	0.8482	-0.7	0.498
ADT:Probiotics	0	0	0.9888	.	0.9888	.	.	.
ADT:Probiotics+ADT	3	1	0.9181	0.918	.	.	.	.
ADT:Probiotics+B7	0	0	0.5489	.	0.5489	.	.	.
ADT:R_rosea	1	0.86	2.2532	3	0.3989	7.5199	1	0.308
ADT:Saffron	5	0.45	1.2707	1	1.5449	0.6473	-0.7	0.46
ADT:Saffron+ADT	1	1	1	1	.	.	.	.
ADT:SAMe	3	0.84	1.2951	1.449	0.7109	2.0381	1.2	0.25
ADT:SAMe+ADT	2	1	1.8892	1.889				
ADT:SAMe+EPA+DHA+Folic+5HTP+Zinc+ADT	1	1	0.7899	0.79				
ADT:SAMe+Vitamin_B12+Folate	1	1	0.6928	0.693				
ADT:SJW	18	0.89	1.3754	1.353	1.5865	0.8525	-0.5	0.618
ADT:Vitamin_C+ADT	1	1	1.6667	1.667				
ADT:Vitamin_D	0	0	3.5392		3.5392			
ADT:Vitamin_D+ADT	2	1	2.0667	2.067				
ADT:Zinc	0	0	1.9008		1.9008			
ADT:Zinc+ADT	1	1	0.4248	0.425				

ADT:Zinc+Vitamin_D	0	0	2.5214		2.5214			
Carnitine:Chlorella+ADT	0	0	0.5653		0.5653			
Carnitine:Chromium	0	0	1.0009		1.0009			
Carnitine:Creatine+ADT	0	0	0.4035		0.4035			
Carnitine:Curcumin	0	0	0.5654		0.5654			
Carnitine:Curcumin+ADT	0	0	0.7495	.	0.7495	.	.	.
Carnitine:Curcumin+Saffron+ADT	0	0	0.715	.	0.715	.	.	.
Carnitine:DHA	0	0	1.2007	.	1.2007	.	.	.
Carnitine:DHA+ADT	0	0	0.5057	.	0.5057	.	.	.
Carnitine:EPA	0	0	0.9368	.	0.9368	.	.	.
Carnitine:EPA+ADT	0	0	0.7796	.	0.7796	.	.	.
Carnitine:EPA+DHA+ADT	0	0	5.0258	.	5.0258	.	.	.
Carnitine:Folate+ADT	0	0	0.9072	.	0.9072	.	.	.
Carnitine:Inositol+ADT	0	0	0.6982	.	0.6982	.	.	.
Carnitine:Mg	0	0	1.0727	.	1.0727	.	.	.
Carnitine:Mg+ADT	0	0	9.9565	.	9.9565	.	.	.
Carnitine:Nepta	0	0	0.8017	.	0.8017	.	.	.
Carnitine:PEA+ADT	0	0	0.8017	.	0.8017	.	.	.
Carnitine:Placebo	1	1	1	1	.	.	.	.
Carnitine:Curcumin+ADT	0	0	0.7495	.	0.7495	.	.	.
Carnitine:Curcumin+Saffron+ADT	0	0	0.715	.	0.715	.	.	.
Carnitine:DHA	0	0	1.2007	.	1.2007	.	.	.
Carnitine:DHA+ADT	0	0	0.5057	.	0.5057	.	.	.
Carnitine:EPA	0	0	0.9368	.	0.9368	.	.	.
Carnitine:EPA+ADT	0	0	0.7796	.	0.7796	.	.	.
Carnitine:EPA+DHA+ADT	0	0	5.0258	.	5.0258	.	.	.
Carnitine:Folate+ADT	0	0	0.9072	.	0.9072	.	.	.
Carnitine:Inositol+ADT	0	0	0.6982	.	0.6982	.	.	.
Carnitine:Mg	0	0	1.0727	.	1.0727	.	.	.
Carnitine:Mg+ADT	0	0	9.9565	.	9.9565	.	.	.
Carnitine:Nepta	0	0	0.8017	.	0.8017	.	.	.
Carnitine:PEA+ADT	0	0	0.8017	.	0.8017	.	.	.
Carnitine:Placebo	1	1	1	1	.	.	.	.
Carnitine:Probiotics	0	0	0.7927	.	0.7927	.	.	.
Carnitine:Probiotics+ADT	0	0	0.736	.	0.736	.	.	.
Carnitine:Probiotics+B7	0	0	0.44	.	0.44	.	.	.
Carnitine:R_rosea	0	0	1.8064	.	1.8064	.	.	.
Carnitine:Saffron	0	0	1.0187	.	1.0187	.	.	.
Carnitine:Saffron+ADT	0	0	0.8017	.	0.8017	.	.	.

Carnitine:SAMe	0	0	1.0383	.	1.0383	.	.	.
Carnitine:SAMe+ADT	0	0	1.5146	.	1.5146	.	.	.
Carnitine:SAMe+EPA+DHA+Folic+5HTP+Zinc+ ADT	0	0	0.6333	.	0.6333	.	.	.
Carnitine:SAMe+Vitamin_B12+Folate	0	0	0.5554	.	0.5554	.	.	.
Carnitine:SJW	0	0	1.1027	.	1.1027	.	.	.
Carnitine:Vitamin_C+ADT	0	0	1.3362	.	1.3362	.		
Carnitine:Vitamin_D	0	0	2.8373	.	2.8373	.	.	.
Carnitine:Vitamin_D+ADT	0	0	1.6569	.	1.6569	.	.	.
Carnitine:Zinc	0	0	1.5238	.	1.5238	.	.	.
Carnitine:Zinc+ADT	0	0	0.3406	.	0.3406	.	.	.
Carnitine:Zinc+Vitamin_D	0	0	2.0214	.	2.0214	.	.	.
Chlorella+ADT:Chromium	0	0	1.7707	.	1.7707	.	.	.
Chlorella+ADT:Creatine+ADT	0	0	0.7137	.	0.7137	.	.	.
Chlorella+ADT:Curcumin	0	0	1.0002	.	1.0002	.	.	.
Chlorella+ADT:Curcumin+ADT	0	0	1.3259	.	1.3259	.	.	.
Chlorella+ADT:Curcumin+Saffron+ADT	0	0	1.2649	.	1.2649	.	.	.
Chlorella+ADT:DHA	0	0	2.1241	.	2.1241	.	.	.
Chlorella+ADT:DHA+ADT	0	0	0.8946	.	0.8946	.	.	.
Chlorella+ADT:EPA	0	0	1.6573	.	1.6573	.	.	.
Chlorella+ADT:Folate+ADT	0	0	1.6048	.	1.6048	.	.	.
Chlorella+ADT:Inositol+ADT	0	0	1.2351	.	1.2351	.	.	.
Chlorella+ADT:Mg	0	0	1.8977	.	1.8977	.	.	.
Chlorella+ADT:Mg+ADT	0	0	17.6133	.	17.6133	.	.	.
Chlorella+ADT:Nepta	0	0	1.4182	.	1.4182	.	.	.
Chlorella+ADT:PEA+ADT	0	0	1.4182	.	1.4182	.	.	.
Chlorella+ADT:Placebo	0	0	1.769	.	1.769	.	.	.
Chlorella+ADT:Probiotics	0	0	1.4023	.	1.4023	.	.	.
Chlorella+ADT:Probiotics+ADT	0	0	1.302	.	1.302	.	.	.
Chlorella+ADT:Probiotics+B7	0	0	0.7784	.	0.7784	.	.	.
Chlorella+ADT:R_rosea	0	0	3.1955	.	3.1955	.	.	.
Chlorella+ADT:Saffron	0	0	1.8022	.	1.8022	.	.	.
Chlorella+ADT:Saffron+ADT	0	0	1.4182	.	1.4182	.	.	.
Chlorella+ADT:SAMe	0	0	1.8367	.	1.8367	.	.	.
Chlorella+ADT:SAMe+ADT	0	0	2.6793	.	2.6793	.	.	.
Chlorella+ADT:SAMe+EPA+DHA+Folic+5HTP+ Zinc+ADT	0	0	1.1203	.	1.1203	.	.	.
Chlorella+ADT:SAMe+Vitamin_B12+Folate	0	0	0.9825	.	0.9825	.	.	.
Chlorella+ADT:SJW	0	0	1.9507	.	1.9507	.	.	.

Chlorella+ADT:Vitamin_C+ADT	0	0	2.3637	.	2.3637	.	.	.
Chlorella+ADT:Vitamin_D	0	0	5.0193	.	5.0193	.	.	.
Chlorella+ADT:Vitamin_D+ADT	0	0	2.9311	.	2.9311	.	.	.
Chlorella+ADT:Zinc	0	0	2.6957	.	2.6957	.	.	.
Chlorella+ADT:Zinc+ADT	0	0	0.6025	.	0.6025	.	.	.
Chlorella+ADT:Zinc+Vitamin_D	0	0	3.5759	.	3.5759	.	.	.
Chromium:Creatine+ADT	0	0	0.4031	.	0.4031	.	.	.
Chromium:Curcumin	0	0	0.5649	.	0.5649	.	.	.
Chromium:Curcumin+ADT	0	0	0.7488	.	0.7488	.	.	.
Chromium:Curcumin+Saffron+ADT	0	0	0.7144	.	0.7144	.	.	.
Chromium:DHA	0	0	1.1996	.	1.1996	.	.	.
Chromium:DHA+ADT	0	0	0.5052	.	0.5052	.	.	.

16.3.F. Evaluation of the inconsistency by node split model for outcome as adverse event

Comparison	k	prop	nma	direct	indir.	Diff	z	p-value
ADT:Carnitine	1	1	3.9581	3.9581				
ADT:Chromium	0	0	3.1847		3.1847			
ADT:Creatine	0	0	1.3404		1.3404			
ADT:Curcumin	1	1	0.9355	0.9355				
ADT:Curcumin+ADT	1	1	0.3959	0.3959				
ADT:DHA+ADT	1	1	1.1277	1.1277				
ADT:EPA	0	0	0.7398		0.7398			
ADT:EPA +DHA+ADT	1	1	1.1482	1.1482				
ADT:EPA+DHA	0	0	3.09		3.09			
ADT:EPA+DHA+ADT	3	1	0.994	0.994				
ADT:Inositol+ADT	0	0	1.2618		1.2618			
ADT:Mg	1	1	6.5789	6.5789				
ADT:Placebo	5	0.51	1.9638	2.1133	1.8187	1.162	0.41	0.6807
ADT:R_rosea	1	0.81	2.1766	3.7179	0.2211	16.8189	1.6	0.109
ADT:SAMe	4	0.95	2.428	2.1074	39.223	0.0537	-2.24	0.0251
ADT:SAMe+ADT	1	1	0.804	0.804	.	.	.	.
ADT:SJW	14	0.91	1.7058	1.7826	1.0967	1.6254	1.04	0.2962
ADT:Tryptophan+ADT	1	1	0.3782	0.3782	.	.	.	.
Carnitine:Chromium	0	0	0.8046	.	0.8046	.	.	.
Carnitine:Creatine	0	0	0.3386	.	0.3386	.	.	.
Carnitine:Curcumin	0	0	0.2363	.	0.2363	.	.	.
Carnitine:Curcumin+ADT	0	0	0.1	.	0.1	.	.	.
Carnitine:DHA+ADT	0	0	0.2849	.	0.2849	.	.	.
Carnitine:EPA	0	0	0.1869	.	0.1869	.	.	.
Carnitine:EPA +DHA+ADT	0	0	0.2901	.	0.2901	.	.	.
Carnitine:EPA+DHA	0	0	0.7807	.	0.7807	.	.	.
Carnitine:EPA+DHA+ADT	0	0	0.2511	.	0.2511	.	.	.
Carnitine:Inositol+ADT	0	0	0.3188	.	0.3188	.	.	.
Carnitine:Mg	0	0	1.6621	.	1.6621	.	.	.
Carnitine:Placebo	0	0	0.4962	.	0.4962	.	.	.
Carnitine:R_rosea	0	0	0.5499	.	0.5499	.	.	.
Carnitine:SAMe	0	0	0.6134	.	0.6134	.	.	.
Carnitine:SAMe+ADT	0	0	0.2031	.	0.2031	.	.	.
Carnitine:SJW	0	0	0.431	.	0.431	.	.	.

Carnitine:Tryptophan+ADT	0	0	0.0955	.	0.0955	.	.	.
Chromium:Creatine	0	0	0.4209	.	0.4209	.	.	.
Chromium:Curcumin	0	0	0.2937	.	0.2937	.	.	.
Chromium:Curcumin+ADT	0	0	0.1243	.	0.1243	.	.	.
Chromium:DHA+ADT	0	0	0.3541	.	0.3541	.	.	.
Chromium:EPA	0	0	0.2323	.	0.2323	.	.	.
Chromium:EPA +DHA+ADT	0	0	0.3605	.	0.3605	.	.	.
Chromium:EPA+DHA	0	0	0.9702	.	0.9702	.	.	.
Chromium:EPA+DHA+ADT	0	0	0.3121	.	0.3121	.	.	.
Chromium:Inositol+ADT	0	0	0.3962	.	0.3962	.	.	.
Chromium:Mg	0	0	2.0658	.	2.0658	.	.	.
Chromium:Placebo	1	1	0.6166	0.6166	.	.	.	.
Chromium:R_rosea	0	0	0.6835	.	0.6835	.	.	.
Chromium:SAMe	0	0	0.7624	.	0.7624	.	.	.
Chromium:SAMe+ADT	0	0	0.2525	.	0.2525	.	.	.
Chromium:SJW	0	0	0.5356	.	0.5356	.	.	.
Chromium:Tryptophan+ADT	0	0	0.1187	.	0.1187	.	.	.
Creatine:Curcumin	0	0	0.6979	.	0.6979	.	.	.
Creatine:Curcumin+ADT	0	0	0.2954	.	0.2954	.	.	.
Creatine:DHA+ADT	0	0	0.8413	.	0.8413	.	.	.
Creatine:EPA	0	0	0.5519	.	0.5519	.	.	.
Creatine:EPA +DHA+ADT	0	0	0.8566	.	0.8566	.	.	.
Creatine:EPA+DHA	0	0	2.3053	.	2.3053	.	.	.
Creatine:EPA+DHA+ADT	0	0	0.7415	.	0.7415	.	.	.
Creatine:Inositol+ADT	0	0	0.9414	.	0.9414	.	.	.
Creatine:Mg	0	0	4.9082	.	4.9082	.	.	.
Creatine:Placebo	1	1	1.4651	1.4651	.	.	.	.
Creatine:R_rosea	0	0	1.6239	.	1.6239	.	.	.
Creatine:SAMe	0	0	1.8114	.	1.8114	.	.	.
Creatine:SAMe+ADT	0	0	0.5998	.	0.5998	.	.	.
Creatine:SJW	0	0	1.2726	.	1.2726	.	.	.
Creatine:Tryptophan+ADT	0	0	0.2821	.	0.2821	.	.	.
Curcumin:Curcumin+ADT	1	1	0.4232	0.4232	.	.	.	.
Curcumin:DHA+ADT	0	0	1.2054	.	1.2054	.	.	.

Curcumin:EPA	0	0	0.7908	.	0.7908	.	.	.
Curcumin:EPA +DHA+ADT	0	0	1.2274	.	1.2274	.	.	.
Curcumin:EPA+DHA	0	0	3.3031	.	3.3031	.	.	.
Curcumin:EPA+DHA+ADT	0	0	1.0625	.	1.0625	.	.	.
Curcumin:Inositol+ADT	0	0	1.3488	.	1.3488	.	.	.
Curcumin:Mg	0	0	7.0327	.	7.0327	.	.	.
Curcumin:Placebo	0	0	2.0993	.	2.0993			
Curcumin:R_rosea	0	0	2.3268	.	2.3268	.	.	
Curcumin:SAMe	0	0	2.5954	.	2.5954	.	.	
Curcumin:SAMe+ADT	0	0	0.8595	.	0.8595	.	.	
Curcumin:SJW	0	0	1.8235	.	1.8235	.	.	
Curcumin:Tryptophan+ADT	0	0	0.4042	.	0.4042	.	.	
Curcumin+ADT:DHA+ADT	0	0	2.8484	.	2.8484	.	.	
Curcumin+ADT:EPA	0	0	1.8687	.	1.8687	.	.	
Curcumin+ADT:EPA +DHA+ADT	0	0	2.9003	.	2.9003	.	.	
Curcumin+ADT:EPA+DHA	0	0	7.805	.	7.805	.	.	
Curcumin+ADT:EPA+DHA+ADT	0	0	2.5107	.	2.5107	.	.	
Curcumin+ADT:Inositol+ADT	0	0	3.1872	.	3.1872	.	.	
Curcumin+ADT:Mg	0	0	16.6179	.	16.6179	.	.	
Curcumin+ADT:Placebo	0	0	4.9605	.	4.9605	.	.	
Curcumin+ADT:R_rosea	0	0	5.498	.	5.498	.	.	
Curcumin+ADT:SAMe	0	0	6.1328	.	6.1328	.	.	
Curcumin+ADT:SAMe+ADT	0	0	2.0309	.	2.0309	.	.	
Curcumin+ADT:SJW	0	0						
Curcumin+ADT:SJW	0	0	4.3088	.	4.3088	.	.	
Curcumin+ADT:Tryptophan+ADT	0	0	0.9552	.	0.9552	.	.	
DHA+ADT:EPA	0	0	0.656	.	0.656	.	.	
DHA+ADT:EPA +DHA+ADT	0	0	1.0182	.	1.0182	.	.	
DHA+ADT:EPA+DHA	0	0	2.7402	.	2.7402	.	.	
DHA+ADT:EPA+DHA+ADT	0	0	0.8814	.	0.8814	.	.	
DHA+ADT:Inositol+ADT	0	0	1.1189	.	1.1189	.	.	

DHA+ADT:Mg	0	0	5.8342	.	5.8342	.	.	
DHA+ADT:Placebo	0	0	1.7415	.	1.7415	.	.	
DHA+ADT:R_rosea	0	0	1.9302	.	1.9302	.	.	
DHA+ADT:SAMe	0	0	2.1531	.	2.1531	.	.	
DHA+ADT:SAMe+ADT	0	0	0.713	.	0.713	.	.	
DHA+ADT:SJW	0	0	1.5127	.	1.5127	.	.	
DHA+ADT:Tryptophan+ADT	0	0	0.3353	.	0.3353	.	.	
EPA:EPA +DHA+ADT	0	0	1.5521	.	1.5521	.	.	
EPA:EPA+DHA	0	0	4.1768	.	4.1768	.	.	
EPA:EPA+DHA+ADT	0	0	1.3436	.	1.3436	.	.	
EPA:Inositol+ADT	0	0						
EPA:Inositol+ADT	0	0	1.7056	.	1.7056	.	.	.
EPA:Mg	0	0	8.893	.	8.893	.		

17.Comparison-adjusted funnel plot for each outcome from the network meta-analysis

17.1. Funnel plot of change in depressive sympotms

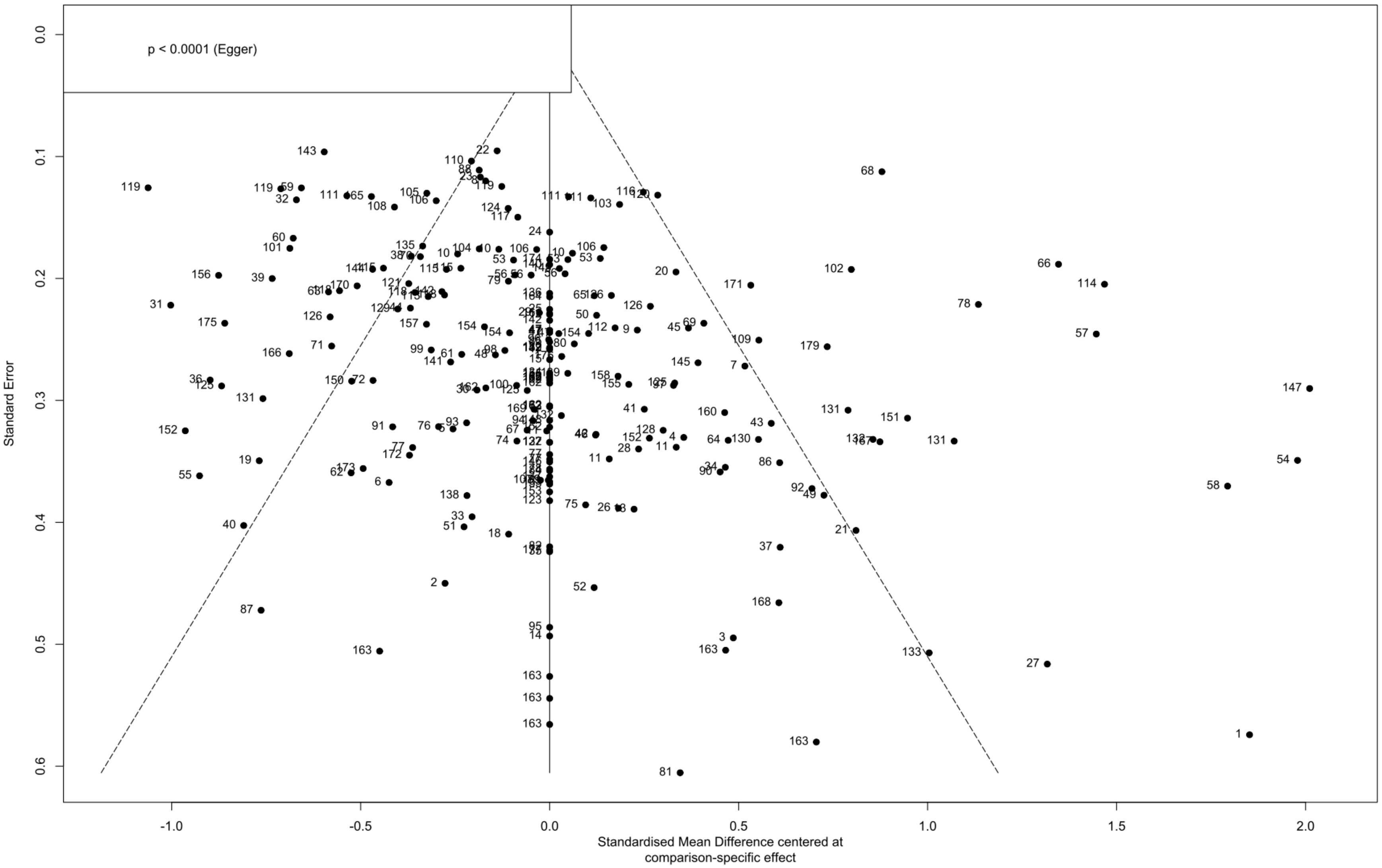

17.2. Funnel plot of reponse rate

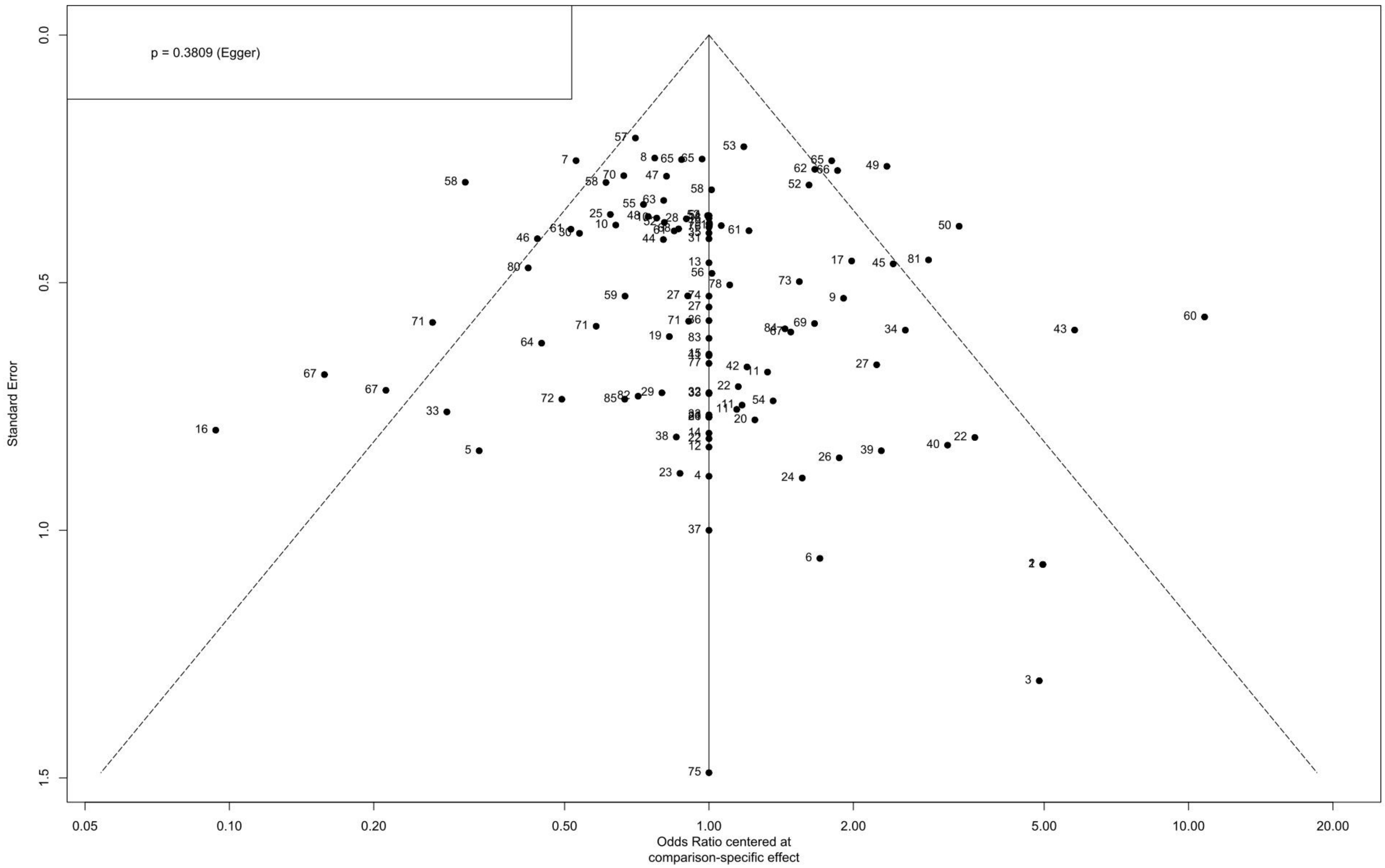

17.3. Funnel plot of remission rate

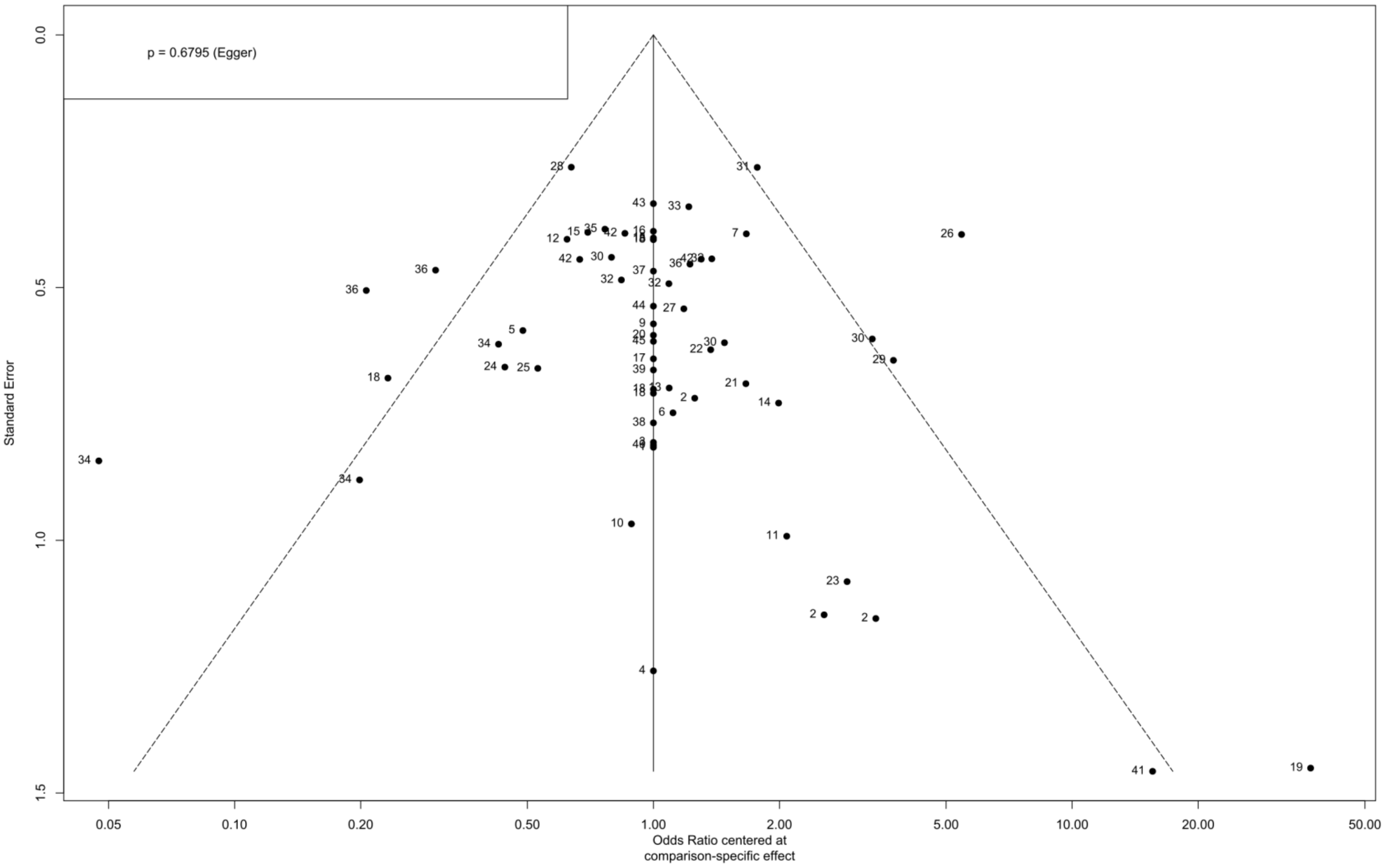

17.4. Funnel plot of change in anxiety sympotms

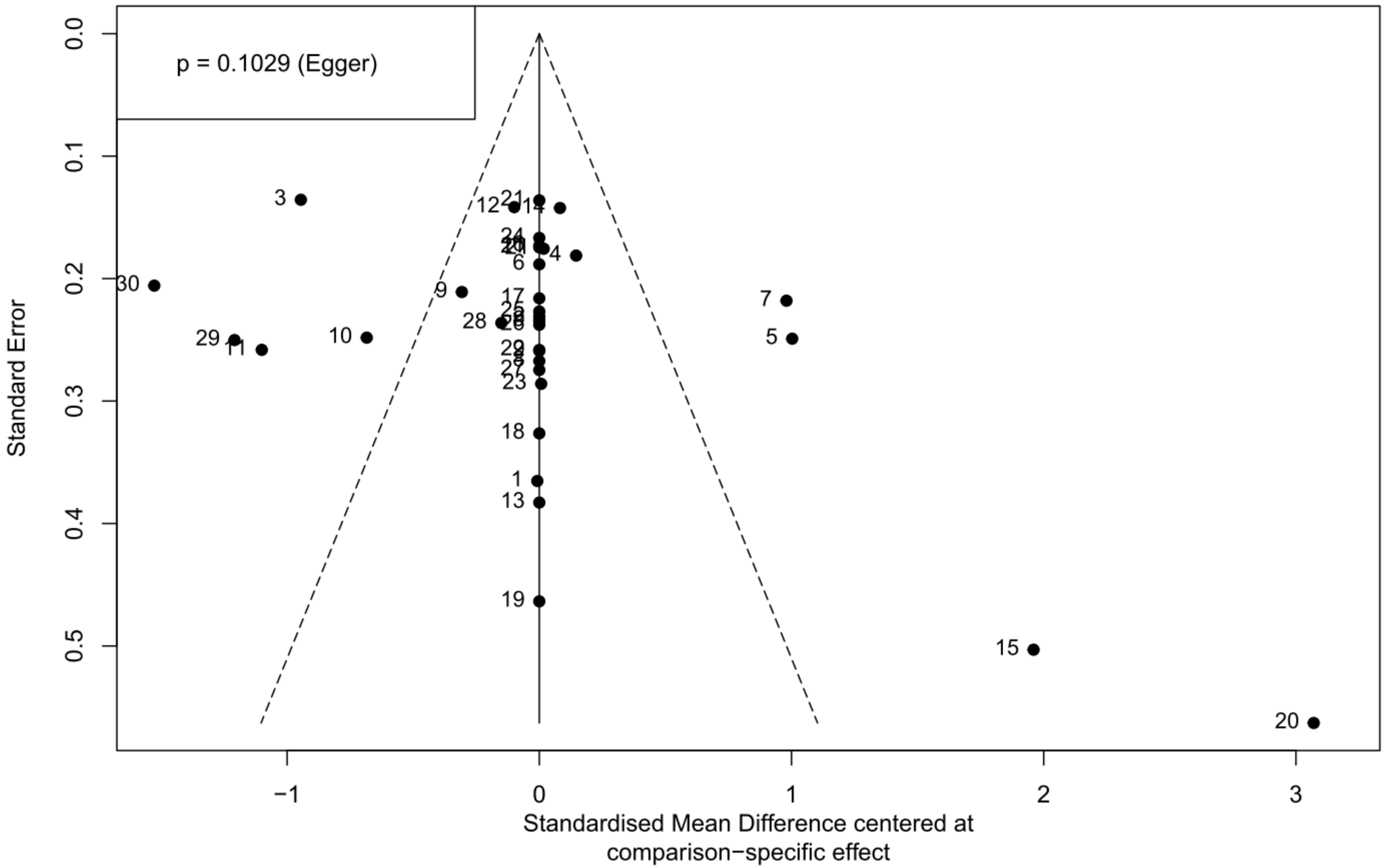

17.5. Funnel plot of all cause discontinuation

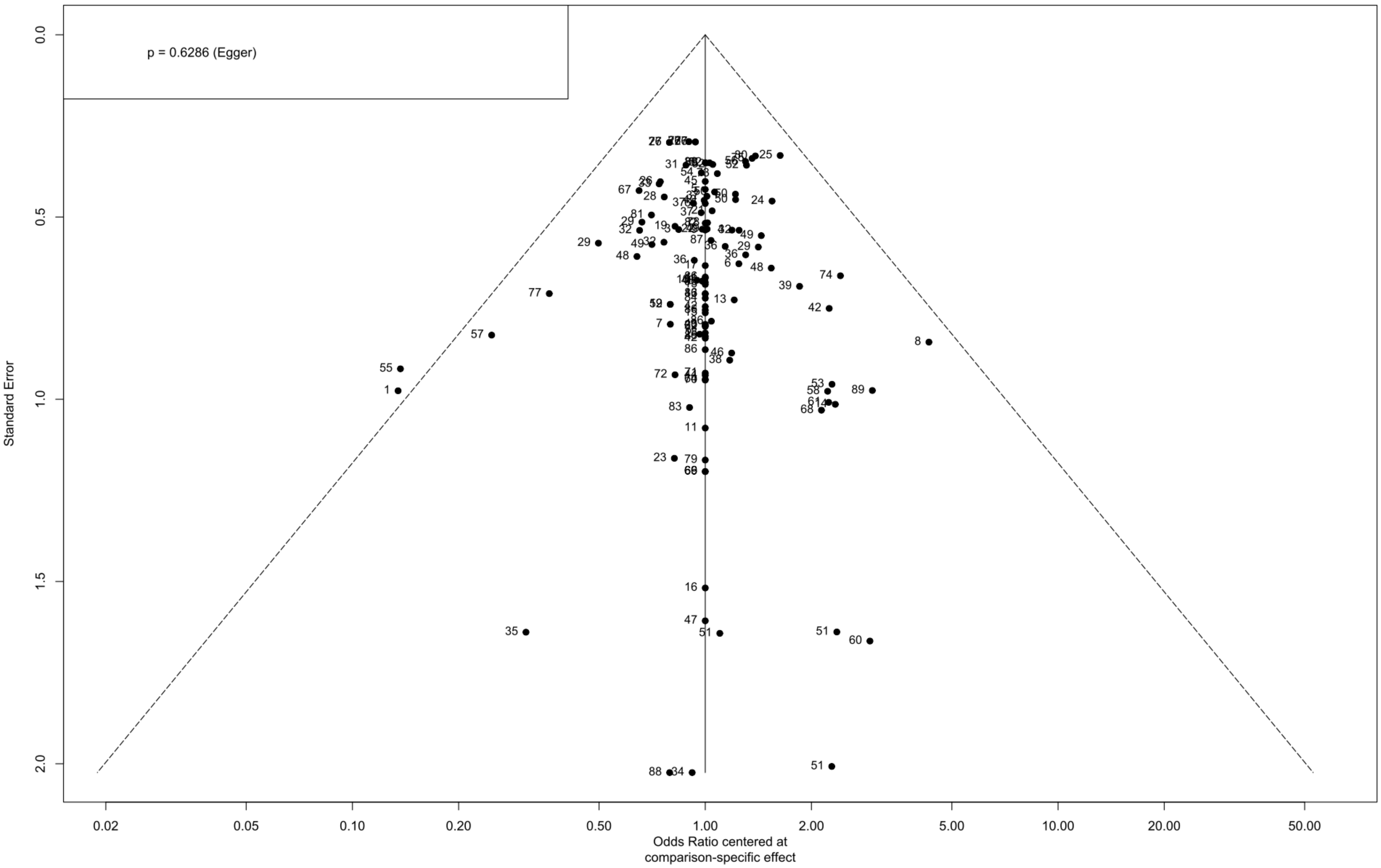

17.6. Funnel plot of adverse event

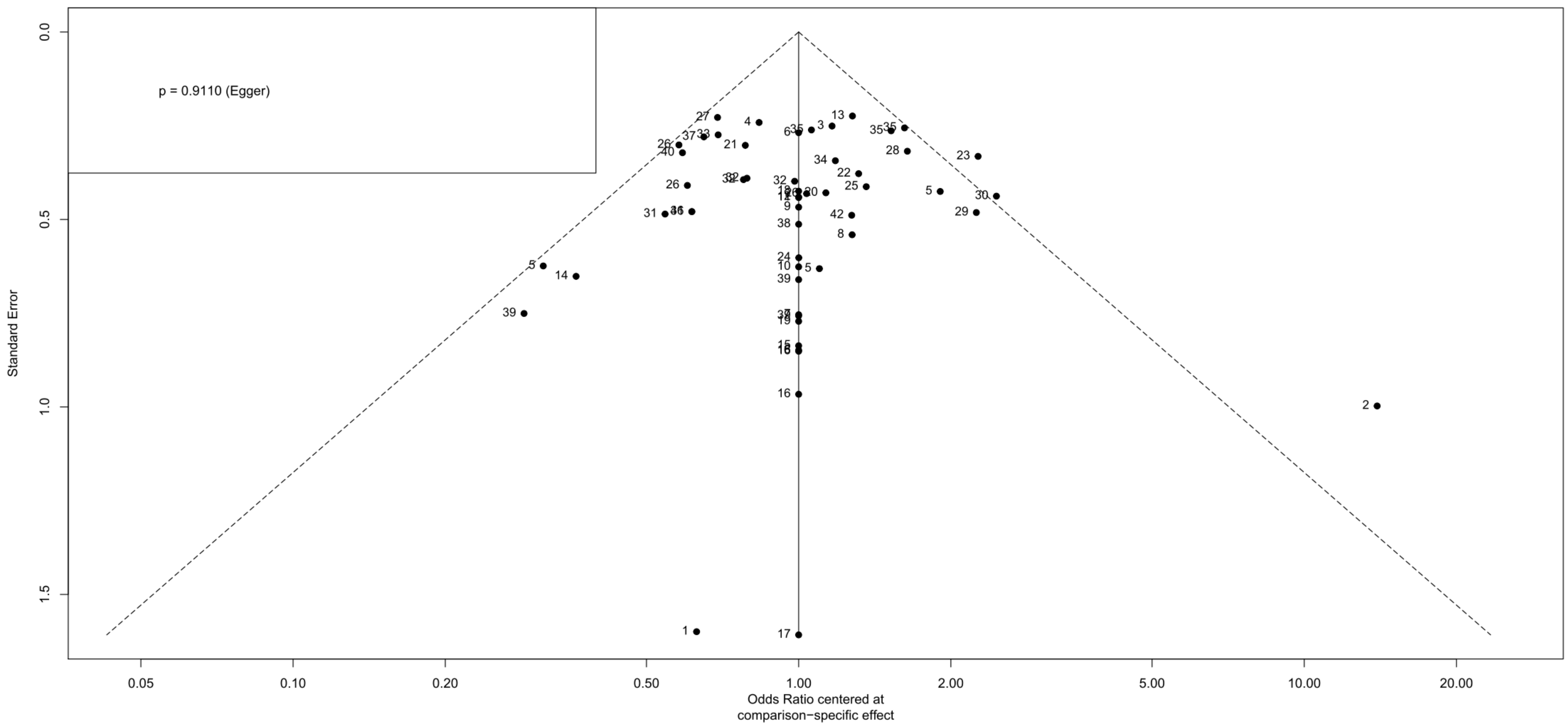

18.Meta-regression

Treatment intervention and their cooresponding code

Treatment	Corresponding code
ADT	AA
Amino_acid+ADT	AB
Carnitine	AC
Carnitine+ADT	AD
Chlorella+ADT	AE
Creatine+ADT	AF
Curcumin	AG
Curcumin+ADT	AH
Curcumin+Saffron+ADT	AI
DHA	AJ
DHA+ADT	AK
EPA	AL
EPA+ADT	AM
EPA+DHA	AN
EPA+DHA+ADT	AO
EPA+DHA+Vitamin_C	AP
E_amoenum	AQ
Fe	AR
Folate+ADT	AS
Folate+Vitamin_B12+Vitamin_B6	AT
Ginkgo+ADT	AU
Inositol+ADT	AV
L-arginine+ADT	AW
L-theanine+ADT	AX
Lavandula	AY
Lavandula+ADT	AZ
Mg	BA
Mg+ADT	BB
Nepta	BC
PEA+ADT	BD
Placebo (reference)	BE
Prebiotics+ADT	BF
Probiotics	BG
Probiotics+ADT	BH
Probiotics+B7	BI
Probiotics+Mg+CoQ10+ADT	BJ
R_rosea	BK

SAMe	BL
SAMe+ADT	BM
SAMe+Probiotics	BN
SAMe+Vitamin_B12+Folate+ADT	BO
SAMe+n3+Folate+5HTP+Zinc+ADT	BP
SJW	BQ
SJW+ADT	BR
SJW+Kava	BS
Saffron	BT
Saffron+ADT	BU
Tryptophan+ADT	BV
Vitamin_B+ADT	BW
Vitamin_B1+ADT	BX
Vitamin_B12+ADT	BY
Vitamin_B6+Tryptophan	BZ
Vitamin_C	CA
Vitamin_C+ADT	CB
Vitamin_D	CC
Vitamin_D+ADT	CD
Vitamin_D+Ca	CE
Zinc	CF
Zinc+ADT	CG
Zinc+Vitamin_D	CH

ADT: Antidepressant; Ca: Calcium ; DHA: Docosahexaenoic Acid ; E Amoenum : Echium amoenum; EPA :Eicosapentaenoic acid; Fe : Ferrum; Mg: Magnesium; PEA: Palmitoylethanolamide; R rosea : Rhodiola rosea; SAMe: S-Adenosyl Methionine ; SJW: St. John's wort ;Vitamin B1: Thiamine ; Vitamin B6: Pyridoxine; Vitamin B7: Biotin; Vitamin B: Vitamin B complex; Vitamin B12 : Cobalamin; Vitamin C: Ascorbic acid; Vitamin D: Cholecalciferol; 5HTP: 5-Hydroxytryptophan

```
Multivariate meta-analysis
Variance-covariance matrix = proportional .5*I(55)+.5*J(55,55,1)
Method = reml                                  Number of dimensions     =     55
Restricted log likelihood = -708.60858         Number of observations   =    165
------------------------------------------------------------------------------
             | Coefficient  Std. err.      z    P>|z|     (95% conf. interval)
-------------+----------------------------------------------------------------
_y_AA        |
       _cons |   .4217411   .1175861     3.59   0.000     .1912765    .6522057
-------------+----------------------------------------------------------------
_y_AB        |
```

_cons	1.975754	.6787467	2.91	0.004	.6454349	3.306073
_y_AC						
_cons	1.032368	.462954	2.23	0.026	.1249953	1.939742
_y_AD						
_cons	1.745375	.6717849	2.60	0.009	.4287008	3.062049
_y_AE						
_cons	.8937753	.6095358	1.47	0.143	-.300893	2.088444
_y_AF						
_cons	1.011288	.5047054	2.00	0.045	.022083	2.000492
_y_AG						
_cons	.5635306	.4232678	1.33	0.183	-.266059	1.39312
_y_AH						
_cons	.9004484	.2508539	3.59	0.000	.4087838	1.392113
_y_AI						
_cons	.6511302	.5564395	1.17	0.242	-.4394711	1.741732
_y_AJ						
_cons	.0054875	.3337666	0.02	0.987	-.648683	.659658
_y_AK						
_cons	.5096137	.435407	1.17	0.242	-.3437684	1.362996
_y_AL						
_cons	.0710552	.2751245	0.26	0.796	-.4681789	.6102893
_y_AM						
_cons	.9910037	.345741	2.87	0.004	.3133638	1.668644
_y_AN						
_cons	.5928784	.1582649	3.75	0.000	.282685	.9030718
_y_AO						
_cons	1.044625	.2219665	4.71	0.000	.609579	1.479672
_y_AP						
_cons	.1090864	.5338084	0.20	0.838	-.9371589	1.155332
_y_AQ						
_cons	.6915604	.6518169	1.06	0.289	-.5859773	1.969098
_y_AR						
_cons	.9348108	.6134813	1.52	0.128	-.2675904	2.137212

_y_AS						
_cons	.5684248	.2504885	2.27	0.023	.0774763	1.059373
_y_AT						
_cons	.1791635	.5821287	0.31	0.758	-.9617878	1.320115
_y_AU						
_cons	1.174913	.4381124	2.68	0.007	.3162283	2.033597
_y_AV						
_cons	.4730707	.4844799	0.98	0.329	-.4764926	1.422634
_y_AW						
_cons	.1003069	.679041	0.15	0.883	-1.230589	1.431203
_y_AX						
_cons	.8954311	.6801755	1.32	0.188	-.4376884	2.228551
_y_AY						
_cons	.6637815	.4694431	1.41	0.157	-.25631	1.583873
_y_AZ						
_cons	.811809	.6743357	1.20	0.229	-.5098647	2.133483
_y_BA						
_cons	1.247724	.6265961	1.99	0.046	.0196185	2.47583
_y_BB						
_cons	.9747235	.6353155	1.53	0.125	-.270472	2.219919
_y_BD						
_cons	.3487701	.3129082	1.11	0.265	-.2645188	.9620589
_y_BE						
_cons	.7886052	.3655107	2.16	0.031	.0722174	1.504993
_y_BF						
_cons	-.1113434	.6154242	-0.18	0.856	-1.317553	1.094866
_y_BG						
_cons	.8747168	.42277	2.07	0.039	.0461028	1.703331
_y_BH						
_cons	.4617945	.1830787	2.52	0.012	.1029668	.8206221
_y_BI						
_cons	.9038124	.387474	2.33	0.020	.1443774	1.663247

_y_BJ						
_cons	.209792	.6017109	0.35	0.727	-.9695397	1.389124
_y_BK						
_cons	.4804373	.5991032	0.80	0.423	-.6937834	1.654658
_y_BL						
_cons	.3265479	.6157753	0.53	0.596	-.8803494	1.533445
_y_BM						
_cons	.4227429	.1220931	3.46	0.001	.1834449	.662041
_y_BN						
_cons	.5155893	.6531251	0.79	0.430	-.7645123	1.795691
_y_BO						
_cons	.3658554	.6774733	0.54	0.589	-.9619679	1.693679
_y_BP						
_cons	.6136239	.1933712	3.17	0.002	.2346234	.9926244
_y_BQ						
_cons	3.655445	.7581224	4.82	0.000	2.169553	5.141338
_y_BR						
_cons	1.148755	.4943152	2.32	0.020	.1799155	2.117595
_y_BS						
_cons	.38279	.6269991	0.61	0.542	-.8461057	1.611686
_y_BT						
_cons	.6094854	.6366239	0.96	0.338	-.6382746	1.857245
_y_BU						
_cons	1.93406	.6310746	3.06	0.002	.6971763	3.170943
_y_BV						
_cons	-.8317805	.7602128	-1.09	0.274	-2.32177	.6582092
_y_BW						
_cons	.1349127	.5337798	0.25	0.800	-.9112765	1.181102
_y_BX						
_cons	.3643236	.6477377	0.56	0.574	-.905219	1.633866
_y_BY						
_cons	.7211241	.2039522	3.54	0.000	.3213852	1.120863
_y_BZ						

_cons	.9567717	.3787565	2.53	0.012	.2144226	1.699121
_y_CA						
_cons	.2436551	.550067	0.44	0.658	-.8344564	1.321767
_y_CB						
_cons	1.172384	.557934	2.10	0.036	.0788531	2.265914
_y_CC						
_cons	1.479672	.5175545	2.86	0.004	.4652834	2.49406
_y_CD						
_cons	1.275658	.5573219	2.29	0.022	.1833269	2.367989

18.1. Meta-regression : Covariate as publication year

Note: 179 observations on 59 variables
Note: variance-covariance matrix is proportional to .5*I(59)+.5*J(59,59,1)

initial: log likelihood = -885.09472
rescale: log likelihood = -885.09472
rescale eq: log likelihood = -866.35195
Iteration 0: log likelihood = -866.35195
Iteration 1: log likelihood = -864.73823
Iteration 2: log likelihood = -864.68497
Iteration 3: log likelihood = -864.68481
Iteration 4: log likelihood = -864.68481

Multivariate meta-analysis
Variance-covariance matrix = proportional .5*I(59)+.5*J(59,59,1)
Method = reml Number of dimensions
= 59
Restricted log likelihood = -864.68481 Number of observations = 179

	Coefficient	Std. err.	z	P>|z|	[95% conf.	interval]
_y_AA						
year	.0003401	.0177076	0.02	0.985	-.0343662	.0350464
_cons	-.2113337	35.5395	-0.01	0.995	-69.86747	69.4448
_y_AB						
_cons	2.025295	.6925184	2.92	0.003	.6679841	3.382606
_y_AC						
year	-.026406	.0435913	-0.61	0.545	-.1118435	.0590314
_cons	53.97176	87.32556	0.62	0.537	-117.1832	225.1267
_y_AD						
_cons	1.797637	.7001589	2.57	0.010	.4253508	3.169923
_y_AE						
_cons	.9460374	.6406724	1.48	0.140	-.3096575	2.201732
_y_AF						
year	-1.338	1.43304	-0.93	0.350	-4.146707	1.470707
_cons	2693.721	2883.921	0.93	0.350	-2958.66	8346.101
_y_AG						
year	.0048587	.147728	0.03	0.974	-.2846828	.2944003
_cons	-9.207012	297.9741	-0.03	0.975	-593.2254	574.8114
_y_AH						
year	.0388462	.148355	0.26	0.793	-.2519242	.3296166
_cons	-77.27903	298.9486	-0.26	0.796	-663.2075	508.6495
_y_AI						
_cons	.765949	.6144538	1.25	0.213	-.4383584	1.970256
_y_AJ						
year	-.0281371	.0629187	-0.45	0.655	-.1514556	.0951813
_cons	56.62925	126.5882	0.45	0.655	-191.4791	304.7376
_y_AK						
year	-.1235428	.109087	-1.13	0.257	-.3373495	.0902638
_cons	248.7622	219.1692	1.14	0.256	-180.8016	678.326
_y_AL						
year	.0025828	.0615381	0.04	0.967	-.1180297	.1231952
_cons	-5.145387	123.7464	-0.04	0.967	-247.6838	237.393
_y_AM						
year	-.0751608	.0591177	-1.27	0.204	-.1910293	.0407077
_cons	152.2059	118.9253	1.28	0.201	-80.88336	385.2952
_y_AN						
year	-.0129985	.0322502	-0.40	0.687	-.0762077	.0502107
_cons	26.7704	64.94722	0.41	0.680	-100.5238	154.0646
_y_AO						
year	.0149509	.0421848	0.35	0.723	-.0677298	.0976317
_cons	-29.07163	84.85445	-0.34	0.732	-195.3833	137.24
_y_AP						
_cons	.1148131	.5448531	0.21	0.833	-.9530794	1.182706
_y_AQ						
_cons	.6915604	.66385	1.04	0.298	-.6095618	1.992683
_y_AR						
_cons	.9348108	.6262514	1.49	0.136	-.2926194	2.162241
_y_AS						
year	-.0053787	.0287577	-0.19	0.852	-.0617428	.0509854
_cons	11.39834	57.67037	0.20	0.843	-101.6335	124.4302
_y_AT						
_cons	.1791635	.5955715	0.30	0.764	-.9881352	1.346462

_y_AT						
_cons	.1791635	.5955715	0.30	0.764	-.9881352	1.346462
_y_AU						
year	.6945784	.8580056	0.81	0.418	-.9870817	2.376238
_cons	-1400.764	1731.864	-0.81	0.419	-4795.156	1993.627
_y_AV						
_cons	.5200205	.515551	1.01	0.313	-.4904408	1.530482
_y_AW						
_cons	.8289299	.7504451	1.10	0.269	-.6419155	2.299775
_y_AX						
_cons	3.570033	.7818397	4.57	0.000	2.037655	5.102411
_y_AY						
_cons	.1484876	.6964272	0.21	0.831	-1.216485	1.51346
_y_AZ						
_cons	.9436118	.6975335	1.35	0.176	-.4235287	2.310752
_y_BA						
year	-.017824	.1074848	-0.17	0.868	-.2284903	.1928424
_cons	36.56557	216.3733	0.17	0.866	-387.5183	460.6494
_y_BB						
_cons	.8650915	.7152059	1.21	0.226	-.5366862	2.266869
_y_BC						
_cons	1.300327	.6609667	1.97	0.049	.0048556	2.595797
_y_BD						
_cons	1.028006	.6785402	1.52	0.130	-.3019084	2.35792
_y_BF						
year	-.5278624	.5261987	-1.00	0.316	-1.559193	.5034681
_cons	1068.555	1064.541	1.00	0.315	-1017.906	3155.017
_y_BG						
year	-.0918712	.2893873	-0.32	0.751	-.6590598	.4753174
_cons	185.7024	583.8466	0.32	0.750	-958.6159	1330.021
_y_BH						
year	.0141313	.0978578	0.14	0.885	-.1776666	.2059291
_cons	-27.42587	197.7753	-0.14	0.890	-415.0583	360.2066
_y_BI						
_cons	-.1113434	.6281549	-0.18	0.859	-1.342504	1.119818
_y_BJ						
_cons	.8495245	.6854703	1.24	0.215	-.4939725	2.193022
_y_BK						
year	-.2304669	.1092244	-2.11	0.035	-.4445428	-.0163909
_cons	464.4872	219.7057	2.11	0.035	33.872	895.1024
_y_BL						
year	-.0116516	.0162122	-0.72	0.472	-.043427	.0201238
_cons	23.85349	32.49258	0.73	0.463	-39.8308	87.53779
_y_BM						
year	-.027738	.0380551	-0.73	0.466	-.1023247	.0468488
_cons	56.65951	76.3981	0.74	0.458	-93.07801	206.397
_y_BN						
_cons	.4804373	.6121734	0.78	0.433	-.7194005	1.680275
_y_BO						
_cons	.3798304	.6602804	0.58	0.565	-.9142954	1.673956
_y_BP						
_cons	.2634147	.6527298	0.40	0.687	-1.015912	1.542742
_y_BQ						
year	-.0125131	.0270475	-0.46	0.644	-.0655253	.0404992
_cons	25.51307	54.20928	0.47	0.638	-80.73517	131.7613
_y_BR						
_cons	.566831	.6732613	0.84	0.400	-.7527369	1.886399
_y_BS						
_cons	.3658554	.6890585	0.53	0.595	-.9846745	1.716385
_y_BT						
year	.0032574	.0300176	0.11	0.914	-.0555761	.0620908
_cons	-5.881793	60.42321	-0.10	0.922	-124.3091	112.5455
_y_BU						
_cons	3.707707	.7833754	4.73	0.000	2.17232	5.243095
_y_BV						
year	.0151754	.0380523	0.40	0.690	-.0594057	.0897565
_cons	-28.93751	75.72796	-0.38	0.702	-177.3616	119.4866
_y_BW						
_cons	.4343719	.6506013	0.67	0.504	-.8407833	1.709527
_y_BX						
_cons	.6620877	.6704809	0.99	0.323	-.6520307	1.976206
_y_BY						
_cons	1.985642	.6545297	3.03	0.002	.7027868	3.268496
_y_BZ						
_cons	-.7910823	.8993631	-0.88	0.379	-2.553802	.971637
_y_CA						
_cons	.1406411	.544825	0.26	0.796	-.9271962	1.208478
_y_CB						
_cons	.4165857	.67712	0.62	0.538	-.9105451	1.743717
_y_CC						
year	-.0796449	.0895602	-0.89	0.374	-.2551796	.0958898
_cons	161.4094	180.7163	0.89	0.372	-192.7881	515.6069
_y_CD						
year	-.1068707	.0985391	-1.08	0.278	-.3000038	.0862623
_cons	216.6401	198.8641	1.09	0.276	-173.1263	606.4065
_y_CE						
_cons	.1467799	.5706022	0.26	0.797	-.97158	1.26514
_y_CF						
_cons	1.071972	.5788619	1.85	0.064	-.0625769	2.20652
_y_CG						
year	-.0743139	.1792048	-0.41	0.678	-.4255488	.276921
_cons	150.6643	359.6298	0.42	0.675	-554.1971	855.5257
_y_CH						
_cons	1.1751	.5783004	2.03	0.042	.0416516	2.308548

18.2. Meta-regression : Covariate as industry sponsorship

Note: 179 observations on 59 variables

Note: variance-covariance matrix is proportional to .5*I(59)+.5*J(59,59,1)

initial: log likelihood = -834.44472

rescale: log likelihood = -834.44472

rescale eq: log likelihood = -815.36993

Iteration 0: log likelihood = -815.36993

Iteration 1: log likelihood = -813.60918

Iteration 2: log likelihood = -813.59872

Iteration 3: log likelihood = -813.59872

Multivariate meta-analysis

Variance-covariance matrix = proportional .5*I(59)+.5*J(59,59,1)

Method = reml Number of dimensions = 59

Restricted log likelihood = -813.59872 Number of observations = 179

	Coefficient	Std. err.	z	P>|z|	[95% conf. interval]	
_y_AA						
_Iindustry_2	-.0676055	.2972615	-0.23	0.820	-.6502274	.5150165
_Iindustry_3	-.0528798	.3648703	-0.14	0.885	-.7680126	.6622529
_cons	.4964703	.1731519	2.87	0.004	.1570987	.8358418
_y_AB						
_cons	2.050483	.7015366	2.92	0.003	.6754964	3.425469
_y_AC						
_Iindustry_3	.5577855	.959032	0.58	0.561	-1.321883	2.437454
_cons	.839536	.63931	1.31	0.189	-.4134886	2.092561
_y_AD						
_cons	1.767224	.7456072	2.37	0.018	.3058608	3.228587
_y_AE						
_cons	.9156245	.6900502	1.33	0.185	-.4368489	2.268098
_y_AF						
_cons	1.083961	.5278361	2.05	0.040	.0494217	2.118501
_y_AG						
_cons	.5677701	.4384527	1.29	0.195	-.2915814	1.427122
_y_AH						
_Iindustry_2	-.1425183	.75029	-0.19	0.849	-1.61306	1.328023
_Iindustry_3	1.260163	.764781	1.65	0.099	-.2387806	2.759106
_cons	.8431081	.323229	2.61	0.009	.2095909	1.476625
_y_AI						
_cons	.6576622	.5867744	1.12	0.262	-.4923946	1.807719
_y_AJ						
_Iindustry_2	.3646238	.7749798	0.47	0.638	-1.154309	1.883556
_cons	-.0888452	.3963268	-0.22	0.823	-.8656316	.6879411
_y_AK						
_cons	.5983156	.4666531	1.28	0.200	-.3163077	1.512939
_y_AL						
_cons	.047707	.2854268	0.17	0.867	-.5117192	.6071332
_y_AM						
_Iindustry_2	-.9874994	.8736556	-1.13	0.258	-2.699833	.7248341
_Iindustry_3	.7610857	.9560764	0.80	0.426	-1.11279	2.634961
_cons	1.124256	.4633334	2.43	0.015	.2161389	2.032372
_y_AN						
_Iindustry_3	-.255999	.7143424	-0.36	0.720	-1.656084	1.144086
_cons	.6081003	.1604089	3.79	0.000	.2937047	.922496
_y_AO						
_Iindustry_2	.0672318	.5863538	0.11	0.909	-1.082001	1.216464
_cons	1.016066	.2601869	3.91	0.000	.5061089	1.526023
_y_AP						
_cons	.1166809	.5446089	0.21	0.830	-.9507329	1.184095
_y_AQ						
_cons	.6915604	.6634507	1.04	0.297	-.6087791	1.9919
_y_AR						
_cons	.9348108	.6258281	1.49	0.135	-.2917897	2.161411
_y_AS						
_Iindustry_2	-3.97e-06	.6927984	-0.00	1.000	-1.357864	1.357856
_Iindustry_3	.17149	.6631179	0.26	0.796	-1.128197	1.471177
_cons	.5178141	.4802244	1.08	0.281	-.4234084	1.459037
_y_AT						
_cons	.1791635	.5951264	0.30	0.763	-.9872627	1.34559
_y_AU						
_cons	1.250205	.4644996	2.69	0.007	.3398027	2.160608
_y_AV						
_Iindustry_3	-.2291999	1.024166	-0.22	0.823	-2.236528	1.778128
_cons	.6397128	.7118402	0.90	0.369	-.7554682	2.034894
_y_AW						
_cons	.8483357	.6965451	1.22	0.223	-.5168676	2.213539
_y_AX						
_cons	3.589779	.7373089	4.87	0.000	2.14468	5.034878
_y_AY						
_cons	.1750358	.7018214	0.25	0.803	-1.200509	1.55058
_y_AZ						
_cons	.9701601	.7029191	1.38	0.168	-.407536	2.347856
_y_BA						
_cons	.6973591	.4810024	1.45	0.147	-.2453883	1.640107
_y_BB						
_cons	.886538	.6972698	1.27	0.204	-.4800857	2.253162
_y_BC						
_cons	1.322453	.6512137	2.03	0.042	.0460979	2.598809
_y_BD						
_cons	1.049452	.6596077	1.59	0.112	-.2433549	2.34226
_y_BF						
_cons	.6603466	.5277386	1.25	0.211	-.374002	1.694695
_y_BG						
_Iindustry_2	.0699099	.6379494	0.11	0.913	-1.180448	1.320268
_cons	.3147972	.4513871	0.70	0.486	-.5699053	1.1995
_y_BH						
_cons	1.160941	.2837781	4.09	0.000	.6047463	1.717136
_y_BI						
_cons	-.1113434	.6277329	-0.18	0.859	-1.341677	1.11899
_y_BJ						
_cons	.8689303	.6260003	1.39	0.165	-.3580077	2.095868
_y_BK						
_Iindustry_2	1.832456	.8711252	2.10	0.035	.1250817	3.53983
_cons	.1077459	.5750967	0.19	0.851	-1.019423	1.234915
_y_BL						
_Iindustry_2	.0074779	.4533597	0.02	0.987	-.8810908	.8960466
_Iindustry_3	.3241839	.4950231	0.65	0.513	-.6460437	1.294411
_cons	.4182693	.3252736	1.29	0.198	-.2192551	1.055794
_y_BM						
_Iindustry_2	-.7632457	.9059535	-0.84	0.400	-2.538882	1.01239
_Iindustry_3	.0601733	.978691	0.06	0.951	-1.858026	1.978373
_cons	1.221388	.6456056	1.89	0.059	-.0439752	2.486752
_y_BN						
_cons	.4804373	.6117403	0.79	0.432	-.7185517	1.679426
_y_BO						
_cons	.3336716	.6625959	0.50	0.615	-.9649925	1.632336
_y_BP						
_cons	.2845211	.6273058	0.45	0.650	-.9449758	1.514018
_y_BQ						
_Iindustry_2	-.137602	.3073317	-0.45	0.654	-.7399611	.4647571
_Iindustry_3	.0668512	.3712659	0.18	0.857	-.6608165	.7945189
_cons	.4730199	.2448522	1.93	0.053	-.0068816	.9529213
_y_BR						

_y_BQ						
_Iindustry_2	-.137602	.3073317	-0.45	0.654	-.7399611	.4647571
_Iindustry_3	.0668512	.3712659	0.18	0.857	-.6608165	.7945189
_cons	.4730199	.2448522	1.93	0.053	-.0068816	.9529213
_y_BR						
_cons	.5903182	.6767783	0.87	0.383	-.7361429	1.916779
_y_BS						
_cons	.3658554	.6886738	0.53	0.595	-.9839205	1.715631
_y_BT						
_cons	.6892517	.2027817	3.40	0.001	.2918068	1.086697
_y_BU						
_cons	3.730174	.7785924	4.79	0.000	2.204161	5.256187
_y_BV						
_Iindustry_3	-.4831042	1.042076	-0.46	0.643	-2.525536	1.559328
_cons	1.440896	.7150577	2.02	0.044	.0394089	2.842384
_y_BW						
_cons	.4575189	.6516014	0.70	0.483	-.8195964	1.734634
_y_BX						
_cons	.6842144	.6608681	1.04	0.301	-.6110632	1.979492
_y_BY						
_cons	2.008789	.6555239	3.06	0.002	.7239853	3.293592
_y_BZ						
_cons	-.8246568	.7986098	-1.03	0.302	-2.389903	.7405896
_y_CA						
_cons	.1425095	.5445808	0.26	0.794	-.9248492	1.209868
_y_CB						
_cons	.4390526	.6715807	0.65	0.513	-.8772215	1.755327
_y_CC						
_Iindustry_2	-.0660805	.7009414	-0.09	0.925	-1.4399	1.307739
_Iindustry_3	.3908427	.5046234	0.77	0.439	-.598201	1.379886
_cons	.6006568	.2817009	2.13	0.033	.0485332	1.15278
_y_CD						
_cons	.9443735	.3336049	2.83	0.005	.29052	1.598227
_y_CE						
_cons	.1836219	.5687052	0.32	0.747	-.9310198	1.298264
_y_CF						
_cons	1.076028	.6490913	1.66	0.097	-.1961672	2.348224
_y_CG						
_cons	1.556074	.5405902	2.88	0.004	.4965367	2.615611
_y_CH						
_cons	1.179162	.6487784	1.82	0.069	-.0924203	2.450744

18.3. Meta-regression : Covariate as with or without comorbidity

initial: log likelihood = -766.29998
rescale: log likelihood = -766.29998
rescale eq: log likelihood = -745.90809
Iteration 0: log likelihood = -745.90809
Iteration 1: log likelihood = -744.61448
Iteration 2: log likelihood = -744.60498
Iteration 3: log likelihood = -744.60497

Multivariate meta-analysis
Variance-covariance matrix = proportional .5*I(56)+.5*J(56,56,1)
Method = reml Number of dimensions
= 56
Restricted log likelihood = -744.60497 Number of observations = 169

	Coefficient	Std. err.	z	P>|z|	[95% conf.	interval]
_y_AA						
_Icomorbidi_2	-.1840814	.4772127	-0.39	0.700	-1.119401	.7512382
_Icomorbidi_3	-.2851385	.3956527	-0.72	0.471	-1.060603	.4903264
_cons	.5389978	.1453154	3.71	0.000	.2541849	.8238107
_y_AB						
_cons	1.807873	.7627482	2.37	0.018	.3129136	3.302832
_y_AC						
_cons	1.099987	.46538	2.36	0.018	.1878593	2.012115
_y_AD						
_cons	1.577494	.7565598	2.09	0.037	.0946636	3.060323
_y_AE						
_cons	1.011032	.6151643	1.64	0.100	-.1946681	2.216732
_y_AF						
_cons	1.128599	.5116014	2.21	0.027	.1258785	2.131319
_y_AG						
_Icomorbidi_2	-.0598412	.8514569	-0.07	0.944	-1.728666	1.608984
_cons	.6674913	.6002775	1.11	0.266	-.509031	1.844014
_y_AH						
_cons	1.069524	.2746505	3.89	0.000	.5312189	1.607829
_y_AI						
_cons	.7951251	.5649306	1.41	0.159	-.3121184	1.902369
_y_AJ						
_Icomorbidi_3	-.0692189	.7243373	-0.10	0.924	-1.488894	1.350456
_cons	.0249614	.4166472	0.06	0.952	-.791652	.8415749
_y_AK						
_cons	.7136686	.4470822	1.60	0.110	-.1625964	1.589934
_y_AL						
_Icomorbidi_2	.2005338	.7655982	0.26	0.793	-1.300011	1.701079
_Icomorbidi_3	.0652516	.6882542	0.09	0.924	-1.283702	1.414205
_cons	.0208045	.3506895	0.06	0.953	-.6665343	.7081432
_y_AM						
_Icomorbidi_2	-1.256876	.8490333	-1.48	0.139	-2.920951	.407199
_cons	1.44111	.4173108	3.45	0.001	.6231962	2.259024
_y_AN						
_Icomorbidi_2	.471278	.3542981	1.33	0.183	-.2231336	1.165689
_Icomorbidi_3	-.1935137	.5058272	-0.38	0.702	-1.184917	.7978893
_cons	.4524012	.210645	2.15	0.032	.0395446	.8652577
_y_AO						
_Icomorbidi_2	-.8556305	.5772888	-1.48	0.138	-1.987096	.2758347
_cons	1.369168	.2708019	5.06	0.000	.838406	1.89993
_y_AP						
_cons	.0390078	.5380173	0.07	0.942	-1.015487	1.093502
_y_AQ						
_cons	.6915604	.6515124	1.06	0.288	-.5853805	1.968501
_y_AR						
_cons	.9348108	.6131577	1.52	0.127	-.2669563	2.136578
_y_AS						
_Icomorbidi_3	-.2315405	.7758182	-0.30	0.765	-1.752116	1.289035
_cons	.6789452	.2775161	2.45	0.014	.1350237	1.222867
_y_AT						
_cons	.1791635	.5817878	0.31	0.758	-.9611196	1.319447
_y_AU						
_Icomorbidi_2	.5101807	.969798	0.53	0.599	-1.390588	2.41095
_cons	.9587387	.6028463	1.59	0.112	-.2228184	2.140296
_y_AV						
_cons	.5903237	.4917493	1.20	0.230	-.3734872	1.554135
_y_AW						
_cons	.2175633	.6840979	0.32	0.750	-1.123244	1.55837
_y_AX						
_cons	1.012688	.685224	1.48	0.139	-.3303268	2.355702
_y_AY						
_Icomorbidi_2	.0437086	1.042613	0.04	0.967	-1.999775	2.087193
_cons	.6146245	.6257459	0.98	0.326	-.611815	1.841064
_y_AZ						
_cons	.9290655	.6794276	1.37	0.171	-.4025881	2.260719
_y_BA						
_cons	1.364981	.6320727	2.16	0.031	.1261411	2.60382
_y_BB						
_cons	1.09198	.6407175	1.70	0.088	-.1638033	2.347763
_y_BD						
_cons	1.280166	.7438796	1.72	0.085	-.1778107	2.738144
_y_BE						
_Icomorbidi_2	.7002006	.7418147	0.94	0.345	-.7537295	2.154131
_cons	.1868806	.3566681	0.52	0.600	-.512176	.8859371

_y_BF						
_Icomorbidi_3	-.219459	.8215866	-0.27	0.789	-1.829739	1.390821
_cons	1.251626	.344518	3.63	0.000	.5763834	1.926869
_y_BG						
_cons	-.1113434	.6151017	-0.18	0.856	-1.316921	1.094234
_y_BH						
_cons	.9082737	.423273	2.15	0.032	.0786739	1.737873
_y_BI						
_Icomorbidi_3	.0179983	.4878529	0.04	0.971	-.9381758	.9741725
_cons	.4652053	.2182396	2.13	0.033	.0374635	.892947
_y_BJ						
_cons	1.036472	.3804318	2.72	0.006	.2908396	1.782105
_y_BK						
_cons	.4804373	.5987719	0.80	0.422	-.6931341	1.654009
_y_BL						
_cons	.4438044	.6213472	0.71	0.475	-.7740138	1.661623
_y_BM						
_cons	.3270485	.6074119	0.54	0.590	-.863457	1.517554
_y_BN						
_Icomorbidi_2	1.997606	.645681	3.09	0.002	.732095	3.263118
_Icomorbidi_3	-.3817365	.3682848	-1.04	0.300	-1.103561	.3400884
_cons	.4561978	.1394206	3.27	0.001	.1829384	.7294572
_y_BO						
_cons	.6328457	.658381	0.96	0.336	-.6575573	1.923249
_y_BP						
_cons	.3658554	.6771803	0.54	0.589	-.9613937	1.693104
_y_BQ						
_Icomorbidi_2	.0857265	.4261843	0.20	0.841	-.7495794	.9210324
_Icomorbidi_3	-.0428527	.6814753	-0.06	0.950	-1.37852	1.292814
_cons	.6292983	.2675507	2.35	0.019	.1049085	1.153688
_y_BR						
_cons	3.772702	.7626551	4.95	0.000	2.277925	5.267478
_y_BS						
_Icomorbidi_3	-.7153624	1.038238	-0.69	0.491	-2.750271	1.319546
_cons	1.483424	.6976705	2.13	0.033	.1160146	2.850833
_y_BT						
_cons	.2149085	.7170883	0.30	0.764	-1.190559	1.620376
_y_BU						
_cons	.7267419	.642015	1.13	0.258	-.5315844	1.985068
_y_BV						
_cons	2.051316	.6365126	3.22	0.001	.8037744	3.298858
_y_BW						
_cons	-.7145241	.7647331	-0.93	0.350	-2.213373	.7843252
_y_BX						
_cons	.064812	.5379918	0.12	0.904	-.9896324	1.119257
_y_BY						
_cons	.4815801	.653037	0.74	0.461	-.7983489	1.761509
_y_BZ						
_Icomorbidi_2	.25632	.4403768	0.58	0.561	-.6068027	1.119443
_cons	.6000589	.2760499	2.17	0.030	.0590111	1.141107
_y_CA						
_cons	.9863934	.3151197	3.13	0.002	.3687701	1.604017
_y_CB						
_cons	.1833456	.5575518	0.33	0.742	-.9094358	1.276127
_y_CC						
_cons	1.24225	.5755903	2.16	0.031	.114114	2.370387
_y_CD						
_cons	1.311746	.6237203	2.10	0.035	.0892764	2.534215
_y_CE						
_cons	1.345627	.5750489	2.34	0.019	.2185517	2.472702

18.3 Meta-regression : Covariate as risk of bias

Note: 179 observations on 59 variables

Note: variance-covariance matrix is proportional to .5*I(59)+.5*J(59,59,1)

initial: log likelihood = -829.16495

rescale: log likelihood = -829.16495

rescale eq: log likelihood = -803.04751

Iteration 0: log likelihood = -803.04751 (not concave)

Iteration 1: log likelihood = -802.61802 (not concave)

Iteration 2: log likelihood = -802.60494 (not concave)

Iteration 3: log likelihood = -802.59622

Iteration 4: log likelihood = -802.59314

Iteration 5: log likelihood = -802.59314

Multivariate meta-analysis

Variance-covariance matrix = proportional .5*I(59)+.5*J(59,59,1)

Method = reml Number of dimensions

= 59

Restricted log likelihood = -802.59314 Number of observations = 179

Multivariate meta-analysis
Variance-covariance matrix = proportional .5*I(59)+.5*J(59,59,1)
Method = reml Number of dimensions
= 59
Restricted log likelihood = -802.59314 Number of observations = 179

	Coefficient	Std. err.	z	P>|z|	[95% conf. interval]	
_y_AA						
_Irob_2	.7459764	98.68901	0.01	0.994	-192.6809	194.1729
_Irob_3	.4741183	98.68922	0.00	0.996	-192.9532	193.9014
_cons	-.2620197	98.68892	-0.00	0.998	-193.6888	193.1647
_y_AB						
_cons	1.766112	.6863314	2.57	0.010	.4209272	3.111297
_y_AC						
_Irob_2	-.5702989	.9018359	-0.63	0.527	-2.337865	1.197267
_cons	1.397321	.680496	2.05	0.040	.0635739	2.731069
_y_AD						
_cons	1.535733	.6794473	2.26	0.024	.2040407	2.867425
_y_AE						
_cons	.2100157	98.69041	0.00	0.998	-193.2196	193.6397
_y_AF						
_Irob_2	-1.066434	.9920978	-1.07	0.282	-3.01091	.8780422
_cons	1.403384	.679554	2.07	0.039	.0714822	2.735285
_y_AG						
_cons	.5587422	.408821	1.37	0.172	-.2425322	1.360017
_y_AH						
_Irob_2	-.5780887	98.69078	-0.01	0.995	-194.0085	192.8523
_cons	1.397662	98.69041	0.01	0.989	-192.032	194.8273
_y_AI						
_cons	.6396605	.541522	1.18	0.238	-.421703	1.701024
_y_AJ						
_Irob_2	-.3669767	.7295278	-0.50	0.615	-1.796825	1.062871
_cons	.2757786	.6289422	0.44	0.661	-.9569255	1.508483
_y_AK						
_cons	.5734127	.4248229	1.35	0.177	-.2592248	1.40605
_y_AL						
_cons	.0430552	.2677066	0.16	0.872	-.48164	.5677504
_y_AM						
_cons	1.046878	.3420749	3.06	0.002	.3764239	1.717333
_y_AN						
_Irob_2	.4135077	.3218996	1.28	0.199	-.2174039	1.044419
_cons	.2977562	.2703029	1.10	0.271	-.2320278	.8275402
_y_AO						
_cons	1.019449	.220137	4.63	0.000	.587988	1.450909
_y_AP						
_cons	.1681312	.5117119	0.33	0.742	-.8348057	1.171068
_y_AQ						
_cons	.6915604	.6262719	1.10	0.269	-.53591	1.919031
_y_AR						
_cons	.9348108	.5862683	1.59	0.111	-.2142539	2.083876
_y_AS						
_Irob_2	.4676	98.6902	0.00	0.996	-192.9616	193.8968
_Irob_3	.2154427	98.69077	0.00	0.998	-193.2149	193.6458
_cons	.0827682	98.68962	0.00	0.999	-193.3453	193.5109
_y_AT						
_cons	.1791635	.5533761	0.32	0.746	-.9054337	1.263761
_y_AU						
_Irob_3	1.168379	98.69236	0.01	0.991	-192.2651	194.6018
_cons	.1577226	98.69034	0.00	0.999	-193.2718	193.5872
_y_AV						
_cons	.5349873	.4732727	1.13	0.258	-.3926102	1.462585
_y_AW						

	Coefficient	Std. err.	z	P>|z|	[95% conf. interval]	
_y_AA						
_Irob_2	.7459764	98.68901	0.01	0.994	-192.6809	194.1729
_Irob_3	.4741183	98.68922	0.00	0.996	-192.9532	193.9014
_cons	-.2620197	98.68892	-0.00	0.998	-193.6888	193.1647
_y_AB						
_cons	1.766112	.6863314	2.57	0.010	.4209272	3.111297
_y_AC						
_Irob_2	-.5702989	.9018359	-0.63	0.527	-2.337865	1.197267
_cons	1.397321	.680496	2.05	0.040	.0635739	2.731069
_y_AD						
_cons	1.535733	.6794473	2.26	0.024	.2040407	2.867425
_y_AE						
_cons	.2100157	98.69041	0.00	0.998	-193.2196	193.6397
_y_AF						
_Irob_2	-1.066434	.9920978	-1.07	0.282	-3.01091	.8780422
_cons	1.403384	.679554	2.07	0.039	.0714822	2.735285
_y_AG						
_cons	.5587422	.408821	1.37	0.172	-.2425322	1.360017
_y_AH						
_Irob_2	-.5780887	98.69078	-0.01	0.995	-194.0085	192.8523
_cons	1.397662	98.69041	0.01	0.989	-192.032	194.8273
_y_AI						
_cons	.6396605	.541522	1.18	0.238	-.421703	1.701024
_y_AJ						
_Irob_2	-.3669767	.7295278	-0.50	0.615	-1.796825	1.062871
_cons	.2757786	.6289422	0.44	0.661	-.9569255	1.508483
_y_AK						
_cons	.5734127	.4248229	1.35	0.177	-.2592248	1.40605
_y_AL						
_cons	.0430552	.2677066	0.16	0.872	-.48164	.5677504
_y_AM						
_cons	1.046878	.3420749	3.06	0.002	.3764239	1.717333
_y_AN						
_Irob_2	.4135077	.3218996	1.28	0.199	-.2174039	1.044419
_cons	.2977562	.2703029	1.10	0.271	-.2320278	.8275402
_y_AO						
_cons	1.019449	.220137	4.63	0.000	.587988	1.450909
_y_AP						
_cons	.1681312	.5117119	0.33	0.742	-.8348057	1.171068
_y_AQ						
_cons	.6915604	.6262719	1.10	0.269	-.53591	1.919031
_y_AR						
_cons	.9348108	.5862683	1.59	0.111	-.2142539	2.083876
_y_AS						
_Irob_2	.4676	98.6902	0.00	0.996	-192.9616	193.8968
_Irob_3	.2154427	98.69077	0.00	0.998	-193.2149	193.6458
_cons	.0827682	98.68962	0.00	0.999	-193.3453	193.5109
_y_AT						
_cons	.1791635	.5533761	0.32	0.746	-.9054337	1.263761
_y_AU						
_Irob_3	1.168379	98.69236	0.01	0.991	-192.2651	194.6018
_cons	.1577226	98.69034	0.00	0.999	-193.2718	193.5872
_y_AV						
_cons	.5349873	.4732727	1.13	0.258	-.3926102	1.462585
_y_AW						

18.4 Meta-regression : Covariate as study length

Note: 179 observations on 59 variables

Note: variance-covariance matrix is proportional to .5*I(59)+.5*J(59,59,1)

initial: log likelihood = -875.62123

rescale: log likelihood = -875.62123

rescale eq: log likelihood = -851.14212

Iteration 0: log likelihood = -851.14212

Iteration 1: log likelihood = -850.47816

Iteration 2: log likelihood = -850.47752

Iteration 3: log likelihood = -850.47752

Multivariate meta-analysis

Variance-covariance matrix = proportional .5*I(59)+.5*J(59,59,1)

Method = reml Number of dimensions = 59

Restricted log likelihood = -850.47752 Number of observations = 179

	Coefficient	Std. err.	z	P>|z|	[95% conf.	interval]
_y_AA						
length	-.0173942	.0257891	-0.67	0.500	-.0679398	.0331515
_cons	.615767	.2652085	2.32	0.020	.0959679	1.135566
_y_AB						
_cons	2.100203	.6766688	3.10	0.002	.7739566	3.42645
_y_AC						
length	-.0475527	.0549524	-0.87	0.387	-.1552573	.060152
_cons	1.682637	.9482825	1.77	0.076	-.1759622	3.541237
_y_AD						
_cons	1.800248	.657147	2.74	0.006	.5122631	3.088232
_y_AE						
_cons	.9834362	.5981129	1.64	0.100	-.1888436	2.155716
_y_AF						
length	.3171787	.2425256	1.31	0.191	-.1581628	.7925202
_cons	-.8695315	1.59485	-0.55	0.586	-3.995379	2.256316
_y_AG						
length	-.031423	.4165815	-0.08	0.940	-.8479078	.7850618
_cons	.859034	2.949766	0.29	0.771	-4.922402	6.64047
_y_AH						
length	-.0483391	.0858867	-0.56	0.574	-.216674	.1199958
_cons	1.392635	.7377664	1.89	0.059	-.0533611	2.83863
_y_AI						
_cons	.59883	.5758331	1.04	0.298	-.5297822	1.727442
_y_AJ						
length	-.1831882	.3702178	-0.49	0.621	-.9088017	.5424253
_cons	1.374908	2.789602	0.49	0.622	-4.092611	6.842427
_y_AK						
_cons	.4457601	.4358067	1.02	0.306	-.4084052	1.299925
_y_AL						
_cons	.0442416	.2719727	0.16	0.871	-.488815	.5772983
_y_AM						
length	-.1111499	.1117991	-0.99	0.320	-.3302721	.1079723
_cons	2.112656	1.157352	1.83	0.068	-.1557125	4.381024
_y_AN						
length	-.0019307	.0510934	-0.04	0.970	-.102072	.0982105
_cons	.6005156	.4883152	1.23	0.219	-.3565645	1.557596
_y_AO						
length	-.2046708	.0655885	-3.12	0.002	-.3332218	-.0761197
_cons	3.13928	.7173253	4.38	0.000	1.733348	4.545211
_y_AP						
_cons	.1061543	.5206397	0.20	0.838	-.9142808	1.126589
_y_AQ						
_cons	.6915604	.635184	1.09	0.276	-.5533774	1.936498
_y_AR						
_cons	.9348108	.5957791	1.57	0.117	-.2328948	2.102516
_y_AS						
length	-.0160487	.0295145	-0.54	0.587	-.073896	.0417986
_cons	.7439405	.3912522	1.90	0.057	-.0228997	1.510781
_y_AT						
_cons	.1791635	.5634424	0.32	0.750	-.9251633	1.28349
_y_AU						
_cons	1.228911	.427994	2.87	0.004	.3900582	2.067764
_y_AV						
_cons	.5973289	.4925321	1.21	0.225	-.3680163	1.562674
_y_AW						
_cons	.8284793	.6589885	1.26	0.209	-.4631144	2.120073
_y_AX						
_cons	3.604711	.7059554	5.11	0.000	2.221064	4.988358
_y_AY						
_cons	.2247559	.676964	0.33	0.740	-1.102069	1.551581
_y_AZ						
_cons	1.01988	.678102	1.50	0.133	-.3091754	2.348936
_y_BA						
length	.0239573	.2316992	0.10	0.918	-.4301647	.4780793
_cons	.4229663	2.299685	0.18	0.854	-4.084333	4.930265
_y_BB						
_cons	.8666816	.6597544	1.31	0.189	-.4264134	2.159777
_y_BC						
_cons	1.337385	.6154899	2.17	0.030	.1310471	2.543723
_y_BD						

_y_BD						
_cons	1.064384	.6243644	1.70	0.088	-.1593474	2.288116
_y_BF						
length	.0486308	.0655365	0.74	0.458	-.0798183	.1770799
_cons	-.2276627	1.067111	-0.21	0.831	-2.319163	1.863837
_y_BG						
length	.1753142	.1806669	0.97	0.332	-.1787865	.5294149
_cons	-1.216689	1.640091	-0.74	0.458	-4.431209	1.997831
_y_BH						
length	-.0121734	.1416868	-0.09	0.932	-.2898744	.2655276
_cons	1.238439	1.141939	1.08	0.278	-.9997213	3.476598
_y_BI						
_cons	-.1113434	.5977796	-0.19	0.852	-1.28297	1.060283
_y_BJ						
_cons	.8490739	.5839239	1.45	0.146	-.2953958	1.993544
_y_BK						
length	-.3128934	.1386657	-2.26	0.024	-.5846732	-.0411136
_cons	3.817562	1.366184	2.79	0.005	1.13989	6.495234
_y_BL						
length	-.0562208	.0493824	-1.14	0.255	-.1530086	.040567
_cons	.9290712	.4136262	2.25	0.025	.1183788	1.739764
_y_BM						
length	-.1380573	.1897237	-0.73	0.467	-.5099089	.2337943
_cons	1.695064	1.006976	1.68	0.092	-.278573	3.668702
_y_BN						
_cons	.4804373	.5809631	0.83	0.408	-.6582295	1.619104
_y_BO						
_cons	.3814205	.599772	0.64	0.525	-.794111	1.556952
_y_BP						
_cons	.2646646	.5853233	0.45	0.651	-.8825479	1.411877
_y_BQ						
length	-.0225072	.0263434	-0.85	0.393	-.0741393	.0291249
_cons	.6316542	.2516257	2.51	0.012	.1384769	1.124831
_y_BR						
_cons	.6052501	.6424776	0.94	0.346	-.6539829	1.864483
_y_BS						
_cons	.3658554	.6614859	0.55	0.580	-.9306331	1.662344
_y_BT						
length	.0124392	.0819823	0.15	0.879	-.1482431	.1731214
_cons	.5879175	.6552038	0.90	0.370	-.6962583	1.872093
_y_BU						
_cons	3.779894	.7562626	5.00	0.000	2.297647	5.262142
_y_BV						
length	.0686508	.189315	0.36	0.717	-.3023998	.4397014
_cons	.8718336	1.159044	0.75	0.452	-1.39985	3.143517
_y_BW						
_cons	.4376625	.6112898	0.72	0.474	-.7604434	1.635768
_y_BX						
_cons	.5947815	.6248867	0.95	0.341	-.6299739	1.819537
_y_BY						
_cons	2.023721	.6200486	3.26	0.001	.8084477	3.238993
_y_BZ						
_cons	-.7073316	.7583582	-0.93	0.351	-2.193686	.7790231
_y_CA						
_cons	.1319797	.520612	0.25	0.800	-.8884011	1.152361
_y_CB						
_cons	.4191961	.6325436	0.66	0.508	-.8205665	1.658959
_y_CC						
length	.0243187	.0138142	1.76	0.078	-.0027568	.0513941
_cons	.3292968	.2970955	1.11	0.268	-.2529997	.9115933
_y_CD						
length	-.1959961	.1413579	-1.39	0.166	-.4730526	.0810603
_cons	2.922473	1.496472	1.95	0.051	-.0105581	5.855505
_y_CE						
_cons	.1454192	.5380841	0.27	0.787	-.9092062	1.200045
_y_CF						
_cons	1.120812	.5443932	2.06	0.040	.0538205	2.187803
_y_CG						
_cons	1.462464	.5127603	2.85	0.004	.4574726	2.467456
_y_CH						
_cons	1.224014	.5437702	2.25	0.024	.1582441	2.289784

18.5 Meta-regression : Covariate as baseline depressive severity

Note: regressing _y_CH on _Iseverity_2 _Iseverity_3
Warning: Collinearity detected: now regressing _y_CH on (nothing)
Note: 179 observations on 59 variables
Note: variance-covariance matrix is proportional to .5*I(59)+.5*J(59,59,1)

initial: log likelihood = -825.97324
rescale: log likelihood = -825.97324
rescale eq: log likelihood = -804.84755
Iteration 0: log likelihood = -804.84755
Iteration 1: log likelihood = -804.00553
Iteration 2: log likelihood = -804.00293
Iteration 3: log likelihood = -804.00293

Multivariate meta-analysis
Variance-covariance matrix = proportional .5*I(59)+.5*J(59,59,1)
Method = reml Number of dimensions
= 59
Restricted log likelihood = -804.00293 Number of observations = 179

	Coefficient	Std. err.	z	P>|z|	[95% conf. interval]	
_y_AA						
_Iseverity_2	-.4738725	.314071	-1.51	0.131	-1.08944	.1416953
_Iseverity_3	.4907538	.5238588	0.94	0.349	-.5359906	1.517498
_cons	.7959271	.2754074	2.89	0.004	.2561386	1.335716
_y_AB						
_cons	1.876068	.6754328	2.78	0.005	.5522439	3.199892
_y_AC						
_Iseverity_2	-.7322007	.9246868	-0.79	0.428	-2.544554	1.080152
_cons	1.397321	.6944584	2.01	0.044	.0362079	2.758435
_y_AD						
_cons	1.645689	.6684365	2.46	0.014	.3355773	2.9558
_y_AE						
_cons	1.758714	.7367913	2.39	0.017	.3146293	3.202798
_y_AF						
_Iseverity_2	-2.302915	1.080657	-2.13	0.033	-4.420963	-.1848675
_cons	2.477964	.789156	3.14	0.002	.9312464	4.024681
_y_AG						
_Iseverity_2	-.0770388	.844513	-0.09	0.927	-1.732254	1.578176
_cons	.6076501	.5929516	1.02	0.305	-.5545136	1.769814
_y_AH						
_Iseverity_2	-.1362341	.6062316	-0.22	0.822	-1.324426	1.051958
_Iseverity_3	.3775119	.9244224	0.41	0.683	-1.434323	2.189346
_cons	1.147815	.5033561	2.28	0.023	.1612548	2.134374
_y_AI						
_cons	.6604782	.5665278	1.17	0.244	-.4498958	1.770852
_y_AJ						
_cons	.0892749	.3374382	0.26	0.791	-.5720918	.7506417
_y_AK						
_Iseverity_2	.48383	.8922276	0.54	0.588	-1.264904	2.232564
_cons	.4264143	.6478402	0.66	0.510	-.8433291	1.696158
_y_AL						
_Iseverity_2	.5853062	.5574583	1.05	0.294	-.5072919	1.677904
_cons	-.2946045	.4398997	-0.67	0.503	-1.156792	.567583
_y_AM						
_Iseverity_2	-.4890444	.7315341	-0.67	0.504	-1.922825	.9447361
_cons	1.296822	.5079326	2.55	0.011	.3012925	2.292352
_y_AN						
_Iseverity_2	.130976	.3029671	0.43	0.666	-.4628287	.7247806
_cons	.5270204	.2033449	2.59	0.010	.1284718	.9255691
_y_AO						
_Iseverity_2	-.2701826	.5174854	-0.52	0.602	-1.284435	.7440702
_Iseverity_3	1.072012	.6957044	1.54	0.123	-.2915438	2.435567
_cons	1.141318	.3851583	2.96	0.003	.3864219	1.896215
_y_AP						
_cons	.076235	.5281271	0.14	0.885	-.958875	1.111345
_y_AQ						
_cons	.6915604	.6414158	1.08	0.281	-.5655916	1.948712

_y_AR						
_cons	.9348108	.6024187	1.55	0.121	-.2459081	2.11553
_y_AS						
_Iseverity_2	-.3707505	.6306022	-0.59	0.557	-1.606708	.8652072
_Iseverity_3	.3641176	.8188941	0.44	0.657	-1.240885	1.969121
_cons	.9203579	.5331668	1.73	0.084	-.1246299	1.965346
_y_AT						
_cons	.1791635	.5704585	0.31	0.753	-.9389145	1.297242
_y_AU						
_cons	1.07469	.4406562	2.44	0.015	.2110195	1.93836
_y_AV						
_cons	.3732668	.4867728	0.77	0.443	-.5807904	1.327324
_y_AW						
_cons	.6739205	.6702469	1.01	0.315	-.6397393	1.98758
_y_AX						
_cons	4.379988	.8267376	5.30	0.000	2.759612	6.000364
_y_AY						
_cons	.0006207	.6757286	0.00	0.999	-1.323783	1.325024
_y_AZ						
_cons	.7957449	.6768686	1.18	0.240	-.5308932	2.122383
_y_BA						
_cons	.6194295	.4640087	1.33	0.182	-.2900109	1.52887
_y_BB						
_cons	.7121228	.671	1.06	0.289	-.6030131	2.027259
_y_BC						
_cons	1.62191	.6642245	2.44	0.015	.3200535	2.923766
_y_BD						
_cons	1.839662	.7582567	2.43	0.015	.353506	3.325817
_y_BF						
_Iseverity_2	-1.53027	1.021675	-1.50	0.134	-3.532716	.4721753
_cons	1.537095	.7713851	1.99	0.046	.0252081	3.048982
_y_BG						
_Iseverity_2	-.7741807	.6164826	-1.26	0.209	-1.982464	.4341029
_cons	.7627504	.4513338	1.69	0.091	-.1218476	1.647348
_y_BH						
_Iseverity_2	-.7867713	.5457757	-1.44	0.149	-1.856472	.2829295
_cons	1.578526	.3892658	4.06	0.000	.815579	2.341473
_y_BI						
_cons	-.1113434	.6043972	-0.18	0.854	-1.29594	1.073253
_y_BJ						
_cons	.6945151	.5966005	1.16	0.244	-.4748005	1.863831
_y_BK						
_Iseverity_2	1.682025	.8485982	1.98	0.047	.0188027	3.345247
_cons	.258177	.5663467	0.46	0.648	-.8518422	1.368196
_y_BL						
_Iseverity_2	.0415199	.6710866	0.06	0.951	-1.273786	1.356825
_Iseverity_3	.8626315	.7365757	1.17	0.242	-.5810303	2.306293
_cons	.3232187	.6180545	0.52	0.601	-.8881458	1.534583
_y_BM						

_y_BM						
_Iseverity_2	.2217687	.8795015	0.25	0.801	-1.502022	1.94556
_Iseverity_3	1.299446	1.000183	1.30	0.194	-.6608762	3.259769
_cons	.8252045	.6266251	1.32	0.188	-.402958	2.053367
_y_BN						
_cons	.4804373	.5877701	0.82	0.414	-.6715709	1.632445
_y_BO						
_cons	.2268616	.6121205	0.37	0.711	-.9728726	1.426596
_y_BP						
_cons	.1101057	.5979703	0.18	0.854	-1.061894	1.282106
_y_BQ						
_Iseverity_2	-.0032336	.2794694	-0.01	0.991	-.5509835	.5445164
_Iseverity_3	1.013654	.5161007	1.96	0.050	.0021147	2.025192
_cons	.3600212	.2341128	1.54	0.124	-.0988315	.8188738
_y_BR						
_cons	.415903	.6496805	0.64	0.522	-.8574473	1.689253
_y_BS						
_cons	.3658554	.6674721	0.55	0.584	-.942366	1.674077
_y_BT						
_Iseverity_2	-.0523087	.383182	-0.14	0.891	-.8033317	.6987142
_cons	.731096	.2603044	2.81	0.005	.2209087	1.241283
_y_BU						
_cons	4.520383	.8637573	5.23	0.000	2.82745	6.213316
_y_BV						
_Iseverity_2	-.5343997	1.056758	-0.51	0.613	-2.605608	1.536809
_cons	1.800881	.8008244	2.25	0.025	.2312938	3.370468
_y_BW						
_cons	.7569753	.6646047	1.14	0.255	-.545626	2.059577
_y_BX						
_cons	1.474424	.7593533	1.94	0.052	-.0138815	2.962729
_y_BY						
_cons	1.834373	.6275089	2.92	0.003	.6044785	3.064268
_y_BZ						
_cons	.0331569	.8655926	0.04	0.969	-1.663373	1.729687
_y_CA						
_cons	.1020507	.5281008	0.19	0.847	-.9330079	1.137109
_y_CB						
_cons	.2646373	.6442643	0.41	0.681	-.9980975	1.527372
_y_CC						
_Iseverity_2	.0864422	.6641624	0.13	0.896	-1.215292	1.388177
_Iseverity_3	-.9883838	.6305204	-1.57	0.117	-2.224181	.2474135
_cons	.8213764	.2437065	3.37	0.001	.3437205	1.299032
_y_CD						
_Iseverity_2	-1.525556	.6847963	-2.23	0.026	-2.867732	-.1833795
_Iseverity_3	.5224209	.9341111	0.56	0.576	-1.308403	2.353245
_cons	1.671161	.5129749	3.26	0.001	.6657485	2.676573
_y_CE						
_cons	.3366591	.613895	0.55	0.583	-.8665531	1.539871
_y_CF						
_cons	1.224332	.553158	2.21	0.027	.1401622	2.308502
_y_CG						
_cons	1.378436	.5192336	2.65	0.008	.3607566	2.396115
_y_CH						
_cons	1.327689	.5525545	2.40	0.016	.2447024	2.410676

19. Grading the confidence in evidence with CINeMA

19.1. General

- ✓ We evaluated confidence in the evidence of network meta-analytic estimates of placebo-controlled comparisons using Confidence In Network Meta-Analysis (CINeMA) framework (1) and the online tool (https://cinema.ispm.unibe.ch/)

- ✓ The following abbreviations are used in the figures and tables throughout the documents: ADT: Antidepressant; Ca: Calcium ; DHA: Docosahexaenoic Acid ; E Amoenum : Echium amoenum; EPA :Eicosapentaenoic acid; Fe : Ferrum; Mg: Magnesium; PEA: Palmitoylethanolamide; R rosea : Rhodiola rosea; SAMe: S-Adenosyl Methionine ; SJW: St. John's wort ;Vitamin B1: Thiamine ; Vitamin B6: Pyridoxine; Vitamin B7: Biotin; Vitamin B: Vitamin B complex; Vitamin B12 : Cobalamin; Vitamin C: Ascorbic acid; Vitamin D: Cholecalciferol; 5HTP: 5-Hydroxytryptophan

19.2.A. Change in depressive symptoms

19.2.A-1.Network plot for the network meta-analysis of change in depressive symptom using four different sizing and coloring combinations.

Green, yellow, and red colors refer to low, moderate, and high risk of bias or indirectness

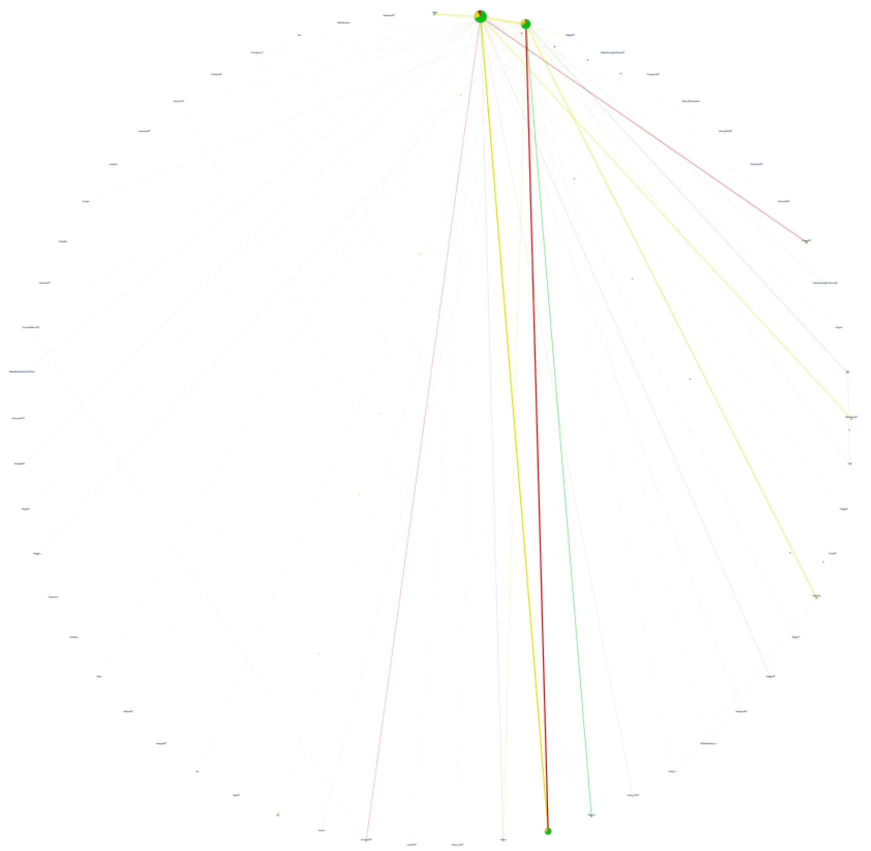

19.2.A-2.Indirectness chart for change in depressive symptom

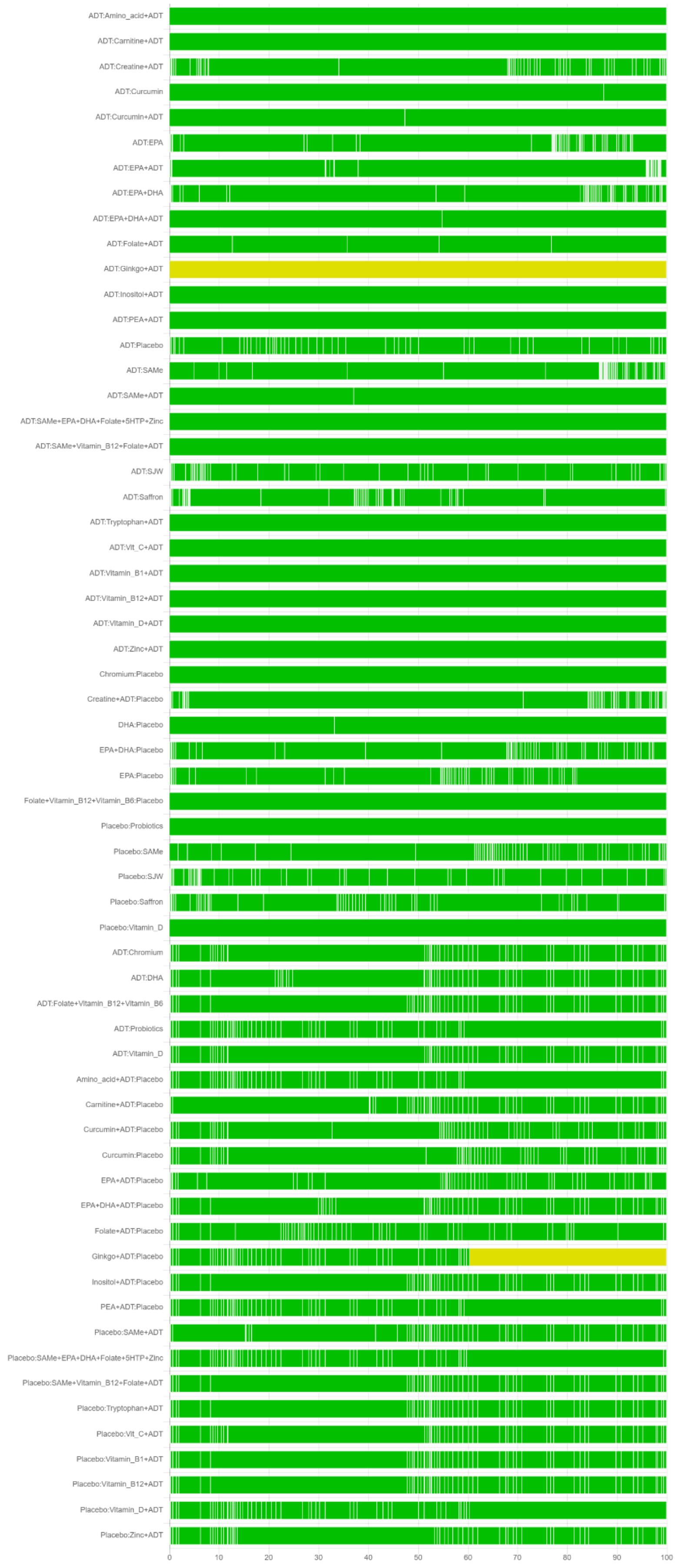

19.2.A-3.Confidence rating in trails for change in depressive symptom

Comparison	Number of studies	Within-study bias	Reporting bias	Indirectness	Imprecision	Heterogeneity	Incoherence	Confidence rating
ADT:Amino_acid+ADT	1	Some concerns	Some concerns	No concerns	No concerns	Some concerns	Major concerns	Very Low
ADT:Carnitine	1	No concerns	Some concerns	No concerns	Some concerns	Some concerns	No concerns	Very Low
ADT:Carnitine+ADT	1	Some concerns	Some concerns	No concerns	No concerns	Some concerns	Major concerns	Low
ADT:Chlorella+ADT	1	Major concerns	Some concerns	Some concerns	Major concerns	No concerns	Major concerns	Very Low
ADT:Creatine+ADT	2	Some concerns	Some concerns	No concerns	Some concerns	Some concerns	Major concerns	Very Low
ADT:Curcumin	1	No concerns	Some concerns	No concerns	Major concerns	No concerns	No concerns	Low
ADT:Curcumin+ADT	7	No concerns	Some concerns	No concerns	No concerns	Major concerns	No concerns	Low
ADT:Curcumin+Saffron+ADT	1	No concerns	Some concerns	No concerns	Major concerns	No concerns	No concerns	Low
ADT:DHA+ADT	2	No concerns	Some concerns	No concerns	Major concerns	No concerns	Major concerns	Low
ADT:EPA+ADT	4	No concerns	Some concerns	No concerns	Some concerns	Some concerns	No concerns	Low
ADT:EPA+DHA	1	No concerns	Some concerns	No concerns	No concerns	Major concerns	Some concerns	Very Low
ADT:EPA+DHA+ADT	11	No concerns	Some concerns	No concerns	No concerns	Some concerns	Some concerns	Low
ADT:Folate+ADT	8	Some concerns	Some concerns	No concerns	Some concerns	Some concerns	Major concerns	Low
ADT:Ginkgo+ADT	2	Major concerns	Some concerns	Some concerns	Some concerns	Some concerns	Major concerns	Very Low
ADT:Inositol+ADT	2	No concerns	Some concerns	No concerns	Major concerns	No concerns	Major concerns	Very Low
ADT:Lavandula	1	Major concerns	Some concerns	Some concerns	Major concerns	No concerns	Major concerns	Very Low
ADT:Lavandula+ADT	1	Major concerns	Some concerns	Some concerns	Major concerns	No concerns	Major concerns	Very Low
ADT:Mg	1	Some concerns	Some concerns	No concerns	Major concerns	No concerns	No concerns	Very Low
ADT:Mg+ADT	1	Some concerns	Some concerns	No concerns	Major concerns	No concerns	Major concerns	Very Low
ADT:Nepta	1	No concerns	Some concerns	No concerns	Some concerns	Some concerns	Major concerns	Very Low
ADT:PEA+ADT	1	No concerns	Some concerns	No concerns	Major concerns	No concerns	Major concerns	Very Low
ADT:Placebo	13	No concerns	Some concerns	No concerns	No concerns	Major concerns	Major concerns	Very Low

ADT:Probiotics+ADT	5	Some concerns	Some concerns	No concerns	Some concerns	Some concerns	Major concerns	Very Low
ADT:R_rosea	1	No concerns	Some concerns	No concerns	Some concerns	Some concerns	No concerns	Very Low
ADT:SAMe	7	No concerns	Some concerns	No concerns	No concerns	Major concerns	No concerns	Low
ADT:SAMe+ADT	3	No concerns	Some concerns	No concerns	Some concerns	Some concerns	Major concerns	Low
ADT:SAMe+EPA+DHA+Folic +5HTP+Zinc+ADT	1	No concerns	Some concerns	No concerns	Major concerns	No concerns	Major concerns	Very Low
ADT:SAMe+Vitamin_B12+Folate+ADT	1	No concerns	Some concerns	No concerns	Major concerns	No concerns	Major concerns	Very Low
ADT:SJW	18	No concerns	Some concerns	No concerns	No concerns	Major concerns	Some concerns	Low
ADT:SJW+ADT	1	No concerns	Some concerns	No concerns	Major concerns	No concerns	Major concerns	Very Low
ADT:Saffron	6	No concerns	Some concerns	No concerns	Some concerns	Some concerns	Major concerns	Low
ADT:Saffron+ADT	1	No concerns	Some concerns	No concerns	No concerns	No concerns	Major concerns	Very Low
ADT:Tryptophan+ADT	2	No concerns	Some concerns	No concerns	Some concerns	Some concerns	Major concerns	Very Low
ADT:Vitamin_B+ADT	1	No concerns	Some concerns	No concerns	Major concerns	No concerns	Major concerns	Very Low
ADT:Vitamin_B1+ADT	1	No concerns	Some concerns	No concerns	Major concerns	No concerns	Major concerns	Very Low
ADT:Vitamin_B12+ADT	1	Major concerns	Some concerns	Some concerns	No concerns	Some concerns	Major concerns	Very Low
ADT:Vitamin_B6+Tryptopohan	1	Major concerns	Some concerns	Some concerns	Some concerns	No concerns	Major concerns	Very Low
ADT:Vitamin_C+ADT	1	No concerns	Some concerns	No concerns	Major concerns	No concerns	Major concerns	Very Low
ADT:Vitamin_D+ADT	4	No concerns	Some concerns	No concerns	Some concerns	Some concerns	Major concerns	Very Low
ADT:Zinc+ADT	2	Some concerns	Some concerns	No concerns	No concerns	Some concerns	Major concerns	Low
Carnitine:Placebo	1	Some concerns	Some concerns	No concerns	No concerns	Some concerns	No concerns	Very Low
Curcumin:Placebo	1	No concerns	Some concerns	No concerns	Some concerns	Some concerns	No concerns	Very Low
DHA:Placebo	3	No concerns	Some concerns	No concerns	Major concerns	No concerns	No concerns	Low
EPA:Placebo	5	No concerns	Some concerns	No concerns	Some concerns	Some concerns	No concerns	Low
EPA+DHA:Placebo	16	No concerns	Some concerns	No concerns	No concerns	Some concerns	No concerns	Moderate
EPA+DHA+ADT:Placebo	1	No concerns	Some concerns	No concerns	No concerns	No concerns	No concerns	Low

EPA+DHA+Vitamin_C:Placebo	1	No concerns	Some concerns	No concerns	Major concerns	No concerns	No concerns	Very Low
E_amoenum:Placebo	1	Major concerns	Some concerns	No concerns	Major concerns	No concerns	Major concerns	Very Low
Fe:Placebo	1	No concerns	Some concerns	No concerns	Some concerns	Some concerns	Major concerns	Very Low
Folate+Vitamin_B12+ Vitamin_B6:Placebo	1	No concerns	Some concerns	No concerns	Major concerns	No concerns	Major concerns	Very Low
Mg:Placebo	1	No concerns	Some concerns	No concerns	Some concerns	Some concerns	No concerns	Very Low
Placebo:Probiotics	4	Some concerns	Some concerns	No concerns	Some concerns	Some concerns	Major concerns	Low
Placebo:Probiotics+B7	1	No concerns	Some concerns	No concerns	Major concerns	No concerns	Major concerns	Very Low
Placebo:R_rosea	2	No concerns	Some concerns	No concerns	No concerns	Some concerns	No concerns	Very Low
Placebo:SAMe	8	No concerns	Some concerns	No concerns	No concerns	Major concerns	No concerns	Low
Placebo:SAMe+Probiotcis	1	No concerns	Some concerns	No concerns	Major concerns	No concerns	Major concerns	Very Low
Placebo:SJW	18	No concerns	Some concerns	No concerns	No concerns	Major concerns	No concerns	Low
Placebo:SJW+Kava	1	No concerns	Some concerns	No concerns	Major concerns	No concerns	Major concerns	Very Low
Placebo:Saffron	6	No concerns	Some concerns	No concerns	No concerns	Some concerns	Some concerns	Very Low
Placebo:Vitamin_C	1	No concerns	Some concerns	No concerns	Major concerns	No concerns	No concerns	Very Low
Placebo:Vitamin_D	9	No concerns	Some concerns	No concerns	No concerns	Some concerns	Major concerns	Very Low
Placebo:Vitamin_D+Ca	1	No concerns	Some concerns	No concerns	Major concerns	No concerns	No concerns	Very Low
Placebo:Zinc	1	No concerns	Some concerns	No concerns	No concerns	Some concerns	No concerns	Very Low
Placebo:Zinc+Vitamin_D	1	No concerns	Some concerns	No concerns	No concerns	Some concerns	No concerns	Very Low
ADT:DHA	0	No concerns	Some concerns	No concerns	Some concerns	Some concerns	Major concerns	Very Low
ADT:EPA	0	No concerns	Some concerns	No concerns	Some concerns	Some concerns	Major concerns	Very Low
ADT:EPA+DHA+Vitamin_C	0	No concerns	Some concerns	No concerns	Major concerns	No concerns	Major concerns	Low
ADT:E_amoenum	0	Some concerns	Some concerns	No concerns	Major concerns	No concerns	Major concerns	Very Low
ADT:Fe	0	No concerns	Some concerns	No concerns	Major concerns	No concerns	Major concerns	Very Low
ADT:Folate+Vitamin_B12+ Vitamin_B6	0	No concerns	Some concerns	No concerns	Major concerns	No concerns	Major concerns	Very Low

ADT:Probiotics	0	No concerns	Some concerns	No concerns	Some concerns	Some concerns	Major concerns	Very Low
ADT:Probiotics+B7	0	No concerns	Some concerns	No concerns	Major concerns	No concerns	Major concerns	Very Low
ADT:SAMe+Probiotcis	0	No concerns	Some concerns	No concerns	Major concerns	No concerns	Major concerns	Very Low
ADT:SJW+Kava	0	No concerns	Some concerns	No concerns	Major concerns	No concerns	Major concerns	Very Low
ADT:Vitamin_C	0	No concerns	Some concerns	No concerns	Major concerns	No concerns	Major concerns	Very Low
ADT:Vitamin_D	0	No concerns	Some concerns	No concerns	Some concerns	Some concerns	Major concerns	Very Low
ADT:Vitamin_D+Ca	0	No concerns	Some concerns	No concerns	Major concerns	No concerns	Major concerns	Very Low
ADT:Zinc	0	No concerns	Some concerns	No concerns	Some concerns	Some concerns	Major concerns	Very Low
ADT:Zinc+Vitamin_D	0	No concerns	Some concerns	No concerns	Some concerns	Some concerns	Major concerns	Very Low
Amino_acid+ADT:Placebo	0	Some concerns	Some concerns	No concerns	No concerns	No concerns	Major concerns	Very Low
Carnitine+ADT:Placebo	0	Some concerns	Some concerns	No concerns	No concerns	No concerns	Major concerns	Low
Chlorella+ADT:Placebo	0	Some concerns	Some concerns	No concerns	Some concerns	Some concerns	Major concerns	Very Low
Creatine+ADT:Placebo	0	No concerns	Some concerns	No concerns	No concerns	Some concerns	Major concerns	Very Low
Curcumin+ADT:Placebo	0	No concerns	Some concerns	No concerns	No concerns	Some concerns	Major concerns	Very Low
Curcumin+Saffron+ADT:Placebo	0	No concerns	Some concerns	No concerns	Some concerns	Some concerns	Major concerns	Very Low
DHA+ADT:Placebo	0	No concerns	Some concerns	No concerns	Some concerns	Some concerns	Major concerns	Very Low
EPA+ADT:Placebo	0	No concerns	Some concerns	No concerns	No concerns	Some concerns	Major concerns	Very Low
Folate+ADT:Placebo	0	No concerns	Some concerns	No concerns	No concerns	Some concerns	Major concerns	Very Low
Ginkgo+ADT:Placebo	0	Some concerns	Some concerns	No concerns	No concerns	Some concerns	Major concerns	Very Low
Inositol+ADT:Placebo	0	No concerns	Some concerns	No concerns	Some concerns	Some concerns	Major concerns	Very Low
Lavandula:Placebo	0	Some concerns	Some concerns	No concerns	Major concerns	No concerns	Major concerns	Very Low
Lavandula+ADT:Placebo	0	Some concerns	Some concerns	No concerns	Some concerns	Some concerns	Major concerns	Very Low
Mg+ADT:Placebo	0	Some concerns	Some concerns	No concerns	Some concerns	Some concerns	Major concerns	Very Low
Nepta:Placebo	0	No concerns	Some concerns	No concerns	No concerns	Some concerns	Major concerns	Very Low

PEA+ADT:Placebo	0	No concerns	Some concerns	No concerns	Some concerns	Some concerns	Major concerns	Very Low
Placebo:Probiotics+ADT	0	No concerns	Some concerns	No concerns	No concerns	Some concerns	Major concerns	Very Low
Placebo:SAMe+ADT	0	No concerns	Some concerns	No concerns	No concerns	Some concerns	Major concerns	Low
Placebo:SAMe+EPA+DHA+Folic+5HTP+Zinc	0	No concerns	Some concerns	No concerns	Major concerns	No concerns	Major concerns	Very Low
Placebo:SAMe+Vitamin_B12+Folate+ADT	0	No concerns	Some concerns	No concerns	Major concerns	No concerns	Major concerns	Very Low
Placebo:SJW+ADT	0	No concerns	Some concerns	No concerns	Major concerns	No concerns	Major concerns	Low
Placebo:Saffron+ADT	0	No concerns	Some concerns	No concerns	No concerns	No concerns	Major concerns	Very Low
Placebo:Tryptophan+ADT	0	No concerns	Some concerns	No concerns	No concerns	Some concerns	Major concerns	Very Low
Placebo:Vitamin_B+ADT	0	No concerns	Some concerns	No concerns	Major concerns	No concerns	Major concerns	Very Low
Placebo:Vitamin_B1+ADT	0	No concerns	Some concerns	No concerns	Some concerns	Some concerns	Major concerns	Very Low
Placebo:Vitamin_B12+ADT	0	Some concerns	Some concerns	No concerns	No concerns	No concerns	Major concerns	Very Low
Placebo:Vitamin_B6+Tryptophan	0	Some concerns	Some concerns	No concerns	Major concerns	No concerns	Major concerns	Very Low
Placebo:Vitamin_C+ADT	0	No concerns	Some concerns	No concerns	Major concerns	No concerns	Major concerns	Very Low
Placebo:Vitamin_D+ADT	0	No concerns	Some concerns	No concerns	No concerns	Some concerns	Major concerns	Very Low
Placebo:Zinc+ADT	0	No concerns	Some concerns	No concerns	No concerns	No concerns	Major concerns	Very Low

19.2.B. Response rate

19.2.B-1.Network plot for the network meta-analysis of response using four different sizing and coloring combinations.

Green, yellow, and red colors refer to low, moderate, and high risk of bias or indirectness

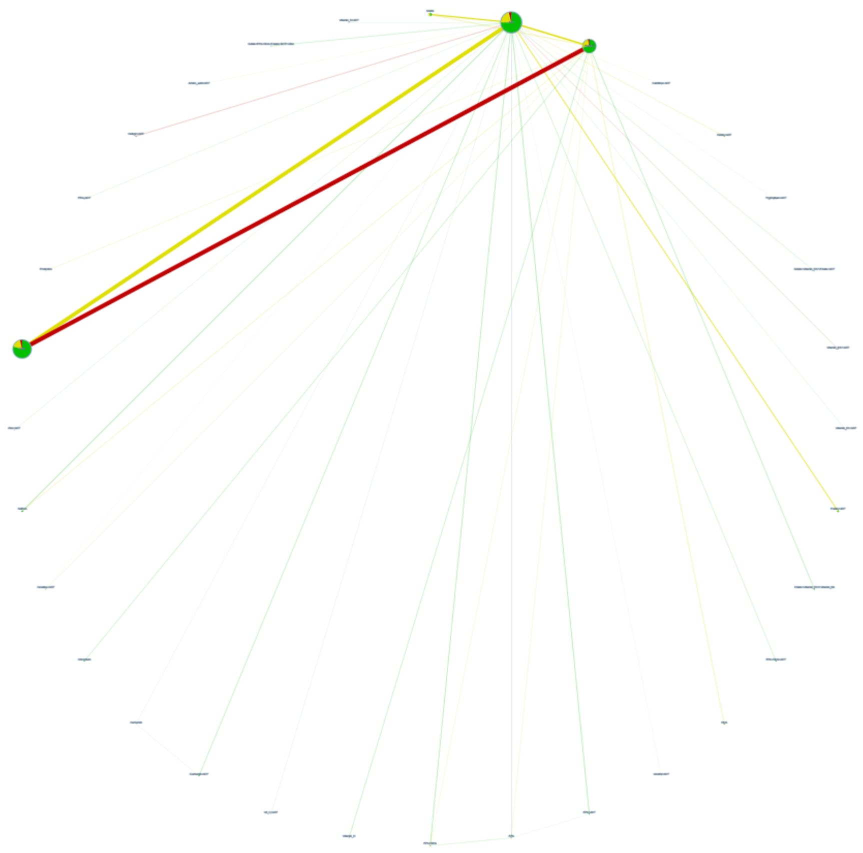

19.2.B-2.Indirectness chart for response rate

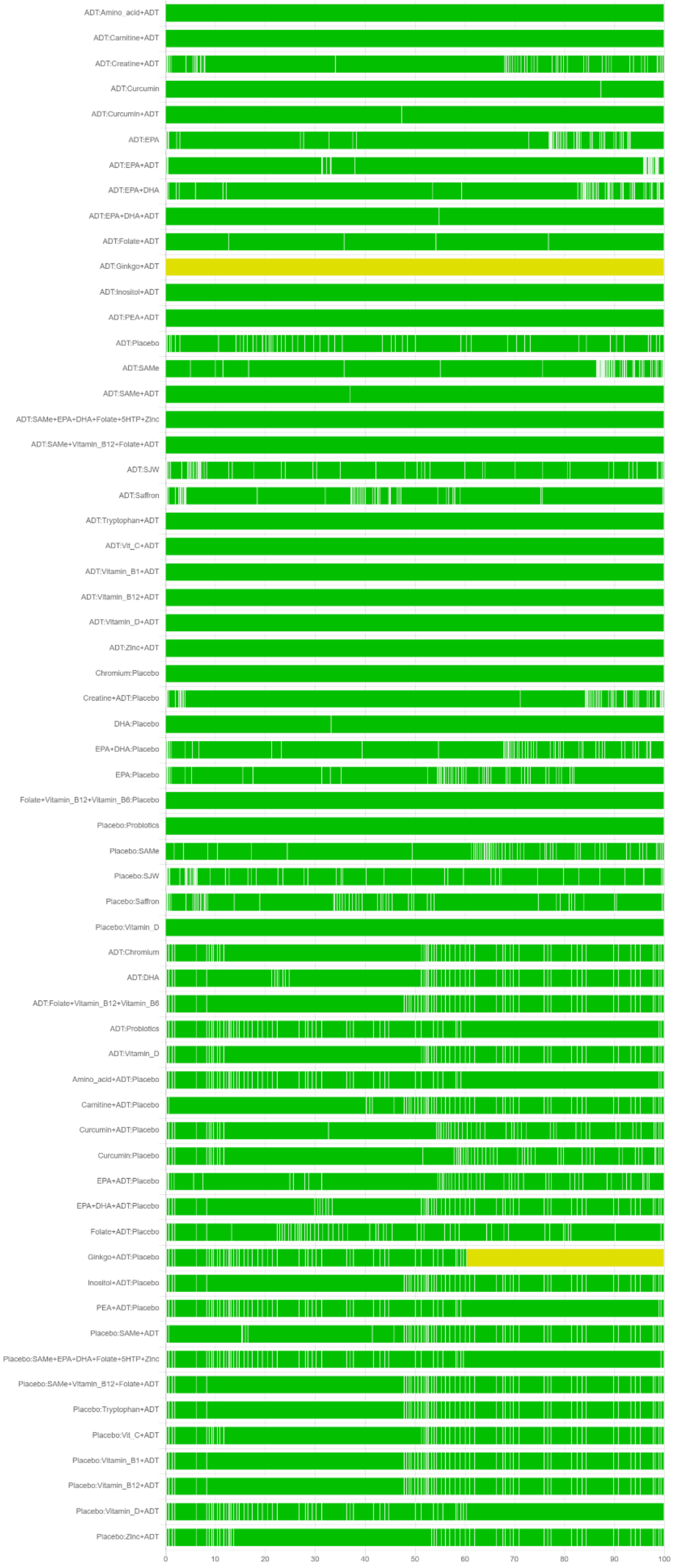

19.2.B.3.Confidence rating in trails for response rate

Comparison	Number of studies	Within-study bias	Reporting bias	Indirectness	Imprecision	Heterogeneity	Incoherence	Confidence rating
ADT:Amino_acid+ADT	1	Some concerns	Some concerns	No concerns	Some concerns	Some concerns	Major concerns	Very Low
ADT:Carnitine+ADT	1	Some concerns	Some concerns	No concerns	No concerns	No concerns	Major concerns	Very Low
ADT:Creatine+ADT	1	No concerns	Some concerns	No concerns	Some concerns	Some concerns	No concerns	Very Low
ADT:Curcumin	1	No concerns	Some concerns	No concerns	Major concerns	No concerns	Some concerns	Very Low
ADT:Curcumin+ADT	2	No concerns	Some concerns	No concerns	No concerns	No concerns	Major concerns	Very Low
ADT:EPA	2	No concerns	Some concerns	No concerns	Major concerns	No concerns	No concerns	Very Low
ADT:EPA+ADT	2	No concerns	Some concerns	No concerns	Major concerns	No concerns	No concerns	Very Low
ADT:EPA+DHA	2	No concerns	Some concerns	No concerns	Some concerns	Some concerns	No concerns	Low
ADT:EPA+DHA+ADT	2	No concerns	Some concerns	No concerns	Major concerns	No concerns	Major concerns	Very Low
ADT:Folate+ADT	5	No concerns	Some concerns	No concerns	Some concerns	Some concerns	Major concerns	Low
ADT:Ginkgo+ADT	1	Major concerns	Some concerns	Some concerns	Major concerns	No concerns	Major concerns	Very Low
ADT:Inositol+ADT	1	No concerns	Some concerns	No concerns	Major concerns	No concerns	Major concerns	Very Low
ADT:PEA+ADT	1	No concerns	Some concerns	No concerns	Some concerns	No concerns	Major concerns	Low
ADT:Placebo	8	No concerns	Some concerns	No concerns	No concerns	Some concerns	Major concerns	Very Low
ADT:SAMe	7	No concerns	Some concerns	No concerns	Some concerns	Some concerns	No concerns	Low
ADT:SAMe+ADT	2	Some concerns	Some concerns	No concerns	Some concerns	Some concerns	Major concerns	Low
ADT:SAMe+EPA+DHA+Folate+5HTP+Zinc+ADT	1	No concerns	Some concerns	No concerns	Major concerns	No concerns	Major concerns	Very Low
ADT:SAMe+Vitamin_B12+Folate+ADT	1	No concerns	Some concerns	No concerns	Major concerns	No concerns	Major concerns	Very Low
ADT:SJW	16	No concerns	Some concerns	No concerns	No concerns	Major concerns	No concerns	Low
ADT:Saffron	4	No concerns	Some concerns	No concerns	Some concerns	Some concerns	Major concerns	Low
ADT:Tryptophan+ADT	1	No concerns	Some concerns	No concerns	Major concerns	No concerns	Major concerns	Very Low
ADT:Vit_C+ADT	1	No concerns	Some concerns	No concerns	Major concerns	No concerns	Major concerns	Very Low

ADT:Vitamin_B1+ADT	1	No concerns	Some concerns	No concerns	No concerns	No concerns	Major concerns	Very Low
ADT:Vitamin_B12+ADT	1	Major concerns	Some concerns	No concerns	No concerns	No concerns	Major concerns	Very Low
ADT:Vitamin_D+ADT	1	No concerns	Some concerns	No concerns	Major concerns	No concerns	Major concerns	Very Low
ADT:Zinc+ADT	1	No concerns	Some concerns	No concerns	Major concerns	No concerns	Major concerns	Very Low
Chromium:Placebo	1	No concerns	Some concerns	No concerns	Major concerns	No concerns	Major concerns	Very Low
Creatine+ADT:Placebo	1	Some concerns	Some concerns	No concerns	Major concerns	No concerns	No concerns	Very Low
DHA:Placebo	2	No concerns	Some concerns	No concerns	Major concerns	No concerns	Major concerns	Low
EPA:Placebo	2	No concerns	Some concerns	No concerns	Some concerns	Some concerns	No concerns	Low
EPA+DHA:Placebo	2	No concerns	Some concerns	No concerns	No concerns	Some concerns	No concerns	Low
Folate+Vitamin_B12+Vitamin_B6:Placebo	1	No concerns	Some concerns	No concerns	Major concerns	No concerns	Major concerns	Very Low
Placebo:Probiotics	1	Some concerns	Some concerns	No concerns	Major concerns	No concerns	Major concerns	Very Low
Placebo:SAMe	3	No concerns	Some concerns	No concerns	No concerns	Some concerns	No concerns	Low
Placebo:SJW	19	No concerns	Some concerns	No concerns	No concerns	Some concerns	No concerns	Low
Placebo:Saffron	2	No concerns	Some concerns	No concerns	No concerns	Some concerns	Major concerns	Low
Placebo:Vitamin_D	1	No concerns	Some concerns	No concerns	Major concerns	No concerns	Major concerns	Very Low
ADT:Chromium	0	No concerns	Some concerns	No concerns	Major concerns	No concerns	Major concerns	Very Low
ADT:DHA	0	No concerns	Some concerns	No concerns	Some concerns	Some concerns	Major concerns	Very Low
ADT:Folate+Vitamin_B12+Vitamin_B6	0	No concerns	Some concerns	No concerns	Some concerns	No concerns	Major concerns	Very Low
ADT:Probiotics	0	Some concerns	Some concerns	No concerns	Some concerns	Some concerns	Major concerns	Very Low
ADT:Vitamin_D	0	No concerns	Some concerns	No concerns	Major concerns	No concerns	Major concerns	Very Low
Amino_acid+ADT:Placebo	0	Some concerns	Some concerns	No concerns	No concerns	Some concerns	Major concerns	Very Low
Carnitine+ADT:Placebo	0	Some concerns	Some concerns	No concerns	No concerns	No concerns	Major concerns	Very Low
Curcumin:Placebo	0	No concerns	Some concerns	No concerns	Some concerns	Some concerns	Major concerns	Very Low
Curcumin+ADT:Placebo	0	No concerns	Some concerns	No concerns	No concerns	No concerns	Major concerns	Very Low

EPA+ADT:Placebo	0	No concerns	Some concerns	No concerns	Some concerns	No concerns	Major concerns	Very Low
EPA+DHA+ADT:Placebo	0	No concerns	Some concerns	No concerns	Some concerns	Some concerns	Major concerns	Very Low
Folate+ADT:Placebo	0	No concerns	Some concerns	No concerns	No concerns	Some concerns	Major concerns	Very Low
Ginkgo+ADT:Placebo	0	Some concerns	Some concerns	No concerns	Some concerns	Some concerns	Major concerns	Very Low
Inositol+ADT:Placebo	0	No concerns	Some concerns	No concerns	Major concerns	No concerns	Major concerns	Very Low
PEA+ADT:Placebo	0	No concerns	Some concerns	No concerns	No concerns	No concerns	Major concerns	Very Low
Placebo:SAMe+ADT	0	No concerns	Some concerns	No concerns	No concerns	Some concerns	Major concerns	Very Low
Placebo:SAMe+EPA+DHA+Folate+5HTP+Zinc	0	No concerns	Some concerns	No concerns	Major concerns	No concerns	Major concerns	Very Low
Placebo:SAMe+Vitamin_B12+Folate+ADT	0	No concerns	Some concerns	No concerns	Some concerns	Some concerns	Major concerns	Very Low
Placebo:Tryptophan+ADT	0	No concerns	Some concerns	No concerns	Some concerns	No concerns	Major concerns	Very Low
Placebo:Vit_C+ADT	0	No concerns	Some concerns	No concerns	Major concerns	No concerns	Major concerns	Very Low
Placebo:Vitamin_B1+ADT	0	No concerns	Some concerns	No concerns	No concerns	No concerns	Major concerns	Very Low
Placebo:Vitamin_B12+ADT	0	Some concerns	Some concerns	No concerns	No concerns	No concerns	Major concerns	Very Low
Placebo:Vitamin_D+ADT	0	No concerns	Some concerns	No concerns	Major concerns	No concerns	Major concerns	Very Low
Placebo:Zinc+ADT	0	No concerns	Some concerns	No concerns	Some concerns	No concerns	Major concerns	Very Low

19.2.C. Remission rate

19.2.C-1.Network plot for the network meta-analysis of remission using four different sizing and coloring combinations.

Green, yellow, and red colors refer to low, moderate, and high risk of bias or indirectness

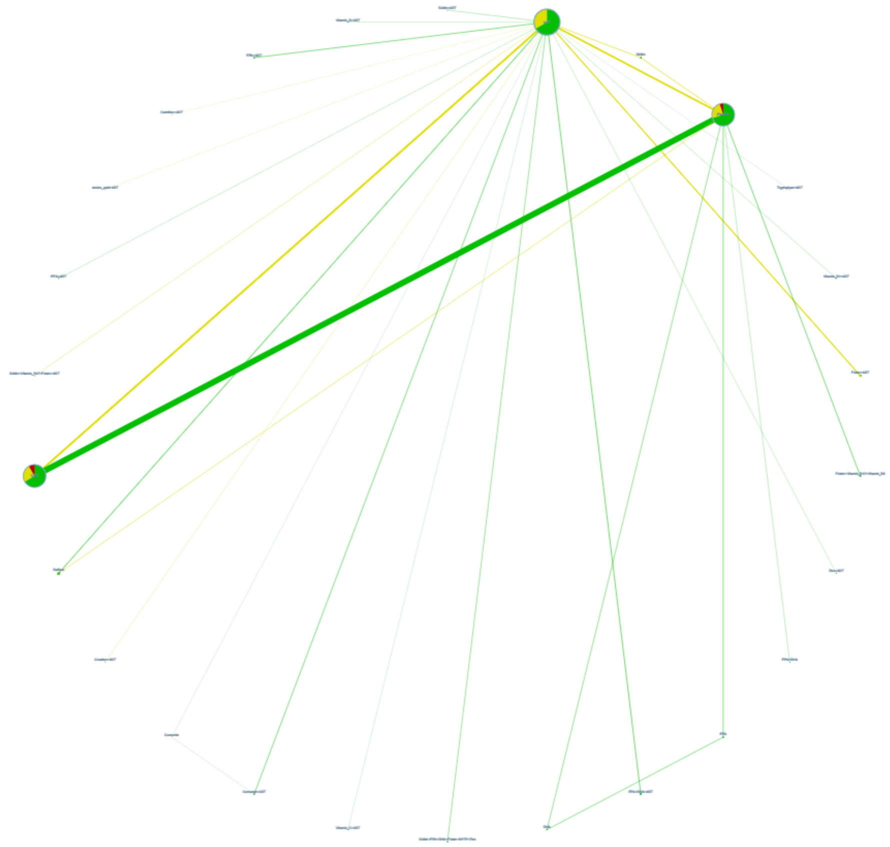

19.2.C-2. Indirectness chart for remission rate

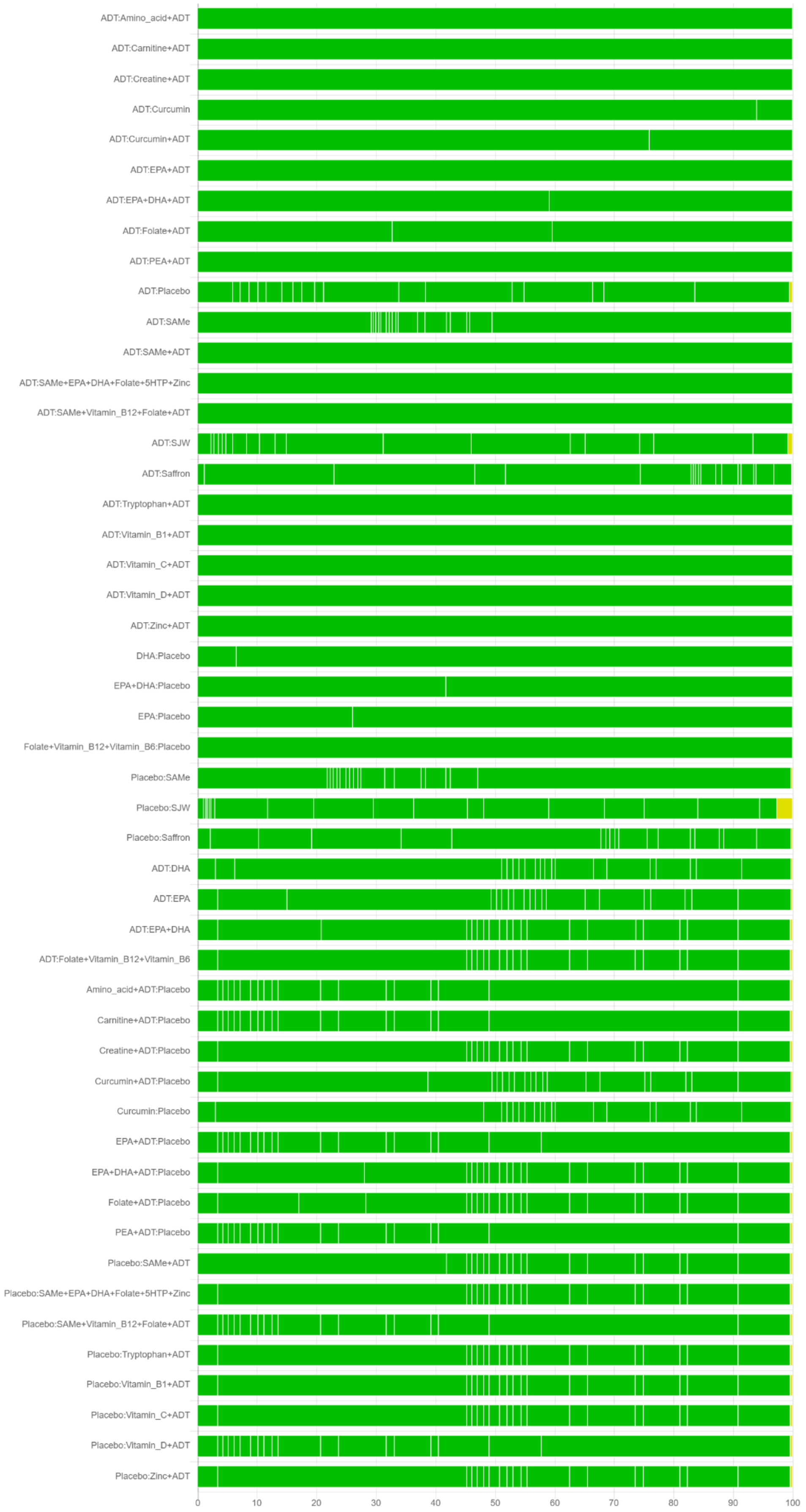

19.2.C-3. Confidence rating in trails for remission rate

Comparison	Number of studies	Within-study bias	Reporting bias	Indirectness	Imprecision	Heterogeneity	Incoherence	Confidence rating
ADT:Amino_acid+ADT	1	Some concerns	Some concerns	No concern	Major concerns	No concerns	Major concerns	Very Low
ADT:Carnitine+ADT	1	Some concerns	Some concerns	No concern	Major concerns	No concerns	Major concerns	Very Low
ADT:Creatine+ADT	1	Some concerns	Some concerns	No concern	Major concerns	No concerns	Major concerns	Very Low
ADT:Curcumin	1	No concerns	Some concerns	No concern	Major concerns	No concerns	Major concerns	Very Low
ADT:Curcumin+ADT	2	No concerns	Some concerns	No concern	Some concerns	Some concerns	Major concerns	Low
ADT:EPA+ADT	1	No concerns	Some concerns	No concern	Major concerns	No concerns	Major concerns	Very Low
ADT:EPA+DHA+ADT	2	No concerns	Some concerns	No concern	Major concerns	No concerns	Major concerns	Low
ADT:Folate+ADT	3	Some concerns	Some concerns	No concern	Major concerns	No concerns	Major concerns	Low
ADT:PEA+ADT	1	No concerns	Some concerns	No concern	Major concerns	No concerns	Major concerns	Very Low
ADT:Placebo	6	Some concerns	Some concerns	No concern	No concerns	Major concerns	Some concerns	Low
ADT:SAMe	2	Some concerns	Some concerns	No concern	Major concerns	No concerns	No concerns	Low
ADT:SAMe+ADT	1	No concerns	Some concerns	No concern	Some concerns	Some concerns	Major concerns	Low
ADT:SAMe+EPA+DHA+Folate+5HTP+Zinc	1	No concerns	Some concerns	No concern	Major concerns	No concerns	Major concerns	Very Low
ADT:SAMe+Vitamin_B12+Folate+ADT	1	Some concerns	Some concerns	No concern	Major concerns	No concerns	Major concerns	Very Low
ADT:SJW	5	Some concerns	Some concerns	No concern	Some concerns	Some concerns	No concerns	Low
ADT:Saffron	3	No concerns	Some concerns	No concern	Major concerns	No concerns	Major concerns	Low
ADT:Tryptophan+ADT	1	No concerns	Some concerns	No concern	Some concerns	Some concerns	Major concerns	Very Low
ADT:Vitamin_B1+ADT	1	No concerns	Some concerns	No concern	Major concerns	No concerns	Major concerns	Very Low
ADT:Vitamin_C+ADT	1	No concerns	Some concerns	No concern	Major concerns	No concerns	Major concerns	Very Low
ADT:Vitamin_D+ADT	1	No concerns	Some concerns	No concern	Major concerns	No concerns	Major concerns	Very Low
ADT:Zinc+ADT	1	No concerns	Some concerns	No concern	Major concerns	No concerns	Major concerns	Very Low
DHA:Placebo	1	No concerns	Some concerns	No concern	Major concerns	No concerns	No concerns	Very Low

EPA:Placebo	2	No concerns	Some concerns	No concern	Major concerns	No concerns	Major concerns	Low
EPA+DHA:Placebo	2	No concerns	Some concerns	No concern	Some concerns	Some concerns	Major concerns	Low
Folate+Vitamin_B12+Vitamin_B6:Placebo	1	No concerns	Some concerns	No concern	Major concerns	No concerns	Major concerns	Very Low
Placebo:SAMe	2	Some concerns	Some concerns	No concern	Some concerns	Some concerns	No concerns	Low
Placebo:SJW	11	No concerns	Some concerns	No concern	No concerns	Some concerns	No concerns	Very Low
Placebo:Saffron	2	No concerns	Some concerns	No concern	No concerns	Major concerns	Major concerns	Very Low
ADT:DHA	0	No concerns	Some concerns	No concern	Major concerns	No concerns	Major concerns	Low
ADT:EPA	0	No concerns	Some concerns	No concern	Major concerns	No concerns	Major concerns	Low
ADT:EPA+DHA	0	No concerns	Some concerns	No concern	Major concerns	No concerns	Major concerns	Low
ADT:Folate+Vitamin_B12+Vitamin_B6	0	No concerns	Some concerns	No concern	Major concerns	No concerns	Major concerns	Very Low
Amino_acid+ADT:Placebo	0	Some concerns	Some concerns	No concern	No concerns	Some concerns	Major concerns	Very Low
Carnitine+ADT:Placebo	0	Some concerns	Some concerns	No concern	Major concerns	No concerns	Major concerns	Low
Creatine+ADT:Placebo	0	Some concerns	Some concerns	No concern	Some concerns	No concerns	Major concerns	Very Low
Curcumin:Placebo	0	No concerns	Some concerns	No concern	Major concerns	No concerns	Major concerns	Very Low
Curcumin+ADT:Placebo	0	No concerns	Some concerns	No concern	No concerns	Some concerns	Major concerns	Very Low
EPA+ADT:Placebo	0	No concerns	Some concerns	No concern	Major concerns	No concerns	Major concerns	Low
EPA+DHA+ADT:Placebo	0	No concerns	Some concerns	No concern	Some concerns	Some concerns	Major concerns	Low
Folate+ADT:Placebo	0	Some concerns	Some concerns	No concern	Some concerns	Some concerns	Major concerns	Low
PEA+ADT:Placebo	0	No concerns	Some concerns	No concern	Some concerns	Some concerns	Major concerns	Low
Placebo:SAMe+ADT	0	No concerns	Some concerns	No concern	No concerns	Some concerns	Major concerns	Very Low
Placebo:SAMe+EPA+DHA+Folate+5HTP+Zinc	0	No concerns	Some concerns	No concern	Major concerns	No concerns	Major concerns	Very Low
Placebo:SAMe+Vitamin_B12+Folate+ADT	0	Some concerns	Some concerns	No concern	Major concerns	No concerns	Major concerns	Very Low
Placebo:Tryptophan+ADT	0	No concerns	Some concerns	No concern	No concerns	Some concerns	Major concerns	Very Low
Placebo:Vitamin_B1+ADT	0	No concerns	Some concerns	No concern	Major concerns	No concerns	Major concerns	Very Low

Placebo:Vitamin_C+ADT	0	No concerns	Some concerns	No concern	Major concerns	No concerns	Major concerns	Very Low
Placebo:Vitamin_D+ADT	0	No concerns	Some concerns	No concern	Major concerns	No concerns	Major concerns	Low
Placebo:Zinc+ADT	0	No concerns	Some concerns	No concern	Some concerns	Some concerns	Major concerns	Very Low

19.2.D. Change in anxiety symptom

19.2.D-1.Network plot for the network meta-analysis of change in anxiety symptom using four different sizing and coloring combinations.

Green, yellow, and red colors refer to low, moderate, and high risk of bias or indirectness

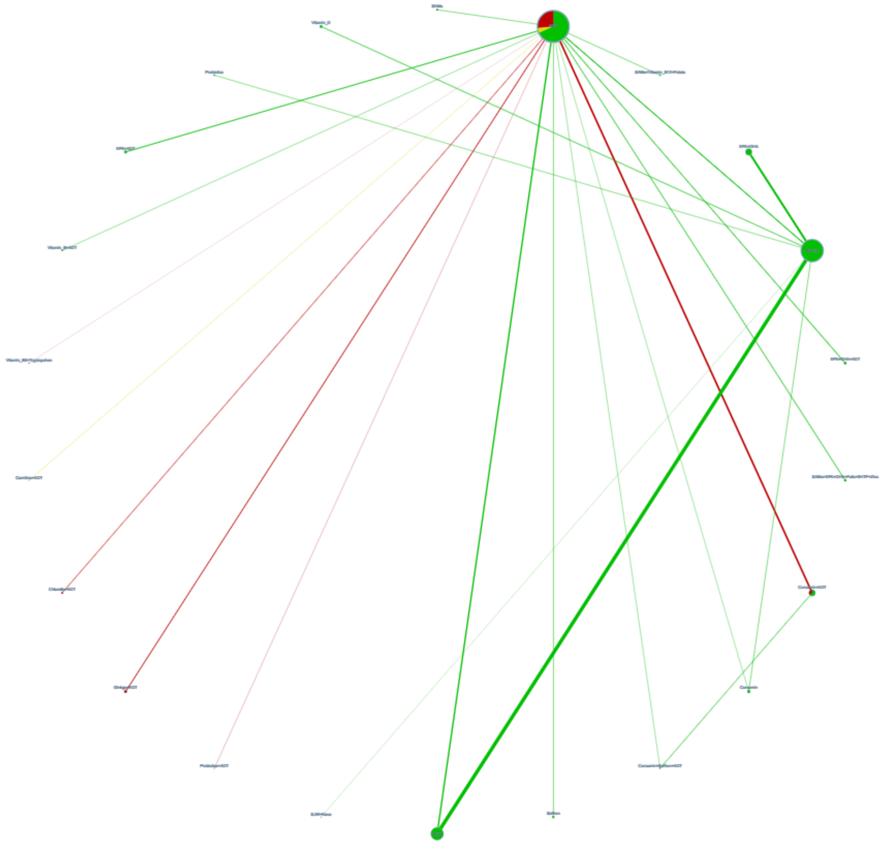

19.2.D-2.Indirectness chart for change in anxiety symptom

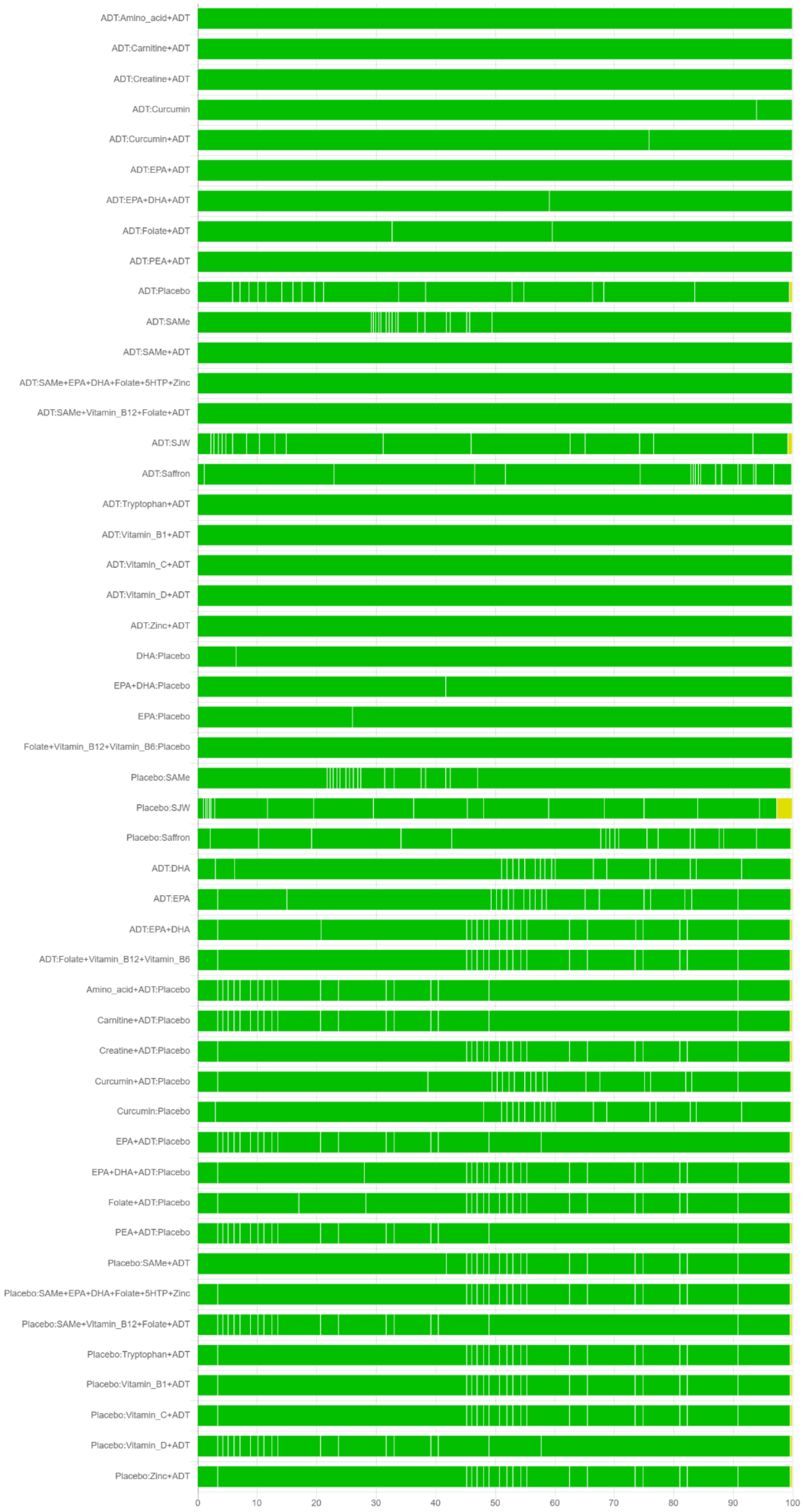

19.2.D-3.Confidence rating in trails for change in anxiety symptom

Comparison	Number of studies	Within-study bias	Reporting bias	Indirectness	Imprecision	Heterogeneity	Incoherence	Confidence rating
ADT:Carnitine+ADT	1	Some concerns	Some concerns	No concerns	Major concerns	No concerns	No concern	Very Low
ADT:Chlorella+ADT	1	Major concerns	Some concerns	Some concerns	Major concerns	No concerns	No concern	Very Low
ADT:Curcumin	1	No concerns	Some concerns	No concerns	Major concerns	No concerns	No concern	Low
ADT:Curcumin+ADT	3	Some concerns	Some concerns	No concerns	Some concerns	Some concerns	No concern	Low
ADT:Curcumin+Saffron+ADT	1	No concerns	Some concerns	No concerns	Major concerns	No concerns	No concern	Very Low
ADT:EPA+ADT	1	No concerns	Some concerns	No concerns	Major concerns	No concerns	No concern	Very Low
ADT:EPA+DHA+ADT	1	No concerns	Some concerns	No concerns	Major concerns	No concerns	No concern	Very Low
ADT:Ginkgo+ADT	1	Major concerns	Some concerns	Some concerns	Major concerns	No concerns	No concern	Very Low
ADT:Placebo	1	No concerns	Some concerns	No concerns	Major concerns	No concerns	No concern	Low
ADT:Probiotics+ADT	1	Major concerns	Some concerns	Some concerns	No concerns	No concerns	No concern	Very Low
ADT:SAMe	2	No concerns	Some concerns	No concerns	Major concerns	No concerns	No concern	Very Low
ADT:SAMe+EPA+DHA+Folic+5HTP+Zinc	1	No concerns	Some concerns	No concerns	Major concerns	No concerns	No concern	Very Low
ADT:SAMe+Vitamin_B12+Folate	1	No concerns	Some concerns	No concerns	Major concerns	No concerns	No concern	Very Low
ADT:SJW	1	No concerns	Some concerns	No concerns	Major concerns	No concerns	No concern	Very Low
ADT:Saffron	2	No concerns	Some concerns	No concerns	No concerns	Major concerns	No concern	Very Low
ADT:Vitamin_B+ADT	1	No concerns	Some concerns	No concerns	Major concerns	No concerns	No concern	Very Low
ADT:Vitamin_B6+Tryptopohan	1	Major concerns	Some concerns	Some concerns	Major concerns	No concerns	No concern	Low
Curcumin:Placebo	1	No concerns	Some concerns	No concerns	Major concerns	No concerns	No concern	Low
EPA+DHA:Placebo	2	No concerns	Some concerns	No concerns	Some concerns	Some concerns	No concern	Very Low
Placebo:Probiotics	1	No concerns	Some concerns	No concerns	Major concerns	No concerns	No concern	Very Low
Placebo:SJW	3	No concerns	Some concerns	No concerns	Major concerns	No concerns	No concern	Low
Placebo:SJW+Kava	1	No concerns	Some concerns	No concerns	Major concerns	No concerns	No concern	Very Low

Placebo:Vitamin_D	1	No concerns	Some concerns	No concerns	No concerns	No concerns	No concern	Very Low
ADT:EPA+DHA	0	No concerns	Some concerns	No concerns	Major concerns	No concerns	No concern	Low
ADT:Probiotics	0	No concerns	Some concerns	No concerns	Major concerns	No concerns	No concern	Very Low
ADT:SJW+Kava	0	No concerns	Some concerns	No concerns	Major concerns	No concerns	No concern	Very Low
ADT:Vitamin_D	0	No concerns	Some concerns	No concerns	No concerns	Major concerns	No concern	Very Low
Carnitine+ADT:Placebo	0	No concerns	Some concerns	No concerns	Major concerns	No concerns	No concern	Very Low
Chlorella+ADT:Placebo	0	Some concerns	Some concerns	No concerns	Major concerns	No concerns	No concern	Very Low
Curcumin+ADT:Placebo	0	No concerns	Some concerns	No concerns	Major concerns	No concerns	No concern	Very Low
Curcumin+Saffron+ADT:Placebo	0	No concerns	Some concerns	No concerns	Major concerns	No concerns	No concern	Very Low
EPA+ADT:Placebo	0	No concerns	Some concerns	No concerns	Major concerns	No concerns	No concern	Very Low
EPA+DHA+ADT:Placebo	0	No concerns	Some concerns	No concerns	Major concerns	No concerns	No concern	Very Low
Ginkgo+ADT:Placebo	0	Some concerns	Some concerns	No concerns	Major concerns	No concerns	No concern	Very Low
Placebo:Probiotics+ADT	0	Some concerns	Some concerns	No concerns	No concerns	No concerns	No concern	Very Low
Placebo:SAMe	0	No concerns	Some concerns	No concerns	Major concerns	No concerns	No concern	Very Low
Placebo:SAMe+EPA+DHA+Folic+5HTP+Zinc	0	No concerns	Some concerns	No concerns	Major concerns	No concerns	No concern	Very Low
Placebo:SAMe+Vitamin_B12+Folate	0	No concerns	Some concerns	No concerns	Major concerns	No concerns	No concern	Very Low
Placebo:Saffron	0	No concerns	Some concerns	No concerns	No concerns	Major concerns	No concern	Low
Placebo:Vitamin_B+ADT	0	No concerns	Some concerns	No concerns	Major concerns	No concerns	No concern	Very Low
Placebo:Vitamin_B6+Tryptophan	0	Some concerns	Some concerns	No concerns	Major concerns	No concerns	No concern	Very Low

19.2.E. All cause discontinuation

19.2.E-1.Network plot for the network meta-analysis of all cause discontinuation using four different sizing and coloring combinations.

Green, yellow, and red colors refer to low, moderate, and high risk of bias or indirectness

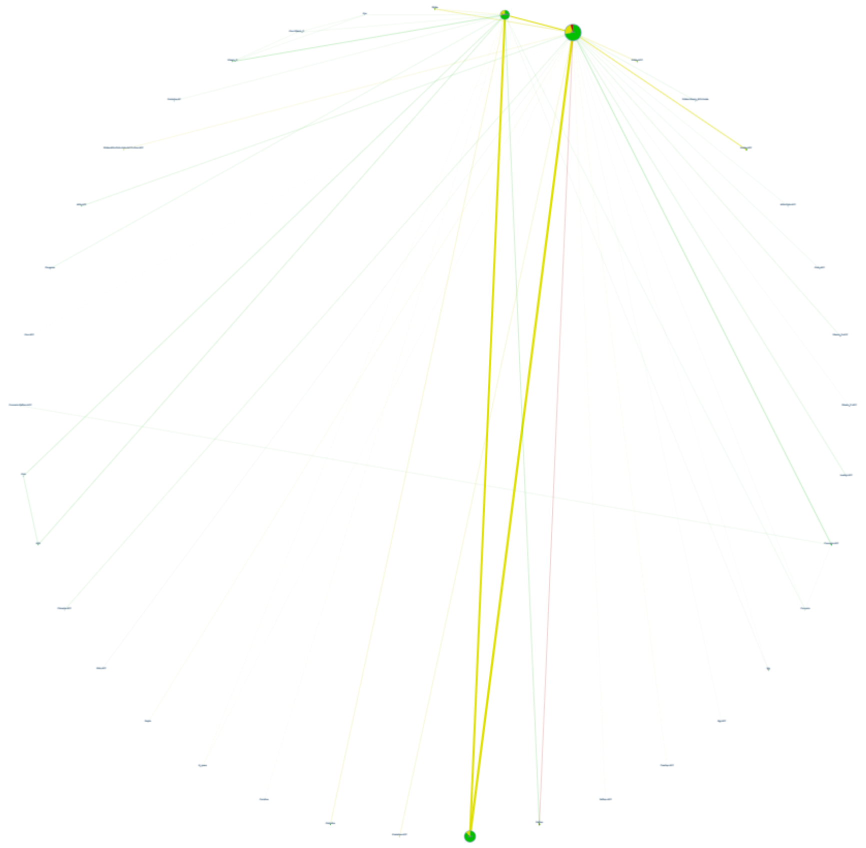

19.2.E-2.Indirectness chart for all cause discontinuation

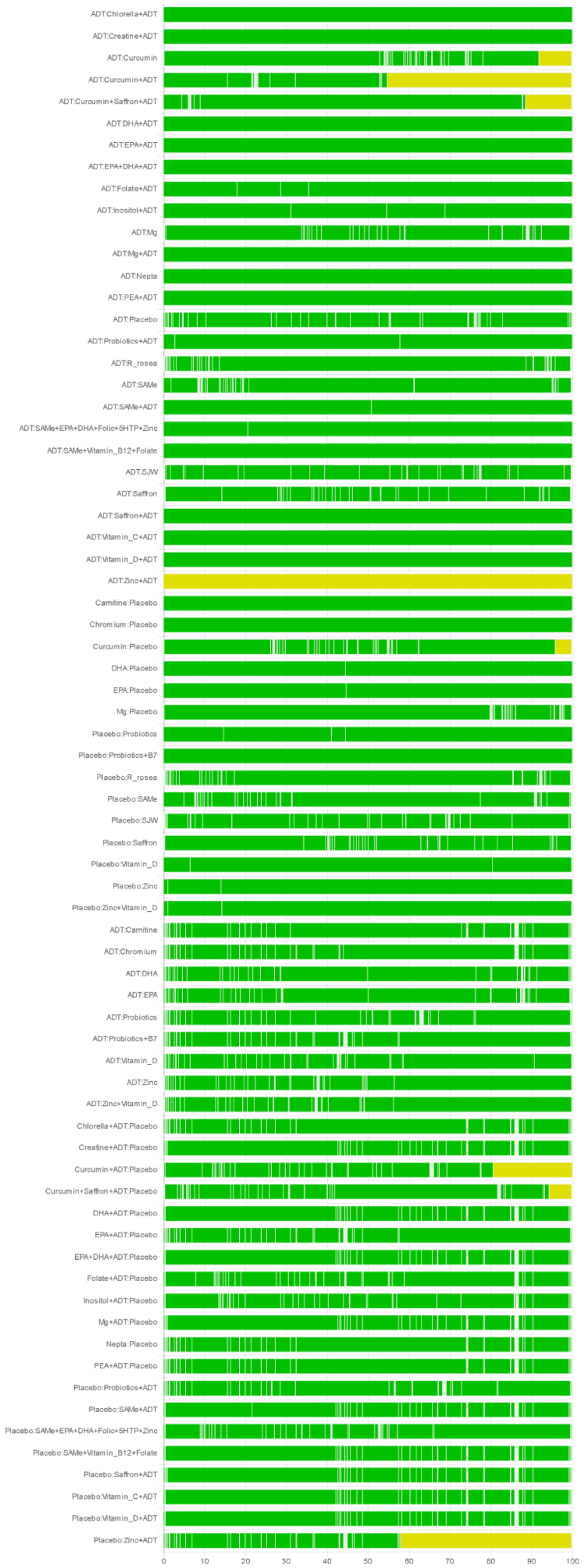

19.2.E-3.Confidence rating in trails for all cause discontinuation

Comparison	Number of studies	Within-study bias	Reporting bias	Indirectness	Imprecision	Heterogeneity	Incoherence	Confidence rating
ADT:Chlorella+ADT	1	No concerns	Some concerns	No concern	Some concerns	No concerns	No concern	Very Low
ADT:Creatine+ADT	1	Some concerns	Some concerns	No concern	Some concerns	No concerns	No concern	Very Low
ADT:Curcumin	1	No concerns	Some concerns	No concern	Some concerns	No concerns	No concern	Very Low
ADT:Curcumin+ADT	5	No concerns	Some concerns	No concern	Some concerns	No concerns	No concern	Low
ADT:DHA+ADT	1	No concerns	Some concerns	No concern	Some concerns	No concerns	No concern	Very Low
ADT:EPA+ADT	1	No concerns	Some concerns	No concern	Major concerns	No concerns	No concern	Low
ADT:EPA+DHA+ADT	1	No concerns	Some concerns	No concern	No concerns	No concerns	No concern	Very Low
ADT:Folate+ADT	4	No concerns	Some concerns	No concern	Some concerns	No concerns	No concern	Low
ADT:Inositol+ADT	4	No concerns	Some concerns	No concern	Major concerns	No concerns	No concern	Low
ADT:Mg	1	Some concerns	Some concerns	No concern	Major concerns	No concerns	No concern	Very Low
ADT:Mg+ADT	1	No concerns	Some concerns	No concern	Some concerns	No concerns	No concern	Very Low
ADT:Nepta	1	Some concerns	Some concerns	No concern	Major concerns	No concerns	No concern	Low
ADT:PEA+ADT	1	No concerns	Some concerns	No concern	Major concerns	No concerns	No concern	Very Low
ADT:Placebo	10	No concerns	Some concerns	No concern	No concerns	No concerns	No concern	Low
ADT:Probiotics+ADT	3	Some concerns	Some concerns	No concern	Some concerns	No concerns	No concern	Low
ADT:R_rosea	1	Some concerns	Some concerns	No concern	Some concerns	No concerns	No concern	Low
ADT:SAMe	3	No concerns	Some concerns	No concern	Some concerns	No concerns	No concern	Low
ADT:SAMe+ADT	2	Some concerns	Some concerns	No concern	Some concerns	No concerns	No concern	Low
ADT:SAMe+EPA+DHA+Folic+5HTP+Zinc+ADT	1	Some concerns	Some concerns	No concern	Some concerns	No concerns	No concern	Very Low
ADT:SAMe+Vitamin_B12+Folate	1	No concerns	Some concerns	No concern	Some concerns	No concerns	No concern	Very Low
ADT:SJW	18	No concerns	Some concerns	No concern	No concerns	No concerns	No concern	Low
ADT:Saffron	5	No concerns	Some concerns	No concern	Some concerns	No concerns	No concern	Low

ADT:Saffron+ADT	1	Some concerns	Some concerns	No concern	Major concerns	No concerns	No concern	Very Low
ADT:Vitamin_C+ADT	1	No concerns	Some concerns	No concern	Major concerns	No concerns	No concern	Very Low
ADT:Vitamin_D+ADT	2	No concerns	Some concerns	No concern	Some concerns	No concerns	No concern	Low
ADT:Zinc+ADT	1	No concerns	Some concerns	No concern	Major concerns	No concerns	No concern	Very Low
Carnitine:Placebo	1	Some concerns	Some concerns	No concern	Major concerns	No concerns	No concern	Very Low
Chromium:Placebo	1	No concerns	Some concerns	No concern	Major concerns	No concerns	No concern	Very Low
Curcumin:Placebo	1	No concerns	Some concerns	No concern	Some concerns	No concerns	No concern	Very Low
DHA:Placebo	2	No concerns	Some concerns	No concern	Some concerns	No concerns	No concern	Very Low
EPA:Placebo	2	No concerns	Some concerns	No concern	Major concerns	No concerns	No concern	Low
Mg:Placebo	1	No concerns	Some concerns	No concern	Major concerns	No concerns	No concern	Very Low
Placebo:Probiotics	4	Some concerns	Some concerns	No concern	Some concerns	No concerns	No concern	Low
Placebo:Probiotics+B7	1	No concerns	Some concerns	No concern	Some concerns	No concerns	No concern	Very Low
Placebo:R_rosea	1	Some concerns	Some concerns	No concern	Major concerns	No concerns	No concern	Very Low
Placebo:SAMe	2	Some concerns	Some concerns	No concern	No concerns	No concerns	No concern	Very Low
Placebo:SJW	15	No concerns	Some concerns	No concern	No concerns	No concerns	No concern	Very Low
Placebo:Saffron	4	No concerns	Some concerns	No concern	No concerns	No concerns	No concern	Very Low
Placebo:Vitamin_D	3	No concerns	Some concerns	No concern	No concerns	No concerns	No concern	Low
Placebo:Zinc	1	No concerns	Some concerns	No concern	Major concerns	No concerns	No concern	Very Low
Placebo:Zinc+Vitamin_D	1	No concerns	Some concerns	No concern	Some concerns	No concerns	No concern	Very Low
ADT:Carnitine	0	Some concerns	Some concerns	No concern	Major concerns	No concerns	No concern	Low
ADT:Chromium	0	No concerns	Some concerns	No concern	Major concerns	No concerns	No concern	Very Low
ADT:Curcumin+Saffron+ADT	0	No concerns	Some concerns	No concern	Major concerns	No concerns	No concern	Very Low
ADT:DHA	0	No concerns	Some concerns	No concern	Some concerns	No concerns	No concern	Very Low
ADT:EPA	0	No concerns	Some concerns	No concern	Major concerns	No concerns	No concern	Very Low

ADT:Probiotics	0	Some concerns	Some concerns	No concern	Major concerns	No concerns	No concern	Very Low
ADT:Probiotics+B7	0	No concerns	Some concerns	No concern	Some concerns	No concerns	No concern	Very Low
ADT:Vitamin_D	0	No concerns	Some concerns	No concern	No concerns	No concerns	No concern	Very Low
ADT:Zinc	0	No concerns	Some concerns	No concern	Some concerns	No concerns	No concern	Very Low
ADT:Zinc+Vitamin_D	0	No concerns	Some concerns	No concern	Some concerns	No concerns	No concern	Very Low
Chlorella+ADT:Placebo	0	No concerns	Some concerns	No concern	Some concerns	No concerns	No concern	Very Low
Creatine+ADT:Placebo	0	Some concerns	Some concerns	No concern	Some concerns	No concerns	No concern	Very Low
Curcumin+ADT:Placebo	0	No concerns	Some concerns	No concern	Some concerns	No concerns	No concern	Very Low
Curcumin+Saffron+ADT:Placebo	0	No concerns	Some concerns	No concern	Major concerns	No concerns	No concern	Very Low
DHA+ADT:Placebo	0	No concerns	Some concerns	No concern	Some concerns	No concerns	No concern	Very Low
EPA+ADT:Placebo	0	No concerns	Some concerns	No concern	Major concerns	No concerns	No concern	Low
EPA+DHA+ADT:Placebo	0	No concerns	Some concerns	No concern	Some concerns	No concerns	No concern	Very Low
Folate+ADT:Placebo	0	No concerns	Some concerns	No concern	No concerns	No concerns	No concern	Very Low
Inositol+ADT:Placebo	0	No concerns	Some concerns	No concern	Some concerns	No concerns	No concern	Very Low
Mg+ADT:Placebo	0	No concerns	Some concerns	No concern	Major concerns	No concerns	No concern	Very Low
Nepta:Placebo	0	Some concerns	Some concerns	No concern	Major concerns	No concerns	No concern	Very Low
PEA+ADT:Placebo	0	No concerns	Some concerns	No concern	Major concerns	No concerns	No concern	Very Low
Placebo:Probiotics+ADT	0	No concerns	Some concerns	No concern	Some concerns	No concerns	No concern	Very Low
Placebo:SAMe+ADT	0	No concerns	Some concerns	No concern	Some concerns	No concerns	No concern	Very Low
Placebo:SAMe+EPA+DHA+Folic +5HTP+Zinc+ADT	0	Some concerns	Some concerns	No concern	Some concerns	No concerns	No concern	Very Low
Placebo:SAMe+Vitamin_B12+Folate	0	No concerns	Some concerns	No concern	Some concerns	No concerns	No concern	Very Low
Placebo:Saffron+ADT	0	Some concerns	Some concerns	No concern	Major concerns	No concerns	No concern	Very Low
Placebo:Vitamin_C+ADT	0	No concerns	Some concerns	No concern	Major concerns	No concerns	No concern	Very Low
Placebo:Vitamin_D+ADT	0	No concerns	Some concerns	No concern	Some concerns	No concerns	No concern	Very Low

Placebo:Zinc+ADT	0	No concerns	Some concerns	No concern[illegible]	Major concerns	No concerns	No concern[illegible]	Very Low

19.2.F Adverse event

19.2.F-1.Network plot for the network meta-analysis of adverse event using four different sizing and coloring combinations.

Green, yellow, and red colors refer to low, moderate, and high risk of bias or indirectness

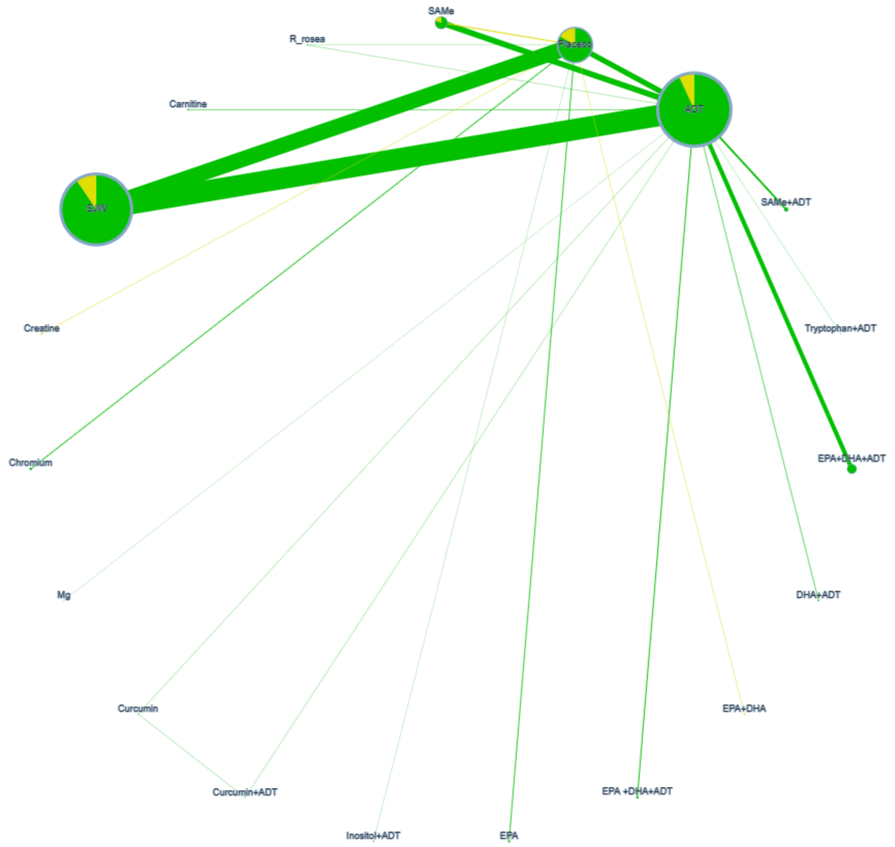

19.2.F-2.Indirectness chart for adverse event

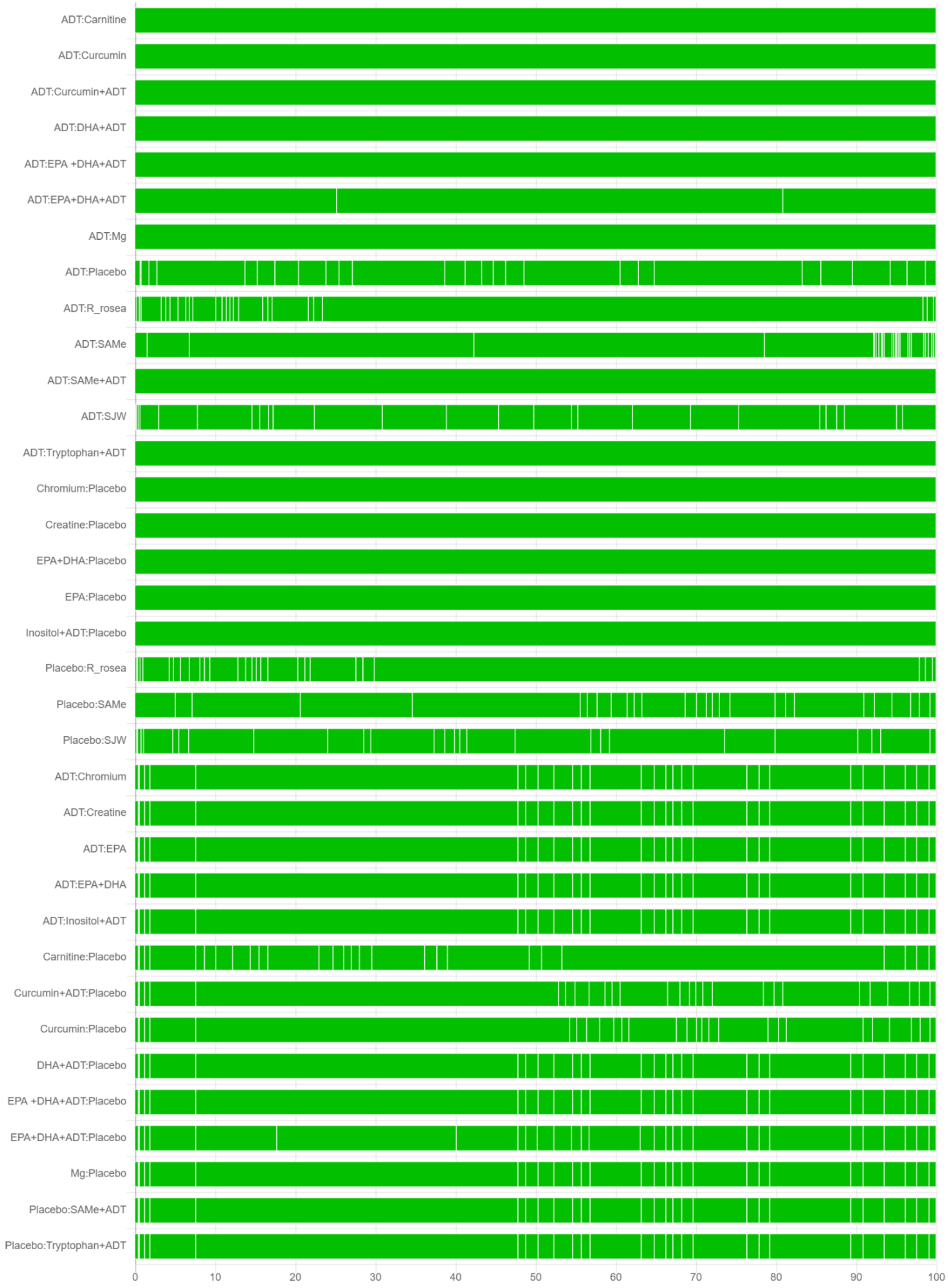

19.2.F-3.Confidence rating in trails for adverse event

Comparison	Number of studies	Within-study bias	Reporting bias	Indirectness	Imprecision	Heterogeneity	Incoherence	Confidence rating
ADT:Carnitine	1	No concerns	Some concerns	No concerns	No concerns	Some concerns	Some concerns	Very Low
ADT:Curcumin	1	No concerns	Some concerns	No concerns	Major concerns	No concerns	Some concerns	Very Low
ADT:Curcumin+ADT	1	No concerns	Some concerns	No concerns	Major concerns	No concerns	Some concerns	Very Low
ADT:DHA+ADT	1	No concerns	Some concerns	No concerns	Major concerns	No concerns	Some concerns	Very Low
ADT:EPA +DHA+ADT	1	No concerns	Some concerns	No concerns	Major concerns	No concerns	Some concerns	Very Low
ADT:EPA+DHA+ADT	3	No concerns	Some concerns	No concerns	No concerns	Major concerns	Some concerns	Low
ADT:Mg	1	No concerns	Some concerns	No concerns	Major concerns	No concerns	Some concerns	Very Low
ADT:Placebo	5	No concerns	Some concerns	No concerns	No concerns	Some concerns	No concerns	Low
ADT:R_rosea	1	No concerns	Some concerns	No concerns	Some concerns	Some concerns	Some concerns	Very Low
ADT:SAMe	4	No concerns	Some concerns	No concerns	No concerns	Some concerns	Some concerns	Low
ADT:SAMe+ADT	1	No concerns	Some concerns	No concerns	Major concerns	No concerns	Some concerns	Very Low
ADT:SJW	14	No concerns	Some concerns	No concerns	No concerns	Some concerns	No concerns	Low
ADT:Tryptophan+ADT	1	No concerns	Some concerns	No concerns	Some concerns	Some concerns	Some concerns	Very Low
Chromium:Placebo	1	No concerns	Some concerns	No concerns	Some concerns	Some concerns	Some concerns	Very Low
Creatine:Placebo	1	Some concerns	Some concerns	No concerns	Major concerns	No concerns	Some concerns	Very Low
EPA:Placebo	1	No concerns	Some concerns	No concerns	Some concerns	No concerns	Some concerns	Very Low
EPA+DHA:Placebo	1	Some concerns	Some concerns	No concerns	Major concerns	No concerns	Some concerns	Very Low
Inositol+ADT:Placebo	1	No concerns	Some concerns	No concerns	Major concerns	No concerns	Some concerns	Very Low
Placebo:R_rosea	1	No concerns	Some concerns	No concerns	Major concerns	No concerns	Some concerns	Very Low
Placebo:SAMe	2	No concerns	Some concerns	No concerns	Some concerns	Some concerns	Major concerns	Low
Placebo:SJW	10	No concerns	Some concerns	No concerns	No concerns	Major concerns	No concerns	Low
ADT:Chromium	0	No concerns	Some concerns	No concerns	Some concerns	No concerns	Some concerns	Very Low

ADT:Creatine	0	No concerns	Some concerns	No concerns	Major concerns	No concerns	Some concerns	Very Low
ADT:EPA	0	No concerns	Some concerns	No concerns	Major concerns	No concerns	Some concerns	Very Low
ADT:EPA+DHA	0	No concerns	Some concerns	No concerns	Some concerns	No concerns	Some concerns	Very Low
ADT:Inositol+ADT	0	No concerns	Some concerns	No concerns	Major concerns	No concerns	Some concerns	Very Low
Carnitine:Placebo	0	No concerns	Some concerns	No concerns	Some concerns	Some concerns	Some concerns	Very Low
Curcumin:Placebo	0	No concerns	Some concerns	No concerns	Major concerns	No concerns	Some concerns	Very Low
Curcumin+ADT:Placebo	0	No concerns	Some concerns	No concerns	Some concerns	No concerns	Some concerns	Very Low
DHA+ADT:Placebo	0	No concerns	Some concerns	No concerns	Major concerns	No concerns	Some concerns	Very Low
EPA +DHA+ADT:Place[illegible]	0	No concerns	Some concerns	No concerns	Some concerns	Some concerns	Some concerns	Very Low
EPA+DHA+ADT: Placebo	0	No concerns	Some concerns	No concerns	Some concerns	No concerns	Some concerns	Low
Mg:Placebo	0	No concerns	Some concerns	No concerns	Major concerns	No concerns	Some concerns	Very Low
Placebo:SAMe+ADT	0	No concerns	Some concerns	No concerns	Some concerns	No concerns	Some concerns	Very Low
Placebo:Tryptophan+ADT	0	No concerns	Some concerns	No concerns	Some concerns	No concerns	Some concerns	Very Low

20. Assessment of transitivity

Treatment	Corresponding code
ADT	AA
Amino_acid+ADT	AB
Carnitine	AC
Carnitine+ADT	AD
Chlorella+ADT	AE
Creatine+ADT	AF
Curcumin	AG
Curcumin+ADT	AH
Curcumin+Saffron+ADT	AI
DHA	AJ
DHA+ADT	AK
EPA	AL
EPA+ADT	AM
EPA+DHA	AN
EPA+DHA+ADT	AO
EPA+DHA+Vitamin_C	AP
E_amoenum	AQ
Fe	AR
Folate+ADT	AS
Folate+Vitamin_B12+Vitamin_B6	AT
Ginkgo+ADT	AU
Inositol+ADT	AV
L-arginine+ADT	AW
L-theanine+ADT	AX
Lavandula	AY
Lavandula+ADT	AZ
Mg	BA
Mg+ADT	BB
Nepta	BC
PEA+ADT	BD
Placebo (reference)	BE
Prebiotics+ADT	BF
Probiotics	BG
Probiotics+ADT	BH
Probiotics+B7	BI
Probiotics+Mg+CoQ10+ADT	BJ
R_rosea	BK
SAMe	BL

SAMe+ADT	BM
SAMe+Probiotics	BN
SAMe+Vitamin_B12+Folate+ADT	BO
SAMe+n3+Folate+5HTP+Zinc+ADT	BP
SJW	BQ
SJW+ADT	BR
SJW+Kava	BS
Saffron	BT
Saffron+ADT	BU
Tryptophan+ADT	BV
Vitamin_B+ADT	BW
Vitamin_B1+ADT	BX
Vitamin_B12+ADT	BY
Vitamin_B6+Tryptophan	BZ
Vitamin_C	CA
Vitamin_C+ADT	CB
Vitamin_D	CC
Vitamin_D+ADT	CD
Vitamin_D+Ca	CE
Zinc	CF
Zinc+ADT	CG
Zinc+Vitamin_D	CH

Before conducting the statistical analysis, we assessed whether the trials included in the NMA were on average similar in terms of characteristics that might modify the treatment effect (so that the transitivity assumption is plausible). Indirect comparisons, in contrast to direct comparisons, are not protected by randomisation and may be confounded by differences between the trials. In our analysis we deemed the following parameters as possible confounders: study length, baseline depression severity, mean age, publication year and sample size based on results from a prior analysis.
The plausibility of the transitivity assumption was evaluated by comparing the distribution of these potential effect modifiers across studies grouped by comparison. The impact of the individual parameters was assessed by several metaregressions (appendix 17) and sensitivity analyses (appendix 12).

Treatment intervention and their corresponding code
ADT: Antidepressant; Ca: Calcium ; DHA: Docosahexaenoic Acid ; E Amoenum : Echium amoenum; EPA :Eicosapentaenoic acid; Fe : Ferrum; Mg: Magnesium; PEA: Palmitoylethanolamide; R rosea : Rhodiola rosea; SAMe: S-Adenosyl Methionine ; SJW: St. John's wort ;Vitamin B1: Thiamine ; Vitamin B6: Pyridoxine; Vitamin B7: Biotin; Vitamin B: Vitamin B complex; Vitamin B12 : Cobalamin; Vitamin C: Ascorbic acid; Vitamin D: Cholecalciferol; 5HTP: 5-Hydroxytryptophan

20.1. Assessment of transitivity-Study length

The overall median study duration was 8 weeks. The study duration ranged between 2-56 weeks.

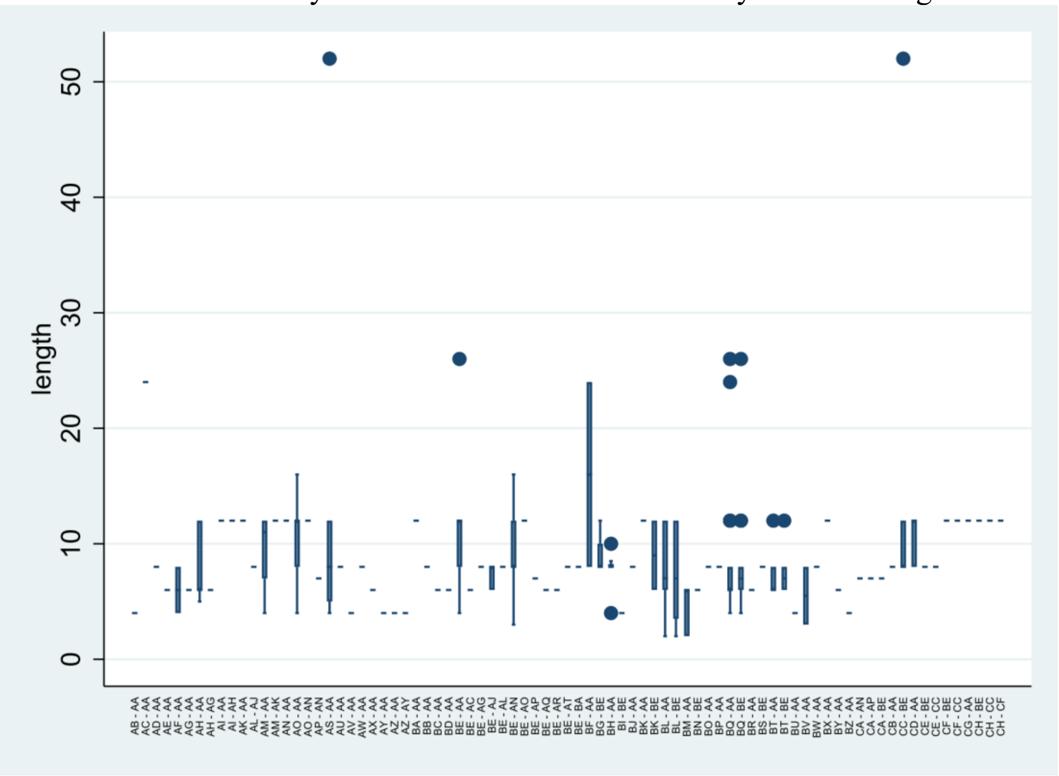

20.2. Assessment of transitivity-Baseline Severity (HAMD-17)
We examined the distribution of baseline severity on the depressive scale over the individual nutraceuticals. The mean depressive severity score was 20.15 on HAMD-17.

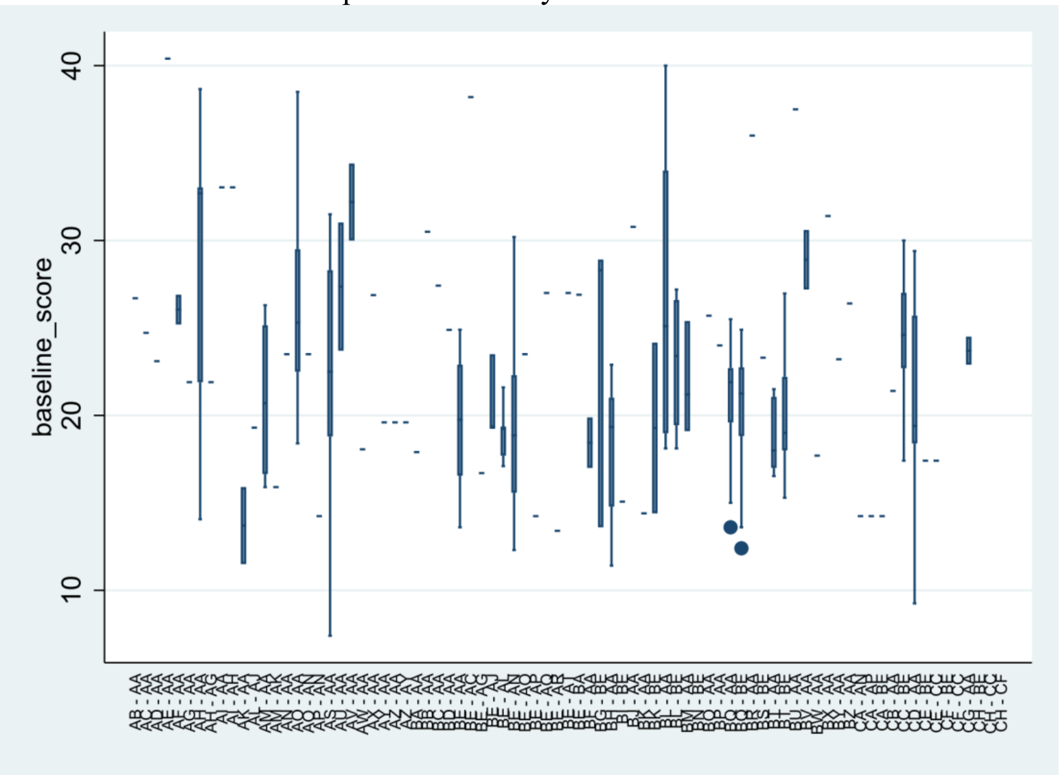

HAMD-17:17-item Hamilton Depression Rating Scale

20.3. Assessment of transitivity-Mean Age

We examined the distribution of mean age over the individual nutraceuticals. The overall median mean age was 43.5 with the range between 20-84.9.

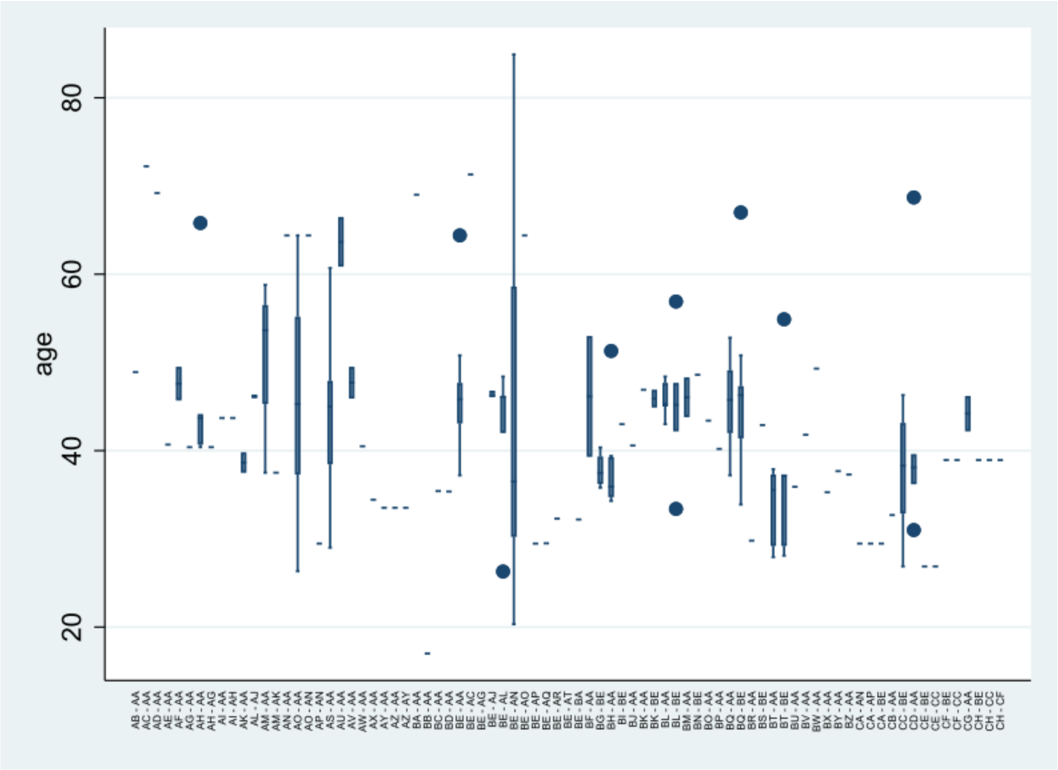

20.4. Assessment of transitivity-Publication year

We examined the distribution of publication year over the individual antipsychotics. The overall range was between 1971 and 2020 with a median of 2012. We examined publication year as a potential effect modifier, because it can be a proxy parameter for a number of factors that may have changed over the years (e.g. changes in trial design, monitoring, trial populations etc), that could have possibly influenced the effect sizes.

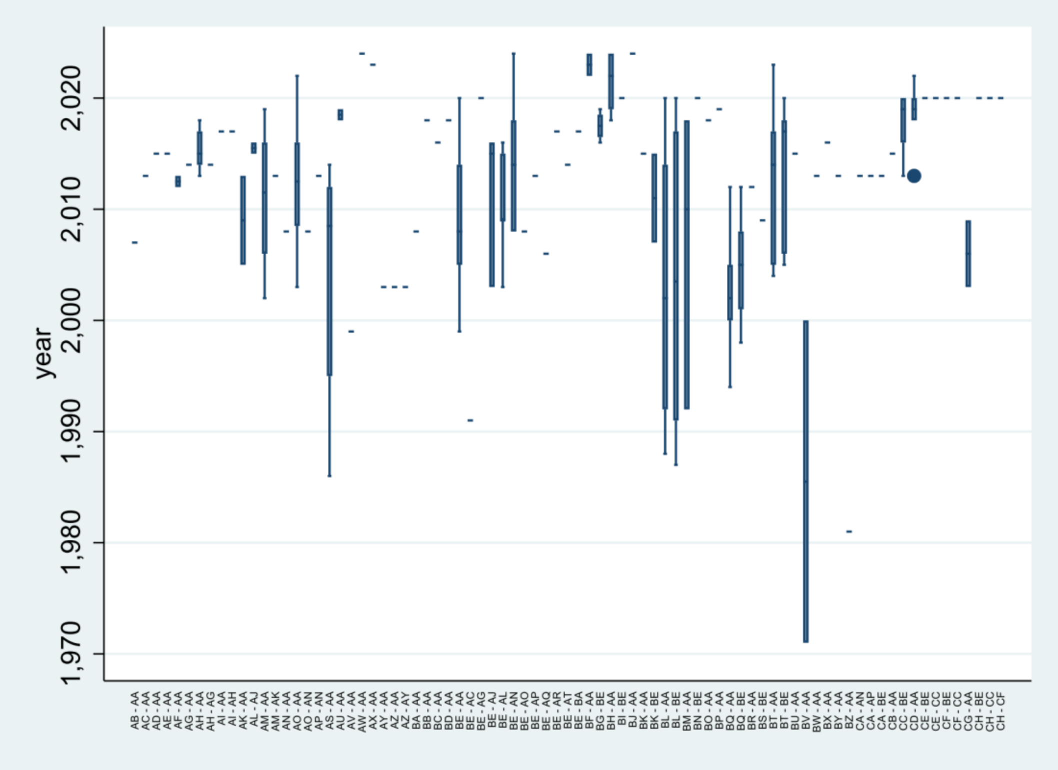

20.5. Assessment of transitivity-Sample size
We examined the distribution of sample size over the individual nutraceuticals.
The overall sample size range was 6 to 243 with a median of 30.Sample size is accounted for by more weight given to larger studies in meta-analysis. In addition, we inspected the effects of sample size as a potential effect modifier.

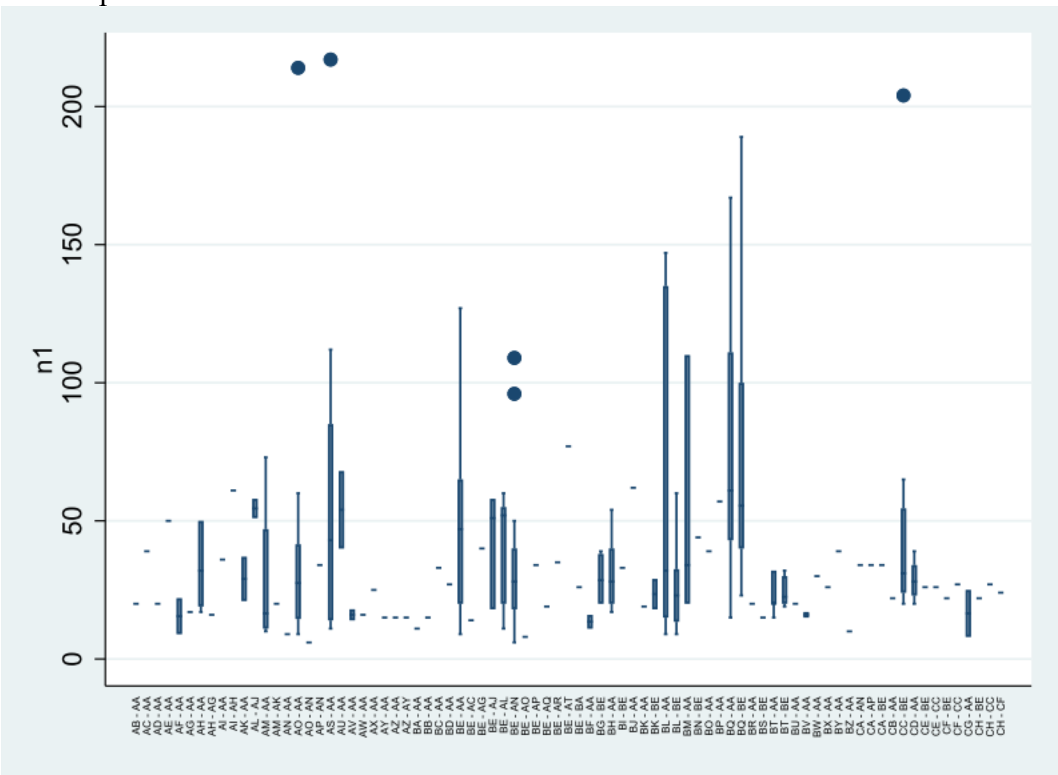

21 Unpublished clinical trials that meet our inclusion criteria.

ClinicalTrials.gov ID	Nutrient	Country	Year	Participants	Study duration	Remark
NCT01022632	Curcumin	India	2009-2010	60	6 weeks	No results posted
NCT04975100	Sarcosine+ADTs	India	2021-2023	60	8 weeks	No results posted
NCT02943096	Flavanol	United state	2016-2018	6	8 weeks	No results posted
NCT01672372	Methylated Vitamin B	United state	2011	60	8 weeks	No results posted
NCT04925440	Bifidobacterium Longum 1714	Ireland	2021-2022	168	8 weeks	No results posted
NCT04721249	D-serine +ADTs	Switzerland	2021-2023	44	6 weeks	No results posted
NCT02469545	Lactobacillus Plantarum 299V +ADTs	Poland	2014-2016	60	8 weeks	No results posted
NCT00480207	Folic Acid +Omega-3 Fatty Acids	Israel	2007-2008	15	12 weeks	No results posted
NCT04482296	Zinc+ADTs	Bangladesh	2020-2021	100	8 weeks	No results posted
NCT00256412	Eicosapentaenoic acid (EPA)	United state	2005-2007	24	4-10 weeks	No results posted
NCT04482296	Zinc+ADTs	United state	2019-2020	100	8 weeks	No results posted
NCT04880460	Magnesium	Bangladesh	2021	20	8 weeks	No results posted
NCT02972398	N-Acetyl Cysteine	China	2015-2023	200	12 weeks	No results posted
NCT00693680	Zinc+ADTs	Poland	2005-2006	60	12 weeks	No results posted
NCT03277586	Probiotics (Lactobacillus and Bifidobacterium)	Canada	2018-2020	28	16 weeks	No results posted
NCT00963196	Omega-3 Fatty Acids	United state	2009-2010	78	6 months	No results posted
NCT00313417	Creatines+ADTs	Israel	2007-2010	18	4 weeks	No results posted

NCT02057406	Omega-3 Fatty Acids	United state	2014-2016	108	12 weeks	No enough result
NCT04395183	5-HTP & Creatine	United state	2021-2023	43	8 weeks	With Result
NCT03732378	Omega-3 Fatty Acids	Pakistan	2017	70	12 weeks	No enough result
NCT00939718	Vitamin B12+ADTs	Pakistan	2009-2010	268	12 weeks	No enough result
NCT02072187	Vitamin D	Canada	2013-2017	9	12 weeks	No enough result
